# Trans-ancestry transcriptome-wide association and functional studies to uncover novel susceptibility genes and therapeutic targets for colorectal cancer

**DOI:** 10.1038/s41698-025-00906-9

**Published:** 2025-04-29

**Authors:** Lijuan Wang, Lidan Hu, Jing Sun, Jianhui Zhao, Siyun Zhou, Lexin Liu, Wei Yu, Yeting Hu, Dan Zhou, Xiangrui Meng, Zhongshang Yuan, Honghe Zhang, Susan Farrington, Maria Timofeeva, Kefeng Ding, Julian Little, Malcolm Dunlop, Evropi Theodoratou, Xue Li

**Affiliations:** 1 School of Public Health, the Second affiliated Hospital, Zhejiang University School of Medicine, Hangzhou, China; 2 Centre for Global Health, Usher Institute, University of Edinburgh, Edinburgh, UK; 3 Department of Nephrology, The Children’s Hospital, Zhejiang University School of Medicine, National Clinical Research Center for Child Health, Hangzhou, China; 4 Department of Colorectal Surgery and Oncology, Key Laboratory of Cancer Prevention and Intervention, Ministry of Education, The Second Affiliated Hospital, Zhejiang University School of Medicine, Hangzhou, China; 5 Division of Psychiatry, University College of London, London, UK; 6 Department of Biostatistics, School of Public Health, Cheeloo College of Medicine, Shandong University, Jinan, China; 7 Department of Pathology and Women’s Hospital, Zhejiang University School of Medicine, Hangzhou, Zhejiang, China; 8 Colon Cancer Genetics Group, Medical Research Council Human Genetics Unit, Institute of Genetics and Cancer, University of Edinburgh, Edinburgh, UK; 9 Danish Institute for Advanced Study (DIAS), Epidemiology, Biostatistics and Biodemography Research Unit, Institute of Public Health, University of Southern Denmark, Odense, Denmark; 10 Center for Medical Research and Innovation in Digestive System Tumors, Ministry of Education, Hangzhou, China; 11 Zhejiang Provincial Clinical Research Center for CANCER, Hangzhou, China; 12 School of Epidemiology and Public Health, University of Ottawa, Ottawa, Ontario, Canada; 13 Cancer Research UK Edinburgh Centre, Medical Research Council Institute of Genetics and Molecular Medicine, University of Edinburgh, Edinburgh, UK

**Keywords:** Colorectal cancer, Gene therapy, Translational research, Oncogenesis, Functional genomics

## Abstract

By integrating findings from large-scale omics analyses with experimental tests, this study aims to decipher susceptibility genes and the underlying biological mechanisms involved in the development of colorectal cancer (CRC). We first conducted a trans-ancestry transcriptome-wide association study (TWAS) among 57,402 CRC cases and 119,110 controls, aiming to examine how altered gene expression influences CRC risk in European and Asian populations. Then, functional experiments in (i) CRC cell lines and (ii) tumor xenografts were conducted to examine potential underlying mechanisms involved in colorectal carcinogenesis. Further, a drug sensitivity test was employed to explore possible clinical implications for CRC treatment. The TWAS identified 67 genes highly associated with CRC risk, 23 of which were novel findings. Functional annotation of variants within TWAS-identified loci revealed that the majority (93.6%) showed evidence of transcriptional regulatory mechanisms via proximal promoter or distal enhancer-promoter interactions. Among the identified susceptibility genes, splicing factor 3a subunit 3 (*SF3A3*) may act as an oncogene on the basis that overexpression of this gene was significantly associated with increased risk of CRC (P = 5.75 × 10^−11^). Further cell and animal experiments confirmed that *SF3A3* plays an oncogenic role in CRC development, and the underlying biological mechanism is likely to be related to its anti-apoptosis effect. The drug sensitivity test suggested that phenethyl isothiocyanate (PEITC) targeting *SF3A3* can inhibit CRC progression. This study identified novel CRC susceptibility genes and potential biological mechanisms of *SF3A3* involved in CRC development, providing important insight into the etiology and potential leads to the treatment of CRC.

## Introduction

Colorectal cancer (CRC) is one of the most frequently diagnosed cancers and the second cause of cancer-related deaths, with 1.93 million new cases and 0.94 million deaths in 2020^1^. Genetic factors play an important role in the etiology of CRC^2,3^. Over the last decade, large-scale genome-wide association studies (GWASs) have identified hundreds of common single nucleotide polymorphisms (SNPs) associated with CRC susceptibility^4^. However, single SNPs typically have only modest effects that account for a small fraction of the overall heritability of CRC. Moreover, most of these GWAS-identified risk loci reside in non-coding regions, and their functional basis is little investigated. Therefore, studies are warranted to decipher the underlying biological mechanisms through which the identified variants exert their effects on CRC risk.

Most GWAS-identified risk variants play regulatory roles in gene expression^5^. Expression quantitative trait loci (eQTL) studies that investigated the associations between genetic variants and gene expression have been increasingly integrated with the results from GWAS to help understand and interpret the biological functions of susceptibility variants and genes^6–9^. One such approach is the transcriptome-wide association study (TWAS), which leverages expression reference panels (eQTL cohorts with expression and genotype data) and GWAS datasets to systematically investigate the role of gene expression in modulating disease risk^10^. Briefly, TWAS estimates the joint effects of multiple functional variants within the cis-genotype region surrounding an expressed gene to predict the cis-heritable component of a gene’s expression, which in turn can be associated with disease status. TWASs have proved useful in prioritizing candidate causal genes, because (i) they take into account that combining cis-SNPs into a single predictor may capture heterogeneous signals better than individual SNPs or cis-eQTLs, and (ii) reduce the multiple-testing burden^11^. In addition, studies have shown that eQTLs with large effects tend to regulate gene expression in multiple tissues^12^. Thus, advanced algorithms to generate reference panels across tissues^13–15^ to perform powerful across-tissue TWAS have been developed, aiming to improve the imputation efficiency and accuracy of TWAS by enlarging the sample size of the genotype-expression model. Furthermore, efforts have increasingly focused on deciphering the biological mechanisms underlying the identified susceptibility variants and genes by conducting functional experiments^16,17^. Currently, several single- or across-tissue TWASs of CRC have been performed in populations derived from single^18,19^ or multiple ancestries^20^. However, it remains unclear how the identified susceptibility signals modify CRC risk beyond changes in gene expression. Moreover, it is also relevant to explore the potential for TWAS findings to inform CRC treatment development.

In the present study, we applied a single- and across-tissue TWAS strategy to detect potential susceptibility genes for CRC in 57,402 cases and 119,110 controls of Asian or European ancestries. We found that over expression of gene-splicing factor 3a subunit 3 (*SF3A3*), which encodes subunit 3 of the splicing factor 3a protein complex, was positively associated with CRC risk. To further clarify the biological mechanisms of *SF3A3* in colorectal tumorigenesis, we performed in vitro and in vivo experiments. To investigate whether *SF3A3* could be a drug target for CRC treatment, we carried out a drug sensitivity test on CRC cells by intervening with phenethyl isothiocyanate (PEITC), a potential chemopreventive agent that targets the protein coded by SF3A3.

## Results

### TWAS associations

**Fig. 1:**
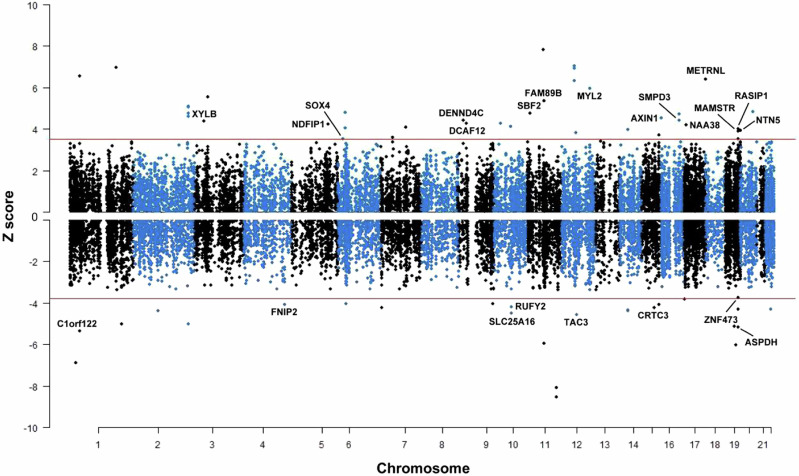
Manhattan plot of significant susceptibility genes identified by single- and across-tissue TWASs of CRC. Each point corresponds to an association test between the expression of gene with CRC risk, with physical position plotted on x-axis and TWAS Z value plotted on y-axis. The red lines indicate the FDR-corrected significance level. The names of novel CRC susceptibility genes are shown.

In single-tissue and across-tissue TWAS analyses, we highlighted genes that showed high evidence of correlating with CRC risk based on the following criteria: (i) genes reached TWAS significance threshold (FDR < 0.05); (ii) genes that were colocalized (colocalization PP4 > 0.75); (iii) genes that were conditionally independent in the identified susceptibility loci. As a result, a total of 67 genes with high confidence were considered as to be most relevant to CRC, 23 of them have not been identified by any of the previously published TWAS studies (Fig. 1)^18–20^.

**Fig. 2:**
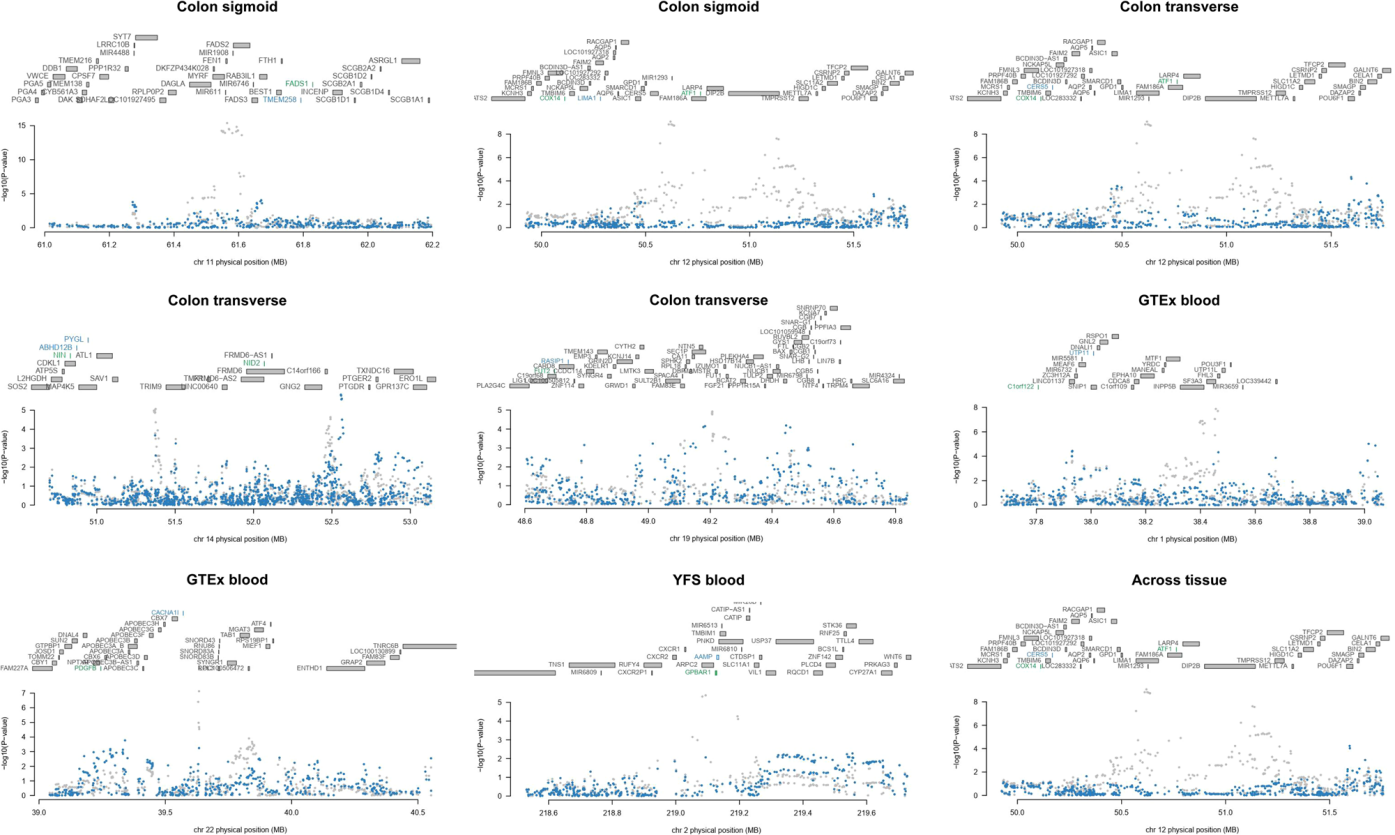
Gene conditional results of TWAS hits. The top panel in each plot highlights all genes in the region. The marginally associated TWAS genes are shown in blue and the jointly significant genes are shown in green. The bottom panel shows a regional Manhattan plot of the GWAS data before (gray) and after (blue) conditioning on the predicted expression of the green genes.

Briefly, in single-tissue TWAS, 295 significant associations between genetically predicted gene expression and CRC risk were observed after FDR correction, representing 195 unique genes (Supplementary Table 2). Of these, 103 associations resulting in 62 unique genes were considered as putative CRC susceptibility genes with a colocalization PP4 > 0.75, which means that the effects of the variants within TWAS-identified susceptibility loci on CRC risk are likely to be mediated by transcription changes of their corresponding genes (Supplementary Table 3). We performed conditional analyses for TWAS-identified loci in which multiple significant signals were identified in the same locus (defined as a 1 Mb window). For example, the results revealed that *FADS1* (11q12.2) was an independent signal at its locus while the association of *TMEM258* was mainly owing to the correlated predicted expression in the region (Fig. 2). For the same signal identified in multiple tissues, we reported the one with the lowest association *P* value (Supplementary Table 4). 17 signals have not been reported by previous studies.

In across-tissue TWAS, we identified 101 significant associations with a FDR < 0.05, 27 of which were verified by colocalization analysis (PP4 > 0.75). One of the 27 associations (with *CERS5* in 12q13.12) was not an independent signal and was therefore excluded from further analysis. As a result, 26 signals with high evidence were identified, of which six were novel findings independent from previous results and that identified in our single-tissue TWAS (Supplementary Table 5).

### Pathway enrichment analysis

Pathway enrichment results revealed that CRC susceptibility genes are principally enriched in genes (i) regulating immune-related biological processes such as Th1 and Th2 cell differentiation and intestinal immune network for IgA production, (ii) involved in encoding proteins in cellular structures such as the actin cytoskeleton and MHC protein complex, and (iii) modulating signaling pathways such as the Rho GTPases (Supplementary Table 6). Co-expression and pathway analysis of novel CRC susceptibility genes showed that over half (13/23) are involved in the regulation of p53 activity, 15 in cell cycle events (mitosis and apoptosis), and 11 in modifying signaling by interleukins (Supplementary Table 7). All these findings provide insights into the potential underlying pathogenic mechanisms through which these genes may influence CRC risk.

### Functional annotation

Given that the predicted genetic component of expression is derived from the joint effects of cis-SNPs surrounding the gene, we performed functional annotation of the variants in order to explore the underlying regulatory mechanisms. We first identified the GWAS SNP with the most stringent *p* value for each TWAS-identified locus (Supplementary Table 8). Then, we performed functional annotation of variants in strong LD (R^2^ > 0.8) with these SNPs by mapping them to multiple regulatory elements. A total of 1398 putative functional variants were included, of which 1,016 (72.7%) were mapped to promoter regions indicating that these variants would most likely play a regulatory role in relation to their located genes. We further examined whether the genes could be regulated by putative functional variants located in enhancer regions via long-distance promoter-enhancer interactions, and found that 1285 variants (91.9%) were mapped to enhancer regions. In addition, among these 1308 variants (93.6%) showed evidence of regulation via proximal promoter or distal enhancer-promoter interactions, 631 of them (48.2%) were further supported with the evidence of their functional location in regions of DHS or TFBS (Supplementary Table 9, Supplementary Fig. 1).

### *SF3A3* functions as an oncogene in CRC cells

**Fig. 3:**
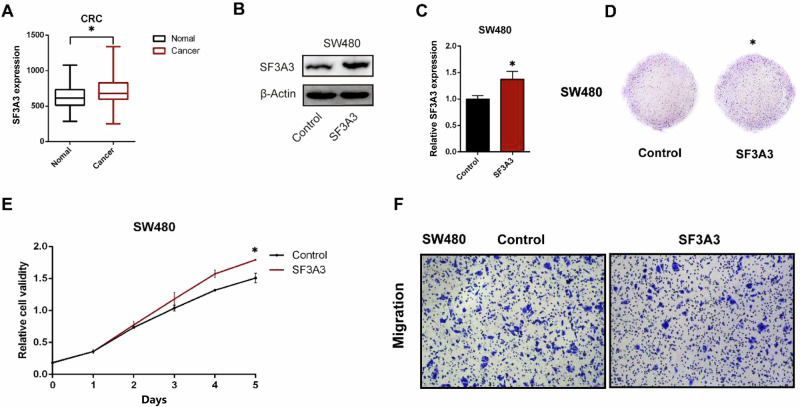
*SF3A3* is over-expressed in CRC cells and promotes cell proliferation. **A** Differential expression of *SF3A3* in CRC tumor and normal tissues based on TCGA transcriptome data. **B** Western blot of *SF3A3* protein levels in *SF3A3* overexpression (*SF3A3*-OE) SW480 cells. **C** Relative mRNA expression levels of *SF3A3* in *SF3A3*-OE SW480 cells. **D** The colony-forming ability of *SF3A3*-OE SW480 cells was determined by a colony formation assay. **E** The prominent effect of *SF3A3*-OE on proliferation of SW480 cells was detected using a CCK-8 assay. **F** Transwell assay for evaluating the migration ability of *SF3A3*-OE SW480 cells. All experiments were repeated three times and we used the mean value to present. All *, *P* < 0.05, calculated by the two-tailed Student *t*-test.

The TWAS results showed that high expression of one particular gene, *SF3A3*, which encodes an important splicing factor, was significantly associated with increased risk of CRC (*P* = 5.75 × 10^−11^, Supplementary Table 4), consistent with previous findings that indicated an oncogenic role for *SF3A3* in tumor progression^21–24^. *SF3A3* plays a crucial role in the process of alternative splicing (AS), which is an important posttranscriptional mechanism contributing to generating distinct mRNA and protein isoforms^25^ and some splicing factors has been reported as oncoproteins when overexpressed and can promote cell proliferation and increase tumorigenic capacity of colon cancer cells^26^. Consistently, based on TCGA transcriptome data, we found that the expression of *SF3A3* in CRC patients was significantly higher in tumor tissues than that in adjacent normal tissues (*P* < 0.05, Fig. 3A). Then, to investigate the cellular function of *SF3A3*, we performed in vitro functional experiments using both an overexpression and a siRNA strategy. The expression levels of *SF3A3* in different CRC cell lines were compared, as a result, SW480 and HCT116 cell lines were selected for overexpression and knockdown experiments, respectively (Supplementary Fig. 2). The expression levels of *SF3A3* was significantly increased in SW480 cell line (*P* < 0.05, Fig. 3B, C). In addition, colony formation of SW480 cells was significantly enhanced over that of the control vehicle (*P* < 0.05, Fig. 3D). Cell viabilities were significantly increased over those of the control vehicle in the CCK8 assay (*P* < 0.05, Fig. 3E), indicating that overexpression of SF3A3 promoted the proliferation of CRC cells. Further, cell migration was measured using Transwell assays. We observed that SF3A3 overexpression was not associated with CRC cell migration when compared to the control vehicle (*P* > 0.05, Fig. 3F).

**Fig. 4:**
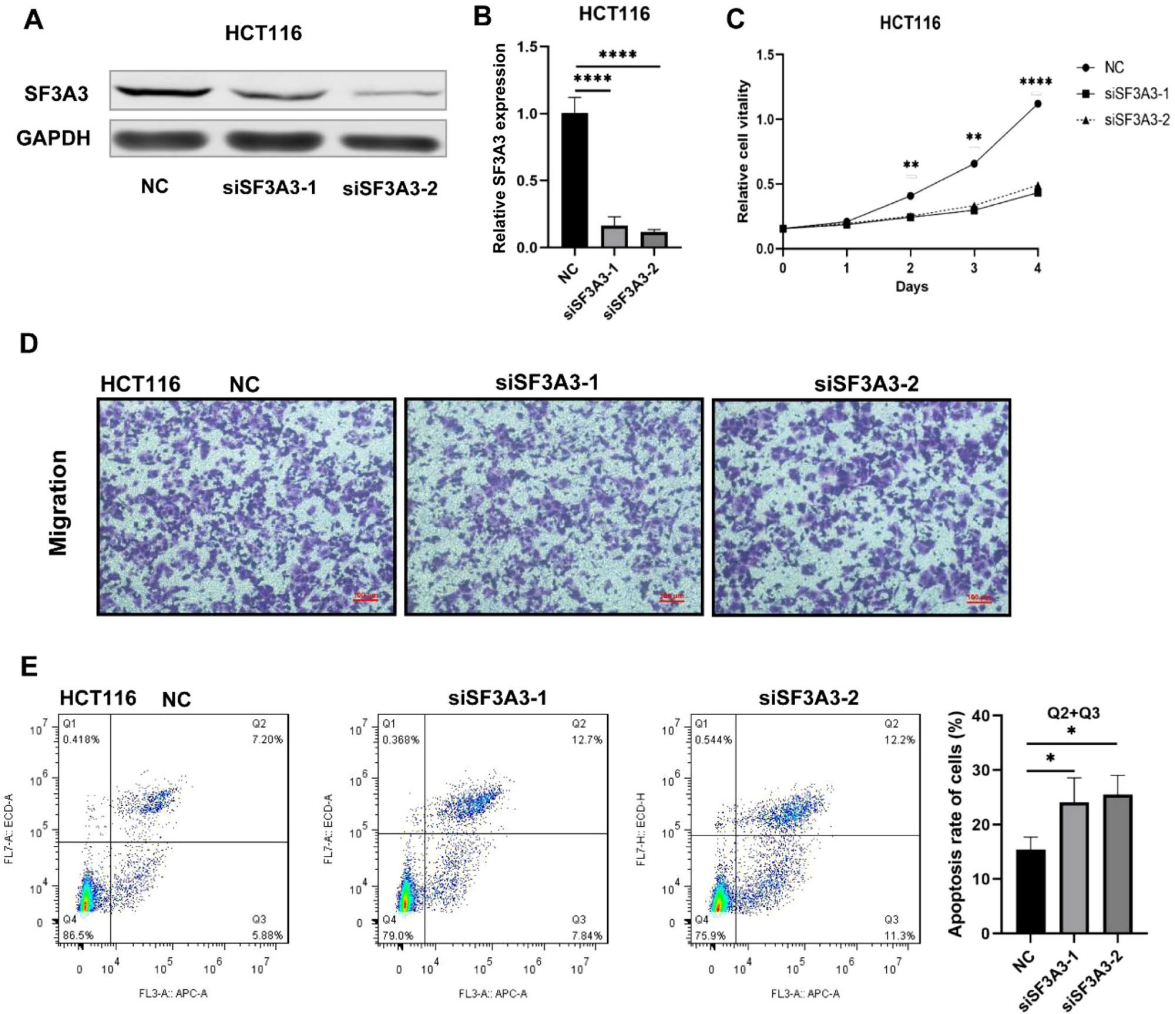
Knockdown of *SF3A3* promotes apoptosis in CRC cells. **A** Western blot of *SF3A3* protein levels in HCT116 cells with silencing of *SF3A3* by siRNA. **B** Relative mRNA expression levels of *SF3A3* in *SF3A3* knockdown (*SF3A3*-KD) HCT116 cells. **C** The prominent effect of *SF3A3*-KD on proliferation of HCT116 cells was detected using a CCK-8 assay. **D** Transwell assay for evaluating the migration ability of *SF3A3*-KD HCT116 cells. **E** The prominent effect of *SF3A3*-KD on apoptosis of HCT116 cells was detected by flow cytometry. All experiments were repeated three times and we used the mean value to present. All *P < 0.05; **P < 0.01; ****P < 0.001, calculated by the two-tailed Student *t*-test.

We performed knockdown experiments in the HCT116 cell line using two siRNAs, and observed that transfection with these two siRNAs were able to reduce the endogenous mRNA level of *SF3A3* (*P* < 0.001, Fig. 4A–C). Similarly, knockdown of *SF3A3* was not associated with CRC cell migration (Fig. 4D). Furthermore, the apoptosis rate in the CRC cells with *SF3A3* knockdown was significantly increased as compared with that in the control group (*P* < 0.05, Fig. 4E). All these findings suggested that knockdown of *SF3A3* could induce CRC cell apoptosis, thus suppressing tumor growth.

### PEITC inhibits tumor progression in CRC cells via *SF3A3*

To explore whether *SF3A3* could be considered as a therapeutic target and provide evidence for its clinical implication in CRC, we consulted the DrugBank database with the predicted relationships between drugs and targets^27^, and found that *SF3A3* might be a drug target of phenethyl isothiocyanate (PEITC), a naturally occurring compound found in some cruciferous vegetables, which is known to possess anti-cancer properties. To investigate whether PEITC plays an anti-cancer role in CRC and whether it exerts effect through inhibiting *SF3A3*, we performed in vitro drug sensitivity test. The IC_50_ of PEITC in CRC SW480 cells was 131.9 µM (Supplementary Fig. 3A). In addition, colony formation and CCK8 assay results demonstrated an obvious anti-cancer effect of PEITC on CRC (*P* < 0.05, Supplementary Fig. 3B, 3C). We performed high-throughput virtual screening of PEITC and *SF3A3*, and the results showed that the active site of PEITC was closely bound to *SF3A3* (Supplementary Fig. 4A–C). Further CCK8 experiments identified that the anti-cancer drug efficacy of PEITC in CRC cells was significantly improved after *SF3A3* overexpression (*P* < 0.05, Supplementary Fig. 4D, 4E). Our findings revealed that PEITC inhibits CRC progression by targeting *SF3A3*, which could make it a promising potential CRC treatment.

### *SF3A3* promotes the growth of xenografts in vivo

**Fig. 5:**
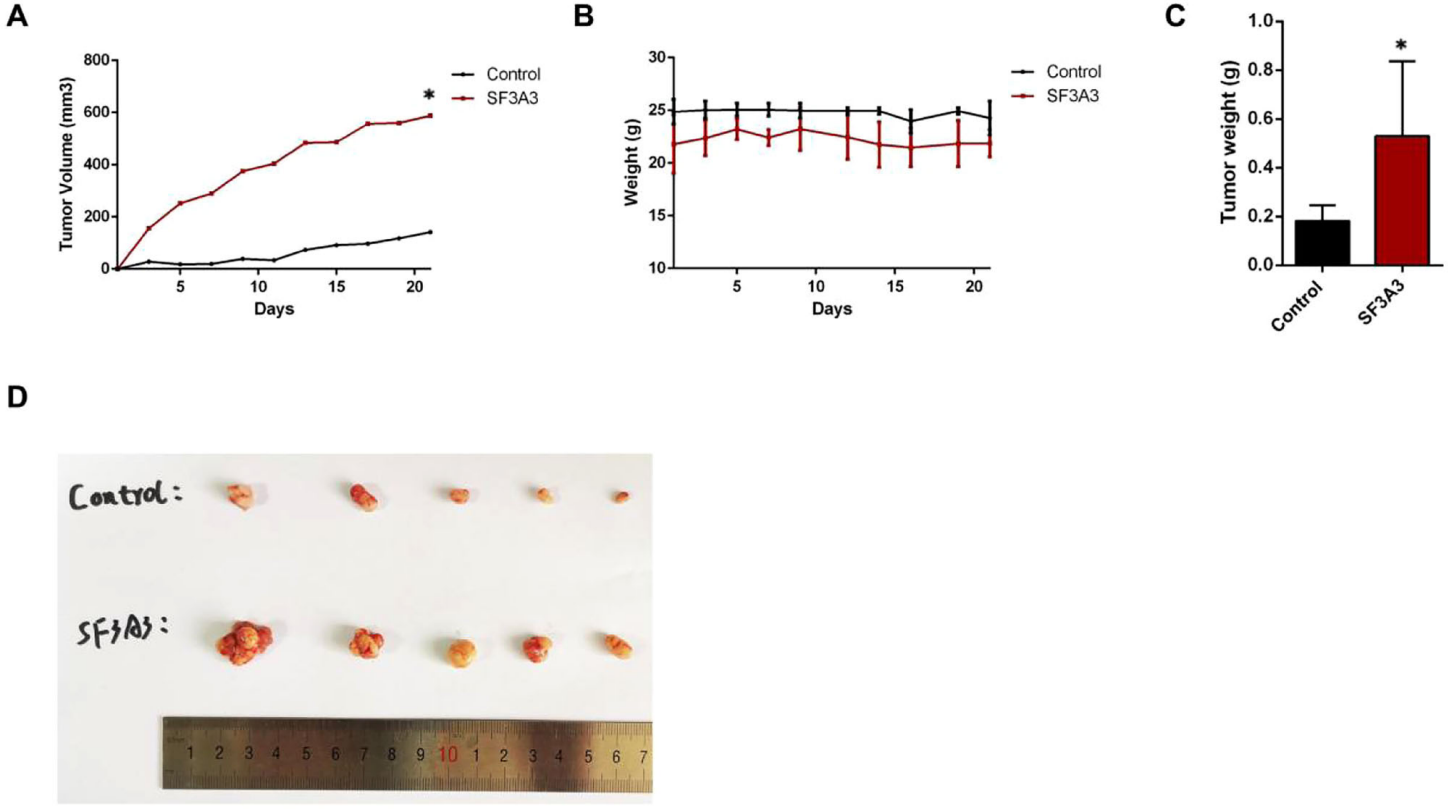
*SF3A3* promotes tumor progression in vivo. **A** Comparison of tumor volumes between control and *SF3A3*-OE mice. Tumor volume was recorded by a measurement of tumor length, height and width three times a week during tumor proliferation, and calculated as length × width × height/2. Quantification of tumor volume was performed three times a week during tumor proliferation. **B** Comparison of body weights between control and *SF3A3*-OE mice. Quantification of body weight was performed three times a week during tumor proliferation. **C** Comparison of tumor weights between control and *SF3A3*-OE mice at day 20 after the anesthetic execution of mice. **D** Xenograft tumor burdens were compared between mice inoculated with control and *SF3A3*-OE cells. All *P < 0.05, calculated by the two-tailed Student *t*-test.

To confirm that *SF3A3* is capable of promoting the progression of CRC, we developed a xenograft model by the subcutaneous injection of SW480 *SF3A3*-OE and control cells in nude mice. We showed that the injected *SF3A3*-OE cells markedly increase tumor volume and weight when compared with control cells (*P* < 0.05, Fig. 5). Collectively, as a potential oncogene in CRC, the investigations of functional mechanisms of *SF3A3* both in vitro and in vivo experiments could deepen the understanding of CRC etiology.

## Discussion

In summary, our trans-ancestry TWAS together with following colocalization and conditional analyses identified 67 susceptibility genes highly associated with CRC risk, 23 of which are novel findings. Both in vitro and in vivo functional assays revealed that the putative oncogene *SF3A3* plays a role in CRC development. Further a drug sensitivity test suggested that PEITC targeting *SF3A3* could be a potential candidate for CRC treatment development.

The identification of 67 transcriptome-wide significant genes represented independent signals showing high evidence of associations between genetically determined gene expression and CRC susceptibility. Of note, one particular gene, *SF3A3*, was identified as a putative oncogene involved in CRC development. *SF3A3*, encodes subunit 3 of the splicing factor 3a protein complex, which is necessary for the in vitro conversion of 15S U2 snRNP into an active 17S particle that performs pre-mRNA splicing^28^. It has been reported that *SF3A3* post-transcriptional regulation affects splicing of transcripts involved in mitochondrial dynamics, thus favoring cancer-associated metabolic reprogramming and stem-like properties that boost MYC-induced breast tumorigenesis in vivo^21^. In addition, *SF3A3* has been validated as an inhibitor of p53 activity in multiple non-small cell lung cancer (NSCLC) cell lines. Thus suppression of *SF3A3* can upregulate the expression of anti-oncogene TP53, thereby inducing cell cycle arrest and death of tumor cells^24^. However, the role of *SF3A3* in CRC has not yet been defined. Therefore, we conducted both in vitro and in vivo functional experiments and proposed that *SF3A3* plays an oncogenic role in CRC by promoting cell proliferation and knockdown of this gene has the potential to induce cell apoptosis. Consistently, a recent study conducted by Chang, J. et al. found that the reduction of *SF3A3* could cause G1/S cell cycle arrest, and thus resulting in proliferation inhibition and cell death in acute promyelocytic leukemia cells^29^. Taken together, we suggested that the oncogenic effect of *SF3A3* on CRC development may be mediated through its anti-apoptotic function, thus leading to colorectal carcinogenesis.

Furthermore, to explore whether *SF3A3* could be considered as a potential drug target for CRC clinical treatment, we referred to the DrugBank database and found that the *SF3A3* gene is a putative target for PEITC, which has been reported to inhibit cancer cell growth through cell-cycle arrest and induction of apoptotic events in various human cancer cells models including colorectal cancer cells^30^, prostate cancer cells^31^, osteogenic sarcoma cells^32^, and oral cancer cells^33^. In this study, we observed an anti-cancer effect of PEITC on CRC cells. In addition, high-throughput virtual screening predicted a close binding of the active site of PEITC to *SF3A3* and the subsequent experimental findings demonstrated that PEITC may exert its effect through inhibiting the expression of *SF3A3*. PEITC has been used in clinical trials studying the prevention and treatment of leukemia^34^ and lung cancer^35^, but has not yet been tested and applied in CRC treatment. Our findings provide evidence suggesting the potential value of clinical trials investigating the anti-cancer effect of PEITC on CRC.

This study has several strengths and limitations. First, we conducted a meta-analysis of CRC GWASs to increase statistical power and identify genetic variants associated with CRC across diverse populations. The strength of this method lies in its ability to detect robust associations by leveraging large sample sizes and genetic diversity. However, limitations include potential heterogeneity between studies, which can introduce bias, and the reliance on summary-level data that may not capture more nuanced effects or rare variants. Second, based on the combined GWAS summary statistics, we applied a trans-ancestry TWAS strategy to identify susceptibility genes associated with CRC risk by integrating genetic data with gene expression data, allowing for insights into the underlying molecular mechanisms of CRC. Besides, the study design gave suggestions for shared and unique genetic etiologies across Asian and European ancestries. In TWAS analysis, we used expression weights from the GTEx database as references to generate genetically predicted gene expression for CRC GWAS population. Due to the limited sample size of the reference panels, the statistical power of TWAS in detecting additional susceptibility signals may be reduced. Third, the following colocalization and gene conditional analyses were performed to further prioritize CRC-associated genes with strong evidence by testing if both gene expression and the identified TWAS associations were driven by the same genetic signals. While these methods can be constrained by sample size, potential biases in the reference datasets, and the difficulty of distinguishing between direct causal effects and those driven by linkage disequilibrium. Last but not least, both functional experiments and drug sensitivity test were carried out in this study, helping explore the biological mechanisms of *SF3A3* underlying CRC development and provide a theoretical basis for future clinical application of PEITC targeting *SF3A3* in CRC treatment. However, the results from preclinical models may not fully recapitulate human CRC biology, highlighting the need for further validation of the findings in clinical settings to confirm the efficacy and safety of PEITC targeting *SF3A3* in CRC treatment.

In summary, our study identified 67 susceptibility genes highly associated with CRC risk, 23 of which have not been reported in previous studies. Subsequent functional experiments suggested that gene *SF3A3* plays an oncogenic role in the development of CRC and the underlying biological mechanism is related to the inhibition of cell apoptosis. In addition, PEITC targeting *SF3A3* could be considered as a promising anti-cancer drug for CRC treatment, providing important insight into clinical application of our research findings. Future clinical trials are warranted to assess the pharmacological effects of PEITC among CRC patients.

## Methods

Collection of patient samples and associated clinico-pathological information was undertaken with written informed consent and relevant ethical review board approval at respective study centers in accordance with the tenets of the Declaration of Helsinki. Specifically: (i) UK National Cancer Research Network Multi-Research Ethics Committee (02/0/097 [NSCCG], 01/0/5 [SOCCS], 05/S1401/89 [GS:SFHS], LREC/1998/4/183 [LBC1921], 2003/2/29 [LBC1936], 17/SC/0079 [CORGI] and 07/S0703/136 [SCOT]) and North West Multi-centre Research Ethics Committee (11/NW/0382); (ii) South East Ethics Committee MREC (03/1/014); (iii) the Ethical Committees of Medical University of Vienna (MUW, EK Nr. 703/2010) and “Ethikkommission Burgenland” (KRAGES, 33/2010); (iv) the Ethical Committee of Hospital District of Helsinki and Uusimaa (HUS/408/13/03/03/09); (v) the Ethical Committees of Shanghai Cancer Registry and Sun Yat-Sen University Cancer Center; (vi) the Ethical Committees of University of Tokyo, RIKEN and Aichi Cancer Center; (vii) the Ethical Committees of Korea Central Cancer Registry, Korean National Cancer Center and Chonnam National University Hwasun Hospital.

### Study design, populations and datasets

Both European and Asian populations were included. Genetic associations with CRC were obtained from large-scale GWAS meta-analyses consisting of 34,627 cases and 71,379 controls for European ancestry, and 22,775 cases and 47,731 controls for Asian ancestry^36,37^. Details on the included studies are presented in Supplementary Table 1. All studies were approved by their respective institutional review boards and conducted with appropriate ethical criteria in each country and in accordance with the Declaration of Helsinki.

**Fig. 6:**
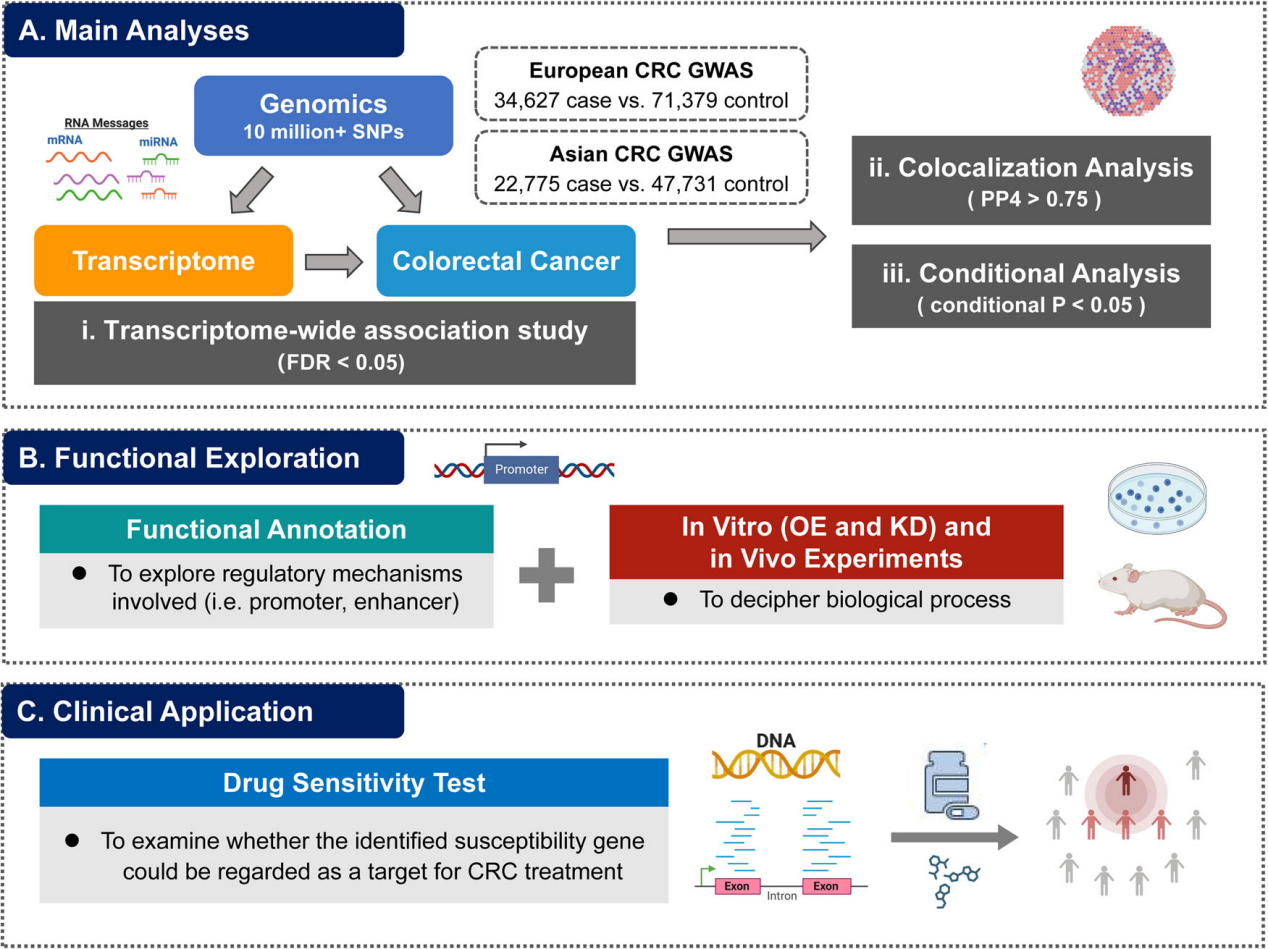
Flowchart of the study design. **A** Main analyses including trans-ancestry TWAS, colocalization and conditional analyses were conducted to identify significant susceptibility genes for CRC. **B** Functional annotation of variants within TWAS-identified loci and in vitro (overexpression and knockdown) and in vivo experiments were performed to further characterize the underlying biological mechanisms involved in CRC development. **C** Drug sensitivity test was carried out to seek clinical application of TWAS findings for future CRC treatment.

We first conducted a meta-analysis of genome-wide association data from all available CRC GWASs conducted in populations of European and Asian origin (totally 57,402 cases and 119,110 controls) by using the inverse variance-weighted fixed-effect model implemented in METAL software^38^. Variants with a heterogeneity *I*^2^ > 75% were excluded from the combined dataset, leaving a total of 11,253,900 variants for the subsequent main analysis including TWAS, colocalization and conditional analyses. Then, functional annotation of the identified TWAS loci and in vitro and in vivo experiments were carried out to further decipher potential biological mechanisms involved in CRC development. Last, drug sensitivity test was performed to provide insights into the application of TWAS findings for future CRC treatment. More details regarding the design of this study can be found in Fig. 6.

### TWAS analysis

We performed TWAS analysis using the FUSION software^39^ to identify susceptibility genes for CRC. First, we used FUSION to estimate the heritability of genes explained by cis-SNPs (SNPs within 1 MB region surrounding the transcription start site) based on individual genotype data derived from reference panels and then restricted analysis to include cis-heritable genes (*P*_heritability_ < 0.01). Second, for these eligible genes, we calculated the effect sizes of cis-SNPs associated with gene expression (i.e., expression weights) using the following predictive linear models: elastic net, least absolute shrinkage and selection operator (LASSO), genomic best linear unbiased prediction (GBLUP), and Bayesian sparse linear mixed model (BSLMM). We used the prepackaged expression weights in colon (sigmoid and transverse) and blood tissues derived from the genotype-tissue expression (GTEx) database (version 8)^40^ together with that in whole blood tissues from the Netherlands Twin Register (NTR)^41^ and the Young Finns Study (YFS)^42^ as reference panels. Cross-tissue weights generated through sparse canonical correlation analysis (sCCA) of data from the GTEx database (version 8) were also employed^13^. Third, we used FUSION to impute the cis-genetic component of expression in large scale GWAS data based on expression weights from the training data while accounting for linkage disequilibrium (LD) among SNPs, and tested the associations between the predicted gene expression and CRC risk. For each gene, we estimated the z-score of the expression and a complex trait (Z_TWAS_) as a linear combination of the vector of GWAS summary Z scores at a given cis-locus with expression weight vector W derived from the reference panels. The imputed z-score of expression and trait (WZ) has variance WVW^t^, where V is a covariance matrix across SNPs at the locus (i.e., LD) and W^t^ is the LD-adjusted weight vector learned from the gene expression data, defined as:

1 $${Z}_{{TWAS}}=\frac{{WZ}}{\sqrt{{WV}{W}^{t}}}$$

We applied a Benjamini-Hochberg correction to account for multiple testing in each tissue and associations with FDR < 0.05 were considered as statistically significant.

### Colocalization analysis

Colocalization tests of GWAS signals and TWAS-identified associations were performed using the “coloc” R package^43^. This Bayesian approach estimates the posterior probability (PP) that associations within a locus for two outcomes are driven by a shared causal variant. It thus enables to distinguish between associations driven by both transcription and CRC (PP4), independent transcription/CRC association (PP3), association for CRC only (PP2), association for transcription only (PP1) and no association (PP0). The significant colocalized signals was determined based on the threshold of PP4 > 0.75.

### Conditional analysis

Conditional analysis was conducted to determine whether multiple associated genes within a given locus represent independent associations or a single association owing to correlated predicted expression between genes. This analysis jointly estimates the effect of all significant genes within each locus by using residual SNP associations with CRC after accounting for the predicted expression of other genes. This process identifies which genes represent independent associations (termed jointly significant) and which genes are not significant when accounting for the predicted expression of other genes in the region (termed marginally significant). This analysis was implemented in the FUSION software^39^. Then, we highlighted genes that showed high evidence of correlating with CRC risk based on the following criteria: (i) genes reached TWAS significance threshold (FDR < 0.05); (ii) genes that were colocalized (colocalization PP4 > 0.75); (iii) genes that were conditionally independent in the identified susceptibility loci.

### Pathway enrichment analysis

To explore potential pathogenic mechanisms of the identified genes involved in the development of CRC, we performed pathway enrichment analysis of susceptibility genes identified by either single-tissue or across-tissue TWASs using Enrichr^44^. In addition, to characterize biological function of the identified highly associated susceptibility genes, we used linear regression model to detect their co-expressed genes based on transcriptome data in colon tissue from the GTEx database (version 8). Significant co-expressed genes were then included in the pathway enrichment analysis using the Kyoto Encyclopedia of Genes and Genomes (KEGG), Gene Ontology (GO), Reactome and MSigDB Hallmark databases as references implemented in R package “clusterProfiler”^45^. The Benjamini-Hochberg method was used for the multiple testing correction and FDR < 0.05 was set as a cut-off threshold.

### Functional exploration

To search for additional evidence for significant associations identified from TWAS, we explored regulatory mechanisms for the best GWAS SNPs (the SNP that has the lowest association *P* value in the locus) as well as variants in strong LD (*R*^*2*^ > 0.8) with these SNPs in the identified susceptibility loci. For each SNP, we investigated whether it was mapped to a region of histone modifications (i.e., promoter or enhancer), DNase I hypersensitive (DHS) or transcription factor binding sites (TFBS) using chromatin state data from the database HaploReg v4^46^. The regulatory motif alterations were also used to predict SNPs that may influence transcriptional regulation. In addition, we made predictions about the functional consequences of variants (i.e. synonymous, missense or nonsense, changing the consensus sequence at splice sites, or residing in introns or UTRs) based on the dbSNP database^47^.

### Construction of CRC cell lines with *SF3A3* overexpression and knockdown

Our study identified that *SF3A3* expression was significantly associated with CRC risk. To characterize the biological function of this gene, we conducted qPCR experiments to compare its relative expression levels in five CRC cell lines including HCT116, LoVo, RKO, SW480 and SW620. *SF3A3* showed lower expression levels in SW480 cell line and higher expression levels in HCT116 cell line, and thus these cell lines were selected for overexpression and knockdown experiments, respectively. The cell lines (SW480 and HCT116) were seeded in tissue culture dishes and grown for ~24 h to reach 50-60% confluence. The medium was then changed to DMEM containing 30 µg/mL polybrene and the appropriate lentiviruses encoding control overexpression vectors (pLenti-CMV-GFP-Puro-SF3A3; YuanJing, Guangzhou, China) were added and incubated at 37°C for 24 h. The medium was exchanged for fresh DMEM medium and the cells were further cultured for an additional 24 h. Stable *SF3A3*-overexpression (*SF3A3*-OE) cell lines were selected by growth in medium containing 10 µM puromycin. Two small interfering RNAs (siRNAs) targeting the *SF3A3* gene (siSF3A3-1 and siSF3A3-2) and nontargeting siRNA (siNC) were designed and synthesized by Shanghai GenePharma Co., Ltd. The sequences of siSF3A3-1 and siSF3A3-2 are 5′-GUGCCAAUGUCAGUGGAAUTT-3′ and 5′-CUGGCUGUAUAAGCUUCAUTT-3′, respectively. The efficiency of overexpression and knockdown were verified by RT-qPCR and Western blot analysis, respectively. The experiments were performed three times. Two-tailed Student *t*-test was used to assess differences in gene expression patterns of *SF3A3* between colorectal cancer tissues and normal tissues and a nominal *P* value < 0.05 was considered statistically significant.

### Colony formation assay

*SF3A3*-OE and *SF3A3*-knockdown (*SF3A3*-KD) CRC cells were trypsinized into a single cell suspension and seeded into 6-well culture plates at 10^3^ cells/well for 10–15 days. In drug sensitivity test, CRC cells were treated with phenethyl isothiocyanate (PEITC) for 10 days (wild-type [WT] cells) or 14 days (*SF3A3*-OE cells). At the end of the incubation, the cells were washed and fixed with 4% paraformaldehyde for 30 min at room temperature, and further stained with crystal violet solution (Beyotime, Jiangsu, China). Clones containing at least 50 cells were considered one formation. The experiments were performed three times.

### Colorectal cell proliferation and migration assay

Cells were seeded in 96-well plates at 2 × 10^4^ cells/well and incubated for 96 h. Every 24 h, cell numbers were determined using a Cell Counting Kit-8 (CCK8; Beyotime, Jiangsu, China) according to the manufacturer’s instructions. Briefly, cells were plated into 96-well plates in triplicate and cultured on different days, then the medium was changed to a solution of 100 μl fresh medium and 10 μl CCK-8 for a 2 h incubation. Finally, the OD values were measured by Infinite M1000 pro (Tecan) at 450 nm. Cell migration ability was examined using an insert Transwell containing an 8 μm-pores membrane. Cells of different groups were trypsinized. 200 μl of the cell suspension in a serum-free medium containing 5 × 10^4^ cells were plated into each well of the inserts. 600 μl of the media containing 10% FBS were then added to the lower chamber. Cells were incubated at 37 °C for 48–72 h for *SF3A3*-OE SW480 cells, and 24 h for *SF3A3*-KD HCT116 cells. The cells that invaded the bottom of the membrane were fixed with 4% formaldehyde for 30 min at room temperature, and stained with crystal violet. The experiments were performed three times.

### Apoptosis assay

Adherent cells attached to the bottom of the culture plate were collected by trypsin digestion without EDTA. Then the cells were washed twice with PBS and centrifuged at 2000 rpm for 5 min to collect 1–5 × 10^5^ cells. 5 µl of 7-AAD dye solution was added to 50 µl of binding buffer and mixed. The collected cells were suspended in the above 7-AAD dye solution and incubated at room temperature in the dark for 5–15 min. After the reaction, 450 µl of binding buffer and 1 µl of Annexin V-PE were added and mixed at room temperature in the dark for 5–15 min. The stained cells were then analyzed by flow cytometry (excitation wavelength Ex = 488 nm; emission wave length Em = 578 nm; Annexin V-PE orange red fluorescence is recommended to use FL2 channel detection; excitation wavelength Ex = 546 nm; emission wave length Em = 647 nm; 7-AAD Red Fluorescence recommends using FL3 channel detection). The experiments were performed three times.

### High-throughput virtual docking and drug sensitivity test

The structural data and sequence information of the protein encoded by the *SF3A3* gene were obtained from the RCSB protein data bank (PDB) website (http://www.rcsb.org/). All heterogeneous atoms were removed for subsequent molecular docking. The protein docking mesh has been maximized for PEITC docking. Before virtual screening, *SF3A3* protein PDB files (5llm) were converted into macromolecules in PDBQT format. Then, AutoDock Vina 1.1.2 was used for subsequent molecular docking. Protein-ligand interactions were visualized using PyMOL version 1.7.4.5. The amino acid residues near the hit ligand (≤1) of spike protein were highlighted as potential interaction residues involved in protein-ligand interaction.

### In vivo tumor xenograft experiments

*SF3A3*-OE and control cells suspended in PBS (2 × 10^6^ cells/100 µl) were inoculated subcutaneously in the right armpit of ten nude mice under aseptic conditions. A power analysis showed that the sample size of five per group has an 80% power to detect a standardized effect size of about 2.0 SDs assuming a two-sided *t*-test with a 5% significance level. The diameter of xenotransplantation tumor in nude mice was measured with vernier caliper. After the tumor grew to 100 mm^3^, the animals were randomly divided into three groups. The tumor volume (TV) was measured three times a week, and the weight of mice was weighed at the same time. TV was used as the detection index, which was calculated as (length × width × height)/2. Then we euthanized the mice after 21 days. For euthanization, the mice were anesthetized with 4% sterile chloral hydrate (7–10 µl/g body weight, Sangon). Mice were euthanized by mouse spinal cord dislocation method, and their tumor tissues were collected for further research. All experiment protocols that involved animals were approved by the Research Animal Care Committee of Zhejiang University, China.

## Supplementary information

Supplementary Information

Supplementary Materials

## Data Availability

All data associated with this study are present in the paper or the supplementary materials. All biological resources from cell lines and animal experiments are available upon request.

## Code availability

Our methods, including the meta-analysis of GWASs using METAL and TWAS and colocalization analysis using FUSION, are freely accessible via the following links: METAL https://github.com/statgen/METAL and FUSION http://gusevlab.org/projects/fusion/.
