# Author Correction: Trans-ancestry transcriptome-wide association and functional studies to uncover novel susceptibility genes and therapeutic targets for colorectal cancer

**DOI:** 10.1038/s41698-025-00951-4

**Published:** 2025-05-27

**Authors:** Lijuan Wang, Lidan Hu, Jing Sun, Jianhui Zhao, Siyun Zhou, Lexin Liu, Wei Yu, Yeting Hu, Dan Zhou, Xiangrui Meng, Zhongshang Yuan, Honghe Zhang, Susan Farrington, Maria Timofeeva, Kefeng Ding, Julian Little, Malcolm Dunlop, Evropi Theodoratou, Xue Li

**Affiliations:** 1 School of Public Health, the Second affiliated Hospital, Zhejiang University School of Medicine, Hangzhou, China; 2 Centre for Global Health, Usher Institute, University of Edinburgh, Edinburgh, UK; 3 Department of Nephrology, The Children’s Hospital, Zhejiang University School of Medicine, National Clinical Research Center for Child Health, Hangzhou, China; 4 Department of Colorectal Surgery and Oncology, Key Laboratory of Cancer Prevention and Intervention, Ministry of Education, The Second Affiliated Hospital, Zhejiang University School of Medicine, Hangzhou, China; 5 Division of Psychiatry, University College of London, London, UK; 6 Department of Biostatistics, School of Public Health, Cheeloo College of Medicine, Shandong University, Jinan, China; 7 Department of Pathology and Women’s Hospital, Zhejiang University School of Medicine, Hangzhou, Zhejiang, China; 8 Colon Cancer Genetics Group, Medical Research Council Human Genetics Unit, Institute of Genetics and Cancer, University of Edinburgh, Edinburgh, UK; 9 Danish Institute for Advanced Study (DIAS), Epidemiology, Biostatistics and Biodemography Research Unit, Institute of Public Health, University of Southern Denmark, Odense, Denmark; 10 Center for Medical Research and Innovation in Digestive System Tumors, Ministry of Education, Hangzhou, China; 11 Zhejiang Provincial Clinical Research Center for CANCER, Hangzhou, China; 12 School of Epidemiology and Public Health, University of Ottawa, Ottawa, Ontario, Canada; 13 Cancer Research UK Edinburgh Centre, Medical Research Council Institute of Genetics and Molecular Medicine, University of Edinburgh, Edinburgh, UK

**Keywords:** Colorectal cancer, Gene therapy, Translational research, Oncogenesis, Functional genomics

Correction to: *npj Precision Oncology* 10.1038/s41698-025-00906-9, published online 29 April 2025

“In this article, Fig. 6 was published with missing data due to a formatting error. The original article has been corrected.”

