# Supplementary material for: Trans-ancestry transcriptome-wide association and functional studies to uncover novel susceptibility genes and therapeutic targets for colorectal cancer
Source: NPJ Precis Oncol. 2025 Apr 29;9:124. doi: 10.1038/s41698-025-00906-9 (PMC12041606; doi:10.1038/s41698-025-00906-9)
Supplement: Supplementary file 1 — Supplementary Information [file 41698_2025_906_MOESM1_ESM.docx]

**Supplementary Information**

**
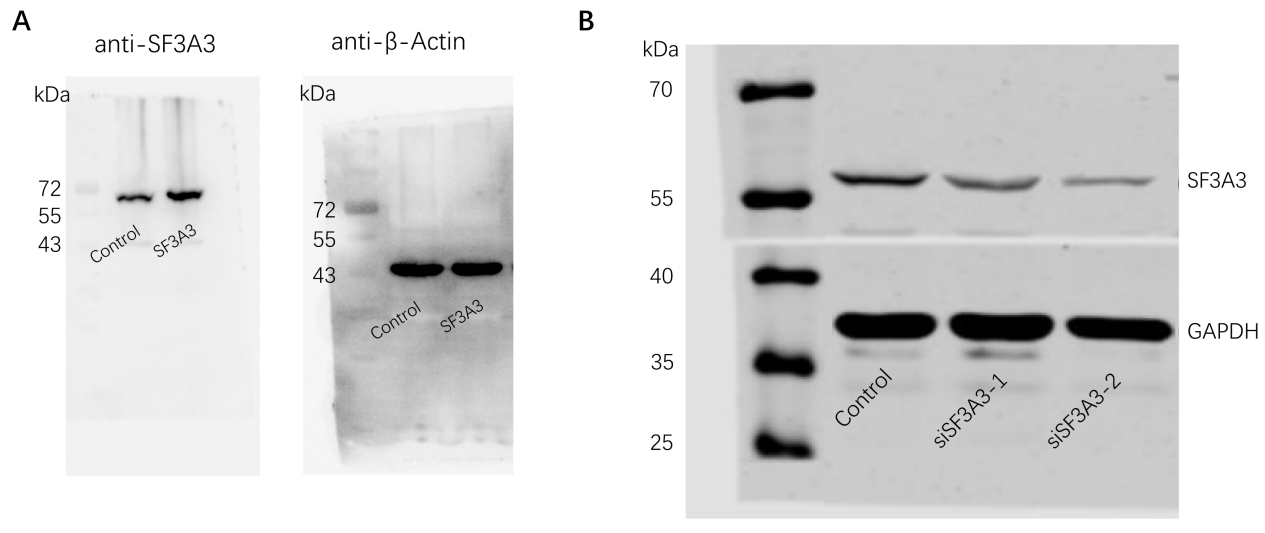
**

**Supplementary Figure 1. Raw data for Western blot of *SF3A3* protein levels.** (A) Western blot of *SF3A3* protein levels in SW480 cell lines. (B) Western blot of *SF3A3* protein levels in HCT116 cell lines.

**
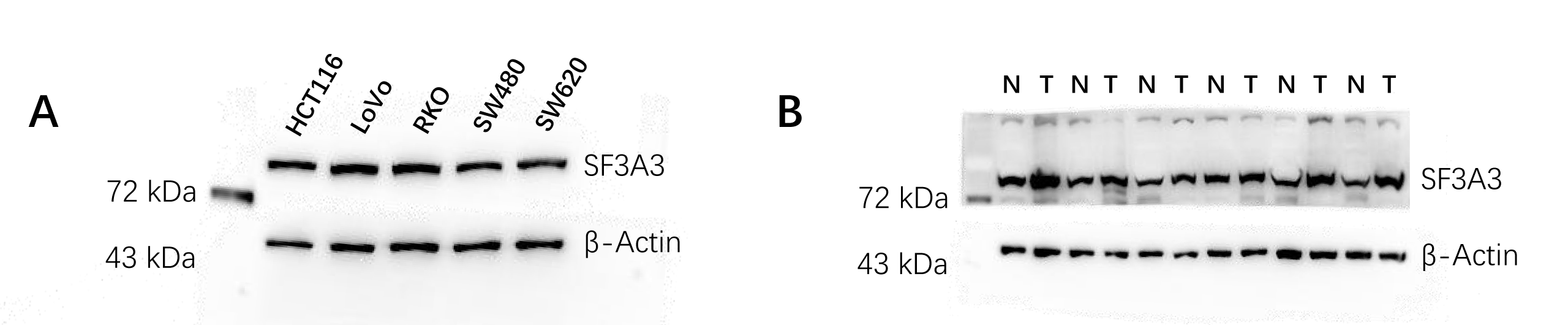
**

**Supplementary Figure 2. Raw data for Western blot of *SF3A3* protein levels.** (A) Western blot of *SF3A3* protein levels in all CRC cell lines. (B) Western blot of *SF3A3* protein levels in CRC normal and tumor tissues.
