# Supplementary material for: Trans-ancestry transcriptome-wide association and functional studies to uncover novel susceptibility genes and therapeutic targets for colorectal cancer
Source: NPJ Precis Oncol. 2025 Apr 29;9:124. doi: 10.1038/s41698-025-00906-9 (PMC12041606; doi:10.1038/s41698-025-00906-9)
Supplement: Supplementary file 2 — Supplementary Materials [file 41698_2025_906_MOESM2_ESM.pdf]

Supplementary Figures

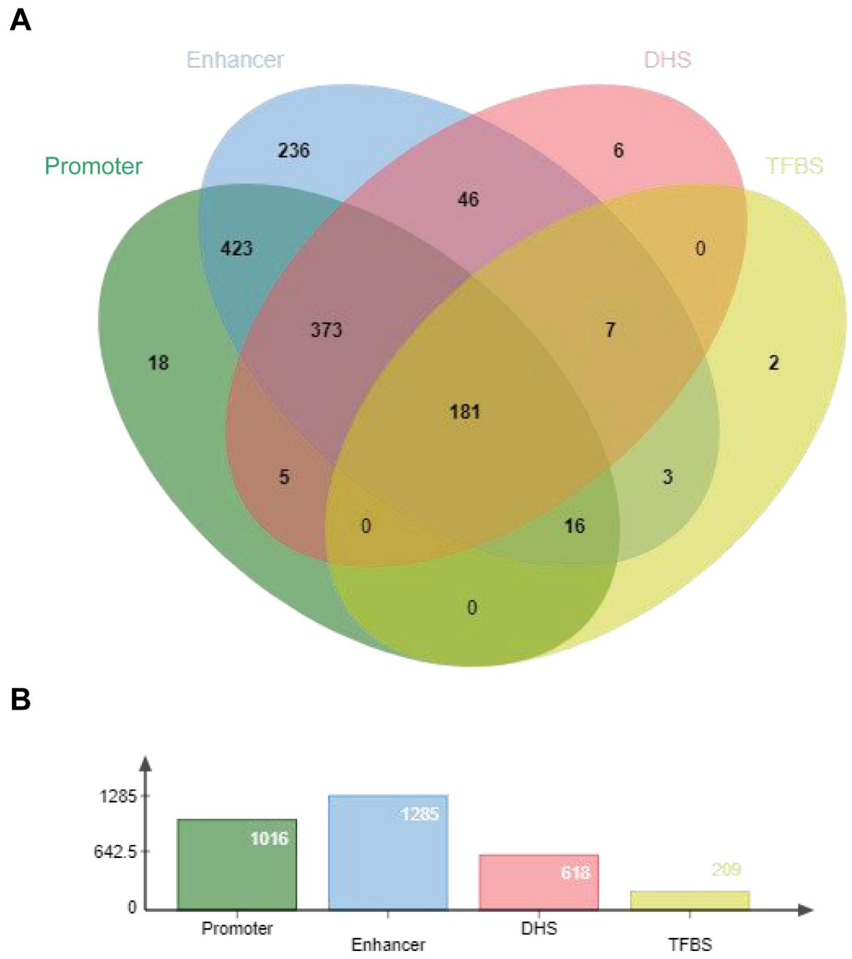

Supplementary Figure 1. Venn diagram of functional annotation of variants within TWAS-identified loci. (A) The Venn diagram shows four functional regions (promoter, enhancer, DHS, TFBS) the variants mainly enriched in. (B) Number of variants in each functional region

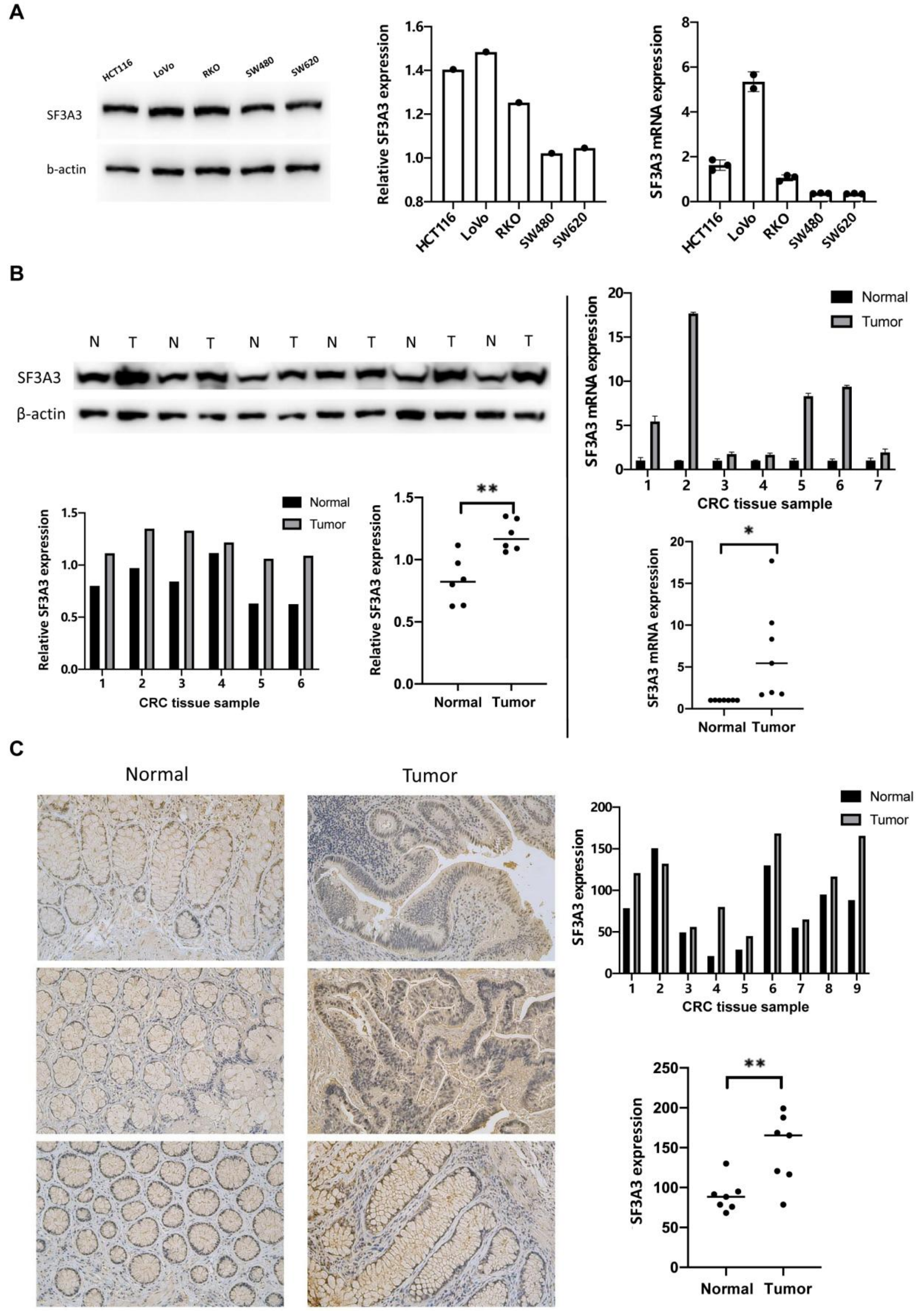

Supplementary Figure 2. Expression levels of *SF3A3* in CRC cell lines and tissue samples. (A) Western blot of *SF3A3* protein levels and relative mRNA expression levels of *SF3A3* in five CRC cell lines. (B) Western blot of *SF3A3* protein levels and relative mRNA expression levels of *SF3A3* in CRC normal and tumor tissues. (C) Expression levels of *SF3A3* in CRC normal and tumor tissues and the corresponding images. All *, $P < 0.05$; **, $P < 0.01$, calculated by the two-tailed Student t test.

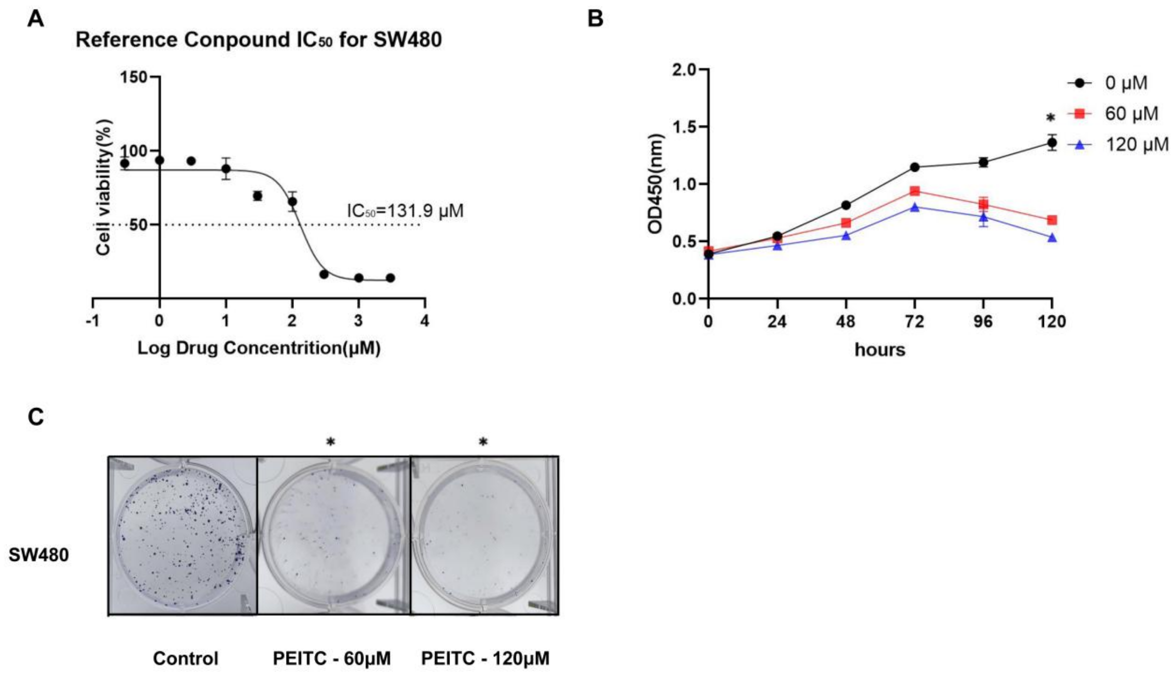

Supplementary Figure 3. PEITC inhibits tumor proliferation in CRC cells. (A) IC_{50} of phenethyl isothiocyanate (PEITC) in SW480 cells. (B) The prominent effect of PEITC treatment on proliferation of SW480 cells was detected using a CCK-8 assay. (C) The colony-forming ability of SW480 cells treated with PEITC was determined by a colony formation assay. All experiments were repeated three times and we used the mean value to present. All *, $P < 0.05$, calculated by the two-tailed Student t test.

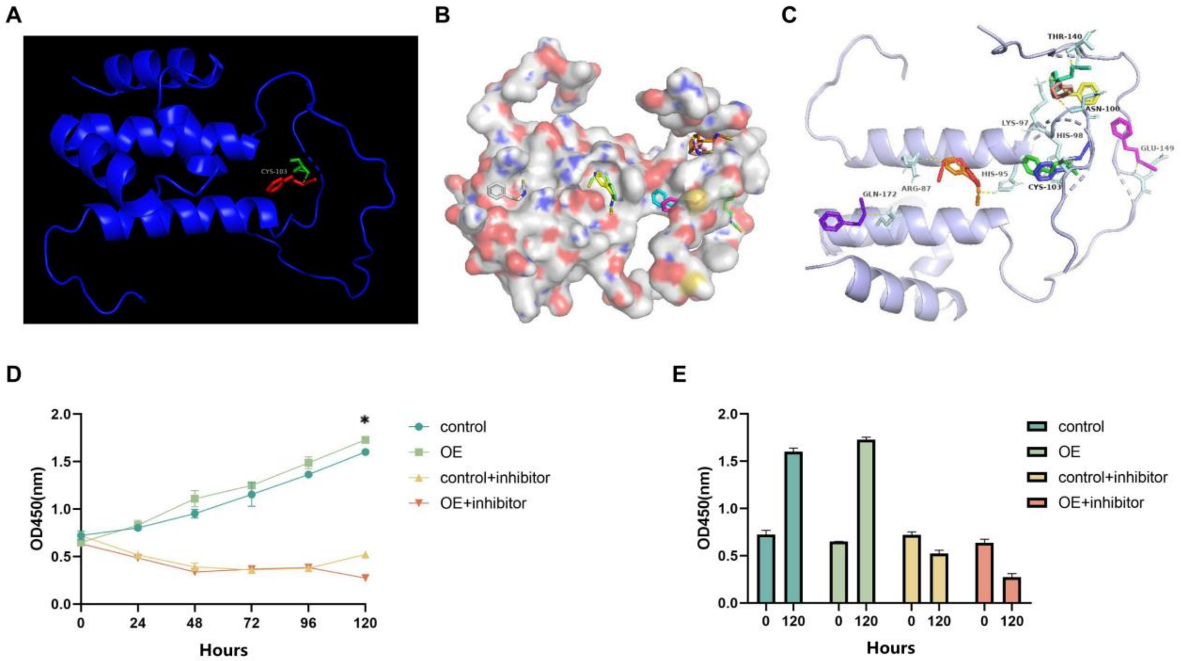

Supplementary Figure 4. PEITC inhibits tumor progression in CRC cells via *SF3A3*. (A-C) Molecular docking map between PEITC and *SF3A3*. (D-E) The prominent inhibitory effect of PEITC treatment on *SF3A3*-OE SW480 cells was detected using drug sensitivity test. All experiments were repeated three times and we used the mean value to present. All *, $P < 0.05$, calculated by the two-tailed Student t test.

Supplementary Tables

Supplementary Table 1. Summary of the included genome-wide association studies of CRC.

Series	Study Setting	Sampling	Genotyping platform
**European populations**			
CCFR1	Colon Cancer Family Registry	Recently diagnosed cases reported to population complete cancer registries in the USA (Seattle Familial Colorectal Cancer Registry). Canada (Ontario Familial Cancer Registry) and Australia (Australasian Colorectal Cancer Family Study). Population-based controls	Illumina Hap1M, Hap1M-Duo or Omni-express
CCFR2	Colon Cancer Family Registry	Cases from the Colon Cancer Family Registry, recruited from centres in Australia, Ontario, Seattle, USC, Mayo and Hawaii. Controls from the Cancer Genetic Markers of Susceptibility studies of breast and prostate cancer	Cases as for CCFR1, controls Illumina Hap300 and Hap240 or Hap550 arrays.
COIN	COIN trial	Multicentre study of cetuximab and other therapies in metastatic CRC. Cases recruited as a clinical-based series and controls as population-based series. Controls were unselected blood donors	Cases Affymetrix Axiom, controls Affymetrix 6.0
CORSA	CORSA (COloRectal cancer Study of Austria)	Population based screening study in colorectal cancer and adenoma	Affymetrix Axiom Genome-Wide CEU 1 Array
Croatia	Case-control study, Cases recruited from a teaching hospital in Zagreb	Recently diagnosed cases. Spouses controls from the same population	OmniExpressExome BeadChip 8v1.1 or 8v1.3

DACHS	DACHS Study, excluding previously reported cases and controls (GECCO study)	Population-based incidence cases aged>30 at diagnosis in the Rhine-Neckar-Odenwald region (southwest Germany). Community-based controls were randomly selected from population registries, matched by age (5-year groups), sex, and county	Illumina OncoArray
FIN	Finnish Colorectal Cancer Predisposition Study	Cases requited through Finnish Hospitals and Finnish Cancer Registry. Population-based controls from FINRISK, Health 2000, Finnish Twin Cohort and Helsinki Birth Cohort Studies	Cases Illumina HumanOmni 2.5M8v1, controls Illumina HumanHap 670k and 610k
NSCCG-OncoArray	National Study of Colorectal Cancer Genetics	Patients were selected for having a family history of CRC (at least one first degree relative) or age of diagnosis below 58. Controls obtained from cancer-free patients from the BCAC and PRACTICAL studies	Illumina OncoArray
SCOT	Short Course Oncology Treatment (SCOT) trial	Cases obtained from a study of adjuvant chemotherapy in colorectal cancer by the CACTUS and OCTO groups. Controls comprised of cancer-free individuals from The Heinz Nixdorf Recall study	Illumina Global Screening Array
Scotland1	COGS (Colorectal Cancer Susceptibility Study)	Population-based incidence cases aged <55 at diagnosis; Scotland. Population-based controls frequency matched by area of residence within Scotland	Illumina HumanHap 240K and 300
SOCCS/GS	Scottish Colorectal Cancer Study 3 (SOCCS3), Generation Scotland	Population based incident cases (Scottish colorectal cancer study 3), and population based controls from Scotland (Generation Scotland and Scottish colorectal cancer study 3 controls)	OmniExpressExome BeadChip 8v1.1 or 8v1.2 or 8v1.3, Omni5M

SOCCS/LBC	Scottish Colorectal Cancer Study 3 (SOCCS3), Lothian Birth Cohort	Population based incidence cases from Scotland. Population based controls from the Lothian Birth Cohorts 1921 and 1936	OmniExpressExome BeadChip 8v1.1 or 8v1.2 or 8v1.3, Illumina610-Quadv1
UK Biobank	Cases: prevalence (~51%) and incidence cases of colorectal cancer across UK: controls: population based controls without history of cancer	Population based incidence and prevalence cases of colorectal cancer,UK. Population based controls without history of cancer and/or colorectal adenoma matched 1:4 by age, gender, date of blood draw, ethnicity, and region of residence (two first letters of postal code)	Affymetrix UK Biobank Axiom Array
UK1	CORGI (Colorectal Tumour Gene Identification Consortium)	Cases enriched for family history of CRC, ascertained through UK clinical genetics clinics. Spouse controls with no personal history or family history of CRC	Illumina Hap300, Hap240S, Hap370, Hap550 or Omni2.5M arrays
VQ58	VICTOR, post treatment stager of a phase III, randomised trial of rofecoxib (VIOXX) in patients after potentially curative therapy. QUASAR2, multi-centre study of capectibine±bevacizumb as adjuvant treatment. 1958 Birth cohort controls	Cases recruited as a clinical-based series, controls as population-based series	Illumina Hap300, Hap240S, Hap370, Hap550 or Omni2.5M, controls Hap1.2M-Duo
**Asian populations**			
Aichi CRC study	Two collections: Aichi-1 in Stage 1 and Aichi-2 in Stage 2	CRC case status was confirmed via the Hospital-based Epidemiologic Research Program database, as well as the hospital-based cancer registry at the Aichi Cancer Center	Illumina OmniExpress, Illumina MEGA-Expanded

BBJ	BioBank Japan CRC Study	CRC cases and cancer-free controls were obtained from the BioBank Japan of the Personalized Medicine Project	Illumina HumanHap610-Quad BeadChip or OmniExpressExome
Guangzhou CRC Study	Two case-control studies conducted in Guangzhou, China (Guangzhou-1 and Guangzhou-2)	Cases were recruited from the Sun Yat-Sen University Cancer Center, Guangdong, China, from January 2002 to January 2012. Controls were cancer-free men and women recruited from the physical examination centers of several large hospitals in Guangdong during the same time period	Illumina OmniExpress, Illumina HumanExome-12v1 Beadchip
HCES-CRC	Hwasun Cancer Epidemiology Study-Colon and Rectum Cancer	Cases were CRC patients at Chonnam National University Hwasun Hospital, Jeollanam-do, South Korea, newly diagnosed from April 2004 to October 2014. Cancer-free controls were randomly selected from participants in the Korean Community Health Survey, an annual nationwide health interview survey, conducted from 2010 to 2012 in the Jindo and Bosung counties, Jeollanam-do, South Korea	Illumina OncoArray, Illumina MEGA-Expanded
KCPS-II	Korean Cancer Prevention Study-II CRC	Cancer status was confirmed via the National Cancer Registry and hospitalization records. Cancer-free controls were randomly selected from the same cohort study. Details of this study have been described elsewhere	Affymetrix 5.0
Korea-NCC	Korean-National Cancer Center CRC Study	Cases were histologically confirmed patients with CRC who received surgery between 2000 and 2004 at the Korean National Cancer Center (NCC). Controls were selected from participants of the Cancer Screening Cohort of the NCC, recruited between August 2002 and	Illumina OncoArray, Illumina MEGA-Expanded

		December 2004	
Korea-Seoul	Seoul CRC Study	Cases were CRC patients who were admitted to two university hospitals or one general cancer hospital in the Seoul Metropolitan Area between 1995 and 2004. Controls were patients of the same hospitals during the same time period from a wide spectrum of inpatients with non-neoplastic conditions	Illumina MEGA-Expanded
Shanghai CRC Study	Four case-control sets conducted in Shanghai, China (Shanghai-1, Shanghai-2, Shanghai-3 and Shanghai-4)	CRC cases for the Shanghai-1, Shanghai-2, and Shanghai-4 studies were identified from two cohort studies, the Shanghai Women's Health Study (SWHS) and the Shanghai Men's Health Study (SMHS). Cancer-free controls were identified from the SWHS and the SMHS [Shanghai-1, Shanghai-2 and Shanghai-4]	Affymetrix 6.0, Illumina OmniExpress, Illumina OncoArray, Illumina MEGA-Expanded

Supplementary Table 2. Significant candidate genes for CRC risk identified by TWAS analysis (at a FDR-corrected threshold of FDR < 0.05 in each tissue).

Gene	Chr	TSS (hg38)	NSNP	Model	Prediction Performance R2	Prediction Performance P	TWAS Z	TWAS P	FDR	Tissue
AAMP	2	218264128	436	susie	0.06	1.40E-05	5.09	3.51E-07	1.35E-04	GTEx Colon Sigmoid
AAMP	2	218264128	434	susie	0.03	1.10E-03	4.91	9.26E-07	3.49E-04	GTEx Colon Transverse
AAMP	2	218264128	431	top1	0.02	5.90E-06	4.62	3.91E-06	1.58E-03	YFS BLOOD RNAARR
AARD	8	116938206	462	susie	0.03	6.60E-04	5.78	7.39E-09	4.09E-06	GTEx Colon Sigmoid
ABCC2	10	99782639	438	enet	0.01	3.70E-03	4.16	3.19E-05	6.25E-03	GTEx Whole Blood
ABCC4	13	95019834	713	top1	0.05	7.70E-06	3.80	1.45E-04	1.70E-02	GTEx Colon Transverse
ABHD12B	14	50872052	486	top1	0.06	5.70E-11	-4.36	1.29E-05	3.44E-03	GTEx Whole Blood
ABHD12B	14	50872052	514	top1	0.04	1.20E-11	-4.36	1.29E-05	3.07E-03	NTR BLOOD RNAARR
ABHD12B	14	50872052	493	susie	0.01	2.30E-02	-4.29	1.82E-05	3.63E-03	GTEx Colon Sigmoid
ABHD12B	14	50872052	495	susie	0.17	4.10E-17	-4.28	1.91E-05	3.74E-03	GTEx Colon Transverse
ABHD12B	14	50872052	511	top1	0.02	7.70E-07	-4.09	4.31E-05	7.69E-03	YFS BLOOD RNAARR
ACTR1B	2	97655938	144	top1	0.16	1.10E-26	-4.40	1.09E-05	3.16E-03	GTEx Whole Blood
ACTR1B	2	97655938	145	susie	0.28	1.00E-27	-4.18	2.86E-05	5.03E-03	GTEx Colon Transverse
ACTR1B	2	97655938	145	susie	0.33	1.20E-29	-3.44	5.76E-04	4.78E-02	GTEx Colon Sigmoid
ADD3	10	109996367	335	enet	0.13	2.20E-11	-3.83	1.26E-04	1.47E-02	GTEx Colon Sigmoid
ADD3	10	109996367	326	top1	0.06	2.80E-10	-3.78	1.58E-04	2.02E-02	GTEx Whole Blood
ADD3	10	109996367	335	susie	0.08	1.00E-08	-3.49	4.90E-04	4.11E-02	GTEx Colon Transverse
AKAP13	15	85380570	577	susie	0.01	1.90E-02	-3.52	4.32E-04	3.91E-02	GTEx Colon Sigmoid
AKNA	9	114334155	673	bslmm	0.06	2.80E-19	3.64	2.71E-04	2.79E-02	YFS BLOOD RNAARR
ALG5	13	36949737	501	bslmm	0.02	1.40E-07	3.69	2.23E-04	2.49E-02	YFS BLOOD RNAARR
ARPC2	2	218217140	462	blup	0.01	4.80E-05	5.04	4.65E-07	2.49E-04	NTR BLOOD RNAARR

ARPC2	2	218217140	451	lasso	0.06	7.40E-07	4.79	1.67E-06	5.88E-04	GTEx Colon Transverse
ARPC2	2	218217140	453	top1	0.08	2.30E-07	4.77	1.87E-06	5.82E-04	GTEx Colon Sigmoid
ARPC2	2	218217140	449	susie	0.02	7.00E-04	4.38	1.21E-05	3.36E-03	GTEx Whole Blood
ASAH2B	10	50739317	464	susie	0.22	1.30E-21	-3.42	6.16E-04	4.71E-02	GTEx Colon Transverse
ASPDH	19	50511599	464	susie	0.13	6.90E-13	-5.17	2.32E-07	1.02E-04	GTEx Colon Transverse
ASPDH	19	50511599	463	lasso	0.09	4.50E-08	-4.46	8.16E-06	2.03E-03	GTEx Colon Sigmoid
ATF1	12	50763709	297	top1	0.08	5.10E-08	7.04	1.89E-12	3.33E-09	GTEx Colon Transverse
ATF1	12	50763709	307	top1	0.22	4.20E-19	7.04	1.91E-12	2.38E-09	GTEx Colon Sigmoid
AXIN1	16	287439	336	top1	0.34	3.60E-116	4.52	6.07E-06	2.26E-03	YFS BLOOD RNAARR
B3GNT8	19	41425358	364	susie	0.10	6.30E-10	-3.97	7.16E-05	9.45E-03	GTEx Colon Transverse
B9D2	19	41354416	368	enet	0.02	5.60E-07	-6.03	1.61E-09	1.79E-06	YFS BLOOD RNAARR
BAMBI	10	28677509	607	enet	0.24	7.80E-43	4.27	1.97E-05	4.69E-03	GTEx Whole Blood
BAMBI	10	28677509	610	bslmm	0.31	3.90E-104	3.44	5.72E-04	4.11E-02	YFS BLOOD RNAARR
BCKDHA	19	41397807	375	enet	0.18	7.30E-31	3.65	2.65E-04	2.94E-02	GTEx Whole Blood
BCKDHA	19	41397807	384	enet	0.06	1.90E-18	3.50	4.59E-04	3.65E-02	YFS BLOOD RNAARR
BORA	13	72727748	499	enet	0.03	4.10E-04	-4.13	3.70E-05	6.11E-03	GTEx Colon Transverse
BRD3	9	134030304	588	top1	0.10	1.90E-10	-4.04	5.32E-05	7.80E-03	GTEx Colon Transverse
C10orf105	10	71711700	629	susie	0.01	8.00E-02	-4.41	1.01E-05	2.40E-03	GTEx Colon Sigmoid
C11orf53	11	111245724	460	lasso	0.24	7.80E-24	-8.54	1.30E-17	6.87E-14	GTEx Colon Transverse
C1orf122	1	37806978	454	susie	0.01	6.10E-03	-5.38	7.44E-08	5.50E-05	GTEx Whole Blood
C4orf33	4	129093316	373	enet	0.20	6.10E-35	-3.58	3.41E-04	3.55E-02	GTEx Whole Blood
C9orf64	9	83938310	535	susie	0.02	1.60E-04	-4.10	4.18E-05	7.95E-03	GTEx Whole Blood
CA11	19	48637945	435	bslmm	0.03	3.60E-11	-4.18	2.91E-05	5.90E-03	YFS BLOOD RNAARR
CABLES2	20	62388633	520	susie	0.36	1.80E-32	-8.33	7.99E-17	3.98E-13	GTEx Colon Sigmoid
CABLES2	20	62388633	517	top1	0.23	2.80E-22	-6.71	1.98E-11	1.74E-08	GTEx Colon Transverse

CABLES2	20	62388633	512	enet	0.15	2.70E-26	-6.68	2.45E-11	8.16E-08	GTEx Whole Blood
CABLES2	20	62388633	440	top1	0.03	1.40E-09	-5.97	2.37E-09	2.11E-06	YFS BLOOD RNAARR
CACNA1I	22	39570752	390	enet	0.01	9.30E-03	-4.20	2.64E-05	5.49E-03	GTEx Whole Blood
CALR	19	12938577	305	top1	0.03	1.10E-09	-3.44	5.88E-04	4.67E-02	NTR BLOOD RNAARR
CAPN15	16	527711	443	enet	0.06	1.70E-11	-3.84	1.22E-04	1.69E-02	GTEx Whole Blood
CAVIN1	17	42402448	259	top1	0.08	1.00E-07	3.52	4.27E-04	3.91E-02	GTEx Colon Sigmoid
CCDC146	7	77122433	285	susie	0.17	5.00E-29	-3.89	1.02E-04	1.54E-02	GTEx Whole Blood
CCM2	7	44999474	413	enet	0.26	5.70E-45	-3.85	1.20E-04	1.69E-02	GTEx Whole Blood
CDH1	16	68737291	315	susie	0.03	2.10E-03	4.70	2.66E-06	7.80E-04	GTEx Colon Sigmoid
CEBPB	20	50190829	419	enet	0.04	2.50E-12	-3.91	9.29E-05	1.38E-02	YFS BLOOD RNAARR
CEP68	2	65056353	429	top1	0.22	1.40E-21	-3.56	3.78E-04	3.56E-02	GTEx Colon Transverse
CERS5	12	50129288	294	susie	0.03	6.70E-04	-6.98	2.96E-12	3.91E-09	GTEx Colon Transverse
CERS5	12	50129288	305	top1	0.08	3.00E-07	-5.42	6.06E-08	2.75E-05	GTEx Colon Sigmoid
CIB1	15	90229974	399	lasso	0.09	2.70E-08	3.45	5.63E-04	4.75E-02	GTEx Colon Sigmoid
CKAP2	13	52455428	359	lasso	0.31	3.00E-27	3.43	5.96E-04	4.84E-02	GTEx Colon Sigmoid
CKAP5	11	46743047	291	bslmm	0.01	3.60E-03	3.59	3.29E-04	3.01E-02	YFS BLOOD RNAARR
CLSTN2	3	139935184	572	susie	0.00	1.60E-01	-3.48	5.08E-04	4.19E-02	GTEx Colon Transverse
CNTN2	1	205042936	603	enet	0.13	1.50E-11	3.83	1.27E-04	1.47E-02	GTEx Colon Sigmoid
CNTN2	1	205042936	590	enet	0.08	1.70E-08	3.50	4.58E-04	3.96E-02	GTEx Colon Transverse
COLCA2	11	111298545	449	enet	0.36	2.80E-37	-8.11	5.18E-16	1.37E-12	GTEx Colon Transverse
COLCA2	11	111298545	449	lasso	0.01	5.20E-02	-5.95	2.62E-09	1.63E-06	GTEx Colon Sigmoid
CORO1C	12	108645108	489	lasso	0.12	9.80E-36	3.62	2.98E-04	2.95E-02	YFS BLOOD RNAARR
CORO1C	12	108645108	502	bslmm	0.05	8.70E-16	3.43	6.11E-04	4.68E-02	NTR BLOOD RNAARR
COX14	12	50112081	294	susie	0.07	4.40E-07	6.31	2.76E-10	2.08E-07	GTEx Colon Transverse
COX14	12	50112081	305	enet	0.17	1.20E-14	6.31	2.87E-10	2.04E-07	GTEx Colon Sigmoid

COX14	12	50112081	291	susie	0.11	1.60E-19	5.16	2.50E-07	1.36E-04	GTEx Whole Blood
COX15	10	99710867	462	lasso	0.03	1.70E-10	6.40	1.59E-10	1.70E-07	NTR BLOOD RNAARR
CRAT	9	129094141	371	enet	0.33	7.10E-60	-3.92	8.99E-05	1.46E-02	GTEx Whole Blood
CRAT	9	129094141	378	susie	0.14	2.10E-12	-3.86	1.12E-04	1.40E-02	GTEx Colon Sigmoid
CRAT	9	129094141	375	enet	0.22	2.60E-71	-3.59	3.34E-04	3.01E-02	YFS BLOOD RNAARR
CRTC3	15	90529922	503	blup	0.01	4.20E-05	-4.10	4.10E-05	7.62E-03	YFS BLOOD RNAARR
CTU1	19	51097605	614	susie	0.02	1.30E-02	-3.93	8.47E-05	1.14E-02	GTEx Colon Sigmoid
CXCR1	2	218162840	454	lasso	0.03	1.10E-08	4.40	1.10E-05	2.95E-03	NTR BLOOD RNAARR
CXCR1	2	218162840	448	enet	0.14	1.30E-44	3.82	1.32E-04	1.84E-02	YFS BLOOD RNAARR
CXCR1	2	218162840	450	susie	0.04	2.90E-07	3.67	2.40E-04	2.80E-02	GTEx Whole Blood
DACT1	14	58633966	483	enet	0.08	1.60E-08	-4.58	4.68E-06	1.30E-03	GTEx Colon Transverse
DCBLD1	6	117453816	419	top1	0.07	5.00E-07	3.57	3.64E-04	3.42E-02	GTEx Colon Sigmoid
DCBLD1	6	117453816	419	top1	0.05	7.40E-06	3.57	3.64E-04	3.52E-02	GTEx Colon Transverse
DENND4C	9	19230434	677	top1	0.04	3.00E-14	4.41	1.05E-05	3.60E-03	YFS BLOOD RNAARR
DIP2B	12	50504984	385	bslmm	0.27	2.80E-90	-4.99	6.06E-07	3.38E-04	YFS BLOOD RNAARR
DIP2B	12	50504984	280	lasso	0.19	2.10E-33	4.74	2.13E-06	7.88E-04	GTEx Whole Blood
DIS3	13	72752168	489	enet	0.01	1.40E-03	-3.40	6.74E-04	4.48E-02	YFS BLOOD RNAARR
DMAC2	19	41431317	360	susie	0.06	2.40E-11	-3.93	8.48E-05	1.41E-02	GTEx Whole Blood
DNA2	10	68414063	384	lasso	0.26	2.40E-26	4.11	4.03E-05	6.28E-03	GTEx Colon Transverse
DNA2	10	68414063	384	enet	0.37	1.70E-33	3.85	1.21E-04	1.47E-02	GTEx Colon Sigmoid
DNA2	10	68414063	382	lasso	0.09	2.80E-16	3.80	1.42E-04	1.85E-02	GTEx Whole Blood
DNAJC16	1	15526812	506	susie	0.03	9.20E-04	3.44	5.91E-04	4.59E-02	GTEx Colon Transverse
DOCK10	2	224765089	555	blup	0.02	6.40E-08	-3.82	1.32E-04	1.63E-02	NTR BLOOD RNAARR
EMC10	19	50476399	440	enet	0.07	1.50E-12	3.70	2.16E-04	2.57E-02	GTEx Whole Blood
EXOSC5	19	41386370	377	lasso	0.03	1.80E-03	-4.12	3.83E-05	6.43E-03	GTEx Colon Sigmoid

F2	11	46719195	238	susie	0.01	1.40E-02	3.45	5.57E-04	4.46E-02	GTEx Colon Transverse
FADS1	11	61799626	418	top1	0.10	2.60E-09	7.83	4.96E-15	1.24E-11	GTEx Colon Sigmoid
FADS1	11	61799626	418	lasso	0.09	6.10E-29	-4.90	9.70E-07	4.80E-04	YFS BLOOD RNAARR
FADS1	11	61799626	398	enet	0.04	3.00E-07	-4.24	2.20E-05	4.88E-03	GTEx Whole Blood
FAM89B	11	65572348	352	top1	0.03	3.50E-06	5.35	8.94E-08	5.95E-05	GTEx Whole Blood
FAM89B	11	65572348	349	bslmm	0.10	1.40E-30	4.37	1.27E-05	3.62E-03	YFS BLOOD RNAARR
FHL3	1	37996769	451	enet	0.58	8.20E-237	5.74	9.57E-09	7.11E-06	YFS BLOOD RNAARR
FHL3	1	37996769	465	bslmm	0.10	7.50E-31	4.88	1.08E-06	4.63E-04	NTR BLOOD RNAARR
FHL3	1	37996769	465	lasso	0.50	9.60E-103	4.84	1.28E-06	5.68E-04	GTEx Whole Blood
FHL3	1	37996769	467	top1	0.02	1.90E-03	4.76	1.92E-06	6.34E-04	GTEx Colon Transverse
FIG4	6	109690608	478	blup	0.04	2.70E-13	3.73	1.94E-04	2.28E-02	YFS BLOOD RNAARR
FNIP2	4	158769025	265	susie	0.02	2.20E-03	-4.09	4.36E-05	6.58E-03	GTEx Colon Transverse
FNIP2	4	158769025	302	bslmm	0.05	6.90E-17	-4.02	5.89E-05	1.05E-02	NTR BLOOD RNAARR
FUT2	19	48695970	449	top1	0.23	1.10E-22	-4.33	1.48E-05	3.17E-03	GTEx Colon Transverse
GABBR1	6	29555628	39	enet	0.03	1.40E-09	-4.05	5.14E-05	8.81E-03	YFS BLOOD RNAARR
GCHFR	15	40764067	300	enet	0.05	4.00E-17	3.64	2.75E-04	2.79E-02	YFS BLOOD RNAARR
GDPGP1	15	90233807	399	enet	0.22	5.80E-19	3.82	1.33E-04	1.51E-02	GTEx Colon Sigmoid
GJC3	7	99923265	259	enet	0.20	2.10E-17	-3.77	1.62E-04	1.79E-02	GTEx Colon Sigmoid
GJC3	7	99923265	243	susie	0.02	5.20E-03	-3.70	2.15E-04	2.32E-02	GTEx Colon Transverse
GNA12	7	2728104	561	enet	0.10	1.20E-10	-4.26	2.05E-05	3.87E-03	GTEx Colon Transverse
GNA12	7	2728104	555	susie	0.10	8.60E-17	-4.01	5.98E-05	1.08E-02	GTEx Whole Blood
GPATCH1	19	33080898	448	enet	0.10	1.20E-16	-5.15	2.66E-07	1.36E-04	GTEx Whole Blood
GPATCH1	19	33080898	456	susie	0.07	2.70E-07	-4.58	4.55E-06	1.30E-03	GTEx Colon Transverse
GPATCH1	19	33080898	456	top1	0.07	2.10E-06	-3.66	2.57E-04	2.62E-02	GTEx Colon Sigmoid
GPATCH1	19	33080898	453	blup	0.03	2.80E-09	-3.39	7.03E-04	4.57E-02	YFS BLOOD RNAARR

GPBAR1	2	218259495	435	top1	0.06	4.70E-20	-5.01	5.38E-07	3.38E-04	YFS BLOOD RNAARR
GPN3	12	110452485	249	susie	0.02	9.00E-03	3.44	5.81E-04	4.58E-02	GTEx Colon Transverse
GTF2A2	15	59638061	510	enet	0.09	1.10E-28	3.56	3.72E-04	3.13E-02	YFS BLOOD RNAARR
HLA-DPB1	6	33075989	301	enet	0.38	6.90E-135	-3.58	3.38E-04	3.01E-02	YFS BLOOD RNAARR
HLA-DQA1	6	32628178	200	enet	0.66	3.30E-301	3.75	1.78E-04	2.14E-02	YFS BLOOD RNAARR
HLA-DRA	6	32439877	209	lasso	0.26	1.10E-84	-3.78	1.57E-04	2.00E-02	YFS BLOOD RNAARR
HLA-DRB5	6	32517352	252	enet	0.66	2.80E-295	-3.85	1.18E-04	1.58E-02	NTR BLOOD RNAARR
HLA-F	6	29722774	40	lasso	0.07	1.00E-06	4.79	1.68E-06	5.58E-04	GTEx Colon Sigmoid
HLA-F	6	29722774	41	susie	0.23	2.80E-23	4.47	7.98E-06	1.92E-03	GTEx Colon Transverse
HLA-F	6	29722774	40	susie	0.35	2.00E-65	3.98	6.94E-05	1.22E-02	GTEx Whole Blood
HLA-F	6	29722774	42	enet	0.26	2.60E-85	3.77	1.65E-04	1.81E-02	NTR BLOOD RNAARR
HNRNPA1L2	13	52642430	406	susie	0.02	5.20E-04	3.54	4.01E-04	4.11E-02	GTEx Whole Blood
ICAM3	19	10333775	375	top1	0.06	1.20E-19	-3.47	5.13E-04	3.94E-02	YFS BLOOD RNAARR
INPP5B	1	37860696	486	enet	0.31	6.10E-105	4.24	2.24E-05	5.26E-03	YFS BLOOD RNAARR
INPP5B	1	37860696	463	susie	0.33	2.30E-29	4.13	3.67E-05	6.43E-03	GTEx Colon Sigmoid
INPP5B	1	37860696	498	lasso	0.04	3.60E-12	3.76	1.69E-04	1.81E-02	NTR BLOOD RNAARR
INPP5B	1	37860696	461	enet	0.43	1.40E-84	3.65	2.59E-04	2.92E-02	GTEx Whole Blood
IQCH	15	67254785	483	lasso	0.05	1.70E-05	4.33	1.46E-05	3.07E-03	GTEx Colon Sigmoid
IQGAP1	15	90388241	387	susie	0.18	1.70E-31	3.85	1.20E-04	1.69E-02	GTEx Whole Blood
IQGAP1	15	90388241	459	enet	0.04	1.50E-12	3.72	2.00E-04	1.95E-02	NTR BLOOD RNAARR
ITIH4	3	52812961	385	enet	0.50	2.90E-192	4.16	3.18E-05	6.16E-03	YFS BLOOD RNAARR
ITIH4	3	52812961	388	susie	0.38	2.00E-34	3.96	7.63E-05	1.09E-02	GTEx Colon Sigmoid
KCNK7	11	65592835	345	top1	0.02	6.70E-04	-3.64	2.69E-04	2.94E-02	GTEx Whole Blood
KIF1C	17	4997949	438	top1	0.01	2.80E-03	-4.19	2.81E-05	5.90E-03	YFS BLOOD RNAARR
KL	13	33016422	522	blup	0.04	7.60E-13	3.76	1.73E-04	2.14E-02	YFS BLOOD RNAARR

LAMC1	1	183023419	469	lasso	0.17	2.70E-52	6.94	3.82E-12	9.03E-09	YFS BLOOD RNAARR
LAMC1	1	183023419	433	enet	0.22	9.20E-39	6.79	1.12E-11	7.46E-08	GTEx Whole Blood
LAMC1	1	183023419	479	lasso	0.07	3.70E-21	6.71	1.91E-11	4.09E-08	NTR BLOOD RNAARR
LAMC1	1	183023419	440	susie	0.04	3.00E-04	-6.57	5.01E-11	4.16E-08	GTEx Colon Sigmoid
LAMC1	1	183023419	440	susie	0.05	1.40E-05	-5.35	8.69E-08	4.17E-05	GTEx Colon Transverse
LIMA1	12	50175787	315	top1	0.06	1.30E-19	6.94	4.05E-12	9.03E-09	YFS BLOOD RNAARR
LIMA1	12	50175787	308	top1	0.05	2.00E-05	-6.62	3.61E-11	3.60E-08	GTEx Colon Sigmoid
LIMA1	12	50175787	293	enet	0.03	9.50E-06	6.54	6.28E-11	1.39E-07	GTEx Whole Blood
LIMA1	12	50175787	297	top1	0.05	1.00E-05	-5.86	4.51E-09	2.98E-06	GTEx Colon Transverse
LIMK2	22	31212238	366	enet	0.06	3.60E-18	3.43	6.01E-04	4.25E-02	YFS BLOOD RNAARR
LMOD1	1	201896455	478	susie	0.07	2.50E-07	-5.02	5.25E-07	2.13E-04	GTEx Colon Transverse
LRP1	12	57128482	336	enet	0.08	4.00E-08	3.83	1.30E-04	1.56E-02	GTEx Colon Transverse
LRRIQ4	3	169812869	409	susie	0.01	1.50E-02	-4.71	2.49E-06	7.74E-04	GTEx Colon Transverse
LZTS1	8	20246164	632	enet	0.03	1.20E-10	3.41	6.40E-04	4.39E-02	YFS BLOOD RNAARR
MAB21L2	4	150582150	330	top1	0.02	2.70E-03	-4.24	2.27E-05	4.13E-03	GTEx Colon Transverse
MAMSTR	19	48712724	441	top1	0.05	2.80E-05	4.33	1.48E-05	3.07E-03	GTEx Colon Sigmoid
MAN2A2	15	90902217	483	susie	0.11	9.00E-19	-4.52	6.30E-06	2.10E-03	GTEx Whole Blood
MAN2A2	15	90902217	486	lasso	0.13	1.90E-39	-4.29	1.78E-05	4.67E-03	YFS BLOOD RNAARR
MCFD2	2	46901869	564	bslmm	0.11	8.90E-34	-3.42	6.29E-04	4.38E-02	YFS BLOOD RNAARR
METRNL	17	83079608	242	susie	0.04	9.60E-08	6.40	1.55E-10	2.06E-07	GTEx Whole Blood
METTL7A	12	50923471	363	lasso	0.02	4.70E-06	-4.52	6.33E-06	1.94E-03	NTR BLOOD RNAARR
METTL7A	12	50923471	360	lasso	0.08	1.80E-25	-3.71	2.04E-04	2.33E-02	YFS BLOOD RNAARR
MEX3C	18	51174549	417	top1	0.01	2.20E-05	3.41	6.54E-04	4.42E-02	YFS BLOOD RNAARR
MGAT3	22	39457011	388	susie	0.03	1.20E-05	3.63	2.88E-04	3.04E-02	GTEx Whole Blood
MRPS31	13	40729127	387	blup	0.03	6.50E-11	3.39	7.07E-04	4.57E-02	YFS BLOOD RNAARR

MTHFSD	16	86530177	922	susie	0.22	8.90E-22	-3.52	4.28E-04	3.90E-02	GTEx Colon Transverse
MYO5C	15	52192321	386	susie	0.10	4.70E-10	3.57	3.57E-04	3.52E-02	GTEx Colon Transverse
MYO5C	15	52192321	415	enet	0.02	5.50E-07	3.47	5.11E-04	3.94E-02	YFS BLOOD RNAARR
MYO9A	15	71822290	520	lasso	0.02	5.30E-08	-4.23	2.36E-05	5.26E-03	YFS BLOOD RNAARR
MYO9A	15	71822290	323	susie	0.01	4.50E-03	-4.19	2.76E-05	5.57E-03	GTEx Whole Blood
MYO9A	15	71822290	341	top1	0.01	1.90E-02	-4.12	3.87E-05	6.43E-03	GTEx Colon Sigmoid
MYRF	11	61752635	367	top1	0.15	2.20E-26	-4.93	8.18E-07	3.89E-04	GTEx Whole Blood
MYRF	11	61752635	372	top1	0.06	1.30E-06	-4.11	4.04E-05	6.28E-03	GTEx Colon Transverse
N6AMT1	21	28872190	466	enet	0.02	1.20E-04	3.66	2.52E-04	2.89E-02	GTEx Whole Blood
NAA38	17	7856684	508	lasso	0.14	2.20E-12	4.18	2.95E-05	5.44E-03	GTEx Colon Sigmoid
NBPF9	1	149052185	3	susie	0.31	1.20E-27	-3.43	6.02E-04	4.84E-02	GTEx Colon Sigmoid
NCF2	1	183554460	538	susie	0.01	5.00E-02	3.69	2.26E-04	2.39E-02	GTEx Colon Transverse
NDFIP1	5	142108778	580	top1	0.05	1.40E-09	4.23	2.33E-05	5.00E-03	GTEx Whole Blood
NID2	14	52004808	631	top1	0.09	3.10E-09	3.98	6.92E-05	9.45E-03	GTEx Colon Transverse
NIN	14	50719762	488	susie	0.06	6.10E-07	-4.40	1.08E-05	2.48E-03	GTEx Colon Transverse
NPLOC4	17	81556886	296	enet	0.08	3.10E-26	3.66	2.54E-04	2.70E-02	YFS BLOOD RNAARR
NTN5	19	48661406	457	enet	0.06	5.50E-11	3.88	1.05E-04	1.55E-02	GTEx Whole Blood
NXN	17	799309	425	enet	0.07	2.50E-12	-3.83	1.30E-04	1.77E-02	GTEx Whole Blood
OSBPL2	20	62231921	475	enet	0.60	7.60E-252	-3.83	1.29E-04	1.84E-02	YFS BLOOD RNAARR
OTUD7A	15	31475397	320	top1	0.02	1.20E-02	3.50	4.65E-04	3.96E-02	GTEx Colon Transverse
PAFAH1B2	11	117144283	564	top1	0.02	3.90E-06	-3.96	7.43E-05	1.14E-02	YFS BLOOD RNAARR
PARP6	15	72241180	235	susie	0.01	2.00E-02	4.27	1.97E-05	4.69E-03	GTEx Whole Blood
PARP6	15	72241180	254	top1	0.04	9.00E-05	4.08	4.46E-05	7.17E-03	GTEx Colon Sigmoid
PCNT	21	46324123	452	top1	0.07	1.00E-06	-3.91	9.19E-05	1.20E-02	GTEx Colon Sigmoid
PCSK7	11	117204336	541	lasso	0.15	7.60E-48	3.55	3.80E-04	3.14E-02	YFS BLOOD RNAARR

PDCD5	19	32581189	408	enet	0.10	5.00E-09	3.65	2.58E-04	2.62E-02	GTEx Colon Sigmoid
PDGFB	22	39223358	405	enet	0.07	4.60E-12	-4.31	1.62E-05	4.15E-03	GTEx Whole Blood
PIP4K2A	10	22534853	321	susie	0.07	3.40E-07	-3.97	7.16E-05	9.45E-03	GTEx Colon Transverse
PKP4	2	158456951	485	susie	0.37	3.10E-70	-3.76	1.69E-04	2.12E-02	GTEx Whole Blood
PKP4	2	158456951	564	top1	0.03	2.50E-11	-3.48	5.09E-04	4.20E-02	NTR BLOOD RNAARR
PNKD	2	218269650	432	enet	0.11	3.10E-18	4.76	1.92E-06	7.52E-04	GTEx Whole Blood
PNKD	2	218269650	446	lasso	0.09	2.90E-27	4.27	1.94E-05	4.80E-03	YFS BLOOD RNAARR
POLR1D	13	27620741	530	lasso	0.10	4.30E-31	3.81	1.37E-04	1.63E-02	NTR BLOOD RNAARR
PREX1	20	48624251	457	enet	0.05	4.80E-09	6.08	1.22E-09	1.35E-06	GTEx Whole Blood
PREX1	20	48624251	462	lasso	0.00	2.30E-01	4.82	1.41E-06	5.02E-04	GTEx Colon Sigmoid
PRKAA1	5	40759388	469	lasso	0.01	1.50E-04	-4.38	1.20E-05	3.62E-03	YFS BLOOD RNAARR
PTMS	12	6765515	230	enet	0.02	1.60E-06	3.59	3.36E-04	3.01E-02	YFS BLOOD RNAARR
PTPA	9	129110949	371	enet	0.30	4.40E-53	-3.91	9.36E-05	1.48E-02	GTEx Whole Blood
PTPA	9	129110949	373	susie	0.18	1.20E-17	-3.86	1.12E-04	1.41E-02	GTEx Colon Transverse
PYGL	14	50857890	518	enet	0.18	8.00E-18	-3.71	2.07E-04	2.28E-02	GTEx Colon Transverse
RAD52	12	911735	406	enet	0.21	3.20E-18	3.99	6.72E-05	9.85E-03	GTEx Colon Sigmoid
RASIP1	19	48720584	428	susie	0.05	1.50E-05	4.16	3.13E-05	5.33E-03	GTEx Colon Transverse
RASIP1	19	48720584	435	enet	0.13	9.10E-12	3.99	6.67E-05	9.85E-03	GTEx Colon Sigmoid
RBM22	5	150690791	524	top1	0.05	5.70E-06	3.64	2.73E-04	2.83E-02	GTEx Colon Transverse
RCCD1	15	90954880	483	lasso	0.34	6.00E-35	-3.73	1.94E-04	2.18E-02	GTEx Colon Transverse
RCCD1	15	90954880	475	top1	0.09	1.10E-15	-3.72	1.96E-04	2.42E-02	GTEx Whole Blood
RCCD1	15	90954880	469	top1	0.02	8.80E-07	-3.72	1.96E-04	1.95E-02	NTR BLOOD RNAARR
RCCD1	15	90954880	473	bslmm	0.29	8.30E-96	-3.58	3.47E-04	3.03E-02	YFS BLOOD RNAARR
RCCD1	15	90954880	487	susie	0.31	4.00E-27	-3.57	3.52E-04	3.37E-02	GTEx Colon Sigmoid
RHPN2	19	32978591	443	top1	0.04	3.00E-05	4.32	1.56E-05	3.17E-03	GTEx Colon Transverse

RHPN2	19	32978591	443	top1	0.11	1.20E-09	3.66	2.57E-04	2.62E-02	GTEx Colon Sigmoid
RNF44	5	176526711	358	enet	0.01	1.50E-05	-3.50	4.63E-04	3.97E-02	NTR BLOOD RNAARR
RPS5	19	58386399	314	susie	0.08	3.20E-07	3.94	8.31E-05	1.14E-02	GTEx Colon Sigmoid
RUFY2	10	68341106	393	susie	0.12	1.60E-10	-4.20	2.64E-05	5.06E-03	GTEx Colon Sigmoid
RUFY2	10	68341106	393	susie	0.12	2.90E-12	-3.85	1.18E-04	1.45E-02	GTEx Colon Transverse
RWDD2B	21	29004383	414	blup	0.01	3.60E-03	3.46	5.38E-04	4.00E-02	YFS BLOOD RNAARR
S100B	21	46598603	306	lasso	0.05	6.40E-05	-4.36	1.30E-05	2.94E-03	GTEx Colon Sigmoid
SBF2	11	9776775	488	susie	0.02	3.80E-05	4.77	1.86E-06	7.52E-04	GTEx Whole Blood
SENP8	15	72114257	340	enet	0.03	3.30E-04	-3.52	4.39E-04	3.90E-02	GTEx Colon Transverse
SENP8	15	72114257	351	susie	0.00	1.70E-01	-3.49	4.75E-04	4.08E-02	GTEx Colon Sigmoid
SF3A3	1	37956974	465	enet	0.44	3.20E-162	6.55	5.75E-11	8.54E-08	YFS BLOOD RNAARR
SF3A3	1	37956974	469	lasso	0.43	5.50E-83	5.98	2.22E-09	1.96E-06	GTEx Whole Blood
SF3A3	1	37956974	471	susie	0.10	3.10E-10	5.64	1.75E-08	1.03E-05	GTEx Colon Transverse
SF3A3	1	37956974	472	top1	0.10	5.30E-09	5.61	1.99E-08	9.92E-06	GTEx Colon Sigmoid
SF3A3	1	37956974	479	lasso	0.13	7.20E-39	-4.56	5.22E-06	1.86E-03	NTR BLOOD RNAARR
SFMBT1	3	52903571	358	enet	0.19	5.90E-19	5.54	2.99E-08	1.58E-05	GTEx Colon Transverse
SH2B3	12	111405922	243	enet	0.02	3.30E-07	-4.64	3.54E-06	1.58E-03	YFS BLOOD RNAARR
SIDT2	11	117178735	558	lasso	0.15	2.40E-46	-3.55	3.79E-04	3.53E-02	NTR BLOOD RNAARR
SIDT2	11	117178735	544	enet	0.45	1.30E-168	-3.47	5.26E-04	3.97E-02	YFS BLOOD RNAARR
SLC25A16	10	68477997	401	top1	0.05	6.60E-09	-4.51	6.61E-06	2.10E-03	GTEx Whole Blood
SLC25A16	10	68477997	403	top1	0.07	1.90E-07	-3.94	8.05E-05	1.04E-02	GTEx Colon Transverse
SLC25A16	10	68477997	407	enet	0.03	1.70E-09	-3.52	4.34E-04	3.88E-02	NTR BLOOD RNAARR
SLC30A8	8	116950272	462	enet	0.08	2.40E-07	5.28	1.32E-07	5.48E-05	GTEx Colon Sigmoid
SLC7A9	19	32830508	419	enet	0.13	3.80E-13	-4.32	1.55E-05	3.17E-03	GTEx Colon Transverse
SMIM4	3	52534012	364	top1	0.03	1.30E-05	-3.95	7.95E-05	1.36E-02	GTEx Whole Blood

SOSTDC1	7	16461480	614	lasso	0.09	2.90E-09	3.75	1.76E-04	2.02E-02	GTEx Colon Transverse
SOX4	6	21593750	465	bslmm	0.02	2.80E-07	3.52	4.29E-04	3.48E-02	YFS BLOOD RNAARR
SPPL2A	15	50702265	401	susie	0.05	3.60E-06	-3.46	5.33E-04	4.33E-02	GTEx Colon Transverse
STAB1	3	52495337	365	susie	0.02	4.20E-03	4.48	7.53E-06	1.89E-03	GTEx Colon Transverse
STAB1	3	52495337	363	susie	0.07	1.30E-11	4.26	2.05E-05	4.71E-03	GTEx Whole Blood
STAB1	3	52495337	363	top1	0.05	3.90E-16	3.98	6.82E-05	1.09E-02	YFS BLOOD RNAARR
STAT6	12	57095407	335	lasso	0.73	0.00E+00	-4.36	1.30E-05	3.62E-03	YFS BLOOD RNAARR
STAT6	12	57095407	326	enet	0.30	8.00E-100	3.94	8.23E-05	1.26E-02	NTR BLOOD RNAARR
STIMATE	3	52836218	378	lasso	0.08	1.30E-07	3.50	4.58E-04	4.08E-02	GTEx Colon Sigmoid
SUGT1	13	52652708	403	susie	0.05	1.60E-09	3.90	9.55E-05	1.48E-02	GTEx Whole Blood
TAC3	12	57009999	327	lasso	0.01	3.20E-02	-4.59	4.49E-06	1.57E-03	GTEx Whole Blood
TAGLN	11	117199369	531	enet	0.54	1.30E-213	-3.45	5.54E-04	4.05E-02	YFS BLOOD RNAARR
TBRG4	7	45100099	419	lasso	0.03	1.30E-08	3.61	3.12E-04	3.01E-02	YFS BLOOD RNAARR
TBRG4	7	45100099	432	top1	0.10	5.30E-10	-3.51	4.43E-04	3.90E-02	GTEx Colon Transverse
TIGAR	12	4307762	580	susie	0.06	7.80E-11	4.07	4.71E-05	8.71E-03	GTEx Whole Blood
TMBIM1	2	218274196	435	susie	0.10	2.90E-09	4.62	3.90E-06	1.08E-03	GTEx Colon Sigmoid
TMBIM1	2	218274196	433	top1	0.06	1.50E-06	4.53	6.01E-06	1.59E-03	GTEx Colon Transverse
TMEM258	11	61768500	398	top1	0.09	3.60E-08	-7.64	2.18E-14	3.62E-11	GTEx Colon Sigmoid
TMEM258	11	61768500	376	lasso	0.05	2.70E-09	-5.97	2.36E-09	1.96E-06	GTEx Whole Blood
TMEM80	11	695590	456	top1	0.01	6.60E-03	3.95	7.69E-05	1.26E-02	NTR BLOOD RNAARR
TMX4	20	7977345	559	bslmm	0.02	1.90E-08	6.30	2.94E-10	2.10E-07	NTR BLOOD RNAARR
TMX4	20	7977345	508	lasso	0.04	3.20E-08	3.49	4.80E-04	4.84E-02	GTEx Whole Blood
TNFAIP8L3	15	51056597	431	lasso	0.05	8.40E-05	3.67	2.38E-04	2.58E-02	GTEx Colon Sigmoid
TRIM26	6	30184454	24	top1	0.03	1.70E-09	4.03	5.67E-05	9.36E-03	YFS BLOOD RNAARR
TRIM4	7	99876957	280	top1	0.14	2.60E-43	4.08	4.43E-05	9.37E-03	NTR BLOOD RNAARR

TRIM4	7	99876957	260	susie	0.44	4.10E-42	-4.04	5.32E-05	8.28E-03	GTEx Colon Sigmoid
TRIM4	7	99876957	242	enet	0.48	5.60E-54	-3.99	6.67E-05	9.45E-03	GTEx Colon Transverse
TRIM4	7	99876957	222	top1	0.24	5.40E-42	-3.81	1.40E-04	1.85E-02	GTEx Whole Blood
TRIM4	7	99876957	276	top1	0.24	9.00E-78	-3.81	1.40E-04	1.89E-02	YFS BLOOD RNAARR
TTC33	5	40512332	428	top1	0.00	1.90E-01	3.91	9.38E-05	1.20E-02	GTEx Colon Sigmoid
UPB1	22	24494106	398	susie	0.13	4.60E-11	3.63	2.87E-04	2.86E-02	GTEx Colon Sigmoid
USO1	4	75724576	458	susie	0.06	3.70E-10	3.71	2.05E-04	2.48E-02	GTEx Whole Blood
USP44	12	95516559	613	enet	0.07	7.50E-07	3.42	6.17E-04	4.88E-02	GTEx Colon Sigmoid
UTP11	1	38009257	463	lasso	0.05	7.00E-09	5.30	1.15E-07	6.96E-05	GTEx Whole Blood
UTP23	8	116766504	426	susie	0.02	2.10E-04	-6.43	1.28E-10	2.06E-07	GTEx Whole Blood
UVSSA	4	1345690	376	susie	0.19	1.10E-16	3.50	4.68E-04	4.08E-02	GTEx Colon Sigmoid
VPS29	12	110491082	230	bslmm	0.02	6.60E-07	-4.06	4.81E-05	9.37E-03	NTR BLOOD RNAARR
VPS36	13	52412601	379	enet	0.02	1.50E-06	3.79	1.53E-04	2.00E-02	YFS BLOOD RNAARR
WDR43	2	28894666	589	susie	0.03	8.50E-06	-3.63	2.82E-04	3.03E-02	GTEx Whole Blood
WDR43	2	28894666	593	top1	0.03	2.30E-04	-3.57	3.57E-04	3.52E-02	GTEx Colon Transverse
WNT4	1	22117312	483	top1	0.03	7.20E-04	-6.88	5.86E-12	6.19E-09	GTEx Colon Transverse
WNT4	1	22117312	482	enet	0.00	6.10E-02	-4.42	9.91E-06	3.00E-03	GTEx Whole Blood
YWHAH	22	31944521	471	lasso	0.13	2.40E-40	3.67	2.46E-04	2.67E-02	YFS BLOOD RNAARR
ZNF213	16	3129776	387	blup	0.01	1.10E-03	3.90	9.57E-05	1.37E-02	NTR BLOOD RNAARR
ZNF324B	19	58451610	296	enet	0.02	2.10E-03	3.56	3.67E-04	3.52E-02	GTEx Colon Transverse
ZNF329	19	58126247	404	enet	0.07	1.60E-06	4.55	5.28E-06	1.38E-03	GTEx Colon Sigmoid
ZNF408	11	46701029	237	susie	0.03	5.60E-04	3.52	4.28E-04	3.90E-02	GTEx Colon Transverse
ZNF446	19	58474016	278	top1	0.03	2.00E-09	-3.56	3.69E-04	3.13E-02	YFS BLOOD RNAARR
ZNF79	9	127424373	359	enet	0.15	8.60E-13	-3.60	3.19E-04	3.12E-02	GTEx Colon Sigmoid

TSS: transcription start site; FDR: false discovery rate.

Supplementary Table 3. Putative susceptibility genes for CRC risk verified by colocalization analysis at a PP4 > 0.75.

Gene	Chr	TSS (hg38)	TWAS Z	TWAS P	Best GWAS SNP	Best GWAS Z	Best eQTL SNP	Best eQTL Z	PP0	PP1	PP2	PP3	PP4	Tissue
AAMP	2	218264128	5.09	3.51E-07	rs2168704	-5.02	rs992157	-4.90	0.00	0.00	0.00	0.02	0.97	GTEx Colon Sigmoid
AAMP	2	218264128	4.91	9.26E-07	rs2168704	-5.05	rs10169718	-4.04	0.01	0.01	0.08	0.04	0.86	GTEx Colon Transverse
AAMP	2	218264128	4.62	3.91E-06	rs2168704	-5.11	rs10932765	-4.81	0.01	0.01	0.07	0.10	0.81	YFS BLOOD RNAARR
ABHD12B	14	50872052	-4.36	1.29E-05	rs8004788	4.45	rs17123103	-6.54	0.00	0.04	0.00	0.01	0.96	GTEx Whole Blood
ABHD12B	14	50872052	-4.36	1.29E-05	rs8004788	4.45	rs17123103	-7.02	0.00	0.04	0.00	0.01	0.96	NTR BLOOD RNAARR
ABHD12B	14	50872052	-4.29	1.82E-05	rs8004788	4.45	rs17123103	-3.93	0.15	0.04	0.04	0.01	0.77	GTEx Colon Sigmoid
ABHD12B	14	50872052	-4.28	1.91E-05	rs8004788	4.45	rs12589665	-8.12	0.00	0.03	0.00	0.01	0.96	GTEx Colon Transverse
ABHD12B	14	50872052	-4.09	4.31E-05	rs8004788	4.45	rs10132049	-5.30	0.01	0.10	0.00	0.03	0.86	YFS BLOOD RNAARR
ACTR1B	2	97655938	-4.40	1.09E-05	rs11692435	-4.40	rs11692435	10.26	0.00	0.03	0.00	0.00	0.97	GTEx Whole Blood
ACTR1B	2	97655938	-4.18	2.86E-05	rs11692435	-4.40	rs11692435	10.06	0.00	0.03	0.00	0.00	0.97	GTEx Colon Transverse
ACTR1B	2	97655938	-3.44	5.76E-04	rs11692435	-4.40	rs11692435	9.08	0.00	0.03	0.00	0.00	0.97	GTEx Colon Sigmoid
ARPC2	2	218217140	5.04	4.65E-07	rs2168704	-5.08	rs6720449	-4.64	0.00	0.01	0.00	0.05	0.94	NTR BLOOD RNAARR
ARPC2	2	218217140	4.79	1.67E-06	rs2168704	-5.08	rs12612347	-5.67	0.00	0.01	0.00	0.05	0.95	GTEx Colon Transverse
ARPC2	2	218217140	4.77	1.87E-06	rs2168704	-5.05	rs12612347	-5.33	0.00	0.01	0.01	0.05	0.94	GTEx Colon Sigmoid
ARPC2	2	218217140	4.38	1.21E-05	rs2168704	-5.08	rs17462354	-5.36	0.00	0.01	0.00	0.07	0.92	GTEx Whole Blood
ASPDH	19	50511599	-5.17	2.32E-07	rs2445828	-5.83	rs2445828	6.82	0.00	0.00	0.00	0.00	1.00	GTEx Colon Transverse
ASPDH	19	50511599	-4.46	8.16E-06	rs2445828	-5.83	rs3745518	5.18	0.00	0.00	0.00	0.03	0.97	GTEx Colon Sigmoid
ATF1	12	50763709	7.04	1.89E-12	rs6580735	7.12	rs11169571	5.64	0.00	0.00	0.00	0.04	0.96	GTEx Colon Transverse
ATF1	12	50763709	7.04	1.91E-12	rs6580735	7.12	rs11169571	8.54	0.00	0.00	0.00	0.03	0.97	GTEx Colon Sigmoid
AXIN1	16	287439	4.52	6.07E-06	rs400037	-4.52	rs400037	-20.76	0.00	0.02	0.00	0.00	0.98	YFS BLOOD RNAARR
B9D2	19	41354416	-6.03	1.61E-09	rs2241714	-6.11	rs1046909	5.04	0.00	0.00	0.00	0.03	0.97	YFS BLOOD RNAARR

BAMBI	10	28677509	4.27	1.97E-05	rs1761985	4.39	rs2797470	12.91	0.00	0.09	0.00	0.01	0.90	GTEx Whole Blood
BAMBI	10	28677509	3.44	5.72E-04	rs1761985	4.39	rs2065695	17.95	0.00	0.09	0.00	0.01	0.90	YFS BLOOD RNAARR
BRD3	9	134030304	-4.04	5.32E-05	rs11789898	4.04	rs11789898	-6.40	0.00	0.13	0.00	0.00	0.87	GTEx Colon Transverse
C11orf53	11	111245724	-8.54	1.30E-17	rs12296076	8.55	rs6589218	-9.54	0.00	0.00	0.00	0.00	1.00	GTEx Colon Transverse
C1orf122	1	37806978	-5.38	7.44E-08	rs4072980	-5.68	rs7366048	4.12	0.00	0.00	0.03	0.01	0.97	GTEx Whole Blood
CACNA1I	22	39570752	-4.20	2.64E-05	rs5757573	5.38	rs5757762	-3.94	0.04	0.01	0.16	0.03	0.77	GTEx Whole Blood
CDH1	16	68737291	4.70	2.66E-06	rs8056538	-5.00	rs12597188	-4.09	0.02	0.01	0.08	0.03	0.87	GTEx Colon Sigmoid
CERS5	12	50129288	-6.98	2.96E-12	rs7315690	6.78	rs7315690	-4.04	0.00	0.00	0.06	0.03	0.91	GTEx Colon Transverse
COLCA2	11	111298545	-8.11	5.18E-16	rs12296076	8.60	rs3087967	-11.68	0.00	0.00	0.00	0.03	0.98	GTEx Colon Transverse
COLCA2	11	111298545	-5.95	2.62E-09	rs12296076	8.60	rs7130173	-4.09	0.00	0.00	0.04	0.01	0.95	GTEx Colon Sigmoid
COX14	12	50112081	6.31	2.76E-10	rs7315690	6.78	rs17124432	5.27	0.00	0.00	0.00	0.15	0.85	GTEx Colon Transverse
COX14	12	50112081	6.31	2.87E-10	rs7315690	6.78	rs7961065	7.48	0.00	0.00	0.00	0.19	0.81	GTEx Colon Sigmoid
COX14	12	50112081	5.16	2.50E-07	rs7315690	6.79	rs7961065	7.60	0.00	0.00	0.00	0.23	0.77	GTEx Whole Blood
CRTC3	15	90529922	-4.10	4.10E-05	rs11852389	-4.62	rs12708583	5.11	0.00	0.15	0.00	0.06	0.79	YFS BLOOD RNAARR
DENND4C	9	19230434	4.41	1.05E-05	rs17818670	5.11	rs7043344	7.59	0.00	0.03	0.00	0.07	0.90	YFS BLOOD RNAARR
DNA2	10	68414063	4.11	4.03E-05	rs10998195	-4.51	rs2031098	-9.86	0.00	0.02	0.00	0.01	0.97	GTEx Colon Transverse
DNA2	10	68414063	3.85	1.21E-04	rs10998195	-4.51	rs10823215	-10.24	0.00	0.16	0.00	0.05	0.79	GTEx Colon Sigmoid
DNA2	10	68414063	3.80	1.42E-04	rs10998195	-4.51	rs2031098	-7.93	0.00	0.02	0.00	0.01	0.97	GTEx Whole Blood
FADS1	11	61799626	7.83	4.96E-15	rs4246215	-8.13	rs1535	-6.00	0.00	0.00	0.00	0.04	0.96	GTEx Colon Sigmoid
FADS1	11	61799626	-4.24	2.20E-05	rs4246215	-8.13	rs968567	5.53	0.00	0.00	0.00	0.16	0.84	GTEx Whole Blood
FAM89B	11	65572348	5.35	8.94E-08	rs4099470	-5.35	rs4099470	-4.87	0.00	0.00	0.00	0.00	1.00	GTEx Whole Blood
FAM89B	11	65572348	4.37	1.27E-05	rs4099470	-5.35	rs11227226	-8.92	0.00	0.00	0.00	0.00	1.00	YFS BLOOD RNAARR
FNIP2	4	158769025	-4.09	4.36E-05	rs13152601	4.47	rs4690916	-3.96	0.13	0.05	0.03	0.01	0.79	GTEx Colon Transverse
FNIP2	4	158769025	-4.02	5.89E-05	rs13152601	4.47	rs10010963	-8.61	0.00	0.08	0.00	0.02	0.91	NTR BLOOD RNAARR
FUT2	19	48695970	-4.33	1.48E-05	rs601338	4.38	rs516246	-9.40	0.00	0.04	0.00	0.00	0.96	GTEx Colon Transverse

GABBR1	6	29555628	-4.05	5.14E-05	rs9257940	4.72	rs9257940	-4.39	0.04	0.01	0.01	0.00	0.94	YFS BLOOD RNAARR
GNA12	7	2728104	-4.26	2.05E-05	rs798502	-5.02	rs798502	6.23	0.00	0.00	0.00	0.01	0.99	GTEx Colon Transverse
GNA12	7	2728104	-4.01	5.98E-05	rs798502	-5.02	rs17132741	-6.89	0.00	0.03	0.00	0.09	0.88	GTEx Whole Blood
GPATCH1	19	33080898	-5.15	2.66E-07	rs10411210	-7.57	rs12460349	5.59	0.00	0.00	0.00	0.13	0.87	GTEx Whole Blood
GPATCH1	19	33080898	-3.39	7.03E-04	rs10411210	-7.57	rs2216594	4.77	0.00	0.00	0.01	0.03	0.97	YFS BLOOD RNAARR
GPBAR1	2	218259495	-5.01	5.38E-07	rs2168704	-5.12	rs736730	9.15	0.00	0.00	0.00	0.02	0.98	YFS BLOOD RNAARR
HLA-F	6	29722774	4.79	1.68E-06	rs9257940	4.72	rs2523405	-4.65	0.01	0.06	0.01	0.02	0.90	GTEx Colon Sigmoid
IQGAP1	15	90388241	3.72	2.00E-04	rs11852389	-4.62	rs2657947	-7.32	0.00	0.16	0.00	0.06	0.78	NTR BLOOD RNAARR
LAMC1	1	183023419	6.94	3.82E-12	rs10752881	6.97	rs12739316	-14.62	0.00	0.00	0.00	0.04	0.96	YFS BLOOD RNAARR
LAMC1	1	183023419	6.79	1.12E-11	rs10752881	6.97	rs12739316	-11.83	0.00	0.00	0.00	0.04	0.96	GTEx Whole Blood
LAMC1	1	183023419	6.71	1.91E-11	rs10752881	6.97	rs12739316	-9.42	0.00	0.00	0.00	0.07	0.93	NTR BLOOD RNAARR
LAMC1	1	183023419	-6.57	5.01E-11	rs10752881	6.97	rs1051473	-4.60	0.00	0.00	0.02	0.07	0.91	GTEx Colon Sigmoid
LAMC1	1	183023419	-5.35	8.69E-08	rs10752881	6.97	rs3768622	-4.44	0.00	0.00	0.02	0.05	0.93	GTEx Colon Transverse
LIMA1	12	50175787	6.94	4.05E-12	rs7315690	6.94	rs7315690	9.41	0.00	0.00	0.00	0.00	1.00	YFS BLOOD RNAARR
LIMA1	12	50175787	-6.62	3.61E-11	rs7315690	6.89	rs7296291	-5.13	0.00	0.00	0.01	0.03	0.96	GTEx Colon Sigmoid
LIMA1	12	50175787	6.54	6.28E-11	rs7315690	6.89	rs7296291	5.44	0.00	0.00	0.00	0.05	0.96	GTEx Whole Blood
LMOD1	1	201896455	-5.02	5.25E-07	rs2820313	5.28	rs2644122	-5.40	0.00	0.00	0.00	0.02	0.98	GTEx Colon Transverse
LRP1	12	57128482	3.83	1.30E-04	rs324015	-5.33	rs715948	-5.59	0.00	0.00	0.00	0.01	0.98	GTEx Colon Transverse
METRNL	17	83079608	6.40	1.55E-10	rs7502442	-6.56	rs7502442	-5.43	0.00	0.00	0.00	0.00	1.00	GTEx Whole Blood
MYO9A	15	71822290	-4.23	2.36E-05	rs4777489	4.43	rs4777489	-5.98	0.00	0.07	0.00	0.05	0.88	YFS BLOOD RNAARR
MYO9A	15	71822290	-4.19	2.76E-05	rs4777489	4.43	rs4777471	-4.30	0.04	0.07	0.03	0.04	0.82	GTEx Whole Blood
NAA38	17	7856684	4.18	2.95E-05	rs11078711	4.14	rs11078711	6.77	0.00	0.11	0.00	0.00	0.89	GTEx Colon Sigmoid
NDFIP1	5	142108778	4.23	2.33E-05	rs1062158	4.28	rs11739961	6.28	0.00	0.06	0.00	0.01	0.93	GTEx Whole Blood
NID2	14	52004808	3.98	6.92E-05	rs1497077	-4.23	rs1151582	5.95	0.00	0.16	0.00	0.01	0.84	GTEx Colon Transverse
NIN	14	50719762	-4.40	1.08E-05	rs8004788	4.45	rs1959527	-5.46	0.00	0.04	0.00	0.01	0.95	GTEx Colon Transverse

NTN5	19	48661406	3.88	1.05E-04	rs601338	4.38	rs516246	6.15	0.00	0.04	0.00	0.00	0.96	GTEx Whole Blood
NXN	17	799309	-3.83	1.30E-04	rs1703824	6.96	rs1703824	-5.41	0.00	0.00	0.00	0.00	1.00	GTEx Whole Blood
PDGFB	22	39223358	-4.31	1.62E-05	rs5757573	5.38	rs5757573	-7.16	0.00	0.00	0.00	0.00	1.00	GTEx Whole Blood
PNKD	2	218269650	4.76	1.92E-06	rs2168704	-5.05	rs10193189	-8.82	0.00	0.01	0.00	0.10	0.89	GTEx Whole Blood
PREX1	20	48624251	4.82	1.41E-06	rs6066825	-8.80	rs12106159	-3.62	0.00	0.00	0.22	0.03	0.76	GTEx Colon Sigmoid
PYGL	14	50857890	-3.71	2.07E-04	rs8004788	4.45	rs12589665	-7.87	0.00	0.05	0.00	0.02	0.93	GTEx Colon Transverse
RASIP1	19	48720584	4.16	3.13E-05	rs601338	4.38	rs602662	4.88	0.01	0.09	0.00	0.01	0.90	GTEx Colon Transverse
RASIP1	19	48720584	3.99	6.67E-05	rs601338	4.38	rs973579	7.16	0.00	0.07	0.00	0.01	0.92	GTEx Colon Sigmoid
RPS5	19	58386399	3.94	8.31E-05	rs11670864	4.21	rs734379	-5.90	0.00	0.19	0.00	0.01	0.80	GTEx Colon Sigmoid
RUFY2	10	68341106	-4.20	2.64E-05	rs10998195	-4.51	rs3199937	6.56	0.00	0.07	0.00	0.02	0.91	GTEx Colon Sigmoid
RUFY2	10	68341106	-3.85	1.18E-04	rs10998195	-4.51	rs10998074	6.24	0.00	0.07	0.00	0.02	0.91	GTEx Colon Transverse
SBF2	11	9776775	4.77	1.86E-06	rs4399321	4.40	rs11042686	5.15	0.00	0.05	0.00	0.00	0.95	GTEx Whole Blood
SF3A3	1	37956974	6.55	5.75E-11	rs4072980	-5.68	rs4072980	-19.37	0.00	0.00	0.00	0.00	1.00	YFS BLOOD RNAARR
SF3A3	1	37956974	5.64	1.75E-08	rs4072980	-5.68	rs7366048	-6.44	0.00	0.00	0.00	0.00	1.00	GTEx Colon Transverse
SF3A3	1	37956974	5.61	1.99E-08	rs4072980	-5.68	rs7366048	-5.94	0.00	0.00	0.00	0.00	1.00	GTEx Colon Sigmoid
SF3A3	1	37956974	-4.56	5.22E-06	rs4072980	-5.68	rs11485595	12.56	0.00	0.00	0.00	0.17	0.83	NTR BLOOD RNAARR
SFMBT1	3	52903571	5.54	2.99E-08	rs2001732	-5.36	rs9847710	8.36	0.00	0.02	0.00	0.11	0.87	GTEx Colon Transverse
SLC25A16	10	68477997	-4.51	6.61E-06	rs10998195	-4.51	rs10998195	6.10	0.00	0.02	0.00	0.01	0.98	GTEx Whole Blood
SLC25A16	10	68477997	-3.94	8.05E-05	rs10998195	-4.51	rs12219699	5.29	0.02	0.16	0.01	0.05	0.76	GTEx Colon Transverse
SOX4	6	21593750	3.52	4.29E-04	rs12530233	-4.18	rs12530233	-5.00	0.01	0.13	0.00	0.00	0.85	YFS BLOOD RNAARR
TAC3	12	57009999	-4.59	4.49E-06	rs324015	-5.33	rs324015	3.56	0.03	0.00	0.10	0.01	0.86	GTEx Whole Blood
TBRG4	7	45100099	3.61	3.12E-04	rs7810512	-4.44	rs11765574	-5.99	0.00	0.11	0.00	0.01	0.88	YFS BLOOD RNAARR
TMBIM1	2	218274196	4.62	3.90E-06	rs2168704	-4.98	rs3817266	-6.41	0.00	0.01	0.00	0.07	0.92	GTEx Colon Sigmoid
TMBIM1	2	218274196	4.53	6.01E-06	rs2168704	-5.01	rs1017698	-5.35	0.00	0.02	0.01	0.12	0.86	GTEx Colon Transverse
TMEM258	11	61768500	-7.64	2.18E-14	rs4246215	-8.13	rs174538	5.45	0.00	0.00	0.01	0.14	0.85	GTEx Colon Sigmoid

TMEM258	11	61768500	-5.97	2.36E-09	rs4246215	-8.13	rs968567	6.04	0.00	0.00	0.00	0.05	0.96	GTEx Whole Blood
TRIM26	6	30184454	4.03	5.67E-05	rs2272874	4.42	rs1362126	-6.03	0.00	0.13	0.00	0.03	0.84	YFS BLOOD RNAARR
TRIM4	7	99876957	4.08	4.43E-05	rs2527927	-4.54	rs2572009	-13.40	0.00	0.10	0.00	0.05	0.85	NTR BLOOD RNAARR
TRIM4	7	99876957	-4.04	5.32E-05	rs2527927	-4.54	rs2572019	11.90	0.00	0.13	0.00	0.07	0.81	GTEx Colon Sigmoid
TRIM4	7	99876957	-3.99	6.67E-05	rs2527927	-4.54	rs2572019	13.26	0.00	0.14	0.00	0.07	0.79	GTEx Colon Transverse
UTP11	1	38009257	5.30	1.15E-07	rs4072980	-5.68	rs12138115	-6.28	0.00	0.00	0.00	0.02	0.99	GTEx Whole Blood
WNT4	1	22117312	-6.88	5.86E-12	rs7524102	-6.96	rs12568930	3.89	0.00	0.00	0.09	0.00	0.91	GTEx Colon Transverse

TSS: transcription start site.

Supplementary Table 4. Susceptibility genes with high evidence of associating with CRC risk identified by single-tissue TWAS.

Gene	Chr	TSS (hg38)	TWAS Z	TWAS P	Best tissue	Category	Reference	Strategy	Direction*
AAMP	2	218264128	5.09	3.51E-07	GTEx Colon Sigmoid	Reported	36539618	TWAS	-
ABHD12B	14	50872052	-4.36	1.29E-05	GTEx Whole Blood	Reported	36539618	TWAS	-
ACTR1B	2	97655938	-4.40	1.09E-05	GTEx Whole Blood	Reported	33058866	TWAS	TRUE
ARPC2	2	218217140	5.04	4.65E-07	NTR BLOOD RNAARR	Reported	36539618	TWAS	-
ASPDH	19	50511599	-5.17	2.32E-07	GTEx Colon Transverse	Novel	-	-	-
ATF1	12	50763709	7.04	1.89E-12	GTEx Colon Transverse	Reported	33481017	Multi-omics	-
AXIN1	16	287439	4.52	6.07E-06	YFS BLOOD RNAARR	Novel	-	-	-
B9D2	19	41354416	-6.03	1.61E-09	YFS BLOOD RNAARR	Reported	36539618	TWAS	-
BAMBI	10	28677509	4.27	1.97E-05	GTEx Whole Blood	Reported	36539618	TWAS	-
BRD3	9	134030304	-4.04	5.32E-05	GTEx Colon Transverse	Reported	36539618	TWAS	-
C11orf53	11	111245724	-8.54	1.30E-17	GTEx Colon Transverse	Reported	33058866	TWAS	TRUE
C1orf122	1	37806978	-5.38	7.44E-08	GTEx Whole Blood	Novel	-	-	-
CDH1	16	68737291	4.70	2.66E-06	GTEx Colon Sigmoid	Reported	36539618	TWAS	-
COLCA2	11	111298545	-8.11	5.18E-16	GTEx Colon Transverse	Reported	33058866	TWAS	TRUE
COX14	12	50112081	6.31	2.76E-10	GTEx Colon Transverse	Reported	36539618	TWAS	-
CRTC3	15	90529922	-4.10	4.10E-05	YFS BLOOD RNAARR	Novel	-	-	-
DENND4C	9	19230434	4.41	1.05E-05	YFS BLOOD RNAARR	Novel	-	-	-
DNA2	10	68414063	4.11	4.03E-05	GTEx Colon Transverse	Reported	33134313	Candidate gene	TRUE
FADS1	11	61799626	7.83	4.96E-15	GTEx Colon Sigmoid	Reported	24836286	Multi-omics	TRUE
FAM89B	11	65572348	5.35	8.94E-08	GTEx Whole Blood	Novel	-	-	-
FNIP2	4	158769025	-4.09	4.36E-05	GTEx Colon Transverse	Novel	-	-	-
FUT2	19	48695970	-4.33	1.48E-05	GTEx Colon Transverse	Reported	33481017	Multi-omics	-
GABBR1	6	29555628	-4.05	5.14E-05	YFS BLOOD RNAARR	Reported	36539618	TWAS	-

GNA12	7	2728104	-4.26	2.05E-05	GTEx Colon Transverse	Reported	36539618	TWAS	-
GPATCH1	19	33080898	-5.15	2.66E-07	GTEx Whole Blood	Reported	36539618	TWAS	-
GPBAR1	2	218259495	-5.01	5.38E-07	YFS BLOOD RNAARR	Reported	32833970	TWAS	-
HLA-F	6	29722774	4.79	1.68E-06	GTEx Colon Sigmoid	Reported	36539618	TWAS	-
IQGAP1	15	90388241	3.72	2.00E-04	NTR BLOOD RNAARR	Reported	36539618	TWAS	-
LAMC1	1	183023419	6.94	3.82E-12	YFS BLOOD RNAARR	Reported	36539618	TWAS	-
LIMA1	12	50175787	6.94	4.05E-12	YFS BLOOD RNAARR	Reported	36539618	TWAS	-
LMOD1	1	201896455	-5.02	5.25E-07	GTEx Colon Transverse	Reported	36539618	TWAS	-
LRP1	12	57128482	3.83	1.30E-04	GTEx Colon Transverse	Reported	33058866	TWAS	TRUE
METRNL	17	83079608	6.40	1.55E-10	GTEx Whole Blood	Novel	-	-	-
MYO9A	15	71822290	-4.23	2.36E-05	YFS BLOOD RNAARR	Reported	36539618	TWAS	-
NAA38	17	7856684	4.18	2.95E-05	GTEx Colon Sigmoid	Novel	-	-	-
NDFIP1	5	142108778	4.23	2.33E-05	GTEx Whole Blood	Novel	-	-	-
NID2	14	52004808	3.98	6.92E-05	GTEx Colon Transverse	Reported	36539618	TWAS	-
NIN	14	50719762	-4.40	1.08E-05	GTEx Colon Transverse	Reported	36539618	TWAS	-
NTN5	19	48661406	3.88	1.05E-04	GTEx Whole Blood	Novel	-	-	-
NXN	17	799309	-3.83	1.30E-04	GTEx Whole Blood	Reported	36539618	TWAS	-
PDGFB	22	39223358	-4.31	1.62E-05	GTEx Whole Blood	Reported	36539618	TWAS	-
PNKD	2	218269650	4.76	1.92E-06	GTEx Whole Blood	Reported	36539618	TWAS	-
PREX1	20	48624251	4.82	1.41E-06	GTEx Colon Sigmoid	Reported	36539618	TWAS	-
RASIP1	19	48720584	3.99	6.67E-05	GTEx Colon Sigmoid	Novel	-	-	-
RPS5	19	58386399	3.94	8.31E-05	GTEx Colon Sigmoid	Reported	36539618	TWAS	-
RUFY2	10	68341106	-4.20	2.64E-05	GTEx Colon Sigmoid	Novel	-	-	-
SBF2	11	9776775	4.77	1.86E-06	GTEx Whole Blood	Novel	-	-	-
SF3A3	1	37956974	6.55	5.75E-11	YFS BLOOD RNAARR	Reported	33058866	TWAS	TRUE

SFMBT1	3	52903571	5.54	2.99E-08	GTEx Colon Transverse	Reported	33058866	TWAS	TRUE
SLC25A16	10	68477997	-4.51	6.61E-06	GTEx Whole Blood	Novel	-	-	-
SOX4	6	21593750	3.52	4.29E-04	YFS BLOOD RNAARR	Novel	-	-	-
TAC3	12	57009999	-4.59	4.49E-06	GTEx Whole Blood	Novel	-	-	-
TBRG4	7	45100099	3.61	3.12E-04	YFS BLOOD RNAARR	Reported	36539618	TWAS	-
TMBIM1	2	218274196	4.62	3.90E-06	GTEx Colon Sigmoid	Reported	36539618	TWAS	-
TMEM258	11	61768500	-5.97	2.36E-09	GTEx Whole Blood	Reported	36539618	TWAS	-
TRIM26	6	30184454	4.03	5.67E-05	YFS BLOOD RNAARR	Reported	36539618	TWAS	-
TRIM4	7	99876957	4.08	4.43E-05	NTR BLOOD RNAARR	Reported	36539618	TWAS	-
WNT4	1	22117312	-6.88	5.86E-12	GTEx Colon Transverse	Reported	36539618	TWAS	-

* If the direcction of the TWAS association is consistent with that reported in previous studies, it is marked as TRUE. "-" represents that the corresponding information is missing in the previous studies.

TSS: transcription start site.

Supplementary Table 5. Significant candidate genes for CRC risk identified by across-tissue TWAS (FDR < 0.05 & PP4 > 0.75 & pass conditional test).

Gene	Chr	TSS (hg38)	NSNP	Model	Prediction R2	Prediction P	TWAS Z	TWAS P	FDR	Best GWAS SNP	Best GWAS Z	Best eQTL SNP	Best eQTL Z	PP0	PP1	PP2	PP3	PP4	Pass conditional test	Category	Reference	Strategy	Direction *
ACTR1B	2	97655938	143	lasso	0.44	2.00E-40	-3.87	1.08E-04	1.96E-02	rs11692435	-4.40	rs11692435	10.86	0.00	0.03	0.00	0.00	0.97	Yes	Reported	36539618	TWAS	-
ADRM1	20	62302092	506	lasso	0.00	4.60E-01	-3.79	1.50E-04	2.22E-02	rs1570027	-11.31	rs6142925	-3.48	0.00	0.00	0.83	0.06	0.11	-	-	-	-	-
APOA1	11	116835750	535	lasso	0.03	3.50E-03	-3.55	3.85E-04	4.23E-02	rs1351452	4.04	rs7122944	-4.39	0.17	0.23	0.01	0.02	0.57	-	-	-	-	-
ARFGEF2	20	48921710	442	top1	0.07	1.20E-06	6.36	2.06E-10	5.04E-07	rs6066825	-8.80	rs729663	-5.79	0.00	0.00	0.01	0.99	0.00	-	-	-	-	-
ARHGAP21	10	24583608	472	enet	0.00	1.70E-01	3.73	1.88E-04	2.59E-02	rs7081091	-4.13	rs11013998	-3.57	0.92	0.06	0.01	0.00	0.00	-	-	-	-	-
ARPC2	2	218217140	452	top1	0.43	2.30E-39	4.62	3.91E-06	1.59E-03	rs2168704	-5.05	rs10206984	-11.51	0.00	0.01	0.00	0.09	0.90	Yes	Reported	36539618	TWAS	-
ATF1	12	50763709	309	enet	0.18	3.20E-15	5.66	1.51E-08	1.48E-05	rs6580735	7.16	rs10783387	7.65	0.00	0.00	0.00	0.05	0.95	Yes	Reported	36539618	TWAS	-
B9D2	19	41354416	379	enet	0.05	2.30E-05	-3.87	1.07E-04	1.96E-02	rs2241714	-6.11	rs1115885	-4.62	0.00	0.00	0.59	0.40	0.01	-	-	-	-	-
BRD3	9	134030304	588	enet	0.45	8.90E-42	4.23	2.32E-05	6.14E-03	rs11789898	4.04	rs11789898	10.85	0.00	0.13	0.00	0.00	0.87	Yes	Reported	36539618	TWAS	-
CABLES2	20	62388633	521	enet	0.68	1.50E-77	8.41	4.06E-17	1.99E-13	rs1570027	-11.31	rs747949	-13.74	0.00	0.00	0.00	1.00	0.00	-	-	-	-	-
CEP170	1	243124427	287	lasso	0.31	2.20E-26	-3.58	3.47E-04	4.05E-02	rs7518350	3.90	rs12741781	-9.81	0.00	0.31	0.00	0.01	0.68	-	-	-	-	-
CEP68	2	65056353	427	lasso	0.47	5.90E-44	-3.49	4.76E-04	4.75E-02	rs11675107	-3.61	rs1009358	-11.85	0.00	0.48	0.00	0.00	0.52	-	-	-	-	-
CEP89	19	32875924	440	lasso	0.16	2.40E-13	4.59	4.51E-06	1.77E-03	rs10411210	-7.57	rs10411735	6.28	0.00	0.00	0.00	1.00	0.00	-	-	-	-	-
CERS5	12	50129288	309	enet	0.17	4.90E-14	5.63	1.80E-08	1.60E-05	rs7138420	6.87	rs3184122	7.10	0.00	0.00	0.00	0.03	0.97	No	-	-	-	-
CKAP5	11	46743047	264	lasso	0.06	4.30E-06	-3.49	4.85E-04	4.78E-02	rs2306029	4.10	rs1872896	-5.25	0.00	0.32	0.00	0.04	0.64	-	-	-	-	-
CNEP1R1	16	50024409	383	lasso	0.01	3.60E-02	-3.89	9.97E-05	1.96E-02	rs9807005	3.63	rs7204293	3.88	0.89	0.09	0.01	0.00	0.01	-	-	-	-	-
COX14	12	50112081	308	top1	0.34	1.60E-29	6.29	3.15E-10	6.17E-07	rs7138420	6.87	rs17124432	10.27	0.00	0.00	0.00	0.21	0.79	Yes	Reported	36539618	TWAS	-
COX15	10	99710867	448	enet	0.04	3.10E-04	-5.14	2.76E-07	1.93E-04	rs11190164	7.85	rs7084921	-3.80	0.00	0.00	0.89	0.10	0.01	-	-	-	-	-

CRAT	9	129094141	380	top1	0.47	1.00E-43	-3.57	3.56E-04	4.10E-02	rs2541164	3.81	rs2768630	-12.00	0.00	0.45	0.00	0.02	0.53	-	-	-	-	-
CUL3	2	224470149	435	lasso	0.12	2.60E-10	-3.50	4.61E-04	4.75E-02	rs10181622	-4.10	rs12623277	-6.21	0.00	0.59	0.00	0.03	0.38	-	-	-	-	-
DACT1	14	58633966	483	enet	0.05	7.50E-05	-5.02	5.28E-07	3.23E-04	rs2163616	-5.49	rs17095008	4.42	0.00	0.00	0.02	0.01	0.98	Yes	Reported	36539618	TWAS	-
DCAF12	9	34086386	325	enet	0.08	1.60E-07	4.26	2.09E-05	5.68E-03	rs11557154	5.67	rs11557154	5.03	0.00	0.00	0.00	0.00	1.00	Yes	Novel	-	-	-
DCAF17	2	171434216	346	top1	0.00	1.30E-01	-3.58	3.48E-04	4.05E-02	rs7580132	-3.64	rs2292814	4.09	0.90	0.04	0.02	0.00	0.04	-	-	-	-	-
DCBLD1	6	117453816	420	lasso	0.14	4.30E-12	3.75	1.76E-04	2.53E-02	rs9401003	4.12	rs4946259	-7.11	0.00	0.26	0.00	0.01	0.73	-	-	-	-	-
DIP2B	12	50504984	295	enet	0.26	3.20E-22	5.91	3.46E-09	4.84E-06	rs10783387	7.08	rs2280503	8.98	0.00	0.00	0.00	0.83	0.17	-	-	-	-	-
DNA2	10	68414063	384	lasso	0.54	2.50E-53	3.92	9.01E-05	1.88E-02	rs10998195	-4.51	rs12217591	-12.57	0.00	0.44	0.00	0.13	0.43	-	-	-	-	-
DRAM1	12	101877579	439	enet	0.19	1.80E-15	-3.51	4.49E-04	4.72E-02	rs11111013	4.15	rs7955730	-6.56	0.00	0.73	0.00	0.05	0.22	-	-	-	-	-
ELAC1	18	50967990	431	top1	0.05	6.20E-05	3.81	1.41E-04	2.17E-02	rs12277	4.00	rs645088	4.49	0.55	0.12	0.02	0.00	0.31	-	-	-	-	-
EMC8	16	85771757	643	enet	0.09	4.10E-08	3.89	1.01E-04	1.96E-02	rs16940641	-4.64	rs2075531	4.59	0.46	0.32	0.12	0.09	0.01	-	-	-	-	-
EXOSC5	19	41386370	379	top1	0.09	4.80E-08	-3.97	7.07E-05	1.54E-02	rs2241714	-6.11	rs2231940	5.62	0.00	0.00	0.02	0.98	0.01	-	-	-	-	-
**FNIP2**	**4**	**158769025**	**265**	**enet**	**0.09**	**5.70E-08**	**-3.73**	**1.94E-04**	**2.64E-02**	**rs13152601**	**4.47**	**rs4331733**	**-5.09**	**0.00**	**0.10**	**0.00**	**0.02**	**0.88**	**Yes**	**Novel**	**-**	**-**	**-**
GDPGP1	15	90233807	401	enet	0.51	9.70E-50	-3.56	3.73E-04	4.23E-02	rs11852389	-4.62	rs1533327	12.35	0.00	0.60	0.00	0.21	0.19	-	-	-	-	-
GNA12	7	2728104	563	enet	0.12	2.00E-10	4.21	2.54E-05	6.54E-03	rs798502	-5.02	rs1636264	-6.04	0.00	0.01	0.00	0.01	0.98	Yes	Reported	36539618	TWAS	-
GNL3	3	52681155	386	top1	0.22	2.40E-18	-3.87	1.08E-04	1.96E-02	rs2001732	-5.36	rs35911561	8.29	0.00	0.09	0.00	0.58	0.33	-	-	-	-	-
GPBAR1	2	218259495	445	lasso	0.10	8.40E-09	-3.88	1.03E-04	1.96E-02	rs2168704	-5.04	rs736731	4.95	0.00	0.00	0.00	0.03	0.96	Yes	Reported	36539618	TWAS	-
HAS3	16	69105652	309	top1	0.04	3.40E-04	3.60	3.18E-04	3.80E-02	rs8056538	-5.00	rs12599393	-4.36	0.18	0.02	0.68	0.08	0.03	-	-	-	-	-
HIP1	7	75533297	265	top1	0.12	6.00E-10	4.39	1.12E-05	3.65E-03	rs6964826	-4.46	rs6964389	-6.40	0.00	0.03	0.00	0.01	0.96	Yes	Reported	12163454	Candidate gene	TRUE
HLA-F	6	29722774	40	lasso	0.55	2.60E-55	-4.64	3.49E-06	1.48E-03	rs9257940	4.72	rs2523405	11.83	0.00	0.24	0.00	0.09	0.68	-	-	-	-	-
HP	16	72054504	358	top1	0.02	1.30E-02	3.74	1.85E-04	2.59E-02	rs1549292	-4.19	rs4788821	-3.58	0.88	0.01	0.10	0.00	0.01	-	-	-	-	-

INTS11	1	1311584	255	enet	0.18	6.90E-15	3.64	2.74E-04	3.39E-02	rs11260584	3.35	rs12103	-6.24	0.00	0.86	0.00	0.00	0.14	-	-	-	-	-
IQGAP1	15	90388241	400	enet	0.25	9.50E-21	-3.49	4.88E-04	4.78E-02	rs11852389	-4.62	rs1505936	-7.04	0.00	0.72	0.00	0.24	0.05	-	-	-	-	-
ITGB3	17	47253826	328	lasso	0.29	1.90E-24	-3.64	2.74E-04	3.39E-02	rs11871251	-3.62	rs2317385	9.42	0.00	0.46	0.00	0.00	0.54	-	-	-	-	-
KCTD20	6	36442766	509	enet	0.03	3.20E-03	-3.66	2.51E-04	3.23E-02	rs1321313	6.83	rs2071809	-3.65	0.00	0.00	0.81	0.04	0.15	-	-	-	-	-
KIAA1671	22	24952715	443	top1	0.06	1.70E-05	3.82	1.33E-04	2.13E-02	rs4822502	3.98	rs1008932	4.64	0.37	0.16	0.01	0.01	0.45	-	-	-	-	-
L3MBTL3	6	130013698	561	top1	0.54	1.40E-52	3.55	3.81E-04	4.23E-02	rs6569648	-3.55	rs6569648	-12.79	0.00	0.48	0.00	0.00	0.52	-	-	-	-	-
LAMA5	20	62307954	511	enet	0.13	8.00E-11	-5.28	1.30E-07	9.79E-05	rs1570027	-11.31	rs6121540	5.10	0.00	0.00	0.03	0.49	0.49	-	-	-	-	-
LAMC1	1	183023419	439	enet	0.29	5.10E-25	5.73	9.87E-09	1.07E-05	rs10752881	6.97	rs6424888	9.48	0.00	0.00	0.00	0.14	0.86	Yes	Reported	36539618	TWAS	-
LAYN	11	111540279	390	enet	0.11	3.00E-09	3.64	2.70E-04	3.39E-02	rs6589218	8.53	rs614725	-4.48	0.00	0.00	0.56	0.39	0.06	-	-	-	-	-
LIMA1	12	50175787	312	lasso	0.17	3.30E-14	3.99	6.53E-05	1.49E-02	rs7315690	6.96	rs17124559	7.10	0.00	0.00	0.00	1.00	0.00	-	-	-	-	-
LMOD1	1	201896455	478	enet	0.31	9.50E-27	4.55	5.24E-06	1.97E-03	rs2820313	5.28	rs2820315	9.46	0.00	0.01	0.00	0.05	0.95	Yes	Reported	36539618	TWAS	-
LRCH1	13	46553167	560	enet	0.03	1.20E-03	3.83	1.26E-04	2.13E-02	rs4942568	-3.92	rs2146890	-3.90	0.84	0.13	0.01	0.00	0.02	-	-	-	-	-
MAMSTR	19	48712724	441	enet	0.05	2.50E-05	3.51	4.49E-04	4.72E-02	rs601338	4.38	rs516246	5.04	0.00	0.08	0.00	0.01	0.91	Yes	Novel	-	-	-
MAPK13	6	36127808	372	lasso	0.43	7.70E-39	3.61	3.03E-04	3.66E-02	rs28675670	-5.21	rs1059227	11.18	0.00	0.10	0.00	0.17	0.73	-	-	-	-	-
**METRNL**	**17**	**83079608**	**243**	**enet**	**0.14**	**1.30E-11**	**-4.65**	**3.29E-06**	**1.46E-03**	**rs7502442**	**-6.56**	**rs4986109**	**5.75**	**0.00**	**0.00**	**0.00**	**0.02**	**0.98**	**Yes**	**Novel**	**-**	**-**	**-**
METTL7A	12	50923471	356	enet	0.04	1.90E-04	-4.30	1.73E-05	4.98E-03	rs10783387	7.06	rs1316607	-3.64	0.00	0.00	0.27	0.02	0.71	-	-	-	-	-
MYC	8	127735433	622	enet	0.07	7.80E-07	-8.68	4.04E-18	3.95E-14	rs6983267	16.67	rs12675207	-4.26	0.00	0.00	0.31	0.09	0.60	-	-	-	-	-
MYL2	12	110910818	237	lasso	0.03	2.30E-03	5.95	2.60E-09	4.24E-06	rs11065822	-6.25	rs4378452	-4.20	0.00	0.00	0.14	0.03	0.83	Yes	Novel	-	-	-
**NAA38**	**17**	**7856684**	**506**	**lasso**	**0.14**	**8.50E-12**	**-3.80**	**1.44E-04**	**2.17E-02**	**rs11078711**	**4.14**	**rs11078711**	**-6.58**	**0.00**	**0.11**	**0.00**	**0.00**	**0.89**	**Yes**	**Novel**	**-**	**-**	**-**
NBPF9	1	149052185	3	top1	0.38	4.50E-33	-3.48	5.01E-04	4.85E-02	rs12124527	-3.48	rs12124527	10.76	0.00	0.54	0.00	0.00	0.46	-	-	-	-	-
NISCH	3	52455117	363	top1	0.06	1.30E-05	3.51	4.44E-04	4.72E-02	rs2001732	-5.36	rs758800	-4.98	0.04	0.09	0.26	0.53	0.09	-	-	-	-	-
OTUD7B	1	149937811	154	top1	0.02	4.10E-03	-3.62	2.92E-04	3.57E-02	rs7531664	3.70	rs11205037	-3.46	0.99	0.01	0.01	0.00	0.01	-	-	-	-	-

PAFAH1B2	11	117144283	544	enet	0.01	3.50E-02	-3.98	6.92E-05	1.54E-02	rs1351452	4.04	rs6589598	-3.91	0.77	0.05	0.06	0.00	0.12	-	-	-	-	-
PCBD2	5	134905119	353	enet	0.00	3.20E-01	4.07	4.75E-05	1.16E-02	rs641455	9.70	rs319597	3.62	0.00	0.00	0.47	0.02	0.51	-	-	-	-	-
PEDS1	20	50118253	436	enet	0.00	3.00E-01	4.79	1.64E-06	8.02E-04	rs6063516	-7.15	rs6020374	-3.65	0.00	0.00	0.93	0.05	0.02	-	-	-	-	-
PIAS1	15	68054308	449	lasso	0.29	1.60E-24	4.35	1.37E-05	4.19E-03	rs11633495	5.29	rs16951524	9.20	0.00	0.06	0.00	0.86	0.08	-	-	-	-	-
PLIN1	15	89664366	385	lasso	0.01	2.10E-02	4.52	6.28E-06	2.19E-03	rs3803533	-3.79	rs2882677	-3.73	0.79	0.17	0.01	0.00	0.03	-	-	-	-	-
PNKD	2	218269650	435	top1	0.19	1.50E-15	-4.80	1.56E-06	8.02E-04	rs2168704	-5.02	rs897877	7.80	0.00	0.01	0.00	0.04	0.96	Yes	Reported	36539618	TWAS	-
PPME1	11	74171266	407	enet	0.02	1.80E-02	3.84	1.21E-04	2.11E-02	rs3824999	8.07	rs7944514	-3.84	0.00	0.00	0.32	0.04	0.64	-	-	-	-	-
PPP1CC	12	110719679	218	enet	0.10	6.40E-09	-4.54	5.56E-06	2.02E-03	rs11065822	-6.25	rs12311093	-5.69	0.00	0.00	0.00	0.72	0.28	-	-	-	-	-
PPP1R11	6	30066708	39	top1	0.03	1.20E-03	3.81	1.38E-04	2.17E-02	rs9257940	4.72	rs2523405	-3.67	0.72	0.01	0.26	0.00	0.02	-	-	-	-	-
PPP1R12A	12	79773562	288	top1	0.02	1.30E-02	3.80	1.42E-04	2.17E-02	rs7297309	4.07	rs10778677	4.02	0.84	0.03	0.07	0.00	0.07	-	-	-	-	-
PREX1	20	48624251	463	lasso	0.16	1.90E-13	7.00	2.52E-12	8.22E-09	rs6066825	-8.80	rs12106159	-7.04	0.00	0.00	0.00	0.02	0.98	Yes	Reported	36539618	TWAS	-
PTPA	9	129110949	380	enet	0.60	5.40E-62	3.90	9.72E-05	1.96E-02	rs2541164	3.81	rs9697030	12.90	0.00	0.32	0.00	0.02	0.66	-	-	-	-	-
RAD52	12	911735	405	lasso	0.32	2.70E-27	3.70	2.18E-04	2.88E-02	rs12312603	-4.47	rs12816367	-9.41	0.00	0.73	0.00	0.07	0.20	-	-	-	-	-
RCCD1	15	90954880	487	lasso	0.54	1.30E-52	-3.83	1.26E-04	2.13E-02	rs11852389	-4.62	rs2290202	12.61	0.00	0.31	0.00	0.09	0.60	-	-	-	-	-
RHOV	15	40872213	285	top1	0.06	1.00E-05	-3.50	4.69E-04	4.75E-02	rs11855560	3.94	rs8042729	4.75	0.43	0.29	0.01	0.01	0.26	-	-	-	-	-
RHPN2	19	32978591	439	lasso	0.40	3.60E-35	3.82	1.33E-04	2.13E-02	rs10411210	-7.57	rs2287679	-10.79	0.00	0.00	0.00	1.00	0.00	-	-	-	-	-
RMI1	9	83980797	528	lasso	0.20	6.30E-17	4.14	3.45E-05	8.66E-03	rs17080575	4.64	rs4877815	-6.87	0.00	0.37	0.00	0.11	0.52	-	-	-	-	-
**RUFY2**	**10**	**68341106**	**392**	**top1**	**0.28**	**2.50E-23**	**4.27**	**1.99E-05**	**5.56E-03**	**rs10998195**	**-4.51**	**rs10998074**	**-9.27**	**0.00**	**0.05**	**0.00**	**0.02**	**0.93**	**Yes**	**Novel**	**-**	**-**	**-**
SH2B3	12	111405922	239	top1	0.07	3.90E-06	5.80	6.59E-09	8.06E-06	rs3184504	-6.26	rs12818548	4.80	0.00	0.00	0.12	0.11	0.77	Yes	Reported	36539618	TWAS	-
SMARCAL1	2	216412382	515	lasso	0.42	2.40E-38	3.92	8.68E-05	1.85E-02	rs3755141	3.78	rs3755141	10.59	0.00	0.29	0.00	0.00	0.71	-	-	-	-	-
SMPD3	16	68358326	315	enet	0.04	2.20E-04	4.40	1.06E-05	3.58E-03	rs8056538	-5.00	rs1862748	-4.48	0.01	0.02	0.02	0.08	0.87	Yes	Novel	-	-	-
SPATA2	20	49903390	466	lasso	0.03	2.30E-03	3.50	4.67E-04	4.75E-02	rs926611	6.45	rs6125931	-4.26	0.00	0.00	0.68	0.31	0.01	-	-	-	-	-
ST6GALNA	9	127907885	344	enet	0.06	4.50E-06	4.98	6.48E-07	3.73E-04	rs1537034	-3.68	rs2502817	-4.15	0.89	0.10	0.01	0.00	0.00	-	-	-	-	-

C4																							
STAB1	3	52495337	370	enet	0.03	1.50E-03	-4.30	1.70E-05	4.98E-03	rs2001732	-5.36	rs7614424	4.30	0.04	0.03	0.23	0.18	0.53	-	-	-	-	-
TANC1	2	158968639	522	enet	0.03	8.30E-04	3.50	4.71E-04	4.75E-02	rs842064	-5.36	rs2711053	4.47	0.01	0.01	0.27	0.28	0.43	-	-	-	-	-
TGFBR1	9	99104037	453	lasso	0.05	1.10E-04	3.70	2.12E-04	2.84E-02	rs17710605	-3.85	rs7031588	-4.03	0.78	0.18	0.01	0.00	0.03	-	-	-	-	-
TMBIM1	2	218274196	434	lasso	0.56	6.80E-57	-4.88	1.05E-06	5.71E-04	rs2168704	-4.97	rs3817266	13.05	0.00	0.01	0.00	0.09	0.90	Yes	Reported	36539618	TWAS	-
TOR4A	9	137277725	361	lasso	0.13	2.70E-11	3.76	1.67E-04	2.44E-02	rs28631372	-3.38	rs28455773	-6.01	0.00	0.87	0.00	0.01	0.13	-	-	-	-	-
TRIM4	7	99876957	268	lasso	0.72	1.50E-86	-3.82	1.33E-04	2.13E-02	rs2527927	-4.54	rs2572019	14.84	0.00	0.14	0.00	0.07	0.80	Yes	Reported	36539618	TWAS	-
TRMT6	20	5937227	600	top1	0.06	7.00E-06	5.08	3.72E-07	2.43E-04	rs961253	8.10	rs6085512	-4.97	0.00	0.00	0.34	0.66	0.00	-	-	-	-	-
TTLL13P	15	90249529	403	enet	0.02	1.90E-02	-5.40	6.62E-08	5.40E-05	rs11852389	-4.62	rs2882677	3.60	0.67	0.07	0.23	0.02	0.01	-	-	-	-	-
TUBA1A	12	49184685	303	top1	0.05	3.90E-05	3.55	3.85E-04	4.23E-02	rs1274726	3.55	rs1274726	4.29	0.84	0.08	0.00	0.00	0.08	-	-	-	-	-
UACA	15	70654553	376	enet	0.09	8.10E-08	-3.68	2.38E-04	3.11E-02	rs1398199	3.40	rs2036951	4.35	0.35	0.61	0.00	0.00	0.03	-	-	-	-	-
UBAP2	9	33921692	313	enet	0.08	3.00E-07	3.53	4.19E-04	4.56E-02	rs11557154	5.67	rs10441723	-5.06	0.00	0.04	0.06	0.77	0.12	-	-	-	-	-
UBE2V1	20	50081123	438	enet	0.12	4.90E-10	-4.05	5.06E-05	1.21E-02	rs6063516	-7.15	rs1000595	5.97	0.00	0.00	0.00	1.00	0.00	-	-	-	-	-
VPS18	15	40894449	276	enet	0.06	1.70E-05	-3.85	1.16E-04	2.06E-02	rs11855560	3.94	rs7179231	3.54	0.90	0.06	0.03	0.00	0.00	-	-	-	-	-
XYLB	3	38346759	438	top1	0.07	1.30E-06	4.38	1.19E-05	3.76E-03	rs843890	4.66	rs818851	5.32	0.00	0.03	0.00	0.03	0.93	Yes	Novel	-	-	-
YWHAH	22	31944521	463	enet	0.13	1.10E-10	4.03	5.52E-05	1.29E-02	rs5998196	3.87	rs4820059	6.99	0.00	0.29	0.00	0.01	0.70	-	-	-	-	-
ZNF408	11	46701029	236	enet	0.08	4.60E-07	-4.74	2.17E-06	1.01E-03	rs2306029	4.10	rs2306036	4.98	0.02	0.45	0.00	0.05	0.48	-	-	-	-	-
ZNF473	19	50025713	337	enet	0.09	1.20E-07	-3.74	1.85E-04	2.59E-02	rs2445828	-5.83	rs1274604	5.00	0.00	0.00	0.02	0.10	0.89	Yes	Novel	-	-	-

* If the direcction of the TWAS association is consistent with that reported in previous studies, it is marked as TRUE. "-" represents that the corresponding information is missing in the previous studies.

Bold indicates that the genes were also identified in single-tissue TWAS analysis.

TSS: transcription start site; FDR: false discovery rate.

Supplementary Table 6. Significant pathways using genes identified by single-tissue and multiple-tissue TWAS.

Database	Pathway	Overlap	P-value	Adjusted P-value	Odds Ratio	Combined Score	Genes
KEGG	Regulation of actin cytoskeleton	11/218	1.02E-04	8.58E-03	4.33	39.76	PPP1CC;PPP1R12A;ARPC2;MYL2;ITGB3;LIMK2;GNA12;PDGFB;PIP4K2A;IQGAP1;F2
KEGG	Allograft rejection	5/38	1.07E-04	8.58E-03	12.14	111.02	HLA-DRB5;HLA-DPB1;HLA-DRA;HLA-F;HLA-DQA1
KEGG	Phagosome	9/152	1.32E-04	8.58E-03	5.10	45.54	HLA-DRB5;TUBA1A;NCF2;ITGB3;HLA-DPB1;HLA-DRA;CALR;HLA-F;HLA-DQA1
KEGG	Graft-versus-host disease	5/42	1.74E-04	8.58E-03	10.83	93.73	HLA-DRB5;HLA-DPB1;HLA-DRA;HLA-F;HLA-DQA1
KEGG	Type I diabetes mellitus	5/43	1.95E-04	8.58E-03	10.54	90.06	HLA-DRB5;HLA-DPB1;HLA-DRA;HLA-F;HLA-DQA1
KEGG	Th17 cell differentiation	7/107	4.05E-04	1.07E-02	5.64	44.03	HLA-DRB5;HLA-DPB1;HLA-DRA;STAT6;HLA-DQA1;TGFBR1;MAPK13
KEGG	Leishmaniasis	6/77	4.15E-04	1.07E-02	6.79	52.86	HLA-DRB5;NCF2;HLA-DPB1;HLA-DRA;HLA-DQA1;MAPK13
KEGG	Antigen processing and presentation	6/78	4.44E-04	1.07E-02	6.69	51.66	HLA-DRB5;HLA-DPB1;HLA-DRA;CALR;HLA-F;HLA-DQA1
KEGG	Autoimmune thyroid disease	5/53	5.25E-04	1.07E-02	8.34	63.00	HLA-DRB5;HLA-DPB1;HLA-DRA;HLA-F;HLA-DQA1
KEGG	Toxoplasmosis	7/112	5.34E-04	1.07E-02	5.37	40.45	LAMA5;HLA-DRB5;HLA-DPB1;HLA-DRA;LAMC1;HLA-DQA1;MAPK13
KEGG	Cell adhesion molecules	8/148	5.73E-04	1.07E-02	4.61	34.42	HLA-DRB5;CDH1;HLA-DPB1;ICAM3;CNTN2;HLA-DRA;HLA-F;HLA-DQA1
KEGG	Asthma	4/31	5.84E-04	1.07E-02	11.83	88.08	HLA-DRB5;HLA-DPB1;HLA-DRA;HLA-DQA1
KEGG	Viral myocarditis	5/60	9.31E-04	1.56E-02	7.28	50.79	HLA-DRB5;HLA-DPB1;HLA-DRA;HLA-F;HLA-DQA1
KEGG	Epstein-Barr virus infection	9/202	1.06E-03	1.56E-02	3.77	25.82	HLA-DRB5;MYC;ADRM1;HLA-DPB1;HLA-DRA;CALR;HLA-F;HLA-DQA1;MAPK13
KEGG	Th1 and Th2 cell differentiation	6/92	1.07E-03	1.56E-02	5.60	38.32	HLA-DRB5;HLA-DPB1;HLA-DRA;STAT6;HLA-DQA1;

							MAPK13
KEGG	Inflammatory bowel disease	5/65	1.34E-03	1.84E-02	6.67	44.13	HLA-DRB5;HLA-DPB1;HLA-DRA;STAT6;HLA-DQA1
KEGG	Human T-cell leukemia virus 1 infection	9/219	1.85E-03	2.39E-02	3.46	21.78	HLA-DRB5;CRTC3;MYC;HLA-DPB1;HLA-DRA;CALR;HLA-F;HLA-DQA1;TGFBR1
KEGG	Intestinal immune network for IgA production	4/48	3.06E-03	3.74E-02	7.25	42.00	HLA-DRB5;HLA-DPB1;HLA-DRA;HLA-DQA1
GO	Lumenal Side Of Endoplasmic Reticulum Membrane (GO:0098553)	6/27	8.73E-07	1.47E-04	23.01	320.96	HLA-DRB5;SPPL2A;HLA-DRA;CALR;HLA-F;HLA-DQA1
GO	Bounding Membrane Of Organelle (GO:0098588)	25/819	4.01E-05	2.68E-03	2.64	26.73	MCFD2;LRP1;USO1;SPPL2A;PDGFB;IQGAP1;PCSK7;FUT2;CORO1C;CXCR1;MAN2A2;STAB1;MGAT3;HLA-DQA1;WNT4;VPS18;ARFGEF2;HLA-DRB5;SLC30A8;HLA-F;FIG4;TMBIM1;HLA-DRA;CALR;ST6GALNAC4
GO	ER To Golgi Transport Vesicle Membrane (GO:0012507)	6/55	6.38E-05	2.68E-03	9.85	95.11	MCFD2;HLA-DRB5;USO1;HLA-DRA;HLA-F;HLA-DQA1
GO	Coated Vesicle Membrane (GO:0030662)	6/55	6.38E-05	2.68E-03	9.85	95.11	MCFD2;HLA-DRB5;USO1;HLA-DRA;HLA-F;HLA-DQA1
GO	Transport Vesicle Membrane (GO:0030658)	6/60	1.05E-04	3.43E-03	8.93	81.87	MCFD2;HLA-DRB5;USO1;HLA-DRA;HLA-F;HLA-DQA1
GO	MHC Protein Complex (GO:0042611)	4/21	1.23E-04	3.43E-03	18.80	169.31	HLA-DRB5;HLA-DRA;HLA-F;HLA-DQA1
GO	Cytoskeleton (GO:0005856)	19/599	2.14E-04	5.15E-03	2.71	22.86	HIP1;PPP1R12A;CCDC146;CKAP2;IQGAP1;ADD3;CKAP5;CORO1C;NISCH;AKAP13;LIMA1;ARHGAP21;ACTR1B;TUBA1A;ARPC2;CDH1;MYL2;MYO5C;PKP4
GO	COPII-coated ER To Golgi Transport Vesicle (GO:0030134)	6/76	3.86E-04	7.98E-03	6.88	54.11	MCFD2;HLA-DRB5;USO1;HLA-DRA;HLA-F;HLA-DQA1
GO	Cytoplasmic Vesicle Membrane (GO:0030659)	14/389	4.27E-04	7.98E-03	3.05	23.68	VPS18;HLA-DRB5;SLC30A8;LRP1;SPPL2A;IQGAP1;

							CORO1C;FIG4;CXCR1;STAB1;TMBIM1;HLA-DRA;HLA-DQA1;WNT4
GO	MHC Class II Protein Complex (GO:0042613)	3/15	7.95E-04	1.28E-02	19.90	142.01	HLA-DRB5;HLA-DRA;HLA-DQA1
GO	Endocytic Vesicle (GO:0030139)	9/196	8.55E-04	1.28E-02	3.89	27.48	HLA-DRB5;LRP1;STAB1;HP;HLA-DRA;APOA1;CALR;HLA-DQA1;WNT4
GO	Endocytic Vesicle Membrane (GO:0030666)	8/159	9.16E-04	1.28E-02	4.27	29.89	HLA-DRB5;LRP1;STAB1;HLA-DRA;CALR;HLA-F;HLA-DQA1;WNT4
GO	Golgi Membrane (GO:0000139)	14/427	1.06E-03	1.37E-02	2.77	18.95	ARFGEF2;HLA-DRB5;SLC30A8;USO1;PDGFB;PCSK7;HLA-F;FUT2;FIG4;MAN2A2;MGAT3;HLA-DRA;ST6GALNAC4;HLA-DQA1
GO	Focal Adhesion (GO:0005925)	13/387	1.28E-03	1.53E-02	2.83	18.85	PPP1R12A;LRP1;RPS5;ITGB3;LAYN;FHL3;IQGAP1;CORO1C;LIMA1;PPP1CC;ARPC2;GNA12;CALR
GO	Cell-Substrate Junction (GO:0030055)	13/395	1.53E-03	1.72E-02	2.77	17.95	PPP1R12A;LRP1;RPS5;ITGB3;LAYN;FHL3;IQGAP1;CORO1C;LIMA1;PPP1CC;ARPC2;GNA12;CALR
GO	PTW/PP1 Phosphatase Complex (GO:0072357)	2/6	2.28E-03	2.25E-02	39.65	241.27	PPP1CC;PPP1R12A
GO	Flotillin Complex (GO:0016600)	2/6	2.28E-03	2.25E-02	39.65	241.27	CDH1;CORO1C
GO	Actin Cytoskeleton (GO:0015629)	11/327	2.93E-03	2.74E-02	2.82	16.44	AKAP13;LIMA1;ARHGAP21;PPP1R12A;ARPC2;CDH1;MYL2;MYO5C;LMOD1;IQGAP1;CORO1C
Reactome	Signaling By Rho GTPases R-HSA-194315	22/644	2.25E-05	1.11E-02	2.95	31.62	PPP1R12A;CAVIN1;NCF2;CUL3;LIMK2;IQGAP1;ADD3;MYO9A;CKAP5;UACA;NISCH;DOCK10;PREX1;AKAP13;PPP1CC;ARHGAP21;ARPC2;PKP4;RHOV;B9D2;RHPN2;YWHAH
Reactome	Signaling By Rho GTPases, Miro GTPases And RHOBTB3 R-HSA-9716542	22/660	3.26E-05	1.11E-02	2.88	29.73	PPP1R12A;CAVIN1;NCF2;CUL3;LIMK2;IQGAP1;ADD3;MYO9A;CKAP5;UACA;NISCH;DOCK10;PREX1;AKAP13;PPP1CC;ARHGAP21;ARPC2;PKP4;RHOV;B9D2;RHPN2;YWHAH

Reactome	Signal Transduction R-HSA-162582	51/2465	2.05E-04	3.89E-02	1.83	15.55	VPS29;NCF2;ITGB3;SPPL2A;OTUD7B;LAMC1;SMPD3;DOCK10;PREX1;AKAP13;PPP1CC;MYC;PIP4K2A;SOX4;DACT1;YWHAH;PPP1R12A;AXIN1;GPBAR1;CKAP5;TGFBR1;NISCH;PKP4;PLIN1;RHOV;LAMA5;GTF2A2;PRKAA1;CUL3;PDGFB;IQGAP1;ADD3;UACA;ARHGAP21;CXCR1;GNA12;STAT6;TAC3;RHPN2;AAMP;SH2B3;WNT4;KL;CAVIN1;LIMK2;S100B;MYO9A;MAPK13;ARPC2;BAMBI;B9D2
Reactome	RHOB GTPase Cycle R-HSA-9013026	6/69	2.28E-04	3.89E-02	7.65	64.18	AKAP13;PREX1;ARHGAP21;CAVIN1;RHPN2;MYO9A
MSigDB Hallmark	Myc Targets V2	5/58	7.97E-04	3.35E-02	7.55	53.89	EXOSC5;MYC;WDR43;GNL3;TBRG4

Supplementary Table 7. Co-expression and pathway results for novel CRC susceptibility genes identified by single-tissue and across-tissue TWASs.

Gene	Description	GeneRatio	BgRatio	P-value	Adjusted P-value
ASPDH	Cilium Assembly	14/203	201/10891	2.32E-05	1.78E-02
AXIN1	Constitutive Signaling by AKT1 E17K in Cancer	12/1237	25/10891	5.52E-06	5.36E-03
AXIN1	Mitochondrial translation elongation	25/1237	87/10891	7.61E-06	5.36E-03
AXIN1	Regulation of TP53 Activity	37/1237	160/10891	1.63E-05	6.34E-03
AXIN1	Mitochondrial translation initiation	24/1237	87/10891	2.45E-05	6.34E-03
AXIN1	Mitochondrial translation termination	24/1237	87/10891	2.45E-05	6.34E-03
AXIN1	Mitochondrial translation	25/1237	93/10891	2.70E-05	6.34E-03
AXIN1	Transcriptional Regulation by TP53	66/1237	362/10891	5.86E-05	1.18E-02
C1orf122	Complex I biogenesis	31/2432	57/10891	1.33E-07	1.26E-04
C1orf122	Influenza Viral RNA Transcription and Replication	57/2432	135/10891	1.65E-07	1.26E-04
C1orf122	Processing of Capped Intron-Containing Pre-mRNA	89/2432	246/10891	4.17E-07	2.11E-04
C1orf122	Influenza Infection	62/2432	156/10891	6.39E-07	2.11E-04
C1orf122	Respiratory electron transport, ATP synthesis by chemiosmotic coupling, and heat production by uncoupling proteins.	53/2432	127/10891	6.93E-07	2.11E-04
C1orf122	mRNA Splicing - Minor Pathway	27/2432	50/10891	1.04E-06	2.63E-04
C1orf122	Respiratory electron transport	44/2432	103/10891	2.85E-06	6.21E-04
C1orf122	Late Phase of HIV Life Cycle	53/2432	136/10891	8.04E-06	1.53E-03
C1orf122	SUMOylation of ubiquitinylation proteins	21/2432	38/10891	1.02E-05	1.73E-03
C1orf122	mRNA Splicing	69/2432	193/10891	1.29E-05	1.97E-03
C1orf122	Selenocysteine synthesis	39/2432	93/10891	1.74E-05	2.41E-03
C1orf122	HIV Infection	79/2432	231/10891	2.03E-05	2.58E-03
C1orf122	Viral Messenger RNA Synthesis	22/2432	43/10891	3.19E-05	3.74E-03
C1orf122	Mitochondrial translation	38/2432	93/10891	4.44E-05	4.77E-03
C1orf122	SUMO E3 ligases SUMOylate target proteins	64/2432	182/10891	4.69E-05	4.77E-03
C1orf122	Mitochondrial translation initiation	36/2432	87/10891	5.08E-05	4.84E-03
C1orf122	HIV Life Cycle	54/2432	149/10891	7.16E-05	6.42E-03
C1orf122	SUMOylation of SUMOylation proteins	18/2432	34/10891	9.36E-05	7.93E-03
C1orf122	rRNA processing in the nucleus and cytosol	66/2432	194/10891	1.13E-04	8.51E-03
C1orf122	SUMOylation of DNA damage response and repair proteins	32/2432	77/10891	1.18E-04	8.51E-03
C1orf122	Mitochondrial translation elongation	35/2432	87/10891	1.27E-04	8.51E-03
C1orf122	Mitochondrial translation termination	35/2432	87/10891	1.27E-04	8.51E-03
C1orf122	SUMOylation	64/2432	188/10891	1.40E-04	8.51E-03
C1orf122	Cellular response to starvation	55/2432	156/10891	1.44E-04	8.51E-03
C1orf122	Nucleotide Excision Repair	42/2432	111/10891	1.48E-04	8.51E-03
C1orf122	Formation of a pool of free 40S subunits	39/2432	101/10891	1.53E-04	8.51E-03
C1orf122	mRNA Splicing - Major Pathway	63/2432	185/10891	1.55E-04	8.51E-03

C1orf122	SARS-CoV Infections	114/2432	376/10891	1.56E-04	8.51E-03
C1orf122	Peptide chain elongation	35/2432	89/10891	2.16E-04	1.10E-02
C1orf122	Viral mRNA Translation	35/2432	89/10891	2.16E-04	1.10E-02
C1orf122	Eukaryotic Translation Elongation	36/2432	93/10891	2.55E-04	1.22E-02
C1orf122	Eukaryotic Translation Termination	36/2432	93/10891	2.55E-04	1.22E-02
C1orf122	The citric acid (TCA) cycle and respiratory electron transport	60/2432	178/10891	3.00E-04	1.39E-02
C1orf122	rRNA processing	67/2432	204/10891	3.21E-04	1.42E-02
C1orf122	Selenoamino acid metabolism	43/2432	118/10891	3.27E-04	1.42E-02
C1orf122	Nonsense-Mediated Decay (NMD)	42/2432	115/10891	3.61E-04	1.49E-02
C1orf122	Nonsense Mediated Decay (NMD) enhanced by the Exon Junction Complex (EJC)	42/2432	115/10891	3.61E-04	1.49E-02
C1orf122	Nonsense Mediated Decay (NMD) independent of the Exon Junction Complex (EJC)	36/2432	95/10891	4.13E-04	1.65E-02
C1orf122	TP53 Regulates Transcription of DNA Repair Genes	26/2432	62/10891	4.23E-04	1.65E-02
C1orf122	Host Interactions of HIV factors	46/2432	130/10891	4.43E-04	1.69E-02
C1orf122	Translocation of SLC2A4 (GLUT4) to the plasma membrane	29/2432	72/10891	4.57E-04	1.70E-02
C1orf122	SARS-CoV-1 Infection	23/2432	53/10891	4.99E-04	1.77E-02
C1orf122	Translation of Structural Proteins	15/2432	29/10891	5.00E-04	1.77E-02
C1orf122	Transcription of the HIV genome	28/2432	70/10891	6.49E-04	2.25E-02
C1orf122	Response of EIF2AK4 (GCN2) to amino acid deficiency	37/2432	101/10891	7.37E-04	2.44E-02
C1orf122	RAS processing	14/2432	27/10891	7.45E-04	2.44E-02
C1orf122	Mitotic Anaphase	74/2432	236/10891	7.53E-04	2.44E-02
C1orf122	SUMOylation of DNA replication proteins	20/2432	45/10891	7.90E-04	2.51E-02
C1orf122	RNA Polymerase II Pre-transcription Events	31/2432	81/10891	8.34E-04	2.60E-02
C1orf122	Mitotic Metaphase and Anaphase	74/2432	237/10891	8.60E-04	2.62E-02
C1orf122	Translation	88/2432	291/10891	9.21E-04	2.75E-02
C1orf122	Nuclear Envelope Breakdown	22/2432	52/10891	9.95E-04	2.92E-02
C1orf122	Transcription-Coupled Nucleotide Excision Repair (TC-NER)	30/2432	79/10891	1.16E-03	3.35E-02
C1orf122	Cristae formation	15/2432	31/10891	1.23E-03	3.41E-02
C1orf122	Antigen processing: Ubiquitination & Proteasome degradation	92/2432	309/10891	1.23E-03	3.41E-02
C1orf122	Dual incision in TC-NER	26/2432	66/10891	1.30E-03	3.54E-02
C1orf122	L13a-mediated translational silencing of Ceruloplasmin expression	39/2432	111/10891	1.35E-03	3.56E-02
C1orf122	Major pathway of rRNA processing in the nucleolus and cytosol	59/2432	184/10891	1.37E-03	3.56E-02
C1orf122	Protein folding	35/2432	97/10891	1.38E-03	3.56E-02
C1orf122	SUMOylation of transcription factors	11/2432	20/10891	1.48E-03	3.74E-02

C1orf122	SUMOylation of RNA binding proteins	20/2432	47/10891	1.53E-03	3.74E-02
C1orf122	SARS-CoV-2 Infection	86/2432	288/10891	1.59E-03	3.74E-02
C1orf122	Eukaryotic Translation Initiation	41/2432	119/10891	1.59E-03	3.74E-02
C1orf122	Cap-dependent Translation Initiation	41/2432	119/10891	1.59E-03	3.74E-02
C1orf122	GTP hydrolysis and joining of the 60S ribosomal subunit	39/2432	112/10891	1.64E-03	3.74E-02
C1orf122	Translation of Structural Proteins	23/2432	57/10891	1.67E-03	3.74E-02
C1orf122	Late SARS-CoV-2 Infection Events	23/2432	57/10891	1.67E-03	3.74E-02
C1orf122	Global Genome Nucleotide Excision Repair (GG-NER)	31/2432	84/10891	1.67E-03	3.74E-02
C1orf122	Formation of TC-NER Pre-Incision Complex	22/2432	54/10891	1.80E-03	3.93E-02
C1orf122	mRNA decay by 5' to 3' exoribonuclease	9/2432	15/10891	1.80E-03	3.93E-02
C1orf122	Mitotic Spindle Checkpoint	39/2432	113/10891	1.97E-03	4.24E-02
C1orf122	Formation of RNA Pol II elongation complex	23/2432	58/10891	2.19E-03	4.57E-02
C1orf122	RNA Polymerase II Transcription Elongation	23/2432	58/10891	2.19E-03	4.57E-02
C1orf122	Formation of HIV elongation complex in the absence of HIV Tat	19/2432	45/10891	2.22E-03	4.57E-02
C1orf122	trans-Golgi Network Vesicle Budding	27/2432	72/10891	2.48E-03	5.00E-02
C1orf122	Nef-mediates down modulation of cell surface receptors by recruiting them to clathrin adapters	11/2432	21/10891	2.49E-03	5.00E-02
CRTC3	Mitotic G2-G2/M phases	119/3917	198/10891	2.83E-12	2.16E-09
CRTC3	G2/M Transition	118/3917	196/10891	2.98E-12	2.16E-09
CRTC3	KEAP1-NFE2L2 pathway	71/3917	102/10891	4.18E-12	2.16E-09
CRTC3	PTEN Regulation	90/3917	140/10891	7.00E-12	2.71E-09
CRTC3	Transcriptional regulation by RUNX3	66/3917	96/10891	5.70E-11	1.77E-08
CRTC3	RHO GTPase cycle	227/3917	449/10891	7.64E-11	1.97E-08
CRTC3	Regulation of Apoptosis	42/3917	53/10891	1.32E-10	2.35E-08
CRTC3	SCF(Skp2)-mediated degradation of p27/p21	46/3917	60/10891	1.34E-10	2.35E-08
CRTC3	Regulation of PTEN stability and activity	51/3917	69/10891	1.37E-10	2.35E-08
CRTC3	Nuclear events mediated by NFE2L2	56/3917	79/10891	2.56E-10	3.97E-08
CRTC3	Mitotic Anaphase	131/3917	236/10891	4.96E-10	6.99E-08
CRTC3	Fc epsilon receptor (FCERI) signaling	83/3917	134/10891	7.08E-10	8.32E-08
CRTC3	Mitotic Metaphase and Anaphase	131/3917	237/10891	7.20E-10	8.32E-08
CRTC3	Dectin-1 mediated noncanonical NF-kB signaling	45/3917	60/10891	7.52E-10	8.32E-08
CRTC3	Downstream TCR signaling	65/3917	98/10891	8.46E-10	8.74E-08
CRTC3	The role of GTSE1 in G2/M progression after G2 checkpoint	54/3917	77/10891	1.01E-09	9.75E-08
CRTC3	Degradation of AXIN	42/3917	55/10891	1.07E-09	9.75E-08
CRTC3	Regulation of activated PAK-2p34 by proteasome mediated degradation	39/3917	50/10891	1.43E-09	1.19E-07
CRTC3	Intracellular signaling by second messengers	162/3917	309/10891	1.52E-09	1.19E-07
CRTC3	NIK-->noncanonical NF-kB signaling	44/3917	59/10891	1.58E-09	1.19E-07
CRTC3	Ubiquitin Mediated Degradation of	40/3917	52/10891	1.84E-09	1.19E-07

	Phosphorylated Cdc25A				
CRTC3	p53-Independent DNA Damage Response	40/3917	52/10891	1.84E-09	1.19E-07
CRTC3	p53-Independent G1/S DNA damage checkpoint	40/3917	52/10891	1.84E-09	1.19E-07
CRTC3	GSK3B and BTRC:CUL1-mediated-degradation of NFE2L2	40/3917	52/10891	1.84E-09	1.19E-07
CRTC3	Host Interactions of HIV factors	80/3917	130/10891	2.23E-09	1.32E-07
CRTC3	FBXL7 down-regulates AURKA during mitotic entry and in early mitosis	41/3917	54/10891	2.29E-09	1.32E-07
CRTC3	Negative regulation of NOTCH4 signaling	41/3917	54/10891	2.29E-09	1.32E-07
CRTC3	Cross-presentation of soluble exogenous antigens (endosomes)	38/3917	49/10891	3.14E-09	1.74E-07
CRTC3	Activation of APC/C and APC/C:Cdc20 mediated degradation of mitotic proteins	53/3917	77/10891	4.18E-09	2.23E-07
CRTC3	Regulation of APC/C activators between G1/S and early anaphase	55/3917	81/10891	4.62E-09	2.39E-07
CRTC3	MAPK6/MAPK4 signaling	59/3917	89/10891	5.23E-09	2.55E-07
CRTC3	ABC-family proteins mediated transport	66/3917	103/10891	5.40E-09	2.55E-07
CRTC3	CLEC7A (Dectin-1) signaling	65/3917	101/10891	5.43E-09	2.55E-07
CRTC3	SCF-beta-TrCP mediated degradation of Emi1	41/3917	55/10891	5.88E-09	2.68E-07
CRTC3	Stabilization of p53	42/3917	57/10891	6.92E-09	3.06E-07
CRTC3	APC:Cdc20 mediated degradation of cell cycle proteins prior to satisfation of the cell cycle checkpoint	51/3917	74/10891	7.66E-09	3.27E-07
CRTC3	TCR signaling	74/3917	120/10891	7.81E-09	3.27E-07
CRTC3	APC/C:Cdc20 mediated degradation of mitotic proteins	52/3917	76/10891	8.10E-09	3.30E-07
CRTC3	Defective CFTR causes cystic fibrosis	44/3917	61/10891	9.07E-09	3.50E-07
CRTC3	FCERI mediated NF-kB activation	55/3917	82/10891	9.15E-09	3.50E-07
CRTC3	APC/C-mediated degradation of cell cycle proteins	58/3917	88/10891	9.77E-09	3.50E-07
CRTC3	Regulation of mitotic cell cycle	58/3917	88/10891	9.77E-09	3.50E-07
CRTC3	HIV Infection	125/3917	231/10891	1.00E-08	3.50E-07
CRTC3	Vpu mediated degradation of CD4	39/3917	52/10891	1.04E-08	3.50E-07
CRTC3	Autodegradation of the E3 ubiquitin ligase COP1	39/3917	52/10891	1.04E-08	3.50E-07
CRTC3	Ubiquitin-dependent degradation of Cyclin D	39/3917	52/10891	1.04E-08	3.50E-07
CRTC3	Activation of NF-kappaB in B cells	47/3917	67/10891	1.22E-08	3.98E-07
CRTC3	Vif-mediated degradation of APOBEC3G	40/3917	54/10891	1.23E-08	3.98E-07
CRTC3	Cdc20:Phospho-APC/C mediated degradation of Cyclin A	50/3917	73/10891	1.49E-08	4.70E-07
CRTC3	Hedgehog 'on' state	56/3917	85/10891	1.79E-08	5.48E-07
CRTC3	Degradation of GLI1 by the proteasome	43/3917	60/10891	1.84E-08	5.48E-07
CRTC3	GLI3 is processed to GLI3R by the proteasome	43/3917	60/10891	1.84E-08	5.48E-07
CRTC3	PIP3 activates AKT signaling	140/3917	267/10891	1.96E-08	5.73E-07

CRTC3	Autodegradation of Cdh1 by Cdh1:APC/C	45/3917	64/10891	2.23E-08	6.38E-07
CRTC3	Separation of Sister Chromatids	106/3917	191/10891	2.26E-08	6.38E-07
CRTC3	Oxygen-dependent proline hydroxylation of Hypoxia-inducible Factor Alpha	46/3917	66/10891	2.41E-08	6.43E-07
CRTC3	p53-Dependent G1 DNA Damage Response	46/3917	66/10891	2.41E-08	6.43E-07
CRTC3	p53-Dependent G1/S DNA damage checkpoint	46/3917	66/10891	2.41E-08	6.43E-07
CRTC3	APC/C:Cdc20 mediated degradation of Securin	47/3917	68/10891	2.57E-08	6.65E-07
CRTC3	G1/S DNA Damage Checkpoints	47/3917	68/10891	2.57E-08	6.65E-07
CRTC3	Signaling by the B Cell Receptor (BCR)	69/3917	112/10891	2.63E-08	6.69E-07
CRTC3	M Phase	204/3917	418/10891	2.86E-08	7.14E-07
CRTC3	Regulation of RUNX3 expression and activity	40/3917	55/10891	2.95E-08	7.26E-07
CRTC3	Beta-catenin independent WNT signaling	85/3917	146/10891	3.02E-08	7.30E-07
CRTC3	Regulation of mRNA stability by proteins that bind AU-rich elements	57/3917	88/10891	3.35E-08	8.00E-07
CRTC3	Programmed Cell Death	114/3917	210/10891	3.52E-08	8.27E-07
CRTC3	Hh mutants abrogate ligand secretion	42/3917	59/10891	3.71E-08	8.58E-07
CRTC3	Interleukin-1 signaling	70/3917	115/10891	4.22E-08	9.63E-07
CRTC3	CDK-mediated phosphorylation and removal of Cdc6	49/3917	73/10891	5.68E-08	1.28E-06
CRTC3	AUF1 (hnRNP D0) binds and destabilizes mRNA	40/3917	56/10891	6.74E-08	1.47E-06
CRTC3	Hh mutants are degraded by ERAD	40/3917	56/10891	6.74E-08	1.47E-06
CRTC3	Degradation of GLI2 by the proteasome	42/3917	60/10891	8.06E-08	1.74E-06
CRTC3	TNFR2 non-canonical NF-kB pathway	63/3917	102/10891	9.21E-08	1.96E-06
CRTC3	Diseases of signal transduction by growth factor receptors and second messengers	208/3917	433/10891	9.92E-08	2.08E-06
CRTC3	Signaling by Hedgehog	85/3917	149/10891	1.07E-07	2.21E-06
CRTC3	Regulation of ornithine decarboxylase (ODC)	37/3917	51/10891	1.09E-07	2.21E-06
CRTC3	APC/C:Cdh1 mediated degradation of Cdc20 and other APC/C:Cdh1 targeted proteins in late mitosis/early G1	49/3917	74/10891	1.10E-07	2.21E-06
CRTC3	ABC transporter disorders	51/3917	78/10891	1.12E-07	2.23E-06
CRTC3	Hedgehog 'off' state	68/3917	113/10891	1.24E-07	2.43E-06
CRTC3	Processing of Capped Intron-Containing Pre-mRNA	128/3917	246/10891	1.41E-07	2.72E-06
CRTC3	Degradation of DVL	40/3917	57/10891	1.48E-07	2.82E-06
CRTC3	Metabolism of polyamines	41/3917	59/10891	1.59E-07	3.00E-06
CRTC3	Hedgehog ligand biogenesis	44/3917	65/10891	1.86E-07	3.47E-06
CRTC3	Cyclin A:Cdk2-associated events at S phase entry	54/3917	85/10891	2.00E-07	3.62E-06
CRTC3	Orc1 removal from chromatin	47/3917	71/10891	2.02E-07	3.62E-06
CRTC3	Degradation of beta-catenin by the destruction complex	53/3917	83/10891	2.03E-07	3.62E-06
CRTC3	Cyclin E associated events during G1/S transition	53/3917	83/10891	2.03E-07	3.62E-06
CRTC3	Cellular response to hypoxia	49/3917	75/10891	2.06E-07	3.63E-06

CRTC3	Interleukin-1 family signaling	86/3917	153/10891	2.23E-07	3.88E-06
CRTC3	Apoptosis	98/3917	180/10891	2.64E-07	4.55E-06
CRTC3	Switching of origins to a post-replicative state	57/3917	92/10891	3.22E-07	5.49E-06
CRTC3	Asymmetric localization of PCP proteins	43/3917	64/10891	3.54E-07	5.97E-06
CRTC3	Regulation of RAS by GAPs	45/3917	68/10891	3.71E-07	6.19E-06
CRTC3	Downstream signaling events of B Cell Receptor (BCR)	52/3917	83/10891	6.38E-07	1.05E-05
CRTC3	Regulation of RUNX2 expression and activity	47/3917	73/10891	6.94E-07	1.13E-05
CRTC3	Signaling by NOTCH4	51/3917	82/10891	1.13E-06	1.82E-05
CRTC3	C-type lectin receptors (CLRs)	79/3917	142/10891	1.20E-06	1.92E-05
CRTC3	Class I MHC mediated antigen processing & presentation	181/3917	381/10891	1.68E-06	2.62E-05
CRTC3	Synthesis of DNA	69/3917	121/10891	1.69E-06	2.62E-05
CRTC3	Transcriptional regulation by RUNX2	69/3917	121/10891	1.69E-06	2.62E-05
CRTC3	S Phase	88/3917	163/10891	1.71E-06	2.62E-05
CRTC3	PCP/CE pathway	55/3917	92/10891	2.61E-06	3.97E-05
CRTC3	Antigen processing: Ubiquitination & Proteasome degradation	150/3917	309/10891	2.90E-06	4.36E-05
CRTC3	Antiviral mechanism by IFN-stimulated genes	49/3917	80/10891	3.45E-06	5.14E-05
CRTC3	Neddylation	120/3917	240/10891	4.74E-06	6.94E-05
CRTC3	Phospholipid metabolism	108/3917	212/10891	4.75E-06	6.94E-05
CRTC3	mRNA Splicing - Major Pathway	96/3917	185/10891	5.82E-06	8.43E-05
CRTC3	Cell Cycle Checkpoints	142/3917	293/10891	5.92E-06	8.49E-05
CRTC3	SUMOylation of DNA damage response and repair proteins	47/3917	77/10891	6.22E-06	8.85E-05
CRTC3	G1/S Transition	72/3917	131/10891	6.33E-06	8.92E-05
CRTC3	Regulation of PLK1 Activity at G2/M Transition	52/3917	88/10891	7.83E-06	1.09E-04
CRTC3	mRNA Splicing	99/3917	193/10891	8.00E-06	1.11E-04
CRTC3	UCH proteinases	58/3917	102/10891	1.26E-05	1.72E-04
CRTC3	Mitotic Prometaphase	103/3917	204/10891	1.29E-05	1.75E-04
CRTC3	Signaling by TGFB family members	67/3917	122/10891	1.36E-05	1.83E-04
CRTC3	Mitotic G1 phase and G1/S transition	79/3917	149/10891	1.39E-05	1.84E-04
CRTC3	SUMOylation	96/3917	188/10891	1.39E-05	1.84E-04
CRTC3	Signaling by ROBO receptors	108/3917	218/10891	2.33E-05	3.06E-04
CRTC3	Regulation of TP53 Activity	83/3917	160/10891	2.48E-05	3.22E-04
CRTC3	DNA Repair	157/3917	336/10891	2.51E-05	3.25E-04
CRTC3	G2/M Checkpoints	86/3917	167/10891	2.55E-05	3.26E-04
CRTC3	Autophagy	79/3917	151/10891	2.60E-05	3.31E-04
CRTC3	Signaling by TGF-beta Receptor Complex	53/3917	93/10891	2.70E-05	3.40E-04
CRTC3	Deubiquitination	141/3917	298/10891	2.99E-05	3.74E-04
CRTC3	Folding of actin by CCT/TriC	10/3917	10/10891	3.59E-05	4.46E-04
CRTC3	Cellular response to chemical stress	97/3917	194/10891	3.75E-05	4.62E-04
CRTC3	ER-Phagosome pathway	51/3917	90/10891	4.68E-05	5.71E-04

CRTC3	Macroautophagy	71/3917	136/10891	7.15E-05	8.66E-04
CRTC3	Cellular response to heat stress	50/3917	89/10891	7.51E-05	9.02E-04
CRTC3	Regulation of expression of SLITs and ROBOs	86/3917	171/10891	7.76E-05	9.26E-04
CRTC3	Disorders of transmembrane transporters	88/3917	176/10891	8.44E-05	9.99E-04
CRTC3	Antigen processing-Cross presentation	57/3917	105/10891	8.99E-05	1.06E-03
CRTC3	SUMO E3 ligases SUMOylate target proteins	90/3917	182/10891	1.18E-04	1.37E-03
CRTC3	Fcgamma receptor (FCGR) dependent phagocytosis	48/3917	86/10891	1.29E-04	1.49E-03
CRTC3	CDC42 GTPase cycle	78/3917	155/10891	1.60E-04	1.83E-03
CRTC3	Transport of Mature mRNAs Derived from Intronless Transcripts	27/3917	42/10891	1.72E-04	1.96E-03
CRTC3	Toll Like Receptor 3 (TLR3) Cascade	56/3917	105/10891	1.94E-04	2.19E-03
CRTC3	Recruitment of NuMA to mitotic centrosomes	51/3917	94/10891	2.11E-04	2.35E-03
CRTC3	RAC3 GTPase cycle	51/3917	94/10891	2.11E-04	2.35E-03
CRTC3	ISG15 antiviral mechanism	41/3917	72/10891	2.19E-04	2.42E-03
CRTC3	RHOA GTPase cycle	74/3917	147/10891	2.29E-04	2.52E-03
CRTC3	Loss of Nlp from mitotic centrosomes	40/3917	70/10891	2.35E-04	2.55E-03
CRTC3	Loss of proteins required for interphase microtubule organization from the centrosome	40/3917	70/10891	2.35E-04	2.55E-03
CRTC3	RAB GEFs exchange GTP for GDP on RABs	49/3917	90/10891	2.49E-04	2.68E-03
CRTC3	Transcriptional activity of SMAD2/SMAD3:SMAD4 heterotrimer	31/3917	51/10891	2.60E-04	2.78E-03
CRTC3	Role of phospholipids in phagocytosis	18/3917	25/10891	2.64E-04	2.80E-03
CRTC3	Transport of Mature mRNA Derived from an Intronless Transcript	26/3917	41/10891	3.13E-04	3.30E-03
CRTC3	PI Metabolism	46/3917	84/10891	3.20E-04	3.35E-03
CRTC3	Nuclear Pore Complex (NPC) Disassembly	23/3917	35/10891	3.24E-04	3.37E-03
CRTC3	Ub-specific processing proteases	104/3917	220/10891	3.30E-04	3.41E-03
CRTC3	Recruitment of mitotic centrosome proteins and complexes	45/3917	82/10891	3.47E-04	3.54E-03
CRTC3	Centrosome maturation	45/3917	82/10891	3.47E-04	3.54E-03
CRTC3	Nuclear Envelope Breakdown	31/3917	52/10891	4.24E-04	4.28E-03
CRTC3	Signaling by WNT	149/3917	332/10891	4.25E-04	4.28E-03
CRTC3	Signaling by NOTCH	110/3917	236/10891	4.40E-04	4.40E-03
CRTC3	Mitotic Telophase/Cytokinesis	11/3917	13/10891	4.54E-04	4.51E-03
CRTC3	MAPK family signaling cascades	146/3917	325/10891	4.59E-04	4.53E-03
CRTC3	Rab regulation of trafficking	63/3917	124/10891	4.73E-04	4.64E-03
CRTC3	RHOG GTPase cycle	41/3917	74/10891	4.81E-04	4.69E-03
CRTC3	Non-integrin membrane-ECM interactions	34/3917	59/10891	5.49E-04	5.31E-03
CRTC3	SUMOylation of SUMOylation proteins	22/3917	34/10891	6.03E-04	5.80E-03
CRTC3	Signaling by Interleukins	204/3917	473/10891	6.06E-04	5.80E-03
CRTC3	Transcriptional Regulation by TP53	160/3917	362/10891	6.29E-04	5.91E-03
CRTC3	Signaling by ALK in cancer	32/3917	55/10891	6.29E-04	5.91E-03

CRTC3	Signaling by ALK fusions and activated point mutants	32/3917	55/10891	6.29E-04	5.91E-03
CRTC3	Cohesin Loading onto Chromatin	9/3917	10/10891	6.77E-04	6.29E-03
CRTC3	Processing and activation of SUMO	9/3917	10/10891	6.77E-04	6.29E-03
CRTC3	RAC1 GTPase cycle	88/3917	185/10891	7.15E-04	6.57E-03
CRTC3	COPI-independent Golgi-to-ER retrograde traffic	30/3917	51/10891	7.17E-04	6.57E-03
CRTC3	AURKA Activation by TPX2	40/3917	73/10891	7.56E-04	6.89E-03
CRTC3	SARS-CoV Infections	165/3917	376/10891	7.77E-04	7.04E-03
CRTC3	SUMOylation of RNA binding proteins	28/3917	47/10891	8.10E-04	7.30E-03
CRTC3	Regulation of HSF1-mediated heat shock response	38/3917	69/10891	8.92E-04	7.91E-03
CRTC3	MyD88-independent TLR4 cascade	56/3917	110/10891	8.93E-04	7.91E-03
CRTC3	TRIF(TICAM1)-mediated TLR4 signaling	56/3917	110/10891	8.93E-04	7.91E-03
CRTC3	ZBP1(DAI) mediated induction of type I IFNs	15/3917	21/10891	1.01E-03	8.88E-03
CRTC3	Opioid Signalling	48/3917	92/10891	1.02E-03	8.97E-03
CRTC3	Transport of the SLBP Dependant Mature mRNA	22/3917	35/10891	1.07E-03	9.36E-03
CRTC3	Nuclear import of Rev protein	21/3917	33/10891	1.11E-03	9.57E-03
CRTC3	Cyclin A/B1/B2 associated events during G2/M transition	17/3917	25/10891	1.12E-03	9.61E-03
CRTC3	Transport of Ribonucleoproteins into the Host Nucleus	20/3917	31/10891	1.13E-03	9.61E-03
CRTC3	NEP/NS2 Interacts with the Cellular Export Machinery	20/3917	31/10891	1.13E-03	9.61E-03
CRTC3	Selective autophagy	43/3917	81/10891	1.17E-03	9.87E-03
CRTC3	Inositol phosphate metabolism	29/3917	50/10891	1.20E-03	1.01E-02
CRTC3	Regulation of PTEN gene transcription	34/3917	61/10891	1.24E-03	1.04E-02
CRTC3	Interferon Signaling	93/3917	200/10891	1.28E-03	1.07E-02
CRTC3	Translation of Replicase and Assembly of the Replication Transcription Complex	11/3917	14/10891	1.43E-03	1.18E-02
CRTC3	Resolution of Sister Chromatid Cohesion	62/3917	126/10891	1.49E-03	1.22E-02
CRTC3	SARS-CoV-2 activates/modulates innate and adaptive immune responses	62/3917	126/10891	1.49E-03	1.22E-02
CRTC3	Nuclear Envelope (NE) Reassembly	40/3917	75/10891	1.52E-03	1.24E-02
CRTC3	Cilium Assembly	93/3917	201/10891	1.56E-03	1.26E-02
CRTC3	Cytosolic sensors of pathogen-associated DNA	35/3917	64/10891	1.66E-03	1.33E-02
CRTC3	Signaling by FGFR2	39/3917	73/10891	1.66E-03	1.33E-02
CRTC3	Glycerophospholipid biosynthesis	63/3917	129/10891	1.73E-03	1.38E-02
CRTC3	Early SARS-CoV-2 Infection Events	23/3917	38/10891	1.74E-03	1.38E-02
CRTC3	Glycolysis	38/3917	71/10891	1.81E-03	1.43E-02
CRTC3	Interactions of Rev with host cellular proteins	22/3917	36/10891	1.83E-03	1.44E-02
CRTC3	Intra-Golgi and retrograde Golgi-to-ER traffic	93/3917	202/10891	1.88E-03	1.47E-02
CRTC3	Transport of the SLBP independent Mature mRNA	21/3917	34/10891	1.92E-03	1.49E-02

CRTC3	Resolution of D-loop Structures through Holliday Junction Intermediates	21/3917	34/10891	1.92E-03	1.49E-02
CRTC3	Costimulation by the CD28 family	37/3917	69/10891	1.98E-03	1.53E-02
CRTC3	VEGFA-VEGFR2 Pathway	50/3917	99/10891	2.05E-03	1.56E-02
CRTC3	Downregulation of SMAD2/3:SMAD4 transcriptional activity	19/3917	30/10891	2.06E-03	1.56E-02
CRTC3	Laminin interactions	19/3917	30/10891	2.06E-03	1.56E-02
CRTC3	Signaling by CSF3 (G-CSF)	19/3917	30/10891	2.06E-03	1.56E-02
CRTC3	Synthesis of PC	18/3917	28/10891	2.12E-03	1.59E-02
CRTC3	Cytosolic tRNA aminoacylation	16/3917	24/10891	2.15E-03	1.61E-02
CRTC3	RHOC GTPase cycle	39/3917	74/10891	2.30E-03	1.72E-02
CRTC3	G-protein mediated events	31/3917	56/10891	2.32E-03	1.72E-02
CRTC3	RAC2 GTPase cycle	45/3917	88/10891	2.45E-03	1.80E-02
CRTC3	Viral Messenger RNA Synthesis	25/3917	43/10891	2.45E-03	1.80E-02
CRTC3	Interleukin-6 signaling	9/3917	11/10891	2.53E-03	1.84E-02
CRTC3	Golgi-to-ER retrograde transport	64/3917	133/10891	2.53E-03	1.84E-02
CRTC3	MAPK1/MAPK3 signaling	126/3917	286/10891	2.60E-03	1.88E-02
CRTC3	tRNA processing	53/3917	107/10891	2.64E-03	1.90E-02
CRTC3	Regulation of actin dynamics for phagocytic cup formation	33/3917	61/10891	2.81E-03	2.02E-02
CRTC3	RHO GTPase Effectors	142/3917	327/10891	2.84E-03	2.03E-02
CRTC3	Late Phase of HIV Life Cycle	65/3917	136/10891	2.89E-03	2.06E-02
CRTC3	RHOQ GTPase cycle	32/3917	59/10891	3.07E-03	2.17E-02
CRTC3	Resolution of D-Loop Structures	21/3917	35/10891	3.18E-03	2.23E-02
CRTC3	Negative regulators of DDX58/IFIH1 signaling	21/3917	35/10891	3.18E-03	2.23E-02
CRTC3	Export of Viral Ribonucleoproteins from Nucleus	20/3917	33/10891	3.37E-03	2.34E-02
CRTC3	FCERI mediated Ca+2 mobilization	20/3917	33/10891	3.37E-03	2.34E-02
CRTC3	HIV Life Cycle	70/3917	149/10891	3.51E-03	2.43E-02
CRTC3	Regulation of Glucokinase by Glucokinase Regulatory Protein	19/3917	31/10891	3.55E-03	2.43E-02
CRTC3	Defective TPR may confer susceptibility towards thyroid papillary carcinoma (TPC)	19/3917	31/10891	3.55E-03	2.43E-02
CRTC3	Translation of Replicase and Assembly of the Replication Transcription Complex	11/3917	15/10891	3.61E-03	2.46E-02
CRTC3	Potential therapeutics for SARS	49/3917	99/10891	3.82E-03	2.60E-02
CRTC3	Regulation of TP53 Activity through Phosphorylation	46/3917	92/10891	3.88E-03	2.62E-02
CRTC3	RIP-mediated NFkB activation via ZBP1	12/3917	17/10891	3.89E-03	2.62E-02
CRTC3	MHC class II antigen presentation	59/3917	123/10891	3.98E-03	2.63E-02
CRTC3	Synthesis of PIPs at the plasma membrane	29/3917	53/10891	3.98E-03	2.63E-02
CRTC3	SARS-CoV-1 Infection	29/3917	53/10891	3.98E-03	2.63E-02
CRTC3	Formation of tubulin folding intermediates by CCT/TriC	16/3917	25/10891	3.99E-03	2.63E-02

CRTC3	Inactivation of CSF3 (G-CSF) signaling	16/3917	25/10891	3.99E-03	2.63E-02
CRTC3	Signal transduction by L1	14/3917	21/10891	4.10E-03	2.69E-02
CRTC3	PLC beta mediated events	28/3917	51/10891	4.34E-03	2.84E-02
CRTC3	FCGR3A-mediated IL10 synthesis	23/3917	40/10891	4.38E-03	2.85E-02
CRTC3	Metabolism of amino acids and derivatives	159/3917	374/10891	4.58E-03	2.97E-02
CRTC3	RHOV GTPase cycle	22/3917	38/10891	4.72E-03	3.05E-02
CRTC3	Eukaryotic Translation Initiation	57/3917	119/10891	4.77E-03	3.06E-02
CRTC3	Cap-dependent Translation Initiation	57/3917	119/10891	4.77E-03	3.06E-02
CRTC3	GTP hydrolysis and joining of the 60S ribosomal subunit	54/3917	112/10891	4.99E-03	3.19E-02
CRTC3	Toll-like Receptor Cascades	77/3917	168/10891	5.07E-03	3.20E-02
CRTC3	SMAD2/SMAD3:SMAD4 heterotrimer regulates transcription	21/3917	36/10891	5.07E-03	3.20E-02
CRTC3	Regulation of TP53 Degradation	21/3917	36/10891	5.07E-03	3.20E-02
CRTC3	Signaling by MET	40/3917	79/10891	5.18E-03	3.25E-02
CRTC3	Organelle biogenesis and maintenance	128/3917	296/10891	5.27E-03	3.29E-02
CRTC3	Rev-mediated nuclear export of HIV RNA	20/3917	34/10891	5.44E-03	3.39E-02
CRTC3	Glucose metabolism	45/3917	91/10891	5.54E-03	3.43E-02
CRTC3	SUMOylation of transcription cofactors	25/3917	45/10891	5.60E-03	3.45E-02
CRTC3	SUMOylation of DNA replication proteins	25/3917	45/10891	5.60E-03	3.45E-02
CRTC3	Transport of Mature Transcript to Cytoplasm	42/3917	84/10891	5.64E-03	3.46E-02
CRTC3	RHO GTPases activate IQGAPs	19/3917	32/10891	5.82E-03	3.53E-02
CRTC3	MAPK3 (ERK1) activation	8/3917	10/10891	5.83E-03	3.53E-02
CRTC3	Regulation of FOXO transcriptional activity by acetylation	8/3917	10/10891	5.83E-03	3.53E-02
CRTC3	rRNA modification in the nucleus and cytosol	32/3917	61/10891	6.02E-03	3.63E-02
CRTC3	tRNA modification in the nucleus and cytosol	24/3917	43/10891	6.09E-03	3.66E-02
CRTC3	Synthesis of active ubiquitin: roles of E1 and E2 enzymes	18/3917	30/10891	6.20E-03	3.70E-02
CRTC3	Transcriptional regulation by RUNX1	105/3917	239/10891	6.21E-03	3.70E-02
CRTC3	Synthesis of IP3 and IP4 in the cytosol	17/3917	28/10891	6.57E-03	3.90E-02
CRTC3	Signaling by FGFR	43/3917	87/10891	6.68E-03	3.95E-02
CRTC3	RAF/MAP kinase cascade	121/3917	280/10891	6.70E-03	3.95E-02
CRTC3	L13a-mediated translational silencing of Ceruloplasmin expression	53/3917	111/10891	6.87E-03	4.03E-02
CRTC3	SARS-CoV-2 Infection	124/3917	288/10891	7.05E-03	4.12E-02
CRTC3	Translesion synthesis by Y family DNA polymerases bypasses lesions on DNA template	22/3917	39/10891	7.19E-03	4.17E-02
CRTC3	Association of TriC/CCT with target proteins during biosynthesis	22/3917	39/10891	7.19E-03	4.17E-02
CRTC3	Growth hormone receptor signaling	15/3917	24/10891	7.27E-03	4.21E-02
CRTC3	DNA Damage Bypass	26/3917	48/10891	7.44E-03	4.29E-02
CRTC3	Toll Like Receptor 10 (TLR10) Cascade	47/3917	97/10891	7.53E-03	4.29E-02

CRTC3	Toll Like Receptor 5 (TLR5) Cascade	47/3917	97/10891	7.53E-03	4.29E-02
CRTC3	MyD88 cascade initiated on plasma membrane	47/3917	97/10891	7.53E-03	4.29E-02
CRTC3	MAP3K8 (TPL2)-dependent MAPK1/3 activation	11/3917	16/10891	7.80E-03	4.38E-02
CRTC3	FOXO-mediated transcription of cell death genes	11/3917	16/10891	7.80E-03	4.38E-02
CRTC3	HDR through Single Strand Annealing (SSA)	21/3917	37/10891	7.80E-03	4.38E-02
CRTC3	Regulation of TP53 Expression and Degradation	21/3917	37/10891	7.80E-03	4.38E-02
CRTC3	Signaling by cytosolic FGFR1 fusion mutants	12/3917	18/10891	7.87E-03	4.40E-02
CRTC3	DNA Replication	84/3917	188/10891	8.04E-03	4.48E-02
CRTC3	Ca2+ pathway	32/3917	62/10891	8.21E-03	4.56E-02
CRTC3	RHOBTB GTPase Cycle	20/3917	35/10891	8.45E-03	4.68E-02
DCAF12	RHO GTPase cycle	136/1912	449/10891	1.04E-11	1.57E-08
DCAF12	RHO GTPases Activate Formins	55/1912	140/10891	8.53E-10	5.63E-07
DCAF12	Mitotic Spindle Checkpoint	47/1912	113/10891	1.59E-09	5.63E-07
DCAF12	Amplification of signal from the kinetochores	42/1912	96/10891	1.86E-09	5.63E-07
DCAF12	Amplification of signal from unattached kinetochores via a MAD2 inhibitory signal	42/1912	96/10891	1.86E-09	5.63E-07
DCAF12	Resolution of Sister Chromatid Cohesion	50/1912	126/10891	3.32E-09	8.37E-07
DCAF12	Mitotic Prometaphase	70/1912	204/10891	4.73E-09	1.02E-06
DCAF12	RAC1 GTPase cycle	65/1912	185/10891	5.77E-09	1.09E-06
DCAF12	EML4 and NUDC in mitotic spindle formation	45/1912	117/10891	6.12E-08	1.03E-05
DCAF12	Mitotic Metaphase and Anaphase	75/1912	237/10891	6.91E-08	1.04E-05
DCAF12	Mitotic Anaphase	74/1912	236/10891	1.28E-07	1.76E-05
DCAF12	Cell Cycle Checkpoints	87/1912	293/10891	1.63E-07	2.05E-05
DCAF12	RHO GTPase Effectors	94/1912	327/10891	2.75E-07	3.20E-05
DCAF12	Asparagine N-linked glycosylation	86/1912	304/10891	1.83E-06	1.98E-04
DCAF12	RHOA GTPase cycle	49/1912	147/10891	2.47E-06	2.49E-04
DCAF12	RHOD GTPase cycle	24/1912	54/10891	3.93E-06	3.59E-04
DCAF12	Separation of Sister Chromatids	59/1912	191/10891	4.04E-06	3.59E-04
DCAF12	CDC42 GTPase cycle	50/1912	155/10891	5.65E-06	4.56E-04
DCAF12	M Phase	109/1912	418/10891	5.73E-06	4.56E-04
DCAF12	Polo-like kinase mediated events	11/1912	16/10891	8.72E-06	6.59E-04
DCAF12	RAC3 GTPase cycle	34/1912	94/10891	1.19E-05	8.06E-04
DCAF12	Translation of Structural Proteins	24/1912	57/10891	1.23E-05	8.06E-04
DCAF12	Late SARS-CoV-2 Infection Events	24/1912	57/10891	1.23E-05	8.06E-04
DCAF12	Intra-Golgi and retrograde Golgi-to-ER traffic	60/1912	202/10891	1.31E-05	8.27E-04
DCAF12	Maturation of spike protein	18/1912	37/10891	1.37E-05	8.30E-04
DCAF12	RAC2 GTPase cycle	32/1912	88/10891	1.88E-05	1.09E-03
DCAF12	RHOF GTPase cycle	19/1912	42/10891	2.93E-05	1.64E-03
DCAF12	COPI-dependent Golgi-to-ER retrograde traffic	34/1912	99/10891	4.15E-05	2.24E-03
DCAF12	Death Receptor Signalling	44/1912	141/10891	5.00E-05	2.61E-03
DCAF12	Golgi-to-ER retrograde transport	42/1912	133/10891	5.41E-05	2.73E-03
DCAF12	G1/S-Specific Transcription	14/1912	28/10891	8.59E-05	4.19E-03
DCAF12	Condensation of Prometaphase Chromosomes	8/1912	11/10891	8.87E-05	4.19E-03

DCAF12	Kinesins	22/1912	59/10891	2.42E-04	1.11E-02
DCAF12	G0 and Early G1	13/1912	27/10891	2.50E-04	1.11E-02
DCAF12	GPVI-mediated activation cascade	15/1912	35/10891	4.17E-04	1.80E-02
DCAF12	Antigen activates B Cell Receptor (BCR) leading to generation of second messengers	14/1912	32/10891	5.01E-04	2.11E-02
DCAF12	Initiation of Nuclear Envelope (NE) Reformation	10/1912	19/10891	5.37E-04	2.19E-02
DCAF12	RHOG GTPase cycle	25/1912	74/10891	5.51E-04	2.19E-02
DCAF12	RHOJ GTPase cycle	20/1912	55/10891	6.69E-04	2.59E-02
DCAF12	TP53 Regulates Transcription of Cell Cycle Genes	18/1912	48/10891	8.06E-04	3.05E-02
DCAF12	p75 NTR receptor-mediated signalling	30/1912	97/10891	8.82E-04	3.25E-02
DCAF12	Regulated Necrosis	20/1912	57/10891	1.12E-03	4.04E-02
DCAF12	RHOH GTPase cycle	15/1912	38/10891	1.17E-03	4.13E-02
DCAF12	Biosynthesis of the N-glycan precursor (dolichol lipid-linked oligosaccharide, LLO) and transfer to a nascent protein	25/1912	78/10891	1.31E-03	4.50E-02
DENND4C	Diseases of signal transduction by growth factor receptors and second messengers	212/3901	433/10891	7.41E-09	1.15E-05
DENND4C	Asparagine N-linked glycosylation	156/3901	304/10891	1.52E-08	1.18E-05
DENND4C	HIV Life Cycle	85/3901	149/10891	8.72E-08	4.46E-05
DENND4C	Transport to the Golgi and subsequent modification	101/3901	185/10891	1.15E-07	4.46E-05
DENND4C	SARS-CoV Infections	182/3901	376/10891	2.39E-07	7.41E-05
DENND4C	Intra-Golgi and retrograde Golgi-to-ER traffic	107/3901	202/10891	3.76E-07	9.69E-05
DENND4C	Late Phase of HIV Life Cycle	77/3901	136/10891	5.30E-07	1.06E-04
DENND4C	HIV Infection	119/3901	231/10891	5.95E-07	1.06E-04
DENND4C	ER to Golgi Anterograde Transport	85/3901	154/10891	6.16E-07	1.06E-04
DENND4C	Processing of Capped Intron-Containing Pre-mRNA	125/3901	246/10891	8.09E-07	1.25E-04
DENND4C	ISG15 antiviral mechanism	46/3901	72/10891	1.10E-06	1.54E-04
DENND4C	RHO GTPase cycle	209/3901	449/10891	1.19E-06	1.54E-04
DENND4C	Citric acid cycle (TCA cycle)	19/3901	22/10891	1.46E-06	1.74E-04
DENND4C	Influenza Infection	83/3901	156/10891	5.93E-06	6.55E-04
DENND4C	Regulation of cholesterol biosynthesis by SREBP (SREBF)	36/3901	55/10891	7.17E-06	7.36E-04
DENND4C	SARS-CoV-2 Infection	139/3901	288/10891	7.62E-06	7.36E-04
DENND4C	Antiviral mechanism by IFN-stimulated genes	48/3901	80/10891	8.57E-06	7.80E-04
DENND4C	Autophagy	80/3901	151/10891	1.07E-05	9.04E-04
DENND4C	NS1 Mediated Effects on Host Pathways	28/3901	40/10891	1.11E-05	9.04E-04
DENND4C	DNA Damage Bypass	32/3901	48/10891	1.31E-05	1.01E-03
DENND4C	tRNA processing	60/3901	107/10891	1.40E-05	1.03E-03
DENND4C	Signaling by NOTCH4	48/3901	82/10891	2.17E-05	1.53E-03
DENND4C	Pyruvate metabolism and Citric Acid (TCA) cycle	35/3901	55/10891	2.40E-05	1.62E-03
DENND4C	mRNA Splicing - Major Pathway	93/3901	185/10891	3.45E-05	2.23E-03

DENND4C	Transcription-Coupled Nucleotide Excision Repair (TC-NER)	46/3901	79/10891	3.86E-05	2.37E-03
DENND4C	Regulation of TP53 Activity	82/3901	160/10891	4.11E-05	2.37E-03
DENND4C	RAB GEFs exchange GTP for GDP on RABs	51/3901	90/10891	4.13E-05	2.37E-03
DENND4C	Influenza Viral RNA Transcription and Replication	71/3901	135/10891	4.51E-05	2.41E-03
DENND4C	Transport of Mature mRNAs Derived from Intronless Transcripts	28/3901	42/10891	4.56E-05	2.41E-03
DENND4C	Signaling by VEGF	59/3901	108/10891	4.67E-05	2.41E-03
DENND4C	Signaling by Hedgehog	77/3901	149/10891	4.84E-05	2.42E-03
DENND4C	KEAP1-NFE2L2 pathway	56/3901	102/10891	6.00E-05	2.89E-03
DENND4C	Macroautophagy	71/3901	136/10891	6.16E-05	2.89E-03
DENND4C	Regulation of expression of SLITs and ROBOs	86/3901	171/10891	6.57E-05	2.90E-03
DENND4C	Neddylation	115/3901	240/10891	6.70E-05	2.90E-03
DENND4C	Signaling by NTRKs	70/3901	134/10891	6.74E-05	2.90E-03
DENND4C	Transport of Ribonucleoproteins into the Host Nucleus	22/3901	31/10891	7.15E-05	2.99E-03
DENND4C	Vpr-mediated nuclear import of PICs	23/3901	33/10891	7.67E-05	3.12E-03
DENND4C	Intracellular signaling by second messengers	143/3901	309/10891	8.08E-05	3.17E-03
DENND4C	Retrograde transport at the Trans-Golgi-Network	31/3901	49/10891	8.22E-05	3.17E-03
DENND4C	Viral Messenger RNA Synthesis	28/3901	43/10891	8.61E-05	3.17E-03
DENND4C	Transport of Mature mRNA Derived from an Intronless Transcript	27/3901	41/10891	8.61E-05	3.17E-03
DENND4C	RNA Polymerase II Pre-transcription Events	46/3901	81/10891	9.02E-05	3.25E-03
DENND4C	COPI-mediated anterograde transport	55/3901	101/10891	9.36E-05	3.27E-03
DENND4C	Cellular response to starvation	79/3901	156/10891	9.53E-05	3.27E-03
DENND4C	Cytosolic sensors of pathogen-associated DNA	38/3901	64/10891	1.00E-04	3.34E-03
DENND4C	SUMOylation of DNA damage response and repair proteins	44/3901	77/10891	1.05E-04	3.34E-03
DENND4C	Deadenylation of mRNA	18/3901	24/10891	1.05E-04	3.34E-03
DENND4C	Signaling by ROBO receptors	105/3901	218/10891	1.06E-04	3.34E-03
DENND4C	Signaling by NTRK1 (TRKA)	61/3901	115/10891	1.10E-04	3.35E-03
DENND4C	Organelle biogenesis and maintenance	137/3901	296/10891	1.12E-04	3.35E-03
DENND4C	Cellular response to hypoxia	43/3901	75/10891	1.13E-04	3.35E-03
DENND4C	SARS-CoV-2-host interactions	98/3901	202/10891	1.26E-04	3.62E-03
DENND4C	Signaling by PTK6	33/3901	54/10891	1.31E-04	3.62E-03
DENND4C	Signaling by Non-Receptor Tyrosine Kinases	33/3901	54/10891	1.31E-04	3.62E-03
DENND4C	Nucleotide Excision Repair	59/3901	111/10891	1.31E-04	3.62E-03
DENND4C	SUMOylation	92/3901	188/10891	1.36E-04	3.65E-03
DENND4C	Nuclear Envelope Breakdown	32/3901	52/10891	1.37E-04	3.65E-03
DENND4C	mRNA Splicing	94/3901	193/10891	1.44E-04	3.76E-03
DENND4C	Signalling to ERKs	23/3901	34/10891	1.57E-04	3.93E-03
DENND4C	Signaling by Insulin receptor	44/3901	78/10891	1.57E-04	3.93E-03
DENND4C	Interactions of Vpr with host cellular proteins	24/3901	36/10891	1.60E-04	3.93E-03

DENND4C	Regulation of TP53 Degradation	24/3901	36/10891	1.60E-04	3.93E-03
DENND4C	Transcriptional regulation by RUNX3	52/3901	96/10891	1.72E-04	4.17E-03
DENND4C	S Phase	81/3901	163/10891	1.76E-04	4.20E-03
DENND4C	Chromatin modifying enzymes	127/3901	274/10891	1.82E-04	4.21E-03
DENND4C	Chromatin organization	127/3901	274/10891	1.82E-04	4.21E-03
DENND4C	PIP3 activates AKT signaling	124/3901	267/10891	1.95E-04	4.40E-03
DENND4C	Degradation of beta-catenin by the destruction complex	46/3901	83/10891	1.98E-04	4.40E-03
DENND4C	PTEN Regulation	71/3901	140/10891	1.99E-04	4.40E-03
DENND4C	Rab regulation of trafficking	64/3901	124/10891	2.13E-04	4.57E-03
DENND4C	Intrinsic Pathway for Apoptosis	33/3901	55/10891	2.15E-04	4.57E-03
DENND4C	Amino acids regulate mTORC1	33/3901	55/10891	2.15E-04	4.57E-03
DENND4C	RAF/MAP kinase cascade	129/3901	280/10891	2.24E-04	4.60E-03
DENND4C	snRNP Assembly	32/3901	53/10891	2.27E-04	4.60E-03
DENND4C	Metabolism of non-coding RNA	32/3901	53/10891	2.27E-04	4.60E-03
DENND4C	Regulation of RAS by GAPs	39/3901	68/10891	2.29E-04	4.60E-03
DENND4C	Dual incision in TC-NER	38/3901	66/10891	2.46E-04	4.86E-03
DENND4C	Hedgehog 'off' state	59/3901	113/10891	2.48E-04	4.86E-03
DENND4C	Activated NTRK2 signals through FRS2 and FRS3	10/3901	11/10891	2.56E-04	4.91E-03
DENND4C	Deubiquitination	136/3901	298/10891	2.57E-04	4.91E-03
DENND4C	MAPK1/MAPK3 signaling	131/3901	286/10891	2.74E-04	5.16E-03
DENND4C	MTOR signalling	26/3901	41/10891	2.90E-04	5.20E-03
DENND4C	DNA Repair	151/3901	336/10891	2.93E-04	5.20E-03
DENND4C	Regulation of Glucokinase by Glucokinase Regulatory Protein	21/3901	31/10891	2.95E-04	5.20E-03
DENND4C	Defective TPR may confer susceptibility towards thyroid papillary carcinoma (TPC)	21/3901	31/10891	2.95E-04	5.20E-03
DENND4C	Export of Viral Ribonucleoproteins from Nucleus	22/3901	33/10891	3.01E-04	5.20E-03
DENND4C	Regulation of TP53 Expression and Degradation	24/3901	37/10891	3.01E-04	5.20E-03
DENND4C	Transport of the SLBP Dependant Mature mRNA	23/3901	35/10891	3.03E-04	5.20E-03
DENND4C	Late endosomal microautophagy	23/3901	35/10891	3.03E-04	5.20E-03
DENND4C	Signaling by PDGF	34/3901	58/10891	3.24E-04	5.51E-03
DENND4C	RORA activates gene expression	14/3901	18/10891	3.43E-04	5.70E-03
DENND4C	Cellular response to heat stress	48/3901	89/10891	3.45E-04	5.70E-03
DENND4C	Regulation of PTEN stability and activity	39/3901	69/10891	3.46E-04	5.70E-03
DENND4C	Formation of TC-NER Pre-Incision Complex	32/3901	54/10891	3.68E-04	5.99E-03
DENND4C	MAPK family signaling cascades	146/3901	325/10891	3.74E-04	6.02E-03
DENND4C	Gap-filling DNA repair synthesis and ligation in TC-NER	37/3901	65/10891	4.03E-04	6.42E-03
DENND4C	Signaling by PDGFR in disease	15/3901	20/10891	4.10E-04	6.42E-03
DENND4C	Hedgehog 'on' state	46/3901	85/10891	4.11E-04	6.42E-03
DENND4C	Signaling by NOTCH1	41/3901	74/10891	4.36E-04	6.75E-03
DENND4C	VEGFA-VEGFR2 Pathway	52/3901	99/10891	4.69E-04	7.18E-03

DENND4C	Activation of gene expression by SREBF (SREBP)	26/3901	42/10891	5.04E-04	7.64E-03
DENND4C	Transcriptional Regulation by TP53	160/3901	362/10891	5.09E-04	7.64E-03
DENND4C	Nuclear events mediated by NFE2L2	43/3901	79/10891	5.33E-04	7.90E-03
DENND4C	Stabilization of p53	33/3901	57/10891	5.40E-04	7.90E-03
DENND4C	SUMO E3 ligases SUMOylate target proteins	87/3901	182/10891	5.41E-04	7.90E-03
DENND4C	FLT3 signaling in disease	19/3901	28/10891	5.58E-04	7.91E-03
DENND4C	Transport of the SLBP independent Mature mRNA	22/3901	34/10891	5.65E-04	7.91E-03
DENND4C	Recognition of DNA damage by PCNA-containing replication complex	20/3901	30/10891	5.68E-04	7.91E-03
DENND4C	Activation of BH3-only proteins	20/3901	30/10891	5.68E-04	7.91E-03
DENND4C	Regulation of TP53 Activity through Acetylation	20/3901	30/10891	5.68E-04	7.91E-03
DENND4C	Host Interactions of HIV factors	65/3901	130/10891	5.97E-04	8.19E-03
DENND4C	p53-Dependent G1 DNA Damage Response	37/3901	66/10891	6.04E-04	8.19E-03
DENND4C	p53-Dependent G1/S DNA damage checkpoint	37/3901	66/10891	6.04E-04	8.19E-03
DENND4C	Class I MHC mediated antigen processing & presentation	167/3901	381/10891	6.23E-04	8.38E-03
DENND4C	Transport of Mature Transcript to Cytoplasm	45/3901	84/10891	6.34E-04	8.45E-03
DENND4C	Cohesin Loading onto Chromatin	9/3901	10/10891	6.54E-04	8.65E-03
DENND4C	Formation of RNA Pol II elongation complex	33/3901	58/10891	8.26E-04	1.03E-02
DENND4C	Signaling by NOTCH1 PEST Domain Mutants in Cancer	33/3901	58/10891	8.26E-04	1.03E-02
DENND4C	Signaling by NOTCH1 in Cancer	33/3901	58/10891	8.26E-04	1.03E-02
DENND4C	Constitutive Signaling by NOTCH1 PEST Domain Mutants	33/3901	58/10891	8.26E-04	1.03E-02
DENND4C	Signaling by NOTCH1 HD+PEST Domain Mutants in Cancer	33/3901	58/10891	8.26E-04	1.03E-02
DENND4C	Constitutive Signaling by NOTCH1 HD+PEST Domain Mutants	33/3901	58/10891	8.26E-04	1.03E-02
DENND4C	RNA Polymerase II Transcription Elongation	33/3901	58/10891	8.26E-04	1.03E-02
DENND4C	Constitutive Signaling by Ligand-Responsive EGFR Cancer Variants	14/3901	19/10891	8.72E-04	1.07E-02
DENND4C	Signaling by Ligand-Responsive EGFR Variants in Cancer	14/3901	19/10891	8.72E-04	1.07E-02
DENND4C	AUF1 (hnRNP D0) binds and destabilizes mRNA	32/3901	56/10891	8.92E-04	1.09E-02
DENND4C	Translesion synthesis by Y family DNA polymerases bypasses lesions on DNA template	24/3901	39/10891	9.31E-04	1.11E-02
DENND4C	Association of TriC/CCT with target proteins during biosynthesis	24/3901	39/10891	9.31E-04	1.11E-02
DENND4C	Clathrin-mediated endocytosis	71/3901	146/10891	9.36E-04	1.11E-02
DENND4C	Vif-mediated degradation of APOBEC3G	31/3901	54/10891	9.61E-04	1.13E-02
DENND4C	Negative regulation of NOTCH4 signaling	31/3901	54/10891	9.61E-04	1.13E-02
DENND4C	Downstream signaling events of B Cell Receptor	44/3901	83/10891	9.68E-04	1.13E-02

	(BCR)				
DENND4C	Apoptosis	85/3901	180/10891	9.99E-04	1.14E-02
DENND4C	Nuclear Pore Complex (NPC) Disassembly	22/3901	35/10891	1.01E-03	1.14E-02
DENND4C	Autodegradation of the E3 ubiquitin ligase COP1	30/3901	52/10891	1.03E-03	1.14E-02
DENND4C	Ubiquitin-dependent degradation of Cyclin D	30/3901	52/10891	1.03E-03	1.14E-02
DENND4C	TP53 Regulates Transcription of Caspase Activators and Caspases	10/3901	12/10891	1.04E-03	1.14E-02
DENND4C	Signaling by PDGFRA transmembrane, juxtamembrane and kinase domain mutants	10/3901	12/10891	1.04E-03	1.14E-02
DENND4C	Signaling by PDGFRA extracellular domain mutants	10/3901	12/10891	1.04E-03	1.14E-02
DENND4C	Nuclear import of Rev protein	21/3901	33/10891	1.04E-03	1.14E-02
DENND4C	Signaling by EGFR in Cancer	17/3901	25/10891	1.06E-03	1.14E-02
DENND4C	Selective autophagy	43/3901	81/10891	1.06E-03	1.14E-02
DENND4C	NEP/NS2 Interacts with the Cellular Export Machinery	20/3901	31/10891	1.06E-03	1.14E-02
DENND4C	Signaling by EGFR	29/3901	50/10891	1.11E-03	1.18E-02
DENND4C	Regulation of mRNA stability by proteins that bind AU-rich elements	46/3901	88/10891	1.11E-03	1.18E-02
DENND4C	Defective CFTR causes cystic fibrosis	34/3901	61/10891	1.14E-03	1.19E-02
DENND4C	Mitochondrial biogenesis	49/3901	95/10891	1.14E-03	1.19E-02
DENND4C	Transcription of the HIV genome	38/3901	70/10891	1.17E-03	1.22E-02
DENND4C	NIK-->noncanonical NF-kB signaling	33/3901	59/10891	1.24E-03	1.28E-02
DENND4C	Golgi-to-ER retrograde transport	65/3901	133/10891	1.28E-03	1.30E-02
DENND4C	COPII-mediated vesicle transport	37/3901	68/10891	1.28E-03	1.30E-02
DENND4C	G1/S DNA Damage Checkpoints	37/3901	68/10891	1.28E-03	1.30E-02
DENND4C	SARS-CoV-2 activates/modulates innate and adaptive immune responses	62/3901	126/10891	1.32E-03	1.33E-02
DENND4C	tRNA processing in the nucleus	32/3901	57/10891	1.34E-03	1.34E-02
DENND4C	Intra-Golgi traffic	26/3901	44/10891	1.36E-03	1.35E-02
DENND4C	Prolonged ERK activation events	11/3901	14/10891	1.37E-03	1.35E-02
DENND4C	Oxygen-dependent proline hydroxylation of Hypoxia-inducible Factor Alpha	36/3901	66/10891	1.40E-03	1.37E-02
DENND4C	Cellular response to chemical stress	90/3901	194/10891	1.44E-03	1.40E-02
DENND4C	Deactivation of the beta-catenin transactivating complex	25/3901	42/10891	1.45E-03	1.40E-02
DENND4C	Translation	129/3901	291/10891	1.49E-03	1.43E-02
DENND4C	ABC-family proteins mediated transport	52/3901	103/10891	1.53E-03	1.46E-02
DENND4C	Fanconi Anemia Pathway	24/3901	40/10891	1.54E-03	1.46E-02
DENND4C	Regulation of Apoptosis	30/3901	53/10891	1.57E-03	1.47E-02
DENND4C	Antigen processing: Ubiquitination & Proteasome degradation	136/3901	309/10891	1.58E-03	1.47E-02
DENND4C	STING mediated induction of host immune	12/3901	16/10891	1.63E-03	1.47E-02

	responses				
DENND4C	NRIF signals cell death from the nucleus	12/3901	16/10891	1.63E-03	1.47E-02
DENND4C	SUMOylation of ubiquitinylation proteins	23/3901	38/10891	1.63E-03	1.47E-02
DENND4C	DNA Damage Recognition in GG-NER	23/3901	38/10891	1.63E-03	1.47E-02
DENND4C	FLT3 Signaling	23/3901	38/10891	1.63E-03	1.47E-02
DENND4C	Nonsense-Mediated Decay (NMD)	57/3901	115/10891	1.64E-03	1.47E-02
DENND4C	Nonsense Mediated Decay (NMD) enhanced by the Exon Junction Complex (EJC)	57/3901	115/10891	1.64E-03	1.47E-02
DENND4C	Interactions of Rev with host cellular proteins	22/3901	36/10891	1.72E-03	1.53E-02
DENND4C	RAF activation	22/3901	36/10891	1.72E-03	1.53E-02
DENND4C	Rev-mediated nuclear export of HIV RNA	21/3901	34/10891	1.81E-03	1.56E-02
DENND4C	SUMOylation of SUMOylation proteins	21/3901	34/10891	1.81E-03	1.56E-02
DENND4C	Dectin-1 mediated noncanonical NF-kB signaling	33/3901	60/10891	1.81E-03	1.56E-02
DENND4C	Degradation of GLI1 by the proteasome	33/3901	60/10891	1.81E-03	1.56E-02
DENND4C	GLI3 is processed to GLI3R by the proteasome	33/3901	60/10891	1.81E-03	1.56E-02
DENND4C	Regulation of HSF1-mediated heat shock response	37/3901	69/10891	1.82E-03	1.56E-02
DENND4C	Ub-specific processing proteases	100/3901	220/10891	1.86E-03	1.59E-02
DENND4C	Endosomal Sorting Complex Required For Transport (ESCRT)	20/3901	32/10891	1.89E-03	1.60E-02
DENND4C	Signalling to RAS	14/3901	20/10891	1.95E-03	1.64E-02
DENND4C	Processing of Intronless Pre-mRNAs	14/3901	20/10891	1.95E-03	1.64E-02
DENND4C	NOTCH2 Activation and Transmission of Signal to the Nucleus	15/3901	22/10891	2.02E-03	1.67E-02
DENND4C	Defects in vitamin and cofactor metabolism	15/3901	22/10891	2.02E-03	1.67E-02
DENND4C	PINK1-PRKN Mediated Mitophagy	15/3901	22/10891	2.02E-03	1.67E-02
DENND4C	Estrogen-dependent nuclear events downstream of ESR-membrane signaling	16/3901	24/10891	2.05E-03	1.69E-02
DENND4C	Hh mutants are degraded by ERAD	31/3901	56/10891	2.15E-03	1.76E-02
DENND4C	Hedgehog ligand biogenesis	35/3901	65/10891	2.18E-03	1.77E-02
DENND4C	FBXL7 down-regulates AURKA during mitotic entry and in early mitosis	30/3901	54/10891	2.34E-03	1.89E-02
DENND4C	Signaling by MET	41/3901	79/10891	2.41E-03	1.93E-02
DENND4C	Synthesis of PIPs at the late endosome membrane	9/3901	11/10891	2.45E-03	1.93E-02
DENND4C	Establishment of Sister Chromatid Cohesion	9/3901	11/10891	2.45E-03	1.93E-02
DENND4C	Pexophagy	9/3901	11/10891	2.45E-03	1.93E-02
DENND4C	Regulation of BACH1 activity	9/3901	11/10891	2.45E-03	1.93E-02
DENND4C	Signaling by TGF-beta Receptor Complex	47/3901	93/10891	2.46E-03	1.93E-02
DENND4C	Dual Incision in GG-NER	24/3901	41/10891	2.47E-03	1.93E-02
DENND4C	GSK3B and BTRC:CUL1-mediated-degradation of NFE2L2	29/3901	52/10891	2.54E-03	1.97E-02
DENND4C	Signaling by the B Cell Receptor (BCR)	55/3901	112/10891	2.55E-03	1.97E-02

DENND4C	Death Receptor Signalling	67/3901	141/10891	2.68E-03	2.06E-02
DENND4C	Global Genome Nucleotide Excision Repair (GG-NER)	43/3901	84/10891	2.69E-03	2.06E-02
DENND4C	Regulation of activated PAK-2p34 by proteasome mediated degradation	28/3901	50/10891	2.77E-03	2.11E-02
DENND4C	Programmed Cell Death	95/3901	210/10891	2.84E-03	2.14E-02
DENND4C	Metabolism of polyamines	32/3901	59/10891	2.85E-03	2.14E-02
DENND4C	Hh mutants abrogate ligand secretion	32/3901	59/10891	2.85E-03	2.14E-02
DENND4C	Transport of Mature mRNA derived from an Intron-Containing Transcript	39/3901	75/10891	2.90E-03	2.17E-02
DENND4C	IRS-mediated signalling	27/3901	48/10891	3.00E-03	2.21E-02
DENND4C	NOTCH1 Intracellular Domain Regulates Transcription	27/3901	48/10891	3.00E-03	2.21E-02
DENND4C	Regulation of TNFR1 signaling	21/3901	35/10891	3.00E-03	2.21E-02
DENND4C	Downregulation of ERBB2:ERBB3 signaling	10/3901	13/10891	3.05E-03	2.21E-02
DENND4C	p75NTR recruits signalling complexes	10/3901	13/10891	3.05E-03	2.21E-02
DENND4C	IRF3-mediated induction of type I IFN	10/3901	13/10891	3.05E-03	2.21E-02
DENND4C	Mitotic Telophase/Cytokinesis	10/3901	13/10891	3.05E-03	2.21E-02
DENND4C	Degradation of DVL	31/3901	57/10891	3.11E-03	2.22E-02
DENND4C	Translation of Structural Proteins	31/3901	57/10891	3.11E-03	2.22E-02
DENND4C	Late SARS-CoV-2 Infection Events	31/3901	57/10891	3.11E-03	2.22E-02
DENND4C	HIV elongation arrest and recovery	20/3901	33/10891	3.19E-03	2.23E-02
DENND4C	Pausing and recovery of HIV elongation	20/3901	33/10891	3.19E-03	2.23E-02
DENND4C	Signaling by NOTCH2	20/3901	33/10891	3.19E-03	2.23E-02
DENND4C	Cargo concentration in the ER	20/3901	33/10891	3.19E-03	2.23E-02
DENND4C	Pausing and recovery of Tat-mediated HIV elongation	19/3901	31/10891	3.36E-03	2.33E-02
DENND4C	Tat-mediated HIV elongation arrest and recovery	19/3901	31/10891	3.36E-03	2.33E-02
DENND4C	Degradation of AXIN	30/3901	55/10891	3.40E-03	2.35E-02
DENND4C	Signaling by NOTCH1 HD Domain Mutants in Cancer	11/3901	15/10891	3.47E-03	2.36E-02
DENND4C	Constitutive Signaling by NOTCH1 HD Domain Mutants	11/3901	15/10891	3.47E-03	2.36E-02
DENND4C	Regulation of innate immune responses to cytosolic DNA	11/3901	15/10891	3.47E-03	2.36E-02
DENND4C	Orc1 removal from chromatin	37/3901	71/10891	3.50E-03	2.36E-02
DENND4C	TNF signaling	25/3901	44/10891	3.53E-03	2.36E-02
DENND4C	Downstream signal transduction	18/3901	29/10891	3.53E-03	2.36E-02
DENND4C	Energy dependent regulation of mTOR by LKB1-AMPK	18/3901	29/10891	3.53E-03	2.36E-02
DENND4C	Processing of Capped Intronless Pre-mRNA	18/3901	29/10891	3.53E-03	2.36E-02
DENND4C	ABC transporter disorders	40/3901	78/10891	3.58E-03	2.37E-02
DENND4C	Translesion synthesis by POLK	12/3901	17/10891	3.74E-03	2.46E-02

DENND4C	Signaling by NTRK3 (TRKC)	12/3901	17/10891	3.74E-03	2.46E-02
DENND4C	Constitutive Signaling by AKT1 E17K in Cancer	16/3901	25/10891	3.81E-03	2.50E-02
DENND4C	Transcriptional regulation by RUNX2	58/3901	121/10891	3.93E-03	2.56E-02
DENND4C	Cyclin E associated events during G1/S transition	42/3901	83/10891	3.94E-03	2.56E-02
DENND4C	Degradation of GLI2 by the proteasome	32/3901	60/10891	4.03E-03	2.61E-02
DENND4C	Transcriptional activity of SMAD2/SMAD3:SMAD4 heterotrimer	28/3901	51/10891	4.06E-03	2.62E-02
DENND4C	Activation of NF-kappaB in B cells	35/3901	67/10891	4.23E-03	2.71E-02
DENND4C	G1/S Transition	62/3901	131/10891	4.24E-03	2.71E-02
DENND4C	Regulation of lipid metabolism by PPARalpha	57/3901	119/10891	4.32E-03	2.74E-02
DENND4C	RHOC GTPase cycle	38/3901	74/10891	4.32E-03	2.74E-02
DENND4C	Signaling by ERBB4	31/3901	58/10891	4.42E-03	2.79E-02
DENND4C	Signaling by NOTCH3	27/3901	49/10891	4.43E-03	2.79E-02
DENND4C	The citric acid (TCA) cycle and respiratory electron transport	81/3901	178/10891	4.60E-03	2.88E-02
DENND4C	FOXO-mediated transcription	34/3901	65/10891	4.65E-03	2.90E-02
DENND4C	PPARA activates gene expression	56/3901	117/10891	4.74E-03	2.94E-02
DENND4C	Fc epsilon receptor (FCERI) signaling	63/3901	134/10891	4.78E-03	2.96E-02
DENND4C	G2/M Transition	88/3901	196/10891	5.11E-03	3.15E-02
DENND4C	SUMOylation of DNA replication proteins	25/3901	45/10891	5.27E-03	3.24E-02
DENND4C	Insulin receptor signalling cascade	29/3901	54/10891	5.32E-03	3.25E-02
DENND4C	Termination of translesion DNA synthesis	19/3901	32/10891	5.53E-03	3.37E-02
DENND4C	rRNA processing	91/3901	204/10891	5.56E-03	3.38E-02
DENND4C	CLEC7A (Dectin-1) signaling	49/3901	101/10891	5.72E-03	3.46E-02
DENND4C	IRS-related events triggered by IGF1R	28/3901	52/10891	5.83E-03	3.51E-02
DENND4C	Downregulation of SMAD2/3:SMAD4 transcriptional activity	18/3901	30/10891	5.90E-03	3.54E-02
DENND4C	RAC3 GTPase cycle	46/3901	94/10891	5.95E-03	3.56E-02
DENND4C	Signaling by FGFR4	23/3901	41/10891	6.26E-03	3.72E-02
DENND4C	Signaling by ERBB2	27/3901	50/10891	6.39E-03	3.77E-02
DENND4C	Signaling by FGFR1	27/3901	50/10891	6.39E-03	3.77E-02
DENND4C	RAC1 GTPase cycle	83/3901	185/10891	6.56E-03	3.86E-02
DENND4C	Insulin receptor recycling	16/3901	26/10891	6.63E-03	3.88E-02
DENND4C	Frs2-mediated activation	9/3901	12/10891	6.66E-03	3.88E-02
DENND4C	Diseases of glycosylation	66/3901	143/10891	6.69E-03	3.88E-02
DENND4C	Synthesis of DNA	57/3901	121/10891	6.70E-03	3.88E-02
DENND4C	Cyclin A:Cdk2-associated events at S phase entry	42/3901	85/10891	6.77E-03	3.91E-02
DENND4C	mTORC1-mediated signalling	15/3901	24/10891	6.97E-03	4.00E-02
DENND4C	Mitotic G2-G2/M phases	88/3901	198/10891	7.12E-03	4.00E-02
DENND4C	Membrane binding and targetting of GAG proteins	10/3901	14/10891	7.25E-03	4.00E-02
DENND4C	Synthesis And Processing Of GAG, GAGPOL Polyproteins	10/3901	14/10891	7.25E-03	4.00E-02

DENND4C	Synthesis of IP2, IP, and Ins in the cytosol	10/3901	14/10891	7.25E-03	4.00E-02
DENND4C	Glycogen synthesis	10/3901	14/10891	7.25E-03	4.00E-02
DENND4C	Regulation of TP53 Activity through Association with Co-factors	10/3901	14/10891	7.25E-03	4.00E-02
DENND4C	Chaperone Mediated Autophagy	14/3901	22/10891	7.26E-03	4.00E-02
DENND4C	Mitotic Anaphase	103/3901	236/10891	7.34E-03	4.00E-02
DENND4C	Maturation of spike protein	21/3901	37/10891	7.40E-03	4.00E-02
DENND4C	Regulation of RUNX3 expression and activity	29/3901	55/10891	7.45E-03	4.00E-02
DENND4C	Signaling by ALK in cancer	29/3901	55/10891	7.45E-03	4.00E-02
DENND4C	Signaling by ALK fusions and activated point mutants	29/3901	55/10891	7.45E-03	4.00E-02
DENND4C	Signaling by Hippo	13/3901	20/10891	7.47E-03	4.00E-02
DENND4C	NOTCH4 Intracellular Domain Regulates Transcription	13/3901	20/10891	7.47E-03	4.00E-02
DENND4C	Translesion synthesis by REV1	11/3901	16/10891	7.53E-03	4.00E-02
DENND4C	Spry regulation of FGF signaling	11/3901	16/10891	7.53E-03	4.00E-02
DENND4C	Assembly Of The HIV Virion	11/3901	16/10891	7.53E-03	4.00E-02
DENND4C	p75NTR signals via NF-kB	11/3901	16/10891	7.53E-03	4.00E-02
DENND4C	Regulation of pyruvate dehydrogenase (PDH) complex	11/3901	16/10891	7.53E-03	4.00E-02
DENND4C	Rap1 signalling	11/3901	16/10891	7.53E-03	4.00E-02
DENND4C	MAP3K8 (TPL2)-dependent MAPK1/3 activation	11/3901	16/10891	7.53E-03	4.00E-02
DENND4C	Signaling by ERBB2 ECD mutants	11/3901	16/10891	7.53E-03	4.00E-02
DENND4C	Pre-NOTCH Processing in Golgi	12/3901	18/10891	7.58E-03	4.00E-02
DENND4C	Gastrin-CREB signalling pathway via PKC and MAPK	12/3901	18/10891	7.58E-03	4.00E-02
DENND4C	Nonsense Mediated Decay (NMD) independent of the Exon Junction Complex (EJC)	46/3901	95/10891	7.60E-03	4.00E-02
DENND4C	Regulation of PLK1 Activity at G2/M Transition	43/3901	88/10891	7.92E-03	4.16E-02
DENND4C	N-glycan trimming in the ER and Calnexin/Calreticulin cycle	20/3901	35/10891	8.03E-03	4.18E-02
DENND4C	Negative regulators of DDX58/IFIH1 signaling	20/3901	35/10891	8.03E-03	4.18E-02
DENND4C	IGF1R signaling cascade	28/3901	53/10891	8.19E-03	4.25E-02
DENND4C	rRNA processing in the nucleus and cytosol	86/3901	194/10891	8.38E-03	4.32E-02
DENND4C	RHOG GTPase cycle	37/3901	74/10891	8.40E-03	4.32E-02
DENND4C	Major pathway of rRNA processing in the nucleolus and cytosol	82/3901	184/10891	8.42E-03	4.32E-02
DENND4C	SCF(Skp2)-mediated degradation of p27/p21	31/3901	60/10891	8.44E-03	4.32E-02
DENND4C	Mitotic Metaphase and Anaphase	103/3901	237/10891	8.49E-03	4.32E-02
DENND4C	Collagen biosynthesis and modifying enzymes	34/3901	67/10891	8.50E-03	4.32E-02
DENND4C	Regulation of ornithine decarboxylase (ODC)	27/3901	51/10891	9.01E-03	4.57E-02
DENND4C	EGFR downregulation	18/3901	31/10891	9.41E-03	4.70E-02
DENND4C	Activated NOTCH1 Transmits Signal to the	18/3901	31/10891	9.41E-03	4.70E-02

	Nucleus				
DENND4C	Disassembly of the destruction complex and recruitment of AXIN to the membrane	18/3901	31/10891	9.41E-03	4.70E-02
DENND4C	Cristae formation	18/3901	31/10891	9.41E-03	4.70E-02
DENND4C	Transferrin endocytosis and recycling	18/3901	31/10891	9.41E-03	4.70E-02
FAM89B	Influenza Viral RNA Transcription and Replication	84/4054	135/10891	2.78E-09	1.99E-06
FAM89B	Influenza Infection	94/4054	156/10891	3.60E-09	1.99E-06
FAM89B	rRNA processing in the nucleus and cytosol	112/4054	194/10891	4.01E-09	1.99E-06
FAM89B	Major pathway of rRNA processing in the nucleolus and cytosol	107/4054	184/10891	5.13E-09	1.99E-06
FAM89B	rRNA processing	115/4054	204/10891	1.63E-08	5.04E-06
FAM89B	Processing of Capped Intron-Containing Pre-mRNA	131/4054	246/10891	1.69E-07	4.38E-05
FAM89B	Late Phase of HIV Life Cycle	80/4054	136/10891	2.23E-07	4.92E-05
FAM89B	Eukaryotic Translation Termination	58/4054	93/10891	6.99E-07	1.35E-04
FAM89B	SUMOylation of DNA replication proteins	33/4054	45/10891	8.83E-07	1.52E-04
FAM89B	SUMOylation of ubiquitinylation proteins	29/4054	38/10891	1.02E-06	1.59E-04
FAM89B	HIV Life Cycle	84/4054	149/10891	1.36E-06	1.62E-04
FAM89B	Nonsense-Mediated Decay (NMD)	68/4054	115/10891	1.36E-06	1.62E-04
FAM89B	Nonsense Mediated Decay (NMD) enhanced by the Exon Junction Complex (EJC)	68/4054	115/10891	1.36E-06	1.62E-04
FAM89B	Nonsense Mediated Decay (NMD) independent of the Exon Junction Complex (EJC)	58/4054	95/10891	1.91E-06	2.12E-04
FAM89B	RNA Polymerase II Pre-transcription Events	51/4054	81/10891	2.16E-06	2.23E-04
FAM89B	SUMOylation of SUMOylation proteins	26/4054	34/10891	3.54E-06	3.43E-04
FAM89B	Selenocysteine synthesis	56/4054	93/10891	5.27E-06	4.80E-04
FAM89B	Viral mRNA Translation	54/4054	89/10891	5.61E-06	4.83E-04
FAM89B	rRNA modification in the nucleus and cytosol	40/4054	61/10891	6.29E-06	4.96E-04
FAM89B	SUMOylation of DNA damage response and repair proteins	48/4054	77/10891	6.40E-06	4.96E-04
FAM89B	Formation of RNA Pol II elongation complex	38/4054	58/10891	1.10E-05	7.17E-04
FAM89B	RNA Polymerase II Transcription Elongation	38/4054	58/10891	1.10E-05	7.17E-04
FAM89B	Selenoamino acid metabolism	67/4054	118/10891	1.13E-05	7.17E-04
FAM89B	Transcription of the HIV genome	44/4054	70/10891	1.14E-05	7.17E-04
FAM89B	mRNA Splicing	101/4054	193/10891	1.16E-05	7.17E-04
FAM89B	Viral Messenger RNA Synthesis	30/4054	43/10891	1.45E-05	8.66E-04
FAM89B	HIV Infection	117/4054	231/10891	1.80E-05	1.03E-03
FAM89B	Regulation of HSF1-mediated heat shock response	43/4054	69/10891	1.95E-05	1.08E-03
FAM89B	Formation of a pool of free 40S subunits	58/4054	101/10891	2.73E-05	1.41E-03
FAM89B	Response of EIF2AK4 (GCN2) to amino acid deficiency	58/4054	101/10891	2.73E-05	1.41E-03
FAM89B	GTP hydrolysis and joining of the 60S ribosomal	63/4054	112/10891	3.03E-05	1.51E-03

	subunit				
FAM89B	Export of Viral Ribonucleoproteins from Nucleus	24/4054	33/10891	3.59E-05	1.73E-03
FAM89B	Peptide chain elongation	52/4054	89/10891	3.68E-05	1.73E-03
FAM89B	SARS-CoV Infections	177/4054	376/10891	4.47E-05	2.04E-03
FAM89B	Translation	141/4054	291/10891	4.79E-05	2.12E-03
FAM89B	Transport of Mature mRNA Derived from an Intronless Transcript	28/4054	41/10891	5.19E-05	2.23E-03
FAM89B	mRNA Splicing - Major Pathway	95/4054	185/10891	5.47E-05	2.29E-03
FAM89B	Formation of HIV elongation complex in the absence of HIV Tat	30/4054	45/10891	5.69E-05	2.30E-03
FAM89B	Translation of Structural Proteins	36/4054	57/10891	6.09E-05	2.30E-03
FAM89B	Late SARS-CoV-2 Infection Events	36/4054	57/10891	6.09E-05	2.30E-03
FAM89B	Cellular response to starvation	82/4054	156/10891	6.10E-05	2.30E-03
FAM89B	SARS-CoV-2 Infection	139/4054	288/10891	6.85E-05	2.52E-03
FAM89B	Transport of the SLBP independent Mature mRNA	24/4054	34/10891	7.89E-05	2.78E-03
FAM89B	Eukaryotic Translation Elongation	53/4054	93/10891	7.91E-05	2.78E-03
FAM89B	Cellular response to heat stress	51/4054	89/10891	8.82E-05	3.04E-03
FAM89B	Transport of Mature mRNAs Derived from Intronless Transcripts	28/4054	42/10891	1.01E-04	3.33E-03
FAM89B	L13a-mediated translational silencing of Ceruloplasmin expression	61/4054	111/10891	1.01E-04	3.33E-03
FAM89B	Transport of Ribonucleoproteins into the Host Nucleus	22/4054	31/10891	1.39E-04	4.22E-03
FAM89B	NEP/NS2 Interacts with the Cellular Export Machinery	22/4054	31/10891	1.39E-04	4.22E-03
FAM89B	Regulation of Glucokinase by Glucokinase Regulatory Protein	22/4054	31/10891	1.39E-04	4.22E-03
FAM89B	Defective TPR may confer susceptibility towards thyroid papillary carcinoma (TPC)	22/4054	31/10891	1.39E-04	4.22E-03
FAM89B	Asparagine N-linked glycosylation	144/4054	304/10891	1.56E-04	4.65E-03
FAM89B	Transport of the SLBP Dependant Mature mRNA	24/4054	35/10891	1.63E-04	4.75E-03
FAM89B	Maturation of spike protein	25/4054	37/10891	1.71E-04	4.91E-03
FAM89B	HIV Transcription Elongation	28/4054	43/10891	1.86E-04	5.06E-03
FAM89B	Formation of HIV-1 elongation complex containing HIV-1 Tat	28/4054	43/10891	1.86E-04	5.06E-03
FAM89B	Tat-mediated elongation of the HIV-1 transcript	28/4054	43/10891	1.86E-04	5.06E-03
FAM89B	trans-Golgi Network Vesicle Budding	42/4054	72/10891	2.12E-04	5.65E-03
FAM89B	Metabolism of amino acids and derivatives	172/4054	374/10891	2.56E-04	6.71E-03
FAM89B	DNA Repair	156/4054	336/10891	2.83E-04	7.30E-03
FAM89B	SRP-dependent cotranslational protein targeting to membrane	60/4054	112/10891	2.92E-04	7.35E-03
FAM89B	Golgi Associated Vesicle Biogenesis	34/4054	56/10891	2.94E-04	7.35E-03

FAM89B	Eukaryotic Translation Initiation	63/4054	119/10891	3.22E-04	7.80E-03
FAM89B	Cap-dependent Translation Initiation	63/4054	119/10891	3.22E-04	7.80E-03
FAM89B	SUMO E3 ligases SUMOylate target proteins	90/4054	182/10891	4.57E-04	1.09E-02
FAM89B	Retrograde transport at the Trans-Golgi-Network	30/4054	49/10891	5.40E-04	1.16E-02
FAM89B	HIV Transcription Initiation	29/4054	47/10891	5.54E-04	1.16E-02
FAM89B	RNA Polymerase II HIV Promoter Escape	29/4054	47/10891	5.54E-04	1.16E-02
FAM89B	SUMOylation of RNA binding proteins	29/4054	47/10891	5.54E-04	1.16E-02
FAM89B	RNA Polymerase II Promoter Escape	29/4054	47/10891	5.54E-04	1.16E-02
FAM89B	RNA Polymerase II Transcription Pre-Initiation And Promoter Opening	29/4054	47/10891	5.54E-04	1.16E-02
FAM89B	RNA Polymerase II Transcription Initiation	29/4054	47/10891	5.54E-04	1.16E-02
FAM89B	RNA Polymerase II Transcription Initiation And Promoter Clearance	29/4054	47/10891	5.54E-04	1.16E-02
FAM89B	Nuclear import of Rev protein	22/4054	33/10891	5.62E-04	1.16E-02
FAM89B	Vpr-mediated nuclear import of PICs	22/4054	33/10891	5.62E-04	1.16E-02
FAM89B	SUMOylation	92/4054	188/10891	6.20E-04	1.26E-02
FAM89B	TP53 Regulates Transcription of DNA Repair Genes	36/4054	62/10891	6.63E-04	1.33E-02
FAM89B	Transport of Mature mRNA derived from an Intron-Containing Transcript	42/4054	75/10891	6.97E-04	1.38E-02
FAM89B	Transport of Mature Transcript to Cytoplasm	46/4054	84/10891	7.68E-04	1.51E-02
FAM89B	Nucleotide Excision Repair	58/4054	111/10891	8.40E-04	1.63E-02
FAM89B	mRNA Splicing - Minor Pathway	30/4054	50/10891	8.73E-04	1.67E-02
FAM89B	DNA Damage Bypass	29/4054	48/10891	9.06E-04	1.71E-02
FAM89B	NS1 Mediated Effects on Host Pathways	25/4054	40/10891	1.01E-03	1.86E-02
FAM89B	Interactions of Vpr with host cellular proteins	23/4054	36/10891	1.04E-03	1.86E-02
FAM89B	Interactions of Rev with host cellular proteins	23/4054	36/10891	1.04E-03	1.86E-02
FAM89B	Rev-mediated nuclear export of HIV RNA	22/4054	34/10891	1.04E-03	1.86E-02
FAM89B	ISG15 antiviral mechanism	40/4054	72/10891	1.14E-03	2.03E-02
FAM89B	snRNP Assembly	31/4054	53/10891	1.31E-03	2.27E-02
FAM89B	Metabolism of non-coding RNA	31/4054	53/10891	1.31E-03	2.27E-02
FAM89B	Dual incision in TC-NER	37/4054	66/10891	1.38E-03	2.36E-02
FAM89B	Mitotic Metaphase and Anaphase	111/4054	237/10891	1.39E-03	2.36E-02
FAM89B	Mitotic Anaphase	110/4054	236/10891	1.78E-03	2.89E-02
FAM89B	Nuclear Pore Complex (NPC) Disassembly	22/4054	35/10891	1.81E-03	2.89E-02
FAM89B	mRNA Capping	19/4054	29/10891	1.83E-03	2.89E-02
FAM89B	Formation of the Early Elongation Complex	21/4054	33/10891	1.84E-03	2.89E-02
FAM89B	Formation of the HIV-1 Early Elongation Complex	21/4054	33/10891	1.84E-03	2.89E-02
FAM89B	HIV elongation arrest and recovery	21/4054	33/10891	1.84E-03	2.89E-02
FAM89B	Pausing and recovery of HIV elongation	21/4054	33/10891	1.84E-03	2.89E-02
FAM89B	Antiviral mechanism by IFN-stimulated genes	43/4054	80/10891	1.84E-03	2.89E-02
FAM89B	Regulation of IFNG signaling	11/4054	14/10891	1.98E-03	3.06E-02
FAM89B	Nuclear Envelope Breakdown	30/4054	52/10891	2.10E-03	3.22E-02

FAM89B	Extracellular matrix organization	136/4054	300/10891	2.14E-03	3.25E-02
FAM89B	Resolution of Sister Chromatid Cohesion	63/4054	126/10891	2.19E-03	3.29E-02
FAM89B	Regulation of expression of SLITs and ROBOs	82/4054	171/10891	2.48E-03	3.69E-02
FAM89B	Mitotic Spindle Checkpoint	57/4054	113/10891	2.69E-03	3.97E-02
FAM89B	tRNA processing in the nucleus	32/4054	57/10891	2.76E-03	4.03E-02
FAM89B	SUMOylation of transcription factors	14/4054	20/10891	2.97E-03	4.26E-02
FAM89B	Processing of Intronless Pre-mRNAs	14/4054	20/10891	2.97E-03	4.26E-02
FAM89B	Resolution of D-loop Structures through Holliday Junction Intermediates	21/4054	34/10891	3.13E-03	4.42E-02
FAM89B	Citric acid cycle (TCA cycle)	15/4054	22/10891	3.14E-03	4.42E-02
FAM89B	Global Genome Nucleotide Excision Repair (GG-NER)	44/4054	84/10891	3.18E-03	4.44E-02
FAM89B	Impaired BRCA2 binding to PALB2	16/4054	24/10891	3.24E-03	4.48E-02
FAM89B	RNA Polymerase III Transcription Initiation From Type 1 Promoter	18/4054	28/10891	3.29E-03	4.52E-02
FAM89B	Establishment of Sister Chromatid Cohesion	9/4054	11/10891	3.33E-03	4.53E-02
FAM89B	tRNA processing	54/4054	107/10891	3.38E-03	4.56E-02
FNIP2	Asparagine N-linked glycosylation	115/2475	304/10891	1.27E-09	1.93E-06
FNIP2	HIV Infection	86/2475	231/10891	3.49E-07	1.97E-04
FNIP2	rRNA modification in the nucleus and cytosol	32/2475	61/10891	3.89E-07	1.97E-04
FNIP2	Signaling by Interleukins	153/2475	473/10891	5.82E-07	2.21E-04
FNIP2	Antiviral mechanism by IFN-stimulated genes	38/2475	80/10891	8.92E-07	2.71E-04
FNIP2	ISG15 antiviral mechanism	35/2475	72/10891	1.21E-06	2.97E-04
FNIP2	ER to Golgi Anterograde Transport	61/2475	154/10891	1.67E-06	2.97E-04
FNIP2	Transport to the Golgi and subsequent modification	70/2475	185/10891	2.16E-06	2.97E-04
FNIP2	MyD88-independent TLR4 cascade	47/2475	110/10891	2.21E-06	2.97E-04
FNIP2	TRIF(TICAM1)-mediated TLR4 signaling	47/2475	110/10891	2.21E-06	2.97E-04
FNIP2	HIV Life Cycle	59/2475	149/10891	2.50E-06	2.97E-04
FNIP2	Late Phase of HIV Life Cycle	55/2475	136/10891	2.53E-06	2.97E-04
FNIP2	COPII-mediated vesicle transport	33/2475	68/10891	2.54E-06	2.97E-04
FNIP2	Toll Like Receptor 3 (TLR3) Cascade	45/2475	105/10891	3.27E-06	3.55E-04
FNIP2	Transport of Mature mRNA Derived from an Intronless Transcript	23/2475	41/10891	3.76E-06	3.80E-04
FNIP2	Transport of the SLBP independent Mature mRNA	20/2475	34/10891	6.05E-06	5.75E-04
FNIP2	Transport of Mature mRNAs Derived from Intronless Transcripts	23/2475	42/10891	6.52E-06	5.82E-04
FNIP2	Processing of Capped Intron-Containing Pre-mRNA	86/2475	246/10891	6.89E-06	5.82E-04
FNIP2	Intra-Golgi and retrograde Golgi-to-ER traffic	73/2475	202/10891	9.07E-06	7.25E-04
FNIP2	Transport of the SLBP Dependant Mature mRNA	20/2475	35/10891	1.11E-05	8.44E-04
FNIP2	Regulation of HSF1-mediated heat shock	32/2475	69/10891	1.20E-05	8.67E-04

	response				
FNIP2	Formation of RNA Pol II elongation complex	28/2475	58/10891	1.64E-05	1.08E-03
FNIP2	RNA Polymerase II Transcription Elongation	28/2475	58/10891	1.64E-05	1.08E-03
FNIP2	Toll-like Receptor Cascades	62/2475	168/10891	2.01E-05	1.24E-03
FNIP2	Death Receptor Signalling	54/2475	141/10891	2.04E-05	1.24E-03
FNIP2	Transport of Ribonucleoproteins into the Host Nucleus	18/2475	31/10891	2.28E-05	1.29E-03
FNIP2	Interleukin-1 signaling	46/2475	115/10891	2.28E-05	1.29E-03
FNIP2	TRAF6 mediated induction of NFkB and MAP kinases upon TLR7/8 or 9 activation	42/2475	103/10891	2.98E-05	1.50E-03
FNIP2	Interleukin-1 family signaling	57/2475	153/10891	3.12E-05	1.50E-03
FNIP2	RNA Polymerase II Pre-transcription Events	35/2475	81/10891	3.18E-05	1.50E-03
FNIP2	SARS-CoV Infections	119/2475	376/10891	3.21E-05	1.50E-03
FNIP2	Toll Like Receptor 10 (TLR10) Cascade	40/2475	97/10891	3.36E-05	1.50E-03
FNIP2	Toll Like Receptor 5 (TLR5) Cascade	40/2475	97/10891	3.36E-05	1.50E-03
FNIP2	MyD88 cascade initiated on plasma membrane	40/2475	97/10891	3.36E-05	1.50E-03
FNIP2	Interleukin-12 family signaling	27/2475	57/10891	3.56E-05	1.54E-03
FNIP2	NS1 Mediated Effects on Host Pathways	21/2475	40/10891	3.85E-05	1.60E-03
FNIP2	MyD88 dependent cascade initiated on endosome	42/2475	104/10891	3.91E-05	1.60E-03
FNIP2	Viral Messenger RNA Synthesis	22/2475	43/10891	4.25E-05	1.70E-03
FNIP2	Toll Like Receptor 9 (TLR9) Cascade	43/2475	108/10891	4.75E-05	1.84E-03
FNIP2	Retrograde transport at the Trans-Golgi-Network	24/2475	49/10891	4.85E-05	1.84E-03
FNIP2	Toll Like Receptor 7/8 (TLR7/8) Cascade	42/2475	105/10891	5.10E-05	1.87E-03
FNIP2	Regulation of cholesterol biosynthesis by SREBP (SREBF)	26/2475	55/10891	5.16E-05	1.87E-03
FNIP2	Toll Like Receptor 4 (TLR4) Cascade	54/2475	146/10891	6.26E-05	2.21E-03
FNIP2	Interleukin-12 signaling	23/2475	47/10891	7.07E-05	2.30E-03
FNIP2	Nuclear import of Rev protein	18/2475	33/10891	7.12E-05	2.30E-03
FNIP2	Vpr-mediated nuclear import of PICs	18/2475	33/10891	7.12E-05	2.30E-03
FNIP2	Cargo concentration in the ER	18/2475	33/10891	7.12E-05	2.30E-03
FNIP2	snRNP Assembly	25/2475	53/10891	7.49E-05	2.30E-03
FNIP2	Metabolism of non-coding RNA	25/2475	53/10891	7.49E-05	2.30E-03
FNIP2	Golgi Associated Vesicle Biogenesis	26/2475	56/10891	7.57E-05	2.30E-03
FNIP2	Interactions of Vpr with host cellular proteins	19/2475	36/10891	8.17E-05	2.43E-03
FNIP2	Rab regulation of trafficking	47/2475	124/10891	9.21E-05	2.69E-03
FNIP2	Formation of HIV elongation complex in the absence of HIV Tat	22/2475	45/10891	1.03E-04	2.91E-03
FNIP2	NEP/NS2 Interacts with the Cellular Export Machinery	17/2475	31/10891	1.03E-04	2.91E-03
FNIP2	Translation of Structural Proteins	26/2475	57/10891	1.09E-04	2.97E-03
FNIP2	Late SARS-CoV-2 Infection Events	26/2475	57/10891	1.09E-04	2.97E-03
FNIP2	Diseases of signal transduction by growth factor	131/2475	433/10891	1.33E-04	3.54E-03

	receptors and second messengers				
FNIP2	Regulation of IFNG signaling	10/2475	14/10891	1.45E-04	3.74E-03
FNIP2	HIV Transcription Elongation	21/2475	43/10891	1.50E-04	3.74E-03
FNIP2	Formation of HIV-1 elongation complex containing HIV-1 Tat	21/2475	43/10891	1.50E-04	3.74E-03
FNIP2	Tat-mediated elongation of the HIV-1 transcript	21/2475	43/10891	1.50E-04	3.74E-03
FNIP2	IRE1alpha activates chaperones	24/2475	52/10891	1.59E-04	3.83E-03
FNIP2	MyD88:MAL(TIRAP) cascade initiated on plasma membrane	43/2475	113/10891	1.62E-04	3.83E-03
FNIP2	Toll Like Receptor TLR6:TLR2 Cascade	43/2475	113/10891	1.62E-04	3.83E-03
FNIP2	Host Interactions of HIV factors	48/2475	130/10891	1.64E-04	3.83E-03
FNIP2	RAB geranylgeranylation	28/2475	65/10891	2.04E-04	4.69E-03
FNIP2	Gene and protein expression by JAK-STAT signaling after Interleukin-12 stimulation	19/2475	38/10891	2.09E-04	4.73E-03
FNIP2	XBP1(S) activates chaperone genes	23/2475	50/10891	2.31E-04	5.15E-03
FNIP2	trans-Golgi Network Vesicle Budding	30/2475	72/10891	2.51E-04	5.52E-03
FNIP2	Export of Viral Ribonucleoproteins from Nucleus	17/2475	33/10891	2.84E-04	6.15E-03
FNIP2	mRNA Splicing	65/2475	193/10891	2.98E-04	6.38E-03
FNIP2	Interactions of Rev with host cellular proteins	18/2475	36/10891	3.06E-04	6.45E-03
FNIP2	Toll Like Receptor TLR1:TLR2 Cascade	43/2475	116/10891	3.18E-04	6.53E-03
FNIP2	Toll Like Receptor 2 (TLR2) Cascade	43/2475	116/10891	3.18E-04	6.53E-03
FNIP2	Signaling by CTNNB1 phospho-site mutants	10/2475	15/10891	3.45E-04	6.56E-03
FNIP2	Signaling by GSK3beta mutants	10/2475	15/10891	3.45E-04	6.56E-03
FNIP2	CTNNB1 S33 mutants aren't phosphorylated	10/2475	15/10891	3.45E-04	6.56E-03
FNIP2	CTNNB1 S37 mutants aren't phosphorylated	10/2475	15/10891	3.45E-04	6.56E-03
FNIP2	CTNNB1 S45 mutants aren't phosphorylated	10/2475	15/10891	3.45E-04	6.56E-03
FNIP2	CTNNB1 T41 mutants aren't phosphorylated	10/2475	15/10891	3.45E-04	6.56E-03
FNIP2	Transcription of the HIV genome	29/2475	70/10891	3.55E-04	6.66E-03
FNIP2	Neutrophil degranulation	141/2475	482/10891	3.96E-04	7.25E-03
FNIP2	RAB GEFs exchange GTP for GDP on RABs	35/2475	90/10891	3.96E-04	7.25E-03
FNIP2	Regulation of Glucokinase by Glucokinase Regulatory Protein	16/2475	31/10891	4.16E-04	7.43E-03
FNIP2	Defective TPR may confer susceptibility towards thyroid papillary carcinoma (TPC)	16/2475	31/10891	4.16E-04	7.43E-03
FNIP2	Rev-mediated nuclear export of HIV RNA	17/2475	34/10891	4.48E-04	7.91E-03
FNIP2	Maturation of spike protein	18/2475	37/10891	4.70E-04	8.13E-03
FNIP2	mRNA Splicing - Major Pathway	62/2475	185/10891	4.71E-04	8.13E-03
FNIP2	RHO GTPase cycle	132/2475	449/10891	4.81E-04	8.21E-03
FNIP2	SARS-CoV-2 activates/modulates innate and adaptive immune responses	45/2475	126/10891	6.02E-04	1.02E-02
FNIP2	mRNA Capping	15/2475	29/10891	6.10E-04	1.02E-02
FNIP2	Disorders of transmembrane transporters	59/2475	176/10891	6.33E-04	1.04E-02
FNIP2	GPVI-mediated activation cascade	17/2475	35/10891	6.88E-04	1.10E-02

FNIP2	RNA Polymerase I Transcription Initiation	21/2475	47/10891	6.91E-04	1.10E-02
FNIP2	Cellular response to heat stress	34/2475	89/10891	6.96E-04	1.10E-02
FNIP2	TNF signaling	20/2475	44/10891	7.04E-04	1.10E-02
FNIP2	SUMOylation of ubiquitinylation proteins	18/2475	38/10891	7.05E-04	1.10E-02
FNIP2	SARS-CoV-2 Infection	89/2475	288/10891	7.27E-04	1.13E-02
FNIP2	Signaling by CSF3 (G-CSF)	15/2475	30/10891	9.66E-04	1.44E-02
FNIP2	SUMOylation of DNA replication proteins	20/2475	45/10891	9.99E-04	1.44E-02
FNIP2	Formation of the Early Elongation Complex	16/2475	33/10891	1.01E-03	1.44E-02
FNIP2	Formation of the HIV-1 Early Elongation Complex	16/2475	33/10891	1.01E-03	1.44E-02
FNIP2	Amino acid transport across the plasma membrane	16/2475	33/10891	1.01E-03	1.44E-02
FNIP2	Signaling by AXIN mutants	9/2475	14/10891	1.03E-03	1.44E-02
FNIP2	Signaling by APC mutants	9/2475	14/10891	1.03E-03	1.44E-02
FNIP2	Signaling by AMER1 mutants	9/2475	14/10891	1.03E-03	1.44E-02
FNIP2	APC truncation mutants have impaired AXIN binding	9/2475	14/10891	1.03E-03	1.44E-02
FNIP2	AXIN missense mutants destabilize the destruction complex	9/2475	14/10891	1.03E-03	1.44E-02
FNIP2	Truncations of AMER1 destabilize the destruction complex	9/2475	14/10891	1.03E-03	1.44E-02
FNIP2	RHOA GTPase cycle	50/2475	147/10891	1.10E-03	1.52E-02
FNIP2	SLC transporter disorders	36/2475	98/10891	1.14E-03	1.56E-02
FNIP2	Basigin interactions	13/2475	25/10891	1.32E-03	1.79E-02
FNIP2	TP53 Regulates Transcription of DNA Repair Genes	25/2475	62/10891	1.41E-03	1.87E-02
FNIP2	Beta-catenin phosphorylation cascade	10/2475	17/10891	1.42E-03	1.87E-02
FNIP2	Platelet sensitization by LDL	10/2475	17/10891	1.42E-03	1.87E-02
FNIP2	ALK mutants bind TKIs	8/2475	12/10891	1.43E-03	1.87E-02
FNIP2	Interleukin-17 signaling	28/2475	72/10891	1.45E-03	1.89E-02
FNIP2	SUMOylation of SUMOylation proteins	16/2475	34/10891	1.51E-03	1.94E-02
FNIP2	Nucleotide Excision Repair	39/2475	111/10891	1.91E-03	2.31E-02
FNIP2	HIV Transcription Initiation	20/2475	47/10891	1.92E-03	2.31E-02
FNIP2	RNA Polymerase II HIV Promoter Escape	20/2475	47/10891	1.92E-03	2.31E-02
FNIP2	RNA Polymerase II Promoter Escape	20/2475	47/10891	1.92E-03	2.31E-02
FNIP2	RNA Polymerase II Transcription Pre-Initiation And Promoter Opening	20/2475	47/10891	1.92E-03	2.31E-02
FNIP2	RNA Polymerase II Transcription Initiation	20/2475	47/10891	1.92E-03	2.31E-02
FNIP2	RNA Polymerase II Transcription Initiation And Promoter Clearance	20/2475	47/10891	1.92E-03	2.31E-02
FNIP2	Metabolism of steroids	51/2475	154/10891	1.92E-03	2.31E-02
FNIP2	Intra-Golgi traffic	19/2475	44/10891	2.01E-03	2.40E-02
FNIP2	Diseases associated with glycosylation precursor biosynthesis	9/2475	15/10891	2.06E-03	2.42E-02

FNIP2	Signaling by NTRK1 (TRKA)	40/2475	115/10891	2.08E-03	2.42E-02
FNIP2	Unfolded Protein Response (UPR)	34/2475	94/10891	2.09E-03	2.42E-02
FNIP2	Calnexin/calreticulin cycle	13/2475	26/10891	2.10E-03	2.42E-02
FNIP2	COPI-mediated anterograde transport	36/2475	101/10891	2.11E-03	2.42E-02
FNIP2	tRNA processing in the nucleus	23/2475	57/10891	2.13E-03	2.43E-02
FNIP2	Nuclear Pore Complex (NPC) Disassembly	16/2475	35/10891	2.20E-03	2.49E-02
FNIP2	FCERI mediated MAPK activation	15/2475	32/10891	2.21E-03	2.49E-02
FNIP2	RHOC GTPase cycle	28/2475	74/10891	2.35E-03	2.62E-02
FNIP2	MAP kinase activation	25/2475	64/10891	2.38E-03	2.64E-02
FNIP2	Interferon Signaling	63/2475	200/10891	2.47E-03	2.72E-02
FNIP2	Signaling by NTRKs	45/2475	134/10891	2.53E-03	2.75E-02
FNIP2	Fc epsilon receptor (FCERI) signaling	45/2475	134/10891	2.53E-03	2.75E-02
FNIP2	Glycolysis	27/2475	71/10891	2.56E-03	2.76E-02
FNIP2	TAK1-dependent IKK and NF-kappa-B activation	19/2475	45/10891	2.75E-03	2.94E-02
FNIP2	Transcriptional Regulation by TP53	105/2475	362/10891	2.81E-03	2.98E-02
FNIP2	Activation of gene expression by SREBF (SREBP)	18/2475	42/10891	2.89E-03	3.03E-02
FNIP2	tRNA Aminoacylation	18/2475	42/10891	2.89E-03	3.03E-02
FNIP2	RHOJ GTPase cycle	22/2475	55/10891	3.00E-03	3.08E-02
FNIP2	Signaling by ALK in cancer	22/2475	55/10891	3.00E-03	3.08E-02
FNIP2	Signaling by ALK fusions and activated point mutants	22/2475	55/10891	3.00E-03	3.08E-02
FNIP2	FCERI mediated NF-kB activation	30/2475	82/10891	3.05E-03	3.11E-02
FNIP2	Cytosolic tRNA aminoacylation	12/2475	24/10891	3.10E-03	3.12E-02
FNIP2	Growth hormone receptor signaling	12/2475	24/10891	3.10E-03	3.12E-02
FNIP2	RNA Pol II CTD phosphorylation and interaction with CE during HIV infection	13/2475	27/10891	3.22E-03	3.18E-02
FNIP2	RNA Pol II CTD phosphorylation and interaction with CE	13/2475	27/10891	3.22E-03	3.18E-02
FNIP2	Nuclear Envelope Breakdown	21/2475	52/10891	3.23E-03	3.18E-02
FNIP2	TBC/RABGAPs	19/2475	46/10891	3.70E-03	3.62E-02
FNIP2	p75 NTR receptor-mediated signalling	34/2475	97/10891	3.76E-03	3.66E-02
FNIP2	Golgi-to-ER retrograde transport	44/2475	133/10891	3.86E-03	3.73E-02
FNIP2	Interleukin-6 signaling	7/2475	11/10891	4.26E-03	4.10E-02
FNIP2	SUMOylation of DNA damage response and repair proteins	28/2475	77/10891	4.54E-03	4.34E-02
FNIP2	Sema4D in semaphorin signaling	12/2475	25/10891	4.75E-03	4.45E-02
FNIP2	Signaling by Erythropoietin	12/2475	25/10891	4.75E-03	4.45E-02
FNIP2	Inactivation of CSF3 (G-CSF) signaling	12/2475	25/10891	4.75E-03	4.45E-02
FNIP2	SUMOylation of RNA binding proteins	19/2475	47/10891	4.91E-03	4.57E-02
FNIP2	RNA polymerase II transcribes snRNA genes	27/2475	74/10891	5.00E-03	4.63E-02
FNIP2	Neddylation	72/2475	240/10891	5.05E-03	4.65E-02
FNIP2	Potential therapeutics for SARS	34/2475	99/10891	5.42E-03	4.96E-02
FNIP2	Formation of TC-NER Pre-Incision Complex	21/2475	54/10891	5.45E-03	4.96E-02

MAMSTR	Signaling by Interleukins	169/2700	473/10891	3.83E-08	5.87E-05
MAMSTR	rRNA modification in the nucleus and cytosol	31/2700	61/10891	1.02E-05	7.79E-03
MAMSTR	Gene and protein expression by JAK-STAT signaling after Interleukin-12 stimulation	21/2700	38/10891	5.56E-05	2.80E-02
MAMSTR	Asparagine N-linked glycosylation	105/2700	304/10891	7.30E-05	2.80E-02
METRNL	Neutrophil degranulation	200/3019	482/10891	1.94E-11	2.99E-08
METRNL	RHO GTPase cycle	181/3019	449/10891	2.78E-09	2.14E-06
METRNL	Phosphorylation of CD3 and TCR zeta chains	17/3019	22/10891	1.90E-06	9.76E-04
METRNL	Immunoregulatory interactions between a Lymphoid and a non-Lymphoid cell	62/3019	134/10891	3.15E-06	1.21E-03
METRNL	Platelet activation, signaling and aggregation	105/3019	263/10891	1.01E-05	3.11E-03
METRNL	PD-1 signaling	16/3019	23/10891	3.55E-05	6.37E-03
METRNL	Antigen activates B Cell Receptor (BCR) leading to generation of second messengers	20/3019	32/10891	4.07E-05	6.37E-03
METRNL	Translocation of ZAP-70 to Immunological synapse	14/3019	19/10891	4.07E-05	6.37E-03
METRNL	Signaling by Interleukins	170/3019	473/10891	4.09E-05	6.37E-03
METRNL	Costimulation by the CD28 family	35/3019	69/10891	4.14E-05	6.37E-03
METRNL	RAC1 GTPase cycle	76/3019	185/10891	5.38E-05	7.53E-03
METRNL	Interleukin-10 signaling	26/3019	47/10891	6.06E-05	7.77E-03
METRNL	Toll-like Receptor Cascades	69/3019	168/10891	1.17E-04	1.39E-02
METRNL	Toll Like Receptor 4 (TLR4) Cascade	61/3019	146/10891	1.62E-04	1.64E-02
METRNL	Interferon gamma signaling	42/3019	92/10891	1.70E-04	1.64E-02
METRNL	CDC42 GTPase cycle	64/3019	155/10891	1.70E-04	1.64E-02
METRNL	RHO GTPases Activate NADPH Oxidases	15/3019	24/10891	3.84E-04	3.30E-02
METRNL	RHOA GTPase cycle	60/3019	147/10891	3.86E-04	3.30E-02
METRNL	Response to elevated platelet cytosolic Ca2+	55/3019	134/10891	5.63E-04	4.22E-02
METRNL	Cell surface interactions at the vascular wall	56/3019	137/10891	5.69E-04	4.22E-02
METRNL	Interleukin receptor SHC signaling	16/3019	27/10891	5.76E-04	4.22E-02
METRNL	RND1 GTPase cycle	22/3019	42/10891	6.17E-04	4.31E-02
METRNL	Smooth Muscle Contraction	23/3019	45/10891	7.34E-04	4.80E-02
METRNL	Regulation of KIT signaling	11/3019	16/10891	7.48E-04	4.80E-02
MYL2	Striated Muscle Contraction	19/64	36/10891	1.71E-34	2.16E-32
MYL2	Muscle contraction	26/64	205/10891	8.93E-29	5.63E-27
MYL2	Formation of the cornified envelope	14/64	130/10891	1.69E-14	7.09E-13
MYL2	Keratinization	14/64	214/10891	1.68E-11	5.30E-10
MYL2	Ion homeostasis	4/64	56/10891	3.17E-04	7.98E-03
MYL2	Cardiac conduction	5/64	132/10891	1.04E-03	2.19E-02
MYL2	Apoptotic cleavage of cell adhesion proteins	2/64	11/10891	1.81E-03	2.85E-02
MYL2	Type I hemidesmosome assembly	2/64	11/10891	1.81E-03	2.85E-02
MYL2	Smooth Muscle Contraction	3/64	45/10891	2.30E-03	3.22E-02
NAA38	Amplification of signal from the kinetochores	46/2836	96/10891	3.12E-06	2.40E-03
NAA38	Amplification of signal from unattached	46/2836	96/10891	3.12E-06	2.40E-03

	kinetochores via a MAD2 inhibitory signal				
NAA38	RND1 GTPase cycle	24/2836	42/10891	1.87E-05	9.56E-03
NAA38	EML4 and NUDC in mitotic spindle formation	51/2836	117/10891	2.68E-05	1.03E-02
NAA38	Resolution of Sister Chromatid Cohesion	53/2836	126/10891	6.13E-05	1.88E-02
NAA38	RHO GTPases Activate Formins	57/2836	140/10891	9.82E-05	2.20E-02
NAA38	Mitotic Spindle Checkpoint	48/2836	113/10891	1.00E-04	2.20E-02
NAA38	Protein-protein interactions at synapses	38/2836	86/10891	1.95E-04	3.41E-02
NAA38	Neurexins and neuroligins	27/2836	55/10891	2.00E-04	3.41E-02
NDFIP1	Intra-Golgi and retrograde Golgi-to-ER traffic	63/1736	202/10891	3.82E-08	5.69E-05
NDFIP1	Asparagine N-linked glycosylation	83/1736	304/10891	2.29E-07	1.33E-04
NDFIP1	Transport to the Golgi and subsequent modification	57/1736	185/10891	2.67E-07	1.33E-04
NDFIP1	Golgi-to-ER retrograde transport	43/1736	133/10891	1.89E-06	6.17E-04
NDFIP1	Neurotransmitter receptors and postsynaptic signal transmission	59/1736	205/10891	2.07E-06	6.17E-04
NDFIP1	Transmission across Chemical Synapses	72/1736	270/10891	3.66E-06	9.09E-04
NDFIP1	Rab regulation of trafficking	40/1736	124/10891	4.55E-06	9.68E-04
NDFIP1	TBC/RABGAPs	20/1736	46/10891	8.47E-06	1.54E-03
NDFIP1	ER to Golgi Anterograde Transport	46/1736	154/10891	9.32E-06	1.54E-03
NDFIP1	G2/M Transition	55/1736	196/10891	1.04E-05	1.55E-03
NDFIP1	Neurotoxicity of clostridium toxins	8/1736	10/10891	1.37E-05	1.78E-03
NDFIP1	Mitotic G2-G2/M phases	55/1736	198/10891	1.43E-05	1.78E-03
NDFIP1	trans-Golgi Network Vesicle Budding	26/1736	72/10891	2.35E-05	2.57E-03
NDFIP1	Uptake and actions of bacterial toxins	15/1736	31/10891	2.42E-05	2.57E-03
NDFIP1	Clathrin-mediated endocytosis	43/1736	146/10891	2.64E-05	2.60E-03
NDFIP1	Translation of Structural Proteins	22/1736	57/10891	3.00E-05	2.60E-03
NDFIP1	Late SARS-CoV-2 Infection Events	22/1736	57/10891	3.00E-05	2.60E-03
NDFIP1	RAB geranylgeranylation	24/1736	65/10891	3.14E-05	2.60E-03
NDFIP1	Neutrophil degranulation	110/1736	482/10891	3.46E-05	2.72E-03
NDFIP1	Cargo concentration in the ER	15/1736	33/10891	6.07E-05	4.52E-03
NDFIP1	Organelle biogenesis and maintenance	72/1736	296/10891	1.02E-04	6.92E-03
NDFIP1	HIV Infection	59/1736	231/10891	1.03E-04	6.92E-03
NDFIP1	Signaling by CTNNB1 phospho-site mutants	9/1736	15/10891	1.30E-04	6.92E-03
NDFIP1	Signaling by GSK3beta mutants	9/1736	15/10891	1.30E-04	6.92E-03
NDFIP1	CTNNB1 S33 mutants aren't phosphorylated	9/1736	15/10891	1.30E-04	6.92E-03
NDFIP1	CTNNB1 S37 mutants aren't phosphorylated	9/1736	15/10891	1.30E-04	6.92E-03
NDFIP1	CTNNB1 S45 mutants aren't phosphorylated	9/1736	15/10891	1.30E-04	6.92E-03
NDFIP1	CTNNB1 T41 mutants aren't phosphorylated	9/1736	15/10891	1.30E-04	6.92E-03
NDFIP1	Neuronal System	93/1736	410/10891	1.74E-04	8.94E-03
NDFIP1	Translocation of SLC2A4 (GLUT4) to the plasma membrane	24/1736	72/10891	2.03E-04	9.59E-03
NDFIP1	RAF activation	15/1736	36/10891	2.03E-04	9.59E-03
NDFIP1	COPII-mediated vesicle transport	23/1736	68/10891	2.14E-04	9.59E-03

NDFIP1	Class I MHC mediated antigen processing & presentation	87/1736	381/10891	2.20E-04	9.59E-03
NDFIP1	RNA Polymerase II Pre-transcription Events	26/1736	81/10891	2.24E-04	9.59E-03
NDFIP1	SARS-CoV Infections	86/1736	376/10891	2.26E-04	9.59E-03
NDFIP1	GABA receptor activation	21/1736	60/10891	2.32E-04	9.59E-03
NDFIP1	Golgi Associated Vesicle Biogenesis	20/1736	56/10891	2.38E-04	9.59E-03
NDFIP1	Maturation of spike protein	15/1736	37/10891	2.91E-04	1.14E-02
NDFIP1	COPI-mediated anterograde transport	30/1736	101/10891	3.55E-04	1.36E-02
NDFIP1	Signaling by Hedgehog	40/1736	149/10891	4.38E-04	1.58E-02
NDFIP1	Beta-catenin phosphorylation cascade	9/1736	17/10891	4.66E-04	1.58E-02
NDFIP1	Signaling by AXIN mutants	8/1736	14/10891	4.98E-04	1.58E-02
NDFIP1	Signaling by APC mutants	8/1736	14/10891	4.98E-04	1.58E-02
NDFIP1	Signaling by AMER1 mutants	8/1736	14/10891	4.98E-04	1.58E-02
NDFIP1	APC truncation mutants have impaired AXIN binding	8/1736	14/10891	4.98E-04	1.58E-02
NDFIP1	AXIN missense mutants destabilize the destruction complex	8/1736	14/10891	4.98E-04	1.58E-02
NDFIP1	Truncations of AMER1 destabilize the destruction complex	8/1736	14/10891	4.98E-04	1.58E-02
NDFIP1	COPI-independent Golgi-to-ER retrograde traffic	18/1736	51/10891	5.68E-04	1.73E-02
NDFIP1	COPI-dependent Golgi-to-ER retrograde traffic	29/1736	99/10891	5.69E-04	1.73E-02
NDFIP1	Cilium Assembly	50/1736	201/10891	6.48E-04	1.93E-02
NDFIP1	SARS-CoV-2 Infection	67/1736	288/10891	6.63E-04	1.94E-02
NDFIP1	Recruitment of mitotic centrosome proteins and complexes	25/1736	82/10891	6.99E-04	1.97E-02
NDFIP1	Centrosome maturation	25/1736	82/10891	6.99E-04	1.97E-02
NDFIP1	Activation of kainate receptors upon glutamate binding	13/1736	32/10891	7.17E-04	1.98E-02
NDFIP1	EPH-Ephrin signaling	27/1736	92/10891	8.42E-04	2.28E-02
NDFIP1	Transcription of the HIV genome	22/1736	70/10891	9.13E-04	2.40E-02
NDFIP1	Activation of BAD and translocation to mitochondria	8/1736	15/10891	9.18E-04	2.40E-02
NDFIP1	SARS-CoV-2 targets host intracellular signalling and regulatory pathways	7/1736	12/10891	9.74E-04	2.50E-02
NDFIP1	ADP signalling through P2Y purinoceptor 12	10/1736	22/10891	1.05E-03	2.62E-02
NDFIP1	Processing of Capped Intron-Containing Pre-mRNA	58/1736	246/10891	1.05E-03	2.62E-02
NDFIP1	Anchoring of the basal body to the plasma membrane	28/1736	98/10891	1.08E-03	2.64E-02
NDFIP1	Formation of RNA Pol II elongation complex	19/1736	58/10891	1.15E-03	2.72E-02
NDFIP1	RNA Polymerase II Transcription Elongation	19/1736	58/10891	1.15E-03	2.72E-02
NDFIP1	Recruitment of NuMA to mitotic centrosomes	27/1736	94/10891	1.20E-03	2.80E-02
NDFIP1	Phospholipid metabolism	51/1736	212/10891	1.29E-03	2.95E-02

NDFIP1	RAB GEFs exchange GTP for GDP on RABs	26/1736	90/10891	1.34E-03	3.03E-02
NDFIP1	L1CAM interactions	32/1736	119/10891	1.52E-03	3.38E-02
NDFIP1	Amino acids regulate mTORC1	18/1736	55/10891	1.55E-03	3.41E-02
NDFIP1	Fc epsilon receptor (FCERI) signaling	35/1736	134/10891	1.63E-03	3.52E-02
NDFIP1	HIV Life Cycle	38/1736	149/10891	1.70E-03	3.61E-02
NDFIP1	Diseases of signal transduction by growth factor receptors and second messengers	92/1736	433/10891	1.78E-03	3.74E-02
NDFIP1	Advanced glycosylation endproduct receptor signaling	7/1736	13/10891	1.82E-03	3.77E-02
NDFIP1	Regulation of insulin secretion	23/1736	78/10891	1.85E-03	3.77E-02
NDFIP1	MASTL Facilitates Mitotic Progression	6/1736	10/10891	1.91E-03	3.80E-02
NDFIP1	N-glycan trimming in the ER and Calnexin/Calreticulin cycle	13/1736	35/10891	1.91E-03	3.80E-02
NDFIP1	Degradation of beta-catenin by the destruction complex	24/1736	83/10891	1.99E-03	3.91E-02
NDFIP1	Processing of Intronless Pre-mRNAs	9/1736	20/10891	2.04E-03	3.96E-02
NDFIP1	Regulation of PLK1 Activity at G2/M Transition	25/1736	88/10891	2.13E-03	4.06E-02
NDFIP1	Activation of NMDA receptors and postsynaptic events	26/1736	93/10891	2.25E-03	4.17E-02
NDFIP1	Loss of Nlp from mitotic centrosomes	21/1736	70/10891	2.26E-03	4.17E-02
NDFIP1	Loss of proteins required for interphase microtubule organization from the centrosome	21/1736	70/10891	2.26E-03	4.17E-02
NDFIP1	EGR2 and SOX10-mediated initiation of Schwann cell myelination	11/1736	28/10891	2.52E-03	4.57E-02
NDFIP1	RHO GTPases activate IQGAPs	12/1736	32/10891	2.58E-03	4.60E-02
NDFIP1	Hedgehog 'off' state	30/1736	113/10891	2.60E-03	4.60E-02
NDFIP1	Antigen processing: Ubiquitination & Proteasome degradation	68/1736	309/10891	2.78E-03	4.88E-02
NTN5	KEAP1-NFE2L2 pathway	67/2833	102/10891	3.64E-17	5.58E-14
NTN5	Nuclear events mediated by NFE2L2	54/2833	79/10891	3.21E-15	2.46E-12
NTN5	HIV Infection	115/2833	231/10891	4.92E-15	2.52E-12
NTN5	Cellular response to chemical stress	100/2833	194/10891	1.77E-14	6.79E-12
NTN5	Asparagine N-linked glycosylation	137/2833	304/10891	2.75E-13	8.44E-11
NTN5	Host Interactions of HIV factors	70/2833	130/10891	1.09E-11	2.80E-09
NTN5	Cross-presentation of soluble exogenous antigens (endosomes)	35/2833	49/10891	3.48E-11	7.63E-09
NTN5	Regulation of mRNA stability by proteins that bind AU-rich elements	52/2833	88/10891	5.01E-11	9.60E-09
NTN5	ER to Golgi Anterograde Transport	77/2833	154/10891	1.26E-10	2.14E-08
NTN5	Citric acid cycle (TCA cycle)	20/2833	22/10891	2.51E-10	3.75E-08
NTN5	Processing of Capped Intron-Containing Pre-mRNA	109/2833	246/10891	2.68E-10	3.75E-08
NTN5	Hedgehog ligand biogenesis	41/2833	65/10891	3.29E-10	3.82E-08

NTN5	Hh mutants are degraded by ERAD	37/2833	56/10891	3.43E-10	3.82E-08
NTN5	Translation	124/2833	291/10891	3.49E-10	3.82E-08
NTN5	Negative regulation of NOTCH4 signaling	36/2833	54/10891	4.06E-10	4.01E-08
NTN5	Transport to the Golgi and subsequent modification	87/2833	185/10891	4.66E-10	4.01E-08
NTN5	Autodegradation of the E3 ubiquitin ligase COP1	35/2833	52/10891	4.77E-10	4.01E-08
NTN5	Ubiquitin-dependent degradation of Cyclin D	35/2833	52/10891	4.77E-10	4.01E-08
NTN5	Defective CFTR causes cystic fibrosis	39/2833	61/10891	4.96E-10	4.01E-08
NTN5	Regulation of activated PAK-2p34 by proteasome mediated degradation	34/2833	50/10891	5.56E-10	4.22E-08
NTN5	Antigen processing-Cross presentation	57/2833	105/10891	5.92E-10	4.22E-08
NTN5	Hh mutants abrogate ligand secretion	38/2833	59/10891	6.05E-10	4.22E-08
NTN5	SCF-beta-TrCP mediated degradation of Emi1	36/2833	55/10891	8.82E-10	5.89E-08
NTN5	Regulation of Apoptosis	35/2833	53/10891	1.05E-09	6.75E-08
NTN5	Regulation of ornithine decarboxylase (ODC)	34/2833	51/10891	1.25E-09	7.68E-08
NTN5	AUF1 (hnRNP D0) binds and destabilizes mRNA	36/2833	56/10891	1.85E-09	1.09E-07
NTN5	Vif-mediated degradation of APOBEC3G	35/2833	54/10891	2.25E-09	1.23E-07
NTN5	FBXL7 down-regulates AURKA during mitotic entry and in early mitosis	35/2833	54/10891	2.25E-09	1.23E-07
NTN5	ER-Phagosome pathway	50/2833	90/10891	2.33E-09	1.23E-07
NTN5	GSK3B and BTRC:CUL1-mediated-degradation of NFE2L2	34/2833	52/10891	2.71E-09	1.39E-07
NTN5	NIK-->noncanonical NF-kB signaling	37/2833	59/10891	3.05E-09	1.51E-07
NTN5	Stabilization of p53	36/2833	57/10891	3.77E-09	1.81E-07
NTN5	Dectin-1 mediated noncanonical NF-kB signaling	37/2833	60/10891	5.98E-09	2.74E-07
NTN5	tRNA Aminoacylation	29/2833	42/10891	6.07E-09	2.74E-07
NTN5	Cytosolic tRNA aminoacylation	20/2833	24/10891	6.57E-09	2.88E-07
NTN5	Mitotic Metaphase and Anaphase	102/2833	237/10891	6.86E-09	2.92E-07
NTN5	Cellular response to hypoxia	43/2833	75/10891	8.48E-09	3.52E-07
NTN5	Mitochondrial translation	50/2833	93/10891	1.03E-08	4.16E-07
NTN5	rRNA modification in the nucleus and cytosol	37/2833	61/10891	1.14E-08	4.34E-07
NTN5	COPI-mediated anterograde transport	53/2833	101/10891	1.15E-08	4.34E-07
NTN5	Mitotic Anaphase	101/2833	236/10891	1.16E-08	4.34E-07
NTN5	Oxygen-dependent proline hydroxylation of Hypoxia-inducible Factor Alpha	39/2833	66/10891	1.30E-08	4.69E-07
NTN5	Vpu mediated degradation of CD4	33/2833	52/10891	1.42E-08	4.69E-07
NTN5	Ubiquitin Mediated Degradation of Phosphorylated Cdc25A	33/2833	52/10891	1.42E-08	4.69E-07
NTN5	p53-Independent DNA Damage Response	33/2833	52/10891	1.42E-08	4.69E-07
NTN5	p53-Independent G1/S DNA damage checkpoint	33/2833	52/10891	1.42E-08	4.69E-07
NTN5	Metabolism of polyamines	36/2833	59/10891	1.44E-08	4.69E-07
NTN5	Autodegradation of Cdh1 by Cdh1:APC/C	38/2833	64/10891	1.66E-08	5.32E-07
NTN5	Degradation of DVL	35/2833	57/10891	1.81E-08	5.66E-07

NTN5	Pyruvate metabolism and Citric Acid (TCA) cycle	34/2833	55/10891	2.26E-08	6.81E-07
NTN5	Regulation of RUNX3 expression and activity	34/2833	55/10891	2.26E-08	6.81E-07
NTN5	Activation of NF-kappaB in B cells	39/2833	67/10891	2.34E-08	6.91E-07
NTN5	The role of GTSE1 in G2/M progression after G2 checkpoint	43/2833	77/10891	2.48E-08	7.20E-07
NTN5	Degradation of GLI1 by the proteasome	36/2833	60/10891	2.70E-08	7.39E-07
NTN5	Degradation of GLI2 by the proteasome	36/2833	60/10891	2.70E-08	7.39E-07
NTN5	GLI3 is processed to GLI3R by the proteasome	36/2833	60/10891	2.70E-08	7.39E-07
NTN5	Intra-Golgi and retrograde Golgi-to-ER traffic	88/2833	202/10891	3.74E-08	1.01E-06
NTN5	mRNA Splicing - Major Pathway	82/2833	185/10891	4.30E-08	1.14E-06
NTN5	HIV Life Cycle	69/2833	149/10891	6.24E-08	1.62E-06
NTN5	Regulation of PTEN stability and activity	39/2833	69/10891	7.10E-08	1.82E-06
NTN5	Mitochondrial translation elongation	46/2833	87/10891	7.79E-08	1.96E-06
NTN5	ABC-family proteins mediated transport	52/2833	103/10891	8.40E-08	2.02E-06
NTN5	FCERI mediated NF-kB activation	44/2833	82/10891	8.44E-08	2.02E-06
NTN5	Signaling by NOTCH4	44/2833	82/10891	8.44E-08	2.02E-06
NTN5	Class I MHC mediated antigen processing & presentation	145/2833	381/10891	1.00E-07	2.37E-06
NTN5	Degradation of AXIN	33/2833	55/10891	1.02E-07	2.38E-06
NTN5	Downstream signaling events of B Cell Receptor (BCR)	44/2833	83/10891	1.35E-07	3.04E-06
NTN5	Degradation of beta-catenin by the destruction complex	44/2833	83/10891	1.35E-07	3.04E-06
NTN5	S Phase	73/2833	163/10891	1.41E-07	3.12E-06
NTN5	M Phase	156/2833	418/10891	1.42E-07	3.12E-06
NTN5	ABC transporter disorders	42/2833	78/10891	1.45E-07	3.14E-06
NTN5	Regulation of RUNX2 expression and activity	40/2833	73/10891	1.53E-07	3.25E-06
NTN5	APC/C:Cdc20 mediated degradation of Securin	38/2833	68/10891	1.57E-07	3.25E-06
NTN5	Regulation of RAS by GAPs	38/2833	68/10891	1.57E-07	3.25E-06
NTN5	Regulation of APC/C activators between G1/S and early anaphase	43/2833	81/10891	1.77E-07	3.63E-06
NTN5	p53-Dependent G1 DNA Damage Response	37/2833	66/10891	2.04E-07	4.07E-06
NTN5	p53-Dependent G1/S DNA damage checkpoint	37/2833	66/10891	2.04E-07	4.07E-06
NTN5	Neutrophil degranulation	175/2833	482/10891	2.20E-07	4.33E-06
NTN5	Separation of Sister Chromatids	82/2833	191/10891	2.32E-07	4.51E-06
NTN5	APC/C:Cdh1 mediated degradation of Cdc20 and other APC/C:Cdh1 targeted proteins in late mitosis/early G1	40/2833	74/10891	2.50E-07	4.69E-06
NTN5	Mitochondrial translation initiation	45/2833	87/10891	2.50E-07	4.69E-06
NTN5	Mitochondrial translation termination	45/2833	87/10891	2.50E-07	4.69E-06
NTN5	ISG15 antiviral mechanism	39/2833	72/10891	3.28E-07	6.07E-06
NTN5	Interleukin-1 signaling	55/2833	115/10891	3.64E-07	6.66E-06
NTN5	mRNA Splicing	82/2833	193/10891	3.95E-07	7.13E-06

NTN5	SCF(Skp2)-mediated degradation of p27/p21	34/2833	60/10891	4.49E-07	8.02E-06
NTN5	Cdc20:Phospho-APC/C mediated degradation of Cyclin A	39/2833	73/10891	5.29E-07	9.22E-06
NTN5	CDK-mediated phosphorylation and removal of Cdc6	39/2833	73/10891	5.29E-07	9.22E-06
NTN5	snRNP Assembly	31/2833	53/10891	5.55E-07	9.46E-06
NTN5	Metabolism of non-coding RNA	31/2833	53/10891	5.55E-07	9.46E-06
NTN5	G1/S DNA Damage Checkpoints	37/2833	68/10891	5.64E-07	9.51E-06
NTN5	Late Phase of HIV Life Cycle	62/2833	136/10891	5.70E-07	9.52E-06
NTN5	APC/C:Cdc20 mediated degradation of mitotic proteins	40/2833	76/10891	6.37E-07	1.05E-05
NTN5	Mitotic G2-G2/M phases	83/2833	198/10891	6.62E-07	1.08E-05
NTN5	APC:Cdc20 mediated degradation of cell cycle proteins prior to satisfation of the cell cycle checkpoint	39/2833	74/10891	8.39E-07	1.36E-05
NTN5	G2/M Transition	82/2833	196/10891	8.50E-07	1.36E-05
NTN5	Activation of APC/C and APC/C:Cdc20 mediated degradation of mitotic proteins	40/2833	77/10891	9.95E-07	1.58E-05
NTN5	Cyclin A:Cdk2-associated events at S phase entry	43/2833	85/10891	1.01E-06	1.59E-05
NTN5	Disorders of transmembrane transporters	75/2833	176/10891	1.06E-06	1.65E-05
NTN5	tRNA processing	51/2833	107/10891	1.09E-06	1.68E-05
NTN5	APC/C-mediated degradation of cell cycle proteins	44/2833	88/10891	1.15E-06	1.73E-05
NTN5	Regulation of mitotic cell cycle	44/2833	88/10891	1.15E-06	1.73E-05
NTN5	Antiviral mechanism by IFN-stimulated genes	41/2833	80/10891	1.16E-06	1.73E-05
NTN5	Hedgehog 'off' state	53/2833	113/10891	1.27E-06	1.87E-05
NTN5	UCH proteinases	49/2833	102/10891	1.32E-06	1.94E-05
NTN5	Cyclin E associated events during G1/S transition	42/2833	83/10891	1.34E-06	1.94E-05
NTN5	Signaling by Hedgehog	65/2833	149/10891	2.08E-06	2.99E-05
NTN5	Orc1 removal from chromatin	37/2833	71/10891	2.28E-06	3.24E-05
NTN5	Transport of Mature mRNA Derived from an Intronless Transcript	25/2833	41/10891	2.41E-06	3.39E-05
NTN5	CLEC7A (Dectin-1) signaling	48/2833	101/10891	2.49E-06	3.48E-05
NTN5	Diseases of signal transduction by growth factor receptors and second messengers	155/2833	433/10891	2.84E-06	3.93E-05
NTN5	Transcriptional regulation by RUNX3	46/2833	96/10891	3.03E-06	4.15E-05
NTN5	Golgi-to-ER retrograde transport	59/2833	133/10891	3.18E-06	4.31E-05
NTN5	Asymmetric localization of PCP proteins	34/2833	64/10891	3.32E-06	4.47E-05
NTN5	Fc epsilon receptor (FCERI) signaling	59/2833	134/10891	4.25E-06	5.67E-05
NTN5	Transport of Mature mRNAs Derived from Intronless Transcripts	25/2833	42/10891	4.48E-06	5.93E-05
NTN5	tRNA processing in the nucleus	31/2833	57/10891	4.69E-06	6.15E-05
NTN5	MAPK6/MAPK4 signaling	43/2833	89/10891	4.81E-06	6.26E-05

NTN5	NS1 Mediated Effects on Host Pathways	24/2833	40/10891	5.73E-06	7.39E-05
NTN5	Nuclear import of Rev protein	21/2833	33/10891	5.93E-06	7.58E-05
NTN5	Apoptosis	74/2833	180/10891	6.16E-06	7.82E-05
NTN5	Transport of Ribonucleoproteins into the Host Nucleus	20/2833	31/10891	7.31E-06	9.19E-05
NTN5	Neddylation	93/2833	240/10891	8.38E-06	1.05E-04
NTN5	G1/S Transition	57/2833	131/10891	9.53E-06	1.18E-04
NTN5	Transport of the SLBP independent Mature mRNA	21/2833	34/10891	1.17E-05	1.44E-04
NTN5	Establishment of Sister Chromatid Cohesion	10/2833	11/10891	1.18E-05	1.44E-04
NTN5	Switching of origins to a post-replicative state	43/2833	92/10891	1.39E-05	1.68E-04
NTN5	Signaling by the B Cell Receptor (BCR)	50/2833	112/10891	1.41E-05	1.70E-04
NTN5	Synthesis of DNA	53/2833	121/10891	1.54E-05	1.83E-04
NTN5	Transport of Mature Transcript to Cytoplasm	40/2833	84/10891	1.58E-05	1.87E-04
NTN5	The citric acid (TCA) cycle and respiratory electron transport	72/2833	178/10891	1.60E-05	1.87E-04
NTN5	C-type lectin receptors (CLRs)	60/2833	142/10891	1.69E-05	1.97E-04
NTN5	COPII-mediated vesicle transport	34/2833	68/10891	1.86E-05	2.15E-04
NTN5	Programmed Cell Death	82/2833	210/10891	2.03E-05	2.32E-04
NTN5	Regulation of cholesterol biosynthesis by SREBP (SREBF)	29/2833	55/10891	2.09E-05	2.38E-04
NTN5	Transport of the SLBP Dependant Mature mRNA	21/2833	35/10891	2.21E-05	2.50E-04
NTN5	Export of Viral Ribonucleoproteins from Nucleus	20/2833	33/10891	2.82E-05	3.16E-04
NTN5	Golgi Associated Vesicle Biogenesis	29/2833	56/10891	3.27E-05	3.63E-04
NTN5	Transcriptional regulation by RUNX2	52/2833	121/10891	3.48E-05	3.85E-04
NTN5	NEP/NS2 Interacts with the Cellular Export Machinery	19/2833	31/10891	3.59E-05	3.88E-04
NTN5	Regulation of Glucokinase by Glucokinase Regulatory Protein	19/2833	31/10891	3.59E-05	3.88E-04
NTN5	Defective TPR may confer susceptibility towards thyroid papillary carcinoma (TPC)	19/2833	31/10891	3.59E-05	3.88E-04
NTN5	Downstream TCR signaling	44/2833	98/10891	3.83E-05	4.11E-04
NTN5	Interactions of Vpr with host cellular proteins	21/2833	36/10891	4.01E-05	4.25E-04
NTN5	Interactions of Rev with host cellular proteins	21/2833	36/10891	4.01E-05	4.25E-04
NTN5	Folding of actin by CCT/TriC	9/2833	10/10891	4.14E-05	4.35E-04
NTN5	Mitotic G1 phase and G1/S transition	61/2833	149/10891	4.53E-05	4.73E-04
NTN5	Rev-mediated nuclear export of HIV RNA	20/2833	34/10891	5.19E-05	5.38E-04
NTN5	TNFR2 non-canonical NF-kB pathway	45/2833	102/10891	5.33E-05	5.49E-04
NTN5	Association of TriC/CCT with target proteins during biosynthesis	22/2833	39/10891	5.36E-05	5.49E-04
NTN5	Hedgehog 'on' state	39/2833	85/10891	5.76E-05	5.85E-04
NTN5	Glycolysis	34/2833	71/10891	5.83E-05	5.88E-04
NTN5	rRNA processing	78/2833	204/10891	7.40E-05	7.43E-04

NTN5	SARS-CoV-2 Infection	104/2833	288/10891	8.12E-05	8.09E-04
NTN5	SUMOylation of DNA replication proteins	24/2833	45/10891	8.40E-05	8.32E-04
NTN5	Transport of Mature mRNA derived from an Intron-Containing Transcript	35/2833	75/10891	8.89E-05	8.74E-04
NTN5	PTEN Regulation	57/2833	140/10891	9.52E-05	9.30E-04
NTN5	Viral Messenger RNA Synthesis	23/2833	43/10891	1.11E-04	1.08E-03
NTN5	SUMOylation of ubiquitinylation proteins	21/2833	38/10891	1.18E-04	1.14E-03
NTN5	Vpr-mediated nuclear import of PICs	19/2833	33/10891	1.19E-04	1.15E-03
NTN5	Autophagy	60/2833	151/10891	1.43E-04	1.36E-03
NTN5	Glucose metabolism	40/2833	91/10891	1.50E-04	1.42E-03
NTN5	SARS-CoV Infections	129/2833	376/10891	1.73E-04	1.63E-03
NTN5	Intra-Golgi traffic	23/2833	44/10891	1.76E-04	1.65E-03
NTN5	trans-Golgi Network Vesicle Budding	33/2833	72/10891	2.14E-04	1.99E-03
NTN5	Interleukin-1 family signaling	60/2833	153/10891	2.18E-04	2.02E-03
NTN5	SARS-CoV-2 activates/modulates innate and adaptive immune responses	51/2833	126/10891	2.57E-04	2.37E-03
NTN5	Antigen processing: Ubiquitination & Proteasome degradation	108/2833	309/10891	2.60E-04	2.38E-03
NTN5	Processing of Intronless Pre-mRNAs	13/2833	20/10891	2.79E-04	2.53E-03
NTN5	Nuclear Pore Complex (NPC) Disassembly	19/2833	35/10891	3.40E-04	3.02E-03
NTN5	N-glycan trimming in the ER and Calnexin/Calreticulin cycle	19/2833	35/10891	3.40E-04	3.02E-03
NTN5	RHOBTB GTPase Cycle	19/2833	35/10891	3.40E-04	3.02E-03
NTN5	Chaperonin-mediated protein folding	39/2833	91/10891	3.41E-04	3.02E-03
NTN5	Deubiquitination	104/2833	298/10891	3.57E-04	3.15E-03
NTN5	TCR signaling	48/2833	120/10891	5.30E-04	4.65E-03
NTN5	Cohesin Loading onto Chromatin	8/2833	10/10891	5.55E-04	4.84E-03
NTN5	Signaling by PDGF	27/2833	58/10891	5.79E-04	5.02E-03
NTN5	Macroautophagy	53/2833	136/10891	5.89E-04	5.08E-03
NTN5	DNA Repair	114/2833	336/10891	6.48E-04	5.56E-03
NTN5	rRNA processing in the nucleus and cytosol	71/2833	194/10891	6.74E-04	5.75E-03
NTN5	Mitotic Prometaphase	74/2833	204/10891	7.06E-04	5.99E-03
NTN5	SUMOylation of SUMOylation proteins	18/2833	34/10891	7.24E-04	6.11E-03
NTN5	Protein folding	40/2833	97/10891	7.38E-04	6.19E-03
NTN5	Processing of Capped Intronless Pre-mRNA	16/2833	29/10891	7.87E-04	6.57E-03
NTN5	mRNA 3'-end processing	27/2833	59/10891	8.06E-04	6.69E-03
NTN5	RNA Polymerase II Transcription Termination	30/2833	68/10891	8.97E-04	7.41E-03
NTN5	COPI-independent Golgi-to-ER retrograde traffic	24/2833	51/10891	9.51E-04	7.78E-03
NTN5	PCP/CE pathway	38/2833	92/10891	9.53E-04	7.78E-03
NTN5	RHO GTPase cycle	146/2833	449/10891	1.00E-03	8.12E-03
NTN5	Organelle biogenesis and maintenance	101/2833	296/10891	1.04E-03	8.37E-03
NTN5	Postmitotic nuclear pore complex (NPC) reformation	15/2833	27/10891	1.04E-03	8.37E-03

NTN5	RAF/MAP kinase cascade	96/2833	280/10891	1.14E-03	9.15E-03
NTN5	COPI-dependent Golgi-to-ER retrograde traffic	40/2833	99/10891	1.18E-03	9.42E-03
NTN5	Regulation of HSF1-mediated heat shock response	30/2833	69/10891	1.20E-03	9.48E-03
NTN5	RAB GEFs exchange GTP for GDP on RABs	37/2833	90/10891	1.22E-03	9.62E-03
NTN5	Nuclear Envelope (NE) Reassembly	32/2833	75/10891	1.23E-03	9.62E-03
NTN5	Cargo concentration in the ER	17/2833	33/10891	1.51E-03	1.18E-02
NTN5	Cytosolic sensors of pathogen-associated DNA	28/2833	64/10891	1.54E-03	1.19E-02
NTN5	Purine ribonucleoside monophosphate biosynthesis	8/2833	11/10891	1.57E-03	1.21E-02
NTN5	Protein ubiquitination	33/2833	79/10891	1.60E-03	1.22E-02
NTN5	MAPK1/MAPK3 signaling	97/2833	286/10891	1.60E-03	1.22E-02
NTN5	Mitochondrial calcium ion transport	13/2833	23/10891	1.83E-03	1.37E-02
NTN5	RHOBTB2 GTPase cycle	13/2833	23/10891	1.83E-03	1.37E-02
NTN5	RHOBTB1 GTPase cycle	13/2833	23/10891	1.83E-03	1.37E-02
NTN5	Major pathway of rRNA processing in the nucleolus and cytosol	66/2833	184/10891	1.85E-03	1.38E-02
NTN5	Cellular response to heat stress	36/2833	89/10891	1.99E-03	1.48E-02
NTN5	SUMOylation of DNA damage response and repair proteins	32/2833	77/10891	2.07E-03	1.53E-02
NTN5	Activation of gene expression by SREBF (SREBP)	20/2833	42/10891	2.07E-03	1.53E-02
NTN5	Nucleotide Excision Repair	43/2833	111/10891	2.12E-03	1.56E-02
NTN5	Cell Cycle Checkpoints	98/2833	293/10891	2.48E-03	1.81E-02
NTN5	Maturation of spike protein	18/2833	37/10891	2.54E-03	1.85E-02
NTN5	Interleukin-12 family signaling	25/2833	57/10891	2.58E-03	1.85E-02
NTN5	Translation of Structural Proteins	25/2833	57/10891	2.58E-03	1.85E-02
NTN5	Late SARS-CoV-2 Infection Events	25/2833	57/10891	2.58E-03	1.85E-02
NTN5	RNA Polymerase II Pre-transcription Events	33/2833	81/10891	2.61E-03	1.87E-02
NTN5	Transcriptional Regulation by TP53	118/2833	362/10891	2.66E-03	1.89E-02
NTN5	Downstream signal transduction	15/2833	29/10891	2.70E-03	1.90E-02
NTN5	Antigen Presentation: Folding, assembly and peptide loading of class I MHC	15/2833	29/10891	2.70E-03	1.90E-02
NTN5	Ub-specific processing proteases	76/2833	220/10891	2.84E-03	1.99E-02
NTN5	MAPK family signaling cascades	107/2833	325/10891	2.88E-03	2.01E-02
NTN5	Nucleotide biosynthesis	9/2833	14/10891	2.89E-03	2.01E-02
NTN5	Retrograde transport at the Trans-Golgi-Network	22/2833	49/10891	3.21E-03	2.22E-02
NTN5	Intracellular signaling by second messengers	102/2833	309/10891	3.26E-03	2.24E-02
NTN5	Nuclear Envelope Breakdown	23/2833	52/10891	3.31E-03	2.27E-02
NTN5	Signaling by ALK in cancer	24/2833	55/10891	3.38E-03	2.29E-02
NTN5	Signaling by ALK fusions and activated point mutants	24/2833	55/10891	3.38E-03	2.29E-02
NTN5	Iron uptake and transport	25/2833	58/10891	3.43E-03	2.32E-02
NTN5	Signaling by ALK	14/2833	27/10891	3.61E-03	2.42E-02

NTN5	Insulin processing	14/2833	27/10891	3.61E-03	2.42E-02
NTN5	ATF6 (ATF6-alpha) activates chaperones	8/2833	12/10891	3.63E-03	2.42E-02
NTN5	DNA Damage Recognition in GG-NER	18/2833	38/10891	3.67E-03	2.42E-02
NTN5	Cooperation of PDCL (PhLP1) and TRiC/CCT in G-protein beta folding	18/2833	38/10891	3.67E-03	2.42E-02
NTN5	Gene and protein expression by JAK-STAT signaling after Interleukin-12 stimulation	18/2833	38/10891	3.67E-03	2.42E-02
NTN5	SARS-CoV-2-host interactions	70/2833	202/10891	3.72E-03	2.44E-02
NTN5	Interconversion of nucleotide di- and triphosphates	15/2833	30/10891	4.11E-03	2.66E-02
NTN5	Regulation of TP53 Activity through Acetylation	15/2833	30/10891	4.11E-03	2.66E-02
NTN5	Protein localization	58/2833	163/10891	4.11E-03	2.66E-02
NTN5	SUMOylation of RNA binding proteins	21/2833	47/10891	4.23E-03	2.72E-02
NTN5	Interleukin-12 signaling	21/2833	47/10891	4.23E-03	2.72E-02
NTN5	Regulation of TP53 Activity	57/2833	160/10891	4.29E-03	2.75E-02
NTN5	RHOG GTPase cycle	30/2833	74/10891	4.36E-03	2.78E-02
NTN5	SARS-CoV-1 Infection	23/2833	53/10891	4.42E-03	2.81E-02
NTN5	rRNA processing in the mitochondrion	7/2833	10/10891	4.46E-03	2.82E-02
NTN5	G2/M Checkpoints	59/2833	167/10891	4.58E-03	2.88E-02
NTN5	Signaling by high-kinase activity BRAF mutants	17/2833	36/10891	4.89E-03	3.06E-02
NTN5	Toll-like Receptor Cascades	59/2833	168/10891	5.30E-03	3.31E-02
NTN5	Prefoldin mediated transfer of substrate to CCT/TriC	14/2833	28/10891	5.51E-03	3.41E-02
NTN5	Diseases associated with glycosylation precursor biosynthesis	9/2833	15/10891	5.56E-03	3.41E-02
NTN5	Constitutive Signaling by EGFRvIII	9/2833	15/10891	5.56E-03	3.41E-02
NTN5	Signaling by EGFRvIII in Cancer	9/2833	15/10891	5.56E-03	3.41E-02
NTN5	Extension of Telomeres	22/2833	51/10891	5.79E-03	3.51E-02
NTN5	Cargo trafficking to the periciliary membrane	22/2833	51/10891	5.79E-03	3.51E-02
NTN5	Unfolded Protein Response (UPR)	36/2833	94/10891	5.81E-03	3.51E-02
NTN5	RAC3 GTPase cycle	36/2833	94/10891	5.81E-03	3.51E-02
NTN5	MHC class II antigen presentation	45/2833	123/10891	6.04E-03	3.63E-02
NTN5	Cristae formation	15/2833	31/10891	6.07E-03	3.64E-02
NTN5	Regulation of PLK1 Activity at G2/M Transition	34/2833	88/10891	6.17E-03	3.68E-02
NTN5	PIP3 activates AKT signaling	88/2833	267/10891	6.25E-03	3.72E-02
NTN5	Recruitment of mitotic centrosome proteins and complexes	32/2833	82/10891	6.52E-03	3.85E-02
NTN5	Centrosome maturation	32/2833	82/10891	6.52E-03	3.85E-02
NTN5	Mitochondrial Fatty Acid Beta-Oxidation	17/2833	37/10891	6.89E-03	4.05E-02
NTN5	Mitochondrial biogenesis	36/2833	95/10891	7.05E-03	4.13E-02
NTN5	MAP2K and MAPK activation	18/2833	40/10891	7.17E-03	4.18E-02
NTN5	Signaling by BRAF and RAF1 fusions	27/2833	67/10891	7.29E-03	4.21E-02
NTN5	Mitotic Telophase/Cytokinesis	8/2833	13/10891	7.30E-03	4.21E-02

NTN5	Synthesis of PIPs at the Golgi membrane	10/2833	18/10891	7.30E-03	4.21E-02
NTN5	Cytoprotection by HMOX1	26/2833	64/10891	7.40E-03	4.24E-02
NTN5	Calnexin/calreticulin cycle	13/2833	26/10891	7.41E-03	4.24E-02
NTN5	Formation of RNA Pol II elongation complex	24/2833	58/10891	7.57E-03	4.30E-02
NTN5	RNA Polymerase II Transcription Elongation	24/2833	58/10891	7.57E-03	4.30E-02
NTN5	HSP90 chaperone cycle for steroid hormone receptors (SHR) in the presence of ligand	23/2833	55/10891	7.61E-03	4.31E-02
NTN5	Protein-protein interactions at synapses	33/2833	86/10891	7.78E-03	4.39E-02
NTN5	RNA polymerase II transcribes snRNA genes	29/2833	74/10891	8.72E-03	4.89E-02
NTN5	Cooperation of Prefoldin and TriC/CCT in actin and tubulin folding	15/2833	32/10891	8.72E-03	4.89E-02
NTN5	ZBP1(DAI) mediated induction of type I IFNs	11/2833	21/10891	8.78E-03	4.90E-02
RASIP1	Platelet activation, signaling and aggregation	38/577	263/10891	1.41E-08	1.42E-05
RASIP1	Platelet degranulation	23/577	129/10891	2.47E-07	1.24E-04
RASIP1	Response to elevated platelet cytosolic Ca2+	23/577	134/10891	5.01E-07	1.68E-04
RASIP1	Regulation of Insulin-like Growth Factor (IGF) transport and uptake by Insulin-like Growth Factor Binding Proteins (IGFBPs)	21/577	125/10891	2.25E-06	5.66E-04
RASIP1	Extracellular matrix organization	36/577	300/10891	3.42E-06	6.89E-04
RASIP1	GPCR ligand binding	46/577	467/10891	3.26E-05	5.47E-03
RASIP1	Complement cascade	12/577	58/10891	4.13E-05	5.95E-03
RASIP1	Post-translational protein phosphorylation	17/577	108/10891	4.90E-05	6.17E-03
RASIP1	Cell surface interactions at the vascular wall	19/577	137/10891	1.07E-04	1.19E-02
RASIP1	Regulation of Complement cascade	10/577	47/10891	1.41E-04	1.42E-02
RASIP1	Formation of Fibrin Clot (Clotting Cascade)	9/577	39/10891	1.57E-04	1.42E-02
RASIP1	Signaling by VEGF	16/577	108/10891	1.69E-04	1.42E-02
RASIP1	Class A/1 (Rhodopsin-like receptors)	34/577	335/10891	1.99E-04	1.54E-02
RASIP1	G alpha (s) signalling events	20/577	157/10891	2.31E-04	1.66E-02
RASIP1	Signaling by NTRK1 (TRKA)	16/577	115/10891	3.54E-04	2.38E-02
RASIP1	Signalling to RAS	6/577	20/10891	4.41E-04	2.77E-02
RASIP1	Diseases of glycosylation	18/577	143/10891	5.42E-04	2.93E-02
RASIP1	Molecules associated with elastic fibres	8/577	37/10891	5.82E-04	2.93E-02
RASIP1	Defective B3GALTL causes PpS	8/577	37/10891	5.82E-04	2.93E-02
RASIP1	Anti-inflammatory response favouring Leishmania parasite infection	20/577	169/10891	6.12E-04	2.93E-02
RASIP1	Leishmania parasite growth and survival	20/577	169/10891	6.12E-04	2.93E-02
RASIP1	Interleukin-10 signaling	9/577	47/10891	6.89E-04	2.95E-02
RASIP1	Signaling by NTRKs	17/577	134/10891	6.99E-04	2.95E-02
RASIP1	O-glycosylation of TSR domain-containing proteins	8/577	38/10891	7.04E-04	2.95E-02
RASIP1	G alpha (z) signalling events	9/577	48/10891	8.09E-04	3.15E-02
RASIP1	Diseases associated with O-glycosylation of proteins	11/577	68/10891	8.18E-04	3.15E-02

RASIP1	Platelet Aggregation (Plug Formation)	8/577	39/10891	8.45E-04	3.15E-02
RASIP1	Intrinsic Pathway of Fibrin Clot Formation	6/577	23/10891	1.00E-03	3.36E-02
RASIP1	Regulation of KIT signaling	5/577	16/10891	1.10E-03	3.36E-02
RASIP1	Rap1 signalling	5/577	16/10891	1.10E-03	3.36E-02
RASIP1	Defects of contact activation system (CAS) and kallikrein/kinin system (KKS)	5/577	16/10891	1.10E-03	3.36E-02
RASIP1	Diseases of hemostasis	5/577	16/10891	1.10E-03	3.36E-02
RASIP1	Chondroitin sulfate/dermatan sulfate metabolism	9/577	50/10891	1.10E-03	3.36E-02
RASIP1	Nuclear Events (kinase and transcription factor activation)	10/577	61/10891	1.26E-03	3.73E-02
RASIP1	Diseases of metabolism	25/577	249/10891	1.57E-03	4.53E-02
RASIP1	Signaling by SCF-KIT	8/577	43/10891	1.65E-03	4.62E-02
RASIP1	ADORA2B mediated anti-inflammatory cytokines production	16/577	134/10891	1.90E-03	4.98E-02
RASIP1	Dermatan sulfate biosynthesis	4/577	11/10891	1.91E-03	4.98E-02
RASIP1	Elastic fibre formation	8/577	44/10891	1.93E-03	4.98E-02
RUFY2	Respiratory electron transport, ATP synthesis by chemiosmotic coupling, and heat production by uncoupling proteins.	49/1642	127/10891	6.95E-11	1.00E-07
RUFY2	Mitochondrial translation elongation	38/1642	87/10891	1.35E-10	1.00E-07
RUFY2	The citric acid (TCA) cycle and respiratory electron transport	60/1642	178/10891	3.42E-10	1.68E-07
RUFY2	Mitochondrial translation	38/1642	93/10891	1.41E-09	5.19E-07
RUFY2	Mitochondrial translation initiation	36/1642	87/10891	2.52E-09	6.20E-07
RUFY2	Mitochondrial translation termination	36/1642	87/10891	2.52E-09	6.20E-07
RUFY2	Respiratory electron transport	38/1642	103/10891	3.93E-08	7.54E-06
RUFY2	Asparagine N-linked glycosylation	82/1642	304/10891	4.08E-08	7.54E-06
RUFY2	Cellular response to chemical stress	58/1642	194/10891	9.37E-08	1.54E-05
RUFY2	Mitochondrial protein import	27/1642	64/10891	1.53E-07	2.26E-05
RUFY2	TP53 Regulates Metabolic Genes	31/1642	87/10891	1.65E-06	2.22E-04
RUFY2	PTEN Regulation	43/1642	140/10891	1.92E-06	2.36E-04
RUFY2	Biosynthesis of the N-glycan precursor (dolichol lipid-linked oligosaccharide, LLO) and transfer to a nascent protein	28/1642	78/10891	4.42E-06	5.02E-04
RUFY2	Class I MHC mediated antigen processing & presentation	89/1642	381/10891	9.39E-06	9.90E-04
RUFY2	Cristae formation	15/1642	31/10891	1.21E-05	1.19E-03
RUFY2	Translation	71/1642	291/10891	1.63E-05	1.49E-03
RUFY2	Antigen processing-Cross presentation	33/1642	105/10891	1.72E-05	1.49E-03
RUFY2	Degradation of AXIN	21/1642	55/10891	2.33E-05	1.86E-03
RUFY2	KEAP1-NFE2L2 pathway	32/1642	102/10891	2.39E-05	1.86E-03
RUFY2	ER-Phagosome pathway	29/1642	90/10891	3.26E-05	2.41E-03
RUFY2	Maturation of spike protein	16/1642	37/10891	3.64E-05	2.55E-03

RUFY2	Transcriptional Regulation by TP53	83/1642	362/10891	3.83E-05	2.55E-03
RUFY2	Diseases associated with N-glycosylation of proteins	11/1642	20/10891	3.97E-05	2.55E-03
RUFY2	Complex I biogenesis	21/1642	57/10891	4.36E-05	2.68E-03
RUFY2	Nuclear events mediated by NFE2L2	26/1642	79/10891	5.49E-05	3.12E-03
RUFY2	Organelle biogenesis and maintenance	70/1642	296/10891	5.50E-05	3.12E-03
RUFY2	Regulation of ornithine decarboxylase (ODC)	19/1642	51/10891	8.38E-05	4.58E-03
RUFY2	APC/C:Cdc20 mediated degradation of Securin	23/1642	68/10891	9.05E-05	4.77E-03
RUFY2	Autodegradation of the E3 ubiquitin ligase COP1	19/1642	52/10891	1.14E-04	5.79E-03
RUFY2	APC/C:Cdh1 mediated degradation of Cdc20 and other APC/C:Cdh1 targeted proteins in late mitosis/early G1	24/1642	74/10891	1.36E-04	6.48E-03
RUFY2	PCP/CE pathway	28/1642	92/10891	1.36E-04	6.48E-03
RUFY2	Host Interactions of HIV factors	36/1642	130/10891	1.45E-04	6.48E-03
RUFY2	Stabilization of p53	20/1642	57/10891	1.45E-04	6.48E-03
RUFY2	Vif-mediated degradation of APOBEC3G	19/1642	54/10891	2.02E-04	8.47E-03
RUFY2	Negative regulation of NOTCH4 signaling	19/1642	54/10891	2.02E-04	8.47E-03
RUFY2	BBSome-mediated cargo-targeting to cilium	11/1642	23/10891	2.06E-04	8.47E-03
RUFY2	APC/C:Cdc20 mediated degradation of mitotic proteins	24/1642	76/10891	2.16E-04	8.60E-03
RUFY2	Antigen processing: Ubiquitination & Proteasome degradation	70/1642	309/10891	2.22E-04	8.63E-03
RUFY2	PIP3 activates AKT signaling	62/1642	267/10891	2.46E-04	9.32E-03
RUFY2	G1/S DNA Damage Checkpoints	22/1642	68/10891	2.65E-04	9.46E-03
RUFY2	Regulation of RUNX3 expression and activity	19/1642	55/10891	2.66E-04	9.46E-03
RUFY2	Activation of APC/C and APC/C:Cdc20 mediated degradation of mitotic proteins	24/1642	77/10891	2.69E-04	9.46E-03
RUFY2	Autodegradation of Cdh1 by Cdh1:APC/C	21/1642	64/10891	2.92E-04	1.00E-02
RUFY2	SCF(Skp2)-mediated degradation of p27/p21	20/1642	60/10891	3.19E-04	1.06E-02
RUFY2	Regulation of PTEN stability and activity	22/1642	69/10891	3.35E-04	1.06E-02
RUFY2	Cyclin E associated events during G1/S transition	25/1642	83/10891	3.65E-04	1.06E-02
RUFY2	Ubiquitin Mediated Degradation of Phosphorylated Cdc25A	18/1642	52/10891	3.72E-04	1.06E-02
RUFY2	p53-Independent DNA Damage Response	18/1642	52/10891	3.72E-04	1.06E-02
RUFY2	p53-Independent G1/S DNA damage checkpoint	18/1642	52/10891	3.72E-04	1.06E-02
RUFY2	Ubiquitin-dependent degradation of Cyclin D	18/1642	52/10891	3.72E-04	1.06E-02
RUFY2	APC:Cdc20 mediated degradation of cell cycle proteins prior to satisfation of the cell cycle checkpoint	23/1642	74/10891	3.75E-04	1.06E-02
RUFY2	Intracellular signaling by second messengers	69/1642	309/10891	3.80E-04	1.06E-02
RUFY2	Beta-catenin independent WNT signaling	38/1642	146/10891	3.82E-04	1.06E-02
RUFY2	Regulation of mRNA stability by proteins that bind AU-rich elements	26/1642	88/10891	3.92E-04	1.07E-02

RUFY2	Defective CFTR causes cystic fibrosis	20/1642	61/10891	4.08E-04	1.10E-02
RUFY2	p53-Dependent G1 DNA Damage Response	21/1642	66/10891	4.68E-04	1.21E-02
RUFY2	p53-Dependent G1/S DNA damage checkpoint	21/1642	66/10891	4.68E-04	1.21E-02
RUFY2	Protein localization	41/1642	163/10891	4.94E-04	1.26E-02
RUFY2	Cross-presentation of soluble exogenous antigens (endosomes)	17/1642	49/10891	5.21E-04	1.29E-02
RUFY2	Formation of ATP by chemiosmotic coupling	9/1642	18/10891	5.25E-04	1.29E-02
RUFY2	Cyclin A:Cdk2-associated events at S phase entry	25/1642	85/10891	5.43E-04	1.31E-02
RUFY2	RAB GEFs exchange GTP for GDP on RABs	26/1642	90/10891	5.75E-04	1.37E-02
RUFY2	Detoxification of Reactive Oxygen Species	14/1642	37/10891	5.91E-04	1.39E-02
RUFY2	FBXL7 down-regulates AURKA during mitotic entry and in early mitosis	18/1642	54/10891	6.27E-04	1.45E-02
RUFY2	Synthesis of substrates in N-glycan biosythesis	20/1642	63/10891	6.54E-04	1.49E-02
RUFY2	Regulation of activated PAK-2p34 by proteasome mediated degradation	17/1642	50/10891	6.81E-04	1.52E-02
RUFY2	Metabolism of polyamines	19/1642	59/10891	7.26E-04	1.58E-02
RUFY2	Hh mutants abrogate ligand secretion	19/1642	59/10891	7.26E-04	1.58E-02
RUFY2	Neddylation	55/1642	240/10891	7.53E-04	1.61E-02
RUFY2	EPHB-mediated forward signaling	15/1642	42/10891	7.76E-04	1.62E-02
RUFY2	Cdc20:Phospho-APC/C mediated degradation of Cyclin A	22/1642	73/10891	7.97E-04	1.62E-02
RUFY2	CDK-mediated phosphorylation and removal of Cdc6	22/1642	73/10891	7.97E-04	1.62E-02
RUFY2	SCF-beta-TrCP mediated degradation of Emi1	18/1642	55/10891	8.02E-04	1.62E-02
RUFY2	Asymmetric localization of PCP proteins	20/1642	64/10891	8.19E-04	1.63E-02
RUFY2	EPH-Ephrin signaling	26/1642	92/10891	8.29E-04	1.63E-02
RUFY2	APC/C-mediated degradation of cell cycle proteins	25/1642	88/10891	9.52E-04	1.83E-02
RUFY2	Regulation of mitotic cell cycle	25/1642	88/10891	9.52E-04	1.83E-02
RUFY2	Hedgehog ligand biogenesis	20/1642	65/10891	1.02E-03	1.87E-02
RUFY2	AUF1 (hnRNP D0) binds and destabilizes mRNA	18/1642	56/10891	1.02E-03	1.87E-02
RUFY2	Hh mutants are degraded by ERAD	18/1642	56/10891	1.02E-03	1.87E-02
RUFY2	Telomere Extension By Telomerase	10/1642	23/10891	1.03E-03	1.87E-02
RUFY2	Vpu mediated degradation of CD4	17/1642	52/10891	1.13E-03	1.99E-02
RUFY2	GSK3B and BTRC:CUL1-mediated-degradation of NFE2L2	17/1642	52/10891	1.13E-03	1.99E-02
RUFY2	MAPK6/MAPK4 signaling	25/1642	89/10891	1.14E-03	1.99E-02
RUFY2	Apoptosis	43/1642	180/10891	1.14E-03	1.99E-02
RUFY2	Rab regulation of trafficking	32/1642	124/10891	1.24E-03	2.13E-02
RUFY2	Translation of Structural Proteins	18/1642	57/10891	1.28E-03	2.15E-02
RUFY2	Late SARS-CoV-2 Infection Events	18/1642	57/10891	1.28E-03	2.15E-02
RUFY2	Hedgehog 'on' state	24/1642	85/10891	1.31E-03	2.17E-02
RUFY2	Regulation of Apoptosis	17/1642	53/10891	1.43E-03	2.34E-02

RUFY2	mTORC1-mediated signalling	10/1642	24/10891	1.52E-03	2.47E-02
RUFY2	Activation of NF-kappaB in B cells	20/1642	67/10891	1.54E-03	2.48E-02
RUFY2	Regulation of RAS by GAPs	20/1642	68/10891	1.88E-03	2.99E-02
RUFY2	Signaling by the B Cell Receptor (BCR)	29/1642	112/10891	1.94E-03	3.05E-02
RUFY2	Signaling by Hedgehog	36/1642	149/10891	2.26E-03	3.51E-02
RUFY2	Cargo trafficking to the periciliary membrane	16/1642	51/10891	2.52E-03	3.88E-02
RUFY2	HIV Infection	51/1642	231/10891	2.68E-03	4.08E-02
RUFY2	Gluconeogenesis	12/1642	34/10891	2.87E-03	4.33E-02
RUFY2	Neutrophil degranulation	95/1642	482/10891	2.92E-03	4.35E-02
RUFY2	Mitochondrial biogenesis	25/1642	95/10891	3.05E-03	4.50E-02
RUFY2	Oxygen-dependent proline hydroxylation of Hypoxia-inducible Factor Alpha	19/1642	66/10891	3.16E-03	4.62E-02
RUFY2	RHO GTPase cycle	89/1642	449/10891	3.30E-03	4.78E-02
RUFY2	Degradation of DVL	17/1642	57/10891	3.41E-03	4.89E-02
RUFY2	Regulation of APC/C activators between G1/S and early anaphase	22/1642	81/10891	3.47E-03	4.92E-02
RUFY2	Cilium Assembly	45/1642	201/10891	3.50E-03	4.92E-02
SBF2	RHO GTPase cycle	200/3231	449/10891	6.95E-12	1.07E-08
SBF2	Diseases of signal transduction by growth factor receptors and second messengers	190/3231	433/10891	1.15E-10	8.86E-08
SBF2	Chromatin modifying enzymes	126/3231	274/10891	5.74E-09	2.21E-06
SBF2	Chromatin organization	126/3231	274/10891	5.74E-09	2.21E-06
SBF2	DNA Repair	146/3231	336/10891	3.69E-08	1.14E-05
SBF2	Intra-Golgi and retrograde Golgi-to-ER traffic	92/3231	202/10891	1.09E-06	2.80E-04
SBF2	PTEN Regulation	68/3231	140/10891	1.72E-06	3.10E-04
SBF2	Intracellular signaling by second messengers	130/3231	309/10891	1.74E-06	3.10E-04
SBF2	SUMOylation	86/3231	188/10891	1.94E-06	3.10E-04
SBF2	Class I MHC mediated antigen processing & presentation	155/3231	381/10891	2.01E-06	3.10E-04
SBF2	RAC1 GTPase cycle	84/3231	185/10891	3.67E-06	5.15E-04
SBF2	ER to Golgi Anterograde Transport	72/3231	154/10891	5.01E-06	6.44E-04
SBF2	VEGFA-VEGFR2 Pathway	50/3231	99/10891	1.01E-05	1.15E-03
SBF2	PIP3 activates AKT signaling	112/3231	267/10891	1.05E-05	1.15E-03
SBF2	SUMO E3 ligases SUMOylate target proteins	81/3231	182/10891	1.37E-05	1.41E-03
SBF2	Autophagy	69/3231	151/10891	2.03E-05	1.96E-03
SBF2	Beta-catenin independent WNT signaling	67/3231	146/10891	2.25E-05	2.04E-03
SBF2	RAC3 GTPase cycle	47/3231	94/10891	2.59E-05	2.18E-03
SBF2	Macroautophagy	63/3231	136/10891	2.73E-05	2.18E-03
SBF2	Regulation of cholesterol biosynthesis by SREBP (SREBF)	31/3231	55/10891	3.21E-05	2.18E-03
SBF2	Signaling by ALK in cancer	31/3231	55/10891	3.21E-05	2.18E-03
SBF2	Signaling by ALK fusions and activated point mutants	31/3231	55/10891	3.21E-05	2.18E-03

SBF2	SUMOylation of DNA damage response and repair proteins	40/3231	77/10891	3.31E-05	2.18E-03
SBF2	Signaling by cytosolic FGFR1 fusion mutants	14/3231	18/10891	3.38E-05	2.18E-03
SBF2	Clathrin-mediated endocytosis	66/3231	146/10891	4.63E-05	2.85E-03
SBF2	Mitotic Prometaphase	87/3231	204/10891	4.80E-05	2.85E-03
SBF2	Transport to the Golgi and subsequent modification	80/3231	185/10891	5.36E-05	3.06E-03
SBF2	Organelle biogenesis and maintenance	119/3231	296/10891	5.67E-05	3.12E-03
SBF2	Downstream signal transduction	19/3231	29/10891	6.85E-05	3.64E-03
SBF2	Antigen processing: Ubiquitination & Proteasome degradation	123/3231	309/10891	7.20E-05	3.70E-03
SBF2	Asparagine N-linked glycosylation	121/3231	304/10891	8.25E-05	3.99E-03
SBF2	Signaling by VEGF	51/3231	108/10891	8.36E-05	3.99E-03
SBF2	Ca2+ pathway	33/3231	62/10891	8.53E-05	3.99E-03
SBF2	MTOR signalling	24/3231	41/10891	1.11E-04	4.92E-03
SBF2	RAC2 GTPase cycle	43/3231	88/10891	1.12E-04	4.92E-03
SBF2	RHO GTPase Effectors	128/3231	327/10891	1.25E-04	5.26E-03
SBF2	Cohesin Loading onto Chromatin	9/3231	10/10891	1.30E-04	5.26E-03
SBF2	Regulation of FOXO transcriptional activity by acetylation	9/3231	10/10891	1.30E-04	5.26E-03
SBF2	Regulation of PTEN gene transcription	32/3231	61/10891	1.56E-04	6.18E-03
SBF2	M Phase	158/3231	418/10891	1.68E-04	6.50E-03
SBF2	Rab regulation of trafficking	56/3231	124/10891	1.74E-04	6.56E-03
SBF2	RHOG GTPase cycle	37/3231	74/10891	1.82E-04	6.56E-03
SBF2	Transcriptional Regulation by TP53	139/3231	362/10891	1.83E-04	6.56E-03
SBF2	CDC42 GTPase cycle	67/3231	155/10891	2.13E-04	7.38E-03
SBF2	Regulation of MECP2 expression and activity	20/3231	33/10891	2.15E-04	7.38E-03
SBF2	Processing of Capped Intron-Containing Pre-mRNA	99/3231	246/10891	2.21E-04	7.41E-03
SBF2	RORA activates gene expression	13/3231	18/10891	2.35E-04	7.71E-03
SBF2	Potential therapeutics for SARS	46/3231	99/10891	2.91E-04	9.22E-03
SBF2	Energy dependent regulation of mTOR by LKB1-AMPK	18/3231	29/10891	2.93E-04	9.22E-03
SBF2	COPII-mediated vesicle transport	34/3231	68/10891	3.28E-04	1.01E-02
SBF2	SARS-CoV Infections	142/3231	376/10891	3.70E-04	1.12E-02
SBF2	EPH-Ephrin signaling	43/3231	92/10891	3.87E-04	1.15E-02
SBF2	Golgi-to-ER retrograde transport	58/3231	133/10891	4.17E-04	1.22E-02
SBF2	Cilium Assembly	82/3231	201/10891	4.48E-04	1.28E-02
SBF2	Cargo recognition for clathrin-mediated endocytosis	48/3231	106/10891	4.56E-04	1.28E-02
SBF2	RAB GEFs exchange GTP for GDP on RABs	42/3231	90/10891	4.68E-04	1.29E-02
SBF2	Cytosolic sensors of pathogen-associated DNA	32/3231	64/10891	4.86E-04	1.32E-02
SBF2	Signaling by TGF-beta Receptor Complex	43/3231	93/10891	5.15E-04	1.32E-02

SBF2	Establishment of Sister Chromatid Cohesion	9/3231	11/10891	5.23E-04	1.32E-02
SBF2	NR1H3 & NR1H2 regulate gene expression linked to cholesterol transport and efflux	21/3231	37/10891	5.30E-04	1.32E-02
SBF2	ISG15 antiviral mechanism	35/3231	72/10891	5.32E-04	1.32E-02
SBF2	trans-Golgi Network Vesicle Budding	35/3231	72/10891	5.32E-04	1.32E-02
SBF2	Mitotic Telophase/Cytokinesis	10/3231	13/10891	5.86E-04	1.43E-02
SBF2	Heme signaling	25/3231	47/10891	6.12E-04	1.45E-02
SBF2	Signaling by WNT	126/3231	332/10891	6.18E-04	1.45E-02
SBF2	Nuclear Envelope Breakdown	27/3231	52/10891	6.19E-04	1.45E-02
SBF2	Deubiquitination	114/3231	298/10891	7.86E-04	1.81E-02
SBF2	mTORC1-mediated signalling	15/3231	24/10891	8.53E-04	1.94E-02
SBF2	RHOC GTPase cycle	35/3231	74/10891	1.00E-03	2.19E-02
SBF2	Recruitment of mitotic centrosome proteins and complexes	38/3231	82/10891	1.01E-03	2.19E-02
SBF2	Centrosome maturation	38/3231	82/10891	1.01E-03	2.19E-02
SBF2	Golgi Associated Vesicle Biogenesis	28/3231	56/10891	1.07E-03	2.26E-02
SBF2	Signaling by Hippo	13/3231	20/10891	1.13E-03	2.26E-02
SBF2	RHO GTPases Activate ROCKs	13/3231	20/10891	1.13E-03	2.26E-02
SBF2	Signaling by KIT in disease	13/3231	20/10891	1.13E-03	2.26E-02
SBF2	Signaling by phosphorylated juxtamembrane, extracellular and kinase domain KIT mutants	13/3231	20/10891	1.13E-03	2.26E-02
SBF2	Signaling by PDGFR in disease	13/3231	20/10891	1.13E-03	2.26E-02
SBF2	Regulation of PLK1 Activity at G2/M Transition	40/3231	88/10891	1.20E-03	2.37E-02
SBF2	Antiviral mechanism by IFN-stimulated genes	37/3231	80/10891	1.22E-03	2.39E-02
SBF2	RHOQ GTPase cycle	29/3231	59/10891	1.25E-03	2.40E-02
SBF2	Integrin signaling	16/3231	27/10891	1.30E-03	2.49E-02
SBF2	RHOA GTPase cycle	61/3231	147/10891	1.39E-03	2.61E-02
SBF2	Regulation of gene expression in late stage (branching morphogenesis) pancreatic bud precursor cells	11/3231	16/10891	1.40E-03	2.61E-02
SBF2	Loss of Nlp from mitotic centrosomes	33/3231	70/10891	1.48E-03	2.65E-02
SBF2	Loss of proteins required for interphase microtubule organization from the centrosome	33/3231	70/10891	1.48E-03	2.65E-02
SBF2	RHOB GTPase cycle	33/3231	70/10891	1.48E-03	2.65E-02
SBF2	Prolonged ERK activation events	10/3231	14/10891	1.50E-03	2.65E-02
SBF2	Signaling by TGFB family members	52/3231	122/10891	1.51E-03	2.65E-02
SBF2	Constitutive Signaling by AKT1 E17K in Cancer	15/3231	25/10891	1.55E-03	2.68E-02
SBF2	Nucleotide Excision Repair	48/3231	111/10891	1.56E-03	2.68E-02
SBF2	mRNA Splicing - Major Pathway	74/3231	185/10891	1.58E-03	2.68E-02
SBF2	Activation of gene expression by SREBF (SREBP)	22/3231	42/10891	1.67E-03	2.78E-02
SBF2	Opioid Signalling	41/3231	92/10891	1.68E-03	2.78E-02
SBF2	Phospholipid metabolism	83/3231	212/10891	1.79E-03	2.94E-02
SBF2	Signaling by ERBB2	25/3231	50/10891	1.95E-03	3.14E-02

SBF2	Inositol phosphate metabolism	25/3231	50/10891	1.95E-03	3.14E-02
SBF2	NS1 Mediated Effects on Host Pathways	21/3231	40/10891	2.04E-03	3.20E-02
SBF2	Signaling by NOTCH1 PEST Domain Mutants in Cancer	28/3231	58/10891	2.12E-03	3.20E-02
SBF2	Signaling by NOTCH1 in Cancer	28/3231	58/10891	2.12E-03	3.20E-02
SBF2	Constitutive Signaling by NOTCH1 PEST Domain Mutants	28/3231	58/10891	2.12E-03	3.20E-02
SBF2	Signaling by NOTCH1 HD+PEST Domain Mutants in Cancer	28/3231	58/10891	2.12E-03	3.20E-02
SBF2	Constitutive Signaling by NOTCH1 HD+PEST Domain Mutants	28/3231	58/10891	2.12E-03	3.20E-02
SBF2	Signaling by NOTCH1	34/3231	74/10891	2.17E-03	3.25E-02
SBF2	Notch-HLH transcription pathway	16/3231	28/10891	2.21E-03	3.28E-02
SBF2	DNA Damage Bypass	24/3231	48/10891	2.39E-03	3.51E-02
SBF2	Vpr-mediated nuclear import of PICs	18/3231	33/10891	2.42E-03	3.52E-02
SBF2	Signaling by SCF-KIT	22/3231	43/10891	2.47E-03	3.56E-02
SBF2	RUNX1 interacts with co-factors whose precise effect on RUNX1 targets is not known	20/3231	38/10891	2.49E-03	3.56E-02
SBF2	Processive synthesis on the C-strand of the telomere	12/3231	19/10891	2.52E-03	3.57E-02
SBF2	G-protein mediated events	27/3231	56/10891	2.58E-03	3.62E-02
SBF2	Recruitment of NuMA to mitotic centrosomes	41/3231	94/10891	2.74E-03	3.78E-02
SBF2	Transcriptional activity of SMAD2/SMAD3:SMAD4 heterotrimer	25/3231	51/10891	2.77E-03	3.78E-02
SBF2	COPI-independent Golgi-to-ER retrograde traffic	25/3231	51/10891	2.77E-03	3.78E-02
SBF2	Non-integrin membrane-ECM interactions	28/3231	59/10891	2.90E-03	3.91E-02
SBF2	Removal of the Flap Intermediate from the C-strand	11/3231	17/10891	2.91E-03	3.91E-02
SBF2	Transport of Ribonucleoproteins into the Host Nucleus	17/3231	31/10891	2.95E-03	3.92E-02
SBF2	RNA Polymerase III Transcription	21/3231	41/10891	3.03E-03	3.95E-02
SBF2	RNA Polymerase III Abortive And Retractive Initiation	21/3231	41/10891	3.03E-03	3.95E-02
SBF2	Interactions of Vpr with host cellular proteins	19/3231	36/10891	3.05E-03	3.95E-02
SBF2	Regulation of TP53 Activity	64/3231	160/10891	3.15E-03	4.05E-02
SBF2	Deadenylation of mRNA	14/3231	24/10891	3.21E-03	4.06E-02
SBF2	Growth hormone receptor signaling	14/3231	24/10891	3.21E-03	4.06E-02
SBF2	Translation of Replicase and Assembly of the Replication Transcription Complex	10/3231	15/10891	3.31E-03	4.16E-02
SBF2	Retrograde transport at the Trans-Golgi-Network	24/3231	49/10891	3.38E-03	4.17E-02
SBF2	Signaling by NOTCH3	24/3231	49/10891	3.38E-03	4.17E-02
SBF2	Neddylation	91/3231	240/10891	3.41E-03	4.17E-02
SBF2	AURKA Activation by TPX2	33/3231	73/10891	3.46E-03	4.20E-02

SBF2	Global Genome Nucleotide Excision Repair (GG-NER)	37/3231	84/10891	3.52E-03	4.24E-02
SBF2	Antigen Presentation: Folding, assembly and peptide loading of class I MHC	16/3231	29/10891	3.59E-03	4.27E-02
SBF2	Signaling by the B Cell Receptor (BCR)	47/3231	112/10891	3.60E-03	4.27E-02
SBF2	mRNA Splicing	75/3231	193/10891	3.63E-03	4.27E-02
SBF2	CLEC7A (Dectin-1) induces NFAT activation	9/3231	13/10891	3.69E-03	4.31E-02
SBF2	ESR-mediated signaling	85/3231	223/10891	3.85E-03	4.46E-02
SBF2	Interleukin-6 signaling	8/3231	11/10891	3.95E-03	4.52E-02
SBF2	Signaling by Leptin	8/3231	11/10891	3.95E-03	4.52E-02
SBF2	tRNA processing	45/3231	107/10891	4.12E-03	4.66E-02
SBF2	NR1H2 and NR1H3-mediated signaling	23/3231	47/10891	4.14E-03	4.66E-02
SBF2	Signaling by FGFR in disease	29/3231	63/10891	4.31E-03	4.82E-02
SBF2	RHOF GTPase cycle	21/3231	42/10891	4.38E-03	4.86E-02
SBF2	Signaling by NOTCH	89/3231	236/10891	4.46E-03	4.92E-02
SLC25A16	Eukaryotic Translation Termination	51/1177	93/10891	1.14E-25	1.64E-22
SLC25A16	Nonsense Mediated Decay (NMD) independent of the Exon Junction Complex (EJC)	51/1177	95/10891	4.33E-25	3.13E-22
SLC25A16	Eukaryotic Translation Elongation	50/1177	93/10891	1.16E-24	5.60E-22
SLC25A16	Peptide chain elongation	48/1177	89/10891	8.38E-24	3.03E-21
SLC25A16	Nonsense-Mediated Decay (NMD)	54/1177	115/10891	7.79E-23	1.70E-20
SLC25A16	Nonsense Mediated Decay (NMD) enhanced by the Exon Junction Complex (EJC)	54/1177	115/10891	7.79E-23	1.70E-20
SLC25A16	Viral mRNA Translation	47/1177	89/10891	8.24E-23	1.70E-20
SLC25A16	Selenocysteine synthesis	48/1177	93/10891	1.07E-22	1.93E-20
SLC25A16	SRP-dependent cotranslational protein targeting to membrane	53/1177	112/10891	1.24E-22	1.99E-20
SLC25A16	Formation of a pool of free 40S subunits	50/1177	101/10891	1.56E-22	2.05E-20
SLC25A16	Response of EIF2AK4 (GCN2) to amino acid deficiency	50/1177	101/10891	1.56E-22	2.05E-20
SLC25A16	L13a-mediated translational silencing of Ceruloplasmin expression	52/1177	111/10891	5.64E-22	6.79E-20
SLC25A16	GTP hydrolysis and joining of the 60S ribosomal subunit	52/1177	112/10891	9.45E-22	1.05E-19
SLC25A16	Selenoamino acid metabolism	52/1177	118/10891	1.80E-20	1.85E-18
SLC25A16	Regulation of expression of SLITs and ROBOs	64/1177	171/10891	2.55E-20	2.43E-18
SLC25A16	Eukaryotic Translation Initiation	52/1177	119/10891	2.86E-20	2.43E-18
SLC25A16	Cap-dependent Translation Initiation	52/1177	119/10891	2.86E-20	2.43E-18
SLC25A16	Translation	86/1177	291/10891	4.49E-19	3.60E-17
SLC25A16	Influenza Viral RNA Transcription and Replication	53/1177	135/10891	4.14E-18	3.15E-16
SLC25A16	Signaling by ROBO receptors	70/1177	218/10891	7.39E-18	5.34E-16
SLC25A16	Cellular response to starvation	57/1177	156/10891	1.16E-17	7.96E-16
SLC25A16	Influenza Infection	56/1177	156/10891	5.70E-17	3.74E-15

SLC25A16	Major pathway of rRNA processing in the nucleolus and cytosol	59/1177	184/10891	3.28E-15	2.06E-13
SLC25A16	rRNA processing	62/1177	204/10891	1.08E-14	6.49E-13
SLC25A16	rRNA processing in the nucleus and cytosol	59/1177	194/10891	4.66E-14	2.70E-12
SLC25A16	Metabolism of amino acids and derivatives	85/1177	374/10891	1.13E-11	6.27E-10
SLC25A16	Translation initiation complex formation	19/1177	58/10891	5.78E-06	2.98E-04
SLC25A16	Ribosomal scanning and start codon recognition	19/1177	58/10891	5.78E-06	2.98E-04
SLC25A16	Activation of the mRNA upon binding of the cap-binding complex and eIFs, and subsequent binding to 43S	19/1177	59/10891	7.67E-06	3.82E-04
SLC25A16	Formation of the ternary complex, and subsequently, the 43S complex	17/1177	51/10891	1.37E-05	6.61E-04
SLC25A16	Cellular response to heat stress	24/1177	89/10891	1.61E-05	7.51E-04
SLC25A16	HIV Infection	46/1177	231/10891	2.75E-05	1.24E-03
SLC25A16	Regulation of HSF1-mediated heat shock response	19/1177	69/10891	8.86E-05	3.88E-03
SLC25A16	Late Phase of HIV Life Cycle	30/1177	136/10891	1.01E-04	4.28E-03
SLC25A16	Neurexins and neuroligins	16/1177	55/10891	1.56E-04	6.43E-03
SLC25A16	SARS-CoV-2 modulates host translation machinery	15/1177	50/10891	1.70E-04	6.83E-03
SLC25A16	HIV Life Cycle	31/1177	149/10891	2.40E-04	9.38E-03
SLC25A16	Attenuation phase	7/1177	14/10891	2.92E-04	1.11E-02
SLC25A16	Dopamine Neurotransmitter Release Cycle	9/1177	23/10891	3.90E-04	1.44E-02
SLC25A16	ER to Golgi Anterograde Transport	31/1177	154/10891	4.41E-04	1.59E-02
SLC25A16	AUF1 (hnRNP D0) binds and destabilizes mRNA	15/1177	56/10891	6.60E-04	2.32E-02
SLC25A16	Protein-protein interactions at synapses	20/1177	86/10891	6.84E-04	2.32E-02
SLC25A16	Apoptotic factor-mediated response	8/1177	20/10891	6.95E-04	2.32E-02
SLC25A16	COPII-mediated vesicle transport	17/1177	68/10891	7.07E-04	2.32E-02
SLC25A16	Apoptosis	34/1177	180/10891	8.04E-04	2.58E-02
SLC25A16	Programmed Cell Death	38/1177	210/10891	9.59E-04	3.01E-02
SLC25A16	Cytochrome c-mediated apoptotic response	6/1177	13/10891	1.38E-03	4.13E-02
SLC25A16	ISG15 antiviral mechanism	17/1177	72/10891	1.41E-03	4.13E-02
SLC25A16	Degradation of GLI2 by the proteasome	15/1177	60/10891	1.43E-03	4.13E-02
SLC25A16	GLI3 is processed to GLI3R by the proteasome	15/1177	60/10891	1.43E-03	4.13E-02
SLC25A16	Regulation of RUNX2 expression and activity	17/1177	73/10891	1.65E-03	4.69E-02
SMPD3	GSK3B and BTRC:CUL1-mediated-degradation of NFE2L2	45/4222	52/10891	1.28E-12	1.99E-09
SMPD3	KEAP1-NFE2L2 pathway	74/4222	102/10891	3.76E-12	2.74E-09
SMPD3	Regulation of Apoptosis	45/4222	53/10891	5.30E-12	2.74E-09
SMPD3	Dectin-1 mediated noncanonical NF-kB signaling	49/4222	60/10891	1.08E-11	3.69E-09
SMPD3	Cross-presentation of soluble exogenous antigens (endosomes)	42/4222	49/10891	1.45E-11	3.69E-09
SMPD3	Programmed Cell Death	129/4222	210/10891	1.74E-11	3.69E-09

SMPD3	Host Interactions of HIV factors	88/4222	130/10891	1.84E-11	3.69E-09
SMPD3	FBXL7 down-regulates AURKA during mitotic entry and in early mitosis	45/4222	54/10891	1.98E-11	3.69E-09
SMPD3	NIK-->noncanonical NF-kB signaling	48/4222	59/10891	2.30E-11	3.69E-09
SMPD3	Autodegradation of Cdh1 by Cdh1:APC/C	51/4222	64/10891	2.38E-11	3.69E-09
SMPD3	ABC-family proteins mediated transport	73/4222	103/10891	3.33E-11	4.70E-09
SMPD3	Transcriptional regulation by RUNX3	69/4222	96/10891	4.10E-11	5.25E-09
SMPD3	G2/M Transition	121/4222	196/10891	4.65E-11	5.25E-09
SMPD3	Downstream TCR signaling	70/4222	98/10891	4.74E-11	5.25E-09
SMPD3	Regulation of activated PAK-2p34 by proteasome mediated degradation	42/4222	50/10891	5.65E-11	5.84E-09
SMPD3	SCF(Skp2)-mediated degradation of p27/p21	48/4222	60/10891	7.18E-11	6.55E-09
SMPD3	GLI3 is processed to GLI3R by the proteasome	48/4222	60/10891	7.18E-11	6.55E-09
SMPD3	Nuclear events mediated by NFE2L2	59/4222	79/10891	8.23E-11	6.91E-09
SMPD3	Regulation of mRNA stability by proteins that bind AU-rich elements	64/4222	88/10891	9.11E-11	6.91E-09
SMPD3	Ubiquitin-dependent degradation of Cyclin D	43/4222	52/10891	9.29E-11	6.91E-09
SMPD3	Activation of NF-kappaB in B cells	52/4222	67/10891	9.35E-11	6.91E-09
SMPD3	Mitotic G2-G2/M phases	121/4222	198/10891	1.20E-10	8.35E-09
SMPD3	Regulation of PTEN stability and activity	53/4222	69/10891	1.24E-10	8.35E-09
SMPD3	HIV Infection	137/4222	231/10891	1.44E-10	8.65E-09
SMPD3	Vif-mediated degradation of APOBEC3G	44/4222	54/10891	1.45E-10	8.65E-09
SMPD3	Negative regulation of NOTCH4 signaling	44/4222	54/10891	1.45E-10	8.65E-09
SMPD3	RHO GTPase cycle	239/4222	449/10891	1.63E-10	9.36E-09
SMPD3	CDK-mediated phosphorylation and removal of Cdc6	55/4222	73/10891	2.03E-10	1.11E-08
SMPD3	Mitotic Anaphase	139/4222	236/10891	2.08E-10	1.11E-08
SMPD3	APC/C:Cdc20 mediated degradation of Securin	52/4222	68/10891	2.48E-10	1.28E-08
SMPD3	The role of GTSE1 in G2/M progression after G2 checkpoint	57/4222	77/10891	3.11E-10	1.52E-08
SMPD3	Mitotic Metaphase and Anaphase	139/4222	237/10891	3.13E-10	1.52E-08
SMPD3	Degradation of GLI1 by the proteasome	47/4222	60/10891	4.31E-10	1.97E-08
SMPD3	Degradation of GLI2 by the proteasome	47/4222	60/10891	4.31E-10	1.97E-08
SMPD3	Degradation of AXIN	44/4222	55/10891	4.52E-10	2.00E-08
SMPD3	Degradation of beta-catenin by the destruction complex	60/4222	83/10891	5.28E-10	2.27E-08
SMPD3	Hedgehog 'on' state	61/4222	85/10891	6.15E-10	2.34E-08
SMPD3	Stabilization of p53	45/4222	57/10891	6.41E-10	2.34E-08
SMPD3	Vpu mediated degradation of CD4	42/4222	52/10891	6.49E-10	2.34E-08
SMPD3	Autodegradation of the E3 ubiquitin ligase COP1	42/4222	52/10891	6.49E-10	2.34E-08
SMPD3	Ubiquitin Mediated Degradation of Phosphorylated Cdc25A	42/4222	52/10891	6.49E-10	2.34E-08
SMPD3	p53-Independent DNA Damage Response	42/4222	52/10891	6.49E-10	2.34E-08

SMPD3	p53-Independent G1/S DNA damage checkpoint	42/4222	52/10891	6.49E-10	2.34E-08
SMPD3	ABC transporter disorders	57/4222	78/10891	7.20E-10	2.49E-08
SMPD3	PTEN Regulation	90/4222	140/10891	7.21E-10	2.49E-08
SMPD3	Apoptosis	110/4222	180/10891	8.35E-10	2.81E-08
SMPD3	Cdc20:Phospho-APC/C mediated degradation of Cyclin A	54/4222	73/10891	9.57E-10	3.16E-08
SMPD3	Oxygen-dependent proline hydroxylation of Hypoxia-inducible Factor Alpha	50/4222	66/10891	9.80E-10	3.17E-08
SMPD3	Defective CFTR causes cystic fibrosis	47/4222	61/10891	1.17E-09	3.71E-08
SMPD3	AUF1 (hnRNP D0) binds and destabilizes mRNA	44/4222	56/10891	1.32E-09	4.09E-08
SMPD3	CLEC7A (Dectin-1) signaling	69/4222	101/10891	1.51E-09	4.53E-08
SMPD3	TCR signaling	79/4222	120/10891	1.52E-09	4.53E-08
SMPD3	Diseases of signal transduction by growth factor receptors and second messengers	228/4222	433/10891	1.59E-09	4.66E-08
SMPD3	Regulation of APC/C activators between G1/S and early anaphase	58/4222	81/10891	1.87E-09	5.27E-08
SMPD3	Switching of origins to a post-replicative state	64/4222	92/10891	1.87E-09	5.27E-08
SMPD3	Class I MHC mediated antigen processing & presentation	204/4222	381/10891	1.95E-09	5.39E-08
SMPD3	Processing of Capped Intron-Containing Pre-mRNA	141/4222	246/10891	2.05E-09	5.57E-08
SMPD3	Downstream signaling events of B Cell Receptor (BCR)	59/4222	83/10891	2.15E-09	5.71E-08
SMPD3	APC/C:Cdh1 mediated degradation of Cdc20 and other APC/C:Cdh1 targeted proteins in late mitosis/early G1	54/4222	74/10891	2.21E-09	5.71E-08
SMPD3	APC:Cdc20 mediated degradation of cell cycle proteins prior to satisfation of the cell cycle checkpoint	54/4222	74/10891	2.21E-09	5.71E-08
SMPD3	SCF-beta-TrCP mediated degradation of Emi1	43/4222	55/10891	2.70E-09	6.75E-08
SMPD3	Regulation of RUNX3 expression and activity	43/4222	55/10891	2.70E-09	6.75E-08
SMPD3	Separation of Sister Chromatids	114/4222	191/10891	3.01E-09	7.42E-08
SMPD3	M Phase	220/4222	418/10891	3.27E-09	7.93E-08
SMPD3	Degradation of DVL	44/4222	57/10891	3.61E-09	8.61E-08
SMPD3	FCERI mediated NF-kB activation	58/4222	82/10891	3.98E-09	9.22E-08
SMPD3	Signaling by NOTCH4	58/4222	82/10891	3.98E-09	9.22E-08
SMPD3	Hh mutants abrogate ligand secretion	45/4222	59/10891	4.70E-09	1.07E-07
SMPD3	Cellular response to hypoxia	54/4222	75/10891	4.92E-09	1.11E-07
SMPD3	Activation of APC/C and APC/C:Cdc20 mediated degradation of mitotic proteins	55/4222	77/10891	5.68E-09	1.26E-07
SMPD3	Hh mutants are degraded by ERAD	43/4222	56/10891	7.26E-09	1.59E-07
SMPD3	Synthesis of DNA	78/4222	121/10891	8.14E-09	1.75E-07
SMPD3	APC/C:Cdc20 mediated degradation of mitotic	54/4222	76/10891	1.06E-08	2.25E-07

	proteins				
SMPD3	Signaling by the B Cell Receptor (BCR)	73/4222	112/10891	1.21E-08	2.54E-07
SMPD3	Orc1 removal from chromatin	51/4222	71/10891	1.50E-08	3.10E-07
SMPD3	Fc epsilon receptor (FCERI) signaling	84/4222	134/10891	1.53E-08	3.13E-07
SMPD3	Beta-catenin independent WNT signaling	90/4222	146/10891	1.56E-08	3.14E-07
SMPD3	Regulation of RUNX2 expression and activity	52/4222	73/10891	1.73E-08	3.43E-07
SMPD3	p53-Dependent G1 DNA Damage Response	48/4222	66/10891	2.08E-08	4.03E-07
SMPD3	p53-Dependent G1/S DNA damage checkpoint	48/4222	66/10891	2.08E-08	4.03E-07
SMPD3	Hedgehog 'off' state	73/4222	113/10891	2.13E-08	4.04E-07
SMPD3	ER-Phagosome pathway	61/4222	90/10891	2.14E-08	4.04E-07
SMPD3	Interleukin-1 signaling	74/4222	115/10891	2.19E-08	4.10E-07
SMPD3	S Phase	98/4222	163/10891	2.28E-08	4.14E-07
SMPD3	PCP/CE pathway	62/4222	92/10891	2.29E-08	4.14E-07
SMPD3	Metabolism of polyamines	44/4222	59/10891	2.30E-08	4.14E-07
SMPD3	Antigen processing: Ubiquitination & Proteasome degradation	167/4222	309/10891	2.47E-08	4.38E-07
SMPD3	Signaling by Hedgehog	91/4222	149/10891	2.49E-08	4.38E-07
SMPD3	TNFR2 non-canonical NF-kB pathway	67/4222	102/10891	3.03E-08	5.28E-07
SMPD3	MAPK6/MAPK4 signaling	60/4222	89/10891	3.78E-08	6.52E-07
SMPD3	Hedgehog ligand biogenesis	47/4222	65/10891	3.94E-08	6.72E-07
SMPD3	mRNA Splicing - Major Pathway	108/4222	185/10891	4.18E-08	7.04E-07
SMPD3	Regulation of ornithine decarboxylase (ODC)	39/4222	51/10891	4.50E-08	7.50E-07
SMPD3	Antiviral mechanism by IFN-stimulated genes	55/4222	80/10891	4.93E-08	8.13E-07
SMPD3	APC/C-mediated degradation of cell cycle proteins	59/4222	88/10891	6.66E-08	1.08E-06
SMPD3	Regulation of mitotic cell cycle	59/4222	88/10891	6.66E-08	1.08E-06
SMPD3	Asymmetric localization of PCP proteins	46/4222	64/10891	7.43E-08	1.19E-06
SMPD3	mRNA Splicing	111/4222	193/10891	8.23E-08	1.30E-06
SMPD3	Neddylation	133/4222	240/10891	9.57E-08	1.49E-06
SMPD3	Regulation of RAS by GAPs	48/4222	68/10891	9.71E-08	1.49E-06
SMPD3	G1/S DNA Damage Checkpoints	48/4222	68/10891	9.71E-08	1.49E-06
SMPD3	Cyclin E associated events during G1/S transition	56/4222	83/10891	1.03E-07	1.57E-06
SMPD3	Antigen processing-Cross presentation	67/4222	105/10891	1.63E-07	2.45E-06
SMPD3	Transcriptional regulation by RUNX2	75/4222	121/10891	1.76E-07	2.63E-06
SMPD3	Cyclin A:Cdk2-associated events at S phase entry	56/4222	85/10891	3.52E-07	5.20E-06
SMPD3	Intracellular signaling by second messengers	162/4222	309/10891	5.45E-07	7.97E-06
SMPD3	RAC3 GTPase cycle	60/4222	94/10891	7.04E-07	1.02E-05
SMPD3	C-type lectin receptors (CLRs)	83/4222	142/10891	1.41E-06	2.02E-05
SMPD3	UCH proteinases	63/4222	102/10891	2.00E-06	2.84E-05
SMPD3	PIP3 activates AKT signaling	140/4222	267/10891	3.14E-06	4.43E-05
SMPD3	ISG15 antiviral mechanism	47/4222	72/10891	4.50E-06	6.29E-05
SMPD3	RAB GEFs exchange GTP for GDP on RABs	56/4222	90/10891	5.28E-06	7.31E-05
SMPD3	G1/S Transition	76/4222	131/10891	5.68E-06	7.80E-05

SMPD3	Autophagy	85/4222	151/10891	8.50E-06	1.16E-04
SMPD3	Macroautophagy	77/4222	136/10891	1.68E-05	2.25E-04
SMPD3	Interleukin-1 family signaling	85/4222	153/10891	1.68E-05	2.25E-04
SMPD3	RHOF GTPase cycle	30/4222	42/10891	1.75E-05	2.32E-04
SMPD3	Non-integrin membrane-ECM interactions	39/4222	59/10891	1.88E-05	2.47E-04
SMPD3	Rab regulation of trafficking	71/4222	124/10891	2.12E-05	2.76E-04
SMPD3	Deubiquitination	150/4222	298/10891	2.59E-05	3.35E-04
SMPD3	Cellular response to chemical stress	103/4222	194/10891	3.13E-05	4.01E-04
SMPD3	Cell Cycle Checkpoints	147/4222	293/10891	3.84E-05	4.89E-04
SMPD3	SARS-CoV-1 Infection	35/4222	53/10891	5.17E-05	6.50E-04
SMPD3	Signaling by TGF-beta Receptor Complex	55/4222	93/10891	5.19E-05	6.50E-04
SMPD3	Cytosolic tRNA aminoacylation	19/4222	24/10891	6.43E-05	7.97E-04
SMPD3	Folding of actin by CCT/TriC	10/4222	10/10891	7.61E-05	9.37E-04
SMPD3	Mitotic Prometaphase	106/4222	204/10891	7.75E-05	9.39E-04
SMPD3	RHOA GTPase cycle	80/4222	147/10891	7.75E-05	9.39E-04
SMPD3	Regulation of PLK1 Activity at G2/M Transition	52/4222	88/10891	8.50E-05	1.02E-03
SMPD3	Signaling by NOTCH	120/4222	236/10891	9.28E-05	1.11E-03
SMPD3	tRNA processing	61/4222	107/10891	9.37E-05	1.11E-03
SMPD3	Loss of Nlp from mitotic centrosomes	43/4222	70/10891	9.91E-05	1.15E-03
SMPD3	Loss of proteins required for interphase microtubule organization from the centrosome	43/4222	70/10891	9.91E-05	1.15E-03
SMPD3	RHOB GTPase cycle	43/4222	70/10891	9.91E-05	1.15E-03
SMPD3	Signaling by WNT	162/4222	332/10891	1.03E-04	1.18E-03
SMPD3	SARS-CoV Infections	181/4222	376/10891	1.06E-04	1.21E-03
SMPD3	Organelle biogenesis and maintenance	146/4222	296/10891	1.17E-04	1.33E-03
SMPD3	SUMOylation	98/4222	188/10891	1.21E-04	1.37E-03
SMPD3	Cellular response to heat stress	52/4222	89/10891	1.28E-04	1.43E-03
SMPD3	Mitotic G1 phase and G1/S transition	80/4222	149/10891	1.42E-04	1.57E-03
SMPD3	Selective autophagy	48/4222	81/10891	1.43E-04	1.57E-03
SMPD3	Ub-specific processing proteases	112/4222	220/10891	1.47E-04	1.61E-03
SMPD3	SUMOylation of DNA damage response and repair proteins	46/4222	77/10891	1.49E-04	1.62E-03
SMPD3	COPI-independent Golgi-to-ER retrograde traffic	33/4222	51/10891	1.53E-04	1.65E-03
SMPD3	Disorders of transmembrane transporters	92/4222	176/10891	1.70E-04	1.82E-03
SMPD3	RAC1 GTPase cycle	96/4222	185/10891	1.76E-04	1.87E-03
SMPD3	Recruitment of NuMA to mitotic centrosomes	54/4222	94/10891	1.77E-04	1.87E-03
SMPD3	Phospholipid metabolism	108/4222	212/10891	1.85E-04	1.94E-03
SMPD3	Recruitment of mitotic centrosome proteins and complexes	48/4222	82/10891	2.16E-04	2.23E-03
SMPD3	Centrosome maturation	48/4222	82/10891	2.16E-04	2.23E-03
SMPD3	DNA Repair	162/4222	336/10891	2.17E-04	2.23E-03
SMPD3	CDC42 GTPase cycle	82/4222	155/10891	2.25E-04	2.30E-03
SMPD3	RHOG GTPase cycle	44/4222	74/10891	2.40E-04	2.43E-03

SMPD3	Nuclear Envelope Breakdown	33/4222	52/10891	2.64E-04	2.66E-03
SMPD3	Cilium Assembly	102/4222	201/10891	3.31E-04	3.31E-03
SMPD3	SARS-CoV-2 Infection	140/4222	288/10891	3.64E-04	3.62E-03
SMPD3	DNA Replication	96/4222	188/10891	3.70E-04	3.66E-03
SMPD3	AURKA Activation by TPX2	43/4222	73/10891	3.75E-04	3.68E-03
SMPD3	Signaling by TGFB family members	66/4222	122/10891	3.96E-04	3.87E-03
SMPD3	Golgi-to-ER retrograde transport	71/4222	133/10891	4.09E-04	3.97E-03
SMPD3	Intra-Golgi and retrograde Golgi-to-ER traffic	102/4222	202/10891	4.17E-04	4.02E-03
SMPD3	Signaling by ALK in cancer	34/4222	55/10891	4.40E-04	4.13E-03
SMPD3	Signaling by ALK fusions and activated point mutants	34/4222	55/10891	4.40E-04	4.13E-03
SMPD3	Transcriptional activity of SMAD2/SMAD3:SMAD4 heterotrimer	32/4222	51/10891	4.40E-04	4.13E-03
SMPD3	SUMO E3 ligases SUMOylate target proteins	93/4222	182/10891	4.40E-04	4.13E-03
SMPD3	Global Genome Nucleotide Excision Repair (GG-NER)	48/4222	84/10891	4.69E-04	4.39E-03
SMPD3	Regulation of TP53 Activity	83/4222	160/10891	4.75E-04	4.41E-03
SMPD3	G2/M Checkpoints	86/4222	167/10891	5.15E-04	4.70E-03
SMPD3	Downregulation of SMAD2/3:SMAD4 transcriptional activity	21/4222	30/10891	5.15E-04	4.70E-03
SMPD3	Signaling by CSF3 (G-CSF)	21/4222	30/10891	5.15E-04	4.70E-03
SMPD3	Interleukin-6 signaling	10/4222	11/10891	5.43E-04	4.92E-03
SMPD3	MAPK family signaling cascades	155/4222	325/10891	5.49E-04	4.95E-03
SMPD3	Cooperation of Prefoldin and TriC/CCT in actin and tubulin folding	22/4222	32/10891	5.63E-04	5.05E-03
SMPD3	Glucose metabolism	51/4222	91/10891	5.94E-04	5.29E-03
SMPD3	SUMOylation of SUMOylation proteins	23/4222	34/10891	6.04E-04	5.35E-03
SMPD3	DNA Damage Recognition in GG-NER	25/4222	38/10891	6.66E-04	5.84E-03
SMPD3	Early SARS-CoV-2 Infection Events	25/4222	38/10891	6.66E-04	5.84E-03
SMPD3	NS1 Mediated Effects on Host Pathways	26/4222	40/10891	6.88E-04	6.00E-03
SMPD3	Transport of Mature mRNAs Derived from Intronless Transcripts	27/4222	42/10891	7.05E-04	6.11E-03
SMPD3	Nucleotide Excision Repair	60/4222	111/10891	7.33E-04	6.32E-03
SMPD3	Protein ubiquitination	45/4222	79/10891	7.66E-04	6.56E-03
SMPD3	HIV Life Cycle	77/4222	149/10891	8.63E-04	7.33E-03
SMPD3	Syndecan interactions	19/4222	27/10891	8.64E-04	7.33E-03
SMPD3	Transport to the Golgi and subsequent modification	93/4222	185/10891	8.83E-04	7.44E-03
SMPD3	MAPK1/MAPK3 signaling	137/4222	286/10891	8.99E-04	7.53E-03
SMPD3	RAB geranylgeranylation	38/4222	65/10891	9.86E-04	8.19E-03
SMPD3	ER to Golgi Anterograde Transport	79/4222	154/10891	9.87E-04	8.19E-03
SMPD3	MHC class II antigen presentation	65/4222	123/10891	1.00E-03	8.25E-03
SMPD3	PI Metabolism	47/4222	84/10891	1.01E-03	8.25E-03

SMPD3	Transport of Ribonucleoproteins into the Host Nucleus	21/4222	31/10891	1.02E-03	8.25E-03
SMPD3	NEP/NS2 Interacts with the Cellular Export Machinery	21/4222	31/10891	1.02E-03	8.25E-03
SMPD3	Nuclear import of Rev protein	22/4222	33/10891	1.07E-03	8.64E-03
SMPD3	RHOQ GTPase cycle	35/4222	59/10891	1.07E-03	8.64E-03
SMPD3	Nuclear Pore Complex (NPC) Disassembly	23/4222	35/10891	1.12E-03	8.93E-03
SMPD3	Transport of Mature mRNA Derived from an Intronless Transcript	26/4222	41/10891	1.19E-03	9.47E-03
SMPD3	MAPK3 (ERK1) activation	9/4222	10/10891	1.28E-03	9.99E-03
SMPD3	Cohesin Loading onto Chromatin	9/4222	10/10891	1.28E-03	9.99E-03
SMPD3	Processing and activation of SUMO	9/4222	10/10891	1.28E-03	9.99E-03
SMPD3	STAT3 nuclear events downstream of ALK signaling	9/4222	10/10891	1.28E-03	9.99E-03
SMPD3	Translation of Replicase and Assembly of the Replication Transcription Complex	12/4222	15/10891	1.39E-03	1.08E-02
SMPD3	DNA Replication Pre-Initiation	81/4222	160/10891	1.42E-03	1.09E-02
SMPD3	Cytosolic sensors of pathogen-associated DNA	37/4222	64/10891	1.52E-03	1.17E-02
SMPD3	Signaling by MET	44/4222	79/10891	1.63E-03	1.24E-02
SMPD3	Toll Like Receptor 3 (TLR3) Cascade	56/4222	105/10891	1.64E-03	1.24E-02
SMPD3	Late Phase of HIV Life Cycle	70/4222	136/10891	1.66E-03	1.24E-02
SMPD3	Signaling by NOTCH1 PEST Domain Mutants in Cancer	34/4222	58/10891	1.68E-03	1.24E-02
SMPD3	Signaling by NOTCH1 in Cancer	34/4222	58/10891	1.68E-03	1.24E-02
SMPD3	Constitutive Signaling by NOTCH1 PEST Domain Mutants	34/4222	58/10891	1.68E-03	1.24E-02
SMPD3	Signaling by NOTCH1 HD+PEST Domain Mutants in Cancer	34/4222	58/10891	1.68E-03	1.24E-02
SMPD3	Constitutive Signaling by NOTCH1 HD+PEST Domain Mutants	34/4222	58/10891	1.68E-03	1.24E-02
SMPD3	Synthesis of PC	19/4222	28/10891	1.71E-03	1.25E-02
SMPD3	Prefoldin mediated transfer of substrate to CCT/TriC	19/4222	28/10891	1.71E-03	1.25E-02
SMPD3	Golgi Associated Vesicle Biogenesis	33/4222	56/10891	1.73E-03	1.26E-02
SMPD3	Signaling by ROBO receptors	106/4222	218/10891	1.76E-03	1.28E-02
SMPD3	RHOD GTPase cycle	32/4222	54/10891	1.78E-03	1.28E-02
SMPD3	Assembly of the pre-replicative complex	73/4222	143/10891	1.78E-03	1.28E-02
SMPD3	Nuclear Envelope (NE) Reassembly	42/4222	75/10891	1.79E-03	1.28E-02
SMPD3	Transcriptional regulation by RUNX1	115/4222	239/10891	1.84E-03	1.31E-02
SMPD3	RAC2 GTPase cycle	48/4222	88/10891	1.86E-03	1.32E-02
SMPD3	Metabolism of carbohydrates	139/4222	295/10891	1.87E-03	1.32E-02
SMPD3	Glycolysis	40/4222	71/10891	1.95E-03	1.36E-02
SMPD3	MyD88-independent TLR4 cascade	58/4222	110/10891	1.95E-03	1.36E-02

SMPD3	TRIF(TICAM1)-mediated TLR4 signaling	58/4222	110/10891	1.95E-03	1.36E-02
SMPD3	Interactions of Rev with host cellular proteins	23/4222	36/10891	1.97E-03	1.36E-02
SMPD3	RHOH GTPase cycle	24/4222	38/10891	1.98E-03	1.37E-02
SMPD3	Regulation of HSF1-mediated heat shock response	39/4222	69/10891	2.04E-03	1.40E-02
SMPD3	Clathrin-mediated endocytosis	74/4222	146/10891	2.14E-03	1.46E-02
SMPD3	Nuclear events stimulated by ALK signaling in cancer	14/4222	19/10891	2.15E-03	1.46E-02
SMPD3	RAF/MAP kinase cascade	132/4222	280/10891	2.35E-03	1.59E-02
SMPD3	Signaling by NOTCH1	41/4222	74/10891	2.64E-03	1.77E-02
SMPD3	RHOC GTPase cycle	41/4222	74/10891	2.64E-03	1.77E-02
SMPD3	HSP90 chaperone cycle for steroid hormone receptors (SHR) in the presence of ligand	32/4222	55/10891	2.69E-03	1.79E-02
SMPD3	RHOJ GTPase cycle	32/4222	55/10891	2.69E-03	1.79E-02
SMPD3	Translation of Replicase and Assembly of the Replication Transcription Complex	11/4222	14/10891	2.90E-03	1.90E-02
SMPD3	Role of phospholipids in phagocytosis	17/4222	25/10891	2.91E-03	1.90E-02
SMPD3	Formation of tubulin folding intermediates by CCT/TriC	17/4222	25/10891	2.91E-03	1.90E-02
SMPD3	Inactivation of CSF3 (G-CSF) signaling	17/4222	25/10891	2.91E-03	1.90E-02
SMPD3	Interferon Signaling	97/4222	200/10891	2.97E-03	1.93E-02
SMPD3	SUMOylation of RNA binding proteins	28/4222	47/10891	3.04E-03	1.97E-02
SMPD3	Signaling by ALK	18/4222	27/10891	3.06E-03	1.98E-02
SMPD3	RIPK1-mediated regulated necrosis	19/4222	29/10891	3.17E-03	2.01E-02
SMPD3	Regulation of necroptotic cell death	19/4222	29/10891	3.17E-03	2.01E-02
SMPD3	Viral Messenger RNA Synthesis	26/4222	43/10891	3.18E-03	2.01E-02
SMPD3	Formation of Incision Complex in GG-NER	26/4222	43/10891	3.18E-03	2.01E-02
SMPD3	Aggrephagy	26/4222	43/10891	3.18E-03	2.01E-02
SMPD3	Opioid Signalling	49/4222	92/10891	3.23E-03	2.02E-02
SMPD3	RNA Polymerase III Transcription	25/4222	41/10891	3.23E-03	2.02E-02
SMPD3	RNA Polymerase III Abortive And Retractive Initiation	25/4222	41/10891	3.23E-03	2.02E-02
SMPD3	Regulation of Glucokinase by Glucokinase Regulatory Protein	20/4222	31/10891	3.25E-03	2.02E-02
SMPD3	Defective TPR may confer susceptibility towards thyroid papillary carcinoma (TPC)	20/4222	31/10891	3.25E-03	2.02E-02
SMPD3	Export of Viral Ribonucleoproteins from Nucleus	21/4222	33/10891	3.29E-03	2.02E-02
SMPD3	Vpr-mediated nuclear import of PICs	21/4222	33/10891	3.29E-03	2.02E-02
SMPD3	Plasma lipoprotein clearance	23/4222	37/10891	3.30E-03	2.02E-02
SMPD3	Transport of the SLBP Dependant Mature mRNA	22/4222	35/10891	3.31E-03	2.02E-02
SMPD3	Resolution of Sister Chromatid Cohesion	64/4222	126/10891	3.85E-03	2.33E-02
SMPD3	SARS-CoV-2 activates/modulates innate and adaptive immune responses	64/4222	126/10891	3.85E-03	2.33E-02

SMPD3	G-protein mediated events	32/4222	56/10891	3.97E-03	2.39E-02
SMPD3	Transcriptional activation of mitochondrial biogenesis	32/4222	56/10891	3.97E-03	2.39E-02
SMPD3	Transport of Mature Transcript to Cytoplasm	45/4222	84/10891	4.03E-03	2.41E-02
SMPD3	Synthesis of PIPs at the Golgi membrane	13/4222	18/10891	4.15E-03	2.48E-02
SMPD3	Synthesis of PIPs at the late endosome membrane	9/4222	11/10891	4.61E-03	2.72E-02
SMPD3	Establishment of Sister Chromatid Cohesion	9/4222	11/10891	4.61E-03	2.72E-02
SMPD3	Signaling by Leptin	9/4222	11/10891	4.61E-03	2.72E-02
SMPD3	NOTCH1 Intracellular Domain Regulates Transcription	28/4222	48/10891	4.62E-03	2.72E-02
SMPD3	PINK1-PRKN Mediated Mitophagy	15/4222	22/10891	4.95E-03	2.90E-02
SMPD3	RND3 GTPase cycle	25/4222	42/10891	5.08E-03	2.96E-02
SMPD3	SUMOylation of ubiquitinylation proteins	23/4222	38/10891	5.33E-03	3.08E-02
SMPD3	RHOV GTPase cycle	23/4222	38/10891	5.33E-03	3.08E-02
SMPD3	Interactions of Vpr with host cellular proteins	22/4222	36/10891	5.42E-03	3.12E-02
SMPD3	E3 ubiquitin ligases ubiquitinate target proteins	33/4222	59/10891	5.46E-03	3.12E-02
SMPD3	Transport of the SLBP independent Mature mRNA	21/4222	34/10891	5.49E-03	3.12E-02
SMPD3	Rev-mediated nuclear export of HIV RNA	21/4222	34/10891	5.49E-03	3.12E-02
SMPD3	RHO GTPases activate IQGAPs	20/4222	32/10891	5.53E-03	3.12E-02
SMPD3	Laminin interactions	19/4222	30/10891	5.53E-03	3.12E-02
SMPD3	Synthesis of active ubiquitin: roles of E1 and E2 enzymes	19/4222	30/10891	5.53E-03	3.12E-02
SMPD3	Translocation of SLC2A4 (GLUT4) to the plasma membrane	39/4222	72/10891	5.59E-03	3.14E-02
SMPD3	tRNA processing in the nucleus	32/4222	57/10891	5.72E-03	3.20E-02
SMPD3	RUNX1 regulates transcription of genes involved in differentiation of HSCs	65/4222	130/10891	5.75E-03	3.21E-02
SMPD3	Mitotic Telophase/Cytokinesis	10/4222	13/10891	5.97E-03	3.32E-02
SMPD3	COPI-mediated anterograde transport	52/4222	101/10891	6.13E-03	3.40E-02
SMPD3	COPII-mediated vesicle transport	37/4222	68/10891	6.22E-03	3.42E-02
SMPD3	Synthesis of PIPs at the plasma membrane	30/4222	53/10891	6.27E-03	3.42E-02
SMPD3	snRNP Assembly	30/4222	53/10891	6.27E-03	3.42E-02
SMPD3	Metabolism of non-coding RNA	30/4222	53/10891	6.27E-03	3.42E-02
SMPD3	Signaling by NOTCH1 HD Domain Mutants in Cancer	11/4222	15/10891	7.06E-03	3.83E-02
SMPD3	Constitutive Signaling by NOTCH1 HD Domain Mutants	11/4222	15/10891	7.06E-03	3.83E-02
SMPD3	TCF dependent signaling in response to WNT	109/4222	233/10891	7.13E-03	3.86E-02
SMPD3	Transcriptional Regulation by TP53	163/4222	362/10891	7.84E-03	4.22E-02
SMPD3	MTOR signalling	24/4222	41/10891	8.00E-03	4.29E-02
SMPD3	Costimulation by the CD28 family	37/4222	69/10891	8.47E-03	4.53E-02

SMPD3	RHOBTB GTPase Cycle	21/4222	35/10891	8.77E-03	4.67E-02
SMPD3	ZBP1(DAI) mediated induction of type I IFNs	14/4222	21/10891	8.91E-03	4.74E-02
SMPD3	Signaling by NOTCH2	20/4222	33/10891	8.98E-03	4.75E-02
SMPD3	Mitophagy	18/4222	29/10891	9.27E-03	4.82E-02
SMPD3	Processing of Capped Intronless Pre-mRNA	18/4222	29/10891	9.27E-03	4.82E-02
SMPD3	Downregulation of ERBB2 signaling	18/4222	29/10891	9.27E-03	4.82E-02
SMPD3	Translation of Structural Proteins	18/4222	29/10891	9.27E-03	4.82E-02
SMPD3	Antigen Presentation: Folding, assembly and peptide loading of class I MHC	18/4222	29/10891	9.27E-03	4.82E-02
SMPD3	Postmitotic nuclear pore complex (NPC) reformation	17/4222	27/10891	9.32E-03	4.84E-02
SOX4	RHO GTPase cycle	149/2453	449/10891	8.05E-08	1.23E-04
SOX4	Phosphorylation of CD3 and TCR zeta chains	16/2453	22/10891	7.63E-07	5.81E-04
SOX4	Translocation of ZAP-70 to Immunological synapse	14/2453	19/10891	3.03E-06	1.54E-03
SOX4	Antigen Presentation: Folding, assembly and peptide loading of class I MHC	18/2453	29/10891	5.38E-06	2.05E-03
SOX4	Signaling by the B Cell Receptor (BCR)	46/2453	112/10891	7.89E-06	2.40E-03
SOX4	PD-1 signaling	15/2453	23/10891	1.41E-05	2.79E-03
SOX4	Chromatin modifying enzymes	92/2453	274/10891	1.47E-05	2.79E-03
SOX4	Chromatin organization	92/2453	274/10891	1.47E-05	2.79E-03
SOX4	RAC1 GTPase cycle	66/2453	185/10891	2.84E-05	4.50E-03
SOX4	Costimulation by the CD28 family	31/2453	69/10891	2.96E-05	4.50E-03
SOX4	Antigen activates B Cell Receptor (BCR) leading to generation of second messengers	18/2453	32/10891	3.61E-05	4.65E-03
SOX4	CDC42 GTPase cycle	57/2453	155/10891	3.66E-05	4.65E-03
SOX4	SUMOylation	66/2453	188/10891	5.05E-05	5.13E-03
SOX4	Amplification of signal from the kinetochores	39/2453	96/10891	5.06E-05	5.13E-03
SOX4	Amplification of signal from unattached kinetochores via a MAD2 inhibitory signal	39/2453	96/10891	5.06E-05	5.13E-03
SOX4	SUMO E3 ligases SUMOylate target proteins	64/2453	182/10891	6.16E-05	5.65E-03
SOX4	TCR signaling	46/2453	120/10891	6.31E-05	5.65E-03
SOX4	RAC3 GTPase cycle	38/2453	94/10891	7.14E-05	6.03E-03
SOX4	Cell Cycle Checkpoints	94/2453	293/10891	8.63E-05	6.92E-03
SOX4	Mitotic Anaphase	78/2453	236/10891	1.16E-04	8.80E-03
SOX4	Mitotic Spindle Checkpoint	43/2453	113/10891	1.31E-04	9.34E-03
SOX4	Mitotic Metaphase and Anaphase	78/2453	237/10891	1.35E-04	9.34E-03
SOX4	Mitotic Prometaphase	68/2453	204/10891	2.34E-04	1.55E-02
SOX4	Potential therapeutics for SARS	38/2453	99/10891	2.57E-04	1.63E-02
SOX4	Separation of Sister Chromatids	64/2453	191/10891	2.99E-04	1.82E-02
SOX4	M Phase	124/2453	418/10891	3.29E-04	1.92E-02
SOX4	RHO GTPase Effectors	100/2453	327/10891	3.81E-04	2.15E-02
SOX4	Generation of second messenger molecules	17/2453	34/10891	4.01E-04	2.18E-02

SOX4	Downstream TCR signaling	37/2453	98/10891	4.49E-04	2.33E-02
SOX4	Cilium Assembly	66/2453	201/10891	4.59E-04	2.33E-02
SOX4	Resolution of Sister Chromatid Cohesion	45/2453	126/10891	4.91E-04	2.41E-02
SOX4	Endosomal/Vacuolar pathway	8/2453	11/10891	5.56E-04	2.65E-02
SOX4	EML4 and NUDC in mitotic spindle formation	42/2453	117/10891	6.60E-04	3.05E-02
SOX4	RHOD GTPase cycle	23/2453	54/10891	7.81E-04	3.49E-02
SOX4	Other semaphorin interactions	11/2453	19/10891	9.02E-04	3.92E-02
SOX4	Intracellular signaling by second messengers	93/2453	309/10891	1.06E-03	4.28E-02
SOX4	MHC class II antigen presentation	43/2453	123/10891	1.07E-03	4.28E-02
SOX4	PIP3 activates AKT signaling	82/2453	267/10891	1.07E-03	4.28E-02
SOX4	Immunoregulatory interactions between a Lymphoid and a non-Lymphoid cell	46/2453	134/10891	1.13E-03	4.42E-02
SOX4	Interferon gamma signaling	34/2453	92/10891	1.16E-03	4.43E-02
SOX4	Mitotic G1 phase and G1/S transition	50/2453	149/10891	1.25E-03	4.66E-02
SOX4	HATs acetylate histones	48/2453	142/10891	1.30E-03	4.72E-02
SOX4	Class I MHC mediated antigen processing & presentation	111/2453	381/10891	1.33E-03	4.72E-02
TAC3	KEAP1-NFE2L2 pathway	46/2105	102/10891	2.44E-09	3.70E-06
TAC3	Nuclear events mediated by NFE2L2	37/2105	79/10891	2.56E-08	1.80E-05
TAC3	Transcriptional regulation by RUNX3	42/2105	96/10891	3.55E-08	1.80E-05
TAC3	SUMOylation	66/2105	188/10891	2.18E-07	8.25E-05
TAC3	SUMO E3 ligases SUMOylate target proteins	62/2105	182/10891	1.63E-06	4.94E-04
TAC3	Diseases of signal transduction by growth factor receptors and second messengers	123/2105	433/10891	2.04E-06	5.15E-04
TAC3	Cohesin Loading onto Chromatin	9/2105	10/10891	3.07E-06	6.65E-04
TAC3	CLEC7A (Dectin-1) signaling	39/2105	101/10891	4.93E-06	9.34E-04
TAC3	Downstream TCR signaling	38/2105	98/10891	5.78E-06	9.74E-04
TAC3	Cellular response to chemical stress	63/2105	194/10891	8.09E-06	1.23E-03
TAC3	Cross-presentation of soluble exogenous antigens (endosomes)	23/2105	49/10891	1.06E-05	1.24E-03
TAC3	Fc epsilon receptor (FCERI) signaling	47/2105	134/10891	1.21E-05	1.24E-03
TAC3	Downstream signaling events of B Cell Receptor (BCR)	33/2105	83/10891	1.26E-05	1.24E-03
TAC3	Signaling by the B Cell Receptor (BCR)	41/2105	112/10891	1.33E-05	1.24E-03
TAC3	Chromatin modifying enzymes	82/2105	274/10891	1.33E-05	1.24E-03
TAC3	Chromatin organization	82/2105	274/10891	1.33E-05	1.24E-03
TAC3	Establishment of Sister Chromatid Cohesion	9/2105	11/10891	1.40E-05	1.24E-03
TAC3	TCR signaling	43/2105	120/10891	1.53E-05	1.29E-03
TAC3	SUMOylation of DNA damage response and repair proteins	31/2105	77/10891	1.71E-05	1.36E-03
TAC3	HIV Infection	71/2105	231/10891	1.88E-05	1.42E-03
TAC3	Neddylation	73/2105	240/10891	2.14E-05	1.54E-03
TAC3	Processing of Capped Intron-Containing	74/2105	246/10891	2.86E-05	1.97E-03

	Pre-mRNA				
TAC3	Ubiquitin-dependent degradation of Cyclin D	23/2105	52/10891	3.53E-05	2.33E-03
TAC3	Metabolism of polyamines	25/2105	59/10891	3.97E-05	2.41E-03
TAC3	NIK-->noncanonical NF-kB signaling	25/2105	59/10891	3.97E-05	2.41E-03
TAC3	AUF1 (hnRNP D0) binds and destabilizes mRNA	24/2105	56/10891	4.52E-05	2.57E-03
TAC3	Regulation of Apoptosis	23/2105	53/10891	5.10E-05	2.57E-03
TAC3	Regulation of mRNA stability by proteins that bind AU-rich elements	33/2105	88/10891	5.17E-05	2.57E-03
TAC3	Antigen processing: Ubiquitination & Proteasome degradation	88/2105	309/10891	5.19E-05	2.57E-03
TAC3	ABC-family proteins mediated transport	37/2105	103/10891	5.52E-05	2.57E-03
TAC3	Signaling by Hedgehog	49/2105	149/10891	5.53E-05	2.57E-03
TAC3	Dectin-1 mediated noncanonical NF-kB signaling	25/2105	60/10891	5.57E-05	2.57E-03
TAC3	Regulation of activated PAK-2p34 by proteasome mediated degradation	22/2105	50/10891	5.73E-05	2.57E-03
TAC3	Activation of NF-kappaB in B cells	27/2105	67/10891	5.76E-05	2.57E-03
TAC3	Host Interactions of HIV factors	44/2105	130/10891	6.07E-05	2.63E-03
TAC3	Degradation of DVL	24/2105	57/10891	6.38E-05	2.69E-03
TAC3	FBXL7 down-regulates AURKA during mitotic entry and in early mitosis	23/2105	54/10891	7.28E-05	2.98E-03
TAC3	Cellular response to hypoxia	29/2105	75/10891	7.61E-05	3.04E-03
TAC3	Regulation of ornithine decarboxylase (ODC)	22/2105	51/10891	8.26E-05	3.21E-03
TAC3	Hedgehog ligand biogenesis	26/2105	65/10891	9.11E-05	3.45E-03
TAC3	Class I MHC mediated antigen processing & presentation	103/2105	381/10891	1.22E-04	4.28E-03
TAC3	Hh mutants abrogate ligand secretion	24/2105	59/10891	1.23E-04	4.28E-03
TAC3	CLEC7A (Dectin-1) induces NFAT activation	9/2105	13/10891	1.24E-04	4.28E-03
TAC3	Mitotic Telophase/Cytokinesis	9/2105	13/10891	1.24E-04	4.28E-03
TAC3	C-type lectin receptors (CLRs)	46/2105	142/10891	1.38E-04	4.65E-03
TAC3	Hh mutants are degraded by ERAD	23/2105	56/10891	1.42E-04	4.69E-03
TAC3	Intracellular signaling by second messengers	86/2105	309/10891	1.51E-04	4.87E-03
TAC3	Hedgehog 'on' state	31/2105	85/10891	1.57E-04	4.96E-03
TAC3	GLI3 is processed to GLI3R by the proteasome	24/2105	60/10891	1.68E-04	5.19E-03
TAC3	FCERI mediated NF-kB activation	30/2105	82/10891	1.87E-04	5.55E-03
TAC3	NS1 Mediated Effects on Host Pathways	18/2105	40/10891	1.89E-04	5.55E-03
TAC3	Deubiquitination	83/2105	298/10891	1.90E-04	5.55E-03
TAC3	Stabilization of p53	23/2105	57/10891	1.96E-04	5.59E-03
TAC3	Regulation of RAS by GAPs	26/2105	68/10891	2.18E-04	6.12E-03
TAC3	Negative regulation of NOTCH4 signaling	22/2105	54/10891	2.27E-04	6.26E-03
TAC3	Transport of Ribonucleoproteins into the Host Nucleus	15/2105	31/10891	2.41E-04	6.54E-03
TAC3	Ub-specific processing proteases	64/2105	220/10891	2.77E-04	7.36E-03
TAC3	Beta-catenin independent WNT signaling	46/2105	146/10891	2.83E-04	7.41E-03

TAC3	SCF-beta-TrCP mediated degradation of Emi1	22/2105	55/10891	3.10E-04	7.84E-03
TAC3	Regulation of RUNX3 expression and activity	22/2105	55/10891	3.10E-04	7.84E-03
TAC3	Oxygen-dependent proline hydroxylation of Hypoxia-inducible Factor Alpha	25/2105	66/10891	3.39E-04	8.18E-03
TAC3	Vpu mediated degradation of CD4	21/2105	52/10891	3.62E-04	8.18E-03
TAC3	Autodegradation of the E3 ubiquitin ligase COP1	21/2105	52/10891	3.62E-04	8.18E-03
TAC3	Ubiquitin Mediated Degradation of Phosphorylated Cdc25A	21/2105	52/10891	3.62E-04	8.18E-03
TAC3	p53-Independent DNA Damage Response	21/2105	52/10891	3.62E-04	8.18E-03
TAC3	p53-Independent G1/S DNA damage checkpoint	21/2105	52/10891	3.62E-04	8.18E-03
TAC3	GSK3B and BTRC:CUL1-mediated-degradation of NFE2L2	21/2105	52/10891	3.62E-04	8.18E-03
TAC3	ABC transporter disorders	28/2105	78/10891	4.33E-04	9.66E-03
TAC3	Signaling by NOTCH4	29/2105	82/10891	4.59E-04	9.97E-03
TAC3	SCF(Skp2)-mediated degradation of p27/p21	23/2105	60/10891	4.74E-04	9.97E-03
TAC3	Degradation of GLI1 by the proteasome	23/2105	60/10891	4.74E-04	9.97E-03
TAC3	Degradation of GLI2 by the proteasome	23/2105	60/10891	4.74E-04	9.97E-03
TAC3	Nuclear import of Rev protein	15/2105	33/10891	5.64E-04	1.16E-02
TAC3	Vpr-mediated nuclear import of PICs	15/2105	33/10891	5.64E-04	1.16E-02
TAC3	S Phase	49/2105	163/10891	6.16E-04	1.24E-02
TAC3	Defective CFTR causes cystic fibrosis	23/2105	61/10891	6.23E-04	1.24E-02
TAC3	Vif-mediated degradation of APOBEC3G	21/2105	54/10891	6.59E-04	1.30E-02
TAC3	Regulation of FOXO transcriptional activity by acetylation	7/2105	10/10891	6.90E-04	1.34E-02
TAC3	mRNA Splicing - Major Pathway	54/2105	185/10891	7.34E-04	1.40E-02
TAC3	Regulation of PTEN stability and activity	25/2105	69/10891	7.36E-04	1.40E-02
TAC3	Degradation of AXIN	21/2105	55/10891	8.75E-04	1.60E-02
TAC3	p53-Dependent G1 DNA Damage Response	24/2105	66/10891	8.79E-04	1.60E-02
TAC3	p53-Dependent G1/S DNA damage checkpoint	24/2105	66/10891	8.79E-04	1.60E-02
TAC3	Cellular response to heat stress	30/2105	89/10891	9.23E-04	1.65E-02
TAC3	MAPK6/MAPK4 signaling	30/2105	89/10891	9.23E-04	1.65E-02
TAC3	Separation of Sister Chromatids	55/2105	191/10891	9.38E-04	1.65E-02
TAC3	Hedgehog 'off' state	36/2105	113/10891	9.91E-04	1.73E-02
TAC3	Role of phospholipids in phagocytosis	12/2105	25/10891	1.11E-03	1.91E-02
TAC3	RHO GTPase cycle	113/2105	449/10891	1.14E-03	1.93E-02
TAC3	G-protein mediated events	21/2105	56/10891	1.15E-03	1.93E-02
TAC3	Transport of the SLBP Dependant Mature mRNA	15/2105	35/10891	1.20E-03	1.97E-02
TAC3	Nuclear Pore Complex (NPC) Disassembly	15/2105	35/10891	1.20E-03	1.97E-02
TAC3	mRNA Splicing	55/2105	193/10891	1.22E-03	1.99E-02
TAC3	Mitotic Anaphase	65/2105	236/10891	1.24E-03	1.99E-02
TAC3	Degradation of beta-catenin by the destruction complex	28/2105	83/10891	1.33E-03	2.10E-02
TAC3	Autodegradation of Cdh1 by Cdh1:APC/C	23/2105	64/10891	1.34E-03	2.10E-02

TAC3	Asymmetric localization of PCP proteins	23/2105	64/10891	1.34E-03	2.10E-02
TAC3	Mitotic Metaphase and Anaphase	65/2105	237/10891	1.39E-03	2.11E-02
TAC3	Interleukin-1 signaling	36/2105	115/10891	1.41E-03	2.11E-02
TAC3	tRNA processing	34/2105	107/10891	1.42E-03	2.11E-02
TAC3	APC/C:Cdc20 mediated degradation of Securin	24/2105	68/10891	1.42E-03	2.11E-02
TAC3	G1/S DNA Damage Checkpoints	24/2105	68/10891	1.42E-03	2.11E-02
TAC3	ISG15 antiviral mechanism	25/2105	72/10891	1.49E-03	2.19E-02
TAC3	PTEN Regulation	42/2105	140/10891	1.52E-03	2.22E-02
TAC3	Opioid Signalling	30/2105	92/10891	1.68E-03	2.33E-02
TAC3	DNA Repair	87/2105	336/10891	1.69E-03	2.33E-02
TAC3	Interactions of Vpr with host cellular proteins	15/2105	36/10891	1.69E-03	2.33E-02
TAC3	Interactions of Rev with host cellular proteins	15/2105	36/10891	1.69E-03	2.33E-02
TAC3	DAG and IP3 signaling	17/2105	43/10891	1.70E-03	2.33E-02
TAC3	Viral Messenger RNA Synthesis	17/2105	43/10891	1.70E-03	2.33E-02
TAC3	FOXO-mediated transcription	23/2105	65/10891	1.70E-03	2.33E-02
TAC3	Signaling by WNT	86/2105	332/10891	1.77E-03	2.39E-02
TAC3	Regulation of RUNX2 expression and activity	25/2105	73/10891	1.86E-03	2.49E-02
TAC3	Translation of Replicase and Assembly of the Replication Transcription Complex	8/2105	14/10891	1.89E-03	2.51E-02
TAC3	Transcriptional regulation by RUNX2	37/2105	121/10891	1.95E-03	2.56E-02
TAC3	Cilium Assembly	56/2105	201/10891	1.96E-03	2.56E-02
TAC3	RAF/MAP kinase cascade	74/2105	280/10891	2.03E-03	2.64E-02
TAC3	Autophagy	44/2105	151/10891	2.25E-03	2.88E-02
TAC3	RAC1 GTPase cycle	52/2105	185/10891	2.27E-03	2.88E-02
TAC3	Sphingolipid de novo biosynthesis	17/2105	44/10891	2.28E-03	2.88E-02
TAC3	Regulation of cholesterol biosynthesis by SREBP (SREBF)	20/2105	55/10891	2.30E-03	2.88E-02
TAC3	Regulation of TP53 Activity	46/2105	160/10891	2.43E-03	3.02E-02
TAC3	Signaling by ALK	12/2105	27/10891	2.53E-03	3.12E-02
TAC3	M Phase	104/2105	418/10891	2.61E-03	3.19E-02
TAC3	Transport of the SLBP independent Mature mRNA	14/2105	34/10891	2.73E-03	3.27E-02
TAC3	SUMOylation of SUMOylation proteins	14/2105	34/10891	2.73E-03	3.27E-02
TAC3	Nuclear Envelope Breakdown	19/2105	52/10891	2.75E-03	3.27E-02
TAC3	Orc1 removal from chromatin	24/2105	71/10891	2.76E-03	3.27E-02
TAC3	Nucleotide Excision Repair	34/2105	111/10891	2.81E-03	3.31E-02
TAC3	Macroautophagy	40/2105	136/10891	2.89E-03	3.36E-02
TAC3	Cyclin E associated events during G1/S transition	27/2105	83/10891	2.91E-03	3.36E-02
TAC3	HIV Life Cycle	43/2105	149/10891	3.06E-03	3.50E-02
TAC3	NEP/NS2 Interacts with the Cellular Export Machinery	13/2105	31/10891	3.17E-03	3.50E-02
TAC3	Regulation of Glucokinase by Glucokinase Regulatory Protein	13/2105	31/10891	3.17E-03	3.50E-02

TAC3	Defective TPR may confer susceptibility towards thyroid papillary carcinoma (TPC)	13/2105	31/10891	3.17E-03	3.50E-02
TAC3	Pyruvate metabolism	13/2105	31/10891	3.17E-03	3.50E-02
TAC3	SUMOylation of ubiquitinylation proteins	15/2105	38/10891	3.19E-03	3.50E-02
TAC3	DNA Damage Recognition in GG-NER	15/2105	38/10891	3.19E-03	3.50E-02
TAC3	MAPK family signaling cascades	83/2105	325/10891	3.21E-03	3.50E-02
TAC3	Translation of Replicase and Assembly of the Replication Transcription Complex	8/2105	15/10891	3.37E-03	3.65E-02
TAC3	PCP/CE pathway	29/2105	92/10891	3.50E-03	3.73E-02
TAC3	snRNP Assembly	19/2105	53/10891	3.52E-03	3.73E-02
TAC3	Metabolism of non-coding RNA	19/2105	53/10891	3.52E-03	3.73E-02
TAC3	Transport of Mature mRNAs Derived from Intronless Transcripts	16/2105	42/10891	3.59E-03	3.78E-02
TAC3	MAPK1/MAPK3 signaling	74/2105	286/10891	3.67E-03	3.83E-02
TAC3	Cdc20:Phospho-APC/C mediated degradation of Cyclin A	24/2105	73/10891	4.15E-03	4.28E-02
TAC3	CDK-mediated phosphorylation and removal of Cdc6	24/2105	73/10891	4.15E-03	4.28E-02
TAC3	Cyclin A:Cdk2-associated events at S phase entry	27/2105	85/10891	4.21E-03	4.32E-02
TAC3	UCH proteinases	31/2105	102/10891	4.79E-03	4.88E-02
TAC3	RAB GEFs exchange GTP for GDP on RABs	28/2105	90/10891	4.99E-03	4.99E-02
TAC3	APC/C:Cdh1 mediated degradation of Cdc20 and other APC/C:Cdh1 targeted proteins in late mitosis/early G1	24/2105	74/10891	5.04E-03	4.99E-02
TAC3	APC:Cdc20 mediated degradation of cell cycle proteins prior to satisfation of the cell cycle checkpoint	24/2105	74/10891	5.04E-03	4.99E-02
TAC3	PIP3 activates AKT signaling	69/2105	267/10891	5.05E-03	4.99E-02
TAC3	SUMOylation of RNA binding proteins	17/2105	47/10891	5.07E-03	4.99E-02
XYLB	Biosynthesis of the N-glycan precursor (dolichol lipid-linked oligosaccharide, LLO) and transfer to a nascent protein	37/2518	78/10891	2.01E-06	1.64E-03
XYLB	Eukaryotic Translation Termination	42/2518	93/10891	2.13E-06	1.64E-03
XYLB	Peptide chain elongation	40/2518	89/10891	4.29E-06	2.20E-03
XYLB	Eukaryotic Translation Elongation	41/2518	93/10891	5.91E-06	2.27E-03
XYLB	Formation of a pool of free 40S subunits	43/2518	101/10891	1.05E-05	2.27E-03
XYLB	Response of EIF2AK4 (GCN2) to amino acid deficiency	43/2518	101/10891	1.05E-05	2.27E-03
XYLB	Nonsense Mediated Decay (NMD) independent of the Exon Junction Complex (EJC)	41/2518	95/10891	1.12E-05	2.27E-03
XYLB	Viral mRNA Translation	39/2518	89/10891	1.18E-05	2.27E-03
XYLB	Selenocysteine synthesis	40/2518	93/10891	1.57E-05	2.51E-03
XYLB	SRP-dependent cotranslational protein targeting	46/2518	112/10891	1.63E-05	2.51E-03

	to membrane				
XYLB	CDC42 GTPase cycle	59/2518	155/10891	1.86E-05	2.61E-03
XYLB	Translation	98/2518	291/10891	2.13E-05	2.73E-03
XYLB	GTP hydrolysis and joining of the 60S ribosomal subunit	45/2518	112/10891	3.85E-05	4.56E-03
XYLB	L13a-mediated translational silencing of Ceruloplasmin expression	44/2518	111/10891	6.84E-05	7.53E-03
XYLB	Diseases associated with N-glycosylation of proteins	13/2518	20/10891	7.64E-05	7.85E-03
XYLB	Eukaryotic Translation Initiation	46/2518	119/10891	9.72E-05	8.81E-03
XYLB	Cap-dependent Translation Initiation	46/2518	119/10891	9.72E-05	8.81E-03
XYLB	Nonsense-Mediated Decay (NMD)	44/2518	115/10891	1.80E-04	1.46E-02
XYLB	Nonsense Mediated Decay (NMD) enhanced by the Exon Junction Complex (EJC)	44/2518	115/10891	1.80E-04	1.46E-02
XYLB	Synthesis of substrates in N-glycan biosythesis	27/2518	63/10891	3.91E-04	3.02E-02
XYLB	RHO GTPase cycle	134/2518	449/10891	4.69E-04	3.44E-02
ZNF473	rRNA processing in the nucleus and cytosol	81/1521	194/10891	9.96E-22	1.43E-18
ZNF473	rRNA processing	83/1521	204/10891	2.32E-21	1.67E-18
ZNF473	Major pathway of rRNA processing in the nucleolus and cytosol	74/1521	184/10891	7.95E-19	3.81E-16
ZNF473	Processing of Capped Intron-Containing Pre-mRNA	80/1521	246/10891	5.01E-14	1.54E-11
ZNF473	mRNA Splicing	68/1521	193/10891	5.38E-14	1.54E-11
ZNF473	mRNA Splicing - Major Pathway	66/1521	185/10891	6.43E-14	1.54E-11
ZNF473	rRNA modification in the nucleus and cytosol	33/1521	61/10891	1.60E-13	3.29E-11
ZNF473	Translation	79/1521	291/10891	1.65E-09	2.95E-07
ZNF473	tRNA processing	37/1521	107/10891	5.46E-08	8.71E-06
ZNF473	Eukaryotic Translation Initiation	39/1521	119/10891	1.25E-07	1.63E-05
ZNF473	Cap-dependent Translation Initiation	39/1521	119/10891	1.25E-07	1.63E-05
ZNF473	RNA polymerase II transcribes snRNA genes	28/1521	74/10891	2.68E-07	3.20E-05
ZNF473	Nonsense-Mediated Decay (NMD)	37/1521	115/10891	4.41E-07	4.53E-05
ZNF473	Nonsense Mediated Decay (NMD) enhanced by the Exon Junction Complex (EJC)	37/1521	115/10891	4.41E-07	4.53E-05
ZNF473	SUMOylation of transcription cofactors	20/1521	45/10891	6.77E-07	6.48E-05
ZNF473	Regulation of expression of SLITs and ROBOs	48/1521	171/10891	9.28E-07	8.33E-05
ZNF473	L13a-mediated translational silencing of Ceruloplasmin expression	35/1521	111/10891	1.53E-06	1.29E-04
ZNF473	GTP hydrolysis and joining of the 60S ribosomal subunit	35/1521	112/10891	1.93E-06	1.54E-04
ZNF473	Late Phase of HIV Life Cycle	40/1521	136/10891	2.07E-06	1.56E-04
ZNF473	HIV Infection	58/1521	231/10891	3.87E-06	2.77E-04
ZNF473	Cytosolic tRNA aminoacylation	13/1521	24/10891	4.06E-06	2.77E-04
ZNF473	KEAP1-NFE2L2 pathway	32/1521	102/10891	4.79E-06	3.07E-04

ZNF473	Folding of actin by CCT/TriC	8/1521	10/10891	4.92E-06	3.07E-04
ZNF473	Influenza Infection	43/1521	156/10891	5.65E-06	3.38E-04
ZNF473	Selenoamino acid metabolism	35/1521	118/10891	7.19E-06	4.10E-04
ZNF473	RNA Polymerase II Pre-transcription Events	27/1521	81/10891	7.42E-06	4.10E-04
ZNF473	Cytosolic sensors of pathogen-associated DNA	23/1521	64/10891	8.51E-06	4.53E-04
ZNF473	RNA Polymerase III Transcription Initiation	16/1521	36/10891	8.89E-06	4.56E-04
ZNF473	HIV Life Cycle	41/1521	149/10891	9.69E-06	4.80E-04
ZNF473	RNA Polymerase III Transcription	17/1521	41/10891	1.45E-05	6.72E-04
ZNF473	RNA Polymerase III Abortive And Retractive Initiation	17/1521	41/10891	1.45E-05	6.72E-04
ZNF473	Signaling by ROBO receptors	53/1521	218/10891	2.64E-05	1.19E-03
ZNF473	Interleukin-1 signaling	33/1521	115/10891	2.77E-05	1.20E-03
ZNF473	Influenza Viral RNA Transcription and Replication	37/1521	135/10891	2.85E-05	1.20E-03
ZNF473	tRNA modification in the nucleus and cytosol	17/1521	43/10891	3.06E-05	1.20E-03
ZNF473	Formation of a pool of free 40S subunits	30/1521	101/10891	3.08E-05	1.20E-03
ZNF473	Response of EIF2AK4 (GCN2) to amino acid deficiency	30/1521	101/10891	3.08E-05	1.20E-03
ZNF473	RNA Polymerase III Transcription Initiation From Type 3 Promoter	13/1521	28/10891	3.53E-05	1.33E-03
ZNF473	Nuclear events mediated by NFE2L2	25/1521	79/10891	4.34E-05	1.60E-03
ZNF473	Activation of NF-kappaB in B cells	22/1521	67/10891	6.60E-05	2.37E-03
ZNF473	Transcription of the HIV genome	22/1521	70/10891	1.38E-04	4.72E-03
ZNF473	Cellular response to heat stress	26/1521	89/10891	1.38E-04	4.72E-03
ZNF473	Nonsense Mediated Decay (NMD) independent of the Exon Junction Complex (EJC)	27/1521	95/10891	1.72E-04	5.75E-03
ZNF473	RNA Polymerase III Transcription Initiation From Type 1 Promoter	12/1521	28/10891	1.83E-04	5.97E-03
ZNF473	Attenuation phase	8/1521	14/10891	1.95E-04	6.21E-03
ZNF473	Nucleotide Excision Repair	30/1521	111/10891	2.07E-04	6.46E-03
ZNF473	Antigen processing: Ubiquitination & Proteasome degradation	66/1521	309/10891	2.13E-04	6.50E-03
ZNF473	Regulation of TP53 Activity	39/1521	160/10891	2.78E-04	8.31E-03
ZNF473	RNA Polymerase III Chain Elongation	9/1521	18/10891	2.91E-04	8.38E-03
ZNF473	Downstream signaling events of B Cell Receptor (BCR)	24/1521	83/10891	2.92E-04	8.38E-03
ZNF473	Eukaryotic Translation Elongation	26/1521	93/10891	3.00E-04	8.46E-03
ZNF473	tRNA Aminoacylation	15/1521	42/10891	3.37E-04	9.29E-03
ZNF473	Dectin-1 mediated noncanonical NF-kB signaling	19/1521	60/10891	3.46E-04	9.38E-03
ZNF473	Transcriptional regulation of white adipocyte differentiation	24/1521	84/10891	3.55E-04	9.40E-03
ZNF473	Peptide chain elongation	25/1521	89/10891	3.60E-04	9.40E-03
ZNF473	HIV Transcription Initiation	16/1521	47/10891	4.03E-04	9.49E-03
ZNF473	RNA Polymerase II HIV Promoter Escape	16/1521	47/10891	4.03E-04	9.49E-03

ZNF473	RNA Polymerase II Promoter Escape	16/1521	47/10891	4.03E-04	9.49E-03
ZNF473	RNA Polymerase II Transcription Pre-Initiation And Promoter Opening	16/1521	47/10891	4.03E-04	9.49E-03
ZNF473	RNA Polymerase II Transcription Initiation	16/1521	47/10891	4.03E-04	9.49E-03
ZNF473	RNA Polymerase II Transcription Initiation And Promoter Clearance	16/1521	47/10891	4.03E-04	9.49E-03
ZNF473	Association of TriC/CCT with target proteins during biosynthesis	14/1521	39/10891	4.97E-04	1.15E-02
ZNF473	DNA Repair	69/1521	336/10891	5.07E-04	1.15E-02
ZNF473	tRNA processing in the nucleus	18/1521	57/10891	5.11E-04	1.15E-02
ZNF473	RHOBTB GTPase Cycle	13/1521	35/10891	5.38E-04	1.17E-02
ZNF473	RNA Polymerase III Transcription Termination	10/1521	23/10891	5.52E-04	1.17E-02
ZNF473	RHOBTB2 GTPase cycle	10/1521	23/10891	5.52E-04	1.17E-02
ZNF473	RHOBTB1 GTPase cycle	10/1521	23/10891	5.52E-04	1.17E-02
ZNF473	RNA Polymerase III Transcription Initiation From Type 2 Promoter	11/1521	27/10891	5.75E-04	1.18E-02
ZNF473	Postmitotic nuclear pore complex (NPC) reformation	11/1521	27/10891	5.75E-04	1.18E-02
ZNF473	snRNP Assembly	17/1521	53/10891	5.92E-04	1.18E-02
ZNF473	Metabolism of non-coding RNA	17/1521	53/10891	5.92E-04	1.18E-02
ZNF473	FCERI mediated NF-kB activation	23/1521	82/10891	6.23E-04	1.19E-02
ZNF473	MAP3K8 (TPL2)-dependent MAPK1/3 activation	8/1521	16/10891	6.43E-04	1.19E-02
ZNF473	Formation of RNA Pol II elongation complex	18/1521	58/10891	6.45E-04	1.19E-02
ZNF473	Translation initiation complex formation	18/1521	58/10891	6.45E-04	1.19E-02
ZNF473	Ribosomal scanning and start codon recognition	18/1521	58/10891	6.45E-04	1.19E-02
ZNF473	RNA Polymerase II Transcription Elongation	18/1521	58/10891	6.45E-04	1.19E-02
ZNF473	RNA Polymerase II Transcription Termination	20/1521	68/10891	7.16E-04	1.30E-02
ZNF473	Selenocysteine synthesis	25/1521	93/10891	7.38E-04	1.31E-02
ZNF473	Eukaryotic Translation Termination	25/1521	93/10891	7.38E-04	1.31E-02
ZNF473	Regulation of mRNA stability by proteins that bind AU-rich elements	24/1521	88/10891	7.47E-04	1.31E-02
ZNF473	RNA Polymerase I Transcription Termination	12/1521	32/10891	7.92E-04	1.37E-02
ZNF473	NIK-->noncanonical NF-kB signaling	18/1521	59/10891	8.09E-04	1.37E-02
ZNF473	Activation of the mRNA upon binding of the cap-binding complex and eIFs, and subsequent binding to 43S	18/1521	59/10891	8.09E-04	1.37E-02
ZNF473	mRNA Splicing - Minor Pathway	16/1521	50/10891	8.76E-04	1.46E-02
ZNF473	Viral mRNA Translation	24/1521	89/10891	8.90E-04	1.47E-02
ZNF473	Transport of Mature Transcript to Cytoplasm	23/1521	84/10891	8.99E-04	1.47E-02
ZNF473	Interleukin-1 family signaling	36/1521	153/10891	9.43E-04	1.52E-02
ZNF473	Deadenylation-dependent mRNA decay	17/1521	55/10891	9.51E-04	1.52E-02
ZNF473	RIP-mediated NFkB activation via ZBP1	8/1521	17/10891	1.07E-03	1.68E-02
ZNF473	Transport of Mature mRNA derived from an	21/1521	75/10891	1.08E-03	1.69E-02

	Intron-Containing Transcript				
ZNF473	Formation of the ternary complex, and subsequently, the 43S complex	16/1521	51/10891	1.11E-03	1.72E-02
ZNF473	CLEC7A (Dectin-1) signaling	26/1521	101/10891	1.18E-03	1.78E-02
ZNF473	ZBP1(DAI) mediated induction of type I IFNs	9/1521	21/10891	1.19E-03	1.78E-02
ZNF473	Transcriptional activation of mitochondrial biogenesis	17/1521	56/10891	1.19E-03	1.78E-02
ZNF473	SRP-dependent cotranslational protein targeting to membrane	28/1521	112/10891	1.27E-03	1.88E-02
ZNF473	Diseases of signal transduction by growth factor receptors and second messengers	83/1521	433/10891	1.35E-03	1.98E-02
ZNF473	GSK3B and BTRC:CUL1-mediated-degradation of NFE2L2	16/1521	52/10891	1.40E-03	2.04E-02
ZNF473	SUMOylation of SUMOylation proteins	12/1521	34/10891	1.47E-03	2.11E-02
ZNF473	Downstream TCR signaling	25/1521	98/10891	1.66E-03	2.37E-02
ZNF473	Polymerase switching on the C-strand of the telomere	10/1521	26/10891	1.72E-03	2.43E-02
ZNF473	Regulation of RUNX2 expression and activity	20/1521	73/10891	1.88E-03	2.62E-02
ZNF473	MyD88-independent TLR4 cascade	27/1521	110/10891	2.04E-03	2.80E-02
ZNF473	TRIF(TICAM1)-mediated TLR4 signaling	27/1521	110/10891	2.04E-03	2.80E-02
ZNF473	Neddylation	50/1521	240/10891	2.07E-03	2.81E-02
ZNF473	Toll Like Receptor 3 (TLR3) Cascade	26/1521	105/10891	2.15E-03	2.86E-02
ZNF473	Global Genome Nucleotide Excision Repair (GG-NER)	22/1521	84/10891	2.15E-03	2.86E-02
ZNF473	mRNA 3'-end processing	17/1521	59/10891	2.24E-03	2.95E-02
ZNF473	Regulation of HSF1-mediated heat shock response	19/1521	69/10891	2.27E-03	2.96E-02
ZNF473	Transcriptional Regulation by TP53	70/1521	362/10891	2.47E-03	3.20E-02
ZNF473	PPARA activates gene expression	28/1521	117/10891	2.55E-03	3.26E-02
ZNF473	Interactions of Rev with host cellular proteins	12/1521	36/10891	2.57E-03	3.27E-02
ZNF473	Cellular response to starvation	35/1521	156/10891	2.62E-03	3.30E-02
ZNF473	Cellular response to hypoxia	20/1521	75/10891	2.67E-03	3.31E-02
ZNF473	SCF-beta-TrCP mediated degradation of Emi1	16/1521	55/10891	2.68E-03	3.31E-02
ZNF473	Signaling by the B Cell Receptor (BCR)	27/1521	112/10891	2.69E-03	3.31E-02
ZNF473	PERK regulates gene expression	11/1521	32/10891	2.92E-03	3.56E-02
ZNF473	Prefoldin mediated transfer of substrate to CCT/TriC	10/1521	28/10891	3.27E-03	3.89E-02
ZNF473	Oxygen-dependent proline hydroxylation of Hypoxia-inducible Factor Alpha	18/1521	66/10891	3.28E-03	3.89E-02
ZNF473	Regulation of lipid metabolism by PPARalpha	28/1521	119/10891	3.30E-03	3.89E-02
ZNF473	Regulation of TP53 Activity through Phosphorylation	23/1521	92/10891	3.30E-03	3.89E-02
ZNF473	RNA Polymerase I Transcription Initiation	14/1521	47/10891	3.82E-03	4.41E-02

ZNF473	Cellular response to chemical stress	41/1521	194/10891	3.83E-03	4.41E-02
ZNF473	Nuclear import of Rev protein	11/1521	33/10891	3.84E-03	4.41E-02
ZNF473	Class I MHC mediated antigen processing & presentation	72/1521	381/10891	3.94E-03	4.50E-02
ZNF473	ABC transporter disorders	20/1521	78/10891	4.36E-03	4.93E-02

Supplementary Table 8. SNPs that showed the strongest associations with CRC risk in TWAS-identified susceptibility loci.

Locus	Gene	Best GWAS SNP	Chr	Pos (hg38)	EA	NEA	GWAS Beta	GWAS SE	GWAS P
2q35	AAMP, ARPC2, GPBAR1, PNKD, TMBIM1	rs2168704	2	218277769	A	G	0.06	0.01	2.95E-09
14q22.1	ABHD12B, NIN, PYGL	rs8004788	14	50909108	T	C	-0.05	0.01	8.53E-06
2q11.2	ACTR1B	rs11692435	2	97658891	A	G	-0.11	0.02	1.09E-05
19q13.33	ASPDH	rs2445828	19	50438550	A	G	-0.06	0.01	5.69E-09
12q13.12	ATF1	rs6580735	12	50271444	T	C	-0.06	0.01	8.15E-09
16p13.3	AXIN1	rs400037	16	286396	A	G	-0.07	0.02	6.07E-06
19q13.2	B9D2	rs2241714	19	41363487	T	C	-0.06	0.01	9.79E-10
10p12.1	BAMBI	rs1761985	10	28805679	T	C	-0.04	0.01	1.13E-05
9q34.2	BRD3	rs11789898	9	134060541	T	G	0.06	0.02	5.32E-05
11q23.1	C11orf53, COLCA2	rs12296076	11	111295779	A	G	-0.10	0.01	3.29E-21
1p34.3	C1orf122, SF3A3, UTP11	rs4072980	1	37990434	A	G	0.06	0.01	1.35E-08
22q13.1	CACNA1I, PDGFB	rs5757573	22	39237617	T	C	-0.08	0.01	7.41E-08
16q22.1	CDH1	rs8056538	16	68768379	A	G	-0.06	0.01	5.78E-07
12q13.12	CERS5, COX14, LIMA1	rs7315690	12	50187707	T	C	0.06	0.01	5.53E-08
15q26.1	CRTC3, IQGAP1	rs11852389	15	90534590	A	G	-0.05	0.01	3.84E-06
9p22.1	DENND4C	rs17818670	9	19331573	A	G	0.05	0.01	3.15E-07
10q21.3	DNA2, RUFY2, SLC25A16	rs10998195	10	68457704	T	C	0.07	0.02	6.60E-06
11q12.2	FADS1, TMEM258	rs4246215	11	61796827	T	G	-0.08	0.01	4.14E-16
11q13.1	FAM89B	rs4099470	11	65552515	T	C	-0.10	0.02	8.95E-08
4q32.1	FNIP2	rs13152601	4	158811622	A	G	0.07	0.02	7.79E-06
19q13.33	FUT2, NTN5, RASIP1	rs601338	19	48703417	A	G	0.06	0.01	1.19E-05
6p22.1	GABBR1, HLA-F	rs9257940	6	29674732	A	G	-0.08	0.02	2.39E-06

7p22.3-p22.2	GNA12	rs798502	7	2750246	A	C	0.05	0.01	5.08E-07
19q13.11	GPATCH1	rs10411210	19	33041394	T	C	-0.11	0.01	3.81E-14
1q25.3	LAMC1	rs10752881	1	183004356	A	G	0.07	0.01	3.10E-12
1q32.1	LMOD1	rs2820313	1	201901093	A	G	-0.07	0.01	1.33E-07
12q13.3	LRP1, TAC3	rs324015	12	57096317	T	C	-0.06	0.01	9.79E-08
17q25.3	METRNL	rs7502442	17	83093262	T	G	0.08	0.01	5.42E-11
15q23	MYO9A	rs4777489	15	72138093	A	G	-0.05	0.01	9.61E-06
17p13.1	NAA38	rs11078711	17	7873896	T	G	0.05	0.01	3.47E-05
5q31.3	NDFIP1	rs1062158	5	142143435	T	C	-0.04	0.01	1.84E-05
14q22.1	NID2	rs1497077	14	52024937	T	C	-0.05	0.01	2.32E-05
17p13.3	NXN	rs1703824	17	910084	A	C	-0.07	0.01	3.32E-12
20q13.13	PREX1	rs6066825	20	48723580	A	G	0.09	0.01	1.39E-18
19q13.43	RPS5	rs11670864	19	58519213	T	G	0.07	0.02	2.51E-05
11p15.4	SBF2	rs4399321	11	10301931	A	G	-0.05	0.01	1.07E-05
3p21.1	SFMBT1	rs2001732	3	52846724	T	C	-0.09	0.02	8.49E-08
6p22.3	SOX4	rs12530233	6	22085429	A	C	0.06	0.01	2.87E-05
7p13	TBRG4	rs7810512	7	45110732	A	C	0.05	0.01	8.88E-06
6p22.1	TRIM26	rs2272874	6	29728468	T	C	-0.06	0.01	9.70E-06
7q22.1	TRIM4	rs2527927	7	99879803	A	G	-0.05	0.01	5.72E-06
1p36.12	WNT4	rs7524102	1	22371954	A	G	0.09	0.01	3.31E-12

Supplementary Table 9. Annotation of putative functional variants in TWAS-identified susceptibility loci.

Chr	Pos (hg38)	LD (r^2)	LD (D')	Variant	Ref	Alt	ASN Freq	EUR Freq	Promoter Histone Mark	Enhancer Histone Mark	DNAse	Protein Bound	Motif Changed	GENCODE gene	dbSNP Functional Annotation
2	218250385	0.97	0.99	rs6736362	C	T	0.60	0.59		BLD, SKIN, GI, HRT, BRN			Pax-4,Pax-5	ARPC2	intronic
2	218255532	0.97	0.99	rs736730	C	T	0.60	0.59	ESC, BLD	FAT, BLD, SKIN, BRN, MUS, GI, BONE	ESDR,LNG,BLD,SKIN,SKIN,BRN,SKIN,LNG			RP11-378A13.1	
2	218255865	0.97	0.99	rs736731	A	G	0.60	0.59	ESC, SKIN, BLD	FAT, BLD, SKIN, BRN, MUS, GI, BONE			E2A,Pou2f2,Pou3f3,Pou5f1,RP58	RP11-378A13.1	
2	218256805	0.96	0.98	rs12999734	A	G	0.59	0.59	ESC, BLD, SKIN, GI	ESDR, IPSC, FAT, BLD, SKIN, BRN, GI, ADRL, MUS, PLCNT, PANC, SPLN			AP-4,Ascl2,HEN1,LBP-1,LUN-1,Nkx2,TCF12	RP11-378A13.1	
2	218256940	0.8	-0.99	rs11677953	G	A	0.40	0.36	ESC, BLD, SKIN, GI	ESC, ESDR, IPSC, FAT, BLD, SKIN, BRN, GI, ADRL, MUS, PLCNT, THYM, HRT, LNG, PANC, SPLN	BLD,BLD		Gcm1,Pou1f1,TATA	RP11-378A13.1	
2	218257367	0.97	0.99	rs10932766	G	A	0.60	0.59	ESC, BLD, SKIN, FAT, GI	ESC, ESDR, IPSC, FAT, BLD, STRM, SKIN, LIV, BRN, GI, ADRL, HRT, MUS, PLCNT, THYM, LNG, PANC, SPLN, VAS	BLD,BLD,BLD,BLD,BLD,BLD	EBF1	CEBPB,Foxf2,Foxj2,Foxl1,Foxq1,Jundm2	2bp 3' of RP11-378A13.1	
2	218258057	0.8	-0.99	rs4674276	A	T	0.40	0.36	BLD, SKIN, FAT, GI	ESC, ESDR, LNG, IPSC, FAT, BLD, STRM, MUS, SKIN, LIV, BRN, GI, ADRL, HRT, PLCNT, THYM, SPLN, VAS			CHOP::CEBPalpha,HNF4,Pax-8	692bp 3' of RP11-378A13.1	
2	218259499	0.97	0.99	rs3731859	G	A	0.60	0.59	IPSC, FAT, STRM, SKIN, BRN, GI, HRT, MUS, BLD	ESC, ESDR, LNG, IPSC, FAT, STRM, BLD, MUS, SKIN, LIV, BRN, GI, ADRL, HRT, PLCNT, THYM, OVRY, PANC, SPLN, VAS, BONE	ESDR,ESDR,ESDR,LNG,HRT,MUS,MUS,GI,OVRY,PANC,MUS		RXRA	GPBAR1	
2	218260198	0.97	0.99	rs13003334	T	A	0.60	0.59	IPSC, FAT, STRM, SKIN, BRN, GI, HRT, MUS, SPLN,	ESC, ESDR, LNG, IPSC, FAT, STRM, BLD, SKIN, LIV, BRN, GI, ADRL, HRT, KID, MUS, PLCNT, THYM, OVRY, PANC, SPLN,	MUS		PPAR	GPBAR1	

									BLD	VAS					
2	218261086	0.97	0.99	rs11554825	C	T	0.60	0.59	IPSC, FAT, BLD, SKIN, BRN, GI, HRT, PLCNT, MUS, SPLN	ESC, ESDR, LNG, IPSC, FAT, STRM, BLD, MUS, SKIN, LIV, BRN, GI, ADRL, HRT, KID, PLCNT, THYM, OVRY, PANC, SPLN	ESC,IPSC,HRT,KID,LNG,MUS,MUS,PLCNT,GI,GI,PANC,MUS,GI,LIV,MUS,MUS,BLD,SKIN	CTCF,RAD21	GR,Hmx,VDR	GPBAR1	5'-UTR
2	218268008	0.81	-1	rs4674279	G	A	0.36	0.36	BLD, SKIN, BRN, GI, CRVX	ESDR, FAT, BRST, BLD, STRM, SKIN, LIV, BRN, GI, ADRL, MUS, PLCNT, THYM, PANC, CRVX			ERalpha-a,Nrf-2,TCF11::MafG	AAMP	intronic
2	218270205	0.81	-0.99	rs1877714	A	G	0.37	0.36	ESC, ESDR, LNG, IPSC, FAT, STRM, BRST, BLD, MUS, BRN, SKIN, VAS, LIV, GI, ADRL, HRT, KID, PANC, PLCNT, THYM, OVRY, SPLN, CRVX, BONE	ESDR, BRST, BLD, SKIN, FAT, LIV, GI, BRN, MUS, PLCNT, THYM, LNG, VAS	ESC,ESDR,ESDR,ESDR,ESDR,ESC,LNG,IPSC,IPSC,BRST,BLD,BLD,BLD,BLD,BLD,BLD,BLD,SKIN,SKIN,SKIN,SKIN,ADRL,BRN,BRN,HRT,GI,GI,KID,LNG,MUS,MUS,PLCNT,GI,THYM,GI,OVRY,PANC,MUS,GI,LNG,BLD,CRVX,LIV,BRST,MUS,MUS,VAS,BLD,BLD,BRN,SKIN,SKIN,LNG	POL2,CTCF,CFOS,CHD2,GABP,OCT2,PAX5C20,PBX3,POU2F2,PU1,SP1,TAF1,USF2,POL24H8,ELK4,IRF3,POL2S2,ELF1,HEY1,RAD21,SREBP1,TBP,CCNT2,EGR1,IRF1,NFYA,NFYB,SP2,USF1,CMYC,HAE2F1,ZNF263	CTCF,Nanog,RXRA	AAMP	
2	218270227	0.99	0.99	rs13419763	C	T	0.63	0.59	ESC, ESDR, LNG, IPSC, FAT, STRM, BRST, BLD, MUS, BRN, SKIN, VAS, LIV, GI, ADRL, HRT, KID, PANC, PLCNT, THYM, OVRY, SPLN,	ESDR, BRST, BLD, SKIN, FAT, LIV, GI, BRN, MUS, PLCNT, THYM, LNG, VAS	ESC,ESDR,ESDR,ESDR,ESDR,ESC,LNG,IPSC,IPSC,BRST,BLD,BLD,BLD,BLD,BLD,BLD,BLD,SKIN,SKIN,SKIN,SKIN,A	CTCF,CFOS,CHD2,GABP,PBX3,POL2,PU1,SP1,USF2,PAX5C20,TAF1,USF1,ELK4,BAF170,IRF3,POL2	E2F,Irf,SP1	AAMP	

									CRVX, BONE		DRL,BRN,BRN,HRT,GI,GI,KID,LNG,MUS,MUS,PLCNT,GI,THYM,GI,OVRY,PANC,MUS,GI,LNG,BLD,CRVX,LIV,BRST,MUS,MUS,VAS,BLD,BLD,BRN,SKIN,SKIN,LNG	S2,ELF1,RAD21,SREBP1,TBP,CCNT2,EGR1,IRF1,NFYA,NFYB,SP2,HAE2F1,ZNF263			
2	218272165	0.8	-0.99	rs3834139	TAA	T	0.36	0.36	ESC, ESDR, LNG, IPSC, FAT, STRM, BRST, BLD, MUS, BRN, SKIN, VAS, LIV, GI, HRT, PANC, PLCNT, THYM, CRVX, BONE	ESC, ESDR, LNG, IPSC, FAT, STRM, BRST, BLD, MUS, BRN, SKIN, VAS, LIV, GI, ADRL, HRT, PANC, PLCNT, THYM, OVRY, SPLN, CRVX, BONE	BLD,SKIN,GI,GI,OVRY,MUS,BRST		AP-2,E2F,MZF1::1-4	PNKD	intronic
2	218273902	0.81	-0.99	rs6758540	A	G	0.37	0.37	FAT, BRST, BLD, SKIN, BRN, GI, PLCNT, MUS, CRVX, BONE	ESDR, FAT, ESC, STRM, BRST, BLD, MUS, SKIN, LIV, BRN, GI, HRT, PLCNT, THYM, LNG, PANC, SPLN, CRVX, BONE	BLD,GI		EBF,Irf,PU.1,TFIIA	PNKD	intronic
2	218274217	0.99	1	rs4791	C	T	0.63	0.59	FAT, SKIN, BRN, GI, PLCNT, MUS, CRVX	ESDR, FAT, STRM, BRST, BLD, MUS, SKIN, LIV, BRN, GI, HRT, PLCNT, THYM, LNG, PANC, SPLN			Cdx2,DMRT2,DMRT3,Foxq1,Hoxa10,Hoxa9,Hoxb13,Hoxb9,Hoxc10,Hoxc9,Hoxd10,Pou2f2,Pou6f1,Sox	PNKD	3'-UTR
2	218275565	0.81	-0.99	rs2292550	G	C	0.37	0.37	SKIN, BRN, GI, HRT, SPLN	FAT, STRM, BRST, BLD, SKIN, LIV, BRN, GI, HRT, MUS, PLCNT, LNG, PANC, THYM, SPLN	BLD,MUS,BLD,SKIN		NRSF,Sin3Ak-20	PNKD	synonymous
2	218276735	0.99	-1	rs4674280	C	G	0.37	0.41	SKIN, BRN, GI, SPLN	FAT, BRST, BLD, STRM, SKIN, BRN, GI, MUS, PLCNT, LNG, PANC, HRT, SPLN	SKIN			PNKD	intronic

2	218277768	0.81	-1	rs2271543	C	T	0.36	0.36	ESDR, SKIN, BRN, GI, SPLN	IPSC, FAT, BRST, BLD, STRM, SKIN, BRN, GI, MUS, PLCNT, LNG, PANC, HRT, SPLN			Ik-1,NF-kappaB	PNKD	intronic
2	218277769	1	1	rs2168704	G	A	0.63	0.59	ESDR, SKIN, BRN, GI, SPLN	IPSC, FAT, BRST, BLD, STRM, SKIN, BRN, GI, MUS, PLCNT, LNG, PANC, HRT, SPLN			GLI,NF-kappaB	PNKD	intronic
2	218277871	0.82	-1	rs3817266	C	T	0.36	0.36	ESDR, SKIN, BRN, GI, SPLN	ESDR, IPSC, FAT, BRST, BLD, STRM, SKIN, BRN, GI, MUS, PLCNT, LNG, PANC, HRT, THYM, SPLN			CCNT2,MAZR,STAT, Zfp410	PNKD	intronic
2	218278050	0.82	1	rs6707559	C	T	0.63	0.64	ESDR, SKIN, BRN, GI, SPLN	ESDR, IPSC, FAT, BRST, BLD, STRM, SKIN, BRN, GI, MUS, PLCNT, LNG, PANC, HRT, THYM, SPLN	SKIN		BCL,Elf5,Ets,GATA,NERF1a,PU.1,Pax-5,RXRA,TATA,p300	PNKD	intronic
2	218278137	1	1	rs13429408	C	A	0.63	0.59	ESDR, SKIN, BRN, GI, SPLN	ESDR, IPSC, FAT, BRST, BLD, STRM, SKIN, BRN, GI, MUS, PLCNT, LNG, PANC, HRT, SPLN	SKIN			PNKD	intronic
2	218278506	0.82	-1	rs2292551	G	A	0.36	0.36	ESDR, SKIN, BRN, GI, SPLN	ESDR, IPSC, FAT, BRST, BLD, STRM, SKIN, BRN, GI, MUS, PLCNT, LNG, PANC, HRT, SPLN	SKIN		HEN1,Nr2f2,Pax-5	PNKD	intronic
		0.82	-1	rs63368160	T	C	0.36	0.36	ESDR, SKIN, GI, SPLN	ESDR, IPSC, FAT, BRST, BLD, STRM, SKIN, BRN, GI, MUS, PLCNT, LNG, PANC, HRT, SPLN	SKIN		AP-1,LBP-1	PNKD	intronic
2	218279366	0.81	-1	rs12987180	G	A	0.36	0.36	ESDR, SKIN, GI, SPLN	ESDR, BRST, BLD, STRM, SKIN, FAT, BRN, GI, MUS, PLCNT, LNG, PANC, HRT, SPLN			PPAR,Smad3	PNKD	intronic
2	218279426	0.81	-1	rs12987219	G	A	0.37	0.36	ESDR, SKIN, GI, SPLN	ESDR, BRST, BLD, STRM, SKIN, FAT, BRN, GI, MUS, PLCNT, LNG, PANC, HRT, SPLN	LIV		Ets,PTF1-beta,Pax-4	PNKD	intronic
2	218279507	0.81	-1	rs4674282	A	T	0.36	0.36	ESDR, SKIN, GI, SPLN	ESDR, IPSC, BRST, BLD, STRM, SKIN, FAT, BRN, GI, MUS, PLCNT, LNG, PANC, HRT, SPLN	SKIN		Gm397,NF-I,TLX1::NFIC	PNKD	intronic
2	218279531	0.81	-1	rs4674283	G	A	0.36	0.36	ESDR, SKIN, GI, SPLN	ESDR, IPSC, BRST, BLD, STRM, SKIN, FAT, BRN, GI, MUS, PLCNT, LNG, PANC, HRT, SPLN			BCL,PU.1,Pax-5,SP1, STAT	PNKD	intronic
2	218279775	0.81	-1	rs2045434	A	G	0.36	0.36	ESDR, SKIN, GI, MUS, SPLN	ESDR, IPSC, BRST, BLD, STRM, SKIN, FAT, BRN, GI, MUS, PLCNT, LNG, PANC, HRT, SPLN				PNKD	intronic
		0.81	-1	rs14556299	AAG	A	0.35	0.36	ESDR, SKIN, GI, MUS, SPLN	ESDR, IPSC, BRST, BLD, STRM, SKIN, FAT, BRN, GI, MUS,			KAP1,Roaz	PNKD	intronic

				5						PLCNT, LNG, PANC, HRT, SPLN					
2	218281476	0.82	-1	rs10932768	T	A	0.36	0.36	FAT, BRST, BLD, MUS, SKIN, BRN, GI, BONE	ESDR, LNG, FAT, STRM, BRST, BLD, MUS, BRN, SKIN, VAS, LIV, GI, PLCNT, THYM, HRT, PANC, SPLN, BONE	MUS		Foxd1	PNKD	intronic
2	218281884	0.82	-1	rs7585702	T	C	0.36	0.36	FAT, BRST, BLD, STRM, MUS, SKIN, BRN, GI, BONE	ESDR, LNG, IPSC, FAT, STRM, BRST, BLD, MUS, BRN, SKIN, VAS, LIV, GI, ADRL, HRT, PLCNT, THYM, PANC, SPLN, BONE	SKIN		CTCF,HEN1,Rad21,ZEB1	PNKD	intronic
2	218282080	1	1	rs2292553	G	A	0.63	0.59	IPSC, FAT, BRST, BLD, STRM, MUS, SKIN, BRN, GI, HRT, LNG, PLCNT, BONE	ESDR, LNG, IPSC, FAT, STRM, BRST, BLD, MUS, BRN, SKIN, VAS, LIV, GI, ADRL, HRT, PLCNT, THYM, PANC, SPLN, BONE	MUS		AP-2,BCL,BDP1,BHLHE40,CCNT2,CTCFL,CTCF,E2F,EBF,ELF1,Ets,GR,Irf,Nrf1,RXRA,Rad21,Roaz,SMC3,SP1,Sin3Ak-20,Sp4,TATA,TR4,WT1,YY1,Zic,Znf143	PNKD	missense
2	218282145	0.82	-1	rs2292554	T	C	0.37	0.36	IPSC, FAT, BRST, BLD, STRM, MUS, SKIN, BRN, GI, HRT, LNG, PLCNT, BONE	ESDR, LNG, IPSC, FAT, ESC, STRM, BRST, BLD, MUS, BRN, SKIN, VAS, LIV, GI, ADRL, HRT, PLCNT, THYM, PANC, SPLN, BONE			THAP1,YY1	PNKD	5'-UTR
2	218282159	0.81	-1	rs2292555	G	C	0.36	0.36	IPSC, FAT, BRST, BLD, STRM, MUS, SKIN, BRN, GI, HRT, LNG, PLCNT, BONE	ESDR, LNG, IPSC, FAT, ESC, STRM, BRST, BLD, MUS, BRN, SKIN, VAS, LIV, GI, ADRL, HRT, PLCNT, THYM, PANC, SPLN, BONE	BLD		ATF3,CACD,MZF1::1-4,Maf,Pou2f2,Sin3Ak-20,Znf143	PNKD	5'-UTR
2	218282522	0.82	-1	rs10932769	T	G	0.36	0.36	IPSC, FAT, BRST, BLD, STRM, MUS, SKIN, BRN, GI, HRT, LNG, PLCNT, BONE	ESC, ESDR, LNG, IPSC, FAT, STRM, BRST, BLD, MUS, BRN, SKIN, VAS, LIV, GI, ADRL, HRT, KID, PLCNT, THYM, PANC, SPLN, BONE	ESDR,BRST,BRN,HRT,MUS,SKIN			PNKD	intronic
2	218283646	0.82	-1	rs6729058	G	A	0.36	0.36	FAT, BRST, BLD, STRM, MUS, SKIN, BRN, GI, PLCNT, HRT, CRVX, BONE	ESC, ESDR, LNG, IPSC, FAT, STRM, BRST, BLD, MUS, BRN, SKIN, VAS, LIV, GI, ADRL, HRT, KID, PLCNT, THYM, OVRY, PANC, SPLN, BONE	SKIN		Mef2,THAP1	PNKD	intronic
2	218284000	0.99	1	rs62182826	C	A	0.63	0.59	FAT, BRST, BLD, STRM,	ESC, ESDR, LNG, FAT, STRM, BRST, BLD, MUS, BRN, SKIN,			HMG-IY,Hoxb13,Ho	PNKD	intronic

									MUS, SKIN, BRN, GI, PLCNT, HRT, CRVX, BONE	LIV, GI, ADRL, HRT, PLCNT, THYM, OVRY, PANC, SPLN, VAS, BONE			xd10,Pou2f2,Pou3f3		
2	218284440	0.82	-1	rs11692780	A	G	0.36	0.36	ESDR, LNG, FAT, STRM, BRST, BLD, MUS, SKIN, LIV, BRN, GI, PANC, PLCNT, HRT, SPLN, CRVX, BONE	ESC, ESDR, LNG, IPSC, FAT, STRM, BRST, BLD, MUS, BRN, SKIN, VAS, LIV, GI, ADRL, HRT, PLCNT, THYM, OVRY, PANC, SPLN, CRVX, BONE	ESDR,ESDR,BRST,BLD ,BLD,SKIN,SKIN,SKIN, ADRL,GI,GI,GI,MUS,G I,LIV,BRST,BLD,SKIN,S KIN	IRF4,POL2,FOSL2,H NF4A,HNF4G,MAF F,MAFK,RXRA	BDP1,NRSF,Sin3Ak-20	PNKD	intronic
2	218285005	1	1	rs55827343	G	A	0.63	0.59	ESDR, FAT, STRM, BRST, BLD, MUS, SKIN, LIV, BRN, GI, PANC, PLCNT, HRT, SPLN, CRVX, LNG, BONE	ESC, ESDR, LNG, IPSC, FAT, STRM, BRST, BLD, MUS, BRN, SKIN, VAS, LIV, GI, ADRL, HRT, PLCNT, THYM, PANC, SPLN, CRVX, BONE			Sox	PNKD	intronic
2	218285017	1	1	rs56109829	G	A	0.63	0.59	ESDR, FAT, STRM, BRST, BLD, MUS, SKIN, LIV, BRN, GI, PANC, PLCNT, HRT, SPLN, CRVX, LNG, BONE	ESC, ESDR, LNG, IPSC, FAT, STRM, BRST, BLD, MUS, BRN, SKIN, VAS, LIV, GI, ADRL, HRT, PLCNT, THYM, PANC, SPLN, CRVX, BONE			Irf,SP1,TATA,TR4	PNKD	intronic
2	218285488	0.82	-1	rs7559416	A	G	0.36	0.36	ESDR, IPSC, FAT, STRM, BRST, BLD, MUS, SKIN, VAS, LIV, BRN, GI, ADRL, HRT, PANC, PLCNT, SPLN, CRVX, LNG, BONE	ESC, ESDR, LNG, IPSC, FAT, STRM, BRST, BLD, MUS, BRN, SKIN, VAS, LIV, GI, ADRL, HRT, PANC, PLCNT, THYM, SPLN, CRVX, BONE	BLD,BLD,ADRL,MUS,L IV		CTCF,NRSF,RREB-1, SMC3	PNKD	intronic
2	218285515	0.82	-1	rs7559428	A	G	0.36	0.36	ESDR, IPSC, FAT, STRM, BRST, BLD, MUS, SKIN, VAS, LIV, BRN, GI, ADRL, HRT, PANC, PLCNT, SPLN, CRVX, LNG, BONE	ESC, ESDR, LNG, IPSC, FAT, STRM, BRST, BLD, MUS, BRN, SKIN, VAS, LIV, GI, ADRL, HRT, PANC, PLCNT, THYM, SPLN, CRVX, BONE	BLD,ADRL,MUS,LIV		EBF,GR	PNKD	intronic
2	218285584	0.82	-1	rs7559525	A	G	0.36	0.36	ESDR, IPSC, FAT, STRM,	ESC, ESDR, LNG, IPSC, FAT, STRM, BRST, BLD, MUS, BRN,	BLD,ADRL,MUS,MUS		ERalpha-a,Sin3Ak-2	PNKD	intronic

									BRST, BLD, MUS, BRN, SKIN, VAS, LIV, GI, ADRL, HRT, LNG, PANC, PLCNT, SPLN, CRVX, BONE	SKIN, VAS, LIV, GI, ADRL, HRT, PANC, PLCNT, THYM, SPLN, CRVX, BONE			0		
2	218286495	0.82	1	rs2382817	A	C	0.63	0.64	ESC, ESDR, LNG, IPSC, FAT, STRM, BRST, BLD, MUS, BRN, SKIN, VAS, LIV, GI, ADRL, HRT, KID, PANC, PLCNT, THYM, OVRY, SPLN, CRVX, BONE	ESC, ESDR, LNG, IPSC, FAT, STRM, BRST, BLD, MUS, BRN, SKIN, LIV, GI, ADRL, HRT, PANC, PLCNT, THYM, SPLN, CRVX, VAS, BONE	BLD,BLD,BLD,BLD,SKIN,HRT,GI,MUS,MUS,GI,THYM,MUS,SKIN		RXRA	PNKD	intronic
2	218287044	0.8	-0.99	rs13005100	C	T	0.36	0.36	ESC, ESDR, LNG, IPSC, FAT, STRM, BRST, BLD, MUS, BRN, SKIN, VAS, LIV, GI, ADRL, HRT, KID, PANC, PLCNT, THYM, OVRY, SPLN, CRVX, BONE	ESC, ESDR, LNG, IPSC, FAT, STRM, BRST, BLD, MUS, BRN, SKIN, LIV, GI, ADRL, HRT, PANC, PLCNT, THYM, SPLN, CRVX, VAS, BONE	ESC,ESDR,ESDR,ESDR,LNG,BRST,BLD,BLD,BLD,BLD,BLD,BLD,BLD,SKIN,SKIN,SKIN,SKIN,BRN,HRT,GI,GI,KID,LNG,MUS,MUS,PLCNT,GI,THYM,GI,OVRY,PANC,MUS,GI,LNG,BLD,CRVX,LIV,BRST,MUS,MUS,VAS,BLD,BLD,BRN,SKIN,SKIN,LNG	AP2GAMMA,POL2,POL24H8,TAL1,ZNF263,KAP1	Dobox4,p53	PNKD	intronic
2	218287203	0.8	-0.99	rs13031757	T	C	0.36	0.36	ESC, ESDR, LNG, IPSC, FAT, STRM, BRST, BLD, MUS, BRN, SKIN, VAS, LIV, GI, ADRL, HRT, KID, PLCNT, THYM, PANC, SPLN, CRVX,	ESC, ESDR, LNG, IPSC, FAT, STRM, BRST, BLD, MUS, BRN, SKIN, LIV, GI, ADRL, HRT, PANC, PLCNT, THYM, SPLN, CRVX, VAS, BONE	ESC,ESDR,ESDR,ESDR,ESDR,ESC,LNG,IPSC,BRST,BLD,BLD,BLD,BLD,BLD,BLD,BLD,SKIN,SKIN,SKIN,SKIN,ADR	ELF1,POL2,SP1,TBP,NFKB,CJUN,FOSL2,HDAC2,HNF4A,HNF4G,JUND,P300,SIN3AK20,IRF1,KAP1	CTCF,E2A,Myc,TCF12,ZEB1	PNKD	intronic

									BONE		L,HRT,GI,GI,KID,LNG,MUS,MUS,PLCNT,GI,THYM,GI,OVRY,PANC,MUS,GI,LNG,BLD,CRVX,LIV,BRST,MUS,MUS,VAS,BLD,BLD,BRN,SKIN,SKIN,LNG				
2	218290058	0.98	0.99	rs992157	G	A	0.63	0.59	ESC, ESDR, LNG, IPSC, FAT, STRM, BRST, BLD, MUS, SKIN, VAS, LIV, BRN, GI, ADRL, HRT, KID, PANC, PLCNT, THYM, SPLN, CRVX, BONE	ESC, ESDR, LNG, IPSC, FAT, STRM, BRST, BLD, MUS, BRN, SKIN, VAS, LIV, GI, ADRL, HRT, KID, PANC, PLCNT, THYM, SPLN, CRVX, BONE	ESDR,ESDR,BRST,BLD,SKIN,SKIN,SKIN,GI,GI,KID,LNG,PLCNT,GI,GI,OVRY,PANC,MUS,GI,LIV,BRST,BLD,BRN,SKIN,SKIN	EBF1,RFX5,RXRA,USF1,MAX,STAT3,POL2	EWSR1-FLI1,VDR	PNKD	intronic
2	218296026	0.92	0.97	rs56344368	T	C	0.63	0.58	ESC, ESDR, BRST, BLD, BRN, GI	ESC, ESDR, LNG, IPSC, BRST, BLD, SKIN, VAS, LIV, BRN, GI, HRT, KID, MUS, PLCNT, THYM, PANC, CRVX			Elf5,Ets,FEV,GATA,HDAC2,STAT	PNKD	intronic
2	218302840	0.93	0.97	rs13427681	G	C	0.63	0.59		BRST, BLD, SKIN, LIV, GI			Pax-5	PNKD	intronic
2	218303709	0.93	0.97	rs1877712	G	A	0.63	0.59		GI, PANC, LIV			LUN-1,Pitx2	PNKD	intronic
2	218314932	0.92	0.96	rs34001204	CA	C	0.63	0.59	BLD	LNG, BLD, SKIN, GI, PLCNT, THYM, LIV			E2A,GR,HEN1,Myf,TAL1	PNKD	intronic
14	50894838	0.83	0.94	rs142964980	G	A	0.61	0.27	ESC, IPSC	IPSC, ESC, GI			PPAR	ABHD12B	intronic
14	50902202	0.96	0.99	rs35653629	CT	C	0.64	0.28		ESC	BLD		Zfp128	ABHD12B	intronic
14	50905380	0.96	0.99	rs3216001	CA	C	0.64	0.28	ESC, BRN	ESC, SKIN, BRN			GCNF,HMG-IY,HNF4,HNF6,Irf,Pou2f2,RXRA,TEF-1	PYGL	3'-UTR
14	50907707	0.97	1	rs6572710	A	T	0.64	0.28	BLD, BRN	ESC, ESDR, IPSC, STRM, SKIN, BRN			Arid5a,Dbx1,Fox,Fo	PYGL	intronic

													xa,Foxj2		
14	50909079	0.98	1	rs8004768	T	G	0.63	0.28		ESC, ESDR, SKIN			ATF3,Nkx6-1	PYGL	intronic
14	50909108	1	1	rs8004788	T	C	0.57	0.28		ESC, ESDR, SKIN			HNF1,PPAR,TATA	PYGL	intronic
14	50923200	0.8	0.98	rs8005400	G	A	0.53	0.24		FAT, BLD, SKIN, LIV, BRN, HRT, PLCNT, LNG			Pou2f2,TCF12,TEF	PYGL	intronic
14	50923414	0.8	0.98	rs8020787	T	A	0.53	0.24		BLD, SKIN, FAT, LIV, HRT, PLCNT, LNG			LUN-1	PYGL	intronic
14	50926131	0.8	0.98	rs1953872	A	G	0.53	0.24	BLD	FAT, ESC, BLD, LIV, ADRL, PLCNT, THYM	SKIN		Ets,NR4A,Nkx2,RXRA,VDR	PYGL	intronic
2	97646864	0.86	1	rs35505243	A	AT	0.00	0.08	ESC, ESDR, LNG, IPSC, FAT, STRM, BRST, BLD, MUS, BRN, SKIN, VAS, LIV, GI, ADRL, HRT, KID, PANC, PLCNT, THYM, OVRY, SPLN, CRVX, BONE	ESDR, ESC, LNG, IPSC, FAT, STRM, BRST, BLD, MUS, BRN, SKIN, VAS, LIV, GI, ADRL, HRT, PLCNT, THYM, PANC, SPLN, CRVX, BONE	SKIN,MUS,LIV	POL2	HDAC2,Irf,NF-kappaB,NRSF,Pax-4,Sin3Ak-20,ZBRK1	COX5B	intronic
2	97658891	1	1	rs11692435	G	A	0.00	0.09	ESDR, IPSC, SKIN, BRN, SPLN	ESDR, IPSC, ESC, BRST, BLD, SKIN, BRN, GI, HRT, MUS, PANC, PLCNT, SPLN		ZNF263	GR	ACTR1B	missense
		0.83	0.94	rs140153080	CCA	C	0.00	0.10	ESDR, SKIN, GI, SPLN	ESDR, IPSC, BLD, SKIN, GI, MUS, LNG, HRT, SPLN			GLI,GR,NF-E2,SREBP,TBX5,UF1H3BETA,Zic	ZAP70	intronic
19	50438550	1	1	rs2445828	A	G	0.64	0.66	SKIN	MUS, SPLN			GR,Pou2f2	MYBPC2	intronic
19	50438648	0.99	1	rs2463240	A	G	0.64	0.66	SKIN	MUS, SPLN	LNG		ATF3,Arnt,BHLHE40,DEC,Myc,NF-E2,SIRT6,SREBP,Sin3Ak-20,TFE	MYBPC2	intronic
19	50448622	0.91	0.96	rs2463242	A	G	0.66	0.66	ESDR	MUS, SPLN			AP-1,Cdx,Pax-4,Pax-6	MYBPC2	intronic
19	50450524	0.89	0.95	rs10405839	T	C	0.65	0.66	IPSC, SPLN	ESC, IPSC, MUS, LNG, BLD			Ik-1,SETDB1,Sin3Ak-	MYBPC2	intronic

													20		
19	50451493	0.9	0.95	rs12985344	T	C	0.67	0.66	IPSC, SPLN	ESC, IPSC, SKIN, PANC, MUS			CHD2,Ets,Irf,Klf7,NRSF,PU.1,Sin3Ak-20,p300	MYBPC2	intronic
19	50453836	0.86	0.93	rs4802706	C	G	0.67	0.66	ESDR	IPSC, GI, PANC, MUS			LBP-9	MYBPC2	intronic
19	50456944	0.87	0.93	rs1726808	T	C	0.70	0.66	ESDR, SKIN	MUS, SPLN	IPSC		NRSF,Sin3Ak-20,TATA,YY1	MYBPC2	intronic
12	50198830	0.88	0.94	rs11169314	A	T	0.28	0.34	FAT, ESC	ESDR, FAT, STRM, BRST, MUS, SKIN, BRN, GI, PLCNT, BONE			Cart1,DMRT3,DMRT4,DMRT5,Foxf1,Foxj2,Foxl1,HNF1,Mrg1::Hoxa9,Pou6f1,RORalpha1,Sox,Zfp105	LIMA1	intronic
12	50200084	0.86	0.94	rs2009072	G	A	0.28	0.33		ESDR, LNG, FAT, STRM, BRST, SKIN, GI, MUS, PLCNT, OVRY, BONE			ZBTB33	LIMA1	intronic
12	50201480	0.88	0.94	rs11169315	A	T	0.28	0.34	MUS, SKIN, LNG	ESDR, ESC, LNG, IPSC, FAT, STRM, BRST, MUS, SKIN, GI, PLCNT, CRVX, VAS, BRN, BONE	SKIN			LIMA1	5'-UTR
12	50205436	0.89	0.95	rs3861100	A	G	0.28	0.33	SKIN	ESC, ESDR, LNG, FAT, BRST, BLD, SKIN, GI, PLCNT, BRN, BONE			Dbx1,Nanog,Nkx6-1,Pou2f2,Pou3f3,TATA	LIMA1	intronic
12	50205926	0.87	0.95	rs2302900	T	C	0.08	0.33	BRST, SKIN	ESC, ESDR, LNG, FAT, BRST, BLD, SKIN, PLCNT, GI, BRN, BONE	BRST,SKIN,BLD,SKIN		CTCF,DMRT7	LIMA1	intronic
12	50208158	0.9	0.95	rs11169317	G	T	0.28	0.34	SKIN	ESDR, LNG, BRST, BLD, SKIN, BRN, BONE	IPSC,SKIN		GR,NRSF	LIMA1	intronic
12	50208512	0.91	0.95	rs34825838	T	A	0.28	0.33	SKIN	ESDR, LNG, BRST, BLD, MUS, SKIN, BRN, BONE	BLD,SKIN		YY1	LIMA1	intronic
12	50208951	0.92	0.96	rs12811291	G	A	0.28	0.33	SKIN, BONE	ESDR, LNG, BRST, BLD, MUS, SKIN, PLCNT, BRN, BONE	SKIN,SKIN		Cdx,Sox	LIMA1	intronic
12	50209931	0.88	0.96	rs11169319	G	A	0.27	0.33	BLD, SKIN, BONE	ESDR, LNG, FAT, STRM, BRST, BLD, MUS, SKIN, GI, THYM, PLCNT, HRT, CRVX, BRN, BONE			NRSF,Nrf1,Pitx2	LIMA1	intronic

12	50210338	0.89	0.96	rs7308095	G	T	0.27	0.33	BLD, SKIN, BONE	ESDR, LNG, FAT, STRM, BRST, BLD, MUS, SKIN, GI, ADRL, PLCNT, THYM, CRVX, BRN, BONE				LIMA1	intronic
12	50216538	0.91	0.96	rs12425705	T	C	0.27	0.34	FAT, STRM, BRST, BRN, SKIN, GI, BLD, MUS, BONE	LNG, FAT, STRM, BRST, BLD, MUS, BRN, SKIN, VAS, GI, PLCNT, CRVX, BONE	SKIN		DMRT3	LIMA1	intronic
12	50217193	0.92	0.96	rs11169322	C	T	0.27	0.33	FAT, STRM, BRST, BRN, SKIN, GI, BLD, MUS, BONE	LNG, FAT, STRM, BRST, BLD, MUS, BRN, SKIN, VAS, GI, PLCNT, THYM, CRVX, BONE	BLD,CRVX		Ik-2,p300	LIMA1	intronic
12	50217694	0.92	0.96	rs12424691	G	A	0.27	0.33	ESDR, ESC, IPSC, FAT, STRM, BRST, BLD, MUS, BRN, SKIN, LNG, GI, CRVX, BONE	ESDR, ESC, LNG, IPSC, FAT, STRM, BRST, BLD, MUS, BRN, SKIN, VAS, GI, ADRL, PLCNT, THYM, HRT, SPLN, CRVX, BONE	PLCNT,GI,BRST		AP-1,EBF,Gcm1,Rad 21,Roaz,Sin3Ak-20	LIMA1	intronic
12	50217834	0.92	0.96	rs12424713	G	A	0.27	0.33	ESDR, ESC, IPSC, FAT, STRM, BRST, BLD, MUS, BRN, SKIN, GI, LNG, CRVX, BONE	ESDR, ESC, LNG, IPSC, FAT, STRM, BRST, BLD, MUS, BRN, SKIN, GI, ADRL, PLCNT, THYM, HRT, SPLN, CRVX, BONE	LNG,BLD,SKIN,MUS,P LCNT			LIMA1	intronic
12	50218907	0.89	0.95	rs20151881 0	AAA C	A	0.26	0.34	ESDR, ESC, LNG, IPSC, FAT, STRM, BRST, BLD, MUS, BRN, SKIN, GI, HRT, CRVX, BONE	ESC, ESDR, LNG, IPSC, FAT, STRM, BRST, BLD, MUS, BRN, SKIN, GI, ADRL, PLCNT, THYM, HRT, SPLN, CRVX, VAS, BONE	SKIN		FAC1,Foxa,Foxj1,Fo xk1,Foxo,Foxp1,HD AC2,Irf,Nanog,Pax-4 ,RREB-1,Sox	LIMA1	intronic
12	50218908	0.9	0.96	rs20134344 5	AAC	A	0.26	0.34	ESDR, ESC, LNG, IPSC, FAT, STRM, BRST, BLD, MUS, BRN, SKIN, GI, HRT, CRVX, BONE	ESC, ESDR, LNG, IPSC, FAT, STRM, BRST, BLD, MUS, BRN, SKIN, GI, ADRL, PLCNT, THYM, HRT, SPLN, CRVX, VAS, BONE	SKIN		FAC1,Foxa,Foxj1,Fo xk1,Foxo,Foxp1,HD AC2,Irf,Nanog,Pax-4 ,RREB-1,Sox	LIMA1	intronic
12	50220924	0.91	0.96	rs1362983	A	G	0.28	0.33	ESC, ESDR, LNG, IPSC, FAT, STRM, BRST, BLD, MUS, BRN, SKIN, VAS, LIV, GI, ADRL, KID, PANC, PLCNT, THYM, HRT, OVRY, CRVX, BONE	ESC, ESDR, IPSC, BLD, STRM, BRN, SKIN, FAT, VAS, GI, ADRL, HRT, KID, PANC, LNG, PLCNT, THYM, MUS, CRVX	ESDR,BRST,BLD,BLD, BLD,BLD,BLD,BLD,SKI N,SKIN,SKIN,HRT,KID, LNG,MUS,MUS,PLCN T,GI,THYM,OVRY,LN G,BLD,CRVX,BRST,M		CIZ,Pax-4	RP3-405J1 0.4	intronic

											US,MUS,VAS,BLD,BLD,SKIN,SKIN,LNG				
12	50222563	0.92	0.96	rs3812825	A	G	0.28	0.33	ESC, ESDR, LNG, IPSC, FAT, STRM, BRST, BLD, MUS, BRN, SKIN, VAS, LIV, GI, ADRL, HRT, KID, PANC, PLCNT, THYM, OVRY, SPLN, CRVX, BONE	ESC, ESDR, IPSC, STRM, BRST, BLD, BRN, SKIN, FAT, VAS, GI, ADRL, HRT, KID, PANC, LNG, MUS, PLCNT, THYM, SPLN, CRVX	ESDR,ESDR,ESDR,ESDR,ESC,LNG,IPSC,IPSC,BRST,BLD,BLD,BLD,BLD,BLD,BLD,SKIN,SKIN,SKIN,SKIN,ADRL,HRT,GI,KID,LNG,MUS,MUS,PLCNT,GI,THYM,OVRY,PANC,LNG,BLD,CRVX,BRST,MUS,MUS,VAS,BLD,BLD,BRN,SKIN,SKIN,LNG	CTCF,POL2,NFKB,POL24H8,TAF1,TAF7,TBP,ELK4,KAP1,CMYC,POL2B,CEBPB,MAX,MXI1,RAD21,SMC3,E2F6,ELF1,HEY1,NRSF,P300	Irf,SIX5,STAT,p300	RP3-405J10.4	5'-UTR
12	50225835	0.91	0.96	rs2358539	T	C	0.27	0.34		ESDR, BLD, SKIN, GI, MUS, THYM, PLCNT, CRVX			TCF12	RP3-405J10.4	intronic
12	50228077	0.92	0.96	rs7308885	C	A	0.27	0.33					Irf	RP3-405J10.4	intronic
12	50229875	0.91	0.96	rs7314465	G	A	0.27	0.34		ESDR, SKIN, VAS, PLCNT, GI	ESDR,SKIN,PLCNT,CRVX		PU.1,Pax-5,p300	RP3-405J10.4	intronic
12	50230767	0.92	0.96	rs71465002	C	T	0.27	0.33		ESDR, VAS, GI			Mef2,Zfp187	LIMA1	intronic
12	50231275	0.92	0.96	rs12425229	A	C	0.28	0.33		ESDR, BRN, VAS, MUS, GI			CTCF,Rad21	LIMA1	intronic
12	50232282	0.92	0.96	rs10876013	A	T	0.28	0.33		FAT, SKIN, VAS, GI, MUS, THYM, LNG, BLD, CRVX	CRVX,BLD			LIMA1	intronic
12	50235127	0.91	0.96	rs11169331	T	C	0.27	0.34		ESDR, FAT, SKIN, GI, MUS, PLCNT, LNG, SPLN, CRVX, BLD, BRN			ERalpha-a,RXRA	LIMA1	intronic
12	50235829	0.91	0.96	rs11169332	G	A	0.27	0.34		FAT, SKIN, GI, LNG, PLCNT, MUS, SPLN			E2F,Foxl1,HMG-IY,HNF1,Ik-2,NF-AT	LIMA1	intronic

12	50239630	0.91	0.95	rs35576436	ACT	A	0.27	0.33	FAT, MUS, SKIN, BRN, GI, BLD	ESC, ESDR, LNG, IPSC, FAT, STRM, BRST, BLD, SKIN, VAS, LIV, GI, HRT, KID, PANC, MUS, PLCNT, OVRY, SPLN, CRVX	IPSC,BLD	PU1	DMRT5,DMRT7,Fox,Foxa,Foxi1,Foxj1,Foxj2,Foxq1,HDAC2,Irf,Mef2,Sox,Zfp105,p300	LIMA1	intronic
12	50242581	0.91	0.96	rs11169335	A	G	0.27	0.34	ESDR, LNG, FAT, STRM, BLD, MUS, SKIN, BRN, GI, CRVX, BONE	ESC, ESDR, LNG, IPSC, FAT, STRM, BRST, BLD, MUS, SKIN, VAS, LIV, BRN, GI, ADRL, HRT, KID, PANC, PLCNT, THYM, CRVX, BONE	ESDR,SKIN,MUS,PLCNT,OVRY,MUS,GI,CRVX,LIV,MUS,BLD,LNG		Nanog	LIMA1	intronic
12	50243157	0.92	0.96	rs17124562	T	C	0.27	0.33	FAT, STRM, MUS, SKIN, BRN, GI, LNG, CRVX, BONE	ESC, ESDR, LNG, IPSC, FAT, STRM, BRST, BLD, MUS, SKIN, VAS, LIV, BRN, GI, ADRL, KID, PANC, PLCNT, THYM, HRT, CRVX, BONE	ESDR,ESDR,LNG,BRST,SKIN,SKIN,SKIN,SKIN,PLCNT,CRVX,BRST,MUS,MUS,BLD,BRN,SKIN,SKIN,LNG	CFOS,JUND,P300	HES1,Myc,TATA	LIMA1	intronic
12	50243512	0.91	0.96	rs12828340	T	C	0.27	0.33	FAT, STRM, MUS, SKIN, BRN, GI, CRVX, BONE	ESC, ESDR, LNG, IPSC, FAT, STRM, BRST, BLD, MUS, SKIN, VAS, LIV, GI, KID, PANC, PLCNT, HRT, CRVX, BRN, BONE	ESDR,SKIN,SKIN,CRVX,SKIN		EBF,Ik-1,PTF1-beta	LIMA1	intronic
12	50247789	0.91	0.96	rs7979830	C	T	0.28	0.33	FAT, STRM, MUS, SKIN, GI, PLCNT, BLD, CRVX, BRN, BONE	ESDR, ESC, LNG, FAT, STRM, BRST, MUS, BRN, SKIN, VAS, LIV, GI, ADRL, PLCNT, THYM, HRT, PANC, SPLN, BLD, CRVX, BONE	MUS,BLD	TCF4	Foxp1,Nkx2,Pax-2,p53	LIMA1	intronic
12	50252971	0.92	0.96	rs7132551	G	A	0.28	0.34	GI, BLD, SKIN	ESDR, STRM, BRST, SKIN, GI, PANC, PLCNT, LNG, BLD, CRVX, LIV, MUS	PLCNT		AIRE,AP-1	LIMA1	intronic
12	50253441	0.93	0.96	rs7953953	C	T	0.28	0.33	ESC, GI, LNG, BLD	ESDR, BRST, BLD, SKIN, GI, PANC, PLCNT, LNG, CRVX, LIV, MUS	GI,GI,GI,BLD		BDP1,E2F,Nanog,Pou2f2,Pou3f3,Pou5f1,TATA	LIMA1	intronic
12	50255177	0.94	0.97	rs12823506	A	G	0.28	0.33	ESC, GI, BLD, CRVX, SKIN	ESC, ESDR, LNG, FAT, STRM, BRST, BLD, SKIN, VAS, GI, PLCNT, THYM, HRT, PANC, SPLN, CRVX, LIV, MUS, BRN, BONE	BLD,CRVX,BRST		DMRT3,EBF,Zfp410	LIMA1	intronic

12	50256781	0.94	0.97	rs7486747	G	A	0.28	0.33	BRN, GI, LNG	STRM, BRST, SKIN, BRN, GI, PANC, PLCNT, LNG, BLD, CRVX, LIV, MUS				LIMA1	intronic
12	50257673	0.94	0.97	rs11169339	T	A	0.28	0.33	FAT, BRN, GI, LNG, CRVX, SKIN	ESDR, LNG, FAT, STRM, BRST, BLD, MUS, BRN, SKIN, LIV, GI, ADRL, HRT, KID, PANC, PLCNT, THYM, OVRY, SPLN, CRVX, VAS, BONE	ESDR,SKIN,SKIN,LNG, MUS,MUS,LNG,CRVX ,MUS,BLD		Egr-1,Elf5,Ets,GATA, Irf,LXR,PU.1,Pax-5,R XRA,TATA,Tel2,p30 0	LIMA1	intronic
12	50257846	0.94	0.97	rs35768991	G	A	0.28	0.33	FAT, STRM, BRN, GI, LNG, MUS, CRVX, SKIN	ESDR, LNG, FAT, STRM, BRST, BLD, MUS, BRN, SKIN, LIV, GI, ADRL, HRT, KID, PANC, PLCNT, THYM, OVRY, SPLN, CRVX, VAS, BONE	ESDR,LNG,SKIN,SKIN, SKIN,SKIN,ADRL,BRN, HRT,KID,LNG,MUS,M US,PLCNT,GI,THYM, OVRY,PANC,MUS,GI, LNG,BLD,CRVX,MUS, MUS,VAS,BLD,BLD,B RN,SKIN,SKIN,LNG		Pax-5,TATA	LIMA1	intronic
12	50259146	0.93	0.97	rs10783344	T	C	0.28	0.33	FAT, LIV, BRN, GI, LNG, SKIN	ESDR, LNG, FAT, STRM, BRST, MUS, BRN, SKIN, LIV, GI, ADRL, HRT, PANC, OVRY, PLCNT, CRVX, VAS, BLD, BONE			SETDB1,Znf143	LIMA1	intronic
12	50260192	0.93	0.96	rs1972611	T	C	0.28	0.33	FAT, GI, BLD, SKIN	ESDR, LNG, IPSC, FAT, STRM, BRST, BLD, MUS, BRN, SKIN, LIV, GI, ADRL, HRT, PANC, OVRY, PLCNT, CRVX, BONE	ESDR,IPSC,GI,LNG,M US,PLCNT,OVRY,PAN C,MUS,GI,BRST,BLD	ELF1,CEBPB	GCM,Pax-5	LIMA1	intronic
12	50263949	0.99	1	rs7956468	C	G	0.28	0.34		STRM, GI, PLCNT, MUS, HRT			EBF	LIMA1	intronic
12	50269144	1	1	rs11169345	T	C	0.28	0.33		ESC, FAT, BRN, GI, PLCNT, MUS			CEBPA,CEBPB,Dbx1, Hmx,Hoxd8,Isl2,LBP -1,Mef2,Ncx,Nkx2,N kx3,TEF	LIMA1	intronic
12	50270073	1	1	rs11169347	A	G	0.28	0.33	BRST	ESC, ESDR, FAT, GI, LNG, MUS, HRT, BLD			Mef2,SIX5	LIMA1	intronic
12	50271444	1	1	rs6580735	T	C	0.28	0.33	FAT, STRM, BRST, LIV, BRN,	ESC, ESDR, LNG, IPSC, FAT, STRM, BRST, MUS, BRN, SKIN,	GI,OVRY,GI,LIV		EWSR1-FLI1,Foxa,H	LIMA1	intronic

									GI, PLCNT, HRT, LNG, BLD, BONE	LIV, GI, ADRL, PANC, PLCNT, CRVX, VAS, BLD, BONE			DAC2,PRDM1,SP1		
12	50272163	0.99	1	rs11169348	G	T	0.28	0.34	LNG, FAT, STRM, BRST, MUS, BRN, VAS, LIV, GI, HRT, PANC, OVRY, PLCNT, BLD, SKIN, BONE	ESC, ESDR, LNG, FAT, STRM, BRST, BLD, MUS, BRN, SKIN, LIV, GI, ADRL, PANC, PLCNT, HRT, CRVX, VAS, BONE	ESDR,ESDR,LNG,SKIN,SKIN,SKIN,GI,KID,LNG,MUS,MUS,PLCNT,GI,THYM,GI,OVRY,MUS,GI,CRVX,MUS,MUS,BLD,BRN,SKIN,LNG	CEBPB,IRF1,STAT1,STAT2,STAT3	AFP1	LIMA1	intronic
12	50274361	1	1	rs7311378	G	A	0.28	0.33	GI, LNG	FAT, BRST, SKIN, GI, ADRL, PANC, LNG, PLCNT, HRT, OVRY, MUS, SPLN, CRVX, LIV	ADRL,GI,LIV	TCF4	Pou2f2,Pou6f1,ZBRK1	LIMA1	intronic
12	50274416	1	1	rs7311491	G	A	0.28	0.33	GI, LNG	FAT, BRST, SKIN, GI, ADRL, PANC, LNG, PLCNT, HRT, OVRY, MUS, SPLN, CRVX, LIV	GI,LIV	TCF4	Foxp1,Hdx,Mef2,Mrg	LIMA1	intronic
12	50274755	1	1	rs2111988	C	T	0.28	0.33	GI, LNG, CRVX	IPSC, FAT, BRST, BLD, SKIN, GI, ADRL, PANC, LNG, PLCNT, HRT, MUS, SPLN, CRVX, LIV	IPSC		Pax-5,Pax-8	LIMA1	intronic
12	50275228	0.99	1	rs35535298	G	A	0.28	0.33	GI, CRVX	IPSC, FAT, BRST, BLD, SKIN, GI, ADRL, PANC, PLCNT, HRT, LNG, MUS, SPLN, CRVX, LIV			TCF12	LIMA1	intronic
12	50276777	0.99	1	rs11169349	T	C	0.28	0.34	GI	ESDR, LNG, IPSC, FAT, STRM, BRST, BLD, SKIN, GI, ADRL, PANC, PLCNT, HRT, OVRY, MUS, SPLN, CRVX, BONE	GI		AP-1,Maf	LIMA1	intronic
12	50277587	0.99	1	rs11169350	T	C	0.28	0.34	FAT, GI	ESDR, LNG, IPSC, FAT, STRM, BRST, BLD, BRN, SKIN, GI, ADRL, PANC, PLCNT, HRT, OVRY, MUS, SPLN, CRVX, LIV, VAS, BONE	ESDR,SKIN,SKIN,PLCNT,MUS,CRVX,BRST,MUS,SKIN		AP-1,CEBPB,E2F,Irf,Lhx8,NF-Y,Pbx3,RFX5,SP2	LIMA1	intronic
12	50278431	0.99	0.99	rs11169351	C	A	0.27	0.33	GI	ESDR, BRN, SKIN, GI, PANC, PLCNT, SPLN, CRVX, VAS			Cart1,Foxp1,Maf,Sox	LIMA1	intronic
12	50280266	0.98	1	rs67138019	C	T	0.24	0.33	FAT, GI, MUS	FAT, SKIN, BRN, GI, PANC, PLCNT, MUS, BONE			ATF3,E2F,ELF1,Maf,NF-E2,Nanog,Pax-5,UF1H3BETA	LIMA1	intronic

12	50280970	1	1	rs10876014	C	T	0.28	0.33	IPSC, FAT, ESC, STRM, BRST, BLD, BRN, SKIN, GI, PLCNT, HRT, MUS, BONE	ESC, FAT, BRST, BLD, SKIN, BRN, GI, PANC, PLCNT, MUS, CRVX, LIV, LNG, BONE	GI,GI		NF-AT,TATA	LIMA1	intronic
12	50283723	0.99	1	rs10876015	T	C	0.28	0.34	ESC, ESDR, LNG, IPSC, FAT, STRM, BRST, BLD, MUS, BRN, SKIN, VAS, LIV, GI, ADRL, HRT, KID, PANC, PLCNT, THYM, OVRY, SPLN, CRVX, BONE	ESC, ESDR, IPSC, STRM, BRST, BLD, MUS, BRN, SKIN, FAT, LIV, GI, ADRL, HRT, PANC, LNG, PLCNT, THYM, SPLN, CRVX, VAS, BONE	ESC,ESDR,ESDR,ESDR,ESDR,ESC,LNG,IPSC,IPSC,BRST,BLD,BLD,BLD,BLD,BLD,BLD,SKIN,SKIN,SKIN,SKIN,ADRL,BRN,BRN,HRT,GI,GI,KID,LNG,MUS,MUS,PLCNT,GI,THYM,GI,OVRY,PANC,MUS,GI,LNG,BLD,CRVX,LIV,BRST,MUS,MUS,VAS,BLD,BLD,BRN,SKIN,SKIN,LNG	POL2,CTCF,EGR1,ELF1,OCT2,POL24H8,POU2F2,RAD21,YY1,TAF1,NFKB,AP2ALPHA,AP2GAMMA,E2F6,INI1,MXI1,POL2S2,TBP,HEY1,CMYC,GTF2F1,HMGN3,JUND,MAX,POL2B,SMC3,E2F4,P300		152bp 5' of LIMA1	
12	50284576	1	1	rs12832940	G	A	0.28	0.33	ESC, ESDR, LNG, IPSC, FAT, STRM, BRST, BLD, MUS, BRN, SKIN, VAS, LIV, GI, ADRL, HRT, KID, PLCNT, THYM, OVRY, PANC, SPLN, CRVX, BONE	ESC, ESDR, LNG, IPSC, FAT, STRM, BRST, BLD, MUS, BRN, SKIN, VAS, LIV, GI, ADRL, HRT, PANC, PLCNT, THYM, OVRY, SPLN, CRVX, BONE	IPSC,BLD,BLD,HRT,PANC,MUS,CRVX,MUS,BLD	SRF,USF1,CFOS,HEY1,NFYA,POL2,POL24H8,TBP	NF-kappaB,Zic	1kb 5' of LIMA1	
12	50284851	0.99	1	rs35933908	G	C	0.28	0.34	ESC, ESDR, LNG, IPSC, FAT, STRM, BRST, BLD, SKIN, LIV, BRN, GI, ADRL, KID, PLCNT, THYM, HRT, PANC, MUS,	ESC, ESDR, IPSC, FAT, STRM, BRST, BLD, MUS, BRN, SKIN, VAS, LIV, GI, ADRL, HRT, PANC, LNG, PLCNT, THYM, OVRY, SPLN, CRVX, BONE			HNF4,Nkx2,RXRA	1.3kb 5' of LIMA1	

									SPLN, CRVX, BONE						
12	50285635	0.92	1	rs6580736	T	C	0.66	0.35	ESDR, FAT, BLD, LIV, BRN, GI, LNG, HRT, MUS, BONE	ESDR, FAT, STRM, BRST, BLD, MUS, BRN, SKIN, VAS, LIV, GI, PANC, LNG, THYM, OVRY, PLCNT, HRT, SPLN, CRVX			AIRE,Nanog,Sox	2.1kb 5' of LIMA1	
12	50285827	1	1	rs7305995	C	G	0.28	0.33	FAT, BLD, LIV, BRN, GI, LNG, HRT, MUS, BONE	ESDR, FAT, STRM, BRST, BLD, SKIN, VAS, LIV, BRN, GI, PANC, LNG, MUS, THYM, OVRY, PLCNT, HRT, SPLN, CRVX			Foxa,Foxd1,Foxo,HDAC2,HNF1,TCF12	2.3kb 5' of LIMA1	
		1	1	rs74382385	G	A	0.28	0.33	GI, LNG, MUS, BLD	BRST, BLD, SKIN, FAT, BRN, GI, PANC, MUS, THYM, HRT, LNG, OVRY, PLCNT, SPLN, CRVX, LIV				2.8kb 5' of LIMA1	
12	50286793	0.94	0.98	rs7974648	A	G	0.28	0.33	GI	BRST, BLD, SKIN, FAT, BRN, GI, MUS, HRT, LNG, SPLN			Eomes,HNF4,SREBP	3.2kb 5' of LIMA1	
12	50287540	0.99	1	rs10876016	G	C	0.28	0.34		BRST, BLD, SKIN, FAT, GI, MUS, HRT, LNG, SPLN	PLCNT,MUS,SKIN		SZF1-1,Spz1	4kb 5' of LIMA1	
12	50287756	0.99	1	rs10876017	C	T	0.28	0.33		BRST, BLD, FAT, GI, MUS, HRT, SPLN			Foxj1,Foxo	4.2kb 5' of LIMA1	
12	50289962	0.99	0.99	rs7311973	T	C	0.28	0.33					Foxk1,Foxo,Foxp1,GR,HDAC2,RREB-1,Sox,Zfp105	6.4kb 5' of LIMA1	
12	50290033	0.99	1	rs28628775	G	T	0.28	0.33					Pax-2	6.5kb 5' of LIMA1	
12	50291958	0.99	1	rs7304445	A	C	0.28	0.33					Dobox4,Pou2f2,Pou3f3,Sox	8.4kb 5' of LIMA1	
12	50291970	0.99	0.99	rs9705460	T	G	0.27	0.33					Dobox4,E2F	8.4kb 5' of LIMA1	
12	50292729	0.92	1	rs12426444	G	A	0.28	0.35			LNG		LXR,Pax-4	9.2kb 5' of LIMA1	
12	50293377	1	1	rs11838347	A	G	0.28	0.33		MUS				9.8kb 5' of LIMA1	

12	50294234	1	1	rs7135777	C	G	0.27	0.33		MUS			CEBPB,Nr2f2,TCF12	11kb 5' of LIMA1	
12	50296202	0.99	1	rs11169357	C	G	0.28	0.33	GI, SKIN	BLD, SKIN, FAT, MUS, HRT, GI, SPLN			PTF1-beta,YY1	13kb 5' of LIMA1	
12	50296671	0.93	0.99	rs7315955	T	C	0.27	0.32	GI, SKIN	BLD, SKIN, FAT, GI, MUS, HRT, PANC, SPLN, LNG, VAS	MUS		TCF12,p53	13kb 5' of LIMA1	
12	50297378	0.81	-0.99	rs200854456	A	ATT	0.34	0.62	FAT	ESC, FAT, BLD, SKIN, GI, ADRL, MUS, PLCNT, LNG, VAS			Cdx,Dbx1,Evi-1,Fox,Foxa,Foxd3,Foxi1,Foxj1,Foxj2,Foxk1,Foxl1,Foxm1,Foxo,Foxp1,HDAC2,HMG-IY,HNF1,Hoxa10,Hoxa9,Irf,Mef2,Ncx,Pax-4,Pou2f2,TATA,Zfp105,p300	14kb 5' of LIMA1	
12	50297381	0.81	-0.99	rs202006386	TA	T	0.34	0.62	FAT	ESC, FAT, BLD, SKIN, GI, ADRL, MUS, PLCNT, LNG, VAS			Cdx,Dbx1,Evi-1,Fox,Foxa,Foxd3,Foxi1,Foxj1,Foxj2,Foxk1,Foxl1,Foxm1,Foxo,Foxp1,HDAC2,HMG-IY,Irf,Ncx,SIX5,Smad,TATA,Zfp105,p300	14kb 5' of LIMA1	
12	50297526	0.98	0.99	rs11169359	T	C	0.28	0.33	FAT	ESC, FAT, BLD, SKIN, GI, ADRL, MUS, HRT, LNG, PLCNT, SPLN, VAS			Pax-4	14kb 5' of LIMA1	
12	50297691	1	1	rs11169360	T	A	0.28	0.33	FAT	ESC, ESDR, IPSC, FAT, BLD, SKIN, GI, ADRL, MUS, THYM, HRT, LNG, PLCNT, SPLN, VAS			DMRT1,DMRT4,DMRT5,HNF1,STAT,Sox	14kb 5' of LIMA1	

12	50299583	0.99	1	rs7488682	G	A	0.28	0.34					Foxp3,ZBTB33	16kb 5' of LIMA1	
12	50299787	0.99	1	rs6580737	C	G	0.28	0.34					Duxl,Elf3,Elf5,NF-Y,Pbx-1,SP2,SRF,STAT,TATA	16kb 5' of LIMA1	
12	50300580	1	1	rs7980911	A	G	0.27	0.33					AIRE,NF-E2,RORalpha1,TCF12,Zfp410	17kb 5' of LIMA1	
12	50302048	0.86	-1	rs12821504	G	C	0.33	0.63		SKIN, LNG				18kb 5' of LIMA1	
12	50304122	0.95	0.99	rs11169367	A	G	0.28	0.33	ESC, FAT, LNG, BLD	ESDR, LNG, FAT, STRM, BRST, MUS, SKIN, GI, SPLN, BLD, BRN, BONE	BLD		FAC1,Foxp1,HDAC2,SIX5	21kb 5' of LIMA1	
12	50305712	0.92	0.99	rs7315871	C	T	0.08	0.32		ESDR, LNG, FAT, STRM, BRST, SKIN, GI			E2A,ELF1,HEN1,Maf,Mxi1,Myc,Myf,NRSF,TAL1	21kb 3' of FAM186A	
12	50306895	0.88	0.96	rs200782572	T	TA	0.36	0.32		SKIN			Cdx,Dbx1,Evi-1,Foxa,Foxf1,Foxi1,Foxj1,Foxj2,Foxl1,Foxo,Foxp1,HDAC2,Ncx,Pou3f2,Pou3f4,TATA,p300	19kb 3' of FAM186A	
12	50306896	0.84	0.93	rs79043170	T	A	0.36	0.33		SKIN			Cdx,Dbx1,Evi-1,Foxa,Foxd3,Foxf1,Foxi1,Foxj1,Foxj2,Foxl1,Foxo,Foxp1,HDAC2,Ncx,Pou3f2,Pou3f4,TATA,Zfp105,p300	19kb 3' of FAM186A	

12	50306900	0.91	0.99	rs112456855	T	A	0.26	0.32		SKIN			Cdx,Dbx1,Foxa,Foxd3,Foxo,Foxp1,HDAC2,HMG-IY,Zfp105,p300	19kb 3' of FAM186A	
12	50307248	0.9	-0.99	rs7485486	G	A	0.35	0.65		SKIN			ZID	19kb 3' of FAM186A	
12	50311905	0.98	0.99	rs11169369	C	A	0.28	0.34	MUS, LNG	LNG, FAT, STRM, MUS, SKIN, PANC, GI, BLD, BRN, BONE			Mef2,TATA	14kb 3' of FAM186A	
12	50311932	0.92	-1	rs6580738	T	C	0.35	0.65	MUS, LNG	LNG, FAT, STRM, MUS, SKIN, PANC, GI, BLD, BRN, BONE	OVRY		Cdx,Duxl,Fox,Foxa,Foxj1,Zfp105	14kb 3' of FAM186A	
12	50312089	0.99	0.99	rs11169370	T	C	0.28	0.33	MUS, LNG	LNG, FAT, STRM, MUS, SKIN, GI, PANC, BLD, BRN, BONE	MUS,MUS		CACD,Foxo,HNF1,Hoxd8,Ncx	14kb 3' of FAM186A	
12	50312563	0.9	-1	rs11169371	T	G	0.35	0.64	MUS, LNG	ESDR, LNG, FAT, STRM, BRST, MUS, SKIN, GI, PANC, BLD, BRN, BONE	ESDR		ATF3,HEN1,SREBP	14kb 3' of FAM186A	
12	50313665	0.98	0.99	rs35209607	C	T	0.28	0.33	LNG, SKIN	LNG, FAT, STRM, BRST, MUS, SKIN, GI, THYM, CRVX, BONE	BRST,SKIN,PLCNT		Nkx3	13kb 3' of FAM186A	
12	50314070	0.91	-0.99	rs10783349	G	A	0.35	0.65		LNG, FAT, STRM, BRST, SKIN, GI, MUS, CRVX, BONE			ATF3,CDP,E2F,FXR	12kb 3' of FAM186A	
12	50314103	0.98	0.99	rs11169373	G	A	0.28	0.33		LNG, FAT, STRM, BRST, SKIN, GI, MUS, CRVX, BONE			Pitx3	12kb 3' of FAM186A	
12	50314316	0.98	0.99	rs11169374	C	T	0.28	0.33		LNG, FAT, STRM, SKIN, GI, MUS, CRVX, BONE			Myf	12kb 3' of FAM186A	
12	50314613	0.89	0.99	rs7301186	C	T	0.25	0.31		LNG, FAT, STRM, SKIN, GI, MUS, CRVX, BONE			BDP1,Maf,NF-E2	12kb 3' of FAM186A	
12	50314622	0.88	0.99	rs7301189	C	G	0.25	0.31		LNG, FAT, STRM, SKIN, GI, MUS, CRVX, BONE			BDP1,Osr	12kb 3' of FAM186A	

12	50314920	0.98	0.99	rs10615610	ACT	A	0.28	0.33		LNG, FAT, STRM, MUS, SKIN, LIV, GI, OVRY, CRVX, BONE	ESC,ESDR,ESDR,ESDR,ESDR,ESC,LNG,BRST,BLD,BLD,BLD,SKIN,SKIN,SKIN,SKIN,BRN,HRT,GI,KID,LNG,MUS,MUS,THYM,OVRY,BLD,CRVX,LIV,MUS,MUS,VAS,BLD,BRN,SKIN,LNG	CTCF,RAD21,SMC3,JUND	Gfi1,NF-AT	11kb 3' of FAM186A	
12	50315087	0.91	0.98	rs35663729	C	T	0.08	0.32		ESDR, LNG, IPSC, FAT, STRM, MUS, SKIN, LIV, GI, OVRY, CRVX, BONE	ESC,ESC,SKIN,SKIN,LNG,THYM,LNG,CRVX		YY1	11kb 3' of FAM186A	
12	50315753	0.96	0.98	rs34849043	T	C	0.28	0.33		LNG, FAT, MUS, SKIN, GI, CRVX			Foxa,Sox	10kb 3' of FAM186A	
12	50316546	0.94	0.97	rs6580739	T	C	0.27	0.33		SKIN			AP-1	9.7kb 3' of FAM186A	
12	50318275	0.89	-0.98	rs10876018	G	A	0.35	0.65		LIV			GR,Zbtb3	8kb 3' of FAM186A	
12	50318297	0.95	0.98	rs11169375	T	C	0.28	0.34		LIV			BCL,ERalpha-a,Ets,Irf,SP1,ZBRK1	7.9kb 3' of FAM186A	
12	50319308	0.95	0.98	rs11169376	G	C	0.27	0.33	ESC		IPSC		ERalpha-a,Esr2	6.9kb 3' of FAM186A	
12	50319311	0.95	0.98	rs11169377	G	A	0.27	0.33	ESC		IPSC		Evi-1,NR4A,SREBP	6.9kb 3' of FAM186A	
12	50319514	0.9	-0.99	rs6580740	G	A	0.35	0.65	ESC				Foxp1,HDAC2,Sox	6.7kb 3' of FAM186A	
12	50320462	0.95	0.98	rs34614542	A	T	0.28	0.33					Arid5b,HMG-IY,Pou	5.8kb 3' of	

													2f2,STAT	FAM186A	
12	50322669	0.89	-0.98	rs10783350	A	G	0.35	0.65	ESC, PLCNT, GI	ESDR, LNG, SKIN, LIV, GI, CRVX			ZBRK1,ZBTB7A	3.6kb 3' of FAM186A	
12	50323175	0.89	-0.98	rs7399343	C	A	0.35	0.65	PLCNT	LIV, GI			DMRT5	3.1kb 3' of FAM186A	
12	50323797	0.95	0.98	rs11836169	T	C	0.28	0.34	PLCNT	LIV, GI			BDP1,Ets,SP1,Znf14 3	2.4kb 3' of FAM186A	
12	50324077	0.95	0.98	rs34858415	T	C	0.28	0.33		LIV, GI			AIRE,IRC900814	2.2kb 3' of FAM186A	
12	50325598	0.89	-0.98	rs11169379	G	A	0.34	0.65	GI	BRST, SKIN, FAT, LIV, BRN, GI, OVRY, LNG, CRVX				631bp 3' of FAM186A	
12	50328538	0.94	0.98	rs66895907	GTC	G	0.27	0.33		FAT, GI, MUS			Mef2,Pax-2	FAM186A	intronic
12	50329782	0.94	0.98	rs34039674	G	A	0.28	0.33		ESC, FAT, STRM, SKIN, CRVX, BONE			Mef2,ZEB1	FAM186A	intronic
12	50329843	0.94	0.97	rs35878271	G	T	0.28	0.33		ESC, FAT, STRM, SKIN, CRVX, BONE			ELF1,Myc,NRSF,YY1, Zfx	FAM186A	intronic
12	50330210	0.89	-0.98	rs7489214	A	G	0.34	0.65		ESC, FAT, STRM, BRST, SKIN, GI, LNG, CRVX, BONE			GATA,HMGN3,Maf, NF-E2,Nrf-2	FAM186A	intronic
12	50332182	0.95	0.98	rs7310541	T	C	0.28	0.34		ESC, IPSC, FAT, STRM, SKIN, MUS	BLD		Nanog,Pou5f1,Sox	FAM186A	intronic
12	50333923	0.94	0.98	rs6580741	G	C	0.28	0.33	STRM	FAT, STRM, MUS, BONE	GI,CRVX		VDR,XBP-1	FAM186A	missense
12	50336460	0.95	0.98	rs7134337	C	T	0.28	0.33		SKIN, GI			CCNT2,Klf4,Klf7,SP1	FAM186A	intronic
12	50336675	0.95	0.98	rs7134595	C	T	0.28	0.33		GI	IPSC,BRST,SKIN,BLD		AP-1,BCL,DMRT2,Irf ,Pax-5,RXRA,STAT,p 300	FAM186A	intronic
12	50337340	0.85	-0.97	rs11169385	G	A	0.34	0.64		ADRL			Hoxa7,Sox	FAM186A	intronic
12	50340416	0.95	-0.98	rs4768900	T	A	0.72	0.66	LNG	ESDR, BLD			Foxj2,HNF1,Hmx,M	FAM186A	intronic

													ef2,Ncx,Pou5f1,TATA		
12	50342506	0.94	0.97	rs12830155	G	T	0.28	0.33					Pax-5	FAM186A	intronic
12	50343015	0.88	-0.98	rs10876021	C	T	0.34	0.65	ESC				Ik-2,Pax-6,Pou5f1	FAM186A	intronic
12	50343764	0.88	-0.98	rs11169386	G	T	0.34	0.65					E2F,Ets,Myc,ZBTB33,Znf143,p300	FAM186A	intronic
12	50344819	0.93	-0.97	rs4768949	A	G	0.72	0.66	GI	GI			DMRT4,Dbx1,Foxp1,HMG-IY,HNF1,Hmx,Hoxd8,Irf,Ncx,Nkx6-1,Pax-4,Pax-6,Sox,Zfp105,p300	FAM186A	intronic
12	50345225	0.88	-0.98	rs4768951	C	T	0.34	0.65		ESC			Hoxa5,RORalpha1	FAM186A	intronic
12	50345422	0.94	-0.97	rs4768872	C	A,T	0.72	0.66		ESC				FAM186A	intronic
12	50348719	0.81	0.9	rs36017775	C	T	0.27	0.33	IPSC	BRST, SKIN, LNG, MUS			TCF12	FAM186A	intronic
12	50350130	0.8	-0.9	rs7295847	C	T	0.72	0.66	SKIN	BRST, SKIN				FAM186A	intronic
12	50350336	0.8	-0.9	rs7296291	G	A	0.72	0.66	SKIN	BRST				FAM186A	missense
12	50350388	0.8	-0.9	rs7312252	T	C	0.72	0.66	SKIN	BRST			AIRE,HNF4,Pax-3,RXRA	FAM186A	synonymous
12	50350970	0.8	-0.9	rs10506292	T	C	0.72	0.66	SKIN	SKIN, FAT, OVRY, HRT, MUS, GI, SPLN, BLD			Evi-1,Pax-4,RFX5	FAM186A	synonymous
16	284890	0.85	0.93	rs419949	G	A	0.00	0.18	ESDR, IPSC, SKIN, PANC, GI, SPLN	ESDR, IPSC, SKIN, BRN, GI, PANC, HRT, SPLN	PANC		PU.1	PDIA2	missense
16	286396	1	1	rs400037	G	A	0.05	0.18	SKIN, PANC	ESDR, SKIN, BRN, GI, PANC	IPSC,PANC			PDIA2	missense
16	287017	0.97	0.98	rs449401	G	A	0.04	0.18	ESDR, SKIN, GI, PANC	ESDR, IPSC, ESC, SKIN, BRN, GI, PANC, HRT, LNG			AP-1,BCL,BHLHE40,CAC-binding-protein,CCNT2,CHD2,E2F,	PDIA2	intronic

													ELF1,Egr-1,Ets,HEY1,INSM1,Irf,Klf4,Klf7,MOVO-B,Myc,NF-E2,NRSF,Nrf1,PU.1,Pou2f2,SP1,STAT,TATA,TR4,UF1H3BETA,ZBTB33,ZNF219,Znf143		
16	287678	0.82	0.98	rs393521	T	G	0.08	0.21	ESDR, SKIN, GI, PANC, SPLN	IPSC, ESC, SKIN, GI, PANC, HRT, LNG		CTCF	Zec	AXIN1	3'-UTR
16	287871	0.85	1	rs394128	T	C	0.08	0.21	ESDR, SKIN, GI, PANC, SPLN	ESDR, IPSC, SKIN, GI, PANC, HRT, LNG	LIV		DEC,NF-E2,YY1	AXIN1	3'-UTR
16	289415	0.97	1	rs387467	G	T	0.06	0.19	ESDR, SKIN, GI, SPLN	SKIN, MUS, PANC, HRT			AP-1,BDP1,CHD2,CTCF,Egr-1,Hic1,MAZ,MAZR,NF-kappaB,PU.1,RXRA,SP1,UF1H3BETA,ZBTB7A,ZNF263,Zfp281,p300	AXIN1	intronic
16	291885	0.94	0.99	rs2858002	C	T	0.04	0.19	ESDR, IPSC, SKIN, GI, SPLN	ESDR, IPSC, BLD, SKIN, PANC, SPLN				AXIN1	intronic
16	295230	0.95	1	rs59260763	G	A	0.04	0.19		SKIN			AP-1,LUN-1	AXIN1	intronic
16	297184	0.83	1	rs214252	A	G	0.07	0.21	ESDR, SKIN	ESDR, IPSC, SKIN, BRN, GI, SPLN	LNG			AXIN1	synonymous
16	298222	0.82	0.99	rs214250	C	T	0.07	0.21	ESDR, IPSC, SKIN	ESDR, IPSC, SKIN, GI	LNG		GR,Pbx-1	AXIN1	synonymous
16	302736	0.82	0.97	rs1204042	G	A	0.04	0.16	ESDR, SKIN, LNG, SPLN	ESDR, IPSC, BLD, SKIN			Pbx-1,TCF4	AXIN1	intronic
16	302992	0.8	0.97	rs169955	C	T	0.04	0.16	ESDR, SKIN, SPLN	ESDR, IPSC, BLD, SKIN			Egr-1,SETDB1	AXIN1	intronic
19	41350981	0.88	0.97	rs2241715	A	C	0.49	0.69	ESC, ESDR, LNG, IPSC, FAT, STRM, BRST, BLD, MUS,	ESC, ESDR, LNG, IPSC, FAT, STRM, BRST, BLD, MUS, BRN, SKIN, LIV, GI, ADRL, HRT, PLCNT, THYM, OVRY, PANC, SPLN,	ESDR,ESDR,ESDR,IPSC,BRST,BLD,BLD,BLD,	GATA2,CMYC,MAX,MXI1,NRSF,POL2,P	NRSF,Nrf1,RFX5,Sin3Ak-20	TGFB1	intronic

									BRN, SKIN, VAS, LIV, GI, ADRL, HRT, KID, PANC, PLCNT, THYM, OVRY, SPLN, CRVX, BONE	CRVX, VAS, BONE	BLD,BLD,BLD,SKIN,SKIN,SKIN,KID,PANC,BLD,BLD,SKIN	OL24H8,PU1,TAF1,TAL1,ZNF263			
19	41351499	0.88	0.97	rs12462166	T	C	0.49	0.69	ESC, ESDR, LNG, IPSC, FAT, STRM, BRST, BLD, MUS, BRN, SKIN, VAS, LIV, GI, ADRL, HRT, KID, PANC, PLCNT, THYM, OVRY, SPLN, CRVX, BONE	ESC, ESDR, LNG, IPSC, FAT, STRM, BRST, BLD, MUS, BRN, SKIN, LIV, GI, ADRL, HRT, PLCNT, THYM, OVRY, PANC, SPLN, CRVX, BONE	ESC,ESDR,ESDR,ESDR,LNG,IPSC,IPSC,BRST,BLD,BLD,BLD,BLD,BLD,BLD,BLD,SKIN,SKIN,SKIN,HRT,GI,KID,LNG,MUS,MUS,PLCNT,GI,THYM,OVRY,PANC,MUS,GI,BLD,LIV,BRST,MUS,VAS,BLD,BLD,BRN,SKIN,SKIN,LNG	NFKB,EGR1,IRF4,PU1,SP1,POL24H8,AP2ALPHA,AP2GAMMA,CCNT2,CMYC,HMGN3,POL2,SIN3AK20,ZBTB7A,HAE2F1	AP-1,AP-2,ATF3,BCL,BHLHE40,CCNT2,CTCF,E2F,ELF1,Egr-1,Ets,HEY1,Irf,Klf7,MAZ,MOVO-B,Myc,Nrf1,Pou2f2,Rad21,SP1,SRF,Sp4,TATA,UF1H3BETA,YY1,Zfp281,Zic,Znf143,p300	TGFB1	intronic
19	41351534	0.85	0.95	rs12983775	G	A	0.48	0.69	ESC, ESDR, LNG, IPSC, FAT, STRM, BRST, BLD, MUS, BRN, SKIN, VAS, LIV, GI, ADRL, HRT, KID, PANC, PLCNT, THYM, OVRY, SPLN, CRVX, BONE	ESC, ESDR, LNG, IPSC, FAT, STRM, BRST, BLD, MUS, BRN, SKIN, LIV, GI, ADRL, HRT, PLCNT, THYM, OVRY, PANC, SPLN, CRVX, BONE	ESC,ESDR,ESDR,ESDR,LNG,IPSC,IPSC,BRST,BLD,BLD,BLD,BLD,BLD,BLD,BLD,SKIN,SKIN,SKIN,HRT,KID,LNG,MUS,MUS,PLCNT,GI,THYM,OVRY,PANC,MUS,GI,BLD,LIV,BRST,MUS,VAS,BLD,BLD,BRN,SKIN,SKIN	NFKB,EBF1,EGR1,IRF4,NRF1,PU1,SP1,POL2,POL24H8,AP2ALPHA,AP2GAMMA,CCNT2,CMYC,HMGN3,SIN3AK20,ZBTB7A,HAE2F1	EWSR1-FLI1,Ets,MAZ,MAZR,MZF1::1-4,PU.1,SP1,STAT,ZNF263,Zfp281	TGFB1	intronic
19	41354391	0.91	0.99	rs1800469	A	G	0.49	0.69	ESC, ESDR, LNG, IPSC, FAT, STRM, BRST, BLD, MUS,	ESC, ESDR, LNG, IPSC, FAT, BRST, BLD, STRM, MUS, BRN, SKIN, LIV, GI, ADRL, HRT, KID, PLCNT, THYM, PANC, SPLN,	BRST,BLD,BLD,BLD,BLD,BLD,BLD,GI,THYM	CTCF,POL2	HNF4,Nkx2	TMEM91	

									BRN, SKIN, VAS, GI, ADRL, HRT, KID, PANC, PLCNT, THYM, OVRY, SPLN, CRVX, LIV, BONE	CRVX	,OVRY,LIV				
19	41355432	0.84	0.93	rs11466313	C	CCC T	0.45	0.68	ESDR, ESC, BLD, STRM, SKIN, BRN, GI, ADRL, LNG, PANC, PLCNT, MUS, SPLN, BONE	ESC, ESDR, IPSC, BRST, BLD, STRM, BRN, SKIN, FAT, LIV, GI, ADRL, MUS, PLCNT, THYM, HRT, LNG, PANC, SPLN	BLD,BLD,SKIN		EWSR1-FLI1,STAT,S pz1	TMEM91	intronic
19	41355769	0.98	0.99	rs2317130	C	T	0.49	0.68	ESDR, ESC, BLD, SKIN, BRN, GI, ADRL, LNG, PANC, PLCNT, MUS, SPLN	ESC, ESDR, IPSC, BRST, BLD, STRM, BRN, SKIN, FAT, LIV, GI, ADRL, MUS, PLCNT, THYM, HRT, LNG, PANC, SPLN	BLD,BLD,BLD		GATA,TAL1	TMEM91	intronic
19	41357872	0.81	0.92	rs11668109	A	C	0.16	0.67	BLD, SKIN, BRN, GI	ESC, ESDR, IPSC, BLD, SKIN, FAT, LIV, BRN, GI, MUS, PLCNT, THYM, HRT, LNG, PANC, SPLN	BLD,BLD,BLD,PLCNT, BLD		BCL,BDP1,Brachyur y,E2F,GR,Rad21,STA T	TMEM91	intronic
19	41358604	0.92	0.99	rs1982072	T	A	0.48	0.69	BLD	ESDR, BLD, SKIN, FAT, BRN, GI, MUS, PLCNT, THYM, LNG, SPLN	BLD		Foxc1,Hoxa13	TMEM91	intronic
19	41359388	0.88	0.95	rs4803458	A	G	0.46	0.68	SKIN	BLD, SKIN, GI, MUS				TMEM91	intronic
19	41363487	1	1	rs2241714	T	C	0.48	0.68	ESC, ESDR, LNG, IPSC, FAT, STRM, BRST, BLD, MUS, BRN, SKIN, LIV, GI, ADRL, HRT, KID, PANC, PLCNT, THYM, OVRY, SPLN, CRVX, VAS, BONE	ESC, ESDR, LNG, IPSC, FAT, STRM, BRST, BLD, MUS, BRN, SKIN, LIV, GI, ADRL, HRT, PLCNT, THYM, PANC, SPLN, CRVX, VAS, BONE	BLD,BLD,BLD,BLD,BL D,HRT,KID,MUS,OVR Y,MUS,BLD		ZBRK1	TMEM91	missense
19	41363582	0.99	1	rs3217387	A	AA, AC	0.48	0.67	ESC, ESDR, LNG, IPSC, FAT, STRM, BRST, BLD, MUS, BRN, SKIN, VAS, LIV, GI,	ESC, ESDR, LNG, IPSC, FAT, STRM, BRST, BLD, MUS, BRN, SKIN, LIV, GI, ADRL, HRT, PLCNT, THYM, PANC, SPLN, CRVX, VAS, BONE	ESC,BLD,BLD,BLD,SKI N,SKIN,MUS,MUS,GI, OVRY,BLD,BRST			TMEM91	intronic

									ADRL, HRT, KID, PANC, PLCNT, THYM, OVRY, SPLN, CRVX, BONE						
19	41363851	1	1	rs2241712	C	T	0.48	0.68	ESC, ESDR, LNG, IPSC, FAT, STRM, BRST, BLD, MUS, BRN, SKIN, VAS, LIV, GI, ADRL, HRT, KID, PANC, PLCNT, THYM, OVRY, SPLN, CRVX, BONE	ESDR, IPSC, ESC, FAT, BRST, BLD, STRM, MUS, BRN, SKIN, LIV, GI, ADRL, HRT, PLCNT, THYM, LNG, SPLN, CRVX, VAS, BONE	ESC,ESDR,ESDR,ESDR,ESDR,ESC,LNG,IPSC,IPSC,BRST,BLD,BLD,BLD,BLD,BLD,BLD,BLD,SKIN,SKIN,SKIN,SKIN,ADRL,BRN,BRN,HRT,GI,GI,KID,LNG,MUS,MUS,PLCNT,GI,THYM,GI,OVRY,PANC,MUS,GI,LNG,BLD,CRVX,LIV,BRST,MUS,MUS,VAS,BLD,BLD,BRN,SKIN,SKIN,LNG	POL2,USF1,SP1,NFKB,PU1,NRF1,EGR1,IRF1,MAX,TBP,ZBTB7A	ATF3,ATF6,AhR::Arnt,AhR,Arnt,NF-E2	TMEM91	intronic
19	41365237	0.98	0.99	rs1549933	A	G	0.48	0.68	ESC, ESDR, IPSC, FAT, STRM, BRST, BLD, BRN, SKIN, VAS, LIV, GI, KID, LNG, HRT, OVRY, PLCNT, MUS, SPLN, CRVX	ESC, ESDR, LNG, IPSC, FAT, STRM, BRST, BLD, MUS, BRN, SKIN, VAS, LIV, GI, ADRL, HRT, KID, PANC, PLCNT, THYM, SPLN, CRVX, BONE	IPSC,BLD,OVRY,GI,LNG		AP-1	TMEM91	intronic
19	41367096	0.97	0.99	rs9797885	A	G	0.48	0.68	FAT, STRM, BLD, SKIN, LNG, CRVX, BONE	ESDR, LNG, FAT, STRM, BRST, BLD, MUS, SKIN, LIV, BRN, GI, ADRL, PANC, PLCNT, THYM, HRT, SPLN, CRVX, VAS, BONE			ATF3,E2F,Egr-1,HNF4,SREBP	TMEM91	intronic
19	41370563	0.86	0.94	rs4803459	T	C	0.46	0.67		FAT, BRST, BLD, SKIN			CTCF,Hic1,Pax-4,Rad21	TMEM91	intronic
10	28804117	1	1	rs2797472	T	C	0.57	0.68	LNG	LNG			Arid5a,Arid5b,Pou2	2.8kb 5' of	

													f2,Sox	RP11-478 H13.3	
10	28805679	1	1	rs1761985	C	T	0.62	0.68	LIV, LNG, BLD	ESDR, BLD, VAS, LIV, BRN, GI, ADRL, KID, LNG, HRT, OVRY, MUS	KID	CEBPB		1.3kb 5' of RP11-478 H13.3	
10	28805805	1	1	rs1775912	G	C	0.62	0.68	LIV, LNG, BLD	ESDR, IPSC, ESC, BLD, VAS, LIV, BRN, GI, ADRL, KID, LNG, HRT, OVRY, SPLN, MUS			BAF155,E2A,ERalpha-a,Hic1,Lmo2-complex,SIX5,ZEB1,Znf143	1.1kb 5' of RP11-478 H13.3	
10	28806070	1	1	rs1761986	G	A	0.62	0.68	LIV, LNG, GI, BLD	ESDR, ESC, LNG, IPSC, BLD, STRM, VAS, LIV, BRN, GI, ADRL, HRT, KID, PLCNT, OVRY, MUS, SPLN	BLD		Myc	876bp 5' of RP11-478 H13.3	
10	28810127	0.93	0.96	rs1761989	G	T	0.62	0.68	ESC, ESDR, IPSC, BLD	ESC, ESDR, LNG, IPSC, STRM, BLD, MUS, SKIN, BRN, GI, ADRL, KID, PLCNT, CRVX, LIV	ESC,ESDR,SKIN,BLD		DMRT3,Evi-1,Foxl1,Foxo,Foxp1,GATA,HNF1,Mef2,Pou6f1,Sox,TATA	1.9kb 3' of RP11-478 H13.3	
10	28815171	0.94	0.99	rs17564455	T	G	0.58	0.67		ESC			CTCF,Hsf	7kb 3' of RP11-478 H13.3	
10	28816789	0.89	0.95	rs2797469	G	A	0.62	0.68		GI			CDP	8.6kb 3' of RP11-478 H13.3	
10	28819126	0.84	0.94	rs1691921	A	T	0.58	0.70		ESDR, STRM, SKIN, KID, LNG, MUS, GI, BLD	SKIN,BLD		FAC1	11kb 3' of RP11-478 H13.3	

10	28819280	0.88	0.94	rs1691920	C	G	0.62	0.68		ESDR, STRM, SKIN, KID, LNG, MUS, GI, BLD	LNG			11kb 3' of RP11-478 H13.3	
10	28820369	0.84	0.95	rs1691919	G	C	0.62	0.70		ESDR, ESC, LNG, MUS, BLD	IPSC,IPSC		CAC-binding-protein,CACD,CCNT2,Egr-1,Klf4,Klf7,MAZR,SP1,UF1H3BETA,Zfp281	12kb 3' of RP11-478 H13.3	
10	28821387	0.8	0.95	rs787349	T	C	0.62	0.71	BLD	ESDR, LNG, OVRY, SPLN, BLD	BLD			13kb 3' of RP11-478 H13.3	
10	28823770	0.81	0.93	rs787346	C	T	0.62	0.70	GI, LNG, OVRY, BLD	ESC, ESDR, IPSC, STRM, BLD, SKIN, GI, ADRL, HRT, LNG, OVRY	ESDR,IPSC,HRT,LNG,OVRY,BLD,BRN		Myc,Smad	16kb 3' of RP11-478 H13.3	
9	134060541	1	1	rs11789898	G	T	0.11	0.20	ESDR, BLD, SKIN, FAT, BRN, GI, MUS, SPLN, CRVX, BONE	ESC, ESDR, LNG, IPSC, FAT, STRM, BRST, BLD, MUS, BRN, SKIN, LIV, GI, ADRL, HRT, PLCNT, THYM, OVRY, PANC, SPLN, CRVX, BONE	ESC,BLD,SKIN,SKIN,SKIN,ADRL,LNG,THYM,MUS,MUS,BLD,SKIN		TCF4	BRD3	intronic
9	134061669	1	1	rs72766630	G	T	0.09	0.20	ESDR, ESC, BLD, SKIN, BRN, GI, MUS, SPLN, CRVX	ESC, ESDR, LNG, IPSC, FAT, BRST, BLD, STRM, MUS, BRN, SKIN, LIV, GI, ADRL, HRT, PLCNT, THYM, OVRY, PANC, SPLN, CRVX			AP-4,LXR,PPAR	BRD3	intronic
9	134064464	1	1	rs55924785	C	T	0.09	0.20	ESDR, ESC, IPSC, BLD, STRM, BRN, SKIN, GI, PLCNT, MUS, SPLN, CRVX	ESC, ESDR, LNG, IPSC, FAT, BRST, BLD, MUS, BRN, SKIN, LIV, GI, ADRL, HRT, PLCNT, THYM, PANC, SPLN, CRVX			CTCF,GR,HNF4,RXRA,Rad21,SMC3,SP1	BRD3	intronic
9	134065012	1	1	rs11795079	T	C	0.09	0.20	ESC, ESDR, IPSC, FAT, BLD, STRM, MUS, BRN, SKIN, LIV, GI, KID, LNG, THYM, PLCNT,	ESC, ESDR, LNG, IPSC, FAT, STRM, BRST, BLD, MUS, BRN, SKIN, LIV, GI, ADRL, HRT, PLCNT, THYM, OVRY, PANC, SPLN, CRVX, VAS	ESC,IPSC,BLD,BLD,BLD,ADRL,PLCNT,BLD,LIV,BLD		BHLHE40,E2F,Ets,SRF,STAT	BRD3	intronic

									SPLN, CRVX					
9	134066656	1	1	rs72766638	C	A	0.09	0.20	ESC, ESDR, LNG, IPSC, FAT, STRM, BRST, BLD, MUS, BRN, SKIN, VAS, LIV, GI, ADRL, HRT, KID, PANC, PLCNT, THYM, OVRY, SPLN, CRVX, BONE	ESDR, LNG, IPSC, FAT, ESC, STRM, BRST, BLD, MUS, BRN, SKIN, LIV, GI, ADRL, HRT, PLCNT, THYM, OVRY, PANC, SPLN, CRVX, VAS, BONE	ESDR,ESDR,BRST,BLD,BLD,BLD,SKIN,SKIN,SKIN,SKIN,BRN,HRT,GI,LNG,MUS,MUS,GI,THYM,OVRY,GI,MUS,MUS,BLD,BLD,BRN,LNG	EGR1,GATA1	Irf	BRD3	intronic
9	134069081	0.97	0.99	rs146671954	G	A	0.09	0.20	ESC, ESDR, LNG, IPSC, FAT, STRM, BRST, BLD, MUS, BRN, SKIN, LIV, GI, ADRL, KID, PANC, PLCNT, THYM, HRT, SPLN, CRVX, VAS, BONE	ESDR, ESC, IPSC, FAT, BRST, BLD, STRM, MUS, BRN, SKIN, LIV, GI, ADRL, LNG, PLCNT, THYM, PANC, HRT, SPLN, CRVX, VAS	ESDR,BLD,BLD,PLCNT,GI		Irf,MIZF,NRSF,Znf143	545bp 5' of BRD3	
9	134071254	0.98	0.99	rs56303154	C	T	0.09	0.20	ESDR, IPSC, BLD, SKIN, GI, PLCNT	BLD, SKIN, MUS, HRT, SPLN, LIV	BLD		GR,Pax-5	2.7kb 5' of BRD3	
9	134084547	0.86	0.93	rs55931012	C	T	0.09	0.20		BRST, SKIN			ATF3,SP1	16kb 5' of BRD3	
9	134085797	0.85	0.93	rs35259020	TTC	T	0.08	0.20			CRVX		FAC1,GCNF,PRDM1,p53	17kb 5' of BRD3	
11	111283347	0.85	0.99	rs7130173	A	C	0.60	0.73	GI	FAT, BRST, BLD, SKIN, BRN, GI, LNG, MUS, THYM, HRT, LIV	ESC,ESDR,ESDR,ESC,IPSC,IPSC,BLD,BLD,SKIN,SKIN,SKIN,BRN,GI,GI,LNG,GI,THYM,BLD,LIV,MUS	CTCF,RAD21,SMC3,YY1	GLI,SP1,SRF	C11orf53	intronic
11	111286111	0.86	1	rs3087967	T	C	0.61	0.73	IPSC, GI, LIV	ESC, ESDR, SKIN, GI, LNG, MUS, LIV	GI,GI,LNG,LIV		Foxj1,Pax-5,RREB-1	C11orf53	

11	111290028	0.86	1	rs4477469	C	T	0.61	0.73		LIV			Pax-4	3.4kb 3' of C11orf92	
11	111291966	0.86	1	rs10789822	A	G	0.61	0.73		BRST, GI			Nrf1,PLAG1,TATA,Zfx	1.4kb 3' of C11orf92	
11	111294284	0.86	1	rs7103178	C	T	0.61	0.73		ESDR, BRST, BRN, GI, MUS, LIV				C11orf92	
11	111295779	1	1	rs12296076	G	A	0.61	0.70		ESDR, IPSC, BRST, GI, PANC, LIV			Cphx,NR4A,VDR	C11orf92	
11	111296832	1	1	rs6589218	A	C	0.61	0.70	ESC, GI, LNG, SPLN	ESC, IPSC, BRST, BLD, SKIN, BRN, GI, MUS, LNG, PANC, HRT, SPLN, LIV			ATF3,Egr-1,MAZR,ZBTB7A,Zfx	C11orf92	
11	111297051	0.99	1	rs7944895	C	G	0.61	0.70	ESC, IPSC, GI, LNG, PANC	ESC, IPSC, BRST, BLD, SKIN, BRN, GI, MUS, LNG, PANC, HRT, SPLN, LIV			BCL,CHD2,EBF,ERalpha-a,MAZ,UF1H3BETA,ZNF263	C11orf92	
11	111299815	0.83	0.98	rs10891246	A	G	0.61	0.73	ESC, ESDR, LNG, IPSC, FAT, STRM, BRST, BLD, MUS, BRN, SKIN, VAS, LIV, GI, ADRL, KID, PANC, PLCNT, THYM, HRT, OVRY, BONE	ESDR, LNG, IPSC, FAT, ESC, STRM, BRST, BLD, MUS, BRN, SKIN, LIV, GI, ADRL, HRT, PLCNT, THYM, PANC, SPLN, VAS	ESC,ESDR,ESDR,ESC,IPSC,IPSC,BLD,BLD,BLD,SKIN,BRN,GI,GI,KID,LNG,MUS,MUS,GI,GI,OVRY,PANC,MUS,GI,LIV,BRST		EWSR1-FLI1,Egr-1,NRSF,PU.1,SP1,SP2,STAT,ZBTB7A	C11orf93	intronic
11	111300019	0.98	0.99	rs7105857	C	T	0.61	0.70	ESC, ESDR, IPSC, FAT, STRM, BRST, BLD, MUS, BRN, SKIN, LIV, GI, ADRL, KID, PANC, LNG, THYM, HRT, OVRY, PLCNT	ESC, ESDR, LNG, IPSC, STRM, BRST, BLD, MUS, BRN, SKIN, FAT, LIV, GI, ADRL, HRT, PLCNT, THYM, PANC, SPLN, VAS	ESC,BLD,KID,LNG,MUS,GI,GI,OVRY,PANC,LIV		Lmo2-complex,Mxi1,Myf,SP1,Sin3Ak-20,TAL1,TATA,TCF12	C11orf93	intronic
11	111300346	0.83	0.98	rs7122375	T	C	0.61	0.73	ESC, ESDR, IPSC, FAT, BRST, BLD, MUS, SKIN, LIV, BRN, GI, PANC, LNG, PLCNT	ESC, ESDR, IPSC, BRST, BLD, BRN, SKIN, FAT, LIV, GI, LNG, MUS, PLCNT, THYM, PANC, HRT, SPLN	LNG,GI		NF-E2	C11orf93	intronic
11	111300921	0.81	0.97	rs3802840	T	G	0.61	0.73	ESDR, ESC, IPSC, BRST, BLD,	ESDR, ESC, BRST, BLD, SKIN, FAT, LIV, BRN, GI, LNG, MUS,			Homez,Nkx3	C11orf93	intronic

									LIV, BRN, GI, LNG, MUS, PLCNT, SKIN	THYM, PANC, HRT, SPLN					
11	111300947	0.81	0.97	rs3802841	G	T	0.61	0.73	ESDR, ESC, IPSC, BRST, BLD, LIV, BRN, GI, LNG, MUS, PLCNT, SKIN	ESDR, ESC, BRST, BLD, SKIN, FAT, LIV, BRN, GI, PANC, LNG, MUS, THYM, HRT, SPLN			Nanog	C11orf93	intronic
11	111300984	0.81	0.97	rs3802842	C	A	0.61	0.73	ESDR, ESC, IPSC, BRST, BLD, LIV, BRN, GI, LNG, MUS, PLCNT	ESDR, ESC, BRST, BLD, SKIN, FAT, LIV, BRN, GI, PANC, LNG, MUS, THYM, HRT, SPLN	BLD		Ik-1,Obox3,Pax-4,Zfp410	C11orf93	intronic
		0.81	0.97	rs111388405	T	C	0.61	0.73	ESDR, BRST, BLD, LIV, BRN, GI, LNG, MUS, PLCNT	ESDR, ESC, BRST, BLD, SKIN, FAT, LIV, BRN, GI, PANC, LNG, MUS, HRT	SKIN,BRN,GI,LNG,MUS,MUS,GI,OVRY,PANC,GI,MUS,MUS,BLD		UF1H3BETA	C11orf93	intronic
11	111301485	0.81	0.97	rs4520624	A	G	0.61	0.73	ESDR, BRST, BLD, LIV, BRN, GI, LNG, MUS, PLCNT	ESDR, ESC, BRST, BLD, SKIN, FAT, LIV, BRN, GI, PANC, LNG, MUS, HRT	SKIN,BRN,LNG,MUS,MUS,GI,PANC,MUS,MUS,BLD		Foxj2,Myb	C11orf93	intronic
11	111301678	0.81	0.97	rs10789823	T	C	0.60	0.73	BRST, BLD, LIV, BRN, GI, LNG, PLCNT	ESDR, ESC, BRST, BLD, SKIN, FAT, LIV, BRN, GI, PANC, LNG, MUS, HRT	MUS		PU.1,SPIB,p300	C11orf93	intronic
1	37949835	0.8	-0.9	rs17531077	A	G	0.35	0.59	SKIN, BRN, GI, SPLN, LIV	ESC, ESDR, IPSC, FAT, BRST, BLD, SKIN, GI, MUS, PLCNT, THYM, HRT, SPLN, LNG, CRVX, LIV		NRSF	BDP1,Egr-1,Irf,PU.1,RXRA,TATA	2.8kb 5' of INPP5B	
1	37968634	0.85	0.93	rs28428561	G	A	0.65	0.41	IPSC, ESC	ESC, IPSC, FAT, STRM, MUS, BONE				SF3A3	intronic
1	37979077	0.85	0.92	rs7555461	T	C	0.65	0.41		LIV			Arid5a,Arid5b,Gfi1,HDAC2,Pax-4,Pou2f2,SIX5,Sox,p300	SF3A3	intronic
1	37979982	0.86	0.93	rs10890238	T	A	0.65	0.41		LIV			Foxl1,Ik-2	SF3A3	intronic
1	37986909	0.92	0.96	rs12138115	A	G	0.65	0.41	SKIN, BRN, PLCNT, BLD	BLD, BRN, GI, MUS, CRVX			Dmbx1,ERalpha-a,Evi-1	SF3A3	intronic
1	37990219	0.85	-0.99	rs4360494	G	C	0.35	0.55	ESC, ESDR, LNG, IPSC, FAT,	ESDR, ESC, STRM, BRST, BLD, BRN, SKIN, FAT, LIV, GI, MUS,	ESC,ESDR,ESDR,ESDR	CTCF,POL2,USF1,C	CTCFL,CTCF,RXRA,R	SF3A3	

									STRM, BRST, BLD, MUS, BRN, SKIN, VAS, LIV, GI, ADRL, HRT, KID, PANC, PLCNT, THYM, OVRY, SPLN, CRVX, BONE	THYM, PANC, PLCNT, HRT, CRVX	,ESDR,ESC,LNG,IPSC,IPSC,BRST,BLD,BLD,BLD,BLD,BLD,SKIN,SKIN,SKIN,SKIN,ADRL,BRN,BRN,HRT,GI,GI,KID,LNG,MUS,MUS,PLCNT,GI,THYM,GI,OVRY,PANC,MUS,GI,LNG,BLD,CRVX,LIV,BRST,MUS,MUS,VAS,BLD,BLD,BRN,SKIN,SKIN,LNG	HD2,EBF1,ELF1,GABP,P300,RAD21,RFX5,SMC3,SP1,SRF,TBP,WHIP,NFKB,ATF3,CTBP2,RXRA,YY1,TCF4,AP2ALPHA,AP2GAMMA,BRCA1,GTF2F1,JUND,MAX,MXI1,FOXA1,HDAC2,TCF12,TR4,CCNT2,CMYC,CTCFL,E2F6,HMGN3,IRF1,JUNB,MAFK,NRSF,SP2,STAT1,STAT2,CTC	ad21,SMC3		
1	37990434	1	1	rs4072980	G	A	0.65	0.41	ESC, ESDR, LNG, IPSC, FAT, STRM, BRST, BLD, MUS, BRN, SKIN, VAS, LIV, GI, ADRL, HRT, KID, PANC, PLCNT, THYM, OVRY, SPLN, CRVX, BONE	ESDR, FAT, ESC, STRM, BRST, BLD, MUS, BRN, SKIN, LIV, GI, ADRL, THYM, PANC, PLCNT, HRT, CRVX	ESDR,ESDR,ESC,BRST,BLD,BLD,BLD,SKIN,SKIN,SKIN,SKIN,BRN,HRT,LNG,MUS,MUS,PLCNT,GI,MUS,GI,BLD,CRVX,LIV,BRST,MUS,VAS,BLD,BRN,SKIN	POL2,ELK4,STAT1,HAE2F1,CTCF	AIRE,STAT	SF3A3	
1	37995647	0.82	-0.98	rs61776719	C	A	0.35	0.56	ESC, ESDR, LNG, IPSC, FAT, STRM, BRST, BLD, BRN, SKIN, VAS, LIV, GI, ADRL, KID, MUS, THYM, HRT, PANC, PLCNT, SPLN, CRVX	ESC, ESDR, LNG, IPSC, FAT, STRM, BRST, BLD, BRN, SKIN, LIV, GI, ADRL, HRT, KID, MUS, PLCNT, THYM, PANC, SPLN, CRVX, VAS, BONE	LNG,BLD,BLD,SKIN,GI,KID,LNG,MUS,GI,PANC,CRVX,BLD,SKIN,SKIN			1.1kb 3' of FHL3	

1	37996149	0.81	-0.97	rs67631072	C	T	0.36	0.56	ESC, ESDR, LNG, IPSC, FAT, STRM, BRST, BLD, BRN, SKIN, VAS, LIV, GI, ADRL, HRT, KID, PANC, MUS, PLCNT, THYM, OVRY, SPLN, CRVX, BONE	ESC, ESDR, LNG, IPSC, FAT, STRM, BRST, BLD, MUS, BRN, SKIN, LIV, GI, ADRL, HRT, PLCNT, THYM, PANC, SPLN, CRVX, VAS, BONE	ESC,ESDR,ESDR,ESDR,ESDR,ESC,LNG,IPSC,IPSC,BLD,BLD,BLD,BLD,SKIN,SKIN,SKIN,ADRL,BRN,BRN,HRT,GI,GI,KID,LNG,MUS,MUS,PLCNT,GI,THYM,GI,OVRY,PANC,MUS,GI,CRVX,BRST,MUS,BRN,SKIN,SKIN,LNG	POL2,POL24H8,BCL3,NANOG,P300,SP1,TAF1,TBP,TCF12,E2F4,HAE2F1,POL2S2,STAT3,CCNT2	BCL,EBF,Ets,Myc,NF-kappaB,NRSF,Sin3Ak-20	620bp 3' of FHL3	
1	37997832	0.94	0.97	rs7366048	C	T	0.64	0.41	ESDR, IPSC, BRST, BLD, STRM, SKIN, BRN, GI, MUS, PLCNT, SPLN, CRVX	ESC, ESDR, LNG, IPSC, FAT, BRST, BLD, STRM, MUS, SKIN, LIV, BRN, GI, ADRL, HRT, KID, PLCNT, THYM, OVRY, PANC, SPLN, CRVX, VAS				FHL3	synonymous
22	39236915	0.99	0.99	rs5757572	C	G	0.87	0.62	BLD, STRM, BRN, SKIN, FAT, GI, HRT, MUS, SPLN, VAS	ESC, ESDR, IPSC, STRM, BRST, BLD, MUS, BRN, SKIN, FAT, LIV, GI, ADRL, HRT, LNG, PLCNT, THYM, OVRY, PANC, SPLN, CRVX, VAS	ESDR,BLD,SKIN,HRT,LNG,MUS,BLD,MUS,MUS,BLD	E2F6,MAX	CTCF,GR,Rad21	PDGFB	intronic
22	39237617	1	1	rs5757573	C	T	0.87	0.62	BLD, STRM, BRN, SKIN, FAT, GI, HRT, MUS, VAS	ESC, ESDR, IPSC, STRM, BRST, BLD, MUS, BRN, SKIN, FAT, LIV, GI, ADRL, HRT, LNG, PLCNT, THYM, OVRY, PANC, SPLN, CRVX, VAS	ESC,ESDR,ESDR,BLD,BLD,BLD,BLD,BLD,SKIN,BRN,BRN,HRT,GI,LNG,MUS,MUS,PLCNT,GI,GI,BLD,LIV,MUS,BLD,SKIN,SKIN	NRSF	Irf,SP1	PDGFB	intronic
22	39244698	0.8	0.96	rs1800818	C	T	0.86	0.59	ESC, ESDR, LNG, IPSC, FAT, STRM, BRST, BLD, MUS, BRN, SKIN, VAS, LIV, GI, ADRL, KID, PANC, PLCNT,	ESC, ESDR, LNG, IPSC, FAT, STRM, BRST, BLD, MUS, BRN, SKIN, LIV, GI, ADRL, HRT, PLCNT, THYM, OVRY, PANC, SPLN, CRVX, BONE	ESDR,ESDR,IPSC,BRST,SKIN,SKIN,SKIN,ADRL,BRN,KID,LNG,MUS,MUS,PLCNT,GI,GI,	HAE2F1,E2F6	AP-1,BCL,CHD2,E2F,HDAC2,HEY1,Hic1,Myc,Nrf1,SRF,YY1	PDGFB	5'-UTR

									THYM, HRT, OVRY, SPLN, CRVX		OVRY,PANC,MUS,LNG,LIV,BRST,MUS,BRN				
22	39251085	0.93	-0.98	rs59744664	A	G	0.13	0.39	BLD, SKIN, MUS, SPLN	ESC, ESDR, LNG, IPSC, FAT, BRST, BLD, BRN, SKIN, GI, HRT, MUS, PLCNT, SPLN, LIV	ESC,ESDR,LNG,IPSC,IPSC,LNG		Cphx,NERF1a	6.3kb 5' of PDGFB	
22	39252092	0.93	-0.98	rs11705523	A	G	0.13	0.39	ESC, BLD, SKIN, MUS, GI, LNG, CRVX, LIV, BRN	ESC, ESDR, LNG, IPSC, FAT, STRM, BRST, BLD, MUS, SKIN, LIV, BRN, GI, HRT, PLCNT, PANC, SPLN, CRVX, VAS, BONE	IPSC,KID,OVRY,MUS,LIV	AP2ALPHA,AP2GAMMA	AP-2,BCL,ELF1,LXR,TR4	7.3kb 5' of PDGFB	
16	68740380	0.8	0.92	rs11865026	T	C	0.24	0.27	ESC, IPSC, BRST, SKIN, LIV, BRN, GI, PANC, PLCNT, LNG, BLD	ESC, ESDR, IPSC, BRST, BLD, STRM, SKIN, LIV, BRN, GI, KID, PANC, LNG, PLCNT, THYM, SPLN, CRVX	BRST,SKIN,SKIN,GI,GI,LNG,GI,GI,PANC,GI,LNG,BLD,LIV,BRST,MUS,BLD,SKIN	FOSL2,FOXA1,HNF4G,JUND,P300,RFX5,SP1,STAT3	Hsf	CDH1	intronic
16	68744554	0.8	0.93	rs7200690	C	T	0.24	0.27	ESC, IPSC, BRST, SKIN, LIV, GI, PANC, SPLN, LNG	ESC, ESDR, IPSC, BRST, BLD, STRM, SKIN, LIV, BRN, GI, KID, PANC, LNG, MUS, PLCNT, THYM, SPLN	ESDR,ESC,IPSC,SKIN,PLCNT,GI,GI,BRST,SKIN		EWSR1-FLI1	CDH1	intronic
16	68746166	0.9	0.98	rs12921546	G	A	0.24	0.27	IPSC, BRST, SKIN, GI, PANC, SPLN, LNG	ESC, ESDR, IPSC, BRST, SKIN, LIV, BRN, GI, PANC, LNG, PLCNT, THYM	LNG,IPSC,BLD,BLD,BLD,KID,GI,PANC,SKIN		ATF3,E2A,ZEB1	CDH1	intronic
16	68750700	0.9	0.98	rs9937664	T	C	0.24	0.27		ESC, IPSC, BRST, BLD, SKIN, LIV, BRN, GI, PANC, LNG, THYM, SPLN	SKIN		BCL,NRSF,SP1,Sin3Ak-20	CDH1	intronic
16	68755006	0.88	0.98	rs17772363	G	T	0.24	0.27	SKIN, GI	ESC, ESDR, IPSC, BRST, BLD, SKIN, LIV, BRN, GI, PANC, PLCNT, THYM, SPLN, LNG, CRVX, MUS	BRST,SKIN,GI,GI,CRVX,BRST,SKIN		AP-1,AP-4,CACD,E2A,Tgif1,ZEB1,Znf143	CDH1	intronic
16	68755542	0.9	0.98	rs35069703	G	A	0.24	0.27	SKIN, GI, BLD	ESC, ESDR, IPSC, BRST, BLD, SKIN, LIV, BRN, GI, PANC, PLCNT, THYM, SPLN, LNG, CRVX, MUS	IPSC,BRST,SKIN,LNG,PLCNT,GI,CRVX,BRST,SKIN	P300,RPC155,TFIIIC110,POL2	NRSF,Znf143,p53	CDH1	intronic
16	68756599	0.9	0.98	rs13333528	C	T	0.24	0.27	SKIN, GI, BLD	ESC, ESDR, IPSC, BRST, BLD, SKIN, LIV, BRN, GI, PANC, PLCNT, THYM, SPLN, LNG, CRVX, MUS	BRST,BLD,BLD,BLD,BLD,SKIN,GI,THYM,GI,PANC,GI,BLD,CRVX,BRST,MUS,MUS,BRN,S	BATF,BCL11A,BCL3,CHD2,EBF1,MEF2A,MEF2C,NFKB,OCT2,P300,PAX5N19,	Irf	CDH1	intronic

											KIN	PBX3,POU2F2,RXRA,SP1,STAT3,TCF12,CJUN,POL2,POL2B,GATA3			
16	68757328	0.88	0.97	rs9646283	A	G	0.24	0.27	BLD, SKIN, GI	ESC, ESDR, IPSC, BRST, BLD, SKIN, LIV, BRN, GI, PANC, PLCNT, THYM, LNG, SPLN, CRVX, MUS	BRST,SKIN,BLD,BRST,SKIN	NFKB	Sp4,Zfx	CDH1	intronic
16	68757403	0.89	0.95	rs9646284	C	G	0.24	0.28	BLD, SKIN, GI	ESC, ESDR, IPSC, BRST, BLD, SKIN, LIV, BRN, GI, PANC, PLCNT, THYM, SPLN, LNG, CRVX, MUS	BRST,SKIN,PLCNT,BLD,BRST,SKIN	EBF1,JUND,NFKB	BCL,Elf5,Ets,FEV,Hsf,Ncx,PU.1,STAT	CDH1	intronic
16	68757741	0.9	0.98	rs12446575	C	G	0.24	0.27	SKIN, GI, BLD	ESC, ESDR, IPSC, BRST, BLD, SKIN, BRN, GI, PANC, PLCNT, THYM, LNG, CRVX, MUS	BRST,BLD		EWSR1-FLI1,HDAC2,Nanog,Sox,TATA,p300	CDH1	intronic
16	68762843	0.84	0.95	rs35158985	A	G	0.24	0.30	ESC, IPSC, BRST, BRN, SPLN, MUS, SKIN	ESC, ESDR, LNG, IPSC, FAT, STRM, BRST, BLD, BRN, SKIN, GI, MUS, PLCNT, THYM, PANC, SPLN, CRVX, LIV, BONE	IPSC,BRST,SKIN		BCL,EBF,Irf,SETDB1,SP1,ZBRK1	CDH1	intronic
16	68763990	1	1	rs9928847	C	T	0.24	0.29	ESC, IPSC, BRN, THYM, MUS, SKIN	ESC, ESDR, IPSC, FAT, BRST, BLD, BRN, SKIN, GI, MUS, THYM, PANC, PLCNT, SPLN, CRVX	ESC,ESC,IPSC,SKIN,KID,THYM,SKIN		GATA,Spz1,UF1H3BETA,Zfp281	CDH1	intronic
16	68764779	1	1	rs9936621	A	G	0.24	0.29	ESC, IPSC, BLD, SKIN, BRN, MUS	ESC, ESDR, IPSC, FAT, BRST, BLD, BRN, SKIN, GI, MUS, THYM, PANC, PLCNT, SPLN, CRVX	IPSC,BRST,SKIN,BRN,THYM		AP-2,Ets,Pou2f2,RXRA,Rad21,SMC3,ZBTB7A	CDH1	intronic
16	68766041	1	1	rs4783674	T	C	0.24	0.29		ESC, IPSC, BRST, BLD, SKIN, BRN, MUS, THYM, PANC, PLCNT, GI, CRVX			AP-1,CTCF,ERalpha-a,RXRA	CDH1	intronic
16	68766132	0.99	1	rs4783675	G	C	0.24	0.28		ESC, IPSC, BRST, BLD, SKIN, BRN, MUS, THYM, PANC, PLCNT, GI, CRVX			GZF1	CDH1	intronic
16	68766549	1	1	rs13334471	T	C	0.24	0.29		ESC, IPSC, BRST, BLD, SKIN, BRN, MUS, THYM, GI, PANC	SKIN		Arid5b,Sox	CDH1	intronic
16	68766853	0.95	1	rs13330378	G	A	0.24	0.28		IPSC, BRST, SKIN, BRN, MUS, THYM, GI, PANC, BLD			BDP1,LUN-1	CDH1	intronic
16	68766935	0.97	0.99	rs28660361	T	C	0.23	0.28		IPSC, BRST, SKIN, BRN, MUS, THYM, GI, PANC, BLD	BRST,SKIN			CDH1	intronic
16	68766946	0.97	1	rs13330350	C	T	0.23	0.28		IPSC, BRST, SKIN, BRN, MUS, THYM, GI, PANC, BLD	BRST		GCNF,LRH1,Nr2f2,P	CDH1	intronic

												ax-6,SF1		
16	68767977	0.99	1	rs8056761	T	C	0.24	0.28		IPSC, ESC, BRST, BLD, SKIN, BRN, GI, MUS, THYM, PANC			Foxa	CDH1	intronic
16	68768135	0.97	1	rs28608872	A	G	0.23	0.28	LNG	IPSC, ESC, BRST, BLD, SKIN, BRN, GI, MUS, THYM, PANC	SKIN		Ets	CDH1	intronic
16	68768165	0.88	1	rs8055912	C	T	0.22	0.26	LNG	IPSC, ESC, BRST, BLD, SKIN, BRN, GI, MUS, THYM, PANC	SKIN		Nr2f2	CDH1	intronic
16	68768169	0.88	1	rs8056206	G	C	0.22	0.26	LNG	IPSC, ESC, BRST, BLD, SKIN, BRN, GI, MUS, THYM, PANC	SKIN		BDP1,Nr2f2	CDH1	intronic
16	68768193	0.96	1	rs8056338	G	A	0.23	0.28	LNG	IPSC, ESC, BRST, BLD, SKIN, BRN, GI, MUS, THYM, PANC			HNF4	CDH1	intronic
16	68768379	1	1	rs8056538	G	A	0.24	0.29	LNG	ESDR, IPSC, ESC, BRST, BLD, SKIN, BRN, GI, MUS, THYM, PANC	ESC,BRST,PLCNT,BRST,MUS,SKIN	CEBPB	CEBPA,CEBPB,CEBPD,CHX10,Dlx5,En-1,HLF,Hlxb9,Hoxa4,Hoxb3,Hsf,Lhx4,Pax-6,Pou2f2,Pou3f2,Pou6f1,Prrx2	CDH1	intronic
16	68768613	1	1	rs12448448	T	G	0.24	0.29	LNG	ESDR, IPSC, ESC, BRST, BLD, SKIN, BRN, GI, MUS, THYM, PANC	BRST,SKIN		AP-2,E2A,EBF,Egr-1,HEN1,Ik-3,RXRA,TBX5,ZEB1	CDH1	intronic
16	68769033	0.97	1	rs12930371	C	T	0.24	0.28	LNG	ESDR, IPSC, ESC, BRST, SKIN, GI, MUS, THYM, PANC, BLD	SKIN,SKIN	GATA1,GATA2	Pax-5	CDH1	intronic
16	68769823	1	1	rs28450087	T	G	0.24	0.29	ESC	ESDR, ESC, BRST, BLD, SKIN, MUS, THYM, PANC, SPLN			E2A,Lmo2-complex,Pax-4,ZEB1	CDH1	intronic
16	68769941	0.97	1	rs28628339	A	G	0.24	0.28	ESC	ESDR, ESC, BRST, BLD, SKIN, MUS, THYM, PANC, SPLN			Ik-1,NRSF	CDH1	intronic
16	68770165	1	1	rs12446407	G	A	0.24	0.29	ESC	ESDR, ESC, BRST, BLD, SKIN, GI, HRT, MUS, THYM, PANC, SPLN	BRST		Esr2,Lmo2-complex,Myc,RXRA,Rad21,SMC3,Sin3Ak-20,TCF12	CDH1	intronic
		1	1	rs76943359	A	G	0.24	0.29	ESC, HRT	ESDR, ESC, BRST, BLD, SKIN, BRN, GI, HRT, MUS, THYM, PANC, SPLN	ESC,ESDR,BRST,THYM,BRST,SKIN		PLAG1,PPAR,Roaz	CDH1	intronic
16	68770439	1	1	rs12443730	T	C	0.24	0.29	HRT	ESDR, ESC, BRST, BLD, SKIN, BRN, GI, HRT, MUS, THYM,	ESDR,IPSC,THYM,BRS		Maf,NF-E2,Nrf-2,PU	CDH1	intronic

										PANC, SPLN	T,SKIN		.1,STAT,TATA		
16	68772605	1	1	rs4783681	C	A	0.24	0.29		IPSC, BLD, SKIN, GI, PLCNT, THYM, PANC	BLD,SKIN		Mef2,PU.1	CDH1	intronic
16	68772636	1	1	rs4783570	G	T	0.24	0.29		IPSC, BLD, SKIN, PLCNT, THYM, PANC, GI	BLD,SKIN		AFP1,Nkx6-1	CDH1	intronic
16	68772875	0.99	1	rs4783571	C	T	0.24	0.28		IPSC, BLD, SKIN, PLCNT, THYM, PANC, GI	IPSC,THYM		Mrg1::Hoxa9,Zbtb3	CDH1	intronic
16	68773184	0.9	1	rs8045022	T	C	0.23	0.27		BLD, SKIN, PLCNT, THYM, GI, PANC	BRST,THYM,PANC,BRST,SKIN		FAC1,RREB-1	CDH1	intronic
16	68773185	0.88	1	rs8044058	G	A	0.23	0.26		BLD, SKIN, PLCNT, THYM, GI, PANC	BRST,THYM,PANC,BRST,SKIN		Ik-1,RREB-1	CDH1	intronic
16	68773925	0.97	1	rs12596061	A	G	0.24	0.28		ESDR, IPSC, BLD, SKIN, GI, PLCNT, THYM, PANC	PLCNT		Cdx,Dbx1,Evi-1,Fox,Foxa,Foxd3,Foxf1,Foxf2,Foxi1,Foxj1,Foxj2,Foxk1,Foxl1,Foxo,Foxp1,Foxq1,HDAC2,HNF1,Hoxb13,Mef2,Ncx,Pax-4,Pou3f4,Sox,TATA,Zfp105,p300	CDH1	intronic
16	68774380	0.96	1	rs12708889	C	T	0.24	0.28		ESDR, IPSC, BLD, SKIN, BRN, GI, PLCNT, THYM, PANC	PLCNT		CACD,Irf,Klf4,Klf7,Myc,NRSF,PPAR,Rad21,STAT,TATA	CDH1	intronic
16	68775304	1	1	rs13334326	C	T	0.24	0.29	BLD, MUS	ESDR, IPSC, BRST, BLD, SKIN, BRN, GI, PANC, PLCNT, THYM, MUS	THYM,BLD,CRVX,BRST	PAX5C20	Znf143	CDH1	intronic
16	68775370	1	1	rs13339591	T	C	0.24	0.29	BLD, MUS	ESDR, IPSC, BRST, BLD, SKIN, BRN, GI, PANC, PLCNT, THYM, MUS	IPSC,SKIN,OVRY,CRVX,MUS,SKIN		ATF3,Foxa,Ik-3	CDH1	intronic
16	68775375	1	1	rs13334294	A	C	0.24	0.29	BLD, MUS	ESDR, IPSC, BRST, BLD, SKIN, BRN, GI, PANC, PLCNT, THYM, MUS	SKIN,OVRY,GI,CRVX,MUS,SKIN,LNG		Ik-3	CDH1	intronic

16	68775737	1	1	rs9282650	T	A	0.24	0.29	BLD, MUS	IPSC, BRST, BLD, SKIN, GI, PANC, THYM, MUS	THYM		Cphx,Spz1	CDH1	intronic
16	68777020	1	1	rs17772411	T	C	0.23	0.29	BLD, MUS	ESC, ESDR, IPSC, STRM, BRST, BLD, SKIN, LIV, GI, KID, PANC, PLCNT, THYM, LNG, MUS, BRN	IPSC,BRST,BLD,SKIN,SKIN,THYM,GI,BLD,BRST,MUS,MUS,BRN,SKIN,LNG	EBF1,IRF4,NFKB,PAX5C20,PAX5N19,SP1,POL2B	Hic1	CDH1	intronic
16	68777834	0.97	0.99	rs35648619	C	T	0.23	0.28	IPSC, BRN	ESC, ESDR, BRST, BLD, SKIN, LIV, BRN, GI, PANC, PLCNT, THYM			HNF4,Nkx2	CDH1	intronic
16	68778398	0.99	1	rs9939049	A	T	0.23	0.29	IPSC, BRST, BLD, BRN, SPLN, SKIN	ESC, ESDR, IPSC, BRST, BLD, SKIN, LIV, BRN, GI, KID, PANC, LNG, PLCNT, THYM	BRST,GI,PANC,GI		AP-3,Arid5a,Foxa,Foxj2,Pax-4,Zfp105	CDH1	intronic
16	68779726	0.97	1	rs12922777	A	G	0.23	0.28	ESDR, SPLN	ESC, ESDR, IPSC, BRST, BLD, BRN, SKIN, LIV, GI, PANC, LNG, PLCNT, THYM, SPLN	BRST		E2F,Myc,RXRA,SIRT6,p53	CDH1	intronic
16	68780301	0.97	1	rs67359183	T	C	0.24	0.28		ESC, ESDR, IPSC, BRST, BRN, SKIN, LIV, GI, PANC, LNG, MUS, PLCNT, THYM, SPLN			Cdx,Cdx2,DMRT2,DMRT3,Dbx1,Evi-1,Foxa,Foxj1,Foxj2,Foxk1,Foxp1,HNF1,Hlx1,Hoxa10,Hoxa9,Hoxb13,Hoxb9,Hoxc6,Hoxc9,Hoxd10,Lhx3,Sox,Zfp105,p300	CDH1	intronic
16	68780413	0.97	1	rs2161664	C	T	0.24	0.28		ESC, ESDR, IPSC, BRST, BRN, SKIN, LIV, GI, PANC, LNG, MUS, PLCNT, THYM, SPLN			NF-E2	CDH1	intronic
16	68780599	0.96	1	rs2113199	A	G	0.24	0.28	ESDR	ESC, ESDR, IPSC, BRST, BRN, SKIN, LIV, GI, PANC, LNG, MUS, PLCNT, THYM, SPLN			Ascl2,BRCA1,E2A,Foxd1,Foxd3,Foxo,GR,Myf,Nanog,Sox	CDH1	intronic
16	68780687	0.99	1	rs12599517	T	C	0.24	0.28	ESDR	ESC, ESDR, IPSC, BRST, BRN, SKIN, LIV, GI, PANC, LNG, MUS, PLCNT, THYM, SPLN	ESC		AP-1,Maf,Nrf-2,SMC3	CDH1	intronic

16	68781045	0.97	1	rs2113200	T	A	0.24	0.28	ESDR	ESC, ESDR, IPSC, BRST, BLD, BRN, SKIN, LIV, GI, PANC, LNG, MUS, THYM, SPLN			Pax-5	CDH1	intronic
16	68781097	0.96	0.99	rs2113201	C	T	0.24	0.28	ESDR	ESC, ESDR, IPSC, BRST, BLD, BRN, SKIN, LIV, GI, PANC, LNG, MUS, THYM, SPLN			AP-1,Foxo,SPIB,YY1, ZID	CDH1	intronic
16	68781935	0.99	0.99	rs7186333	T	A	0.24	0.29	ESDR, BLD, THYM, GI	ESC, ESDR, IPSC, BRST, BLD, SKIN, LIV, BRN, GI, PANC, LNG, MUS, THYM, SPLN	KID,THYM,BLD,MUS, MUS	SRF		CDH1	intronic
16	68782357	0.99	0.99	rs7186084	G	C	0.19	0.29	ESDR, IPSC, BLD, SKIN, THYM, GI, HRT	ESC, ESDR, IPSC, BRST, BLD, SKIN, LIV, BRN, GI, PANC, LNG, MUS, THYM, SPLN	IPSC,BLD,BLD,BLD,SK IN,KID,LNG,GI,THYM, BLD,MUS,BLD	EBF1,ELF1,PAX5N1 9,PU1,TCF12	Hsf,Irf,Pax-5,Pou2f2 ,RXRA,STAT	CDH1	intronic
16	68783536	0.99	0.99	rs2059254	C	T	0.19	0.29	BRN, THYM, BLD	ESDR, ESC, IPSC, BRST, BLD, SKIN, LIV, BRN, GI, MUS, THYM, PANC	SKIN,THYM,BRST		HNF1	CDH1	intronic
16	68784342	0.99	0.99	rs7199991	T	G	0.19	0.29	BRN, THYM	ESDR, ESC, BRST, BLD, SKIN, LIV, BRN, GI, MUS, THYM, PANC, SPLN	SKIN,BRST		GATA,p53	CDH1	intronic
16	68784487	0.96	0.99	rs7198799	C	T	0.19	0.28	BRN, THYM	ESDR, ESC, BRST, SKIN, LIV, BRN, MUS, THYM, GI, PANC, BLD	LNG,BRST,SKIN,MUS	CMYC	E2F	CDH1	intronic
16	68785000	0.99	0.99	rs2961	T	C	0.19	0.29	BRN, GI	ESC, BRST, SKIN, LIV, BRN, PANC, THYM, GI, MUS	SKIN		ERalpha-a,Gm397,P ou2f2,Pou3f3,RORal pha1,Sox	CDH1	intronic
16	68785104	0.96	0.99	rs1981871	C	T	0.19	0.28	BRN, GI	ESC, BRST, SKIN, LIV, BRN, PANC, THYM, GI, MUS	SKIN		INSM1,MZF1::1-4,T FII-I,WT1,ZNF219	CDH1	intronic
16	68785304	0.97	0.99	rs9923610	C	G	0.20	0.29	BRN, GI	ESC, BRST, SKIN, LIV, BRN, PANC, THYM, GI, MUS	IPSC,BLD		Irf,Maf,NF-E2,SP1,T FII-I,ZNF263	CDH1	intronic
16	68785541	0.97	0.99	rs9923925	G	A	0.19	0.29	BRN, GI	ESC, BRST, BRN, SKIN, LIV, GI, PANC, MUS, THYM	IPSC		Pax-5	CDH1	intronic
16	68785711	0.95	0.99	rs9925923	C	T	0.19	0.28	BRN, GI, MUS	ESC, BRST, BRN, SKIN, LIV, GI, PANC, MUS, THYM			CDP	CDH1	intronic
16	68786815	0.97	0.99	rs9928796	A	G	0.19	0.29	IPSC, BRN, GI, LIV, MUS, BONE	ESDR, ESC, IPSC, FAT, STRM, BRST, MUS, BRN, SKIN, LIV, GI, KID, PANC, LNG, THYM, BONE	ESDR,LNG,BRST,SKIN ,SKIN,BRN,BRN,HRT,	USF1,FOSL2,FOXA1 ,FOXA2,HDAC2,HN	Myc	CDH1	intronic

											GI,GI,KID,MUS,MUS,PLCNT,GI,OVRY,PANC,GI,BLD,LIV,BRST,MUS,MUS,BRN,SKIN,SKIN,LNG	F4A,HNF4G,JUND,P300,RAD21,SP1,USF2			
16	68787043	0.97	0.99	rs9929218	G	A	0.19	0.29	IPSC, BRN, GI, LIV, MUS	ESDR, ESC, IPSC, FAT, STRM, BRST, MUS, BRN, SKIN, LIV, GI, KID, PANC, LNG, THYM, BONE	ESDR,BRST,SKIN,SKIN,ADRL,BRN,BRN,GI,MUS,MUS,GI,GI,PANC,LIV,BRST,MUS,MUS,BRN,SKIN,SKIN,LNG	HEY1	Myb	CDH1	intronic
16	68787223	0.95	0.99	rs9929239	C	T	0.19	0.28	IPSC, BRN, GI, LIV, MUS	ESDR, ESC, IPSC, FAT, STRM, BRST, MUS, BRN, SKIN, LIV, GI, KID, PANC, LNG, THYM, BONE	ESDR,SKIN,BRN,BRN,GI,OVRY,MUS,MUS,BRN,LNG	NRSF	CTCFL	CDH1	intronic
16	68787368	0.94	0.99	rs9929479	G	C	0.19	0.28	BRN, GI, LIV, MUS	ESDR, IPSC, FAT, STRM, BRST, MUS, BRN, SKIN, LIV, GI, KID, PANC, THYM, LNG, BONE	ESDR,SKIN,LIV			CDH1	intronic
16	68788438	0.87	0.95	rs12919719	C	G	0.20	0.28	BRN, GI, MUS	ESC, IPSC, STRM, BRST, SKIN, LIV, BRN, GI, MUS, PANC, PLCNT, LNG	LNG,MUS,MUS		GATA	CDH1	intronic
16	68788696	0.85	0.94	rs12924033	G	A	0.20	0.28	BRN, GI, MUS	ESC, IPSC, FAT, STRM, BRST, BLD, SKIN, LIV, BRN, GI, MUS, THYM, PANC, PLCNT	SKIN		CEBPG,Foxa,HDAC2,Pou1f1	CDH1	intronic
16	68788951	0.86	0.94	rs4552018	T	G	0.20	0.28	GI, MUS	ESC, ESDR, IPSC, FAT, STRM, BRST, BLD, SKIN, LIV, BRN, GI, MUS, THYM, PANC, PLCNT, SPLN	IPSC		ATF3,E2F,RXRA	CDH1	intronic
16	68789068	0.85	0.94	rs4420522	A	G	0.20	0.28	BLD, GI, MUS	ESC, ESDR, IPSC, FAT, STRM, BRST, BLD, SKIN, LIV, BRN, GI, MUS, THYM, LNG, PANC, PLCNT, SPLN	SKIN		TCF11::MafG	CDH1	intronic
16	68789221	0.86	0.94	rs10163398	C	G	0.20	0.28	FAT, BLD, GI, MUS	ESC, ESDR, IPSC, FAT, STRM, BRST, BLD, SKIN, LIV, BRN, GI, MUS, THYM, LNG, PANC, PLCNT, SPLN	SKIN		BCL,MAZ	CDH1	intronic
16	68789296	0.86	0.94	rs12596834	T	C	0.20	0.28	IPSC, FAT, BLD, GI, MUS	ESC, ESDR, IPSC, FAT, STRM, BRST, BLD, SKIN, LIV, BRN, GI,	ESDR		CACD,EBF,NRSF,Sin	CDH1	intronic

										MUS, THYM, LNG, PANC, PLCNT, SPLN, BONE			3Ak-20		
16	68790245	0.86	0.94	rs34097984	T	G	0.20	0.28	ESC, IPSC, FAT, STRM, BRST, BLD, LIV, GI, LNG, MUS, BRN, SKIN, BONE	ESC, ESDR, LNG, IPSC, FAT, STRM, BRST, BLD, MUS, BRN, SKIN, LIV, GI, PLCNT, THYM, OVRY, PANC, SPLN, BONE	ESC,ESDR,ESDR,ESDR,ESC,LNG,IPSC,BRST,SKIN,SKIN,KID,LNG,PLCNT,GI,THYM,GI,OVRY,PANC,LNG,LIV,BRST,MUS,MUS,BRN,SKIN,SKIN,LNG	TCF4,ERALPHA_A	ATF3,CTCF,Ets,GATA	CDH1	intronic
16	68790317	0.86	0.94	rs60302411	A	C	0.20	0.28	ESC, ESDR, IPSC, FAT, STRM, BRST, BLD, LIV, GI, LNG, MUS, BRN, SKIN, BONE	ESC, ESDR, LNG, IPSC, FAT, STRM, BRST, BLD, MUS, BRN, SKIN, LIV, GI, PLCNT, THYM, OVRY, PANC, SPLN, BONE	ESC,ESDR,ESDR,ESDR,ESC,LNG,IPSC,BRST,SKIN,SKIN,GI,PLCNT,GI,GI,OVRY,PANC,LNG,LIV,BRST,MUS,MUS,BRN,SKIN,LNG	GR,FOXA1,FOXA2,HDAC2,HNF4A,HNF4G,P300,TBP,TCF12,USF1,TCF4,CEBPB,ERALPHA_A,GATA3	AP-2,ERalpha-a	CDH1	intronic
16	68790589	0.86	0.94	rs35444713	C	T	0.20	0.28	ESC, ESDR, IPSC, FAT, STRM, BRST, BLD, LIV, GI, LNG, MUS, BRN, SKIN, BONE	ESC, ESDR, LNG, IPSC, FAT, STRM, BRST, BLD, MUS, BRN, SKIN, LIV, GI, PLCNT, THYM, OVRY, PANC, SPLN, BONE	ESC,ESC,IPSC,BRST,SKIN,SKIN,BRN,GI,OVRY,LNG,LIV,BRST,MUS,MUS,BRN,LNG	HEY1,POL2,TBP,TCF4,P300	HMG-IY,Irf	CDH1	intronic
16	68792206	0.88	0.95	rs57688464	G	C	0.20	0.28	ESC, IPSC, BRST, GI, LIV	ESC, ESDR, IPSC, FAT, BRST, BLD, SKIN, LIV, BRN, GI, PLCNT, LNG, PANC, SPLN, MUS, BONE	PLCNT			CDH1	intronic
16	68793717	0.84	0.93	rs34530130	C	T	0.20	0.28	LIV, GI	ESC, ESDR, LNG, IPSC, FAT, STRM, BRST, BLD, SKIN, LIV, BRN, GI, PANC, MUS, PLCNT, THYM, BONE	IPSC		BATF,Ets,Irf,RXRA,ZBTB33	CDH1	intronic
16	68793882	0.84	0.93	rs35794312	G	A	0.20	0.28	LIV, GI	ESC, ESDR, LNG, IPSC, BRST, BLD, STRM, SKIN, LIV, BRN, GI, PANC, MUS, THYM, PLCNT, BONE	PLCNT		CEBPB,CEBPD,Irf,PU.1	CDH1	intronic
16	68794270	0.84	0.93	rs33965787	G	A	0.20	0.28	LIV, GI	ESC, ESDR, IPSC, BRST, SKIN, LIV, BRN, GI, PANC, THYM, LNG, MUS, BLD	BLD		p300	CDH1	intronic

16	68794982	0.83	0.93	rs12928043	G	A	0.20	0.28	ESDR, LIV, LNG	ESC, IPSC, BRST, SKIN, LIV, BRN, GI, LNG, MUS			E2A,Lmo2-complex,NRSF,Sin3Ak-20,TCF12,ZEB1	CDH1	intronic
16	68795118	0.84	0.93	rs12599393	C	T	0.20	0.28	ESDR, LIV, LNG	ESC, IPSC, BRST, SKIN, LIV, BRN, GI, LNG, MUS			Mrg1::Hoxa9	CDH1	intronic
16	68796608	0.84	0.93	rs17715799	A	T	0.20	0.28	LIV, MUS	ESC, IPSC, BRST, SKIN, LIV, BRN, GI, PLCNT, PANC, LNG, MUS	IPSC,MUS,MUS		Foxj1,Foxo,Irf,Sox	CDH1	intronic
16	68798847	0.83	0.92	rs1075959	A	G	0.21	0.28	SKIN, MUS	ESC, ESDR, IPSC, FAT, STRM, BRST, SKIN, BRN, GI, HRT, MUS, PLCNT, THYM, PANC, LNG, BLD, LIV	IPSC,BRST,SKIN,PLCNT,BRST,MUS,MUS,BLD,SKIN		PLZF,TCF4	CDH1	intronic
16	68799040	0.81	0.92	rs1862748	C	T	0.20	0.29	SKIN, MUS	ESC, ESDR, IPSC, FAT, STRM, BRST, SKIN, BRN, GI, HRT, MUS, PLCNT, THYM, PANC, LNG, BLD	PLCNT,THYM,BRST,MUS,MUS			CDH1	intronic
16	68800204	0.83	0.92	rs4783685	C	T	0.20	0.28		ESC, ESDR, LNG, IPSC, FAT, STRM, BRST, SKIN, BRN, GI, ADRL, MUS, PLCNT, THYM, PANC, BLD, LIV, BONE	ESC,SKIN,BLD	YY1	HDAC2,Irf,Zfp105	CDH1	intronic
16	68800253	0.83	0.92	rs4783686	C	G	0.20	0.28		ESC, ESDR, LNG, IPSC, FAT, STRM, BRST, SKIN, BRN, GI, ADRL, MUS, PLCNT, THYM, PANC, BLD, CRVX, LIV, BONE	ESDR,ESC,BLD,BLD,BLD,SKIN,LNG,MUS,MUS,PLCNT,THYM,GI,BLD,CRVX,BRST,MUS	YY1,CTCF		CDH1	intronic
16	68802518	0.82	0.92	rs8060418	T	C	0.20	0.29	ESDR, FAT, SKIN	ESDR, ESC, LNG, FAT, STRM, BRST, MUS, SKIN, GI, PLCNT, BRN, BONE	LNG,BRST,SKIN,SKIN,SKIN,PLCNT,GI,PANC,BRST,MUS,MUS,BRN,SKIN,SKIN,LNG		AP-1	CDH1	intronic
16	68802588	0.82	0.92	rs8063387	C	G	0.20	0.29	ESDR, FAT, SKIN	ESDR, ESC, LNG, FAT, STRM, BRST, MUS, SKIN, GI, PLCNT, BRN, BONE	LNG,BRST,SKIN,MUS,MUS,BRN,SKIN,SKIN,LNG		Egr-1,Irf,Myf,NF-E2,RXRA,Rad21,SP1	CDH1	intronic
12	50187707	1	1	rs7315690	C	T	0.20	0.32	FAT, STRM, BLD, MUS, BRN, SKIN, GI, BONE	ESDR, LNG, IPSC, FAT, STRM, BRST, BLD, MUS, BRN, SKIN, VAS, GI, PANC, PLCNT, THYM, HRT, OVRY, SPLN, LIV, BONE	ESDR,LNG,BLD,SKIN,SKIN,SKIN,SKIN,BRN,	CTCF,EBF1,POL24H8	ERalpha-a,STAT,Zbtb3,Znf143	RP3-405J10.3	intronic

											BRN,KID,MUS,MUS,OVRY,MUS,BLD,MUS,MUS,BRN,SKIN,SKIN,LNG				
12	50189367	1	1	rs7138420	A	G	0.20	0.32	FAT, STRM, BLD, SKIN, GI, BRN, BONE	ESDR, LNG, FAT, STRM, BRST, BLD, MUS, BRN, SKIN, VAS, GI, ADRL, PLCNT, THYM, HRT, SPLN, LIV, BONE	SKIN,ADRL,KID,PLCNT,THYM,BLD,SKIN,LNG		E2F,PLZF,Pbx-1	RP3-405J10.3	intronic
12	50189421	1	1	rs7138622	C	T	0.20	0.32	FAT, STRM, BLD, SKIN, GI, BRN, BONE	ESDR, LNG, FAT, STRM, BRST, BLD, MUS, BRN, SKIN, VAS, GI, ADRL, PLCNT, THYM, HRT, LIV, BONE	THYM,SKIN		LBP-1	RP3-405J10.3	intronic
12	50198830	0.81	0.94	rs11169314	A	T	0.28	0.34	FAT, ESC	ESDR, FAT, STRM, BRST, MUS, SKIN, BRN, GI, PLCNT, BONE			Cart1,DMRT3,DMRT4,DMRT5,Foxf1,Foxj2,Foxl1,HNF1,Mrg1::Hoxa9,Pou6f1,RORalpha1,Sox,Zfp105	LIMA1	intronic
12	50201480	0.81	0.94	rs11169315	A	T	0.28	0.34	MUS, SKIN, LNG	ESDR, ESC, LNG, IPSC, FAT, STRM, BRST, MUS, SKIN, GI, PLCNT, CRVX, VAS, BRN, BONE	SKIN			LIMA1	5'-UTR
15	90517889	0.86	0.95	rs12903058	G	T	0.24	0.70	CRVX	BLD, SKIN, CRVX, MUS	BLD		Foxj1,Sox	12kb 5' of CRTC3	
15	90519669	0.86	0.95	rs1973537	T	C	0.24	0.70	CRVX	ESDR, LNG, FAT, STRM, BRST, MUS, SKIN, BLD, CRVX, VAS, BRN, BONE	KID		Foxp1,Mef2	10kb 5' of CRTC3	
15	90521301	0.92	0.96	rs4031382	A	G	0.24	0.71	CRVX	ESDR, LNG, BLD, SKIN, VAS, GI, CRVX			Mef2	8.6kb 5' of CRTC3	
15	90525650	0.94	0.97	rs7495784	T	C	0.24	0.71	CRVX	SKIN			Roaz,SRF	4.3kb 5' of CRTC3	
15	90526132	0.88	0.95	rs28406958	C	A	0.24	0.70	LNG				FAC1,Foxd3,Foxj1,Foxk1,Foxo,Foxp1,H	3.8kb 5' of CRTC3	

													DAC2,Mef2,Sox,Zfp 105		
15	90527277	0.94	0.97	rs11633723	T	C	0.24	0.71	CRVX	CRVX			SIRT6	2.6kb 5' of CRTC3	
15	90531554	0.92	0.97	rs7496461	T	G	0.20	0.71	ESC, ESDR, LNG, IPSC, FAT, STRM, BRST, BLD, MUS, BRN, SKIN, VAS, LIV, GI, HRT, KID, PANC, PLCNT, THYM, OVRY, CRVX, BONE	ESC, ESDR, LNG, IPSC, FAT, STRM, BRST, BLD, MUS, BRN, SKIN, LIV, GI, ADRL, HRT, KID, PANC, PLCNT, THYM, OVRY, SPLN, CRVX, VAS, BONE	CRVX		Foxp1,HDAC2,Nanog,TATA	CRTC3	intronic
15	90534590	1	1	rs11852389	G	A	0.24	0.71	BLD, BRN, GI, CRVX	ESC, ESDR, LNG, FAT, STRM, BRST, BLD, SKIN, VAS, LIV, BRN, GI, HRT, MUS, PLCNT, OVRY, PANC, SPLN, CRVX, BONE			Arid3a,Dbx2,Esx1,Hbp1,Hoxb13,Hoxb7,Pax7,Phox2a,Pou3f2,Pou4f3,Sox,Vax2	CRTC3	intronic
15	90536233	0.99	1	rs6496685	G	C	0.24	0.71	ESDR, BLD, LIV, BRN, HRT, GI, CRVX	ESC, ESDR, LNG, IPSC, FAT, STRM, BRST, BLD, BRN, SKIN, VAS, LIV, GI, ADRL, HRT, KID, PANC, MUS, PLCNT, OVRY, SPLN, CRVX, BONE	SKIN,HRT,CRVX		Mef2,Pou2f2	CRTC3	intronic
15	90536631	0.97	1	rs11637971	A	C	0.24	0.72	ESDR, BLD, LIV, BRN, HRT, GI, CRVX	ESC, ESDR, LNG, IPSC, FAT, STRM, BRST, BLD, BRN, SKIN, VAS, LIV, GI, ADRL, HRT, KID, PANC, MUS, PLCNT, OVRY, SPLN, CRVX	HRT,KID,GI,CRVX		Pou3f4,p300	CRTC3	intronic
15	90536688	0.97	1	rs11638010	A	G	0.24	0.72	ESDR, BLD, LIV, BRN, HRT, GI, CRVX	ESC, ESDR, LNG, IPSC, FAT, STRM, BRST, BLD, BRN, SKIN, VAS, LIV, GI, ADRL, HRT, KID, PANC, MUS, PLCNT, OVRY, SPLN, CRVX	ESDR,BLD,SKIN,ADRL,BRN,HRT,GI,KID,PLCNT,GI,CRVX		Foxp1	CRTC3	intronic
		0.9	0.97	rs34362718	A	AAAG,AAGT	0.23	0.70	ESDR, BLD, LIV, GI	ESC, ESDR, LNG, FAT, BLD, SKIN, VAS, LIV, BRN, GI, ADRL, HRT, KID, PANC, PLCNT, OVRY, MUS, CRVX	BLD,CRVX	HNF4G,MAFF,MAFK,SP1		CRTC3	intronic
15	90537544	0.97	1	rs8027179	T	C	0.24	0.72	BLD, LIV, GI	ESC, ESDR, FAT, BLD, SKIN, LIV, BRN, GI, ADRL, HRT, KID,			TCF12	CRTC3	intronic

										PANC, LNG, OVRY, MUS, CRVX					
15	90537588	0.88	0.97	rs8042568	C	G	0.22	0.70	BLD, LIV, GI	ESC, ESDR, FAT, BLD, SKIN, LIV, BRN, GI, ADRL, HRT, KID, PANC, LNG, OVRY, MUS, CRVX			YY1	CRTC3	intronic
15	90537789	0.96	0.99	rs8027581	T	C	0.24	0.71	BLD, LIV, GI	ESC, ESDR, FAT, BLD, SKIN, LIV, BRN, GI, ADRL, HRT, KID, PANC, LNG, OVRY, MUS, CRVX				CRTC3	intronic
15	90538559	0.97	1	rs11635361	G	T	0.23	0.72	BLD	ESC, BLD, SKIN, FAT, LIV, BRN, GI, HRT, PANC, OVRY, MUS, CRVX	BLD			CRTC3	intronic
9	19278493	0.8	0.92	rs2175380	G	T	0.46	0.27	GI	FAT, BLD, VAS, BRN, PANC, HRT, MUS, GI				10kb 5' of DENND4C	
9	19278528	0.8	0.92	rs2175379	C	T	0.46	0.27	GI	FAT, BLD, VAS, BRN, PANC, HRT, MUS, GI	BRN		Sox	10kb 5' of DENND4C	
9	19278905	0.8	0.92	rs4977294	G	C	0.46	0.27		FAT, BLD, VAS, BRN, PANC, HRT, MUS			ELF1,PU.1,TATA	9.7kb 5' of DENND4C	
9	19278909	0.8	0.92	rs4977295	G	A	0.46	0.27		FAT, BLD, VAS, BRN, PANC, HRT, MUS			En-1,Hoxa3,Hoxa7, Hoxd8,Nkx2	9.7kb 5' of DENND4C	
9	19279436	0.8	0.92	rs56286908	A	G	0.45	0.26		BLD, VAS, PANC, HRT, MUS			BCL,CCNT2,CHD2,Egr-1,Ik-1,Irf,Nrf1,Rad21,SETDB1,SP1,Sp4,TATA,ZBTB7A,Zfp740,Znf143	9.2kb 5' of DENND4C	
9	19279500	0.8	0.92	rs4977524	C	T	0.46	0.27		BLD, VAS, PANC, HRT, OVRY, MUS			CHOP::CEBPalpha,NF-kappaB	9.1kb 5' of DENND4C	
9	19279569	0.81	0.92	rs4977525	T	C	0.46	0.27		BLD, VAS, PANC, HRT, OVRY, MUS			BDP1	9.1kb 5' of DENND4C	
9	19279691	0.81	0.92	rs4977526	A	G	0.46	0.27		BLD, VAS, PANC, HRT, OVRY, MUS			CIZ,Evi-1,Foxp1,HDAC2,Zfp105,p300	8.9kb 5' of DENND4C	

9	19280213	0.81	0.92	rs1854550	G	C	0.46	0.27		BLD, VAS, HRT, OVRY			GR,Rad21,YY1,Zbtb3	8.4kb 5' of DENND4C	
9	19304144	0.81	0.91	rs10964090	C	T	0.44	0.27					BCL,Evi-1,Foxp1,GATA,HDAC2,Irf,Pax-4,RXRA,TATA,Zfp105,p300	DENND4C	intronic
9	19304319	0.82	0.93	rs12684097	G	C	0.44	0.27					Crx,LUN-1,Pitx2	DENND4C	intronic
9	19309761	0.8	0.94	rs10964094	C	T	0.45	0.26					Arid3a,Arid5b,Foxp1,Hmx,Hoxa3,Hoxb6,Hoxc6,Isl2,Msx-1,Nkx6-1,Pax7,Pou2f2,Pou5f1,SIX5,Sox,p300	DENND4C	intronic
9	19331035	0.99	1	rs67601721	G	A	0.44	0.27					RFX5	DENND4C	intronic
9	19331573	1	1	rs17818670	G	A	0.44	0.28		GI			Hdx,STAT	DENND4C	intronic
10	68447236	0.82	0.93	rs61855132	G	A	0.14	0.10					AP-4,E2A,HEN1,Hic1,Klf7,LBP-1,Nanog,RP58,Sin3Ak-20,TCF12	DNA2	intronic
10	68447561	0.82	0.93	rs78353373	C	T	0.15	0.10					BCL,GZF1,RREB-1	DNA2	intronic
10	68451280	0.84	0.93	rs10998177	A	G	0.15	0.10					AP-2rep,Arid5a,Foxa,Foxj2,GATA,HP1-site-factor,Tgif1	DNA2	intronic
10	68454207	0.97	0.99	rs10998184	T	A	0.15	0.11		VAS			Mef2	DNA2	intronic
10	68456870	0.99	1	rs10998191	C	T	0.15	0.10					ATF3	DNA2	intronic
10	68457295	1	1	rs10998194	T	C	0.16	0.11		BLD	BLD			DNA2	intronic

10	68457704	1	1	rs10998195	T	C	0.15	0.11					Pax-4	DNA2	intronic
10	68460778	1	1	rs2031098	G	A	0.15	0.11	ESDR		BLD		Brachyury,Eomes,Irf,SRF	DNA2	intronic
10	68461437	1	1	rs4388762	G	A	0.15	0.11				TFIIIC110	Gfi1,Hoxa7	RN5S319	intronic
10	68461731	1	1	rs61855137	G	T	0.15	0.11					AP-1,BDP1,Evi-1,Maf,Myc,p300	DNA2	intronic
10	68463824	1	1	rs61855140	C	T	0.15	0.11		HRT, MUS			Irf	DNA2	intronic
10	68464816	0.97	0.99	rs138356255	C	T	0.15	0.11					Irf	DNA2	intronic
10	68465024	1	1	rs201249484	T	TC	0.15	0.11		BLD			CIZ,Evi-1,Foxm1,Foxp1,HDAC2,HMG-IY,Irf,Pax-4,Pou3f2,SIX5,Smad,Zfp105,Znf143,p300	DNA2	intronic
10	68466475	0.99	1	rs147636877	GA	G	0.15	0.11		BLD			Foxp3,Pax-4	DNA2	intronic
10	68468207	0.97	0.99	rs10998205	C	T	0.15	0.11	BLD, BRN	IPSC, LNG, BLD			DMRT5,Smad3	DNA2	synonymous
10	68470718	0.97	0.99	rs10509302	G	C	0.15	0.11	ESC, ESDR, LNG, IPSC, FAT, STRM, BRST, BLD, MUS, BRN, SKIN, VAS, GI, ADRL, HRT, KID, THYM, OVRY, PLCNT, CRVX, LIV, BONE	ESDR, ESC, LNG, IPSC, FAT, STRM, BRST, BLD, MUS, BRN, SKIN, LIV, GI, ADRL, HRT, KID, PANC, THYM, OVRY, SPLN, CRVX, VAS, BONE	ESDR,BRST,PLCNT,BLD	INI1	Mtf1	DNA2	intronic
10	68471470	0.97	0.99	rs2281701	G	A	0.15	0.11	ESC, ESDR, LNG, IPSC, FAT, STRM, BRST, BLD, MUS, BRN, SKIN, VAS, LIV, GI,	ESDR, ESC, FAT, STRM, BRST, BLD, MUS, BRN, SKIN, LIV, GI, ADRL, HRT, PANC, LNG, PLCNT, THYM, SPLN, CRVX, VAS, BONE	ESDR,ESDR,ESC,LNG,BRST,BLD,SKIN,SKIN,SKIN,GI,MUS,GI,CRV	GATA2	Foxq1,Zfp105	DNA2	intronic

									ADRL, HRT, KID, PANC, PLCNT, THYM, OVRY, SPLN, CRVX, BONE		X,BLD				
10	68471655	0.97	0.99	rs11818335	G	A	0.15	0.11	ESC, ESDR, LNG, IPSC, FAT, STRM, BRST, BLD, MUS, BRN, SKIN, VAS, LIV, GI, ADRL, HRT, KID, PANC, PLCNT, THYM, OVRY, SPLN, CRVX, BONE	ESDR, ESC, IPSC, FAT, STRM, BRST, BLD, MUS, BRN, SKIN, LIV, GI, ADRL, HRT, LNG, PLCNT, THYM, SPLN, CRVX, VAS, BONE	ESC,ESDR,ESDR,ESDR,ESC,IPSC,IPSC,BRST,SKIN,SKIN,ADRL,HRT,KID,LNG,MUS,GI,THYM,BLD,CRVX,LIV,BRST,BLD,SKIN	BCLAF1,SIN3AK20,POL2,POL24H8,TAF1,E2F4,GTF2B,HEY1,HMGN3,HAE2F1	Hic1	DNA2	intronic
10	68473370	0.97	0.99	rs145182274	CT	C	0.15	0.11	BRST, BLD, BRN, GI, KID, LNG, PLCNT, SPLN, CRVX	STRM, BRST, SKIN, BRN, GI, LNG, CRVX, MUS			GR,HIF1,HP1-site-factor	1.2kb 5' of DNA2	
10	68473496	0.97	0.99	rs61855143	T	C	0.15	0.11	BRST, BLD, BRN, KID, LNG, SPLN	STRM, BRST, SKIN, BRN, GI, LNG, CRVX	VAS,BLD		Msx-1,Nkx3	1.4kb 5' of DNA2	
10	68476877	0.97	0.99	rs7907286	T	C	0.15	0.11					Mef2,Pou2f2,Pou3f3,SIX5	1.1kb 3' of RP11-9E13.2	
10	68477244	0.97	0.99	rs74797509	G	A	0.15	0.11					Myc,NRSF	753bp 3' of RP11-9E13.2	
10	68477894	0.97	0.99	rs61856467	C	T	0.15	0.11						103bp 3' of RP11-9E13.2	
10	68480566	0.95	0.99	rs78924920	G	A	0.15	0.11						RP11-9E13.2	

10	68480585	0.97	0.99	rs76456007	C	T	0.15	0.11					HNF4,Irf,SP1,TATA	RP11-9E13.2	
10	68480898	0.97	0.99	rs61856468	T	G	0.15	0.11					Cdx,GR,HDAC2	133bp 5' of RP11-9E13.2	
10	68481292	0.97	0.99	rs10998215	C	T	0.15	0.11	GI				CTCF,GR	527bp 5' of RP11-9E13.2	
10	68482042	0.97	0.99	rs11813313	G	A	0.15	0.11					Ets,Gfi1,Mef2,NF-kappaB	721bp 3' of SLC25A16	
10	68483189	0.97	0.99	rs4350264	G	A	0.15	0.11						SLC25A16	3'-UTR
10	68483862	0.97	0.99	rs10998220	C	T	0.15	0.11					Sin3Ak-20,YY1	SLC25A16	intronic
10	68484599	0.97	0.99	rs76111109	T	C	0.15	0.11					Fox,Foxa,Foxd3,Foxf1,Foxi1,Foxj1,Foxj2,Foxl1,Foxp1,Foxq1,IRC900814,Pax-4,Sox,Zfp105	SLC25A16	intronic
10	68485214	0.83	0.99	rs7906130	A	C	0.15	0.12			BRST		BDP1,LUN-1	SLC25A16	intronic
10	68485240	0.97	0.99	rs7906263	C	A	0.15	0.11			BRST		STAT	SLC25A16	intronic
10	68486738	0.97	0.99	rs12220392	T	C	0.15	0.11					ATF3,CDP,DEC,Nkx2,SREBP	SLC25A16	intronic
10	68487863	0.97	0.99	rs10998223	T	C	0.15	0.11					HDAC2,TATA,Zfp105,p300	SLC25A16	intronic

10	68492445	0.97	0.99	rs10998225	T	C	0.15	0.11	FAT, BLD	LNG, FAT, STRM, BLD	LNG	CEBPB	AIRE,Dlx5,En-1,p300	SLC25A16	intronic
10	68492540	0.97	0.99	rs61856472	G	A	0.15	0.11	FAT, BLD	LNG, FAT, STRM, BLD			CDP,PLAG1,Zfx	SLC25A16	intronic
10	68493852	0.97	0.99	rs76064348	A	T	0.15	0.11		FAT			Mef2,PLZF,TATA,Zfp105	SLC25A16	intronic
10	68496054	0.89	0.94	rs10998228	T	A	0.15	0.11	ESC, IPSC	ESC, ESDR, IPSC, FAT			Pou5f1,Sox	SLC25A16	intronic
10	68500156	0.96	0.99	rs61856473	A	G	0.15	0.10					AFP1,CDP,HNF1,Homez,Hoxa10,Hoxc9,Pou4f3	SLC25A16	intronic
10	68502099	0.96	0.99	rs77601011	G	C	0.15	0.10	GI	ESC, PANC, LNG			Maf,Myc,NF-E2,NRSF,ZNF263	SLC25A16	intronic
10	68508104	0.9	0.97	rs10998233	G	A	0.15	0.10		ESDR, GI			E2A,ZEB1	SLC25A16	intronic
10	68509493	0.93	0.97	rs9633584	T	A	0.15	0.10						SLC25A16	intronic
10	68510265	0.9	0.96	rs79096616	C	T	0.13	0.10					ATF3,Maf	SLC25A16	intronic
10	68510656	0.9	0.96	rs61856474	C	G	0.13	0.10						SLC25A16	intronic
10	68513939	0.9	0.96	rs10998241	A	C	0.13	0.10						SLC25A16	intronic
10	68514173	0.9	0.96	rs10998242	C	T	0.13	0.10					ERalpha-a,Nrf-2,Rad21,TCF11::MafG	SLC25A16	intronic
10	68514501	0.9	0.96	rs10998243	A	C	0.13	0.10					CDP,Cart1,Dbx1,Foxd3,HNF1,Hoxb13,Hoxd8,Mef2,Ncx,Pax-4,Sox,Zfp105	SLC25A16	intronic
10	68514862	0.9	0.96	rs10998244	C	T	0.13	0.10					HNF1	SLC25A16	intronic
10	68516323	0.9	0.96	rs61856475	C	G	0.13	0.10	SKIN	ESC, LNG, FAT, BRST, MUS, SKIN, GI, LIV, BRN, BONE	BRST,SKIN,PLCNT,GI	GATA3,P300		SLC25A16	intronic
10	68518123	0.86	0.96	rs144616853	T	TA	0.13	0.10	SKIN	ESC, LNG, FAT, SKIN, GI, LIV, BONE	PLCNT		Foxa,Foxo,Mef2,SIX5,Zfp105	SLC25A16	intronic

10	68518968	0.85	0.96	rs148457232	G	A	0.13	0.10		SKIN, GI			LXR,Myc,Sp4	SLC25A16	intronic
10	68519669	0.89	0.96	rs61856477	C	T	0.13	0.10		GI			Maf,NRSF	SLC25A16	intronic
10	68521176	0.89	0.96	rs12219699	A	G	0.13	0.10		LNG				SLC25A16	intronic
10	68522737	0.9	0.96	rs61856494	C	T	0.13	0.10		BLD, FAT, LNG, PANC			HNF4,NRSF,Znf143	SLC25A16	intronic
10	68523214	0.9	0.96	rs11816020	T	C	0.13	0.10	GI	BLD, FAT, LIV, GI, LNG, PANC			BDP1,GR,LUN-1	SLC25A16	intronic
10	68525691	0.9	0.96	rs142458817	G	A	0.13	0.10	ESC, ESDR, IPSC, FAT, BLD, STRM, BRN, SKIN, LIV, GI, HRT, LNG, PANC, PLCNT	ESC, ESDR, FAT, BRST, BLD, STRM, MUS, BRN, SKIN, LIV, GI, THYM, LNG, PANC, PLCNT			p300	SLC25A16	intronic
10	68526637	0.9	0.96	rs10998249	C	A	0.13	0.10	ESC, ESDR, LNG, IPSC, FAT, STRM, BRST, BLD, MUS, BRN, SKIN, VAS, LIV, GI, ADRL, HRT, PANC, PLCNT, THYM, OVRY, CRVX, BONE	ESC, ESDR, LNG, IPSC, FAT, STRM, BRST, BLD, MUS, BRN, SKIN, LIV, GI, ADRL, HRT, PLCNT, THYM, PANC, CRVX, VAS, BONE	BLD,SKIN,GI,PLCNT		PPAR,RREB-1,SIX5,Znf143	SLC25A16	intronic
10	68533035	0.85	0.93	rs144294219	C	T	0.13	0.10					BDP1,GR,LUN-1	5.6kb 5' of SLC25A16	
10	68533049	0.82	0.93	rs142630378	TAC	T	0.13	0.11					Crx,Pitx2	5.6kb 5' of SLC25A16	
10	68537990	0.85	0.93	rs3847341	G	A	0.13	0.10	ESDR, GI, MUS, SPLN	ESDR, SKIN, MUS, LIV	PLCNT		CTCF,SETDB1	11kb 5' of SLC25A16	
10	68545235	0.84	0.93	rs61187453	G	A	0.13	0.10		MUS			BDP1,Maf,Nr2f2	15kb 5' of TET1	
11	61781553	0.88	0.99	rs174533	G	A	0.41	0.36	ESDR, SKIN, SPLN, CRVX	ESDR, IPSC, ESC, BRST, BLD, SKIN, BRN, GI, HRT, MUS, PLCNT, LNG	LNG		PU.1	C11orf9	intronic
11	61783884	0.86	0.97	rs174535	T	C	0.41	0.36	SKIN, SPLN, CRVX	ESDR, LNG, IPSC, ESC, STRM, BLD, SKIN, BRN, GI, MUS, PLCNT	ESC,KID,BRN	CTCF	Mrg	C11orf9	synonymous

11	61784455	0.87	0.98	rs174536	A	C	0.41	0.36	SKIN, SPLN, CRVX	ESDR, LNG, IPSC, ESC, STRM, BLD, SKIN, BRN, GI, MUS, PLCNT	LIV		HEN1,ZBTB7A	C11orf9	intronic
11	61785208	0.87	0.98	rs174537	G	T	0.41	0.36	ESDR, SKIN, SPLN, CRVX	ESDR, IPSC, ESC, BLD, STRM, SKIN, BRN, GI, MUS, PLCNT			HNF4	C11orf9	intronic
11	61790331	0.83	0.94	rs102275	T	C	0.41	0.37	ESDR, ESC, BLD, BRN, SKIN, GI, THYM, CRVX, BONE	ESDR, LNG, IPSC, FAT, BRST, BLD, STRM, MUS, BRN, SKIN, LIV, GI, HRT, KID, THYM, PANC, PLCNT, SPLN, CRVX				C11orf10	intronic
11	61790354	0.89	0.99	rs102274	T	C	0.41	0.36	ESC, ESDR, BLD, BRN, SKIN, GI, THYM, CRVX, BONE	ESDR, LNG, IPSC, FAT, BRST, BLD, STRM, MUS, BRN, SKIN, LIV, GI, HRT, KID, THYM, OVRY, PANC, PLCNT, SPLN, CRVX			EWSR1-FLI1	C11orf10	intronic
11	61796827	1	1	rs4246215	G	T	0.41	0.38	SKIN, BRN, GI, BLD, CRVX	ESC, ESDR, IPSC, BRST, BLD, BRN, SKIN, GI, ADRL, KID, MUS, THYM, PANC, LNG, CRVX	CRVX	CTCF	Nanog	FEN1	3'-UTR
11	61798436	1	1	rs174541	T	C	0.41	0.38	SKIN, BRN, CRVX, BLD	ESDR, FAT, BRST, BLD, MUS, SKIN, LIV, BRN, GI, SPLN, LNG, CRVX	THYM,BLD	POL2	Arnt,BHLHE40,Myc, SREBP,XBP-1	FADS2	
11	61801834	0.89	1	rs174545	C	G	0.41	0.36	SKIN, LNG, BLD	ESDR, FAT, SKIN, BLD, GI, PLCNT, LNG, CRVX, BRN	IPSC		BDP1,NF-Y,PU.1,TA TA	FADS1	3'-UTR
11	61802358	0.89	1	rs174546	C	T	0.41	0.36	ESDR, SKIN, LNG, BLD	ESDR, SKIN, BLD, GI, PLCNT, LNG, CRVX	BLD		BDP1,RXRA	FADS1	3'-UTR
11	61803311	0.89	1	rs174547	T	C	0.41	0.36	ESDR, SKIN, BLD	ESDR, SKIN, BLD, ADRL, GI, PLCNT, HRT, LNG, CRVX, BONE			AIRE,Egr-1	FADS1	intronic
11	61804006	0.89	1	rs174550	T	C	0.41	0.36	ESDR, SKIN, LNG, BLD	ESDR, FAT, BRN, SKIN, ADRL, MUS, GI, LNG, PLCNT, HRT, CRVX, VAS, BLD, BONE		POL2,POL24H8	ERalpha-a	FADS1	intronic
11	61806212	0.82	0.99	rs174551	T	C	0.38	0.34	SKIN	BRN, SKIN, LNG, BLD			AhR::Arnt,Arnt,CTC F,ELF1,NRSF,Rad21, YY1,p300	FADS1	intronic
11	61807686	0.89	1	rs174553	A	G,T	0.41	0.36		FAT, SKIN, BLD	MUS,BLD			FADS1	intronic
11	61811991	0.89	1	rs174554	A	G	0.41	0.36	SKIN, SPLN, CRVX	ESDR, FAT, BLD, SKIN, GI, ADRL, BRN, THYM, PLCNT, MUS, CRVX	PANC,MUS,CRVX,BL D		AP-1,BATF,GATA,H MGN3	FADS1	intronic
11	61817672	0.89	1	rs174562	A	G	0.41	0.36	ESC, ESDR, LNG, IPSC, FAT, STRM, BRST, BLD, MUS, BRN, SKIN, GI, ADRL, HRT,	ESC, ESDR, LNG, IPSC, FAT, STRM, BRST, BLD, MUS, BRN, SKIN, LIV, GI, ADRL, HRT, PLCNT, THYM, PANC, SPLN, CRVX, VAS, BONE	ESDR,ESDR,ESDR,ESD R,SKIN,BRN,KID,LNG, OVRY,MUS,SKIN		Znf143	FADS1	

									KID, THYM, OVRY, PLCNT, SPLN, CRVX, LIV, VAS, BONE						
11	61820833	0.88	0.99	rs174564	A	G	0.41	0.36	SKIN, LNG, BRST	ESC, ESDR, LNG, FAT, BRST, STRM, MUS, SKIN, VAS, LIV, GI, PANC, HRT, CRVX, BLD, BRN, BONE			ERalpha-a,Rad21,SMC3,SP1	FADS1	
11	61824890	0.84	0.96	rs174566	A	G	0.41	0.36	ESC, SKIN	BLD, SKIN, BRN, GI				FADS1	
11	61825533	0.84	0.96	rs174567	A	G	0.41	0.36	SKIN	SKIN, BRN			HNF4,RXRA,VDR	FADS1	
11	61826344	0.86	0.99	rs174568	C	T	0.41	0.35	ESDR, ESC, IPSC, BLD, BRN, SKIN, LIV, GI, THYM, SPLN, BONE	ESC, ESDR, LNG, IPSC, BLD, STRM, BRN, SKIN, LIV, GI, ADRL, HRT, MUS, PLCNT, THYM	MUS,LIV			FADS1	
11	61827448	0.86	0.98	rs3834458	CT	C	0.41	0.36	ESC, ESDR, LNG, IPSC, FAT, STRM, BRST, BLD, MUS, BRN, SKIN, VAS, LIV, GI, ADRL, HRT, KID, PANC, PLCNT, THYM, OVRY, SPLN, BONE	ESDR, ESC, LNG, IPSC, FAT, STRM, BRST, BLD, MUS, BRN, SKIN, LIV, GI, ADRL, PLCNT, THYM, HRT, PANC, SPLN, VAS, BONE	ESC,ESDR,ESDR,ESDR,ESC,IPSC,BLD,BLD,BLD,BLD,BLD,BLD,SKIN,SKIN,SKIN,ADRL,BRN,BRN,HRT,KID,LNG,MUS,MUS,PLCNT,GI,THYM,MUS,BLD,VAS,BLD,BRN,SKIN	PU1,YY1,NFKB,TAF1	CTCF,Smad3,Smad,TCF4	FADS1	
11	61828850	0.87	0.98	rs5792235	CA	C	0.41	0.36	ESC, ESDR, LNG, IPSC, FAT, STRM, BRST, BLD, MUS, BRN, SKIN, VAS, LIV, GI, ADRL, HRT, KID, PANC, PLCNT, THYM, OVRY, SPLN, CRVX, BONE	ESDR, ESC, LNG, FAT, STRM, BRST, BLD, MUS, BRN, SKIN, LIV, GI, ADRL, HRT, PLCNT, THYM, OVRY, PANC, SPLN, BONE	ESDR,ESDR,ESC,BLD,BLD,BLD,BLD,SKIN,SKIN,SKIN,BRN,BRN,HRT,KID,LNG,MUS,GI,THYM,OVRY,BLD,LIV,BRST,BLD,BRN,LNG	CTCF,POL2,NANOG,TAF1,YY1	BCL,Egr-1,Myc,Nrf1,Rad21,SETDB1,Zfp161,Znf143,p300	FADS1	intronic
11	61829161	0.83	0.95	rs99780	C	T	0.41	0.37	ESC, ESDR, LNG, IPSC, FAT, STRM, BRST, BLD, MUS, BRN, SKIN, VAS, LIV, GI,	ESDR, ESC, LNG, FAT, STRM, BRST, BLD, MUS, BRN, SKIN, LIV, GI, ADRL, HRT, PLCNT, THYM, OVRY, PANC, SPLN, BONE	ESC,ESDR,ESDR,ESC,IPSC,IPSC,BLD,BLD,SKIN,SKIN,SKIN,ADRL,BR	POL2	NRSF,Nanog,Osr,Sin3Ak-20	FADS1	intronic

									ADRL, HRT, KID, PANC, PLCNT, THYM, OVRY, SPLN, CRVX, BONE		N,HRT,GI,KID,LNG,MUS,MUS,PLCNT,GI,THYM,OVRY,PANC,MUS,GI,LNG,BLD,LIV,BRST,MUS,VAS,BLD,SKIN				
11	61830500	0.85	0.96	rs1535	A	G	0.41	0.36	ESC, ESDR, LNG, IPSC, FAT, STRM, BRST, BLD, MUS, BRN, SKIN, VAS, LIV, GI, ADRL, HRT, KID, PLCNT, THYM, SPLN, CRVX	ESC, ESDR, LNG, IPSC, FAT, STRM, BRST, BLD, MUS, BRN, SKIN, VAS, LIV, GI, ADRL, HRT, KID, PANC, PLCNT, THYM, OVRY, SPLN, BONE	SKIN,ADRL,MUS,MUS,OVRY,PANC,MUS	ERALPHA_A	AIRE,ERalpha-a,Nrf1	FADS2	intronic
11	61832870	0.82	-0.93	rs174574	A	C	0.59	0.63	ESDR, ESC, IPSC, BLD, BRN, SKIN, GI, MUS, THYM, SPLN	ESC, ESDR, IPSC, FAT, BLD, STRM, MUS, BRN, SKIN, VAS, LIV, GI, ADRL, HRT, PANC, LNG, PLCNT, THYM, SPLN	IPSC,BLD,BLD,HRT,GI,PANC,GI	POL2	Gfi1,Ik-1,PU.1,RBP-Jkappa	FADS2	intronic
11	65552515	1	1	rs4099470	C	T	0.11	0.05	ESC, ESDR, IPSC, BLD, SKIN, LIV, GI, THYM, LNG, PANC, PLCNT, MUS, SPLN, CRVX	ESC, ESDR, LNG, IPSC, BRST, BLD, SKIN, FAT, LIV, BRN, GI, ADRL, HRT, MUS, PLCNT, THYM, PANC, SPLN, CRVX	ESC,ESDR,ESC,IPSC,BLD,BLD,BLD,BLD,SKIN,BRN,HRT,PLCNT,THYM,PANC,CRVX,LIV,BLD		Myc,RXRA,SIX5,TFE	LTBP3	intronic
11	65556805	1	1	rs11227223	C	T	0.11	0.05	ESDR, ESC, LNG, IPSC, FAT, STRM, BRST, BLD, MUS, BRN, SKIN, VAS, LIV, GI, HRT, KID, PLCNT, THYM, PANC, SPLN, CRVX, BONE	ESC, ESDR, LNG, IPSC, FAT, STRM, BRST, BLD, MUS, BRN, SKIN, VAS, LIV, GI, ADRL, HRT, PLCNT, THYM, OVRY, PANC, SPLN, CRVX, BONE	BLD,BLD,MUS,LNG		Bsx,CHX10,Dlx2,Dlx5,Gbx2,Hlxb9,Hoxa5,Pdx1,RORalpha1,THAP1,YY1	LTBP3	intronic
11	65560184	1	1	rs12224858	G	A	0.10	0.05	ESC, ESDR, IPSC, FAT, STRM, BRST, BLD, MUS, BRN, SKIN, GI, LNG, PLCNT, SPLN, CRVX, LIV, VAS, BONE	ESC, ESDR, LNG, IPSC, FAT, STRM, BRST, BLD, MUS, BRN, SKIN, VAS, LIV, GI, ADRL, HRT, KID, PLCNT, THYM, OVRY, PANC, SPLN, CRVX, BONE	ESC,ESDR,ESDR,ESDR,LNG,IPSC,IPSC,BRST,BLD,SKIN,SKIN,SKIN,ADRL,HRT,GI,MUS,M	GR,USF1,POL24H8,AP2GAMMA,CJUN,JUND,FOSL2	LUN-1	1.3kb 5' of LTBP3	

											US,PLCNT,GI,OVRY,PANC,MUS,GI,LNG,CRVX,LIV,BRST,MUS,MUS,VAS,BLD,BRN,SKIN,SKIN,LNG				
11	65563039	1	1	rs11227226	C	T	0.10	0.05	BLD, SKIN, GI, SPLN, CRVX, BRN	ESDR, IPSC, ESC, BRST, BLD, SKIN, LIV, MUS, PLCNT, THYM, HRT, PANC, GI, SPLN, LNG, CRVX, BRN	ESDR,IPSC,PLCNT,OVRY,MUS		BHLHE40,ELF1,Egr-1,Ets,HEN1,NRSF,YY1,Zfp161	4.1kb 5' of LTBP3	
11	65571473	0.94	1	rs2236684	T	G	0.10	0.05	ESC, ESDR, LNG, IPSC, FAT, STRM, BRST, BLD, MUS, BRN, SKIN, LIV, GI, ADRL, HRT, KID, PANC, PLCNT, THYM, SPLN, CRVX, VAS, BONE	ESC, ESDR, LNG, IPSC, FAT, STRM, BRST, BLD, MUS, BRN, SKIN, LIV, GI, ADRL, HRT, KID, PANC, PLCNT, THYM, SPLN, CRVX, VAS, BONE	BLD,BLD,BLD,BLD,OVRY,MUS,BLD,BLD		BCL,EBF,ELF1,HEY1,YY1	SSSCA1	synonymous
11	65571907	1	1	rs11227227	G	A	0.10	0.05	ESC, ESDR, LNG, IPSC, FAT, STRM, BRST, BLD, MUS, BRN, SKIN, LIV, GI, ADRL, HRT, KID, PANC, PLCNT, THYM, SPLN, CRVX, VAS, BONE	ESC, ESDR, LNG, IPSC, FAT, STRM, BRST, BLD, MUS, BRN, SKIN, LIV, GI, ADRL, HRT, KID, PANC, PLCNT, THYM, SPLN, CRVX, VAS, BONE	ESC,ESDR,ESDR,ESC,BLD,BLD,BLD,HRT,MUS,THYM,OVRY,MUS,LNG,BLD,CRVX	CTCF		SSSCA1	
11	65572664	0.83	1	rs10896017	C	T	0.11	0.06	ESC, ESDR, LNG, IPSC, FAT, STRM, BRST, BLD, MUS, BRN, SKIN, VAS, LIV, GI, ADRL, HRT, KID, PANC, PLCNT, THYM, OVRY, SPLN, CRVX, BONE	ESC, ESDR, IPSC, FAT, BRST, BLD, STRM, BRN, SKIN, LIV, GI, ADRL, HRT, LNG, MUS, PLCNT, THYM, PANC, SPLN, CRVX, VAS, BONE	ESDR,LNG,BLD,BLD,BLD,BLD,BLD,SKIN,SKIN,ADRL,BRN,HRT,GI,KID,LNG,MUS,MUS,PLCNT,GI,THYM,OVRY,GI,BLD,BRST,BLD,SKI	POL2,TCF4,CCNT2,HMGN3,YY1	BCL,BHLHE40,E2F,ELF1,Ets,HEY1,NRSF,Nrf1,SRF,YY1,Znf143,p300	FAM89B	5'-UTR

											N				
4	158730729	0.82	0.93	rs13151920	G	A	0.07	0.38		BLD, LIV, BRN, MUS			CEBPA,CEBPB,CIZ,DMRT2,Pax-4,VDR	7.3kb 5' of PPID	
4	158733660	0.81	0.95	rs13150139	A	G	0.06	0.35					GR	10kb 5' of PPID	
4	158738954	0.93	0.98	rs62351219	C	T	0.07	0.36		IPSC, BRN	BRN,BRN		Nkx3	16kb 5' of PPID	
4	158739503	0.93	0.98	rs34460746	C	G	0.07	0.36		ESC, IPSC, BRN			HEY1,Ik-1,UF1H3BETA,ZBTB7A,Zfp740	16kb 5' of PPID	
4	158740771	0.92	0.99	rs112819353	T	TATAC	0.07	0.35		ESDR, BRN			Foxp1	17kb 5' of PPID	
4	158742999	0.94	0.98	rs34897727	T	C	0.07	0.36		VAS, PLCNT			HDAC2,NRSF,Nkx3	20kb 5' of PPID	
4	158743477	0.92	0.96	rs28415143	C	T	0.07	0.37					CEBPB,SREBP	20kb 5' of PPID	
4	158745961	0.8	0.95	rs6858519	T	C	0.07	0.34					LUN-1,Nkx2,TAL1	23kb 5' of PPID	
4	158748430	0.96	0.99	rs35069181	CA	C	0.07	0.36		STRM, BLD, BONE			GR	21kb 5' of FNIP2	
4	158748712	0.84	0.99	rs3924237	G	A	0.04	0.33	ESC	STRM, BLD, BONE			Hand1,Smad	20kb 5' of FNIP2	
4	158752862	0.94	0.98	rs34033556	G	C	0.07	0.37		LNG, FAT, STRM, MUS, BONE			GR,HNF1,LUN-1	16kb 5' of FNIP2	
4	158756560	0.93	0.97	rs7666007	G	A	0.07	0.37		MUS				13kb 5' of FNIP2	
4	158756709	0.82	0.99	rs11148919	C	CTA	0.06	0.33		MUS			Arid5a,DMRT2,Foxl	12kb 5' of	

				6		TCA ATA							1	FNIP2	
4	158757411	0.97	0.99	rs13120341	T	C	0.07	0.37		BRST, FAT, MUS				12kb 5' of FNIP2	
4	158758422	0.93	0.97	rs2069052	C	T	0.07	0.37		FAT, STRM, BRST, MUS, LIV, PLCNT, BONE	MUS,MUS,BRN,LNG	CEBPB		11kb 5' of FNIP2	
4	158760407	0.95	0.98	rs13114888	C	T	0.07	0.37		BONE			STAT	8.7kb 5' of FNIP2	
4	158762105	0.97	0.99	rs62351226	C	T	0.07	0.37	BRN	HRT			Mtf1,Zbtb3	7kb 5' of FNIP2	
4	158766232	0.95	0.98	rs35350460	A	G	0.07	0.37	BLD, BRN	BLD, LIV, BRN, HRT			Cdx,Foxp1	2.9kb 5' of FNIP2	
4	158766608	0.97	0.99	rs36029896	A	G	0.07	0.37	IPSC, BLD, BRN, GI, HRT	ESDR, IPSC, BLD, VAS, LIV, BRN, GI, HRT, PANC, PLCNT, THYM	BLD,BLD,MUS,BLD,BLD		ATF2,CEBPB,E4BP4,Foxp1,Gmeb1,Hoxa5	2.5kb 5' of FNIP2	
4	158768071	0.96	0.99	rs13133730	A	G	0.07	0.37	ESC, ESDR, LNG, IPSC, FAT, BRST, BLD, STRM, BRN, SKIN, GI, HRT, KID, PANC, MUS, OVRY, PLCNT, THYM, LIV, VAS, BONE	ESC, ESDR, LNG, IPSC, FAT, STRM, BRST, BLD, BRN, SKIN, VAS, LIV, GI, ADRL, HRT, PANC, MUS, PLCNT, THYM, OVRY, SPLN, BONE	BLD,GI		Nkx2	1.1kb 5' of FNIP2	
4	158769470	0.98	0.99	rs55960593	C	T	0.07	0.37	ESC, ESDR, LNG, IPSC, FAT, STRM, BRST, BLD, MUS, BRN, SKIN, VAS, LIV, GI, ADRL, HRT, KID, PANC, PLCNT, THYM, OVRY, SPLN, CRVX, BONE	ESDR, ESC, BRST, BLD, STRM, BRN, SKIN, FAT, VAS, GI, MUS, PLCNT, THYM, PANC, HRT, SPLN	ESDR,IPSC,BRST,BLD,BLD,BLD,BLD,BLD,SKIN,SKIN,ADRL,BRN,HRT,KID,LNG,MUS,MUS,PLCNT,GI,THYM,GI,MUS,LIV,BRST,BLD,B	E2F6,POL2	HIF1,HIF1::Arnt	FNIP2	intronic

											RN			
4	158769488	0.98	0.99	rs59971723	C	A	0.07	0.37	ESC, ESDR, LNG, IPSC, FAT, STRM, BRST, BLD, MUS, BRN, SKIN, VAS, LIV, GI, ADRL, HRT, KID, PANC, PLCNT, THYM, OVRY, SPLN, CRVX, BONE	ESDR, ESC, BRST, BLD, STRM, BRN, SKIN, FAT, VAS, GI, MUS, PLCNT, THYM, PANC, HRT, SPLN	ESDR,ESC,BRST,BLD, BLD,BLD,BLD,BLD,BL D,SKIN,SKIN,SKIN,AD RL,BRN,HRT,GI,KID,L NG,MUS,MUS,PLCNT ,GI,THYM,GI,MUS,LIV ,BRST,MUS,BLD,BRN, SKIN	E2F6,POL2	AP-1,Spdef	FNIP2	intronic
4	158770991	0.96	0.99	rs13116697	A	G	0.07	0.37	ESC, ESDR, LNG, FAT, STRM, BRST, BLD, MUS, BRN, SKIN, VAS, LIV, GI, HRT, PANC, PLCNT, THYM, OVRY, BONE	ESC, ESDR, LNG, IPSC, FAT, STRM, BRST, BLD, MUS, BRN, SKIN, VAS, LIV, GI, ADRL, HRT, PANC, PLCNT, THYM, OVRY, SPLN, BONE	SKIN,LNG		DMRT7,FAC1,Mef2, THAP1,Zfp105	FNIP2	intronic
4	158776184	0.96	0.99	rs6852508	A	T	0.07	0.37	FAT, STRM, BLD, MUS, BRN, GI, HRT, BONE	ESC, ESDR, LNG, IPSC, FAT, STRM, BRST, BLD, MUS, BRN, SKIN, VAS, LIV, GI, ADRL, HRT, PANC, PLCNT, SPLN, BONE			p300	FNIP2	intronic
4	158776407	0.96	0.99	rs6853442	G	T	0.07	0.37	FAT, BLD, STRM, MUS, BRN, GI, HRT	ESC, ESDR, LNG, IPSC, FAT, STRM, BRST, BLD, MUS, BRN, SKIN, VAS, LIV, GI, ADRL, HRT, PANC, OVRY, PLCNT, SPLN			Eomes,HNF6,Hand1	FNIP2	intronic
4	158776594	0.94	0.98	rs10003054	T	A	0.07	0.37	BLD, STRM, BRN, PANC, MUS, GI	ESC, ESDR, LNG, IPSC, FAT, STRM, BRST, BLD, MUS, BRN, SKIN, LIV, GI, ADRL, HRT, PANC, OVRY, PLCNT, VAS			DMRT2,DMRT3,DM RT4,Dobox4,Mef2	FNIP2	intronic
4	158776796	0.96	0.99	rs7695844	G	A	0.07	0.37	BLD, STRM, BRN, PANC, MUS, GI	ESC, ESDR, IPSC, FAT, STRM, BLD, MUS, BRN, SKIN, LIV, GI, ADRL, HRT, PANC, LNG, OVRY, PLCNT, VAS			Bbx,Hbp1,Pou1f1,P ou3f2,Pou3f4,Sox	FNIP2	intronic
4	158776874	0.96	0.99	rs4493489	T	C	0.07	0.37	BLD, STRM, BRN, PANC, MUS, GI	ESC, ESDR, IPSC, FAT, STRM, BLD, MUS, BRN, SKIN, LIV, GI, ADRL, HRT, PANC, LNG, PLCNT, VAS			Arid5a,DMRT1,DMR T7,STAT	FNIP2	intronic
4	158776934	0.96	0.99	rs4331733	A	G	0.07	0.37	BLD, STRM, BRN, PANC, MUS, GI	ESC, ESDR, IPSC, FAT, STRM, BLD, MUS, BRN, SKIN, LIV, GI, ADRL, HRT, PANC, LNG, PLCNT, VAS				FNIP2	intronic
4	158777633	0.96	0.99	rs12171338	C	T	0.07	0.37	FAT, BLD, STRM, BRN, GI,	ESC, ESDR, LNG, IPSC, FAT, STRM, BRST, BLD, MUS, BRN,	GI,GI,MUS,LIV	GR,FOXA1,P300	ATF3,CAC-binding-p	FNIP2	intronic

									PANC, MUS	SKIN, VAS, LIV, GI, ADRL, HRT, PANC, PLCNT, THYM, SPLN, BONE			rotein,CACD,CTCF,E2F,Egr-1,Ets,RXRA,Rad21,SMC3,Sp4,YY1,Zfp740		
4	158778524	0.96	0.99	rs11723981	G	A	0.07	0.37	FAT, BLD, BRN, GI, PANC, MUS	ESC, ESDR, LNG, IPSC, FAT, STRM, BRST, BLD, MUS, BRN, SKIN, VAS, LIV, GI, ADRL, HRT, PANC, THYM, BONE	BLD		Sox	FNIP2	intronic
4	158778908	0.96	0.99	rs13123982	G	A	0.07	0.37	BLD, BRN, GI, MUS	ESC, ESDR, LNG, IPSC, FAT, STRM, BRST, BLD, MUS, BRN, SKIN, VAS, LIV, GI, ADRL, HRT, PANC, THYM, BONE	GI,GI,LNG,MUS,MUS,GI,GI,PANC,MUS,GI,MUS,MUS,BRN,LNG		GATA	FNIP2	intronic
4	158781154	0.96	0.99	rs72695428	A	C	0.07	0.37	ESC, ESDR, LIV, GI	IPSC, BRST, BLD, SKIN, VAS, LIV, BRN, GI, HRT, PANC, LNG, MUS			Osr,Pou2f2	FNIP2	intronic
4	158782073	0.96	0.99	rs6829398	T	A	0.07	0.37	ESC, ESDR, FAT, STRM, MUS, LIV, BRN, HRT, GI, BONE	ESDR, ESC, LNG, IPSC, FAT, STRM, BRST, BLD, MUS, BRN, SKIN, VAS, LIV, GI, ADRL, HRT, KID, PANC, THYM, OVRY, SPLN, BONE	LNG,BLD	CTCF,RAD21	Myf,NRSF,PRDM1,SZF1-1,Spz1	FNIP2	intronic
4	158783506	0.98	0.99	rs35723165	G	C	0.07	0.37	ESC, ESDR, FAT, STRM, BLD, MUS, SKIN, VAS, LIV, BRN, GI, HRT, LNG, BONE	ESC, ESDR, LNG, IPSC, FAT, STRM, BRST, BLD, MUS, BRN, SKIN, VAS, LIV, GI, ADRL, HRT, KID, PANC, THYM, OVRY, PLCNT, SPLN, BONE			Cdx2,Hoxa10,Hoxb8,Hoxc9,Pax-5	FNIP2	intronic
4	158784688	0.96	0.99	rs11933662	G	A	0.07	0.37	ESDR, FAT, BLD, MUS, LIV, BRN, GI, LNG, BONE	ESDR, ESC, LNG, FAT, STRM, BRST, BLD, MUS, BRN, SKIN, VAS, LIV, GI, ADRL, HRT, KID, PANC, THYM, OVRY, SPLN, BONE			BDP1	FNIP2	intronic
4	158784715	0.96	0.99	rs11942564	A	G	0.07	0.37	ESDR, BLD, MUS, LIV, BRN, GI, LNG, BONE	ESDR, ESC, LNG, FAT, STRM, BRST, BLD, MUS, BRN, SKIN, VAS, LIV, GI, ADRL, HRT, KID, PANC, THYM, OVRY, SPLN, BONE				FNIP2	intronic
4	158784834	0.96	0.99	rs9884355	G	C	0.07	0.37	ESDR, BLD, MUS, LIV, BRN, GI, LNG	ESC, ESDR, LNG, FAT, STRM, BRST, BLD, MUS, BRN, SKIN, VAS, LIV, GI, ADRL, HRT, PANC, THYM, OVRY, SPLN, BONE			CCNT2	FNIP2	intronic
4	158784969	0.98	0.99	rs4538434	C	G	0.07	0.37	BLD, MUS, LIV, BRN, GI,	ESC, ESDR, LNG, FAT, STRM, BRST, BLD, MUS, BRN, SKIN,	BLD		GR	FNIP2	intronic

									LNG	VAS, LIV, GI, ADRL, HRT, PANC, THYM, OVRY, SPLN, BONE					
4	158785155	0.96	0.99	rs9884798	A	G	0.07	0.37	BLD, LIV, BRN, GI, LNG	ESC, ESDR, LNG, FAT, STRM, BRST, BLD, MUS, BRN, SKIN, VAS, LIV, GI, ADRL, HRT, PANC, THYM, SPLN, BONE			TCF12	FNIP2	intronic
4	158787008	0.95	0.99	rs4434197	C	T	0.06	0.37	FAT, ESC, STRM, BLD, VAS, LIV, BRN, GI, HRT, LNG, MUS	ESC, ESDR, LNG, IPSC, FAT, STRM, BRST, BLD, MUS, BRN, SKIN, VAS, LIV, GI, ADRL, HRT, KID, PANC, THYM, BONE	HRT,MUS,MUS		CIZ,Gfi1,Mef2	FNIP2	intronic
4	158787024	0.95	0.98	rs4328866	G	C	0.06	0.37	FAT, ESC, STRM, BLD, VAS, LIV, BRN, GI, HRT, LNG, MUS	ESC, ESDR, LNG, IPSC, FAT, STRM, BRST, BLD, MUS, BRN, SKIN, VAS, LIV, GI, ADRL, HRT, KID, PANC, THYM, BONE	HRT,MUS,MUS			FNIP2	intronic
4	158791118	0.9	0.99	rs139202522	GA	G	0.04	0.35		ESC, ESDR, LNG, IPSC, FAT, BRST, BLD, SKIN, LIV, BRN, GI, HRT, PANC, MUS			Ets,Foxa,Foxl1,GATA,HMG-IY,Hoxb8,STAT,TAL1	FNIP2	intronic
4	158794329	0.98	0.99	rs35237848	T	A	0.07	0.37		BRST, SKIN, LIV, BRN, GI, HRT, PANC, OVRY, MUS			Dbx1,Foxp3,Zfp105	FNIP2	intronic
4	158796406	0.98	0.99	rs143826465	CTTA	C	0.07	0.37	FAT, MUS, LIV, BRN	ESC, ESDR, LNG, IPSC, FAT, BRST, STRM, MUS, SKIN, VAS, LIV, BRN, GI, HRT, PANC, BLD, BONE	MUS		GR,MIF-1,Sox	FNIP2	intronic
4	158800615	0.98	0.99	rs35185176	T	C	0.07	0.37	ESDR, LIV, HRT, GI	ESC, ESDR, LNG, IPSC, FAT, BLD, BRN, SKIN, LIV, GI, HRT, PANC, PLCNT, MUS			Bcl6b,Mef2,Pou2f2	FNIP2	intronic
4	158805132	0.99	0.99	rs35367187	G	A	0.07	0.37	BRN, HRT, BLD	ESC, ESDR, IPSC, FAT, STRM, BRST, BLD, BRN, SKIN, VAS, LIV, GI, HRT, PANC, PLCNT, MUS			Barx2,Hoxa5,Hoxb6,Lhx4,Rhox11	FNIP2	intronic
4	158805913	0.99	0.99	rs35906938	A	G	0.07	0.37	BLD, SKIN, BRN, GI, HRT, MUS	ESC, ESDR, IPSC, FAT, STRM, BRST, BLD, BRN, SKIN, VAS, LIV, GI, ADRL, HRT, PANC, LNG, MUS, PLCNT, THYM	ESDR,BRST,BLD,SKIN,SKIN,BRN,BRN,HRT,GI,GI,PLCNT,GI,CRVX,BRST,BLD,SKIN	KAP1	GR,Sox	FNIP2	intronic
4	158808858	0.99	0.99	rs4482713	A	C	0.07	0.37	FAT, STRM, BLD, SKIN, LIV, BRN, GI, HRT, MUS, BONE	ESDR, LNG, FAT, STRM, BRST, BLD, MUS, SKIN, VAS, LIV, BRN, GI, ADRL, HRT, KID, PANC, PLCNT, THYM, CRVX, BONE	HRT,GI,BLD		p300	FNIP2	intronic
4	158809191	0.97	0.99	rs13124267	G	A	0.07	0.36	FAT, STRM, BLD, SKIN, LIV,	ESDR, LNG, FAT, STRM, BRST, BLD, MUS, SKIN, VAS, LIV,			Pax-4	FNIP2	intronic

									BRN, GI, HRT, MUS, BONE	BRN, GI, ADRL, HRT, KID, PANC, PLCNT, THYM, CRVX, BONE					
4	158811622	1	1	rs13152601	G	A	0.07	0.37	FAT, STRM, BLD, MUS, LIV, BRN, GI, HRT, PANC, BONE	ESC, ESDR, LNG, IPSC, FAT, STRM, BRST, BLD, MUS, SKIN, VAS, LIV, BRN, GI, ADRL, HRT, KID, PANC, PLCNT, THYM, SPLN, CRVX, BONE	BLD,MUS		Bcl6b,EBF,STAT	FNIP2	intronic
4	158812089	1	1	rs13106780	C	G	0.07	0.37	BLD, FAT, LIV, BRN, GI, HRT, PANC, THYM, MUS, SKIN, BONE	ESDR, ESC, LNG, IPSC, FAT, STRM, BRST, BLD, MUS, SKIN, VAS, LIV, BRN, GI, ADRL, HRT, KID, PANC, PLCNT, THYM, SPLN, CRVX, BONE	BLD,BLD,BLD,SKIN,SKIN,HRT,GI,BLD,BLD		COMP1	FNIP2	intronic
4	158815960	1	1	rs4131480	A	G	0.07	0.37	HRT	STRM, BLD, SKIN, VAS, LIV, BRN, GI, HRT, PANC, MUS			Tgif1	FNIP2	intronic
4	158819398	0.96	1	rs144146694	AATTAAT	A	0.07	0.36	FAT, STRM, BLD, SKIN, LIV, BRN, GI, HRT, MUS, BONE	ESC, ESDR, LNG, IPSC, FAT, STRM, BRST, BLD, SKIN, LIV, BRN, GI, ADRL, HRT, KID, PANC, PLCNT, OVRY, MUS, VAS, BONE			Arid5a,CDP,Dbx1,Evi-1,Foxd3,Foxj2,GATA,HNF1,Hoxd10,Hoxd8,Ncx,PLZF,Pou2f2,Pou3f2,Pou3f3,TATA	FNIP2	intronic
4	158820280	1	1	rs35563827	T	G	0.07	0.37	FAT, STRM, BLD, SKIN, LIV, BRN, GI, HRT, MUS, LNG, BONE	ESC, ESDR, LNG, IPSC, FAT, STRM, BRST, BLD, MUS, SKIN, VAS, LIV, BRN, GI, ADRL, HRT, KID, PANC, OVRY, BONE	ESDR,SKIN,GI,GI,KID,LNG,MUS,PLCNT,GI,PANC,LIV,VAS,SKIN,LNG	HNF4A,P300,SP1,TCF4	Ik-2,NF-AT1	FNIP2	intronic
4	158822034	1	1	rs2881547	A	G	0.07	0.37	FAT, STRM, BLD, BRN, GI, HRT, MUS	ESC, ESDR, LNG, IPSC, FAT, STRM, BRST, BLD, MUS, SKIN, VAS, LIV, BRN, GI, ADRL, HRT, KID, PANC, PLCNT, BONE			ATF3,GR,TCF12,YY1	FNIP2	intronic
4	158824017	1	1	rs13103926	C	T	0.07	0.37	ESDR, FAT, BLD, STRM, BRN, GI, HRT, MUS, SPLN	ESC, ESDR, LNG, IPSC, FAT, STRM, BRST, BLD, MUS, SKIN, VAS, LIV, BRN, GI, ADRL, HRT, KID, PANC, PLCNT, THYM, OVRY, SPLN, BONE			Cdx,FAC1,Gfi1b,Ik-2,PLZF	FNIP2	intronic
4	158829903	1	1	rs11728672	A	G	0.07	0.37	GI	BLD, SKIN, BRN, GI, HRT, MUS			Foxo,STAT,Sox	FNIP2	intronic
4	158830001	1	1	rs11728711	A	T	0.07	0.37	GI	BLD, SKIN, BRN, GI, HRT, MUS			Pax-5,RXRA,Zfp691	FNIP2	intronic
4	158831513	1	1	rs10517696	A	G	0.06	0.37	GI	SKIN, BRN, GI			EBF,NRSF,Sin3Ak-20	FNIP2	intronic

4	158832770	1	1	rs2347078	T	G	0.07	0.37		ESDR, SKIN, LIV, BRN, PANC, GI			Dbx1,Evi-1,Foxd3,Foxi1,Foxj1,Foxj2,Foxk1,Foxl1,Foxo,Foxp1,HDAC2,HNF1,Hoxb13,Ncx,Nkx6-2,Sox,TATA,Zfp105	FNIP2	intronic
4	158837764	1	1	rs60547563	G	A	0.07	0.37	ESDR, BRN	ESDR, ESC, IPSC, BLD, LIV, BRN, GI, HRT, OVRY, PANC, MUS	GI,GI		AP-1,CEBPB,Mef2	FNIP2	intronic
4	158843710	0.98	0.99	rs35422904	T	C	0.07	0.37	HRT	ESC, IPSC, LIV, BRN, HRT				FNIP2	intronic
4	158844132	0.99	0.99	rs66633017	G	C	0.07	0.37	IPSC, HRT	ESC, IPSC, SKIN, LIV, BRN, HRT, GI			PLZF,Sox	FNIP2	intronic
4	158845445	0.99	0.99	rs34293068	T	C	0.07	0.37	ESC, IPSC, HRT	ESC, ESDR, LNG, IPSC, SKIN, LIV, BRN, HRT, GI, PANC	ESDR,ESC,IPSC,IPSC	POU5F1	GR	FNIP2	intronic
4	158862497	0.99	0.99	rs34083477	A	C	0.07	0.37	GI, BLD	SKIN, BRN, GI, PANC, BLD			Crx,EWSR1-FLI1,Foxm1,Nkx2,Pou2f2,STAT	FNIP2	intronic
4	158863810	0.99	0.99	rs36023021	TC	T	0.06	0.37		SKIN, BRN, PANC			Nkx2,Pdx1,RORalpha1	FNIP2	intronic
4	158867453	0.98	0.99	rs35322790	A	G	0.07	0.37	GI, SPLN	ESDR, BRST, SKIN, BRN, GI, PANC, MUS, HRT, OVRY			EBF,Ik-1,Maf,NRSF,SETDB1,SP1	FNIP2	intronic
4	158867848	0.99	0.99	rs11726096	A	C	0.07	0.37	GI, SPLN	ESDR, IPSC, BRST, SKIN, BRN, GI, PANC, MUS, HRT, OVRY			AP-2,BDP1,BHLHE40,CACD,CTCF,GR,Klf7,Myc,NRSF,Rad21,SMC3,SP1,Znf143,p300	FNIP2	intronic
4	158871478	0.99	0.99	rs17286116	C	T	0.06	0.37	BLD, GI, SPLN	ESC, ESDR, LNG, IPSC, FAT, STRM, BRST, BLD, MUS, SKIN, VAS, LIV, BRN, GI, ADRL, KID, PANC, THYM, HRT, OVRY, SPLN, BONE	SKIN,ADRL,OVRY		Foxa,Foxq1	FNIP2	intronic
4	158872731	0.94	0.99	rs20151378	A	AGT	0.06	0.36		ESDR, ESC, LNG, FAT, STRM, BRST, BLD, MUS, SKIN, LIV,			FAC1,Foxa,Foxp1,H	FNIP2	intronic

				2						BRN, GI, KID, PANC, PLCNT, HRT, VAS, BONE			DAC2,Irf,Pax-4,TATA,Zfp105,p300		
4	158872735	0.94	0.99	rs200002419	T	TG	0.06	0.36		ESDR, ESC, LNG, FAT, STRM, BRST, BLD, MUS, SKIN, LIV, BRN, GI, KID, PANC, PLCNT, HRT, VAS, BONE			FAC1,Foxa,Foxp1,HDAC2,Pax-4,Pou2f2,TATA,Zfp105,p300	FNIP2	intronic
4	158872736	0.86	0.99	rs2291008	T	G	0.06	0.34		ESDR, ESC, LNG, FAT, STRM, BRST, BLD, MUS, SKIN, LIV, BRN, GI, KID, PANC, PLCNT, HRT, VAS, BONE			FAC1,Foxa,Foxp1,HDAC2,Pax-4,Zfp105,p300	FNIP2	intronic
4	158874579	0.99	0.99	rs34680567	A	G	0.07	0.37	LIV, BRN	ESC, ESDR, LNG, FAT, STRM, BRST, BLD, MUS, SKIN, VAS, LIV, BRN, GI, ADRL, HRT, PLCNT, OVRY, PANC, CRVX, BONE	BRST		Hand1,LBP-9,RP58,TAL1	FNIP2	intronic
4	158875091	0.89	0.97	rs13143603	A	G	0.07	0.35	LIV, BRN	ESC, ESDR, STRM, BRST, BLD, SKIN, VAS, LIV, BRN, GI, ADRL, HRT, PLCNT, OVRY, PANC, MUS, LNG			Foxp1,HDAC2,Irf,Zfp105,p300	FNIP2	intronic
4	158875159	0.99	0.99	rs13143943	C	G	0.07	0.37	LIV, BRN	ESC, ESDR, STRM, BRST, BLD, SKIN, VAS, LIV, BRN, GI, ADRL, HRT, PLCNT, OVRY, PANC, MUS, LNG	BRN		Evi-1,FXR,Nanog,SP2	FNIP2	intronic
4	158875412	0.99	0.99	rs36024718	C	T	0.07	0.37	LIV	ESDR, STRM, BRST, BLD, SKIN, VAS, LIV, BRN, HRT, GI, PANC, MUS, LNG	BRN,BRN		DMRT5,Osr	FNIP2	intronic
4	158877386	0.99	0.99	rs4619844	A	C	0.07	0.37	HRT, GI	BRST, LIV, HRT, PANC, GI, MUS, BLD			Pou1f1,Znf143	FNIP2	intronic
4	158878790	0.98	0.99	rs13115760	C	T	0.06	0.37	HRT	BRST, GI			Foxd1,Foxf2,Foxj2,Foxl1,Foxo,GATA,Hoxa9,Pou2f2,YY1	FNIP2	intronic
4	158880139	0.9	0.97	rs13129071	C	G	0.06	0.38					XBP-1,p53	FNIP2	intronic
4	158880959	0.99	0.99	rs13148521	A	G	0.06	0.37			BLD		CEBPB,Pax-5	FNIP2	intronic
4	158882197	0.81	0.99	rs149170475	C	G	0.10	0.33		GI			BCL,BDP1,BHLHE40,CTCF,E2F,ETF,Rad21,TCF12,YY1,p300	FNIP2	intronic
4	158882568	0.96	0.98	rs34198529	C	T	0.06	0.37		HRT			Maf,TAL1,VDR	FNIP2	intronic

4	158884218	0.99	0.99	rs35747642	T	G	0.06	0.37	GI	FAT, BRST, SKIN, VAS, BRN, GI, ADRL, HRT, LNG, OVRY, MUS	BRN,LNG,SKIN,LNG		AP-1,ATF4,Isl2,Nkx3,Nkx6-1,OTX	FNIP2	intronic
4	158888830	0.99	0.99	rs35613171	C	T	0.07	0.37		ESDR, BRST, VAS, LIV, PLCNT, LNG				FNIP2	intronic
4	158891247	0.99	0.99	rs11726311	C	A	0.06	0.37	GI	ESC, ESDR, IPSC, VAS, LIV, GI			Dobox4,HP1-site-factor,TATA	FNIP2	intronic
4	158898104	0.86	0.99	rs146866082	C	T	0.04	0.34		PANC			AP-2rep,GATA,MZF1::1-4	FNIP2	intronic
4	158901634	0.97	0.98	rs146564509	G	A	0.06	0.37		ESC, IPSC, HRT, LIV, BONE			Foxd3,Gfi1,Pbx-1	FNIP2	intronic
4	158902419	0.95	0.98	rs115849351	C	T	0.06	0.36	ESC, ESDR, GI, SPLN, LNG	ESC, ESDR, LNG, IPSC, STRM, BRST, BLD, MUS, SKIN, VAS, LIV, BRN, GI, ADRL, HRT, PLCNT, THYM, OVRY, PANC, SPLN, CRVX, BONE			BRCA1,CTCF,RXRA,Rad21	FNIP2	intronic
4	158905227	0.97	0.98	rs2279153	T	C	0.06	0.37		ESC, ESDR, FAT, BLD, SKIN, GI, LNG, LIV	BLD		Foxd3,Foxm1,Pbx3	FNIP2	3'-UTR
4	158906161	0.97	0.98	rs10589	T	C	0.06	0.37		ESDR, BLD, GI, LNG	THYM,BLD		MIF-1,RFX5	FNIP2	3'-UTR
4	158910245	0.96	-0.98	rs6536374	T	C	0.94	0.63		SKIN, VAS			Dmbx1,GATA,HNF1,Hoxa7	C4orf45	intronic
4	158911575	0.95	-0.98	rs202115158	T	TCC	0.93	0.63	FAT	ESC, ESDR, IPSC, FAT, STRM, SKIN, VAS, BRN, GI, SPLN, MUS, LNG, BONE			MAZR,MZF1::1-4	C4orf45	intronic
4	158911903	0.95	-0.98	rs4691522	A	G	0.94	0.63	FAT	ESC, ESDR, IPSC, FAT, STRM, SKIN, BRN, GI, MUS, LNG, BONE	SKIN		Cdx,Evi-1,Foxp1,Hlx1,Pou4f3,Pou6f1,STAT,Sox,TATA,p300	C4orf45	intronic
4	158913005	0.95	-0.98	rs7698561	G	A	0.94	0.63	FAT	ESC, ESDR, LNG, FAT, STRM, MUS, SKIN, VAS, BRN, THYM, GI, HRT, BONE	LNG,SKIN,SKIN,MUS,MUS,BRN,SKIN,LNG		AP-1,Foxd1,Pax-5,Pax-6,Pax-8	C4orf45	intronic
4	158913988	0.95	-0.98	rs9998057	C	A	0.94	0.63	FAT, STRM, SKIN, BONE	ESDR, LNG, FAT, STRM, MUS, BRN, SKIN, VAS, GI, PLCNT, BLD, BONE	SKIN,SKIN,BRN,SKIN		GR	C4orf45	intronic
4	158914260	0.94	-0.97	rs9998332	C	T	0.94	0.63	ESC, FAT, STRM, SKIN,	ESC, ESDR, LNG, FAT, STRM, BRST, MUS, BRN, SKIN, VAS,	SKIN,SKIN,SKIN,SKIN,	GR,CFOS	PPAR,RXRA	C4orf45	intronic

									BONE	GI, PLCNT, HRT, BLD, BONE	MUS,MUS,MUS,MUS,SKIN,LNG				
4	158914341	0.94	-0.97	rs10020775	A	G	0.94	0.63	ESC, ESDR, FAT, STRM, SKIN, BRN, BONE	ESC, ESDR, LNG, FAT, STRM, BRST, MUS, BRN, SKIN, VAS, GI, HRT, PLCNT, BLD, BONE	ESDR,LNG,BRST,BLD,SKIN,SKIN,SKIN,SKIN,ADRL,MUS,MUS,PLCNT,MUS,LNG,BRST,MUS,MUS,VAS,BLD,BRN,SKIN,SKIN,LNG	GR,CFOS	DMRT1,DMRT2,DMRT7	C4orf45	intronic
4	158914546	0.94	-0.97	rs9998816	G	C	0.94	0.63	ESC, ESDR, FAT, STRM, SKIN, HRT, BRN, BONE	ESC, ESDR, LNG, FAT, STRM, BRST, MUS, BRN, SKIN, VAS, GI, ADRL, HRT, PLCNT, BLD, BONE	ESDR,SKIN,SKIN,HRT,MUS,MUS,MUS,BRN,SKIN,LNG	CFOS	AFP1,CIZ,HNF4	C4orf45	intronic
4	158914592	0.94	0.97	rs6846784	T	C	0.06	0.37	ESC, ESDR, FAT, STRM, SKIN, HRT, BRN, BONE	ESC, ESDR, LNG, FAT, STRM, BRST, MUS, BRN, SKIN, VAS, GI, ADRL, HRT, PLCNT, BLD, BONE	MUS,MUS,SKIN,LNG		Foxj2,Hoxd8,Mef2,Pax-4	C4orf45	intronic
4	158917317	0.85	-0.95	rs28530562	C	A	0.91	0.62	ESDR, GI	FAT, BRST, SKIN, VAS, LNG, MUS, HRT, GI, SPLN, BONE			LUN-1,Lhx8,Pax-5	C4orf45	intronic
4	158918161	0.93	-0.97	rs10010963	C	T	0.94	0.63	GI	ESC, IPSC, BLD, VAS, LNG	ESC,IPSC		Pax-6,Pou2f2	C4orf45	intronic
4	158918258	0.94	-0.97	rs10011224	G	A	0.94	0.63	GI	ESC, IPSC, BLD, VAS, LNG	ESC,IPSC,IPSC		BDP1,E2F,Egr-1,GR,PU.1,SP1,STAT,Spz1,TATA,VDR,WT1,Znf143,p300	C4orf45	intronic
4	158919325	0.93	-0.97	rs4690916	C	T	0.94	0.63	ESDR				Arnt,Myc,SREBP,XBP-1	C4orf45	intronic
4	158920532	0.93	0.97	rs1820512	G	T	0.06	0.37	ESDR	BLD			Pou5f1,Smad3,Smad	C4orf45	intronic
4	158920640	0.92	0.96	rs1821199	G	A	0.06	0.37	ESDR	BLD	BLD,BLD,PLCNT,BLD		LBP-1,NF-I	C4orf45	intronic
4	158925677	0.93	-0.97	rs4691524	A	C	0.94	0.63	ESC, SKIN				BDP1,ELF1,Egr-1,Ets,Pax-5,SP1	C4orf45	intronic

4	158927285	0.92	-0.96	rs4691525	A	G	0.94	0.63						C4orf45	intronic
4	158927733	0.92	-0.96	rs7668454	A	G	0.94	0.63	ESDR, SPLN				BCL,NRSF,YY1	C4orf45	intronic
19	48700572	0.85	0.98	rs35106244	C	T	0.01	0.40		SKIN, GI			E2F	FUT2	intronic
19	48702851	1	1	rs679574	C	G	0.01	0.43	SKIN, GI	SKIN, GI, SPLN			RREB-1	FUT2	intronic
19	48702888	1	1	rs516316	G	C	0.01	0.43	SKIN, GI	SKIN, GI, SPLN			Myc,Nrf1	FUT2	intronic
19	48702915	1	1	rs516246	C	T	0.01	0.43	SKIN, GI	SKIN, GI, SPLN			AP-2,Egr-1,Ets,GATA,Rad21,TFII-I,Znf143,p300	FUT2	intronic
19	48703160	0.99	1	rs492602	A	G	0.01	0.43	SKIN, GI	SKIN, GI, OVRY, PANC, SPLN			CHOP::CEBPalpha,Znf143	FUT2	synonymous
19	48703205	1	1	rs681343	C	T	0.01	0.43	SKIN, GI	SKIN, GI, OVRY, PANC, SPLN				FUT2	synonymous
19	48703417	1	1	rs601338	G	A	0.01	0.43	SKIN, GI, SPLN	SKIN, GI, OVRY, PANC, SPLN			HDAC2,HNF4	FUT2	nonsense
19	48703728	0.88	1	rs602662	G	A	0.01	0.46	SKIN, GI, SPLN	SKIN, GI, OVRY, PANC, SPLN			RREB-1,SP1	FUT2	missense
19	48703949	0.88	1	rs485186	A	G	0.01	0.46	SKIN, SPLN	ESDR, SKIN, GI, OVRY, SPLN			CEBPB,GATA,SP1	FUT2	synonymous
19	48703998	0.88	1	rs485073	A	G	0.01	0.46	SKIN, SPLN	ESDR, SKIN, GI, OVRY, SPLN			GCM	FUT2	3'-UTR
19	48704000	0.88	1	rs603985	T	C	0.01	0.46	SKIN, SPLN	ESDR, SKIN, GI, OVRY, SPLN			Nr2f2,Sox	FUT2	3'-UTR
19	48704297	0.87	0.99	rs571689	C	T	0.01	0.46	SKIN, SPLN	GI			E2A,Pax-4,ZEB1	FUT2	3'-UTR
19	48704394	0.88	1	rs570794	T	C	0.01	0.46	SKIN, SPLN	GI			TCF11::MafG	FUT2	3'-UTR
19	48704461	0.88	1	rs569970	T	C	0.01	0.46	SKIN, SPLN	GI			BATF,RXRA,Sox,p300	FUT2	3'-UTR
19	48704535	0.88	1	rs2251034	G	A	0.01	0.46	SKIN, SPLN	GI			CDP,Pax-6	FUT2	3'-UTR
19	48705087	0.84	0.96	rs28362844	AC	A	0.01	0.45	SPLN	GI			BCL,Evi-1,Foxa,Foxd1,Foxp1,GATA,HDA	FUT2	3'-UTR

													C2,Irf,Nanog,Pax-5,RXRA,p300		
19	48705244	0.83	0.95	rs507855	A	G	0.01	0.45	SPLN	GI			LUN-1,Pitx2	FUT2	3'-UTR
19	48705286	0.88	1	rs507766	T	C	0.01	0.46	SPLN	GI			Fox,Foxl1,Hoxb13,TATA	FUT2	3'-UTR
19	48705307	0.88	1	rs507711	C	T	0.01	0.46	SPLN	GI			GR,SIX5	FUT2	3'-UTR
19	48705372	0.88	1	rs506897	G	C	0.01	0.46	SPLN	GI			NRSF	FUT2	3'-UTR
19	48705608	0.88	0.99	rs504963	G	A	0.01	0.46	SPLN	GI, LIV	KID		Ets,GR,Hbp1	FUT2	3'-UTR
19	48705721	0.88	0.99	rs632111	A	G	0.01	0.46	SPLN	GI, LIV	SKIN,GI,KID		DMRT2,SPIB,TCF11::MafG,TCF4	FUT2	3'-UTR
19	48705753	0.88	1	rs503279	T	C	0.01	0.46	SPLN	GI, LIV	GI		Gm397,Mtf1,TCF12	FUT2	3'-UTR
19	48705969	0.88	1	rs633372	G	A	0.01	0.46	SPLN	GI	GI		CEBPB	18bp 3' of FUT2	
19	48706068	0.85	0.97	rs2548458	C	T	0.01	0.45	SPLN	GI			ATF3,DEC,LXR,Nkx2,SREBP	117bp 3' of FUT2	
19	48706082	0.85	0.97	rs2548459	T	C	0.01	0.45	SPLN	GI			Nr2f2	131bp 3' of FUT2	
19	48706207	0.88	0.99	rs692854	C	A	0.01	0.46	SPLN	GI	KID		Myc	256bp 3' of FUT2	
19	48706303	0.81	0.92	rs1688264	T	G	0.01	0.44	SPLN	GI			Cdx,Foxd3,HDAC2,Irf,PLZF,Zfp105	352bp 3' of FUT2	
19	48706309	0.81	0.92	rs1704773	A	G	0.01	0.44	SPLN	GI			Foxp1,PLZF	358bp 3' of FUT2	
19	48706594	0.86	0.98	rs646327	A	G	0.01	0.46	SPLN	GI			ATF3,HNF4,SREBP	643bp 3' of FUT2	
19	48708712	0.82	0.97	rs676388	T	C	0.01	0.46	ESDR, IPSC, SKIN, SPLN	ESC, ESDR, IPSC, SKIN, ADRL, PANC, PLCNT, HRT, SPLN, BLD	ESC,ESC,IPSC,IPSC,M	USF1	AhR	2.8kb 3' of	

											US			FUT2	
19	48710027	0.82	0.97	rs584768	G	A	0.01	0.46	SPLN	SKIN, SPLN			CTCF,E2A,Lmo2-complex,Myf,Rad21,TCF12,ZEB1,Zbtb3	3kb 3' of MAMSTR	
19	48710247	0.82	0.97	rs2452170	G	A	0.01	0.46	SPLN	SKIN, GI			E2A,ZEB1	2.8kb 3' of MAMSTR	
19	48710576	0.82	0.97	rs2638282	G	A	0.01	0.46	SPLN	GI, LNG	MUS			2.4kb 3' of MAMSTR	
19	48711017	0.82	0.95	rs281379	G	A	0.01	0.45	ESC, SKIN, SPLN	BLD, GI, LNG	ADRL,MUS		Bach1,Pax-2,Smad3	2kb 3' of MAMSTR	
6	29658377	0.89	0.96	rs149521679	AAAAC	A	0.06	0.04	BLD, BRN, SPLN	ESC, IPSC, BRST, GI, HRT			DMRT2,ERalpha-a,FAC1,Foxa,GATA,HDAC2,PU.1,STAT,Zfp105,p300	MOG	intronic
6	29658599	0.8	0.95	rs9257932	T	C	0.06	0.03	BRN, SPLN	ESC, IPSC, BRST, HRT			Maf,THAP1	MOG	intronic
6	29665998	0.96	1	rs6905408	C	T	0.14	0.04	ESC, ESDR, IPSC, BRST, BRN, HRT	ESC, ESDR, IPSC, BRN, HRT, PLCNT, BLD	ESC,ESDR,ESDR,ESC,IPSC,IPSC,HRT		Ik-2,NF-AT	MOG	intronic
6	29667540	0.96	1	rs2071652	C	T	0.14	0.04	ESC, ESDR, IPSC, BRN, HRT	ESC, ESDR, IPSC, BRST, SKIN, VAS, BRN, HRT, GI, PLCNT, MUS	ESDR,ESC,HRT		RFX5,Znf143	MOG	intronic
6	29674732	1	1	rs9257940	A	G	0.14	0.03	SKIN	ESC, IPSC, BRST, SKIN, BRN, HRT				ZFP57	intronic
6	29679088	1	1	rs9257953	G	A	0.14	0.03	BRST, BLD, SKIN, HRT, PLCNT	ESC, ESDR, IPSC, BRST, BLD, SKIN, HRT, LNG, SPLN, CRVX, VAS	SKIN		PU.1	ZFP57	
6	29684564	0.96	1	rs9257986	C	G	0.14	0.04	ESDR	ESC, ESDR, IPSC, BRST, BLD, LNG, GI			MZF1::1-4	3.5kb 5' of ZFP57	
7	2718707	0.81	0.94	rs798559	T	C	0.20	0.32	ESDR, IPSC, BLD, SKIN, GI, MUS, SPLN	ESC, ESDR, LNG, IPSC, BLD, STRM, SKIN, FAT, BRN, GI, ADRL, HRT, KID, MUS, PLCNT, THYM, PANC, SPLN	BLD,BLD,BLD,BLD,LNG,MUS,GI,OVRY		Irf,PRDM1	AC006028.9	

7	2719301	0.81	0.94	rs798558	T	G	0.21	0.31	ESDR, BLD, SKIN, GI, MUS, SPLN	ESC, ESDR, IPSC, BLD, BRN, SKIN, FAT, HRT, GI, KID, LNG, MUS, PLCNT, THYM, PANC, SPLN	ESC,ESC,IPSC,BLD,BLD,BLD,BLD,BRN,HRT,KID,THYM,LIV,BLD,BRN	CTCF	Hic1	AC006028.9	
7	2719348	0.81	0.94	rs798557	G	A	0.20	0.31	ESDR, BLD, SKIN, GI, MUS, SPLN	ESC, ESDR, IPSC, BLD, BRN, SKIN, HRT, GI, KID, MUS, PLCNT, THYM, LNG, PANC, SPLN	ESC,ESC,IPSC,IPSC,BLD,BLD,BLD,BLD,HRT,KID,THYM,OVRY,BLD			AC006028.9	
7	2719368	0.81	0.94	rs798556	C	A	0.21	0.31	BLD, SKIN, GI, SPLN	ESC, ESDR, IPSC, BLD, BRN, SKIN, HRT, GI, KID, MUS, PLCNT, THYM, LNG, PANC, SPLN	ESC,ESC,BLD,BLD,BLD,BLD,BRN,HRT,KID,OVRY		E2F,Egr-1,HNF4,Nrf1,PPAR,Pax-5,RXR::LXR,RXRA,Zfp161	AC006028.9	
7	2719839	0.81	0.94	rs798555	T	C	0.20	0.31	BLD, GI	ESDR, IPSC, ESC, BLD, SKIN, BRN, HRT, KID, MUS, GI, THYM, LNG, SPLN			AhR,CACD,Rad21	AC006028.9	
7	2720161	0.81	0.94	rs798554	C	T	0.21	0.31	ESDR, GI	ESDR, IPSC, ESC, BRST, BLD, MUS, SKIN, BRN, GI, KID, THYM, LNG, SPLN, CRVX	LNG,THYM,MUS,LNG	EGR1	Whn,ZBRK1	AC006028.9	
7	2720490	0.81	0.94	rs798552	G	A	0.21	0.31	ESDR, SKIN	ESDR, BRST, BLD, MUS, SKIN, BRN, GI, PLCNT, THYM, SPLN, LNG, CRVX			CTCF,RXRA,Rad21,SMC3,SP1	AC006028.9	
7	2720975	0.81	0.94	rs798550	A	G	0.21	0.31	ESDR, BLD, SKIN	ESDR, ESC, LNG, IPSC, BRST, BLD, MUS, SKIN, BRN, GI, PLCNT, THYM, PANC, SPLN, CRVX	ESDR,ESDR,ESDR,ESC,LNG,IPSC,BRST,BLD,BLD,BLD,BLD,BLD,BLD,BLD,SKIN,SKIN,HRT,LNG,PLCNT,THYM,OVRY,LNG,BLD,CRVX,LIV,BRST,MUS,MUS,BLD,BRN,SKIN,LNG	CTCF,EGR1,ZEB1,AP2ALPHA,AP2GAMMA,CEBPB,INI1,JUND,MAX,MXI1	Klf4,Klf7,SP1,Sin3Ak-20	AC006028.9	
7	2722492	0.82	0.94	rs798546	C	T	0.20	0.31	ESDR	BLD, SKIN, BRN, MUS, THYM, LNG, HRT, SPLN			Ets	AC006028.9	

7	2722752	0.81	0.93	rs798545	C	T	0.20	0.31	ESDR	BLD, SKIN, MUS, THYM, HRT, SPLN, LNG			DEC,Nkx2,OTX	AC006028.9	
7	2723468	0.8	0.92	rs798544	C	T	0.20	0.31	ESDR, ESC, IPSC, PANC, SPLN	IPSC, BLD, SKIN, GI, MUS, THYM, OVRY, PANC, HRT, SPLN	BLD,BLD,MUS,OVRY,MUS		EBF,TFIIA	AMZ1	
7	2724496	0.81	0.93	rs798540	G	A	0.20	0.31	SPLN	BLD, SKIN, GI, MUS, THYM, PANC, SPLN	THYM,OVRY		Ets,STAT	AMZ1	
7	2724982	0.81	0.94	rs78018097	C	CA	0.20	0.31	ESDR, IPSC, SKIN, SPLN	IPSC, BLD, SKIN, BRN, GI, MUS, THYM, PANC, SPLN	THYM		Evi-1,Irf,Osf2,PEBP,PU.1	AMZ1	
7	2726749	0.83	0.95	rs798536	G	A	0.20	0.31	IPSC, SKIN	IPSC, BLD, SKIN, BRN, GI, MUS, THYM, OVRY, PANC	BLD,PANC		Znf143,p53	AMZ1	
7	2732797	0.84	0.97	rs798528	A	C	0.21	0.32	ESDR, FAT, BLD, BRN, GI, MUS	ESC, ESDR, LNG, IPSC, FAT, BLD, MUS, BRN, SKIN, GI, ADRL, PANC, PLCNT, THYM, HRT, OVRY, SPLN, VAS	ADRL,BLD		AIRE,CDP,Foxf2,Foxo,Zfp105	AMZ1	intronic
7	2735908	0.91	0.97	rs798526	G	C	0.22	0.30	ESDR, BLD, SKIN, BRN, GI, HRT, MUS, SPLN	ESC, ESDR, LNG, IPSC, FAT, BLD, BRN, SKIN, GI, ADRL, HRT, KID, MUS, PLCNT, THYM, OVRY, PANC, SPLN, VAS	BLD,BLD,BLD,HRT,MUS,MUS,MUS		HDAC2,SP1,VDR	AMZ1	intronic
7	2738191	0.87	0.98	rs798518	A	G	0.22	0.32	BLD, BRN, GI, MUS	ESC, ESDR, LNG, IPSC, FAT, BLD, SKIN, BRN, GI, ADRL, HRT, PANC, MUS, PLCNT, THYM, OVRY, SPLN	BLD,BLD,BLD,LNG,MUS,OVRY,PANC,MUS,BLD	GATA1	YY1,Zfx	AMZ1	intronic
7	2742258	0.96	0.99	rs798514	T	C	0.21	0.30	ESC, ESDR	ESDR, ESC, BLD, SKIN, BRN, HRT, LNG			ATF3,ELF1,Isl2,LXR,Myc,NRSF,Rad21	AMZ1	intronic
7	2743672	0.97	0.99	rs798511	T	C	0.21	0.30	ESC, ESDR	ESDR, ESC, BRN, HRT, LNG, SPLN			SREBP	AMZ1	intronic
7	2744181	0.95	0.99	rs798508	C	G	0.21	0.30	ESC, ESDR					AMZ1	intronic
7	2749278	0.94	1	rs798506	T	C	0.27	0.28					Dmbx1	AMZ1	intronic
7	2750246	1	1	rs798502	A	C	0.27	0.30	HRT, GI, SPLN	ESC, ESDR, LNG, IPSC, FAT, BLD, GI, ADRL, HRT, OVRY, PANC, PLCNT, BRN, BONE	ESDR,ESDR,ESDR,IPSC,IPSC,HRT,KID,BLD	CTCF	AP-1,Glis2,HNF4	AMZ1	intronic
7	2751051	0.95	0.99	rs798500	T	C	0.21	0.30	HRT, GI, OVRY, PLCNT, SPLN	ESC, ESDR, LNG, IPSC, FAT, BLD, SKIN, BRN, GI, ADRL, HRT, MUS, OVRY, PANC, PLCNT, BONE				AMZ1	intronic
7	2756248	0.87	0.99	rs798498	T	G	0.22	0.32	MUS, GI, LNG	FAT, STRM, BRST, BLD, SKIN, BRN, GI, PANC, PLCNT, LNG, CRVX			Gm397	AMZ1	intronic

7	2756323	0.93	0.99	rs798497	A	G	0.21	0.31	MUS, GI, LNG	FAT, STRM, BRST, BLD, MUS, SKIN, BRN, GI, ADRL, PANC, PLCNT, LNG, CRVX			Pou3f1,TATA	AMZ1	intronic
7	2757129	0.83	0.93	rs798496	C	T	0.21	0.30	FAT, STRM, BLD, MUS, SKIN, BRN, GI, LNG, CRVX	ESDR, ESC, LNG, IPSC, FAT, STRM, BRST, BLD, MUS, BRN, SKIN, VAS, GI, ADRL, HRT, PANC, PLCNT, THYM, OVRY, SPLN, CRVX, BONE	ADRL		Foxp1,HDAC2,Pax-4,p300	AMZ1	intronic
7	2757633	0.92	1	rs798495	T	C	0.21	0.31	ESC, LNG, FAT, STRM, BRST, BLD, MUS, BRN, SKIN, GI, HRT, PLCNT, CRVX, VAS, BONE	ESC, ESDR, LNG, IPSC, FAT, STRM, BRST, BLD, MUS, BRN, SKIN, VAS, LIV, GI, ADRL, HRT, KID, PANC, PLCNT, THYM, OVRY, SPLN, CRVX, BONE	ESDR,ESDR,BRST,BLD,SKIN,SKIN,SKIN,SKIN,ADRL,BRN,HRT,KID,MUS,MUS,GI,OVRY,MUS,LNG,BLD,CRVX,BRST,MUS,MUS,VAS,BLD,BLD,BRN,SKIN,SKIN,LNG	USF1,BAF155,CMYC,MAX,MXI1,POL2,STAT3,P300		AMZ1	intronic
7	2758660	0.96	1	rs798494	C	A	0.21	0.31	ESDR, LNG, FAT, STRM, BRST, BLD, MUS, BRN, SKIN, GI, HRT, PLCNT, CRVX, BONE	ESC, ESDR, LNG, IPSC, FAT, STRM, BRST, BLD, MUS, BRN, SKIN, VAS, LIV, GI, ADRL, HRT, PANC, PLCNT, THYM, OVRY, SPLN, CRVX, BONE	ESDR,ESDR,BRST,SKIN,SKIN,HRT,GI,PLCNT,OVRY,MUS,BLD,LIV,BLD,BLD,BRN,SKIN	POL2,ZBTB7A		AMZ1	intronic
7	2759097	0.96	1	rs798493	A	G	0.21	0.31	FAT, BRST, STRM, BLD, MUS, BRN, SKIN, GI, HRT, PLCNT, LNG, CRVX	ESC, ESDR, LNG, IPSC, FAT, STRM, BRST, BLD, MUS, BRN, SKIN, LIV, GI, ADRL, HRT, PANC, PLCNT, THYM, OVRY, SPLN, CRVX, VAS, BONE	ADRL,OVRY		CEBPD,CHOP::CEBPalpha,Gfi1,HLF	AMZ1	intronic
7	2760887	0.96	1	rs798491	A	G	0.21	0.31	ESDR, FAT, BLD, MUS, SKIN, BRN, GI, HRT, PLCNT, SPLN, LNG, CRVX, BONE	ESC, ESDR, LNG, IPSC, FAT, STRM, BRST, BLD, MUS, BRN, SKIN, LIV, GI, ADRL, HRT, PANC, PLCNT, THYM, OVRY, SPLN, CRVX, BONE	ESDR,ESDR,ESC,BLD,BLD,BLD,BLD,ADRL,BRN,HRT,KID,LNG,MUS,MUS,GI,THYM,OVRY,MUS,GI,LNG,BLD,CRVX,LIV,MUS,BLD,BL	CTCF,RAD21		AMZ1	intronic

											D				
7	2761908	0.94	0.99	rs798490	G	A	0.20	0.30	BLD, MUS, SKIN, FAT, BRN, GI, HRT, SPLN, CRVX, BONE	ESC, ESDR, LNG, IPSC, FAT, STRM, BRST, BLD, MUS, BRN, SKIN, GI, ADRL, HRT, KID, PLCNT, THYM, OVRY, PANC, SPLN, CRVX, LIV, BONE	ESDR,PLCNT,OVRY,MUS,MUS,MUS		Mrg,Tgif1	AMZ1	intronic
7	2762169	0.87	0.95	rs798489	C	T	0.09	0.29	FAT, BLD, MUS, SKIN, BRN, GI, HRT, SPLN, CRVX, BONE	ESC, ESDR, LNG, IPSC, FAT, STRM, BRST, BLD, MUS, BRN, SKIN, LIV, GI, ADRL, HRT, KID, PLCNT, THYM, OVRY, PANC, SPLN, VAS, BONE	ESDR,ESDR,IPSC,PLCNT,OVRY		BDP1,CACD	AMZ1	intronic
7	2762888	0.94	0.99	rs798488	T	C	0.20	0.30	LNG, FAT, STRM, BRST, BLD, MUS, SKIN, BRN, GI, OVRY, SPLN, BONE	ESC, ESDR, LNG, IPSC, FAT, STRM, BRST, BLD, MUS, BRN, SKIN, GI, ADRL, HRT, KID, PLCNT, THYM, OVRY, PANC, SPLN, LIV, VAS, BONE	LNG,BLD,BLD,SKIN,SKIN,HRT,KID,LNG,MUS,MUS,PLCNT,GI,OVRY,MUS,LIV,MUS,BLD,LNG		NRSF,Roaz	AMZ1	intronic
7	2763309	0.94	0.99	rs798487	G	A	0.20	0.30	ESDR, LNG, FAT, STRM, BRST, BLD, MUS, SKIN, BRN, GI, KID, HRT, OVRY, SPLN, BONE	ESC, ESDR, LNG, IPSC, FAT, STRM, BRST, BLD, MUS, BRN, SKIN, GI, ADRL, HRT, KID, PLCNT, THYM, OVRY, PANC, SPLN, LIV, VAS, BONE	ESC,LNG,IPSC,BRST,BLD,BLD,BLD,SKIN,SKIN,SKIN,SKIN,ADRL,BRN,BRN,HRT,GI,KID,LNG,MUS,MUS,PLCNT,GI,OVRY,MUS,BRST,VAS,BRN,SKIN,SKIN,LNG			AMZ1	intronic
7	2763403	0.94	0.99	rs798486	A	G	0.20	0.30	ESDR, LNG, FAT, STRM, BRST, BLD, MUS, SKIN, BRN, GI, KID, HRT, OVRY, BONE	ESC, ESDR, LNG, IPSC, FAT, STRM, BRST, BLD, MUS, BRN, SKIN, GI, ADRL, HRT, KID, PLCNT, THYM, OVRY, PANC, SPLN, LIV, VAS, BONE	LNG,IPSC,IPSC,BRST,SKIN,SKIN,SKIN,BRN,HRT,GI,KID,LNG,MUS,MUS,PLCNT,GI,OVRY,PANC,MUS,BLD,BRN,SKIN,LNG		DMRT2,NRSF	AMZ1	intronic

7	2763876	0.94	0.99	rs798485	C	T	0.20	0.30	FAT, STRM, BRST, BLD, SKIN, BRN, GI, KID, MUS, BONE	ESC, ESDR, LNG, IPSC, FAT, STRM, BRST, BLD, MUS, BRN, SKIN, LIV, GI, ADRL, HRT, KID, PLCNT, THYM, OVRY, PANC, SPLN, VAS, BONE	ESC,HRT,KID,MUS,MUS,PLCNT,OVRY		TATA,ZID	AMZ1	intronic
7	2769098	0.95	1	rs1182208	T	G	0.20	0.31		BLD			Brachyury,Mxi1,Myc,Sin3Ak-20,YY1	AC006028.10	intronic
7	2771112	0.94	0.99	rs1182207	G	A	0.20	0.30	ESDR, BLD, GI	ESDR, LNG, BLD, GI, HRT, OVRY, PANC, MUS, SPLN			GR,TATA,THAP1,YY1	AC006028.10	intronic
7	2772998	0.94	0.99	rs35957220	C	G	0.20	0.31	LNG, BLD, SPLN	ESDR, LNG, IPSC, FAT, BLD, SKIN, BRN, GI, THYM, OVRY, BONE	LNG,BLD,BLD,BLD,BLD,BLD,BLD,SKIN,MUS,BLD,BLD,BRN,LNG	NFKB,BCL11A,IRF4,PAX5N19,SP1	AP-1,AP-2,BAF155,BATF,BCL,Bach1,GATA,GR,HMGN3,Irf,KAP1,Mef2,Myc,NF-E2,PRDM1,STAT,TCF4,p300	AC006028.10	intronic
7	2776956	0.93	0.99	rs141304588	TAAG	T	0.20	0.31	ESC, FAT, BRN, GI, SPLN	ESDR, LNG, IPSC, FAT, ESC, STRM, BRST, BLD, BRN, SKIN, GI, MUS, PLCNT, OVRY, PANC, HRT, SPLN			Cdx,Cdx2,Dbx1,Evi-1,Fox,Foxa,Foxd3,Foxp3,HNF1,HNF4,Hlx1,Hoxa10,Hoxa9,Ncx,Pou2f2,Sox,TCF12	GNA12	intronic
7	2777821	0.92	0.99	rs13243214	G	T	0.20	0.31	FAT, GI	ESDR, ESC, LNG, IPSC, FAT, STRM, BLD, BRN, GI, PLCNT, OVRY, HRT, MUS	OVRY			GNA12	intronic
7	2783352	0.89	0.97	rs2260230	T	A	0.20	0.31		ESDR, LNG, FAT, BRST, BLD, MUS, SKIN, HRT, THYM, GI, OVRY, PANC, PLCNT, SPLN, VAS, BRN	KID,PLCNT		PPAR,RXRA,Zic	GNA12	intronic
7	2783842	0.88	0.97	rs2644294	G	A	0.20	0.31		ESDR, IPSC, FAT, BLD, SKIN, ADRL, HRT, MUS, GI, THYM, LNG, PANC, PLCNT, VAS, BRN			Hand1,SRF,Smad3,Smad	GNA12	intronic
7	2797214	0.87	0.96	rs2533884	T	G	0.23	0.31	ESDR, MUS, SKIN, BRN,	ESC, ESDR, LNG, IPSC, FAT, STRM, BLD, MUS, BRN, SKIN, GI,	HRT,LNG,MUS		GR,TATA,YY1,Zbtb3	GNA12	intronic

									HRT, LNG, GI, BONE	ADRL, HRT, KID, PLCNT, OVRY, PANC, SPLN, LIV, VAS, BONE					
7	2801530	0.87	0.96	rs2644312	G	A	0.20	0.31	ESDR, LNG, FAT, STRM, BLD, MUS, BRN, SKIN, HRT, GI, CRVX, LIV, BONE	ESC, ESDR, LNG, IPSC, FAT, STRM, BRST, BLD, MUS, BRN, SKIN, LIV, GI, ADRL, HRT, KID, PLCNT, THYM, OVRY, PANC, SPLN, CRVX, VAS, BONE	ESDR,ESDR,LNG,BRST,BLD,BLD,SKIN,SKIN,SKIN,BRN,BRN,HRT,GI,GI,KID,LNG,MUS,MUS,PLCNT,GI,GI,OVRY,PANC,MUS,GI,LIV,MUS,MUS,BLD,BLD,BRN,SKIN,SKIN,LNG	P300	AP-4,LBP-1,LBP-9,NRSF	GNA12	intronic
7	2807260	0.83	0.93	rs2533882	G	T	0.20	0.30	FAT, BLD, SKIN	ESC, ESDR, IPSC, FAT, BRST, BLD, MUS, SKIN, LIV, BRN, GI, ADRL, HRT, KID, PANC, LNG, BONE	LNG		Foxo,Foxp1,Mef2,Nkx3,Pou2f2,Pou3f2,TATA	GNA12	intronic
7	2818242	0.83	0.94	rs960273	T	C	0.20	0.31	IPSC, FAT, SKIN, GI	ESC, ESDR, LNG, IPSC, FAT, STRM, BRST, BLD, MUS, BRN, SKIN, LIV, GI, ADRL, HRT, PANC, PLCNT, OVRY, SPLN, BONE	ESDR,SKIN,SKIN,LNG,MUS,MUS,SKIN		AP-4,CTCF,Rad21,SEF-1,SMC3	GNA12	intronic
7	2820213	0.83	0.94	rs2533879	G	A	0.20	0.31	IPSC, FAT, SKIN, GI, LNG, CRVX	ESC, ESDR, LNG, IPSC, FAT, STRM, BRST, BLD, MUS, BRN, SKIN, LIV, GI, ADRL, HRT, PANC, PLCNT, OVRY, SPLN, CRVX, VAS, BONE	IPSC		AP-2,AP-4,BCL,BHLHE40,CTCF,Egr-1,Rad21,Zic,p300	GNA12	intronic
7	2823809	0.82	0.93	rs2158694	T	G	0.21	0.31	ESC, IPSC, FAT, STRM, MUS, SKIN, BRN, HRT	ESC, ESDR, LNG, IPSC, FAT, STRM, BRST, BLD, MUS, BRN, SKIN, VAS, GI, ADRL, HRT, KID, PANC, PLCNT, SPLN, CRVX, LIV, BONE	HRT,MUS			GNA12	intronic
7	2825072	0.82	0.93	rs1713919	C	A	0.19	0.31	ESC, ESDR, IPSC, MUS, SKIN, BRN, GI, HRT, OVRY	ESC, ESDR, LNG, IPSC, FAT, BRST, BLD, STRM, MUS, BRN, SKIN, GI, ADRL, HRT, KID, PANC, PLCNT, THYM, OVRY, SPLN, CRVX, LIV, BONE	ESC,ESDR,ESC,IPSC,IPSC,BLD,OVRY	ELF1,TCF12,ZEB1,POL2,POL24H8	Foxd1,Foxq1,YY1	GNA12	intronic
7	2829888	0.83	0.93	rs1182189	G	A	0.18	0.30		ESC, ESDR, LNG, IPSC, FAT, STRM, BLD, BRN, SKIN, GI, ADRL, PANC, PLCNT, HRT, OVRY, SPLN, LIV	ESDR,PLCNT		SP2,Smad3,Smad	GNA12	intronic
7	2830351	0.83	0.94	rs1182188	T	C	0.18	0.31		ESC, ESDR, LNG, IPSC, FAT, STRM, BLD, BRN, SKIN, GI,	SKIN		ZEB1	GNA12	intronic

										ADRL, HRT, PANC, PLCNT, OVRY, SPLN, LIV, BONE					
7	2830820	0.83	0.94	rs1182187	T	G	0.17	0.31	ESDR	ESC, ESDR, LNG, IPSC, FAT, STRM, BLD, SKIN, BRN, GI, ADRL, HRT, PANC, PLCNT, OVRY, SPLN, LIV, BONE	ESDR,LNG,PLCNT,LIV	TCF4	E2A,ERalpha-a,HEN1,Hic1,Lmo2-complex,TCF12,ZEB1	GNA12	intronic
7	2831683	0.81	0.93	rs1182186	A	G	0.18	0.31		ESC, ESDR, LNG, IPSC, FAT, BLD, SKIN, BRN, GI, ADRL, HRT, PANC, PLCNT, OVRY, LIV, BONE	KID		LBP-1	GNA12	intronic
7	2832599	0.8	0.92	rs1182185	A	G	0.18	0.31	LIV, GI, SPLN	ESC, ESDR, LNG, IPSC, FAT, BLD, SKIN, LIV, BRN, GI, ADRL, HRT, PANC, PLCNT, OVRY, SPLN	GI,GI,LNG,GI		EWSR1-FLI1,Hoxd8,Pou3f2,TCF12	GNA12	intronic
7	2832812	0.81	0.92	rs1182184	G	A	0.17	0.31	LIV, GI, PANC, SPLN	ESC, ESDR, LNG, IPSC, FAT, BLD, SKIN, LIV, BRN, GI, ADRL, HRT, PANC, PLCNT, OVRY, SPLN	BLD,GI,GI,PLCNT,THYM,GI		E2A,LBP-1,NRSF,RP58,TAL1,TCF12	GNA12	intronic
7	2833502	0.81	0.92	rs1182183	C	T	0.18	0.31	HRT, SPLN	ESC, ESDR, LNG, IPSC, FAT, BLD, SKIN, BRN, GI, ADRL, HRT, PANC, PLCNT, OVRY, SPLN, LIV	HRT,PLCNT,BLD,SKIN	GATA2	En-1,Evi-1,Foxa,Gbx1,Hlxb9,Hoxa3,Hoxb3,Hoxb7,Mef2,Msx-1,Nkx6-1,Nkx6-2,Pax7,Phox2a,Pou2f2,Pou3f2,Pou3f4,Pou6f1,Prrx1	GNA12	intronic
		0.81	0.92	rs80098895	C	T	0.18	0.31	BLD, HRT, SPLN	ESC, ESDR, LNG, IPSC, FAT, BLD, SKIN, BRN, GI, ADRL, HRT, PANC, PLCNT, OVRY, SPLN, LIV	HRT,PLCNT		HNF1,HNF4	GNA12	intronic
7	2833613	0.81	0.92	rs1182181	C	G	0.17	0.31	BLD, HRT, SPLN	ESC, ESDR, LNG, IPSC, FAT, BLD, SKIN, BRN, GI, ADRL, HRT, PANC, PLCNT, OVRY, SPLN, LIV	HRT,PLCNT		GR,HNF1,Homez,Maf,Pax-4,ZBRK1	GNA12	intronic
7	2833739	0.81	0.92	rs33942482	CTG	C	0.18	0.31	BLD, HRT, SPLN	ESC, ESDR, LNG, IPSC, FAT, BLD, SKIN, BRN, GI, ADRL, HRT, PANC, PLCNT, OVRY, SPLN, LIV	IPSC,PLCNT		Hand1,LF-A1,NERF1a	GNA12	intronic
7	2834014	0.81	0.92	rs1182179	A	G	0.18	0.31	BLD, HRT, SPLN	ESC, ESDR, LNG, IPSC, FAT, BLD, SKIN, BRN, GI, ADRL, HRT, PANC, PLCNT, OVRY, SPLN, LIV	OVRY			GNA12	intronic
7	2834129	0.81	0.92	rs1182178	G	A	0.17	0.31	BLD, HRT, SPLN	ESC, ESDR, LNG, IPSC, FAT, BLD, SKIN, BRN, GI, HRT, PANC,	ESDR,BLD		Egr-1,HNF4	GNA12	intronic

										PLCNT, OVRY, SPLN, LIV				
7	2834906	0.81	0.92	rs1182177	C	T	0.17	0.31	ESC, BLD, HRT	ESC, ESDR, LNG, IPSC, FAT, BRST, BLD, SKIN, BRN, GI, HRT, PANC, PLCNT, OVRY, SPLN, LIV	IPSC,BLD,PLCNT,OVRY,CRVX,BLD,SKIN	STAT3	Ets	GNA12	intronic
7	2834967	0.81	0.92	rs1182176	A	G	0.18	0.31	ESC	ESC, ESDR, LNG, IPSC, FAT, BRST, BLD, SKIN, BRN, GI, HRT, PANC, PLCNT, OVRY, SPLN, LIV	BLD,KID,PLCNT,CRVX,BLD,SKIN	STAT3	GZF1,Nrf-2,Pax-2,Pax-5	GNA12	intronic
7	2835786	0.82	0.92	rs1182174	G	A	0.18	0.31		ESC, ESDR, IPSC, FAT, BRST, BLD, SKIN, VAS, BRN, GI, PANC, LNG, PLCNT, HRT, OVRY, LIV	IPSC		Mrg	GNA12	intronic
7	2835936	0.81	0.92	rs1183085	G	A	0.17	0.31		ESC, ESDR, IPSC, FAT, BRST, BLD, SKIN, BRN, GI, PANC, LNG, MUS, PLCNT, HRT, OVRY, LIV			AIRE,Irf	GNA12	intronic
7	2838876	0.8	0.92	rs1182171	A	G	0.17	0.31	ESDR, FAT, BLD, BRN, GI, LNG	ESC, ESDR, LNG, IPSC, FAT, STRM, BRST, BLD, BRN, SKIN, GI, ADRL, KID, PANC, MUS, THYM, OVRY, HRT, SPLN, LIV, VAS, BONE	BLD,BLD		BRCA1,RREB-1	GNA12	intronic
19	33034807	0.87	0.94	rs9749364	T	G	0.18	0.09		ESDR, GI, LNG			Bcl6b,Evi-1,Foxp1,HDAC2,Hoxa13,PLZF,Pax-4,STAT,Zfp105,p300	RHPN2	intronic
19	33036042	0.94	0.98	rs11880141	G	A	0.18	0.10	GI	ESDR, BLD, PLCNT, THYM, GI, LNG			Rad21,SMC3,SP1,ZBRK1	RHPN2	intronic
19	33036690	0.94	0.98	rs10424333	C	G	0.18	0.10	BLD	ESDR, IPSC, BLD, GI, THYM, PANC			Pax-5	RHPN2	intronic
19	33036771	0.94	0.98	rs10404631	T	A	0.18	0.10	BLD	ESDR, IPSC, BLD, GI, THYM, PANC			Ascl2,Brachyury,E2A,HDAC2,Myf,NRSF,Pitx3,Sin3Ak-20	RHPN2	intronic
19	33041394	1	1	rs10411210	C	T	0.19	0.10	IPSC, BRN, SKIN, LIV, GI, LNG, CRVX	ESC, ESDR, IPSC, FAT, BRST, STRM, MUS, BRN, SKIN, LIV, GI, ADRL, KID, LNG, THYM, PANC, PLCNT, HRT, CRVX	ESC,BRST,BLD,SKIN,SKIN,SKIN,SKIN,BRN,BRN,HRT,GI,GI,KID,LNG,MUS,PLCNT,GI,GI,	GR,POL24H8,AP2ALPHA,CEBPB,RFX5,STAT3,FOSL2,FOXA1,FOXA2,HDAC2,H	FAC1,Foxa,Foxo,Ik-1,Ik-2,Mef2,Myb,STAT,Sox	RHPN2	intronic

											PANC,GI,LNG,BLD,CRVX,LIV,BRST,MUS,BRN,SKIN,SKIN,LNG	NF4A,HNF4G,JUND,P300,RXRA,SP1,TCF4			
19	33047877	0.9	0.98	rs12459751	A	G	0.19	0.10	ESDR, GI, CRVX, SKIN	ESC, ESDR, IPSC, BRST, SKIN, LIV, GI, PLCNT, PANC, LNG, CRVX, MUS, BLD	PLCNT,GI,CRVX,BRST		Zbtb3	RHPN2	intronic
19	33047994	0.9	0.98	rs73585909	A	G	0.19	0.10	ESDR, GI, CRVX, SKIN	ESC, ESDR, IPSC, BRST, SKIN, LIV, GI, PLCNT, PANC, LNG, CRVX, MUS, BLD	GI,CRVX		CCNT2,ERalpha-a,Ets,PLAG1,Pax-4,RREB-1,SP1,Zfp281,Zic	RHPN2	intronic
19	33048155	0.9	0.98	rs73585910	A	G	0.19	0.10	ESDR, GI, CRVX	ESC, ESDR, IPSC, BRST, SKIN, LIV, GI, PLCNT, PANC, LNG, CRVX, MUS, BLD	PLCNT,GI		CDP,HNF4,Nkx2,RXRA	RHPN2	intronic
19	33049306	0.9	0.98	rs28363937	C	T	0.19	0.10	ESDR, GI, LNG, CRVX	ESC, ESDR, IPSC, BRST, SKIN, LIV, BRN, GI, KID, PLCNT, PANC, LNG, CRVX, BLD	BRST,GI,GI,GI,GI,CRVX,LIV,BRN		CTCF,NRSF,RXRA	RHPN2	intronic
19	33049363	0.9	0.98	rs28403377	C	T	0.19	0.10	ESDR, GI, LNG, CRVX	ESC, ESDR, IPSC, BRST, SKIN, LIV, BRN, GI, KID, PLCNT, PANC, LNG, CRVX, BLD	ESDR,BRST,GI,GI,GI,GI,GI,LNG,CRVX,LIV,LNG			RHPN2	intronic
19	33050062	0.9	0.98	rs7247582	T	C	0.19	0.10	ESDR, IPSC, GI, LNG, CRVX	ESC, ESDR, IPSC, SKIN, LIV, BRN, GI, KID, LNG, PANC, PLCNT, CRVX	LNG		Elf5,Myb,ZNF219	RHPN2	intronic
19	33053281	0.9	0.98	rs60507951	G	A	0.20	0.10	ESDR, GI, LNG, CRVX	ESC, ESDR, BRST, SKIN, LIV, BRN, GI, KID, PANC, PLCNT, HRT, LNG, CRVX			Mtf1,Zbtb3	RHPN2	intronic
19	33058869	0.84	0.94	rs7255601	G	C	0.13	0.10		ESDR, ESC, BRN, GI, PANC, LNG, LIV			PLAG1	RHPN2	intronic
19	33060522	0.84	0.94	rs11881367	G	A	0.13	0.10	ESDR, IPSC, GI, SPLN, CRVX	ESDR, ESC, IPSC, LIV, GI, PLCNT, PANC, LNG			Foxp1,STAT,Sox,YY1	RHPN2	intronic
19	33060866	0.83	0.94	rs7249860	C	T	0.13	0.10	ESDR, IPSC, GI, SPLN, CRVX	ESDR, ESC, IPSC, LIV, GI, PLCNT, LNG, PANC	GI			RHPN2	intronic
19	33063203	0.83	0.94	rs7258173	T	A	0.13	0.10	ESC, ESDR, LNG, IPSC, BRST, BLD, BRN, SKIN, VAS, LIV, GI, KID, HRT, PANC, PLCNT, MUS, SPLN, CRVX	ESC, ESDR, IPSC, FAT, STRM, BRST, BLD, BRN, SKIN, LIV, GI, HRT, LNG, MUS, PLCNT, THYM, OVRY, PANC, SPLN, CRVX	GI,GI,PLCNT,GI		Gbx1,Hoxa4,Isx,Lhx4,Lhx8,Pou2f2,Pou6f1	RHPN2	intronic

19	33065984	0.82	0.92	rs8104911	G	C	0.13	0.10	ESC, ESDR, IPSC, BLD, BRN, SKIN, GI, PLCNT, LNG, HRT, SPLN	ESC, ESDR, BLD, SKIN, GI, PLCNT, LNG, SPLN, LIV			ERalpha-a,Nr2f2,SF1,T3R	1.1kb 5' of RHPN2	
19	33066624	0.83	0.94	rs8109970	T	C	0.12	0.10	ESDR, ESC, SKIN, LNG, GI	ESC, ESDR, IPSC, BLD, SKIN, GI, PLCNT, LNG, LIV	BLD,BRN,PLCNT,LIV			1.7kb 5' of RHPN2	
19	33067556	0.82	0.92	rs7256899	C	G	0.12	0.10	ESDR, GI	ESDR, SKIN, GI, PLCNT, LNG, CRVX, LIV	CRVX		Irx	2.6kb 5' of RHPN2	
19	33071558	0.82	0.92	rs7250288	G	A	0.12	0.10	LNG	ESDR, BRST, SKIN, GI, PLCNT, LNG, CRVX			Pbx3	6.6kb 5' of RHPN2	
19	33073956	0.82	0.92	rs7248625	A	C	0.12	0.10	ESDR		LNG		BDP1,GR,LUN-1	6.9kb 5' of GPATCH1	
1	182998336	0.92	0.97	rs10690446	G	GGCAAA	0.57	0.55	ESC	ESDR, FAT, BRST, SKIN			Osf2,PEBP,STAT	16kb 5' of RNU6-3	
1	182999875	1	1	rs3935221	A	G	0.58	0.55	ESC, FAT	ESDR, FAT, BRN, LNG			Rad21	18kb 5' of RNU6-3	
1	183000693	1	1	rs10797800	C	A	0.58	0.55	FAT	ESDR, FAT, STRM, BRN, GI, LNG, MUS	LNG,MUS,SKIN		CCNT2,Evi-1,GATA,Nkx3,TAL1	18kb 5' of RNU6-3	
1	183001103	1	1	rs4304541	G	T	0.58	0.55	FAT	ESDR, FAT, STRM, VAS, GI, LNG, PLCNT, HRT, MUS			DBP,DMRT3,DMRT5,Fox,Foxj2,Pou1f1,Sox,TCF12,Zfp105,p300	19kb 5' of RNU6-3	
1	183001160	0.92	1	rs200348453	AAG	A	0.57	0.53	FAT	FAT, STRM, VAS, GI, LNG, PLCNT, HRT, MUS			Evi-1,GR,HDAC2,HMG-IY,Hoxa10,Hoxa9,Irf,Nanog,STAT,Zfp105,p300	19kb 5' of RNU6-3	
1	183001161	0.98	1	rs5779148	AG	A	0.57	0.54	FAT	FAT, STRM, VAS, GI, LNG, PLCNT, HRT, MUS			Evi-1,GR,HDAC2,HM	19kb 5' of	

													G-IY,Hoxa10,Hoxa9,Irf,Nanog,Pou2f2,STAT,Zfp105,p300	RNU6-3	
1	183001202	0.99	1	rs12753817	T	A	0.58	0.54	FAT	FAT, STRM, VAS, GI, LNG, PLCNT, HRT, MUS				19kb 5' of RNU6-3	
1	183001339	0.99	1	rs34850574	C	CA	0.58	0.54	FAT	FAT, STRM, VAS, GI, LNG, PLCNT, HRT, MUS, SKIN				19kb 5' of RNU6-3	
1	183001389	0.94	0.98	rs4651134	T	C	0.57	0.54	FAT	FAT, STRM, GI, LNG, PLCNT, HRT, MUS, SKIN			Maf,Pitx2	19kb 5' of RNU6-3	
1	183001563	0.99	0.99	rs4651136	T	C	0.58	0.55	ESDR, FAT	FAT, STRM, GI, LNG, PLCNT, HRT, OVRY, MUS, SKIN			HNF4,Myf,RXRA	19kb 5' of RNU6-3	
1	183001702	0.99	0.99	rs4233192	G	T	0.58	0.55	ESDR, FAT	FAT, STRM, GI, KID, LNG, PLCNT, HRT, OVRY, MUS, SKIN			GATA,HNF1,Hoxa3,Myc,Pax-4,XBP-1	19kb 5' of RNU6-3	
1	183001730	0.98	0.99	rs4233193	C	T	0.58	0.55	ESDR, FAT	FAT, STRM, GI, KID, LNG, PLCNT, HRT, OVRY, MUS, SKIN				19kb 5' of RNU6-3	
1	183002612	1	1	rs10911185	G	C	0.58	0.55	FAT, STRM	FAT, STRM, BRST, MUS, SKIN, LIV, GI, KID, LNG, OVRY, CRVX, VAS, BONE	CRVX		Foxj1,GR,RFX5,ZEB1	20kb 5' of RNU6-3	
1	183002681	1	1	rs10737235	T	C	0.58	0.55	FAT, STRM	FAT, STRM, BRST, MUS, SKIN, LIV, GI, KID, LNG, OVRY, CRVX, VAS, BONE	CRVX		STAT,Sox	20kb 5' of RNU6-3	
1	183003407	0.99	1	rs10911186	A	G	0.58	0.55	FAT, STRM, LNG, SKIN, BONE	ESDR, FAT, STRM, BRST, MUS, SKIN, LIV, GI, KID, LNG, OVRY, CRVX, VAS, BONE	ESDR,LNG,SKIN,SKIN,SKIN,HRT,KID,LNG,MUS,MUS,GI,MUS,MUS,MUS,VAS,BRN,SKIN,SKIN,LNG	CJUN,GATA2		20kb 5' of LAMC1	
1	183004023	1	1	rs10797802	T	A	0.58	0.55	FAT, STRM, LNG, SKIN, BONE	FAT, STRM, BRST, SKIN, LIV, GI, KID, LNG, OVRY, MUS, VAS, BONE			AIRE,Arid5b,GR	19kb 5' of LAMC1	

1	183004051	1	1	rs8179262	C	T	0.58	0.55	FAT, STRM, LNG, SKIN, BONE	FAT, BRST, SKIN, LIV, GI, KID, LNG, OVRY, MUS, VAS, BONE			Mtf1,NRSF,Pou2f2	19kb 5' of LAMC1	
1	183004356	1	1	rs10752881	A	G	0.58	0.55	FAT, LNG, SKIN, BONE	FAT, BRST, SKIN, GI, KID, LNG, OVRY, MUS, VAS, BONE	SKIN	USF1	Bbx,Sox	19kb 5' of LAMC1	
1	183005437	0.96	0.98	rs8179361	T	C	0.58	0.55	FAT				AP-1,Egr-1,PRDM1, RXRA	18kb 5' of LAMC1	
1	183005463	0.94	0.98	rs8179282	C	G	0.57	0.54	FAT				Egr-1,Zic	18kb 5' of LAMC1	
1	183005467	0.94	0.98	rs8179283	C	T	0.57	0.54	FAT				Egr-1,Zic	18kb 5' of LAMC1	
1	183005572	0.96	0.98	rs8179284	C	T	0.58	0.55					Pou2f2,RXRA,SP2,SRF,TCF12,WT1	18kb 5' of LAMC1	
1	183005615	0.96	0.98	rs4652757	T	A	0.58	0.55					DMRT2,Foxp1,Hoxa10,Hoxa9,Hoxb9,SRF,TATA	18kb 5' of LAMC1	
1	183005965	0.95	0.98	rs4652758	T	C	0.58	0.54					BHLHE40,ELF1,Egr-1,Hic1,NRSF,YY1,Zfp161	17kb 5' of LAMC1	
1	183006067	0.95	0.98	rs9943069	G	A	0.58	0.54					Mef2,ZBTB33	17kb 5' of LAMC1	
1	183006893	0.96	0.98	rs4575047	A	G	0.58	0.55					Smad4,Tgif1	17kb 5' of LAMC1	
1	183006899	0.96	0.98	rs4596846	C	T	0.58	0.55						17kb 5' of LAMC1	
1	183006998	0.95	0.98	rs4420053	A	C	0.57	0.54					Barx1,Barx2,Bsx,Dbx1,Dlx2,Dlx3,Dlx5,F	16kb 5' of LAMC1	

													oxa,HP1-site-factor,Hlx1,Hlxb9,Hoxa5,Hoxb7,Hoxb8,Hoxc6,Hoxd8,Msx-1,Nkx6-1,Nkx6-2,Pax-4,Pax-6,Pou1f1,Pou2f2,Pou3f4,TAL1		
1	183007988	0.94	0.98	rs9943229	A	G	0.58	0.54	SKIN	FAT, BRST, SKIN			ERalpha-a,TCF11::MafG,ZID	15kb 5' of LAMC1	
1	183008062	0.95	0.98	rs6701400	C	T	0.58	0.54	SKIN	FAT, BRST, SKIN				15kb 5' of LAMC1	
1	183008192	0.93	0.98	rs6701679	G	A	0.58	0.54		FAT, BRST, SKIN			ATF3,CDP,DEC,Nkx2,SREBP	15kb 5' of LAMC1	
1	183008558	0.95	0.98	rs10797803	A	T	0.58	0.54		BRST, SKIN			Crx	15kb 5' of LAMC1	
1	183008598	0.93	0.98	rs10797804	T	C	0.58	0.54		BRST, SKIN			Pax-8	15kb 5' of LAMC1	
1	183009171	0.92	0.97	rs9787083	C	T	0.58	0.54		ESC, IPSC			Foxm1,Mef2,SIX5,Znf143	14kb 5' of LAMC1	
1	183009239	0.94	0.97	rs9787228	T	C	0.58	0.54		ESC, IPSC			Ik-1,Rad21	14kb 5' of LAMC1	
1	183009324	0.94	0.97	rs9787327	A	G	0.58	0.54		ESC, IPSC			AP-1,Maf,NF-E2,Nrf-2,TCF11::MafG	14kb 5' of LAMC1	
1	183009471	0.9	0.97	rs10797805	T	C	0.58	0.53		ESC, IPSC			Foxa,Mef2	14kb 5' of LAMC1	
1	183009474	0.89	0.96	rs10797806	G	A	0.57	0.54		ESC, IPSC			Mef2	14kb 5' of	

														LAMC1	
1	183009548	0.94	0.97	rs10911189	C	T	0.58	0.54	ESC	ESC, IPSC			Ik-1	14kb 5' of LAMC1	
1	183009738	0.95	0.97	rs10797807	T	C	0.58	0.55	ESC	ESC, IPSC			GATA,Pou5f1	14kb 5' of LAMC1	
1	183009945	0.95	0.97	rs10911190	C	T	0.58	0.55	IPSC	ESC, IPSC			Hoxa10	14kb 5' of LAMC1	
1	183010173	0.91	0.96	rs71641843	TAC CAT AG	T	0.58	0.54	ESC, IPSC	ESC, IPSC		ELF1	Pou2f2,Zic	13kb 5' of LAMC1	
1	183010488	0.94	0.97	rs10797808	T	C	0.58	0.54	ESC, IPSC	ESC, IPSC				13kb 5' of LAMC1	
1	183010881	0.88	0.97	rs56058235	ACC	A	0.55	0.53	ESC, IPSC	ESC, IPSC			CTCF,Cdx2,ERalpha-a,Sox,ZEB1	13kb 5' of LAMC1	
1	183011060	0.86	0.97	rs4403588	A	G	0.57	0.53	ESC, IPSC	ESC, IPSC			BHLHE40,LXR,Pou2f2	12kb 5' of LAMC1	
1	183011745	0.94	0.97	rs10797809	T	C	0.58	0.54	IPSC	ESC, IPSC, MUS, SKIN, ADRL			ERalpha-a,STAT,TLX1::NFIC	12kb 5' of LAMC1	
1	183011815	0.94	0.97	rs10797810	C	T	0.58	0.54	IPSC	ESC, IPSC, MUS, SKIN, ADRL			BCL,Gfi1,NRSF,Sin3Ak-20	12kb 5' of LAMC1	
1	183011828	0.94	0.97	rs10752883	G	A	0.58	0.54	IPSC	ESC, IPSC, MUS, SKIN, ADRL			Ets,NRSF,Znf143	12kb 5' of LAMC1	
1	183011873	0.93	0.97	rs10752884	G	A	0.57	0.54	IPSC	ESC, ESDR, IPSC, MUS, SKIN, ADRL			CHOP::CEBPalpha,Gfi1,NF-kappaB	12kb 5' of LAMC1	
1	183013038	0.94	0.97	rs4487994	T	A	0.58	0.55	IPSC	ESC, IPSC, MUS, SKIN, FAT, ADRL, HRT, VAS, LNG			MZF1::1-4,Pbx-1,RREB-1,UF1H3BETA	10kb 5' of LAMC1	

1	183013168	0.94	0.97	rs4454510	G	A	0.58	0.55		IPSC, MUS, SKIN, FAT, ADRL, HRT, VAS, LNG				10kb 5' of LAMC1	
1	183013403	0.94	0.97	rs4442336	T	G	0.58	0.55		IPSC, MUS, SKIN, FAT, ADRL, HRT, VAS, LNG			Cart1,Dobox4,Foxj2,NF-kappaB,RFX5,Sox	10kb 5' of LAMC1	
1	183013462	0.94	0.97	rs11321321	TG	T	0.58	0.55		IPSC, MUS, SKIN, FAT, ADRL, HRT, VAS, LNG			Dbx1,Fox,Hoxd8,Ncx,Pou3f2	10kb 5' of LAMC1	
1	183013743	0.95	0.97	rs10752885	G	C	0.58	0.55		IPSC, MUS, SKIN, FAT, ADRL, HRT, LNG			Nanog	9.7kb 5' of LAMC1	
1	183014062	0.95	0.97	rs6673559	G	C	0.59	0.55		IPSC, MUS, SKIN, FAT, ADRL, HRT, LNG				9.4kb 5' of LAMC1	
1	183014198	0.95	0.97	rs6697739	A	C	0.58	0.55		IPSC, MUS, SKIN, ADRL, HRT, LNG			NF-Y,SP1,SP2,TATA,Tgif1	9.3kb 5' of LAMC1	
1	183014500	0.92	0.97	rs79318669	GTCTT	G	0.58	0.54		IPSC, MUS, SKIN, ADRL, LNG, BONE			HDAC2	9kb 5' of LAMC1	
1	183014697	0.95	0.97	rs12403189	G	C	0.58	0.55		SKIN			DMRT7,Ets,Foxp1,Irf,Mef2	8.8kb 5' of LAMC1	
1	183014892	0.95	0.97	rs36043504	C	T	0.58	0.55		SKIN			Mef2	8.6kb 5' of LAMC1	
1	183015297	0.95	0.97	rs10797811	G	A	0.58	0.55						8.2kb 5' of LAMC1	
1	183015389	0.95	0.97	rs10911191	T	C	0.58	0.55					AIRE,Pou3f2	8.1kb 5' of LAMC1	
1	183015462	0.95	0.97	rs10797812	G	A	0.58	0.55					CIZ	8kb 5' of LAMC1	
1	183015848	0.94	0.97	rs4593781	C	T	0.58	0.54					Arid3a,Dbx1,Foxi1,F	7.6kb 5' of	

													oxj1,HNF1,Irx,Pou2f2,Pou3f2,Zfp187	LAMC1	
1	183016390	0.95	0.97	rs12028732	G	C	0.58	0.55					Irf	7.1kb 5' of LAMC1	
1	183016446	0.95	0.97	rs12028740	G	A	0.58	0.55						7kb 5' of LAMC1	
1	183016581	0.95	0.97	rs12035773	A	G	0.57	0.55					DMRT1,DMRT2,DMRT3,DMRT5,RFX5	6.9kb 5' of LAMC1	
1	183017306	0.95	0.97	rs4402094	G	A	0.58	0.55		SKIN, CRVX				6.2kb 5' of LAMC1	
1	183017457	0.95	0.97	rs6424879	T	C	0.58	0.55		SKIN, CRVX			CACD,CTCF,Egr-1,Irf,RXRA,SMC3,SP1,Sp4,Zfp281	6kb 5' of LAMC1	
1	183017544	0.94	0.97	rs6424880	C	T	0.58	0.54		SKIN, CRVX			Ascl2,E2A,Myf,NRSF,p300	5.9kb 5' of LAMC1	
1	183017652	0.88	0.97	rs6424882	T	C	0.55	0.53		SKIN, CRVX			AP-1,TCF11::MafG	5.8kb 5' of LAMC1	
1	183018113	0.95	0.97	rs10752886	A	G	0.58	0.55		BRST, SKIN, CRVX	SKIN		TEF-1	5.3kb 5' of LAMC1	
1	183018892	0.94	0.97	rs12404594	G	A	0.58	0.55		IPSC, BRST, SKIN, FAT, VAS, LIV, GI, MUS, PANC, CRVX			Dbx1,PLZF,Pax-4,Pou2f2,RXRA,Zfp105	4.6kb 5' of LAMC1	
1	183019117	0.95	0.97	rs12133714	A	G	0.58	0.55	IPSC, SKIN	ESC, ESDR, IPSC, BRST, SKIN, FAT, VAS, LIV, GI, MUS, PANC, CRVX, BRN			Foxc1,Foxl1,Pou2f2	4.3kb 5' of LAMC1	
1	183019217	0.95	0.97	rs4483371	G	A	0.58	0.55	IPSC, SKIN, CRVX	ESC, ESDR, IPSC, BRST, SKIN, FAT, VAS, LIV, GI, ADRL, PANC, MUS, CRVX, BRN	CRVX	GATA2,CJUN,P300	Brachyury,CAC-binding-protein,EWSR1-FLI1,INSM1,Pou2f2,	4.2kb 5' of LAMC1	

													SP1,STAT		
1	183019279	0.95	0.97	rs12126434	G	A	0.58	0.55	IPSC, SKIN, CRVX	ESC, ESDR, IPSC, FAT, BRST, SKIN, VAS, LIV, GI, ADRL, LNG, MUS, PANC, PLCNT, CRVX, BRN	BRST,GI,PANC,CRVX, SKIN,LNG	GATA2,CJUN,P300	Dobox4,SIX5	4.2kb 5' of LAMC1	
1	183019473	0.95	0.97	rs10737236	T	C	0.58	0.55	IPSC, SKIN, CRVX	ESC, ESDR, IPSC, FAT, STRM, BRST, MUS, SKIN, VAS, LIV, GI, ADRL, LNG, PANC, PLCNT, CRVX, BRN	BRST,GI,GI,PANC,CR VX,VAS	GATA2	Evi-1	4kb 5' of LAMC1	
1	183020134	0.95	0.97	rs10737237	A	G	0.58	0.55	IPSC, SKIN, FAT, LNG, CRVX	ESC, ESDR, IPSC, FAT, STRM, BRST, MUS, BRN, SKIN, VAS, LIV, GI, ADRL, LNG, OVRY, PANC, PLCNT, CRVX	ESDR,ESC,LNG,BRST, SKIN,LNG,MUS,GI,GI, PANC,LNG,CRVX,VAS ,BRN	CTCF,USF1,FOXA1	AP-1,Duxl,Elf5,Lhx4, NF-Y,Pbx-1,Pbx3,RF X5,SP1,SP2,TATA	3.3kb 5' of LAMC1	
1	183020231	0.95	0.97	rs10797813	C	T	0.58	0.55	IPSC, SKIN, BRN, LNG, CRVX	ESC, ESDR, IPSC, FAT, BRST, STRM, MUS, BRN, SKIN, VAS, LIV, GI, ADRL, LNG, PANC, PLCNT, CRVX	VAS		Dbx1,Foxa,Foxj2,Ho xb8,Hoxd10,Sox	3.2kb 5' of LAMC1	
1	183020476	0.84	0.96	rs20162302 5	CTT TCT	C	0.55	0.52	IPSC, SKIN, BRN, LNG, CRVX	ESC, ESDR, IPSC, FAT, BRST, BLD, BRN, SKIN, VAS, LIV, GI, ADRL, MUS, LNG, PANC, PLCNT, CRVX			EWSR1-FLI1,Evi-1,F oxp1,GATA,HDAC2,I rf,Nanog,Pax-5,RXR A,STAT,TATA,Zfp10 5,p300	3kb 5' of LAMC1	
1	183020814	0.91	0.98	rs12132262	T	A	0.57	0.53	ESC, IPSC, SKIN, BRN, LNG, CRVX	ESC, ESDR, IPSC, FAT, BRST, BLD, BRN, SKIN, VAS, LIV, GI, ADRL, MUS, LNG, PANC, PLCNT, CRVX			Dbx1,Evi-1,Foxa,Fox d3,Foxo,Foxp1,HDA C2,HMG-IY,Ncx,Nkx 6-1,Pax-4,Pax-6,Pou 2f2,Pou3f4,Zfp105	2.6kb 5' of LAMC1	
1	183021699	0.95	0.97	rs10752887	T	C	0.58	0.55	ESC, ESDR, IPSC, FAT, STRM, BLD, MUS, SKIN, VAS, BRN, GI, HRT, LNG, CRVX	ESC, ESDR, IPSC, FAT, BRST, BLD, MUS, BRN, SKIN, VAS, GI, ADRL, KID, LNG, PLCNT, CRVX			DMRT2,Nanog,Pou1 f1,Pou2f2,Pou5f1,T ATA	1.8kb 5' of LAMC1	
1	183021794	0.95	0.97	rs10797814	C	A	0.58	0.55	ESC, ESDR, IPSC, FAT, STRM, BRST, BLD, MUS, SKIN, VAS,	ESC, ESDR, IPSC, FAT, STRM, BRST, BLD, MUS, BRN, SKIN, VAS, GI, ADRL, KID, LNG, PLCNT, CRVX, BONE			XBP-1	1.7kb 5' of LAMC1	

									BRN, GI, HRT, LNG, CRVX						
1	183022220	0.95	0.97	rs6695746	T	C	0.58	0.55	ESC, ESDR, LNG, IPSC, FAT, STRM, BRST, BLD, MUS, BRN, SKIN, VAS, LIV, GI, ADRL, HRT, KID, PANC, PLCNT, OVRY, THYM, CRVX, BONE	ESC, ESDR, IPSC, FAT, STRM, BRST, BLD, MUS, BRN, SKIN, VAS, LIV, GI, ADRL, HRT, KID, LNG, PLCNT, THYM, SPLN, CRVX, BONE	ESDR,ESDR,ESDR,ESC,BRST,SKIN,SKIN,SKIN,ADRL,GI,GI,KID,LNG,MUS,MUS,PLCNT,GI,OVRY,PANC,MUS,GI,CRVX,BRST,MUS,MUS,VAS,BLD	POL2	Foxp1,Pou1f1,Pou2f2,Pou3f3	1.2kb 5' of LAMC1	
1	183022282	0.94	0.97	rs6658501	A	G	0.58	0.55	ESC, ESDR, LNG, IPSC, FAT, STRM, BRST, BLD, MUS, BRN, SKIN, VAS, LIV, GI, ADRL, HRT, KID, PANC, PLCNT, OVRY, THYM, CRVX, BONE	ESC, ESDR, IPSC, FAT, STRM, BRST, BLD, MUS, BRN, SKIN, VAS, LIV, GI, ADRL, HRT, KID, LNG, PLCNT, THYM, SPLN, CRVX, BONE	ESC,ESDR,ESDR,ESC,BRST,SKIN,SKIN,SKIN,HRT,GI,GI,LNG,MUS,MUS,PLCNT,GI,OVRY,PANC,MUS,GI,CRVX,BRST,MUS,MUS,VAS,BLD,SKIN	POL2,POL2B,INI1,HAE2F1	Bcl6b,Foxa,Pax-5,Pdx1,Pou2f2,STAT	1.2kb 5' of LAMC1	
1	183022327	0.95	0.97	rs6695837	T	C	0.58	0.55	ESC, ESDR, LNG, IPSC, FAT, STRM, BRST, BLD, MUS, BRN, SKIN, VAS, LIV, GI, ADRL, HRT, KID, PANC, PLCNT, OVRY, THYM, CRVX, BONE	ESC, ESDR, IPSC, FAT, STRM, BRST, BLD, MUS, BRN, SKIN, VAS, LIV, GI, ADRL, HRT, KID, LNG, THYM, PLCNT, SPLN, CRVX, BONE	ESC,ESDR,ESDR,ESC,IPSC,BRST,BLD,BLD,BLD,SKIN,SKIN,SKIN,SKIN,BRN,HRT,GI,GI,LNG,MUS,MUS,PLCNT,GI,OVRY,PANC,MUS,GI,LNG,BLD,CRVX,BRST,MUS,MUS,VAS,BLD,BRN,SKIN,LNG	POL2,POL2B,BAF155,INI1,JUNB,HAE2F1,RAD21		1.1kb 5' of LAMC1	
1	183023026	0.91	0.97	rs11582514	T	C	0.57	0.54	ESC, ESDR, LNG, IPSC, FAT, STRM, BRST, BLD, MUS,	ESDR, BLD, SKIN, FAT, BRN, GI, MUS, THYM, SPLN	ESC,ESDR,ESDR,ESDR,ESDR,ESC,LNG,IPSC,I	POL2,POL24H8,RXRA,TAF1,TBP,HEY1	AP-2,BHLHE40,ERalpha-a,HEY1,MOVO-	435bp 5' of LAMC1	

									BRN, SKIN, VAS, LIV, GI, ADRL, HRT, KID, PANC, PLCNT, THYM, OVRY, SPLN, CRVX, BONE		PSC,BRST,BLD,BLD,BLD,BLD,SKIN,SKIN,SKIN,SKIN,ADRL,BRN,HRT,GI,KID,LNG,MUS,MUS,PLCNT,GI,OVRY,PANC,MUS,LNG,BLD,LIV,BRST,MUS,MUS,BLD,BRN,SKIN,LNG		B,Nrf1,TATA,VDR,Zic,p300		
1	183023890	0.94	0.97	rs10911194	C	T	0.58	0.55	ESC, ESDR, LNG, IPSC, FAT, STRM, BRST, BLD, MUS, BRN, SKIN, VAS, LIV, GI, ADRL, HRT, KID, PANC, PLCNT, THYM, OVRY, SPLN, CRVX, BONE	ESC, ESDR, IPSC, BLD, SKIN, FAT, LIV, BRN, GI, THYM, LNG, PLCNT, MUS, SPLN	ESC,ESDR,ESDR,ESDR,ESDR,ESC,IPSC,BRST,BLD,BLD,SKIN,SKIN,SKIN,SKIN,ADRL,HRT,GI,KID,MUS,MUS,PLCNT,GI,THYM,GI,OVRY,MUS,GI,LNG,BLD,CRVX,LIV,BRST,MUS,BRN,SKIN,LNG	POL2,ERALPHA_A,NFKB,POL24H8,AP2ALPHA,AP2GAMMA,BAF155,E2F6,HAE2F1	AP-1,ZBTB7A	LAMC1	synonymous
1	183024303	0.94	0.97	rs10911195	T	C	0.58	0.55	ESC, ESDR, LNG, IPSC, FAT, STRM, BRST, BLD, MUS, BRN, SKIN, VAS, LIV, GI, ADRL, HRT, KID, PANC, PLCNT, THYM, OVRY, SPLN, CRVX, BONE	ESC, ESDR, BRST, BLD, STRM, BRN, SKIN, FAT, LIV, GI, ADRL, HRT, LNG, PLCNT, THYM, MUS, SPLN, CRVX, VAS	ESC,ESDR,ESDR,ESDR,ESDR,ESC,BLD,BLD,SKIN,SKIN,ADRL,BRN,HRT,GI,GI,LNG,MUS,PLCNT,GI,OVRY,GI,LNG,BLD,CRVX,LIV,BRST,MUS,MUS,VAS,SKIN,LNG	BAF155	Dobox4	LAMC1	intronic
1	183024616	0.94	0.97	rs10752888	A	G	0.58	0.55	ESC, ESDR, LNG, IPSC, FAT,	ESC, ESDR, IPSC, BRST, BLD, STRM, BRN, SKIN, FAT, LIV, GI,	ESDR,SKIN,SKIN,HRT,	PRDM1	Ik-1,Maf,PPAR,Pou3	LAMC1	intronic

									STRM, BRST, BLD, MUS, BRN, SKIN, VAS, LIV, GI, ADRL, HRT, KID, PANC, PLCNT, THYM, OVRY, SPLN, CRVX, BONE	ADRL, HRT, KID, PANC, LNG, PLCNT, THYM, MUS, SPLN, CRVX, VAS	LNG,MUS,MUS,PLCNT,GI,CRVX,MUS,VAS		f1,RREB-1,ZBTB7A,Zfp740		
1	183024825	0.94	0.97	rs4652762	T	C	0.58	0.55	ESC, ESDR, LNG, IPSC, FAT, STRM, BRST, BLD, MUS, BRN, SKIN, VAS, LIV, GI, ADRL, HRT, KID, PANC, PLCNT, THYM, OVRY, SPLN, CRVX, BONE	ESC, ESDR, IPSC, BRST, BLD, STRM, BRN, SKIN, FAT, LIV, GI, ADRL, HRT, KID, PANC, LNG, PLCNT, THYM, MUS, SPLN, CRVX, VAS	ESDR,IPSC,BRST,SKIN,ADRL,HRT,GI,KID,LNG,MUS,MUS,THYM,OVRY,LNG,BLD,CRVX,BRST,MUS,VAS,SKIN,LNG		CTCF,HDAC2	LAMC1	intronic
1	183024949	0.94	0.97	rs4652763	A	G	0.58	0.55	ESC, ESDR, LNG, IPSC, FAT, STRM, BRST, BLD, MUS, BRN, SKIN, VAS, LIV, GI, ADRL, HRT, KID, PANC, PLCNT, THYM, OVRY, CRVX, BONE	ESC, ESDR, IPSC, BRST, BLD, STRM, BRN, SKIN, FAT, LIV, GI, ADRL, HRT, KID, PANC, LNG, PLCNT, MUS, SPLN, CRVX, VAS	ESDR,ESDR,SKIN,SKIN,HRT,MUS,MUS,OVRY,LNG,BLD,CRVX,BRST,MUS,VAS			LAMC1	intronic
1	183024980	0.94	0.97	rs4652764	G	A	0.58	0.55	ESC, ESDR, LNG, IPSC, FAT, STRM, BRST, BLD, MUS, BRN, SKIN, VAS, LIV, GI, ADRL, HRT, KID, PANC, PLCNT, THYM, OVRY, CRVX, BONE	ESC, ESDR, IPSC, BRST, BLD, STRM, BRN, SKIN, FAT, LIV, GI, ADRL, HRT, KID, PANC, LNG, PLCNT, MUS, SPLN, CRVX, VAS	ESDR,SKIN,SKIN,HRT,MUS,MUS,BLD,CRVX,BRST			LAMC1	intronic
1	183025012	0.94	0.97	rs4652765	C	T	0.58	0.55	ESC, ESDR, LNG, IPSC, FAT, STRM, BRST, BLD, MUS, BRN, SKIN, VAS, LIV, GI,	ESC, ESDR, IPSC, BRST, BLD, STRM, BRN, SKIN, FAT, LIV, GI, ADRL, HRT, KID, PANC, LNG, PLCNT, MUS, SPLN, CRVX, VAS	ESDR,SKIN,HRT,CRVX,BRST,VAS,SKIN		Foxa,Foxc1,Foxi1,HDAC2,Nanog,Pou2f2,TATA,TCF12	LAMC1	intronic

									ADRL, HRT, KID, PANC, PLCNT, THYM, OVRY, CRVX, BONE						
1	183025145	0.94	0.97	rs11322326	TC	T	0.58	0.55	ESC, ESDR, LNG, IPSC, FAT, STRM, BRST, BLD, MUS, BRN, SKIN, VAS, LIV, GI, ADRL, HRT, KID, PANC, PLCNT, THYM, OVRY, CRVX, BONE	ESC, ESDR, IPSC, BRST, BLD, STRM, BRN, SKIN, FAT, LIV, GI, ADRL, HRT, KID, PANC, LNG, PLCNT, MUS, CRVX, VAS	ESDR,HRT,MUS,MUS,OVRY,CRVX,BRST,MUS,MUS,SKIN		AP-2,GR,HNF1,Smad	LAMC1	intronic
1	183025165	0.94	0.97	rs4651137	T	C	0.58	0.55	ESC, ESDR, LNG, IPSC, FAT, STRM, BRST, BLD, MUS, BRN, SKIN, VAS, LIV, GI, ADRL, HRT, KID, PANC, PLCNT, THYM, OVRY, CRVX, BONE	ESC, ESDR, IPSC, BRST, BLD, STRM, BRN, SKIN, FAT, LIV, GI, ADRL, HRT, KID, PANC, LNG, PLCNT, MUS, CRVX, VAS	HRT,MUS,MUS,OVRY,CRVX,BRST,MUS,MUS,SKIN		AP-2,CEBPB,COMP1,Esr2,GR	LAMC1	intronic
1	183025201	0.94	0.97	rs4652766	A	G	0.58	0.55	ESC, ESDR, LNG, IPSC, FAT, STRM, BRST, BLD, MUS, BRN, SKIN, VAS, LIV, GI, ADRL, HRT, KID, PANC, PLCNT, THYM, OVRY, CRVX, BONE	ESC, ESDR, IPSC, BRST, BLD, STRM, BRN, SKIN, FAT, LIV, GI, ADRL, HRT, KID, PANC, LNG, PLCNT, MUS, CRVX, VAS	HRT,MUS,MUS,CRVX,MUS,MUS,SKIN		CEBPB,Mef2,Pou5f1	LAMC1	intronic
1	183025333	0.94	0.97	rs66475151	TA	T	0.58	0.55	ESC, ESDR, LNG, IPSC, FAT, STRM, BRST, BLD, MUS, BRN, SKIN, VAS, LIV, GI, ADRL, HRT, KID, PANC, PLCNT, THYM, OVRY, CRVX,	ESC, ESDR, IPSC, BRST, BLD, STRM, BRN, SKIN, FAT, LIV, GI, ADRL, HRT, KID, PANC, LNG, PLCNT, MUS, CRVX, VAS			GR,Nkx2,Nkx3,Sox	LAMC1	intronic

									BONE						
1	183026098	0.94	0.97	rs10911196	G	A	0.58	0.55	ESC, ESDR, LNG, IPSC, FAT, STRM, BRST, BLD, MUS, BRN, SKIN, VAS, LIV, GI, ADRL, HRT, KID, PANC, PLCNT, THYM, OVRY, CRVX, BONE	ESC, ESDR, IPSC, FAT, BRST, BLD, STRM, MUS, BRN, SKIN, LIV, GI, ADRL, HRT, KID, PANC, LNG, OVRY, PLCNT, CRVX, VAS, BONE	CRVX,BRST	EBF1,KAP1,P300,RAD21,CTCF	Cdx2,TATA	LAMC1	intronic
1	183026264	0.94	0.97	rs10911197	T	C	0.58	0.55	ESC, ESDR, LNG, IPSC, FAT, STRM, BRST, BLD, MUS, BRN, SKIN, VAS, LIV, GI, ADRL, HRT, KID, PANC, THYM, OVRY, PLCNT, CRVX, BONE	ESC, ESDR, IPSC, FAT, BRST, BLD, STRM, MUS, BRN, SKIN, GI, ADRL, HRT, KID, PANC, LNG, OVRY, PLCNT, CRVX, LIV, VAS, BONE	CRVX,BRST,BRN		CTCF	LAMC1	intronic
1	183026473	0.94	0.97	rs61081633	A	G	0.58	0.55	ESC, ESDR, LNG, IPSC, FAT, STRM, BRST, BLD, MUS, BRN, SKIN, VAS, LIV, GI, ADRL, HRT, KID, PANC, OVRY, PLCNT, CRVX, BONE	ESC, ESDR, IPSC, FAT, STRM, BRST, BLD, MUS, BRN, SKIN, GI, ADRL, HRT, KID, PANC, LNG, OVRY, PLCNT, CRVX, LIV, VAS, BONE	HRT,GI,KID,MUS,BLD,VAS	TBP,KAP1	Rad21,SP1	LAMC1	intronic
1	183027107	0.94	0.97	rs4642853	C	T	0.58	0.55	ESC, ESDR, LNG, IPSC, FAT, STRM, BRST, MUS, BRN, SKIN, VAS, GI, HRT, KID, PANC, OVRY, PLCNT, BLD, BONE	ESC, ESDR, LNG, IPSC, FAT, STRM, BRST, BLD, MUS, BRN, SKIN, GI, ADRL, HRT, KID, PANC, OVRY, PLCNT, CRVX, LIV, VAS, BONE	BLD,MUS		Tel2	LAMC1	intronic
1	183027156	0.94	0.97	rs4369195	G	C	0.58	0.55	ESC, ESDR, LNG, IPSC, FAT, STRM, BRST, MUS, BRN, SKIN, VAS, GI, HRT, KID,	ESC, ESDR, LNG, IPSC, FAT, STRM, BRST, BLD, MUS, BRN, SKIN, GI, ADRL, HRT, KID, PANC, OVRY, PLCNT, CRVX, LIV, VAS, BONE	BLD,MUS,MUS,VAS,BRN,LNG		Mef2,NRSF,Pax-4,Rhox11	LAMC1	intronic

									OVRY, PLCNT, BLD, BONE					
1	183027287	0.94	0.97	rs113711531	A	ACT	0.58	0.55	ESC, ESDR, LNG, IPSC, FAT, STRM, BRST, MUS, BRN, SKIN, VAS, GI, HRT, KID, OVRY, PLCNT, BLD, BONE	ESC, ESDR, LNG, IPSC, FAT, STRM, BRST, BLD, MUS, BRN, SKIN, GI, ADRL, HRT, KID, PANC, OVRY, PLCNT, CRVX, LIV, VAS, BONE	ESDR		Mef2,STAT,Sox	LAMC1	intronic
1	183027289	0.84	0.97	rs5779150	T	TC,TCT	0.53	0.52	ESC, ESDR, LNG, IPSC, FAT, STRM, BRST, MUS, BRN, SKIN, VAS, GI, HRT, KID, OVRY, PLCNT, BLD, BONE	ESC, ESDR, LNG, IPSC, FAT, STRM, BRST, BLD, MUS, BRN, SKIN, GI, ADRL, HRT, KID, PANC, OVRY, PLCNT, CRVX, LIV, VAS, BONE	ESDR			LAMC1	intronic
1	183027692	0.94	0.97	rs10797815	A	G	0.58	0.55	ESC, ESDR, LNG, IPSC, FAT, STRM, BRST, MUS, BRN, SKIN, VAS, GI, HRT, KID, OVRY, PLCNT, BLD, BONE	ESC, ESDR, LNG, IPSC, FAT, STRM, BRST, BLD, MUS, BRN, SKIN, GI, ADRL, HRT, KID, PANC, OVRY, PLCNT, CRVX, LIV, VAS, BONE	ESDR		E2F,Nr2f2,Pou2f2,Pou5f1	LAMC1	intronic
1	183028149	0.82	0.96	rs200189406	TTCC	T	0.54	0.52	ESC, ESDR, IPSC, FAT, STRM, BRST, MUS, BRN, SKIN, VAS, GI, HRT, KID, LNG, OVRY, PLCNT, BLD, BONE	ESC, ESDR, LNG, IPSC, FAT, STRM, BRST, BLD, MUS, BRN, SKIN, VAS, GI, ADRL, HRT, KID, PANC, OVRY, PLCNT, CRVX, LIV, BONE	ESDR,SKIN,SKIN		AP-1,ATF3,BCL,CHD2,EWSR1-FLI1,Egr-1,Ets,MAZR,MZF1::1-4,Myc,Pou2f2,SP1,SRF,STAT,Sp4,Spz1,TEF-1,TFII-I,UF1H3BETA,WT1,YY1,ZNF219,ZNF263,Zfp281,Zfp740,Znf143	LAMC1	intronic
1	183028151	0.91	0.97	rs34099733	CCT	C	0.56	0.54	ESC, ESDR, IPSC, FAT, STRM, BRST, MUS, BRN, SKIN, VAS, GI, HRT, KID, LNG, OVRY, PLCNT, BLD, BONE	ESC, ESDR, LNG, IPSC, FAT, STRM, BRST, BLD, MUS, BRN, SKIN, VAS, GI, ADRL, HRT, KID, PANC, OVRY, PLCNT, CRVX, LIV, BONE	SKIN,SKIN		AP-1,ATF3,BCL,CHD2,EWSR1-FLI1,Egr-1,Ets,MAZR,MZF1::1-4,Myc,NRSF,Pou2f2,	LAMC1	intronic

													SP1,SRF,STAT,Sp4,Spz1,TEF-1,TFII-I,UF1H3BETA,WT1,YY1,ZNF219,ZNF263,Zfp281,Zfp740,Znf143		
1	183028183	0.94	0.97	rs10911198	G	T	0.58	0.55	ESC, ESDR, IPSC, FAT, STRM, BRST, MUS, BRN, SKIN, VAS, GI, HRT, KID, LNG, OVRY, PLCNT, BLD, BONE	ESC, ESDR, LNG, IPSC, FAT, STRM, BRST, BLD, MUS, BRN, SKIN, VAS, GI, ADRL, HRT, KID, PANC, OVRY, PLCNT, CRVX, LIV, BONE			AIRE,AP-1,DMRT4,DMRT7,Irf,NF-Y,SP1,SP2,Sin3Ak-20,TATA	LAMC1	intronic
1	183028626	0.94	0.97	rs5022057	T	C	0.58	0.55	ESC, ESDR, IPSC, FAT, STRM, BRST, MUS, BRN, SKIN, VAS, GI, HRT, KID, LNG, OVRY, PLCNT, BLD, BONE	ESC, ESDR, LNG, IPSC, FAT, STRM, BRST, MUS, BRN, SKIN, VAS, GI, ADRL, HRT, KID, PANC, THYM, OVRY, PLCNT, BLD, CRVX, LIV, BONE			EBF,TCF12	LAMC1	intronic
1	183028901	0.94	0.97	rs10911199	T	A	0.58	0.55	ESC, ESDR, IPSC, FAT, STRM, BRST, MUS, BRN, SKIN, VAS, GI, HRT, LNG, BLD, BONE	ESC, ESDR, LNG, IPSC, FAT, STRM, BRST, MUS, BRN, SKIN, VAS, GI, ADRL, HRT, KID, PANC, THYM, OVRY, PLCNT, BLD, CRVX, LIV, BONE			Zfp105	LAMC1	intronic
1	183029351	0.94	0.97	rs10797816	A	T	0.58	0.55	ESC, ESDR, IPSC, FAT, STRM, BRST, MUS, BRN, SKIN, VAS, GI, HRT, LNG, BLD, BONE	ESC, ESDR, LNG, IPSC, FAT, STRM, BRST, MUS, BRN, SKIN, VAS, LIV, GI, ADRL, HRT, KID, PANC, OVRY, PLCNT, SPLN, BLD, CRVX, BONE			RFX5	LAMC1	intronic
1	183030001	0.94	0.97	rs10797817	G	T	0.58	0.55	ESC, ESDR, IPSC, FAT, STRM, BRST, MUS, BRN, SKIN, VAS, GI, HRT, LNG, BLD, BONE	ESC, ESDR, LNG, IPSC, FAT, STRM, BRST, BLD, MUS, BRN, SKIN, VAS, LIV, GI, ADRL, HRT, KID, PANC, OVRY, PLCNT, SPLN, CRVX, BONE	ESDR,ESDR,BRST,SKIN,SKIN,SKIN,CRVX,MUS,VAS,SKIN	CFOS,GATA2	LBP-9,Mef2	LAMC1	intronic
1	183030906	0.94	0.97	rs12037585	G	T	0.58	0.55	ESC, FAT, STRM, BRST, MUS, SKIN, VAS, GI, HRT, LNG, PLCNT, BLD, BRN	ESC, ESDR, LNG, IPSC, FAT, STRM, BRST, MUS, BRN, SKIN, VAS, GI, HRT, KID, PANC, OVRY, PLCNT, BLD, CRVX, LIV, BONE			STAT	LAMC1	intronic
1	183031013	0.94	0.97	rs12037623	G	A	0.58	0.55	ESC, FAT, STRM, BRST,	ESC, ESDR, LNG, IPSC, FAT, STRM, BRST, MUS, BRN, SKIN,			Dbx1,Foxa,Foxj1,Fo	LAMC1	intronic

									MUS, SKIN, VAS, GI, HRT, LNG, PLCNT, BLD, BRN	VAS, GI, HRT, KID, PANC, OVRY, PLCNT, BLD, CRVX, LIV, BONE			xj2,Foxk1,Foxp1,Pou1f1,Pou2f2,TATA,Zfp105		
1	183032177	0.94	0.97	rs4651138	C	A	0.58	0.55	ESC, FAT, STRM, BRST, MUS, SKIN, GI, HRT, BLD	ESC, ESDR, LNG, IPSC, FAT, STRM, BRST, BLD, MUS, BRN, SKIN, VAS, GI, ADRL, HRT, KID, PANC, THYM, OVRY, PLCNT, CRVX, LIV, BONE			Egr-1	LAMC1	intronic
1	183032487	0.94	0.97	rs10911201	G	T	0.58	0.55	ESC, FAT, STRM, BRST, MUS, SKIN, GI, HRT, BLD	ESC, ESDR, LNG, IPSC, FAT, STRM, BRST, BLD, MUS, BRN, SKIN, VAS, GI, ADRL, HRT, KID, PANC, THYM, OVRY, PLCNT, CRVX, LIV, BONE		ERALPHA_A	Arid3a,Hbp1,Pou2f2,Pou3f2,Sox	LAMC1	intronic
1	183033348	0.94	0.97	rs4652767	G	C	0.58	0.55	FAT, STRM, BRST, SKIN, GI, BLD	ESC, ESDR, LNG, IPSC, FAT, STRM, BRST, BLD, MUS, BRN, SKIN, VAS, GI, ADRL, PANC, THYM, OVRY, PLCNT, SPLN, LIV, BONE	ESDR,BRST,BLD,BLD,SKIN,THYM,BLD,SKIN	BCL11A,EBF1,OCT2,PAX5C20,PAX5N19,POU2F2	AP-1,Ets,Hsf,Myf	LAMC1	intronic
1	183034301	0.94	0.97	rs10752889	C	T	0.58	0.55	FAT, STRM, SKIN, GI	ESC, ESDR, LNG, IPSC, FAT, BRST, BLD, STRM, BRN, SKIN, VAS, GI, OVRY, PLCNT, MUS, THYM, SPLN, LIV				LAMC1	intronic
1	183034517	0.93	0.97	rs6657533	C	T	0.58	0.54	FAT, STRM, SKIN	ESC, ESDR, LNG, IPSC, FAT, BRST, BLD, STRM, BRN, SKIN, VAS, GI, OVRY, PLCNT, MUS, THYM, LIV			NRSF,Rad21,YY1,Zfx	LAMC1	intronic
1	183035412	0.94	0.97	rs4129857	C	T	0.58	0.55	FAT, STRM	ESC, ESDR, IPSC, FAT, BRST, BLD, SKIN, VAS, BRN, GI, LNG, OVRY, PLCNT, MUS, HRT				LAMC1	intronic
1	183036646	0.94	0.97	rs10797818	T	C	0.58	0.55	GI	ESC, ESDR, IPSC, FAT, BRST, BLD, SKIN, VAS, BRN, GI, LNG, MUS, PLCNT, OVRY, HRT			Ik-1,Ik-2,NRSF,Rad21,STAT,Sin3Ak-20,YY1,Zfx	LAMC1	intronic
1	183037776	0.94	0.97	rs10797819	A	G	0.58	0.55	ESC, IPSC, FAT, BRST, SKIN, GI, OVRY	ESC, ESDR, LNG, IPSC, FAT, STRM, BRST, BLD, MUS, BRN, SKIN, VAS, GI, ADRL, HRT, KID, PLCNT, LIV, BONE			CDP,Obox6,SETDB1	LAMC1	intronic
1	183038556	0.9	0.97	rs34812197	T	TCCC	0.57	0.54	ESC, FAT, STRM, BRST, MUS, SKIN, GI, OVRY, BRN	ESC, ESDR, LNG, IPSC, FAT, STRM, BRST, BLD, MUS, BRN, SKIN, VAS, LIV, GI, ADRL, HRT, KID, OVRY, PLCNT, BONE	BRN,SKIN		Bcl6b,HNF1,Hdx,Ik-2,Pou2f2,STAT	LAMC1	intronic
1	183040061	0.94	0.97	rs4652769	T	C	0.58	0.55	ESC, ESDR, IPSC, FAT, STRM,	ESC, ESDR, LNG, IPSC, FAT, STRM, BRST, BLD, MUS, BRN,	ESDR,LNG,BRST,SKIN	EBF1,KAP1	Evi-1,Hmbox1	LAMC1	intronic

									BRST, BLD, MUS, BRN, SKIN, GI, HRT, OVRY, VAS, BONE	SKIN, VAS, LIV, GI, ADRL, HRT, KID, PANC, PLCNT, THYM, OVRY, SPLN, BONE	,SKIN,SKIN,MUS,BLD,MUS,MUS,VAS,BLD,BRN,SKIN,LNG				
1	183042231	0.94	0.97	rs10797820	G	A	0.58	0.55	FAT, STRM, BRST, SKIN, GI	ESC, ESDR, LNG, IPSC, FAT, STRM, BRST, BLD, MUS, BRN, SKIN, VAS, LIV, GI, HRT, KID, OVRY, PANC, PLCNT, BONE			SIX5	LAMC1	intronic
1	183043288	0.94	0.97	rs10911206	A	G	0.58	0.55	FAT, STRM, BRST, MUS, SKIN, GI, HRT, OVRY, LNG, BRN, BONE	ESDR, ESC, LNG, IPSC, FAT, STRM, BRST, BLD, MUS, BRN, SKIN, VAS, LIV, GI, HRT, PANC, OVRY, CRVX, BONE	HRT		Myc	LAMC1	intronic
1	183043424	0.94	0.97	rs10797821	G	A	0.58	0.55	FAT, STRM, MUS, SKIN, GI, HRT, OVRY, LNG, BRN, BONE	ESDR, ESC, LNG, IPSC, FAT, STRM, BRST, MUS, BRN, SKIN, LIV, GI, HRT, PANC, CRVX, VAS, BONE			ATF2,CEBPB,Cdc5,Cphx,Duxl,E4BP4,Evi-1,Foxd1,Gmeb1,Hltf,Hlx1,p300	LAMC1	intronic
1	183044853	0.94	0.97	rs10911209	T	G	0.57	0.55	FAT, STRM, MUS, SKIN, VAS, GI, HRT, OVRY, LNG, BLD, BRN, BONE	ESC, ESDR, LNG, IPSC, FAT, STRM, BRST, BLD, MUS, BRN, SKIN, LIV, GI, ADRL, HRT, KID, PANC, OVRY, CRVX, VAS, BONE	ESDR,SKIN,MUS,MUS,BRN			LAMC1	intronic
1	183045154	0.94	0.97	rs6660111	G	A	0.58	0.55	FAT, STRM, MUS, VAS, GI, HRT, OVRY, LNG, BLD, BRN, SKIN, BONE	ESDR, ESC, LNG, IPSC, FAT, STRM, BRST, BLD, MUS, BRN, SKIN, LIV, GI, ADRL, HRT, KID, PANC, CRVX, VAS, BONE	ESDR		Pou2f2,Sox,TATA	LAMC1	intronic
1	183048210	0.94	0.97	rs6664995	T	C	0.58	0.55	FAT, STRM, GI, HRT, BLD	ESDR, ESC, FAT, STRM, BRST, BLD, MUS, BRN, SKIN, GI, HRT, LNG, VAS, BONE			ZEB1	LAMC1	intronic
1	183048634	0.94	0.97	rs10797822	A	G	0.58	0.55	FAT, BLD, HRT	ESDR, FAT, STRM, BRST, BLD, MUS, BRN, SKIN, GI, HRT, LNG, VAS, BONE	BLD		Mxi1	LAMC1	intronic
1	183049722	0.94	0.97	rs6668980	T	C	0.57	0.55		ESDR, LNG, FAT, STRM, BRST, BLD, SKIN, GI, HRT, OVRY, MUS			AP-1	LAMC1	intronic
1	183050573	0.94	0.97	rs61804970	T	C	0.58	0.55		ESDR, LNG, FAT, BRST, BLD, SKIN, GI, HRT, PLCNT, OVRY, MUS			Sin3Ak-20	LAMC1	intronic

1	183050747	0.93	0.97	rs28477876	T	C	0.58	0.54	SPLN	ESDR, LNG, FAT, BRST, BLD, SKIN, GI, HRT, MUS, PLCNT, OVRY, PANC			Mef2,NRSF	LAMC1	intronic
1	183051627	0.94	0.97	rs4422969	G	A	0.58	0.55	ESDR, SKIN, GI, HRT, MUS, SPLN	ESDR, ESC, LNG, IPSC, FAT, STRM, BRST, BLD, SKIN, VAS, BRN, GI, HRT, MUS, PLCNT, OVRY, PANC, SPLN, CRVX, BONE	HRT,MUS		Foxa,SIX5,Znf143	LAMC1	intronic
1	183052366	0.94	0.97	rs10797823	A	G	0.58	0.55	GI, SPLN	ESDR, ESC, IPSC, FAT, STRM, BRST, BLD, SKIN, VAS, BRN, GI, HRT, LNG, MUS, OVRY, PANC, BONE				LAMC1	intronic
1	183052775	0.94	0.97	rs10752890	A	G	0.58	0.55	GI	ESDR, ESC, IPSC, FAT, STRM, BRST, BLD, SKIN, BRN, GI, LNG, MUS, OVRY, HRT, VAS, BONE			Dbx1,Ncx,Nkx2	LAMC1	intronic
1	183054148	0.93	0.97	rs10752891	G	A	0.58	0.55	BRN	ESDR, IPSC, FAT, STRM, BRST, SKIN, VAS, BRN, GI, LNG, PANC, PLCNT, MUS, HRT, BONE		ZNF263,SETDB1		LAMC1	intronic
1	183054368	0.93	0.97	rs4652771	G	A	0.58	0.55	BRN	ESDR, LNG, IPSC, FAT, STRM, BRST, MUS, SKIN, VAS, GI, HRT, OVRY, PANC, PLCNT, BLD, BRN, BONE		ZNF263,SETDB1		LAMC1	intronic
1	183054561	0.93	0.97	rs4652772	G	C	0.58	0.55		ESDR, LNG, IPSC, FAT, STRM, BRST, MUS, SKIN, VAS, GI, HRT, OVRY, PANC, PLCNT, BLD, BRN, BONE		SETDB1	GATA,SZF1-1,VDR	LAMC1	intronic
1	183055334	0.93	0.97	rs10911212	C	T	0.58	0.55		ESDR, LNG, IPSC, FAT, STRM, BRST, MUS, SKIN, GI, HRT, OVRY, PANC, PLCNT, BLD, BRN, BONE	BRST,SKIN,SKIN,MUS,CRVX,BRST,SKIN,LNG		BAF155,SIX5,Znf143	LAMC1	intronic
1	183055446	0.93	0.97	rs10797825	G	A	0.58	0.55		ESDR, LNG, IPSC, FAT, STRM, BRST, MUS, SKIN, GI, HRT, OVRY, PANC, PLCNT, BLD, BRN, BONE	SKIN		Hoxa3,Hoxa4,Mef2,Pax-4,Zfp105	LAMC1	intronic
1	183056168	0.93	0.97	rs10737238	A	G	0.58	0.55	SKIN	ESC, ESDR, LNG, IPSC, FAT, STRM, BRST, MUS, BRN, SKIN, VAS, GI, ADRL, HRT, OVRY, PANC, PLCNT, SPLN, BLD, BONE		ZNF143		LAMC1	intronic
1	183056763	0.93	0.97	rs10732271	C	T	0.58	0.55	SKIN, GI	ESC, ESDR, LNG, IPSC, FAT, STRM, BRST, MUS, BRN, SKIN, VAS, GI, ADRL, OVRY, PLCNT, HRT, BONE	ESDR		DMRT5,NF-I,STAT,ZEB1	LAMC1	intronic
1	183058081	0.91	0.97	rs3934578	T	C	0.58	0.55	FAT, GI, PLCNT	ESC, ESDR, LNG, IPSC, FAT, STRM, BRST, MUS, BRN, SKIN, GI, PLCNT, HRT, VAS, BONE			Foxa,Foxj2,Foxl1,GATA,Irf,Pou1f1,TATA,	LAMC1	intronic

													p300		
1	183058297	0.93	0.97	rs3934579	G	A	0.58	0.55	FAT, GI, PLCNT	ESC, ESDR, LNG, IPSC, FAT, STRM, BRST, MUS, BRN, SKIN, VAS, GI, PANC, OVRY, PLCNT, BONE			AP-1	LAMC1	intronic
1	183058377	0.93	0.97	rs3934697	A	G	0.58	0.55	FAT, GI, PLCNT	ESC, ESDR, LNG, IPSC, FAT, STRM, BRST, MUS, BRN, SKIN, VAS, GI, PANC, OVRY, PLCNT, CRVX, BONE			SRF	LAMC1	intronic
1	183059720	0.93	0.97	rs4072709	C	T	0.58	0.55	FAT, GI, MUS, LNG, BONE	ESC, ESDR, LNG, IPSC, FAT, STRM, BRST, MUS, BRN, SKIN, VAS, GI, HRT, PANC, PLCNT, OVRY, BLD, CRVX, LIV, BONE	BLD		NF-I	LAMC1	intronic
1	183059835	0.93	0.97	rs3935384	C	T	0.58	0.55	FAT, GI, MUS, LNG, BONE	ESC, ESDR, LNG, IPSC, FAT, STRM, BRST, MUS, BRN, SKIN, VAS, GI, HRT, PANC, PLCNT, OVRY, BLD, CRVX, LIV, BONE			Ncx,Nkx2,Nkx3	LAMC1	intronic
1	183059935	0.93	0.97	rs4266836	T	G	0.58	0.55	FAT, GI, MUS, LNG, BONE	ESC, ESDR, LNG, IPSC, FAT, STRM, BRST, MUS, BRN, SKIN, VAS, GI, HRT, PANC, PLCNT, OVRY, BLD, CRVX, LIV, BONE	VAS		Hand1,Smad3	LAMC1	intronic
1	183061035	0.92	0.96	rs10737239	A	C	0.58	0.55	FAT, STRM, GI, MUS, LNG, BONE	ESC, ESDR, LNG, IPSC, FAT, STRM, BRST, MUS, BRN, SKIN, GI, ADRL, HRT, PANC, PLCNT, OVRY, BLD, CRVX, LIV, VAS, BONE	ESDR,MUS,PLCNT,MUS,CRVX		Nkx3,Pou2f2,Pou3f2	LAMC1	intronic
1	183062506	0.92	0.96	rs7547953	T	G	0.58	0.55	FAT, GI	ESDR, LNG, IPSC, FAT, STRM, BRST, MUS, BRN, SKIN, GI, KID, PANC, HRT, OVRY, CRVX, VAS, BONE			HNF1,Hoxa4,Klf7,NRSF,Pax-4,Pou2f2,TATA	LAMC1	intronic
1	183064093	0.92	0.96	rs10797829	A	G	0.58	0.55		ESC, LNG, IPSC, FAT, STRM, BRST, MUS, BRN, SKIN, VAS, GI, KID, PANC, HRT, BONE			PPAR,Sin3Ak-20	LAMC1	intronic
1	183066920	0.92	0.96	rs10752892	A	G	0.58	0.55		SKIN, FAT, MUS			CEBPG,DMRT3,DMRT5,Foxp3,TFIIA	LAMC1	intronic
1	183067139	0.92	0.96	rs10797830	A	T	0.58	0.55		IPSC, BRST, SKIN, FAT, LNG			DMRT2,GR,HNF1,Ik-2,Mef2,Sox	LAMC1	intronic
1	183067520	0.85	0.96	rs10911217	G	A	0.58	0.53		IPSC, FAT, BRST, SKIN, VAS, GI, LNG, BONE			ERalpha-a,TCF11::MafG,ZID	LAMC1	intronic
1	183067681	0.92	0.96	rs10797831	A	G	0.58	0.55	GI	ESDR, IPSC, FAT, BRST, SKIN, VAS, GI, HRT, OVRY, LNG,			GATA,Lmo2-comple	LAMC1	intronic

										BONE			x,SIX5		
1	183068242	0.85	0.96	rs6697320	T	G	0.58	0.53	GI	ESDR, ESC, IPSC, FAT, BRST, MUS, SKIN, GI, HRT, LNG, BLD, CRVX, LIV, BONE			FAC1,Foxa,Foxp1,HDAC2,RREB-1,Sox,Zfp105	LAMC1	intronic
1	183068668	0.92	0.96	rs7515822	A	G	0.58	0.55	GI	ESDR, ESC, FAT, STRM, BRST, MUS, BRN, SKIN, GI, HRT, OVRY, LNG, BLD, CRVX, LIV, BONE			Egr-1,Ets,Osr,Zfp161	LAMC1	intronic
1	183072955	0.92	0.96	rs6665685	T	A	0.58	0.55	ESC, FAT, STRM, MUS, SKIN, GI, LNG	ESC, ESDR, LNG, IPSC, FAT, STRM, BRST, BLD, MUS, BRN, SKIN, VAS, LIV, GI, ADRL, HRT, KID, PANC, PLCNT, THYM, OVRY, BONE			AP-3,HNF1,PLZF	LAMC1	intronic
1	183074899	0.92	0.96	rs10797832	A	G	0.58	0.55	FAT, MUS, GI	ESC, ESDR, LNG, FAT, STRM, BRST, MUS, BRN, SKIN, GI, HRT, PANC, PLCNT, BLD, LIV, BONE			Dobox4,Foxp1,TATA	LAMC1	intronic
1	183074932	0.92	0.96	rs10797833	C	T	0.58	0.55	FAT, MUS, GI	ESC, ESDR, LNG, FAT, STRM, BRST, MUS, BRN, SKIN, GI, HRT, PANC, PLCNT, BLD, LIV, BONE			Elf5,Ets,GATA,HDAC2,Hoxa10,Hoxa5,Hoxa9,Hoxb9,Mef2,PU.1,RXRA,STAT,Tel2,ZNF263,p300	LAMC1	intronic
1	183077602	0.85	0.96	rs10911221	G	C	0.58	0.53	FAT, MUS, SKIN	ESC, ESDR, LNG, IPSC, FAT, STRM, BRST, MUS, SKIN, VAS, BRN, GI, HRT, PANC, OVRY, PLCNT, CRVX, BONE			TCF11::MafG	LAMC1	intronic
1	183078228	0.92	0.96	rs10911222	G	A	0.57	0.55	FAT, SKIN, MUS	ESDR, ESC, LNG, FAT, STRM, BRST, MUS, SKIN, GI, HRT, PANC, OVRY, PLCNT, CRVX, BRN, BONE	SKIN,SKIN		AFP1,Foxa,HNF4,Isl2,Nkx2,Nkx6-1,Pou3f4,Pou4f3,Prrx2,RXR::LXR,Sox	LAMC1	intronic
1	183078949	0.9	0.96	rs10911223	T	C	0.57	0.54	FAT, SKIN, HRT, MUS, GI	ESDR, LNG, FAT, STRM, BRST, MUS, SKIN, LIV, GI, ADRL, HRT, PANC, OVRY, PLCNT, CRVX, BRN, BONE			GR	LAMC1	intronic
1	183079614	0.92	0.96	rs10797834	T	G	0.57	0.55	FAT, MUS, GI, BONE	ESDR, LNG, IPSC, FAT, STRM, BRST, BLD, MUS, SKIN, VAS, LIV, GI, ADRL, HRT, PANC, OVRY, PLCNT, SPLN, CRVX, BRN,			EBF	LAMC1	intronic

										BONE					
1	183080254	0.86	0.95	rs1889307	T	A	0.57	0.53	FAT, GI, LNG, BONE	ESDR, LNG, IPSC, FAT, STRM, BRST, BLD, MUS, SKIN, VAS, LIV, GI, ADRL, HRT, PANC, PLCNT, OVRY, SPLN, CRVX, BRN, BONE		RPC155	Foxp1,HDAC2,Irf,Zfp105,p300	LAMC1	intronic
1	183080952	0.92	0.96	rs12128323	G	A	0.58	0.55	FAT, BONE	ESDR, LNG, FAT, STRM, BRST, BLD, MUS, SKIN, VAS, GI, ADRL, PLCNT, HRT, OVRY, PANC, SPLN, CRVX, LIV, BONE	MUS,MUS		ATF4,CEBPA,GR,LBP-1,Nrf1	LAMC1	intronic
1	183081233	0.92	0.96	rs10911229	C	G	0.58	0.55	FAT, BONE	ESDR, LNG, FAT, STRM, BRST, BLD, MUS, BRN, SKIN, VAS, GI, ADRL, HRT, OVRY, PANC, PLCNT, CRVX, LIV, BONE			COMP1	LAMC1	intronic
1	183081475	0.91	0.96	rs10752894	A	G	0.56	0.54		ESDR, ESC, LNG, FAT, STRM, BRST, BLD, MUS, BRN, SKIN, VAS, GI, ADRL, HRT, OVRY, PANC, PLCNT, CRVX, LIV, BONE			E2A,HEN1,Myf,ZEB1	LAMC1	intronic
1	183081983	0.92	0.96	rs10911230	A	C	0.58	0.55		ESC, ESDR, LNG, IPSC, FAT, STRM, BRST, BLD, MUS, BRN, SKIN, LIV, GI, ADRL, HRT, OVRY, PLCNT, CRVX	SKIN		Cdx,Hlx1,TATA	LAMC1	intronic
1	183082367	0.92	0.96	rs35782443	A	C	0.58	0.55		ESC, ESDR, LNG, IPSC, FAT, STRM, BRST, BLD, BRN, SKIN, VAS, LIV, GI, ADRL, HRT, OVRY, PLCNT, MUS, CRVX	SKIN		Ik-1,Ik-3,Pax-8,Pou2f2,RBP-Jkappa,Spz1	LAMC1	intronic
1	183082479	0.92	0.96	rs12082051	C	T	0.58	0.55		ESC, ESDR, LNG, IPSC, FAT, STRM, BRST, BLD, BRN, SKIN, VAS, LIV, GI, ADRL, HRT, OVRY, PLCNT, MUS, CRVX	SKIN		TEF	LAMC1	intronic
1	183082610	0.92	0.96	rs10911231	T	A	0.58	0.55		ESC, ESDR, LNG, IPSC, FAT, STRM, BRST, BLD, BRN, SKIN, VAS, LIV, GI, ADRL, HRT, OVRY, PANC, PLCNT, MUS, CRVX			CACD	LAMC1	intronic
1	183083866	0.92	0.96	rs10911234	A	C	0.58	0.55	BLD	ESDR, ESC, IPSC, FAT, BRST, SKIN, GI, HRT, MUS, CRVX, LIV			HMG-IY,Ncx,Pou1f1,Pou2f2,Pou3f2,Pou5f1,Sox,TATA,p300	LAMC1	intronic
1	183083996	0.92	0.96	rs4397624	C	T	0.58	0.55	BLD	ESDR, ESC, IPSC, FAT, BRST, SKIN, GI, HRT, MUS, CRVX, LIV			AP-1,Foxa,Foxp1,NF-Y,Pbx3,RFX5,SP1,SP2,TATA	LAMC1	intronic
1	183084611	0.92	0.96	rs10911235	G	A	0.58	0.55	BLD	IPSC, FAT, STRM, BRST, SKIN, GI, HRT, LNG, MUS, LIV, BONE	SKIN,BLD		Evi-1,GATA,HDAC2	LAMC1	intronic

1	183085692	0.92	0.96	rs4652773	A	G	0.58	0.55	LNG	ESC, ESDR, IPSC, FAT, STRM, BRST, BLD, BRN, SKIN, GI, HRT, PANC, LNG, MUS, OVRY, PLCNT, LIV, BONE		USF1	AP-1,BCL,CHD2,E2F,Egr-1,Irf,Klf4,Klf7,MOVO-B,Myc,SP1,STAT,Spz1,TATA,WT1,ZNF219	LAMC1	intronic
1	183086263	0.92	0.96	rs2151669	T	G	0.58	0.55	LNG	ESC, ESDR, IPSC, FAT, STRM, BRST, BLD, BRN, SKIN, GI, HRT, PANC, LNG, PLCNT, MUS, LIV, BONE			Obox3,Obox6,Pitx3	LAMC1	intronic
1	183088798	0.92	0.96	rs2027086	G	A	0.58	0.55	FAT, BLD, GI, LNG, LIV	ESC, ESDR, LNG, IPSC, FAT, STRM, BRST, BRN, SKIN, VAS, LIV, GI, HRT, KID, PANC, THYM, PLCNT, MUS, BLD, CRVX, BONE			EWSR1-FLI1,Evi-1,Pax-4	LAMC1	intronic
1	183089763	0.92	0.96	rs6672306	G	A	0.58	0.55	FAT, GI, LNG, LIV	ESC, ESDR, LNG, IPSC, FAT, STRM, BRST, MUS, BRN, SKIN, VAS, LIV, GI, HRT, KID, PANC, PLCNT, THYM, BLD, CRVX, BONE			GR,PPAR,STAT,Zbtb12	LAMC1	intronic
1	183090937	0.81	0.95	rs12078729	T	A	0.58	0.57	FAT, BRN, SKIN, GI, HRT, LNG	ESC, ESDR, LNG, IPSC, FAT, STRM, BRST, BLD, MUS, BRN, SKIN, VAS, LIV, GI, HRT, KID, PANC, PLCNT, THYM, OVRY, CRVX, BONE	GI,LNG,MUS	KAP1	AP-1,HNF4,Hoxa10,Mef2,Nkx6-2,Pdx1	LAMC1	intronic
1	183093315	0.82	0.96	rs10911239	T	C	0.58	0.57	ESC, FAT, SKIN, HRT, GI, LNG	ESC, ESDR, LNG, IPSC, FAT, STRM, BRST, MUS, BRN, SKIN, VAS, GI, HRT, KID, PLCNT, OVRY, PANC, LIV, BONE	PANC		GATA,PLZF	LAMC1	intronic
1	183093813	0.82	0.96	rs10911240	T	C	0.58	0.57	ESC, FAT, SKIN, HRT, GI, LNG	ESC, ESDR, LNG, IPSC, FAT, STRM, BRST, MUS, BRN, SKIN, VAS, GI, HRT, KID, PLCNT, OVRY, PANC, CRVX, LIV, BONE	ESDR,LNG,SKIN,SKIN,SKIN,SKIN,MUS,BRN,SKIN			LAMC1	intronic
1	183094603	0.82	0.96	rs7542640	G	A	0.58	0.57	ESC, FAT, SKIN, HRT, LNG	ESC, ESDR, LNG, IPSC, FAT, STRM, BRST, MUS, BRN, SKIN, VAS, GI, HRT, PANC, PLCNT, OVRY, CRVX, LIV, BONE	ESDR,LNG,SKIN,SKIN,CRVX		SF1	LAMC1	intronic
1	183094858	0.81	0.95	rs10458355	C	T	0.58	0.57	FAT, SKIN, HRT, LNG	ESC, ESDR, LNG, IPSC, FAT, STRM, BRST, MUS, BRN, SKIN, VAS, GI, PANC, PLCNT, HRT, OVRY, CRVX, LIV, BONE			FAC1,Foxo,Foxp1,HDAC2,Irf,Nanog,Pax-4,Pou5f1,RREB-1,S	LAMC1	intronic

													ox,Zfp105,p300		
1	183096048	0.82	0.96	rs4491025	C	T	0.58	0.57	FAT, HRT	ESC, ESDR, LNG, IPSC, FAT, STRM, BRST, MUS, BRN, SKIN, GI, ADRL, HRT, KID, PANC, OVRY, LIV, BONE	LNG			LAMC1	intronic
1	183096271	0.81	0.95	rs7525917	A	G	0.58	0.57	FAT, STRM, HRT	ESC, ESDR, LNG, IPSC, FAT, STRM, BRST, MUS, BRN, SKIN, GI, ADRL, HRT, KID, PANC, OVRY, LIV, BONE			GATA,Otx2	LAMC1	intronic
1	183096769	0.82	0.96	rs6424884	C	T	0.58	0.57	FAT, STRM, SKIN, GI, BRN, HRT, LNG, MUS	ESC, ESDR, LNG, IPSC, FAT, STRM, BRST, MUS, BRN, SKIN, VAS, GI, ADRL, HRT, KID, PANC, PLCNT, OVRY, LIV, BONE	SKIN,SKIN,LNG,MUS, MUS		HNF1,Mrg1::Hoxa9, Pbx3,RXR::LXR	LAMC1	intronic
1	183097119	0.82	0.95	rs6424885	T	G	0.58	0.57	ESC, IPSC, FAT, STRM, BRN, SKIN, GI, HRT, LNG, MUS	ESC, ESDR, LNG, IPSC, FAT, STRM, BRST, MUS, BRN, SKIN, VAS, GI, ADRL, HRT, KID, PANC, PLCNT, THYM, OVRY, CRVX, LIV, BONE	ESDR,LNG,SKIN,SKIN,SKIN,ADRL,HRT,GI,KID,LNG,MUS,MUS,GI,THYM,OVRY,PANC,MUS,MUS,MUS,SKIN,LNG		Ascl2,CDP,LBP-1,Myf,Tgif1	LAMC1	intronic
1	183099370	0.82	0.96	rs10752895	C	G	0.58	0.57	FAT, GI, LNG	ESC, ESDR, LNG, IPSC, FAT, STRM, BRST, MUS, BRN, SKIN, VAS, GI, HRT, KID, PLCNT, THYM, OVRY, PANC, LIV, BONE			Osr	LAMC1	intronic
1	183099381	0.82	0.96	rs10752896	A	C	0.58	0.57	FAT, GI, LNG	ESC, ESDR, LNG, IPSC, FAT, STRM, BRST, MUS, BRN, SKIN, VAS, GI, HRT, KID, PLCNT, THYM, OVRY, PANC, LIV, BONE			LF-A1,Mtf1,Osr,SRF	LAMC1	intronic
1	183103012	0.82	0.96	rs10911242	C	T	0.58	0.57	FAT, STRM, SKIN, GI	ESC, ESDR, LNG, IPSC, FAT, STRM, BRST, MUS, SKIN, GI, HRT, KID, PLCNT, BLD, BONE			Foxd3,SIX5	LAMC1	intronic
1	183103455	0.81	0.96	rs2296288	T	C	0.58	0.58	FAT, STRM, SKIN, GI, MUS, SPLN	ESC, ESDR, LNG, IPSC, FAT, STRM, BRST, SKIN, VAS, GI, HRT, KID, MUS, PLCNT, OVRY, BONE	IPSC,KID,LNG,MUS		GR,ZBTB33	LAMC1	synonymous
1	183103773	0.82	0.96	rs10797835	T	C	0.58	0.57	SKIN, GI, MUS	ESDR, ESC, LNG, IPSC, FAT, STRM, BRST, SKIN, VAS, GI, HRT, KID, PLCNT, MUS, BONE	LNG			LAMC1	intronic
1	183104131	0.82	0.96	rs2027075	A	G	0.58	0.57	MUS	ESDR, IPSC, FAT, STRM, SKIN, VAS, GI, HRT, KID, LNG, PLCNT, MUS, BONE			Arid3a,Cart1,Duxl,Evi-1,GATA,Lhx4	LAMC1	intronic
1	183104272	0.82	0.96	rs2027076	C	A	0.58	0.57	MUS	ESDR, IPSC, FAT, STRM, SKIN, VAS, GI, HRT, KID, LNG,			Nanog,PRDM1,STAT	LAMC1	intronic

										PLCNT, MUS, BONE					
1	183104343	0.82	0.95	rs2027078	G	A	0.58	0.57	MUS	ESDR, IPSC, FAT, STRM, MUS, SKIN, VAS, GI, HRT, KID, LNG, PLCNT, BONE			Arid5b	LAMC1	intronic
1	183104948	0.82	0.96	rs10797836	G	C	0.58	0.57	FAT, SKIN, MUS, GI	LNG, FAT, STRM, BRST, BLD, MUS, SKIN, VAS, GI, HRT, KID, OVRY, PLCNT, BRN, BONE				LAMC1	intronic
1	183105280	0.82	0.95	rs2027080	C	T	0.58	0.57	FAT, BLD, SKIN, MUS, GI, BONE	LNG, FAT, STRM, BRST, BLD, MUS, SKIN, VAS, GI, HRT, KID, OVRY, PLCNT, BRN, BONE	ESDR		Arid5b,Smad4	LAMC1	intronic
1	183105432	0.82	0.96	rs5779153	CA	C	0.58	0.57	FAT, BLD, SKIN, LNG, MUS, GI, BONE	LNG, FAT, STRM, BRST, BLD, MUS, SKIN, VAS, GI, HRT, KID, OVRY, PLCNT, BRN, BONE	ESDR,SKIN,SKIN		HMG-IY,Zbtb3	LAMC1	intronic
1	183105447	0.82	0.96	rs3768629	T	C	0.58	0.57	FAT, BLD, SKIN, LNG, MUS, GI, BONE	LNG, FAT, STRM, BRST, BLD, MUS, SKIN, VAS, GI, HRT, KID, OVRY, PLCNT, BRN, BONE	ESDR,LNG,SKIN,SKIN, BRN		HMG-IY,Zbtb3	LAMC1	intronic
1	183105740	0.82	0.96	rs2147585	C	T	0.58	0.57	FAT, SKIN, HRT, LNG, MUS, GI, BONE	LNG, FAT, STRM, BRST, BLD, MUS, SKIN, VAS, GI, HRT, KID, OVRY, PLCNT, BRN, BONE	ESDR,LNG,SKIN,LNG, MUS,MUS,MUS,BRN, LNG			LAMC1	intronic
1	183105829	0.82	0.96	rs2182020	C	T	0.58	0.57	FAT, SKIN, HRT, LNG, MUS, GI, BONE	LNG, FAT, STRM, BRST, BLD, MUS, SKIN, VAS, GI, HRT, KID, OVRY, PLCNT, BRN, BONE	ESDR,LNG		EWSR1-FLI1,Pax-5	LAMC1	intronic
1	183105882	0.82	0.96	rs7414273	T	C	0.58	0.57	FAT, SKIN, HRT, MUS, GI, BONE	LNG, FAT, STRM, BRST, BLD, MUS, SKIN, VAS, GI, HRT, KID, OVRY, PLCNT, BRN, BONE				LAMC1	intronic
1	183105986	0.82	0.96	rs10797837	C	G	0.58	0.57	FAT, SKIN, HRT, MUS, GI, BONE	ESC, LNG, FAT, STRM, BRST, BLD, MUS, SKIN, VAS, GI, HRT, KID, OVRY, PLCNT, BRN, BONE				LAMC1	intronic
1	183106280	0.82	0.96	rs10911243	G	A	0.58	0.57	FAT, SKIN, HRT, MUS, GI, BONE	ESC, LNG, FAT, STRM, BRST, BLD, MUS, SKIN, VAS, GI, HRT, KID, OVRY, PLCNT, BRN, BONE	ESDR,LNG,SKIN,MUS, LNG		Fox,Foxa,Foxj1,Foxl 1,Foxp1,Foxq1,HNF 1,Hoxa10,Hoxd10,Zf p105	LAMC1	intronic
1	183106321	0.82	0.96	rs2333622	T	C	0.58	0.57	FAT, SKIN, HRT, MUS, GI, BONE	ESC, LNG, FAT, STRM, BRST, MUS, SKIN, VAS, GI, HRT, KID, OVRY, PLCNT, BRN, BONE	ESDR,LNG,SKIN,LNG, MUS,MUS,BRN,LNG		Pou3f2,Pou5f1	LAMC1	intronic

1	183106597	0.82	0.96	rs12091137	A	G	0.58	0.57	FAT, SKIN, HRT, MUS, GI, BONE	ESDR, ESC, LNG, FAT, STRM, BRST, MUS, SKIN, VAS, GI, HRT, OVRY, PLCNT, BRN, BONE	ESDR		NF-kappaB	LAMC1	intronic
1	183106643	0.82	0.96	rs12086466	T	C	0.58	0.57	FAT, SKIN, HRT, MUS, GI, BONE	ESDR, ESC, LNG, FAT, STRM, BRST, MUS, SKIN, VAS, GI, HRT, OVRY, PLCNT, BRN, BONE	ESDR		Irf,Maf,Mef2,NRSF,Pou2f2,Smad,YY1	LAMC1	intronic
1	183107152	0.81	0.95	rs723014	A	G	0.58	0.57	FAT, BRN, HRT, MUS, SPLN, BONE	ESC, LNG, FAT, STRM, BRST, MUS, SKIN, VAS, GI, HRT, OVRY, PLCNT, BRN, BONE	ESDR		Evi-1,Osf2,PTF1-beta,Pou2f2	LAMC1	intronic
1	183107858	0.81	0.95	rs10911247	A	G	0.58	0.57	HRT, SPLN	ESC, LNG, IPSC, FAT, STRM, BRST, SKIN, BRN, GI, HRT, MUS, OVRY, BLD			LBP-1,LXR,MAZ,SP1,SP2,SRF,TCF12	LAMC1	intronic
1	183107885	0.81	0.95	rs10797838	A	G	0.58	0.57	HRT	ESC, IPSC, FAT, STRM, BRST, SKIN, BRN, GI, HRT, LNG, MUS, OVRY, BLD				LAMC1	intronic
1	183108160	0.8	0.94	rs199520097	T	TA	0.58	0.57	HRT	ESC, IPSC, FAT, STRM, BRST, BRN, SKIN, GI, HRT, LNG, OVRY, MUS, BLD			PLZF,Rad21	LAMC1	intronic
1	183108161	0.81	0.95	rs3835273	A	AG	0.58	0.57	HRT	ESC, IPSC, FAT, STRM, BRST, BRN, SKIN, GI, HRT, LNG, OVRY, MUS, BLD			PLZF,RXRA,Rad21,Sox	LAMC1	intronic
1	183108480	0.81	0.95	rs3765521	C	T	0.58	0.57	HRT	ESC, ESDR, IPSC, FAT, BRST, BRN, SKIN, GI, HRT, LNG, OVRY, MUS, BLD			Cdx,Foxd1,Foxf1,Foxk1,Foxl1,Foxo,Pou2f2	LAMC1	intronic
1	183108483	0.81	0.95	rs3765522	C	T	0.58	0.57	HRT	ESC, ESDR, IPSC, FAT, BRST, BRN, SKIN, GI, HRT, LNG, OVRY, MUS, BLD			Cdc5,Cdx,Foxj1,Foxk1,Foxo,Nkx2,Pou2f2,Pou3f2	LAMC1	intronic
1	183108662	0.81	0.95	rs10797839	T	C	0.58	0.57	HRT	ESC, ESDR, IPSC, FAT, BRN, SKIN, GI, HRT, LNG, OVRY, MUS, BLD			Sox	LAMC1	intronic
1	183109327	0.81	0.95	rs3768626	A	G	0.58	0.57	ESC, IPSC, HRT	ESC, ESDR, IPSC, FAT, BRN, SKIN, GI, HRT, MUS			Foxj1,Mef2,Sox	LAMC1	intronic
1	183110117	0.81	0.95	rs3768624	C	T	0.58	0.57		ESC, ESDR, IPSC, FAT, MUS, BRN, SKIN, GI, HRT, LNG, OVRY				LAMC1	intronic
1	183110241	0.81	0.95	rs3818417	G	A	0.58	0.57		ESC, ESDR, IPSC, FAT, MUS, BRN, SKIN, GI, HRT, LNG, OVRY				LAMC1	intronic
1	183110277	0.81	0.95	rs2296290	C	T	0.58	0.57		ESC, ESDR, IPSC, FAT, MUS, BRN, SKIN, HRT, LNG, OVRY			Evi-1,Gsc,Hmbox1,	LAMC1	intronic

													Otx2,Pax-4,Pax-5,Pitx3,p300		
1	183110374	0.81	0.95	rs2296291	C	T	0.58	0.57		ESC, ESDR, IPSC, FAT, MUS, BRN, SKIN, HRT, LNG, OVRY			Irf,Mef2	LAMC1	intronic
1	183110718	0.81	0.95	rs6672093	T	C	0.58	0.57	BRN	ESC, ESDR, IPSC, FAT, MUS, BRN, SKIN, HRT, LNG, OVRY	ESDR		PU.1,Pax-4,p300	LAMC1	intronic
1	183111404	0.8	0.94	rs6686682	A	G	0.58	0.57		ESC, ESDR, FAT, BRN, SKIN, MUS			Homez	LAMC1	intronic
1	183111429	0.81	0.95	rs10911248	G	A	0.58	0.58		ESC, ESDR, FAT, BRN, SKIN, MUS			CEBPG,GATA,PLZF	LAMC1	intronic
1	183111555	0.81	0.95	rs10911249	G	A	0.58	0.57		ESDR, FAT, BRN, SKIN, MUS			DMRT1,DMRT2,DMRT3,DMRT4,DMRT5,DMRT7,Pou2f2,Pou3f2,Sox	LAMC1	intronic
1	183111996	0.81	0.95	rs10797840	G	A	0.58	0.57		FAT, SKIN, MUS			CEBPA,CEBPD,Zbtb3	LAMC1	intronic
1	183112010	0.81	0.95	rs10797841	G	A	0.58	0.57		FAT, SKIN, MUS			EBF	LAMC1	intronic
1	183112106	0.81	0.95	rs10752899	T	G	0.58	0.57		FAT, SKIN, MUS			AP-1	LAMC1	intronic
1	183112732	0.81	0.95	rs10797842	G	A	0.58	0.57	LNG, GI	FAT, BLD, SKIN, VAS, MUS, LNG			Nkx2	LAMC1	intronic
1	183112860	0.81	0.95	rs10797843	A	G	0.58	0.57	LNG, GI	FAT, BLD, SKIN, VAS, MUS, LNG			Foxp3	LAMC1	intronic
1	183112932	0.81	0.95	rs10797844	C	T	0.58	0.57	LNG, GI	FAT, BLD, SKIN, VAS, MUS, LNG			NRSF,PU.1,Pax-6	LAMC1	intronic
1	183113286	0.8	0.94	rs6669199	C	A	0.58	0.57	GI	FAT, SKIN, VAS, MUS, LNG			Maf,Nkx2,Pax-8,Roaz,Zbtb3	LAMC1	intronic
1	183114131	0.81	0.95	rs3768623	G	A	0.58	0.57	ESDR, GI	FAT, MUS, LNG			ATF3,HNF4,Rad21,SREBP	LAMC1	intronic
1	183116561	0.8	0.95	rs6424888	A	G	0.58	0.57		FAT, SKIN, LNG			Hbp1,Ik-2,Obox6,Pou1f1	LAMC1	intronic
1	183116620	0.8	0.95	rs20563	A	G	0.58	0.57		FAT, SKIN, LNG			AP-1,Arid5a,Foxa,Irf	LAMC1	missense
1	183117103	0.8	0.95	rs10752900	G	A	0.58	0.57	SPLN	FAT, SKIN			Bbx,GATA,Hbp1	LAMC1	intronic
1	183117622	0.8	0.95	rs2296292	A	C	0.58	0.57	LNG, SPLN	FAT, SKIN, GI			GR	LAMC1	synonymous

1	183118350	0.8	0.95	rs10797846	A	T	0.58	0.57		FAT			DMRT2,DMRT3,Fox,Foxa,Foxk1,Hmbox1,Sox,TCF12,Zfp187	LAMC1	intronic
1	183118878	0.8	0.94	rs1547712	T	A	0.58	0.57					DMRT7,Foxo,Pou6f1,RXRA	LAMC1	intronic
1	183119492	0.8	0.95	rs10911254	A	G	0.58	0.57					AIRE,EWSR1-FLI1,HDAC2,NF-AT1	LAMC1	intronic
1	183119733	0.8	0.94	rs10911255	G	C	0.57	0.57					Foxp1,Foxp3,HDAC2,Smad,p300	LAMC1	intronic
1	201818812	0.83	0.94	rs2678210	T	C	0.11	0.31		FAT, STRM, MUS, SKIN, HRT, BRN			Ets,FEV,NF-AT,RBP-Jkappa	NAV1	intronic
1	201828407	0.93	0.97	rs2644109	C	T	0.11	0.32	ESC, ESDR, IPSC, FAT, STRM, BRST, BLD, MUS, BRN, SKIN, GI, ADRL, HRT, LNG, PLCNT, CRVX, BONE	ESC, ESDR, LNG, IPSC, FAT, STRM, BRST, BLD, MUS, BRN, SKIN, VAS, LIV, GI, ADRL, HRT, PLCNT, THYM, OVRY, PANC, SPLN, CRVX, BONE	LNG,BLD,SKIN,SKIN,HRT,MUS		Pax-5	IPO9-AS1	
1	201831383	0.96	0.98	rs2678204	T	G	0.11	0.32	ESC, ESDR, IPSC, FAT, STRM, BRST, BLD, MUS, BRN, SKIN, VAS, LIV, GI, ADRL, HRT, LNG, THYM, CRVX, BONE	ESC, ESDR, IPSC, FAT, STRM, BRST, BLD, MUS, BRN, SKIN, VAS, LIV, GI, ADRL, HRT, PANC, LNG, PLCNT, THYM, OVRY, SPLN, CRVX, BONE			Osf2	IPO9	intronic
1	201831740	0.95	0.98	rs2820295	G	A	0.11	0.32	ESC, ESDR, IPSC, FAT, BRST, BLD, STRM, BRN, SKIN, GI, HRT, LNG, THYM, MUS, CRVX, LIV, BONE	ESC, ESDR, IPSC, FAT, STRM, BRST, BLD, MUS, BRN, SKIN, VAS, LIV, GI, ADRL, HRT, PANC, LNG, THYM, OVRY, SPLN, CRVX, BONE			BCL,Ets,Gfi1,Gfi1b	IPO9	intronic
1	201831883	0.96	0.98	rs2494114	C	G	0.11	0.32	ESC, ESDR, IPSC, BRST, BLD, STRM, BRN, SKIN, GI, HRT, LNG, CRVX	ESC, ESDR, IPSC, FAT, STRM, BRST, BLD, MUS, BRN, SKIN, VAS, LIV, GI, HRT, PANC, THYM, OVRY, SPLN, LNG, CRVX			FAC1,RREB-1,Sox,Zfp105	IPO9	intronic

1	201832924	0.95	0.98	rs2644111	G	A	0.11	0.32	IPSC, BLD, BRN, SKIN, GI, CRVX	ESC, ESDR, LNG, IPSC, FAT, STRM, BRST, BLD, BRN, SKIN, VAS, LIV, GI, ADRL, HRT, PANC, THYM, MUS, CRVX			BATF,GR,PU.1,T3R	IPO9	intronic
1	201836978	0.95	0.98	rs2644112	T	C	0.11	0.32	BLD	BLD, BRN, SKIN, LNG, MUS, HRT, CRVX, VAS			HNF4,Irx,Pou2f2	IPO9	intronic
1	201838648	0.96	0.99	rs2644119	C	T	0.12	0.32		FAT, STRM, BLD, SKIN, LIV, LNG, CRVX, VAS, BONE	LNG	CEBPB	Zic	IPO9	intronic
1	201839341	0.96	0.99	rs2459761	C	G	0.11	0.32	ESDR, GI	BLD, SKIN, OVRY, MUS, LNG, VAS	BLD		LUN-1	IPO9	intronic
1	201840790	0.97	0.99	rs903678	G	A	0.11	0.32	BLD	ESC, ESDR, IPSC, BLD, BRN, SKIN, HRT, MUS, GI, LNG, LIV			Dobox4	IPO9	intronic
1	201841279	0.96	0.99	rs2644122	A	G	0.11	0.32	BLD	ESC, ESDR, IPSC, BLD, BRN, SKIN, LIV, HRT, MUS, GI, LNG			AFP1,Arid3a,Bbx,Nkx2	IPO9	intronic
1	201842633	0.96	0.99	rs2820316	C	T	0.11	0.32	ESDR	ESC, ESDR, IPSC, FAT, STRM, BRST, BLD, MUS, BRN, SKIN, VAS, LIV, GI, HRT, PANC, LNG, OVRY, SPLN, BONE	ESDR,LIV	CEBPB,FOXA1,FOXA2,HNF4G,P300,SP1	CEBPB,TATA	IPO9	intronic
1	201846726	0.96	0.99	rs3951628	A	G	0.12	0.32		ESC, ESDR, LNG, STRM, BRST, BLD, MUS, BRN, SKIN, VAS, LIV, PANC, HRT, CRVX, BONE			HP1-site-factor,Sox	IPO9	intronic
1	201847511	0.91	0.97	rs146207613	ATCTTTT	A	0.11	0.31	BRN	ESC, ESDR, STRM, BRST, BLD, MUS, BRN, SKIN, VAS, LIV, HRT, PANC, LNG, CRVX, BONE			Foxp3,Homez	IPO9	intronic
1	201849501	0.96	0.99	rs2644107	T	C	0.11	0.32	FAT, STRM, BRST, MUS, SKIN, VAS, BRN, BONE	ESC, ESDR, FAT, STRM, BRST, MUS, BRN, SKIN, VAS, LIV, GI, HRT, PANC, LNG, CRVX, BONE	BRST,SKIN,VAS		Evi-1,GATA	IPO9	intronic
1	201852315	0.96	0.99	rs1400875	T	C	0.11	0.32		ESDR, STRM, SKIN, BRN, HRT, LNG, VAS	ESDR		AP-1	IPO9	intronic
1	201856148	0.89	0.98	rs58473985	TC	T	0.11	0.34				STAT3	NF-AT1	IPO9	intronic
1	201856669	0.97	0.99	rs145657297	TTTG	T	0.12	0.32					Foxp1,RREB-1,SIX5	IPO9	intronic
1	201857212	0.96	0.99	rs2172935	C	T	0.11	0.32		CRVX				IPO9	intronic
1	201857354	0.96	0.99	rs2132363	C	T	0.11	0.32		CRVX				IPO9	intronic
1	201861119	0.98	0.99	rs2254614	T	C	0.11	0.32		SKIN, BRN				IPO9	intronic
1	201863736	0.98	0.99	rs2494115	G	C	0.11	0.32		LNG			DMRT4,p300	IPO9	intronic

1	201865816	0.94	0.98	rs2820309	A	G	0.11	0.33		ESDR, BLD, MUS			NRSF,PU.1	IPO9	intronic
1	201868979	0.98	0.99	rs2820310	G	A	0.11	0.32	ESDR, SKIN	SKIN				IPO9	intronic
1	201872348	0.97	0.99	rs2820311	A	G	0.12	0.32		SKIN			CTCF,Myf,RAR	IPO9	intronic
1	201876447	0.99	1	rs8024	C	A	0.11	0.32	MUS	ESDR, FAT, BRST, SKIN, BRN, GI, ADRL, HRT, LNG	SKIN,CRVX	CTCF,SMC3,RAD21		IPO9	3'-UTR
1	201880929	0.99	1	rs1517810	T	C	0.11	0.32		ESC, ESDR, IPSC, FAT, BLD, SKIN, VAS, LIV, BRN, GI, MUS, PLCNT, HRT, CRVX				IPO9	3'-UTR
1	201882364	0.88	-0.96	rs2644134	A	G	0.89	0.69	BLD, SKIN, MUS	ESC, ESDR, LNG, IPSC, FAT, STRM, BRST, BLD, MUS, SKIN, VAS, BRN, GI, ADRL, HRT, PLCNT, CRVX			BCL,ERalpha-a,Ets,Irf,SP1,ZBRK1	IPO9	3'-UTR
		0.94	-0.99	rs141129128	AC	A	0.88	0.69	BLD, SKIN, MUS	ESC, ESDR, LNG, IPSC, FAT, STRM, BRST, BLD, MUS, SKIN, VAS, BRN, GI, ADRL, HRT, PLCNT, CRVX, BONE			FAC1,Foxa,Foxd3,Foxj1,Foxk1,Foxo,Foxp1,HMG-IY,Nanog,Sox,Zfp105,p300	IPO9	3'-UTR
1	201887128	0.86	-0.98	rs2644135	C	G	0.87	0.65	IPSC, BLD, STRM, BRN, SKIN, GI, LNG, MUS, SPLN, BONE	ESDR, ESC, FAT, STRM, BRST, BLD, BRN, SKIN, VAS, GI, ADRL, HRT, MUS, LNG, OVRY, PANC, PLCNT, CRVX, LIV, BONE	BLD		Nanog,RREB-1	1.6kb 5' of SHISA4	
1	201891498	0.99	-1	rs2250377	A	G	0.89	0.68	ESDR, STRM, BRST, BRN, SKIN, ADRL, MUS, LNG, PLCNT, GI, SPLN, BONE	ESDR, FAT, ESC, STRM, BRST, BLD, BRN, SKIN, GI, MUS, PLCNT, HRT, LNG, PANC, SPLN			ATF4,CTCF,Rad21	SHISA4	missense
1	201895702	0.99	-1	rs2644114	A	G	0.89	0.68	ESC, IPSC, BLD, STRM, SKIN, BRN, GI, KID, SPLN, CRVX	ESC, ESDR, LNG, IPSC, FAT, STRM, BRST, BLD, BRN, SKIN, GI, MUS, PLCNT, THYM, OVRY, HRT, SPLN, CRVX	ESDR,ESC,OVRY	GATA2	CCNT2,Evi-1,GATA,HDAC2,HMGN3,TAL1	RP11-307B6.3	
1	201896635	0.99	-1	rs8028	A	G	0.89	0.68	ESDR, ESC, IPSC, STRM, BLD, BRN, SKIN, GI, HRT, MUS, SPLN, CRVX, LNG	ESC, ESDR, LNG, IPSC, FAT, STRM, BRST, BLD, MUS, BRN, SKIN, VAS, GI, HRT, THYM, OVRY, PLCNT, SPLN, CRVX, BONE			Hic1,Pbx3,Pou2f2,Zbtb3	RP11-307B6.3	3'-UTR
1	201900129	0.99	1	rs2820312	G	A	0.11	0.32	SKIN, BRN, GI, SPLN	ESDR, LNG, FAT, STRM, BRST, BRN, SKIN, VAS, GI, ADRL, MUS, OVRY, HRT, SPLN, CRVX, BLD		PU1	TAL1	LMOD1	missense

1	201901093	1	1	rs2820313	A	G	0.11	0.32	SKIN, GI	ESDR, FAT, STRM, BRST, BRN, SKIN, VAS, GI, ADRL, MUS, OVRY, HRT, BLD, BONE			BDP1	LMOD1	intronic
1	201901964	0.8	0.94	rs72310529	TTA	T	0.11	0.34	STRM, SKIN, GI	ESC, LNG, FAT, STRM, BRST, SKIN, BRN, GI, MUS, OVRY, HRT, BONE			CDP,Evi-1,Mef2,Pax-4,Sox	LMOD1	intronic
1	201903081	0.87	0.93	rs2820314	A	C	0.11	0.32	ESC, FAT, STRM, SKIN, GI, MUS, BONE	ESC, ESDR, LNG, IPSC, FAT, STRM, BRST, MUS, BRN, SKIN, VAS, GI, ADRL, PLCNT, HRT, OVRY, PANC, SPLN, BONE	SKIN,SKIN,MUS		EWSR1-FLI1,HDAC2,Irf,STAT	LMOD1	intronic
1	201903136	0.83	0.94	rs2820315	C	T	0.11	0.30	ESC, FAT, STRM, SKIN, GI, MUS, BONE	ESC, ESDR, LNG, IPSC, FAT, STRM, BRST, MUS, BRN, SKIN, VAS, GI, ADRL, PLCNT, HRT, OVRY, PANC, SPLN, BONE	ESDR,ESDR,ESDR,SKIN,OVRY,MUS,BRN			LMOD1	intronic
1	201909409	0.86	0.94	rs2820317	G	C	0.11	0.33	SKIN, GI	ESDR, LNG, BRST, SKIN, VAS, BRN, GI, MUS, PLCNT, OVRY, HRT			Cdx,DMRT5,EWSR1-FLI1,Foxp1,HDAC2,Irf,Nanog,STAT,Sox,TATA,p300	LMOD1	intronic
1	201909492	0.86	0.94	rs2820318	T	C	0.11	0.33	SKIN, GI	ESDR, LNG, BRST, SKIN, VAS, BRN, GI, MUS, PLCNT, OVRY, HRT			E2F,PLAG1,ZNF263,Zfx	LMOD1	intronic
1	201909522	0.85	0.94	rs2820319	G	A	0.11	0.33	SKIN, GI	ESDR, LNG, BRST, SKIN, VAS, BRN, GI, MUS, PLCNT, OVRY, HRT			E2A,TBX5,ZEB1	LMOD1	intronic
1	201911168	0.85	0.94	rs2820321	G	C	0.11	0.33	SKIN, GI	IPSC, FAT, ESC, BRST, BRN, SKIN, VAS, GI, PLCNT, OVRY, MUS, HRT			EBF,LF-A1,NRSF,PU.1,Sin3Ak-20	LMOD1	intronic
1	201911172	0.86	0.94	rs2820322	T	C	0.11	0.33	SKIN, GI	IPSC, FAT, ESC, BRST, BRN, SKIN, VAS, GI, PLCNT, OVRY, MUS, HRT			BCL,GR,NRSF,Sin3Ak-20,p300	LMOD1	intronic
1	201912156	0.85	0.94	rs2644121	A	G	0.11	0.33	SKIN, GI, MUS, SPLN	ESC, ESDR, LNG, IPSC, FAT, BRST, BRN, SKIN, VAS, GI, PLCNT, OVRY, MUS, HRT	ESDR		BDP1,Egr-1,NF-kappaB,Pou2f2,p300	LMOD1	intronic
1	201912959	0.86	0.94	rs2819346	A	C	0.11	0.33	FAT, SKIN, GI, MUS	ESC, ESDR, LNG, IPSC, FAT, BRST, BRN, SKIN, VAS, GI, MUS, PLCNT, OVRY, HRT, SPLN, BONE	ESDR,ESDR,LNG,SKIN,KID,PLCNT,OVRY			LMOD1	intronic
1	201914032	0.85	0.94	rs2820323	G	A	0.11	0.33	FAT, SKIN, GI, MUS, SPLN	ESC, ESDR, LNG, IPSC, FAT, BRST, SKIN, VAS, GI, KID, MUS, PLCNT, HRT, OVRY, SPLN, BONE			Irf,SRF	LMOD1	intronic

1	201915160	0.85	0.94	rs2819347	C	G	0.11	0.33	IPSC, FAT, SKIN, GI, MUS, CRVX, BRN, BONE	ESC, ESDR, LNG, FAT, STRM, BRST, MUS, SKIN, VAS, GI, ADRL, KID, PLCNT, HRT, OVRY, SPLN, CRVX, BLD, BRN, BONE	BRN		MZF1::1-4,PU.1	LMOD1	intronic
1	201915824	0.86	0.94	rs2819348	T	C	0.12	0.33	ESDR, IPSC, FAT, SKIN, GI, MUS, CRVX, BRN, BONE	ESDR, LNG, IPSC, FAT, STRM, BRST, MUS, SKIN, VAS, GI, ADRL, KID, PLCNT, HRT, OVRY, SPLN, CRVX, BLD, BRN, BONE	ADRL		Ets,Spz1,TATA,TCF12,ZNF263,Znf143	LMOD1	intronic
1	201915898	0.85	0.94	rs2819349	C	T	0.12	0.33	ESDR, IPSC, FAT, SKIN, GI, MUS, CRVX, BRN, BONE	ESDR, LNG, IPSC, FAT, STRM, BRST, MUS, SKIN, VAS, GI, ADRL, KID, PLCNT, HRT, OVRY, SPLN, CRVX, BLD, BRN, BONE	ADRL		DEC,Hand1	LMOD1	intronic
1	201916029	0.85	0.94	rs2819351	C	T	0.12	0.33	ESDR, IPSC, FAT, SKIN, GI, MUS, CRVX, BRN, BONE	ESDR, LNG, IPSC, FAT, STRM, BRST, MUS, SKIN, VAS, GI, ADRL, KID, PLCNT, HRT, OVRY, SPLN, CRVX, BLD, BRN, BONE	ADRL		Pax-6,TFII-I	LMOD1	intronic
1	201917641	0.8	0.91	rs34091558	TA	T	0.12	0.32	FAT, STRM, MUS, SKIN, GI, LNG, CRVX, BRN, BONE	ESC, ESDR, LNG, IPSC, FAT, STRM, BRST, MUS, BRN, SKIN, VAS, GI, ADRL, KID, PLCNT, HRT, OVRY, SPLN, CRVX, LIV, BLD, BONE	ESDR,LNG,BRST,SKIN,SKIN,OVRY,PANC,MUS,CRVX,MUS,MUS,VAS,BLD,BRN,SKIN,LNG	CFOS,CJUN,GATA2,AP2ALPHA,CEBPB,ELK4,JUND,P300,STAT3	DMRT2,Foxf2,Foxo,GATA,GR	LMOD1	intronic
12	57094031	0.81	0.99	rs324017	A	C	0.50	0.72	ESC, ESDR, LNG, IPSC, FAT, STRM, BRST, BLD, MUS, BRN, SKIN, GI, KID, THYM, PLCNT, HRT, SPLN, CRVX, BONE	ESC, ESDR, LNG, IPSC, FAT, STRM, BRST, BLD, MUS, BRN, SKIN, LIV, GI, ADRL, HRT, KID, PLCNT, THYM, PANC, SPLN, CRVX, VAS, BONE	ESC,ESDR,ESDR,ESDR,ESDR,ESC,LNG,IPSC,IPSC,BRST,BLD,BLD,BLD,BLD,BLD,BLD,BLD,SKIN,SKIN,SKIN,SKIN,ADRL,BRN,BRN,HRT,KID,LNG,MUS,MUS,GI,THYM,GI,OVRY,PANC,MUS,BLD,LIV,BRST,	POL2,EGR1,MEF2A,PBX3,POL24H8,NFKB,TAF1,ELF1	AP-2,BCL,CTCF,Egr-1,Ets,HEY1,PU.1,Sin3Ak-20,Znf143,p300	NAB2	intronic

											MUS,MUS,BLD,BLD,BRN,SKIN,SKIN,LNG				
12	57096317	1	1	rs324015	T	C	0.51	0.76	BRST, BLD, SKIN, GI, PLCNT	ESC, ESDR, LNG, IPSC, FAT, STRM, BRST, BLD, MUS, BRN, SKIN, LIV, GI, ADRL, PLCNT, THYM, HRT, SPLN, CRVX, BONE	LNG,IPSC,BRST,SKIN,SKIN,SKIN,SKIN,GI,KID,LNG,PLCNT,GI,THYM,GI,GI,BLD,CRVX,BRST,MUS,BLD,BRN,SKIN,SKIN	POL2,POL24H8,STAT3	Klf7,RREB-1,SREBP,p53	STAT6	3'-UTR
17	83093262	1	1	rs7502442	G	T	0.41	0.72	ESDR, FAT, SKIN, GI, PLCNT, SPLN, CRVX	ESC, ESDR, IPSC, FAT, STRM, BLD, MUS, SKIN, BRN, GI, HRT, LNG, PLCNT, OVRY, PANC, SPLN, CRVX	BLD		AP-1,ERalpha-a,SMC3	METRNL	intronic
17	83095585	0.86	0.98	rs35204860	T	C	0.40	0.70	ESDR, SKIN, GI, CRVX	ESC, FAT, BLD, SKIN, GI, PLCNT, LNG, PANC, HRT, MUS, BONE	PLCNT		AP-2,ERalpha-a,Ets,HNF4,RAR,RXRA,p300	465bp 3' of METRNL	
		0.84	0.98	rs35256515	C	CG	0.40	0.69	ESDR, SKIN, GI	ESC, FAT, BLD, SKIN, GI, PLCNT, LNG, PANC, HRT, MUS, BONE	PLCNT		AP-2,ELF1,Ets,RXRA,p300	488bp 3' of METRNL	
17	83099877	0.82	0.93	rs34400960	C	G	0.41	0.71	ESC, ESDR, LNG, IPSC, FAT, STRM, BRST, BLD, MUS, BRN, SKIN, VAS, GI, HRT, OVRY, PLCNT, CRVX, LIV, BONE	ESC, ESDR, LNG, IPSC, FAT, STRM, BRST, BLD, MUS, BRN, SKIN, VAS, LIV, GI, ADRL, HRT, PLCNT, THYM, OVRY, PANC, SPLN, CRVX, BONE	BLD,BLD,BLD,BLD,HRT,KID,LNG,MUS,PLCNT,GI,OVRY,PANC,MUS,BLD		AP-2,BCL,ELF1,EWSR1-FLI1,Egr-1,Rad21,SMC3,SP1,Sp4	3.6kb 5' of AC144831.1	
15	71899358	0.8	0.92	rs67715200	TTTTAA	T	0.37	0.20	BRN	BRN			Arid3a,Eomes,HNF1,Nkx6-1,Pax-5,Pax-6,Pou2f2,Pou3f1	MYO9A	
15	71914730	0.83	0.93	rs4776581	G	A	0.37	0.20					Barhl1,Gbx2,Hoxa9,Hoxc9,Hoxd8,Isx,Lh	MYO9A	intronic

												x4,Msx2,Nobox,SP1,TATA		
15	71915178	0.8	0.92	rs202059518	TA	T	0.37	0.20					GATA,Hoxa5,Irf,Isl2,Nanog,Pou2f2,Pou6f1,STAT,TCF4,Zfp410	MYO9A	intronic
15	71915179	0.8	0.92	rs4776582	A	T	0.37	0.20					Isl2,Pou2f2,RORalpha1	MYO9A	intronic
15	71915463	0.83	0.93	rs4777471	A	G	0.37	0.21		ESC, PANC				MYO9A	intronic
15	71920312	0.83	0.93	rs12911154	G	A	0.37	0.20		BRST			Mef2	MYO9A	intronic
15	71922724	0.83	0.93	rs12906711	A	G	0.37	0.20					Arid3a,Cart1,Esx1,Hoxa10,Hoxa3,Hoxa5,Hoxa9,Hoxb13,Hoxd10,Lhx4,Mef2,Mrg1::Hoxa9,Nkx6-1,Pax7,Pou2f2,Pou3f2,Pou6f1,Prrx2,Sox	MYO9A	intronic
15	71925865	0.8	0.93	rs4777475	C	T	0.37	0.20	ESC				Sox	MYO9A	intronic
15	71926253	0.82	0.93	rs7170754	G	A	0.37	0.20	BLD, LNG	PANC			E2F,EBF,Egr-1,Nrf1,PPAR,SETDB1	MYO9A	intronic
15	71926258	0.82	0.93	rs7170461	C	A	0.37	0.20	BLD, LNG	PANC			E2F,Egr-1,Nrf1,SETDB1	MYO9A	intronic
15	71929657	0.82	0.93	rs12917428	T	C	0.37	0.20	BRST, LIV, GI, HRT, LNG, OVRY				DMRT2,GR,Nanog,Pou3f2	MYO9A	intronic
15	71935496	0.83	0.93	rs2306490	A	G	0.37	0.20					Foxj1,HMG-IY,PLZF,SIX5,Sox	MYO9A	intronic

15	71937193	0.83	0.93	rs1384007	T	C	0.37	0.20		BRST, STRM				MYO9A	intronic
15	71943734	0.82	0.93	rs62025574	C	T	0.37	0.20	LNG	LNG	KID			MYO9A	intronic
15	71946632	0.82	0.93	rs11853268	T	C	0.37	0.20					Foxa,Foxf1,Foxi1,Foxj1,p300	MYO9A	intronic
15	71947448	0.82	0.93	rs71395051	C	T	0.37	0.20					Evi-1,Foxa,Foxc1,Foxf1,Foxi1,Foxj1,Foxk1,Foxl1,Foxo,Mef2,Nkx2,Nkx3,Pou5f1,TATA,Zfp105	MYO9A	intronic
15	71951325	0.8	0.9	rs2279283	T	C	0.37	0.21	STRM, MUS, BONE	ESC, FAT, STRM, MUS, VAS, PANC, BONE	LNG,MUS,BRN,LNG		Cart1,Evi-1,Pou2f2,Sox	MYO9A	intronic
15	71953106	0.83	0.93	rs11072338	T	C	0.37	0.20		ESC, IPSC, STRM, PANC			Cdx,DMRT2,Foxa,Foxf1,SIX5	MYO9A	intronic
15	71954849	0.83	0.93	rs11072339	C	T	0.37	0.20		MUS, SKIN			CEBPA	MYO9A	intronic
15	71958400	0.83	0.93	rs1074330	C	T	0.37	0.20	PLCNT	LNG, GI, BONE			Foxo,Hoxa10,Hoxd10,Irf	MYO9A	intronic
15	71960157	0.83	0.93	rs12916951	C	T	0.37	0.20	PLCNT	GI	PLCNT		Pax-4,TATA	MYO9A	intronic
15	71960655	0.83	0.93	rs12902006	C	T	0.37	0.20	PLCNT	VAS, GI, PLCNT			Dobox4,SIX5,TAL1	MYO9A	intronic
15	71964724	0.83	0.93	rs34137768	T	G	0.37	0.21		PLCNT			BDP1,ERalpha-a,Rad21,VDR,ZBRK1,Zbtb3	MYO9A	intronic
15	71973153	0.83	0.93	rs11852789	G	C	0.37	0.20	PLCNT	ESDR, SKIN, KID, LNG, PLCNT	PLCNT		SP1	RP11-390D11.1	intronic
15	71974874	0.83	0.93	rs12595228	T	C	0.37	0.20	PLCNT	ESDR, ESC, BRST, SKIN, GI, KID, LNG, PLCNT	ESDR,PLCNT			RP11-390D11.1	intronic
15	71976645	0.83	0.93	rs10518979	A	G	0.37	0.20	PLCNT	ESDR, BRST, KID, PLCNT			HNF1,Nanog	RP11-390	intronic

														D11.1	
15	71977271	0.83	0.93	rs4777477	G	A	0.37	0.20	PLCNT	ESDR, BRST, PLCNT			ERalpha-a,Nrf-2,TCF11::MafG	RP11-390D11.1	intronic
15	71978894	0.83	0.93	rs16956444	C	T	0.37	0.20			SKIN		Foxp1,GATA,p300	RP11-390D11.1	intronic
15	71986744	0.83	0.93	rs4777478	T	C	0.37	0.20	BLD	GI			Barhl1,Foxa,Foxi1,Foxj2,Foxl1,Mef2,PLZF,Pou5f1,SRF	RP11-390D11.1	intronic
15	71991751	0.83	0.93	rs16956452	T	C	0.37	0.20					HNF1,Irf	RP11-390D11.1	intronic
15	71992306	0.83	0.93	rs12592364	C	A	0.37	0.20					AFP1	RP11-390D11.1	intronic
15	71999774	0.83	0.93	rs2306488	A	G	0.37	0.20	ESC	PANC	BLD		Rhox11	RP11-390D11.1	intronic
15	72000861	0.83	0.93	rs2035379	C	T	0.37	0.20					Fox,Osf2	RP11-390D11.1	intronic
15	72002057	0.83	0.93	rs12595464	C	T	0.37	0.20	ESDR		GI		HNF4	RP11-390D11.1	intronic
15	72002416	0.83	0.93	rs8025504	C	T	0.37	0.20	ESDR				TCF12,ZEB1	RP11-390D11.1	intronic
15	72005799	0.83	0.93	rs12916694	C	T	0.37	0.20		LNG, GI, KID, PANC			GR,Mef2,Zbtb12	RP11-390D11.1	intronic
15	72006126	0.83	0.93	rs35115747	G	A	0.37	0.20		ESDR, LNG, GI, KID, PANC				RP11-390D11.1	intronic
15	72007140	0.83	0.93	rs12903484	G	A	0.37	0.20		GI, KID, PANC			Pou1f1,Pou2f2,Pou5f1,XBP-1	RP11-390D11.1	intronic

15	72010326	0.83	0.93	rs2200056	T	C	0.37	0.20		ESDR, ESC, GI, KID, LNG			DMRT7,Mef2,Pax-4,Pou2f2,TATA	RP11-390D11.1	intronic
15	72010666	0.83	0.93	rs12593134	G	C	0.37	0.20		ESDR, ESC, GI, LNG	LNG,GI		GR,HDAC2,HNF1,Nanog,Sox	RP11-390D11.1	intronic
15	72023460	0.83	0.93	rs4776584	A	G	0.36	0.20	ESDR				CAC-binding-protein,NF-E2,Pou2f2,Rad21,SP1	RP11-390D11.1	intronic
15	72031450	0.83	0.94	rs8032038	C	T	0.37	0.20		SKIN, GI			TEF-1,Zbtb3	RP11-390D11.1	intronic
15	72035368	0.84	0.93	rs35770840	CG	C	0.37	0.21	STRM, SKIN, GI	LNG, FAT, STRM, BRST, MUS, SKIN, VAS, BRN, GI, OVRY, HRT			CEBPD,Foxd3,Foxo,GR,Zfp105	RP11-390D11.1	intronic
15	72036656	0.85	0.94	rs4777480	A	C	0.37	0.20	ESDR, STRM, GI, HRT, LNG	LNG, FAT, STRM, BRST, BLD, SKIN, VAS, BRN, GI, OVRY, MUS, HRT, SPLN, BONE	MUS		BCL,GATA,HDAC2,HMGN3,Hand1,TAL1	RP11-390D11.1	intronic
15	72041938	0.85	0.94	rs12914070	T	C	0.37	0.20		IPSC, STRM, BRST, BLD, SKIN, VAS, BRN, HRT, GI, MUS			BCL,DMRT1,DMRT2,DMRT7,Irf,Pax-5,Pou5f1,RXRA,STAT	MYO9A	intronic
15	72055713	0.86	0.95	rs8028532	G	A	0.36	0.20		ESC, IPSC, BLD, PLCNT			CIZ,Evi-1,Foxa,Foxp1,Maf,Pou2f2	MYO9A	intronic
15	72062257	0.86	0.95	rs4445848	C	T	0.37	0.20	GI	ESC, ESDR, LNG, IPSC, FAT, STRM, BRST, BLD, SKIN, BRN, GI, HRT, OVRY, MUS, THYM, VAS, BONE	ESDR,LNG		DMRT5,Smad3	MYO9A	intronic
15	72063057	0.86	0.95	rs4131630	C	T	0.37	0.20	FAT, GI	ESDR, LNG, FAT, STRM, BRST, MUS, OVRY, HRT, BONE			Hbp1,RFX5	MYO9A	intronic
15	72065440	0.83	0.93	rs12908300	G	A	0.34	0.20		STRM, BRN			Ets,HNF4,Irf,RXRA,Rad21,SMC3,SP1,TATA	MYO9A	intronic
15	72069865	0.85	0.94	rs12901580	C	A	0.36	0.20		ESC, IPSC, BLD, BRN, SKIN, VAS, GI, LNG			Pitx2,SETDB1	MYO9A	intronic
15	72081299	0.86	0.95	rs12439900	A	G	0.37	0.20		FAT, BRST, BLD, SKIN, OVRY, MUS	MUS		Pou2f2	MYO9A	intronic

15	72084202	0.86	0.95	rs4777484	T	C	0.37	0.20	MUS	MUS, GI			Lhx3,Obox3,RORalpha1	MYO9A	intronic
15	72084937	0.86	0.95	rs7183749	G	A	0.37	0.20	MUS	MUS, GI		SETDB1	AP-1,AP-2,BAF155,BATF,BCL,Bach1,Brachyury,E2A,GATA,GR,HMGN3,Irf,KAP1,Mef2,Myc,Myf,PRDM1,RXRA,SIX5,STAT,TBX5,TCF4,p300	MYO9A	intronic
15	72086619	0.86	0.95	rs35167572	A	G	0.37	0.20	SKIN, GI	ESDR, FAT, BRST, STRM, SKIN, BRN, GI, HRT, LNG, MUS, OVRY, PANC, SPLN	HRT,GI,GI		Gm397,Mtf1,TCF12	MYO9A	intronic
15	72092142	0.86	0.95	rs7172309	C	T	0.37	0.20		LNG, GI, HRT, OVRY, MUS, BLD	BLD	GATA1		MYO9A	intronic
15	72100174	0.88	0.96	rs35186252	G	A	0.36	0.20		ESDR			GCNF,RXRA,p300	MYO9A	intronic
15	72103532	0.88	0.97	rs76734127	G	T	0.35	0.20	ESDR, ESC, BRN, FAT, LNG, MUS	ESDR, LNG, IPSC, FAT, STRM, BRST, BLD, MUS, BRN, SKIN, GI, PANC, HRT, OVRY, SPLN, BONE	BLD,BLD,BLD,BLD		NRSF,TATA	MYO9A	intronic
15	72107324	0.85	0.95	rs12593908	A	C	0.37	0.20		ESC, ESDR, LNG, IPSC, FAT, STRM, BRST, BLD, MUS, SKIN, BRN, GI, HRT, PANC, OVRY, CRVX, BONE	LNG,IPSC,MUS,CRVX,BRN		CDP	MYO9A	intronic
15	72107528	0.88	0.97	rs12050895	G	A	0.36	0.20		ESC, ESDR, LNG, IPSC, FAT, BRST, BLD, STRM, SKIN, BRN, GI, HRT, PANC, OVRY, MUS, CRVX, BONE			EBF,Pax-5	MYO9A	intronic
15	72107545	0.88	0.96	rs35673773	C	A	0.36	0.20		ESC, ESDR, LNG, IPSC, FAT, BRST, BLD, STRM, SKIN, BRN, GI, HRT, PANC, OVRY, CRVX, BONE			AIRE,AP-1,CCNT2,HNF4,Klf4,Klf7,MAZR,MZF1::1-4,Pou2f2,SP1,UF1H3BETA,Zfp281	MYO9A	intronic
15	72108093	0.87	0.97	rs11072346	G	A	0.37	0.20		ESC, LNG, IPSC, FAT, BRST, BLD, STRM, SKIN, BRN, GI, HRT, PANC, CRVX			Foxp1,Hoxd10,Irf,Pou2f2,Pou6f1	MYO9A	intronic

15	72109897	0.87	0.97	rs35741721	CA	C	0.37	0.20		ESDR, FAT, BLD, MUS, SKIN, LNG, GI, HRT			Cdx,Dbx1,Evi-1,Foxa,Foxd3,Foxf1,Foxi1,Foxj1,Foxj2,Foxk1,Foxl1,Foxo,Foxp1,GATA,HDAC2,HMG-IY,Ncx,Pou3f2,Pou3f4,TATA,Zfp105	MYO9A	intronic
15	72112232	0.81	0.92	rs34189331	A	T	0.39	0.22	FAT, GI, LNG, MUS, BLD	FAT, STRM, BRST, BLD, MUS, SKIN, BRN, GI, HRT, KID, LNG, PLCNT, BONE	LNG,MUS,MUS,MUS		Cdx,Dbx1,Evi-1,FAC1,Foxa,Foxd3,Foxf1,Foxj1,Foxk1,Foxl1,Foxo,Foxp1,HDAC2,Mef2,Ncx,Pax-4,Pou3f2,Pou3f4,TATA,Zfp105	MYO9A	intronic
15	72115728	0.89	0.97	rs12901886	G	A	0.37	0.20	ESC, ESDR, IPSC, FAT, STRM, BRST, BLD, MUS, BRN, SKIN, VAS, LIV, GI, KID, PANC, LNG, OVRY, PLCNT, HRT, THYM, CRVX, BONE	ESC, ESDR, IPSC, FAT, STRM, BRST, BLD, MUS, BRN, SKIN, LIV, GI, ADRL, HRT, KID, PANC, LNG, PLCNT, CRVX, VAS, BONE			CDP,Foxp1	SENP8	intronic
15	72125883	0.92	0.99	rs12591426	G	C	0.37	0.20					AP-2rep,HNF4,SREBP,VDR,Zbtb3	SENP8	intronic
15	72126774	0.92	0.99	rs12592044	A	G	0.37	0.20	GI	KID				SENP8	intronic
15	72129262	0.96	1	rs4238449	G	A	0.37	0.21		KID, GI, HRT			AP-1,Pax-5,ZBTB33	SENP8	intronic
15	72129348	0.95	0.98	rs4411469	A	G	0.37	0.21		GI, HRT			Pou6f1	SENP8	intronic
15	72132597	0.92	1	rs199577141	GTTA	G	0.34	0.20		BLD, GI, LNG			AP-1,GATA,Zfp740	SENP8	intronic

15	72132602	0.93	1	rs60305971	TATC	T	0.36	0.20		BLD, GI, LNG			RXRA,Zfp740	SENP8	intronic
15	72137648	0.8	-1	rs2034879	G	A	0.42	0.75		BRN, HRT, KID, LNG, GI			ELF1,EWSR1-FLI1,Elf3,Ets,Pax-4,ZNF263	SENP8	intronic
15	72138093	1	1	rs4777489	A	G	0.37	0.21		BRN, HRT, KID, LNG, GI			Hoxb6,Nanog,Nrf-2,Nrf1,Pou1f1,Pou2f2,Pou3f3,Pou5f1,TATA	SENP8	intronic
15	72138162	0.93	1	rs4776586	C	A	0.37	0.20		BRN, HRT, KID, LNG, GI			Hoxa9,Hoxd10,Pou2f2	SENP8	intronic
15	72140841	0.93	1	rs55854204	C	T	0.37	0.20	ESDR, HRT, BLD	ESDR, ESC, LNG, BLD, STRM, BRN, HRT, GI, KID, PANC, MUS			Foxf1,Foxi1,Foxl1,Mef2,TATA	SENP8	3'-UTR
15	72145231	0.96	1	rs12908882	G	A	0.37	0.21		BRN, HRT, GI			Brachyury,Eomes,GZF1,Hsf,YY1	RP11-2I17.4	
15	72155985	0.88	0.97	rs35112434	G	T	0.37	0.20	ESC, ESDR, LNG, IPSC, FAT, STRM, BRST, BLD, MUS, BRN, SKIN, VAS, LIV, GI, ADRL, HRT, KID, PANC, PLCNT, THYM, OVRY, SPLN, CRVX, BONE	ESC, ESDR, LNG, IPSC, FAT, STRM, BRST, BLD, MUS, BRN, SKIN, VAS, LIV, GI, ADRL, HRT, KID, PANC, PLCNT, THYM, SPLN, CRVX, BONE	ESC,ESDR,ESC,LNG,IPSC,IPSC,BRST,BLD,BLD,BLD,BLD,BLD,SKIN,SKIN,SKIN,SKIN,ADRL,HRT,GI,GI,KID,LNG,MUS,MUS,PLCNT,GI,THYM,GI,OVRY,PANC,MUS,GI,LNG,BLD,BRST,MUS,BLD,BRN,SKIN,LNG	ELF1,CTCF,ZNF263	EWSR1-FLI1	525bp 5' of RP11-2I17.4	
15	72167630	0.92	0.98	rs35713471	C	A	0.36	0.21	ESDR, LNG, PLCNT, GI, SPLN	ESC, ESDR, IPSC, BRST, BLD, BRN, SKIN, GI, ADRL, HRT, LNG, MUS, PLCNT, SPLN, LIV	PLCNT		CTCF,ELF1,Pbx3	GRAMD2	intronic

15	72170009	0.92	0.98	rs34487006	G	T	0.36	0.21	BRST, GI, LNG, PLCNT, SPLN, LIV	ESC, ESDR, IPSC, BRST, BLD, STRM, BRN, SKIN, GI, LNG, MUS, PLCNT, THYM, PANC, HRT, LIV	PLCNT		Ets,RBP-Jkappa	GRAMD2	intronic
15	72170490	0.83	-0.91	rs7182776	T	C	0.63	0.79	ESC, BRST, GI, PLCNT, SPLN, LIV	ESDR, ESC, IPSC, BRST, BLD, STRM, SKIN, BRN, GI, MUS, PLCNT, THYM, LNG, PANC, HRT, LIV	ESDR,BRST,GI,GI,PLCNT,GI,GI,GI,BRST	ELF1	Ehf	GRAMD2	intronic
15	72170984	0.88	0.97	rs16953769	G	A	0.37	0.20	ESC, BRST, PLCNT, GI, SPLN	ESDR, IPSC, BRST, STRM, SKIN, BRN, GI, PLCNT, LNG, PANC	PLCNT		AP-1	GRAMD2	intronic
15	72174458	0.87	0.97	rs3743224	C	T	0.37	0.20	IPSC, STRM, SKIN	ESDR, IPSC, BRST, STRM, SKIN, BRN, GI, PLCNT, THYM, LNG	PLCNT		GR,STAT	GRAMD2	intronic
15	72187817	0.89	0.95	rs12902661	T	G	0.37	0.21		BRST, SKIN, GI	BRST,SKIN			GRAMD2	intronic
15	72200011	0.85	0.96	rs8192431	G	A	0.37	0.20	ESDR, SKIN, BRN, GI, LNG, MUS, SPLN, CRVX	ESDR, LNG, FAT, STRM, BRST, BLD, MUS, BRN, SKIN, LIV, GI, ADRL, HRT, PANC, PLCNT, THYM, SPLN, CRVX, BONE	ESC,ESDR,ESC,BRST,BLD,BLD,BLD,BLD,BLD,SKIN,SKIN,SKIN,SKIN,ADRL,BRN,HRT,GI,GI,KID,LNG,MUS,PLCNT,GI,THYM,MUS,GI,LNG,BLD,CRVX,LIV,BRST,VAS,BLD,BLD,SKIN	POL2,CTCF,ELF1,SMC3,NFKB,JUND,MAX,HNF4A,HNF4G,GATA3,ZNF263	ERalpha-a,TR4	PKM	intronic
15	72203670	0.89	0.96	rs2607090	C	A	0.37	0.21	ESDR, FAT, BRN, SKIN, GI, MUS, SPLN, CRVX, BONE	ESDR, LNG, FAT, STRM, BRST, BLD, MUS, BRN, SKIN, GI, HRT, KID, PANC, THYM, SPLN, CRVX, LIV, VAS, BONE	ESDR,ESDR,LNG,BRST,SKIN,SKIN,SKIN,SKIN,BRN,BRN,HRT,GI,KID,LNG,MUS,MUS,GI,THYM,GI,OVRY,PANC,MUS,GI,CRVX,BRST,MUS,MUS,VAS,BRN,SKIN,SKIN,LNG	MAX,POL2,P300,RAD21,USF1,YY1		PKM	intronic
15	72204421	0.88	0.95	rs2856929	T	C	0.37	0.21	ESDR, FAT, BRN, SKIN, MUS, GI, SPLN, CRVX, BONE	ESDR, LNG, FAT, STRM, BRST, BLD, MUS, BRN, SKIN, GI, HRT, PANC, THYM, SPLN, CRVX, VAS, BONE	BRST,SKIN,SKIN		Pou2f2,p300	PKM	intronic
15	72208445	0.89	0.96	rs3803471	A	G	0.37	0.21	ESC, ESDR, SKIN, BRN, GI,	ESDR, FAT, BRST, BLD, STRM, BRN, SKIN, GI, HRT, MUS,			FAC1,Foxd3,Foxk1,F	PKM	intronic

									SPLN, CRVX	LNG, CRVX			oxo,Foxp1,GR,HDAC2,Irf,Nanog,Pax-4,REB-1,Sox,Zfp105,p300		
15	72211718	0.82	-0.93	rs8023703	G	A	0.63	0.78	SPLN, CRVX	ESDR, BRST, SKIN, HRT, GI, PANC, MUS, LNG, CRVX			AP-1	PKM	intronic
15	72220807	0.85	0.96	rs8192386	G	T	0.37	0.20	ESDR, STRM, BLD, SKIN, BRN, HRT, GI, MUS, LNG, CRVX	ESC, ESDR, LNG, IPSC, FAT, STRM, BRST, BLD, MUS, BRN, SKIN, GI, HRT, KID, PANC, PLCNT, SPLN, CRVX, LIV, VAS, BONE	ESDR,ESC,IPSC,BLD,SKIN,HRT,BLD,SKIN,LNG	POL24H8,POL2	Ik-3,STAT	PKM	intronic
15	72221978	0.88	0.96	rs1037680	T	C	0.37	0.21	ESDR, FAT, STRM, BRST, BLD, SKIN, BRN, GI, MUS, LNG, CRVX	ESC, ESDR, LNG, IPSC, FAT, STRM, BRST, BLD, MUS, BRN, SKIN, GI, ADRL, HRT, PANC, PLCNT, SPLN, CRVX, LIV, VAS, BONE	BLD,HRT,SKIN			PKM	intronic
15	72223934	0.84	0.96	rs200644761	AT	A	0.37	0.20	ESDR, BLD, STRM, MUS, SKIN, BRN, GI, HRT, LNG, CRVX, BRST, BONE	ESC, ESDR, LNG, IPSC, FAT, STRM, BRST, BLD, MUS, BRN, SKIN, VAS, GI, HRT, PANC, PLCNT, THYM, CRVX, LIV, BONE			Foxd3,Foxp1,Gfi1,Irf,STAT	PKM	intronic
15	72225332	0.84	0.96	rs35494718	TA	T	0.37	0.20	ESDR, BLD, STRM, SKIN, HRT, LNG, CRVX, BRN	ESC, ESDR, LNG, FAT, STRM, BRST, BLD, MUS, BRN, SKIN, GI, HRT, PANC, THYM, PLCNT, CRVX, LIV, VAS, BONE			Evi-1,Fox,Foxa,Foxd3,Foxf1,Foxi1,Foxj1,Foxl1,Foxo,Foxp1,HDAC2,Irf,TATA,Zfp105,p300	PKM	intronic
15	72227487	0.88	0.96	rs8192364	G	A	0.37	0.21	ESC, ESDR, LNG, IPSC, FAT, STRM, BRST, BLD, MUS, BRN, SKIN, GI, HRT, KID, PANC, THYM, PLCNT, CRVX, LIV, VAS, BONE	ESC, ESDR, LNG, IPSC, FAT, STRM, BRST, BLD, MUS, BRN, SKIN, GI, ADRL, HRT, KID, PANC, PLCNT, THYM, SPLN, CRVX, LIV, VAS, BONE	ESDR,LNG,BRST,BLD,BLD,BLD,SKIN,SKIN,SKIN,ADRL,GI,GI,PLCNT,GI,GI,OVRY,MUS,GI,LNG,BLD,CRVX,BRST,MUS,MUS,VAS,BLD,BLD,BRN,SKIN,SKIN,L	POL2,POL24H8,TAF1,GATA2,JUND,FOSL1,STAT3,P300	Gfi1,Pitx3	PKM	intronic

											NG				
15	72233391	0.88	0.95	rs77685254	T	G	0.38	0.21	ESDR, BLD, SKIN, THYM, GI, SPLN, CRVX	ESDR, IPSC, FAT, BLD, SKIN, VAS, BRN, GI, ADRL, MUS, THYM, LNG, PLCNT, HRT, SPLN, CRVX, LIV			ERalpha-a,SETDB1	1.6kb 5' of PKM	
15	72247153	0.85	0.96	rs35206011	T	C	0.37	0.20		ESDR			Smad	PARP6	intronic
15	72259902	0.84	0.95	rs16956634	G	C	0.37	0.20		ESDR			AFP1,Egr-1,HNF1	PARP6	intronic
15	72260151	0.84	0.95	rs11858015	G	A	0.37	0.20		ESDR				PARP6	intronic
		0.83	0.94	rs66520586	A	G	0.37	0.20	ESDR	ESDR			Pax-4,TFII-I	PARP6	intronic
15	72264405	0.82	0.94	rs12591313	T	C	0.37	0.20		ESDR, BRST, BLD, SKIN				PARP6	intronic
15	72264506	0.82	0.94	rs12591338	T	A	0.37	0.20		ESDR, BRST, BLD, SKIN			ERalpha-a,EWSR1-FLI1,GR,Pax-6,TATA,TFII-I	PARP6	intronic
15	72274919	0.82	0.94	rs72733369	G	A	0.38	0.20	BLD, SKIN, GI	BRST, BLD, SKIN, BRN, GI, MUS, THYM			GCNF,Smad	CELF6	
17	7835897	0.92	0.96	rs74692204	C	T	0.37	0.07	ESC, ESDR, LNG, IPSC, FAT, STRM, BRST, BLD, MUS, BRN, SKIN, VAS, LIV, GI, ADRL, HRT, KID, PANC, PLCNT, THYM, OVRY, SPLN, CRVX, BONE	ESDR, ESC, LNG, IPSC, FAT, STRM, BRST, BLD, MUS, BRN, SKIN, LIV, GI, ADRL, PLCNT, THYM, PANC, CRVX, VAS, BONE	ESC,ESDR,ESDR,ESDR,ESDR,ESC,LNG,IPSC,IPSC,BRST,BLD,BLD,BLD,BLD,BLD,BLD,SKIN,SKIN,SKIN,SKIN,ADRL,BRN,BRN,HRT,GI,KID,LNG,MUS,MUS,PLCNT,GI,THYM,GI,OVRY,PANC,MUS,GI,LNG,BLD,CRVX,LIV,BRST,MUS,MUS,VAS,BLD,BLD,BRN,SKIN,SKIN,LNG	EBF1,NRF1,POL2,ZEB1,TAF1,HEY1		2.2kb 3' of DNAH2	
17	7840475	0.92	0.96	rs55977787	C	T	0.37	0.07	ESC, ESDR, IPSC, FAT, STRM,	ESC, ESDR, LNG, IPSC, FAT, STRM, BRST, BLD, MUS, BRN,	BLD,SKIN			KDM6B	intronic

									BRST, BLD, MUS, BRN, SKIN, GI, HRT, KID, LNG, THYM, PLCNT, SPLN, CRVX	SKIN, LIV, GI, ADRL, HRT, KID, PANC, PLCNT, THYM, SPLN, CRVX, BONE					
17	7846303	0.92	0.96	rs80152199	A	G	0.37	0.07	ESDR, FAT, ESC, BRST, BLD, STRM, SKIN, BRN, GI, THYM, PLCNT, MUS, HRT, SPLN	ESDR, LNG, IPSC, FAT, BRST, BLD, BRN, SKIN, LIV, GI, MUS, THYM, HRT, PANC, PLCNT, SPLN, BONE	BLD,BLD,PLCNT,MUS		Ets,MZF1::1-4,PU.1,SP1,STAT,UF1H3BETA	KDM6B	intronic
17	7848070	0.92	0.96	rs3744247	C	A	0.37	0.07	ESC, BRST, BLD, SKIN, SPLN	IPSC, ESC, BRST, BLD, SKIN, GI, BRN, MUS, LNG, PANC, HRT	BLD,BLD		EBF,Evi-1	KDM6B	synonymous
17	7848433	0.92	0.96	rs3744248	C	T	0.37	0.07	ESC, BRST, BLD, SKIN, SPLN	IPSC, BRST, BLD, SKIN, GI, PANC	BLD,BLD,BLD,MUS		BRCA1,FAC1	KDM6B	synonymous
17	7850977	0.92	0.96	rs146364592	TCCTCTG	T	0.36	0.07	ESDR, ESC, IPSC, BRST, BLD, SKIN, FAT, BRN, GI, PANC, PLCNT, MUS, THYM, SPLN, BONE	ESC, ESDR, IPSC, BRST, BLD, BRN, SKIN, FAT, GI, MUS, PLCNT, THYM, HRT, LNG, PANC, SPLN	ESC,BLD,BLD,BLD,ADRL,OVRY,MUS		BCL,BDP1,ERalpha-a,PU.1,STAT,TR4,UF1H3BETA,p300	KDM6B	intronic
17	7857665	0.88	0.96	rs3744251	G	A	0.37	0.07	ESC, ESDR, LNG, IPSC, FAT, STRM, BRST, BLD, MUS, BRN, SKIN, VAS, LIV, GI, ADRL, HRT, KID, PANC, PLCNT, THYM, OVRY, SPLN, CRVX, BONE	ESDR, IPSC, STRM, BLD, SKIN, FAT, LIV, BRN, GI, HRT, LNG, MUS, PLCNT, THYM, PANC, CRVX, BRST, BONE	ESC,ESDR,ESDR,ESDR,ESDR,ESC,LNG,IPSC,IPSC,BRST,BLD,BLD,BLD,BLD,BLD,BLD,BLD,BLD,SKIN,SKIN,SKIN,SKIN,ADRL,BRN,BRN,HRT,GI,GI,KID,LNG,MUS,MUS,PLCNT,GI,THYM,GI,OVRY,PANC,MUS,GI,LNG,BLD,CRVX,LIV,BRST,MUS,MUS,VAS,B	BRCA1,CHD2,PAX5C20,POL24H8,RFX5,SIN3AK20,TBP,ZBTB33,POL2,ELK4,MXI1,TAF1,GRP20,GATA1	Ets,Hand1,p300	LSMD1	5'-UTR

											LD,BLD,BRN,SKIN,SKIN,LNG				
17	7868144	1	1	rs76730553	C	T	0.37	0.07	BLD, SKIN, GI	BRST, BLD, SKIN, GI, LNG, THYM, PANC, PLCNT, SPLN, LIV	IPSC,BRST,BLD,BLD,BLD,SKIN,GI,BRST,BLD,SKIN		EBF	LSMD1	
17	7870729	1	1	rs55680968	A	G	0.37	0.07	BLD, GI	BLD, GI, PLCNT	BLD		Fox,Foxj1,Foxk1,Foxp1,HMG-IY,Pax-4	LSMD1	intronic
17	7873667	1	1	rs55722807	CA	C	0.37	0.07	SKIN, GI, BLD	ESC, ESDR, BLD, LNG, THYM, PLCNT			ATF3,BDP1,E2F,ERalpha-a,LUN-1,PU.1,Rad21,SP1	LSMD1	
17	7873896	1	1	rs11078711	G	T	0.37	0.07	SKIN, GI, BLD	ESC, ESDR, BLD, LNG, THYM, PLCNT				LSMD1	
17	7875207	1	1	rs79951937	A	T	0.37	0.07	BLD	ESDR, BLD			AIRE,FAC1,Foxp1,Irf,Nanog,Pax-4,RREB-1,Sox,p300	LSMD1	
17	7877702	0.98	1	rs76833657	G	A	0.34	0.06		BLD, PANC			DMRT5,Zfp105	LSMD1	
17	7878722	1	1	rs59328845	G	A	0.37	0.07		BLD, BRN, PANC, THYM			BAF155,Ets,Hoxa13,Hoxb13,Hoxd10,Myc,SIX5,Znf143	LSMD1	
17	7880977	1	1	rs17807204	A	G	0.37	0.07	ESDR, BLD, SKIN, BRN, GI, KID, LNG, SPLN, CRVX, MUS	ESDR, ESC, FAT, BRST, BLD, STRM, MUS, BRN, SKIN, GI, ADRL, HRT, KID, PANC, LNG, PLCNT, THYM, SPLN, CRVX, LIV, VAS, BONE	BRN,BRN,BLD		Pbx3	LSMD1	
17	7882272	1	1	rs78209469	C	T	0.37	0.07	ESDR, LNG, FAT, STRM, BRST, BLD, MUS, SKIN, BRN, GI, KID, PANC, THYM, SPLN, CRVX	ESC, ESDR, LNG, FAT, STRM, BRST, BLD, MUS, BRN, SKIN, GI, ADRL, HRT, KID, PANC, PLCNT, THYM, SPLN, CRVX, LIV, VAS, BONE	IPSC,SKIN,BRN,BRN,MUS,THYM,BLD,BRN	POL2,ZNF263	RXRA,Zbtb3	LSMD1	
17	7882587	1	1	rs74252295	A	G	0.37	0.07	ESDR, LNG, FAT, STRM,	ESC, ESDR, LNG, FAT, STRM, BRST, BLD, MUS, BRN, SKIN,	LNG,BLD,BRN,MUS,G		EWSR1-FLI1,Evi-1,R	LSMD1	

									BRST, BLD, MUS, SKIN, BRN, GI, KID, PANC, THYM, SPLN, CRVX	GI, ADRL, HRT, KID, PANC, PLCNT, THYM, SPLN, CRVX, LIV, VAS, BONE	I		ad21		
17	7886730	0.92	0.98	rs55752997	C	A	0.27	0.07	ESC, ESDR, LNG, IPSC, FAT, STRM, BRST, BLD, MUS, BRN, SKIN, VAS, LIV, GI, ADRL, HRT, KID, PANC, PLCNT, THYM, OVRY, SPLN, CRVX, BONE	ESC, ESDR, LNG, IPSC, FAT, STRM, BRST, BLD, MUS, BRN, SKIN, LIV, GI, ADRL, HRT, KID, PLCNT, THYM, PANC, SPLN, CRVX, VAS, BONE	ESC,ESDR,ESDR,ESDR,ESC,IPSC,IPSC,BLD,BLD,BLD,BLD,BLD,BLD,SKIN,SKIN,SKIN,SKIN,ADRL,BRN,BRN,HRT,GI,GI,KID,LNG,MUS,MUS,PLCNT,GI,THYM,GI,OVRY,GI,LNG,BLD,CRVX,LIV,MUS,VAS,BLD,BLD,SKIN,SKIN,LNG	POL2,CTCF	HDAC2,Irf,NRSF,PU.1,Pax-5	CHD3	intronic
5	142109462	0.93	0.97	rs6580223	G	T	0.65	0.61	ESC, ESDR, LNG, IPSC, FAT, STRM, BRST, BLD, MUS, BRN, SKIN, VAS, LIV, GI, ADRL, HRT, KID, PANC, PLCNT, THYM, OVRY, SPLN, CRVX, BONE	ESDR, ESC, IPSC, STRM, BLD, BRN, SKIN, FAT, LIV, GI, PLCNT, THYM, HRT, MUS, BONE	ESDR,ESDR,LNG,BRST,BLD,BLD,BLD,BLD,BLD,SKIN,ADRL,BRN,BRN,HRT,GI,KID,LNG,MUS,PLCNT,GI,THYM,MUS,GI,CRVX,VAS,BLD,SKIN	GATA2,CEBPB,GATA1	Myf	NDFIP1	intronic
5	142109741	0.93	0.97	rs6580224	A	G	0.65	0.61	ESC, ESDR, LNG, IPSC, FAT, STRM, BRST, BLD, MUS, BRN, SKIN, VAS, LIV, GI, ADRL, HRT, KID, PANC, PLCNT, THYM, OVRY, SPLN,	ESC, ESDR, STRM, BRST, BLD, MUS, BRN, SKIN, FAT, LIV, GI, ADRL, KID, LNG, PLCNT, THYM, HRT, CRVX, VAS, BONE	ESDR,ESDR,BLD,BRN,BRN,HRT,LNG,MUS,PLCNT,GI,CRVX,MUS,VAS,BLD	POL2	AIRE,AP-1,BATF,Duxl,Evi-1,Ik-2,Mef2	NDFIP1	intronic

									CRVX, BONE					
5	142109816	0.92	0.97	rs6580225	G	C	0.65	0.61	ESC, ESDR, LNG, IPSC, FAT, STRM, BRST, BLD, MUS, BRN, SKIN, VAS, LIV, GI, ADRL, HRT, KID, PANC, PLCNT, THYM, OVRY, SPLN, CRVX, BONE	ESC, ESDR, IPSC, FAT, STRM, BRST, BLD, MUS, BRN, SKIN, LIV, GI, ADRL, HRT, KID, LNG, PLCNT, THYM, CRVX, VAS, BONE	BLD,BLD,SKIN,ADRL, BRN,BRN,HRT,LNG,MUS,MUS,GI,THYM,CRVX,VAS,BLD	E2F6	BCL,GZF1,Irf,PRDM1,PU.1,p300	NDFIP1	intronic
5	142110103	0.93	0.97	rs10569192	GCGGC	G	0.65	0.61	ESC, ESDR, LNG, IPSC, FAT, STRM, BRST, BLD, MUS, BRN, SKIN, VAS, LIV, GI, ADRL, HRT, KID, PANC, PLCNT, THYM, OVRY, SPLN, CRVX, BONE	ESC, ESDR, IPSC, FAT, STRM, BRST, BLD, MUS, BRN, SKIN, LIV, GI, ADRL, HRT, KID, PANC, LNG, PLCNT, THYM, CRVX, VAS, BONE	ESDR,SKIN,SKIN,ADRL,BRN,HRT,KID,LNG,GI,MUS,GI,CRVX,LIV,MUS,BLD,SKIN	BATF,IRF4,PAX5C20,PAX5N19,PU1,CEBPB,CMYC,GATA2	BCL,BHLHE40,SRF,Sin3Ak-20,YY1,p300	NDFIP1	intronic
5	142110288	0.93	0.97	rs12653848	C	T	0.65	0.61	ESC, ESDR, LNG, IPSC, FAT, STRM, BRST, BLD, MUS, BRN, SKIN, VAS, LIV, GI, ADRL, HRT, KID, PANC, PLCNT, THYM, OVRY, SPLN, CRVX, BONE	ESC, ESDR, LNG, IPSC, FAT, STRM, BRST, BLD, MUS, BRN, SKIN, LIV, GI, ADRL, HRT, KID, PANC, PLCNT, THYM, CRVX, VAS, BONE	ESDR,BLD,BLD,ADRL,BRN,BRN,HRT,LNG,MUS,GI,THYM,OVRY,PANC,MUS,CRVX,MUS,MUS,BLD,BLD	IRF4,PU1,CTCF,CMYC,GATA2,MAX	Foxd3,HDAC2,Myc,Zbtb12	NDFIP1	intronic
5	142110356	0.93	0.97	rs12653866	C	T	0.65	0.61	ESC, ESDR, LNG, IPSC, FAT, STRM, BRST, BLD, MUS, BRN, SKIN, VAS, LIV, GI, ADRL, HRT, KID, PANC, PLCNT, THYM, OVRY, SPLN, CRVX, BONE	ESC, ESDR, LNG, IPSC, FAT, STRM, BRST, BLD, MUS, BRN, SKIN, LIV, GI, ADRL, HRT, KID, PANC, PLCNT, THYM, CRVX, VAS, BONE	ESDR,BLD,ADRL,BRN,HRT,GI,LNG,MUS,MUS,GI,CRVX,BRST,MUS,MUS,VAS,BLD,BLD	CTCF,CMYC,MAX	Mrg1::Hoxa9,Nkx2,Nkx3,TAL1	NDFIP1	intronic
5	142110526	0.93	0.97	rs12656877	T	C	0.65	0.61	ESC, ESDR, LNG, IPSC, FAT,	ESC, ESDR, LNG, IPSC, FAT, STRM, BRST, BLD, MUS, BRN,	LNG,BLD,BLD,BLD,BR	CTCF	HMG-IY,Pou1f1	NDFIP1	intronic

									STRM, BRST, BLD, MUS, BRN, SKIN, VAS, LIV, GI, ADRL, HRT, KID, PANC, THYM, OVRY, CRVX, BONE	SKIN, LIV, GI, ADRL, HRT, KID, PANC, PLCNT, THYM, CRVX, VAS, BONE	N,BRN,LNG,GI,SKIN				
5	142111022	0.95	0.97	rs4912622	G	A	0.65	0.61	ESC, ESDR, LNG, IPSC, FAT, STRM, BRST, BLD, MUS, BRN, SKIN, VAS, LIV, GI, ADRL, HRT, KID, PANC, THYM, OVRY, CRVX	ESC, ESDR, LNG, IPSC, FAT, STRM, BRST, BLD, MUS, BRN, SKIN, LIV, GI, ADRL, HRT, KID, PANC, PLCNT, THYM, SPLN, CRVX, VAS, BONE	HRT		Mef2,SRF	NDFIP1	intronic
5	142112420	0.86	0.97	rs7700687	C	T	0.64	0.59	ESC, ESDR, BLD, FAT, LIV, BRN, GI, THYM, CRVX	ESC, LNG, FAT, BLD, SKIN, VAS, LIV, BRN, GI, HRT, PANC, MUS, SPLN	BLD		LUN-1,STAT	NDFIP1	intronic
5	142112854	0.88	0.95	rs7705042	C	A	0.65	0.60	ESC, ESDR, BLD, FAT, BRN, LNG, GI, THYM, CRVX	ESC, LNG, FAT, BRST, BLD, SKIN, VAS, LIV, BRN, GI, HRT, PANC, MUS, SPLN			Irf,Pou1f1,Pou2f2,Pou3f3,p300	NDFIP1	intronic
5	142114049	0.96	0.98	rs4912804	T	C	0.66	0.61	ESDR, IPSC, FAT, BLD, BRN, GI, THYM	ESC, IPSC, FAT, BRST, BLD, STRM, MUS, SKIN, VAS, LIV, BRN, GI, HRT, PANC, LNG, CRVX			BCL,GATA,Irf,PU.1,Pax-5,RXRA,STAT,TATA,p300	NDFIP1	intronic
5	142114120	0.96	0.98	rs4912805	A	G	0.66	0.61	ESDR, IPSC, FAT, BLD, BRN, GI, THYM	ESC, IPSC, FAT, STRM, BRST, BLD, MUS, SKIN, VAS, LIV, BRN, GI, HRT, PANC, LNG, CRVX			Bcl6b,NF-kappaB,STAT,Sp100	NDFIP1	intronic
5	142115154	0.96	0.98	rs13184323	T	G	0.66	0.61	FAT, BLD, BRN	ESC, LNG, FAT, STRM, BRST, BLD, MUS, SKIN, VAS, LIV, BRN, GI, HRT, PANC, OVRY, THYM, SPLN, CRVX, BONE			DMRT1,HDAC2	NDFIP1	intronic
5	142115369	0.83	0.98	rs10068717	C	T	0.61	0.58	FAT, BLD, BRN	ESC, LNG, FAT, STRM, BRST, BLD, MUS, SKIN, VAS, LIV, BRN, GI, HRT, PANC, OVRY, THYM, SPLN, CRVX, BONE			BCL,ERalpha-a,Irf,SP1,ZBRK1	NDFIP1	intronic
5	142115574	0.96	0.98	rs13188700	T	C	0.65	0.61	FAT, BLD, BRN	ESC, LNG, FAT, STRM, BRST, BLD, MUS, SKIN, VAS, LIV, BRN, GI, HRT, PANC, OVRY, THYM, SPLN, CRVX, BONE			CHD2,E2F,Egr-1,Ets,Mtf1,NRSF,Nrf1,SRF,Sin3Ak-20,YY1,Zfp161,Zfx	NDFIP1	intronic

5	142116150	0.96	0.98	rs10875596	T	C	0.65	0.61	FAT, BLD, MUS	ESC, LNG, FAT, STRM, BRST, BLD, MUS, BRN, SKIN, VAS, LIV, GI, HRT, PANC, OVRY, THYM, SPLN, CRVX, BONE	LNG,BRST,BLD,SKIN,SKIN,MUS,CRVX,BRST,MUS,MUS,BLD,BRN,SKIN,SKIN,LNG	STAT3,ZNF263	GATA,Irf	NDFIP1	intronic
5	142116525	0.89	0.98	rs12515668	G	T	0.64	0.59	FAT, BLD	ESDR, LNG, FAT, ESC, STRM, BRST, BLD, MUS, BRN, SKIN, VAS, LIV, GI, HRT, PANC, OVRY, THYM, SPLN, CRVX, BONE			AP-2,CTCF,E2F,Egr-1,GCM,SMC3,TCF12,p53	NDFIP1	intronic
5	142116899	0.96	0.98	rs7712237	T	C	0.65	0.61	FAT, BLD	ESDR, FAT, ESC, STRM, BRST, BLD, MUS, BRN, SKIN, VAS, LIV, GI, HRT, PANC, LNG, THYM, OVRY, SPLN, CRVX, BONE			Mef2	NDFIP1	intronic
5	142118085	0.96	0.98	rs9324866	A	G	0.65	0.61	FAT, BLD, GI, LNG, OVRY	FAT, ESC, BRST, BLD, STRM, MUS, BRN, SKIN, VAS, LIV, GI, ADRL, HRT, PANC, LNG, THYM, OVRY, SPLN, BONE	LNG			NDFIP1	intronic
5	142119067	0.96	0.98	rs1036209	T	A	0.65	0.61	BLD, LNG, OVRY, GI	LNG, FAT, STRM, BRST, BLD, MUS, BRN, SKIN, VAS, LIV, GI, ADRL, HRT, PANC, THYM, OVRY, SPLN, BONE	BLD		Fox,HNF1	NDFIP1	intronic
5	142119476	0.96	0.98	rs1036207	A	G	0.65	0.61	BLD, LNG	LNG, FAT, STRM, BRST, BLD, MUS, BRN, SKIN, LIV, GI, ADRL, HRT, PANC, THYM, OVRY, VAS, BONE	BLD		Cdx2	NDFIP1	intronic
5	142120871	0.96	0.98	rs11749731	A	C	0.66	0.61	BLD	ESDR, LNG, FAT, STRM, BRST, BLD, MUS, BRN, SKIN, VAS, LIV, GI, ADRL, HRT, PANC, THYM, OVRY, SPLN	BLD		Egr-1,Ets,GATA,GR,Maf,Nanog,Pax-5,STAT,Sox,TATA,TCF4,ZBTB33	NDFIP1	intronic
5	142121139	0.96	0.98	rs11750521	T	C	0.66	0.61	BLD	FAT, STRM, BRST, BLD, MUS, SKIN, LIV, BRN, GI, ADRL, HRT, PANC, LNG, THYM, SPLN, VAS			Barhl1,Barx1,Barx2,En-1,Esx1,Gbx1,Gbx2,Hmx,Hoxa5,Hoxb3,Isl2,Isx,Lhx4,Lhx8,Msx-1,Msx2,NF-Y,Nkx1-1,Nkx3,Nobox,Pax7,Phox2a,Prrx1,Pr	NDFIP1	intronic

													rx2		
5	142121943	0.96	0.98	rs1835966	C	T	0.65	0.61	BLD, GI	LNG, FAT, STRM, BRST, BLD, MUS, SKIN, VAS, LIV, BRN, GI, ADRL, HRT, PANC, SPLN			Zbtb3	NDFIP1	intronic
5	142122206	0.96	0.98	rs1835965	G	A	0.65	0.61	BLD, GI	LNG, FAT, BRST, BLD, STRM, MUS, SKIN, VAS, LIV, BRN, GI, HRT, PANC, SPLN	SKIN		Pax-6,ZNF263	NDFIP1	intronic
5	142122470	0.96	0.98	rs10463348	G	T	0.65	0.61	BLD, GI	FAT, BRST, BLD, STRM, LIV, BRN, PANC, MUS, GI, LNG			Cdx,Foxa,SRF	NDFIP1	intronic
5	142123494	0.94	0.98	rs2338822	T	C	0.65	0.61	BLD	BRST, BLD, SKIN, LIV, BRN, GI, HRT, OVRY, PANC, LNG			HDAC2,p300	NDFIP1	intronic
5	142124274	0.96	0.98	rs6877864	A	G	0.65	0.61	BLD, SKIN	FAT, BRST, BLD, SKIN, LIV, BRN, GI, PANC, HRT, OVRY, MUS, SPLN	SKIN	GATA1	BCL,CDP,NRSF,Pax-5,Pou5f1	NDFIP1	intronic
5	142124305	0.96	0.98	rs6882226	T	A	0.65	0.61	BLD, SKIN	FAT, BRST, BLD, SKIN, LIV, BRN, GI, PANC, HRT, OVRY, MUS, SPLN	SKIN	GATA1	AP-2,Ets,Irf,PPAR,STAT,VDR,Znf143	NDFIP1	intronic
5	142125426	0.96	0.98	rs6883827	A	G	0.66	0.61	BLD	BRST, BLD, SKIN, FAT, BRN, GI, PANC, MUS, HRT, SPLN			Evi-1,Rad21	NDFIP1	intronic
5	142125897	0.96	0.98	rs4912623	T	A	0.66	0.61	BLD	BRST, BLD, SKIN, FAT, BRN, GI, PANC, HRT, MUS			Foxk1,Pou2f2,Sox	NDFIP1	intronic
5	142127050	0.96	0.98	rs7737631	T	G	0.67	0.61	BLD	BRST, BLD, SKIN, FAT, VAS, LIV, BRN, GI, PANC, LNG, THYM, HRT, OVRY, MUS				NDFIP1	intronic
5	142127346	0.96	0.98	rs6874308	C	T	0.65	0.61	BLD	IPSC, FAT, BRST, BLD, SKIN, VAS, LIV, BRN, GI, PANC, LNG, THYM, HRT, OVRY, MUS, SPLN	GI		Barhl1,Foxd3,Foxj1,HDAC2,Mef2,Pou1f1	NDFIP1	intronic
5	142128923	0.96	0.98	rs11739961	T	G	0.65	0.61	BLD, SKIN, FAT, MUS	ESC, ESDR, IPSC, FAT, STRM, BRST, BLD, BRN, SKIN, VAS, LIV, GI, ADRL, HRT, PANC, LNG, MUS, THYM, OVRY, CRVX, BONE	BLD,BLD,BLD		Mxi1,RFX5	NDFIP1	intronic
5	142129868	0.96	0.98	rs2338821	A	G	0.66	0.61	FAT, BLD, SKIN, LIV	ESC, ESDR, IPSC, FAT, BRST, BLD, STRM, MUS, BRN, SKIN, VAS, LIV, GI, ADRL, PANC, LNG, HRT, SPLN, CRVX, BONE			YY1	NDFIP1	intronic
5	142129972	0.96	0.98	rs4391200	A	G	0.66	0.61	FAT, BLD, SKIN, LIV	ESC, ESDR, FAT, BRST, BLD, STRM, BRN, SKIN, VAS, LIV, GI, ADRL, PANC, LNG, MUS, HRT, SPLN, CRVX, BONE			Zfp187	NDFIP1	intronic
5	142130032	0.96	0.98	rs10062349	G	A	0.66	0.61	FAT, BLD, SKIN, LIV	ESC, ESDR, FAT, BRST, BLD, STRM, BRN, SKIN, VAS, LIV, GI,			DMRT7,Mef2,Pax-4	NDFIP1	intronic

										ADRL, PANC, LNG, MUS, HRT, SPLN, CRVX, BONE					
5	142130420	0.96	0.98	rs6860138	G	A	0.66	0.61	FAT, BLD, SKIN, LIV	ESDR, IPSC, FAT, BRST, BLD, SKIN, VAS, LIV, BRN, GI, PANC, LNG, HRT, MUS, BONE	BLD		DMRT7,Foxd1,Pou1f1,Prrx2	NDFIP1	intronic
5	142130883	0.96	0.98	rs6580228	C	T	0.66	0.61	FAT, BLD, SKIN, LIV	ESDR, IPSC, FAT, BRST, BLD, SKIN, VAS, LIV, BRN, GI, PANC, OVRY, HRT, BONE			Sox	NDFIP1	intronic
5	142131189	0.96	0.98	rs6580229	A	G	0.66	0.61	BLD, SKIN	ESDR, IPSC, FAT, BRST, BLD, SKIN, VAS, LIV, BRN, GI, PANC, HRT, OVRY			Nr2f2,Pax-2	NDFIP1	intronic
5	142133639	0.96	0.98	rs6863411	A	T	0.66	0.61	BLD, LIV, GI	BLD, SKIN, FAT, LIV, BRN, GI, PANC, HRT, MUS	GI	FOXA1,FOXA2,HNF4A	Crx,Gfi1	NDFIP1	intronic
5	142133831	0.95	0.98	rs4385236	G	A	0.66	0.61	BLD, LIV, GI	BLD, SKIN, FAT, LIV, BRN, GI, PANC, HRT, MUS			RXRA,SRF	NDFIP1	intronic
5	142134126	0.96	0.98	rs2043280	C	G	0.66	0.61	BLD, LIV	BRST, BLD, FAT, LIV, BRN, GI, PANC, OVRY, MUS			Gcm1,Mef2	NDFIP1	intronic
5	142134289	0.96	0.98	rs7709361	T	C	0.66	0.61	BLD	BLD, FAT, LIV, BRN, GI, OVRY, MUS			RXRA	NDFIP1	intronic
5	142134401	0.96	0.98	rs7723666	C	T	0.66	0.61	BLD	BLD, FAT, LIV, BRN, GI, OVRY, MUS			Maf,Pou1f1	NDFIP1	intronic
5	142134462	0.94	0.98	rs7705547	A	G	0.66	0.61	BLD	BLD, FAT, LIV, BRN, GI, OVRY, MUS	BLD		HMG-IY,Homez,PLZF,Pou2f2,Pou5f1,TATA	NDFIP1	intronic
5	142135017	0.87	0.98	rs10041497	T	C	0.66	0.59		BLD, LIV, OVRY, GI			EBF	NDFIP1	intronic
5	142135999	0.96	0.98	rs7733850	C	T	0.65	0.61		BLD, LIV, THYM			Foxp1,GATA,Nanog	NDFIP1	intronic
5	142138100	0.97	0.98	rs3765011	G	C	0.66	0.61		BLD			Fox,Foxa,Foxd3,Foxf1,Foxj1,Foxj2,Foxl1,Foxo,Foxq1,HDAC2,Mef2,Myf,Nanog,Pou2f2,Sox,TAL1,p300	NDFIP1	intronic
5	142139731	0.97	0.98	rs4912807	G	C	0.66	0.61		BLD			Gfi1,HDAC2	NDFIP1	intronic
5	142141894	0.91	0.97	rs7718955	A	G	0.66	0.60		BRN		CTCF	AP-1,AP-2,ATF4,BAF155,BATF,BCL,Bach	NDFIP1	intronic

													1,Egr-1,GATA,GR,HMGN3,Ik-1,Irf,KAP1,Maf,NF-E2,Nrf-2,SETDB1,TCF4,p300		
5	142142648	0.96	0.98	rs12655465	C	T	0.66	0.61		BLD, BRN			NRSF	NDFIP1	intronic
5	142143435	1	1	rs1062158	C	T	0.66	0.61		BLD, BRN			RBP-Jkappa	NDFIP1	intronic
5	142145181	1	1	rs249643	C	G	0.66	0.61		ESC, ESDR, IPSC, FAT, BLD, STRM, SKIN, VAS, BRN, ADRL			COMP1,Ik-1,Ik-2	NDFIP1	intronic
5	142145415	1	1	rs249642	G	C	0.66	0.61		ESC, IPSC, FAT, BLD, STRM, SKIN, VAS, LIV, BRN, ADRL	ESDR,ESC,LNG,IPSC,BLD,BLD,BLD,SKIN,SKIN,SKIN,SKIN,BRN,HRT,GI,LNG,MUS,GI,THYM,MUS,BLD,CRVX,BRST,VAS,BLD,SKIN,SKIN	CTCF,RAD21,SMC3		NDFIP1	intronic
5	142145740	1	1	rs249641	A	G	0.65	0.61	GI	ESC, IPSC, FAT, BLD, STRM, SKIN, LIV, BRN, ADRL	IPSC		LBP-9,ZEB1	NDFIP1	intronic
5	142146492	0.99	1	rs181826	C	A	0.65	0.61		IPSC, BLD, STRM, SKIN, BRN				NDFIP1	intronic
5	142149394	1	1	rs166079	C	T	0.66	0.61		BLD, SKIN, LIV			Foxo,GR,Ik-2,NF-AT,PU.1,Pax-5	NDFIP1	intronic
5	142150592	0.97	0.98	rs413214	G	A	0.66	0.61	SKIN	ESDR, LNG, BLD, SKIN, PANC	GI		SETDB1	NDFIP1	intronic
5	142151131	0.97	0.98	rs249638	G	C	0.66	0.61	SKIN	ESDR, ESC, LNG, IPSC, FAT, STRM, BLD, MUS, SKIN, HRT, PANC, GI, VAS, BONE			Irx,Nkx2,SIX5	NDFIP1	intronic
5	142153497	0.96	0.98	rs449454	A	G	0.66	0.61	ESDR, IPSC	ESC, ESDR, IPSC, BLD, SKIN, LIV, HRT, GI, KID, PANC, LNG, MUS, VAS			Mrg1::Hoxa9,Pou5f1	NDFIP1	3'-UTR
5	142158452	0.96	0.98	rs249681	G	C	0.66	0.61	IPSC	ESC, ESDR, IPSC, FAT, BLD, STRM, MUS, GI, BRN, LNG			Barx1,Dbx1,Nkx6-1,Obox3,PLZF,Sox	4kb 3' of NDFIP1	intronic
5	142159774	0.9	0.98	rs249677	C	A	0.66	0.62		ESC, ESDR, LNG, IPSC, FAT, BLD, STRM, MUS, GI, BRN	BLD		DMRT7,Foxl1,Mef2	5.3kb 3' of	intronic

														NDFIP1	
14	52024937	1	1	rs1497077	T	C	0.80	0.66	ESDR, FAT, STRM, MUS, SKIN, BRN, HRT	ESDR, LNG, FAT, STRM, BRST, MUS, SKIN, BRN, GI, ADRL, HRT, PANC, CRVX, VAS, BONE			Cart1,PLZF,Rad21	NID2	intronic
14	52025036	1	1	rs2516599	T	C	0.81	0.66	ESDR, FAT, STRM, MUS, SKIN, BRN, HRT, VAS	ESDR, LNG, FAT, STRM, BRST, MUS, SKIN, BRN, GI, ADRL, HRT, PANC, CRVX, VAS, BONE				NID2	intronic
17	907319	0.81	0.99	rs2132512	G	A	0.52	0.55	SKIN, GI	ESC, ESDR, LNG, IPSC, BRST, BLD, SKIN, GI, MUS, THYM, HRT, LIV, BRN	LNG		Pax-4	NXN	intronic
17	907409	0.81	0.98	rs2132513	A	C	0.51	0.55	SKIN, GI	ESC, ESDR, LNG, IPSC, BRST, BLD, SKIN, GI, MUS, THYM, HRT, LIV, BRN			HNF4	NXN	intronic
17	907902	0.81	0.98	rs8069566	T	C	0.51	0.55	SKIN, GI	ESDR, LNG, IPSC, ESC, FAT, BRST, SKIN, LIV, GI, ADRL, HRT, MUS, THYM			RXRA	NXN	intronic
17	908269	0.8	0.98	rs11652877	C	G	0.50	0.55	GI	ESDR, LNG, IPSC, ESC, FAT, BRST, SKIN, LIV, GI, ADRL, HRT, MUS, THYM			Ets,Hic1,LBP-1	NXN	intronic
17	909164	0.81	0.99	rs9328722	G	A	0.50	0.55	FAT, SKIN, LIV	ESC, ESDR, LNG, IPSC, FAT, STRM, BRST, SKIN, LIV, GI, ADRL, HRT, MUS, PLCNT, THYM, BRN, BONE	HRT,LNG,SKIN		GR,PU.1,SPIB	NXN	intronic
17	909251	0.81	0.99	rs9328723	T	C	0.50	0.55	FAT, SKIN, LIV	ESC, ESDR, LNG, IPSC, FAT, STRM, BRST, SKIN, LIV, GI, ADRL, HRT, MUS, PLCNT, THYM, VAS, BRN, BONE	BLD,LNG,OVRY,SKIN		Crx,Hoxa13,Hoxb13 ,Hoxc10,Hoxd10,Ob ox3,Otx2,Pou3f1,SE TDB1,TATA	NXN	intronic
17	909828	0.98	0.99	rs2940810	G	A	0.49	0.50	FAT, SKIN, LIV, THYM	ESC, ESDR, LNG, IPSC, FAT, STRM, BRST, SKIN, LIV, GI, ADRL, HRT, KID, MUS, PLCNT, THYM, VAS, BRN, BONE	ESDR,IPSC,SKIN,SKIN, SKIN,KID,MUS,MUS,P LCNT,OVRY,LIV,BRST, BLD,BLD,BRN,SKIN	EGR1,USF1,STAT3	AP-1,CACD,CHD2,CT CF,E2F,Egr-1,Ets,HE Y1,NRSF,Nrf1,SETD B1,Sin3Ak-20,YY1,Zf p161	RP11-676J 12.7	intronic
17	909892	0.98	0.99	rs7218626	A	G	0.50	0.50	FAT, SKIN, LIV, THYM, BRN	ESC, ESDR, LNG, IPSC, FAT, STRM, BRST, SKIN, LIV, GI, ADRL, HRT, KID, MUS, PLCNT, VAS, BRN, BONE	ESDR,LNG,SKIN,SKIN, SKIN,KID,MUS,MUS,	USF1,STAT3	AP-4,Ets,Foxo,STAT	RP11-676J 12.7	intronic

											OVRY,BRST,BLD,BLD,SKIN				
17	910084	1	1	rs1703824	C	A	0.49	0.50	FAT, SKIN, LIV, THYM, BRN	ESC, ESDR, LNG, IPSC, FAT, STRM, BRST, SKIN, LIV, GI, ADRL, HRT, KID, MUS, PLCNT, SPLN, VAS, BRN, BONE	ESDR,SKIN,SKIN,SKIN,HRT,MUS,MUS,MUS,LNG,BLD,SKIN,SKIN		BAF155,Ets,SIX5,Znf143	RP11-676J12.7	intronic
20	48723580	1	1	rs6066825	A	G	0.30	0.36	BLD, STRM, MUS, SKIN, BRN, GI, SPLN, LIV, BONE	ESC, ESDR, LNG, IPSC, STRM, BLD, MUS, BRN, SKIN, FAT, VAS, LIV, GI, ADRL, HRT, PANC, PLCNT, THYM, OVRY, SPLN, CRVX, BONE	ESDR,BLD,BLD,BLD,BLD,BLD,SKIN,SKIN,GI,KID,LNG,MUS,MUS,GI,GI,OVRY,GI,BLD,LIV,MUS,MUS,VAS,BLD	FOXA1,FOXA2,CMYC	AP-4,Foxj1,LBP-1,RFX5,RP58	PREX1	intronic
19	58505423	0.88	0.96	rs35983901	G	A	0.06	0.21	ESC, LIV, LNG	LIV, PLCNT				SLC27A5	intronic
19	58506273	0.87	0.94	rs11670192	G	A	0.06	0.20	SKIN, LIV	LIV			Brachyury,E2A,Eomes,LBP-1,TBX5,VDR,ZEB1	SLC27A5	intronic
19	58511799	0.95	0.98	rs34415062	G	A	0.06	0.20	ESDR, IPSC, FAT, SKIN, LIV, GI, PANC, SPLN	ESDR, IPSC, BLD, SKIN, LIV, BRN, GI, ADRL, OVRY, PANC, PLCNT, HRT, MUS, SPLN	LIV,BRST	POL2	Esr2,HEY1,Pax-5,YY1	SLC27A5	missense
19	58511807	0.95	0.98	rs35350976	A	G	0.06	0.20	ESDR, IPSC, FAT, SKIN, LIV, GI, PANC, SPLN	ESDR, IPSC, BLD, SKIN, LIV, BRN, GI, ADRL, OVRY, PANC, PLCNT, HRT, MUS, SPLN	LIV,BRST	POL2	THAP1,YY1	SLC27A5	missense
19	58513279	0.95	0.98	rs56225452	C	T	0.06	0.20	ESDR, BLD, BRN, SKIN, FAT, LIV, GI, THYM, PLCNT, HRT, SPLN, CRVX	ESC, ESDR, IPSC, BRST, BLD, SKIN, FAT, LIV, BRN, GI, LNG, MUS, PLCNT, PANC, HRT, THYM, SPLN, CRVX	IPSC,BLD,MUS,THYM,PANC,MUS			252bp 3' of ZBTB45	
19	58517218	0.91	0.99	rs11545185	G	A	0.06	0.19	ESDR, ESC, LNG, IPSC, FAT, BLD, STRM, BRN, SKIN, GI, PANC, MUS, SPLN, CRVX, BRST	ESC, ESDR, IPSC, FAT, BRST, BLD, BRN, SKIN, LIV, GI, HRT, MUS, PLCNT, THYM, LNG, PANC, SPLN, CRVX	KID,THYM,LIV	CTCF	ATF3,BHLHE40,CTCF,RXRA,Rad21,SETDB1,YY1	ZBTB45	synonymous
19	58519213	1	1	rs11670864	G	T	0.06	0.20	ESC, ESDR, LNG, IPSC, FAT,	ESDR, IPSC, ESC, STRM, BRST, BLD, MUS, BRN, SKIN, FAT,	ESC,ESDR,ESDR,ESDR	CTCF,POL2,SMC3,R	E2F,ELF1,Hic1,Zfx	ZBTB45	intronic

									STRM, BRST, BLD, MUS, BRN, SKIN, VAS, LIV, GI, ADRL, HRT, KID, PANC, PLCNT, THYM, OVRY, SPLN, CRVX, BONE	LIV, GI, ADRL, LNG, PLCNT, THYM, PANC, HRT, SPLN, CRVX, BONE	,ESDR,ESC,LNG,IPSC,BRST,BLD,BLD,BLD,BLD,BLD,BLD,SKIN,SKIN,SKIN,SKIN,ADRL,BRN,BRN,HRT,GI,GI,KID,LNG,MUS,MUS,PLCNT,GI,THYM,GI,OVRY,PANC,MUS,GI,LNG,BLD,CRVX,LIV,BRST,MUS,MUS,VAS,BLD,BLD,BRN,SKIN,SKIN,LNG	AD21,TAF1,TBP,POL2S2,ELF1,MAX,CMYC,HAE2F1			
19	58522151	0.95	0.99	rs55691818	C	G	0.07	0.21	ESC, ESDR, IPSC, SKIN, GI	ESC, IPSC, SKIN, GI, LNG			ATF3,ZBTB33	2.6kb 5' of ZBTB45	
19	58537387	0.87	0.94	rs55716128	C	T	0.06	0.21	ESC, ESDR, IPSC, BLD, SKIN, BRN, GI, THYM, LNG, PLCNT, HRT, SPLN, LIV	ESC, ESDR, IPSC, BRST, BLD, SKIN, BRN, GI, MUS, THYM, LNG, PANC, PLCNT, HRT, SPLN, LIV	GI		TFIIA	5.9kb 3' of Metazoa_SRP	
19	58567729	0.8	0.9	rs73068325	C	T	0.06	0.20	SKIN, BRN, PLCNT, BLD	ADRL, BRN, BLD	BLD			MZF1	intronic
11	10242575	0.86	0.94	rs11042686	A	G	0.30	0.35		ESC, LNG, FAT, BRST, SKIN, OVRY, GI			LBP-1	SBF2	intronic
11	10243931	0.84	0.93	rs7952229	T	C	0.30	0.35		ESC, IPSC, FAT, SKIN, LNG			GATA,HMG-IY,TATA	SBF2	intronic
11	10245451	0.84	0.92	rs10743132	A	G	0.30	0.35		SKIN, VAS, LNG, OVRY, HRT			CTCF,Hdx,Pou2f2,Pou6f1,SIX5	SBF2	intronic
11	10246071	0.86	0.94	rs11042689	T	C	0.30	0.35		SKIN, HRT				SBF2	intronic
11	10246344	0.86	0.94	rs61889798	C	T	0.30	0.35		ESDR, FAT, SKIN, LNG, OVRY, PANC, HRT			CEBPA,CEBPD,CHOP::CEBPalpha,YY1	SBF2	intronic
11	10247003	0.86	0.94	rs12418690	C	A	0.30	0.35	ESC	ESDR, LNG, FAT, STRM, BRST, MUS, SKIN, GI, HRT, OVRY,			DEC,NR4A,Nkx2	SBF2	intronic

										PANC, BRN, BONE					
11	10249342	0.86	0.94	rs7933018	G	A	0.30	0.35	ESC	ESC, LNG, IPSC, FAT, STRM, MUS, SKIN, GI, BRN, BONE			Ik-2,Pou2f2	SBF2	intronic
11	10256913	0.85	0.94	rs10840379	G	A	0.30	0.34		ESC, LNG, IPSC, FAT, BRST, MUS, BRN, SKIN, VAS, LIV, GI, ADRL, PANC, HRT, OVRY, BONE				SBF2	intronic
11	10260487	0.83	0.94	rs11607621	C	T	0.30	0.34		LNG, BRST, MUS, SKIN, BRN			Zfx	SBF2	intronic
11	10262752	0.86	0.94	rs16907615	T	C	0.30	0.35		ESDR, LNG, FAT, STRM, BRST, MUS, SKIN, GI, PANC, CRVX, VAS, BONE			Gfi1,HDAC2	SBF2	intronic
11	10264563	0.83	0.94	rs2957699	T	G	0.30	0.34		LNG, STRM, MUS, BRN, SKIN, GI, PANC, BONE			Cdx,Dbx1,Fox,Foxa,Foxd3,Foxf1,Foxj1,Foxj2,Foxk1,Foxl1,Foxo,Foxp1,HDAC2,HMG-IY,Pou3f2,Pou3f3,Pou6f1,SIX5,TATA	SBF2	intronic
11	10270456	0.86	0.95	rs2957671	A	G	0.30	0.34		STRM, BRST, SKIN, HRT, GI, MUS, LNG			HNF4	SBF2	intronic
11	10279248	0.88	0.96	rs2957674	C	T	0.30	0.34		ESDR, ESC, SKIN			Mef2	SBF2	intronic
11	10279877	0.88	0.96	rs35598338	C	T	0.30	0.34		ESDR, SKIN, GI, PANC, LNG			AP-1,HNF4,Nr2f2,Pax-4,RXRA,TR4	SBF2	intronic
11	10285257	0.82	0.96	rs5789632	CTG	C	0.29	0.33	FAT, BLD, LIV, LNG	ESC, ESDR, LNG, IPSC, FAT, STRM, BRST, BLD, MUS, BRN, SKIN, LIV, GI, ADRL, HRT, PANC, THYM, OVRY, PLCNT, SPLN, CRVX, BONE			Ets,Gfi1,Mrg1::Hoxa9,STAT	SBF2	intronic
11	10286054	0.89	0.96	rs10840384	C	T	0.30	0.34	BLD, LIV, LNG	ESC, LNG, IPSC, FAT, STRM, BRST, BLD, MUS, BRN, SKIN, LIV, GI, HRT, PANC, OVRY, PLCNT, SPLN, CRVX, BONE	SKIN,BLD		p300	SBF2	intronic
11	10286487	0.87	0.97	rs10840386	G	A	0.28	0.34	BLD	ESC, LNG, FAT, STRM, BRST, BLD, SKIN, LIV, BRN, GI, HRT, PANC, PLCNT, MUS, CRVX			Egr-1,LUN-1,SP1	SBF2	intronic
11	10287444	0.93	0.97	rs6484161	G	T	0.27	0.35	ESDR, SKIN, GI	ESDR, LNG, IPSC, FAT, BRST, BLD, MUS, SKIN, LIV, BRN, GI,	BLD,SKIN,GI,MUS,CR	ZNF263	HMG-IY,p300	SBF2	intronic

										ADRL, HRT, OVRY, PLCNT, SPLN, CRVX, BONE	VX,MUS,BLD,BLD,SKIN				
11	10289926	0.93	0.98	rs74794749	G	A	0.27	0.35	ESC, SKIN, BRN, GI, LNG, PLCNT, SPLN, LIV, BLD	ESC, ESDR, LNG, IPSC, FAT, BRST, BLD, BRN, SKIN, LIV, GI, ADRL, HRT, MUS, PLCNT, THYM, OVRY, PANC, SPLN	OVRY,LIV		AP-1,CCNT2,CHD2,ERalpha-a,Egr-1,Irf,Klf4,Klf7,MOVO-B,RXRA,SP1,TATA,THAP1	SBF2	intronic
11	10290242	0.94	0.98	rs11042721	T	C	0.27	0.35	ESC, SKIN, BRN, GI, LNG, PLCNT, SPLN, LIV, BLD	ESC, ESDR, LNG, IPSC, FAT, BRST, BLD, BRN, SKIN, LIV, GI, ADRL, HRT, MUS, PLCNT, THYM, OVRY, PANC, SPLN	BLD,BLD,HRT,BLD		Foxp3	SBF2	intronic
11	10296599	0.98	0.99	rs12418494	T	C	0.26	0.35	ESC, ESDR, IPSC, FAT, BLD, SKIN, GI, MUS, LNG	ESC, ESDR, IPSC, FAT, BRST, BLD, SKIN, GI, PANC, PLCNT, CRVX, LNG, BONE	BLD,BLD		Dobox4,Foxl1,Myc,Nanog,TATA	2.4kb 5' of SBF2	
11	10301479	0.98	0.99	rs4641466	G	C	0.29	0.35	ESC, ESDR, LNG, IPSC, FAT, STRM, BLD, MUS, SKIN, BRN, GI, PLCNT, CRVX, BRST	ESC, ESDR, LNG, IPSC, FAT, STRM, BRST, BLD, MUS, BRN, SKIN, LIV, GI, ADRL, KID, PANC, PLCNT, CRVX, BONE	LNG,GI		Foxo,HNF4,RXRA,Sox	1.2kb 3' of RP11-351I24.1	
11	10301931	1	1	rs4399321	A	G	0.29	0.35	ESC, ESDR, LNG, IPSC, FAT, STRM, BRST, BLD, MUS, BRN, SKIN, VAS, LIV, GI, PANC, PLCNT, THYM, HRT, CRVX, BONE	ESC, ESDR, IPSC, FAT, STRM, BRST, BLD, MUS, BRN, SKIN, LIV, GI, ADRL, KID, PANC, LNG, PLCNT, SPLN, CRVX, VAS, BONE	ESDR,LNG,BRST,BLD,BLD,SKIN,SKIN,SKIN,LNG,MUS,MUS,LNG,CRVX,BRST,MUS,BRN,SKIN,SKIN,LNG	CEBPB	Crx,ERalpha-a,HNF1,RAR	725bp 3' of RP11-351I24.1	
		0.8	0.93	rs74767935	C	A	0.04	0.14	ESC, ESDR, FAT, STRM, SKIN, LIV, BRN, PANC, MUS, GI, SPLN, CRVX, BLD, BONE	ESC, ESDR, LNG, IPSC, FAT, STRM, BRST, BLD, MUS, SKIN, VAS, LIV, BRN, GI, ADRL, PLCNT, THYM, HRT, OVRY, PANC, SPLN, CRVX, BONE	ESC,PANC		SP1,Znf143	ITIH4	
3	52832920	0.84	0.99	rs79664140	A	G	0.04	0.15	ESDR, SKIN, LIV, BRN, GI, MUS, HRT, SPLN, BONE	ESC, ESDR, IPSC, SKIN, VAS, LIV, BRN, GI, ADRL, MUS, THYM, HRT, LNG, PANC, SPLN, CRVX, BONE	MUS		TATA	MUSTN1	
3	52834507	0.84	0.99	rs76029595	C	T	0.04	0.15	ESDR, SKIN, FAT, BRN, GI, LNG, MUS, HRT, OVRY, SPLN, LIV	ESC, ESDR, LNG, IPSC, FAT, BRST, BLD, MUS, SKIN, VAS, LIV, BRN, GI, ADRL, HRT, PLCNT, THYM, OVRY, PANC, SPLN			Gm397,TCF12	MUSTN1	intronic

3	52837085	0.85	1	rs1139106	G	A	0.04	0.15	ESDR, LNG, MUS, SPLN	ESDR, BRST, BLD, SKIN, VAS, LIV, BRN, GI, HRT, MUS, THYM, OVRY, PANC, SPLN, LNG	MUS,MUS,MUS		HNF4	MUSTN1	intronic
3	52839968	0.9	1	rs11626	C	T	0.04	0.15	MUS, SPLN	ESDR, BLD, SKIN, LIV, BRN, GI, MUS, PLCNT, THYM, SPLN	CRVX		DMRT1,DMRT5,DMRT7,Sox	MUSTN1	intronic
3	52841530	0.9	1	rs11718060	G	A	0.04	0.15	BRN, MUS, GI, SPLN	ESDR, IPSC, BRST, BLD, MUS, SKIN, FAT, LIV, BRN, GI, ADRL, HRT, PLCNT, THYM, LNG, SPLN	ADRL,MUS,LNG		COMP1,Hoxa7	TMEM110-MUSTN1	intronic
3	52846724	1	1	rs2001732	C	T	0.04	0.13	BLD, FAT, BRN, GI, PANC, MUS	ESDR, IPSC, FAT, ESC, BLD, SKIN, VAS, LIV, BRN, GI, HRT, PANC, LNG, MUS, THYM	BLD	EBF1	CTCF,ERalpha-a,HEN1,Hand1,RXRA,SMC3	TMEM110-MUSTN1	intronic
3	52851096	0.87	0.99	rs13080929	A	C	0.04	0.15	ESDR, GI	SKIN, LIV, MUS, THYM, LNG			KAP1,Myc,PU.1,Pax-5,ZNF263	TMEM110-MUSTN1	intronic
3	52856402	0.82	-0.96	rs4687676	C	T	0.96	0.85	LNG, SPLN	BLD, ADRL, GI, THYM, LNG, PANC, HRT, SPLN			ATF3,HDAC2,Klf4,ZID	TMEM110-MUSTN1	intronic
3	52856459	0.81	-0.96	rs4687556	T	C	0.94	0.85	LNG, SPLN	BLD, ADRL, GI, THYM, LNG, PANC, HRT, SPLN			GR	TMEM110-MUSTN1	intronic
3	52857410	0.82	-0.96	rs9637477	G	C	0.94	0.85	GI, LNG, SPLN	BLD, BRN, GI, MUS, THYM, HRT, LNG, PANC	MUS		CTCF,Hand1,NRSF,Pax-6,Rad21	TMEM110-MUSTN1	intronic
3	52861918	0.88	0.94	rs77572134	T	C	0.03	0.13	SKIN, SPLN	ESDR, FAT, BRN, GI, MUS, PANC, HRT, SPLN			Myc,SETDB1	TMEM110-MUSTN1	intronic
3	52862393	0.89	0.95	rs11708390	C	A	0.03	0.13	SPLN	ESDR, FAT, BRN, GI, PANC, HRT, SPLN			Sin3Ak-20,Zfp691	TMEM110-MUSTN1	intronic
3	52862839	0.88	-0.95	rs11130329	C	A	0.97	0.86	SPLN	FAT, BRN, PANC, HRT, SPLN			En-1,NF-kappaB,Ncx,Nkx6-1,TATA,Zfp410	TMEM110-MUSTN1	intronic
3	52870068	0.89	0.95	rs112847844	G	GA	0.03	0.13		ESDR, BLD, SKIN, LIV, BRN, GI, THYM, LNG, PANC, HRT, MUS	BLD,BLD,GI,GI,LIV		Ets,Foxo,Foxp1,HDAC2,Irf,Mef2,p300	TMEM110-MUSTN1	intronic

6	22073425	0.87	1	rs10484390	G	A	0.67	0.03	GI, HRT, LNG, BLD	ESDR, ESC, FAT, BRST, BLD, BRN, SKIN, LIV, GI, ADRL, HRT, KID, LNG, CRVX			DMRT3,Pbx-1	LINC00340	intronic
6	22073692	0.91	1	rs12523974	G	A	0.67	0.03	GI, HRT, LNG, BLD	ESDR, FAT, ESC, BRST, BLD, BRN, SKIN, LIV, GI, ADRL, HRT, KID, LNG, CRVX	ESDR,BLD,BLD			LINC00340	intronic
6	22073964	0.91	1	rs10946506	C	T	0.68	0.03	GI, HRT, LNG, BLD	FAT, BRST, BLD, BRN, SKIN, LIV, GI, ADRL, KID, LNG	KID,OVRY,BLD		CEBPA,CEBPB,ERalpha-a,RORalpha1	LINC00340	intronic
6	22074816	1	1	rs73389647	G	T	0.67	0.03	LNG, GI, BLD	BLD, BRN, GI			Gcm1,PLAG1,STAT,VDR,ZNF219,Zfp281,Znf143	LINC00340	intronic
6	22080671	1	1	rs73389656	G	T	0.68	0.03		FAT, ESC, STRM, BRST, MUS, SKIN, VAS, LIV, GI, THYM, BLD, BONE			Cphx,Foxa	LINC00340	intronic
6	22081261	1	1	rs113753759	A	C	0.68	0.03		STRM, BRST, VAS, LIV, GI, THYM, OVRY			Hand1,Pax-6,Zbtb12	LINC00340	intronic
6	22085429	1	1	rs12530233	A	C	0.67	0.03		GI			E2F,Irx,Mef2,Nanog,Pou1f1,Pou2f2,Pou3f3	LINC00340	intronic
6	22092680	1	1	rs9368399	C	T	0.66	0.03		GI			DMRT1,DMRT7,GR,RFX5	LINC00340	intronic
6	22094707	0.95	1	rs12528963	C	T	0.67	0.03		ESC, GI, BLD			Mxi1,NF-E2,Sin3Ak-20,TFE	LINC00340	intronic
6	22095377	1	1	rs16885582	T	A	0.66	0.03		ESDR, ESC, FAT, BLD			BCL,Irf,Maf,Nkx2,PU.1,Pax-5,RXRA,SETDB1,STAT	LINC00340	intronic
6	22104940	0.9	0.95	rs9466265	C	T	0.65	0.03	GI	BRN, THYM, LNG				LINC00340	intronic
6	22105624	0.95	1	rs5874852	TG	T	0.65	0.03		BRN, THYM			AP-3,CEBPB,CEBPD,Hltf,Ik-2,STAT,TATA	LINC00340	intronic

6	22106938	0.95	1	rs9466266	C	T	0.64	0.03		BRST, BRN			AIRE,Foxa,Irx	LINC00340	intronic
7	45110732	1	1	rs7810512	A	C	0.23	0.28	ESC, ESDR, LNG, IPSC, FAT, STRM, BRST, BLD, MUS, BRN, SKIN, VAS, LIV, GI, ADRL, KID, PANC, THYM, HRT, SPLN, CRVX, BONE	ESC, ESDR, LNG, IPSC, FAT, STRM, BRST, BLD, MUS, BRN, SKIN, LIV, GI, ADRL, HRT, PLCNT, THYM, PANC, SPLN, CRVX, VAS, BONE	BLD,BLD,BLD,BLD,BLD,BLD,GI,GI,THYM,MUS,BLD,LIV	ELF1		TBRG4	intronic
6	29717217	0.93	0.97	rs7759272	T	C	0.27	0.12	ESC, BLD	ESC, BLD			Cart1,Irf,Nkx2,PU.1,Pou2f2	5.6kb 5' of HLA-F	
6	29717248	0.9	0.95	rs7738919	C	T	0.26	0.12	ESC, BLD	ESC, BLD			CDP,Foxp1,GATA,Maf,Mef2,Pax-4,Pou1f1,Pou2f2,Pou6f1,Sox,Zfp105	5.5kb 5' of HLA-F	
6	29717249	0.9	0.95	rs7738786	A	G	0.26	0.12	ESC, BLD	ESC, BLD			CDP,Foxp1,GATA,Maf,Mef2,Pou1f1,Pou6f1,Sox	5.5kb 5' of HLA-F	
6	29717328	0.93	0.97	rs7739360	G	T	0.27	0.12	ESC, BLD	ESC, BLD				5.4kb 5' of HLA-F	
6	29717378	0.93	0.97	rs7739388	G	C	0.27	0.12	ESC, BLD	ESC, BLD			LUN-1,NRSF	5.4kb 5' of HLA-F	
6	29717673	0.93	0.97	rs9258156	C	A	0.27	0.12	ESC	BLD			E2A,Egr-1,Irf,PPAR,ZEB1	5.1kb 5' of HLA-F	
6	29717711	0.93	0.97	rs9258157	T	A	0.27	0.12		ESC, BLD, BRN			Pax-4,Pax-8	2.5kb 5' of HLA-F	
6	29720403	0.93	0.97	rs9258158	A	G	0.27	0.12	BLD	ESC, BLD, BRN			Fox,Zfp105	2.4kb 5' of HLA-F	
6	29720708	0.93	0.97	rs9258160	T	G	0.27	0.12	BLD, BRN	ESC, BLD, GI			HNF4,Myb	2.1kb 5' of	

														HLA-F	
6	29721037	0.93	0.97	rs9258163	G	C	0.27	0.12	BLD, BRN	ESC, BLD, GI, SPLN	ESC,ESDR,ESC,IPSC,BLD,BLD,BLD,SKIN,GI	CTCF,RAD21,ZEB1	LUN-1,Mxi1,Smad3	1.7kb 5' of HLA-F	
6	29721092	0.93	0.97	rs9258164	G	A,C,T	0.27	0.12	BLD, BRN	ESC, BLD, GI, SPLN	ESC,ESDR,ESC,IPSC,IPSC,BLD,BLD,BLD,BLD,BLD,BLD,SKIN,GI,SKIN	CTCF,RAD21,ZEB1		1.7kb 5' of HLA-F	
6	29721107	0.93	0.97	rs9258165	G	A	0.27	0.12	BLD, BRN	ESC, BLD, GI, SPLN	ESC,ESDR,ESC,IPSC,IPSC,BLD,BLD,BLD,BLD,BLD,SKIN,GI,SKIN	CTCF,RAD21,ZEB1	Zbtb3	1.7kb 5' of HLA-F	
6	29721123	0.93	0.97	rs9258166	G	C	0.27	0.12	BLD, BRN	ESC, BLD, GI, SPLN	ESDR,ESC,BLD,BLD,BLD,SKIN	CTCF,RAD21,ZEB1	BCL,NRSF,Nanog,PPAR	1.7kb 5' of HLA-F	
6	29721196	0.93	0.97	rs9258167	G	A	0.27	0.12	BLD, BRN, BRST	ESC, BLD, GI, SPLN		CTCF	COMP1,Mef2,PEBP,SP2	1.6kb 5' of HLA-F	
6	29721226	0.93	0.97	rs9258168	C	T	0.27	0.12	BLD, BRN, GI, BRST	ESC, BLD, GI, SPLN		CTCF	GATA	1.5kb 5' of HLA-F	
6	29721350	0.93	0.97	rs3757324	G	T	0.27	0.12	BLD, BRN, GI, BRST	ESC, BLD, GI, SPLN			Cdx2,Cphx,Hoxa9,Hoxd10,Hoxd8,Ncx,Nkx6-1,OTX	1.4kb 5' of HLA-F	
6	29721425	0.93	0.97	rs3757325	A	T	0.27	0.12	BLD, BRN, GI, BRST	ESC, BLD, GI, HRT, SPLN			SRF,p53	1.3kb 5' of HLA-F	
6	29721472	0.93	0.97	rs3757326	C	G	0.27	0.12	IPSC, BLD, BRN, GI, BRST	ESC, BLD, SKIN, FAT, GI, HRT, SPLN			Arid5b	1.3kb 5' of HLA-F	
6	29721755	0.93	0.97	rs9258169	T	G	0.27	0.12	IPSC, BLD, BRN, GI, BRST	ESC, BLD, SKIN, FAT, BRN, GI, HRT, SPLN			Dbx1,Hoxa10,Hoxd10,Pax-4,TATA	1kb 5' of HLA-F	
6	29721813	0.93	0.97	rs9258170	T	G	0.27	0.12	IPSC, BLD, BRN, GI, BRST	ESC, BLD, SKIN, FAT, BRN, GI, HRT, SPLN			Brachyury,Eomes,H	961bp 5'	

													NF4,Rhox11,SRF,TBX5	of HLA-F	
6	29721940	0.93	0.97	rs9258171	G	T	0.27	0.12	IPSC, BLD, BRN, GI, LNG, SPLN, BRST	ESC, BLD, SKIN, FAT, BRN, GI, HRT, SPLN	BLD,BLD	PU1	Ets,Pax-5,Zfp691,p300	834bp 5' of HLA-F	
6	29722196	0.93	0.97	rs9258172	C	T	0.27	0.12	IPSC, BLD, FAT, BRN, GI, MUS, LNG, HRT, THYM, SPLN, BRST	ESC, BLD, SKIN, FAT, BRN, GI, HRT, SPLN, BONE	BLD		Foxf1,Foxi1,Foxl1,Pou2f2,Pou3f2,TATA	578bp 5' of HLA-F	
6	29722225	0.93	0.97	rs9258173	G	T	0.27	0.12	IPSC, BLD, FAT, BRN, GI, MUS, LNG, HRT, THYM, SPLN, BRST	ESC, BLD, SKIN, FAT, BRN, GI, HRT, SPLN, BONE	BLD		DMRT1,DMRT3,DMRT4,DMRT5,DMRT7,MIF-1,RFX5	549bp 5' of HLA-F	
6	29722249	0.93	0.97	rs9258174	T	C	0.27	0.12	IPSC, BRST, BLD, FAT, BRN, GI, MUS, LNG, HRT, THYM, SPLN	ESC, IPSC, BLD, SKIN, FAT, BRN, GI, HRT, SPLN, BONE	BLD		AIRE,AP-1,E2F,Irf,NF-Y,Nobox,Pbx3,RFX5,SP1,SP2,TATA	525bp 5' of HLA-F	
6	29722348	0.93	0.97	rs9258175	G	A	0.27	0.12	IPSC, BRST, BLD, SKIN, FAT, BRN, GI, MUS, LNG, HRT, THYM, SPLN	ESC, IPSC, BLD, SKIN, FAT, BRN, GI, HRT, SPLN, BONE			SZF1-1	426bp 5' of HLA-F	
6	29723320	0.97	1	rs2075682	A	T	0.27	0.12	ESC, ESDR, LNG, IPSC, FAT, STRM, BRST, BLD, MUS, BRN, SKIN, VAS, LIV, GI, ADRL, HRT, KID, PANC, PLCNT, THYM, OVRY, SPLN, CRVX, BONE	ESC, ESDR, LNG, IPSC, FAT, BLD, STRM, MUS, BRN, SKIN, LIV, GI, ADRL, HRT, PLCNT, THYM, PANC, SPLN, BRST, BONE	ESC,ESDR,ESDR,ESDR,ESC,IPSC,IPSC,BRST,BLD,BLD,BLD,BLD,BLD,BLD,BLD,SKIN,SKIN,SKIN,SKIN,ADRL,BRN,HRT,GI,GI,KID,LNG,MUS,MUS,PLCNT,GI,THYM,GI,OVRY,PANC,MUS,GI,LNG,BLD,LIV,BRST,MUS,MUS,VAS	CFOS,ELF1,NFKB,RFX5,SP1,TBP,POL2	Ehf,Elf3,STAT	HLA-F	

											,BLD,BRN,SKIN,SKIN,LNG				
6	29723363	0.97	1	rs2072896	C	G	0.27	0.12	ESC, ESDR, LNG, IPSC, FAT, STRM, BRST, BLD, MUS, BRN, SKIN, VAS, LIV, GI, ADRL, HRT, KID, PANC, PLCNT, THYM, OVRY, SPLN, CRVX, BONE	ESC, ESDR, LNG, IPSC, FAT, BLD, STRM, MUS, BRN, SKIN, LIV, GI, ADRL, HRT, PLCNT, THYM, PANC, SPLN, BRST, BONE	ESC,ESDR,ESDR,ESDR,ESC,LNG,IPSC,IPSC,BRST,BLD,BLD,BLD,BLD,BLD,BLD,BLD,SKIN,SKIN,SKIN,SKIN,ADRL,BRN,HRT,GI,GI,KID,LNG,MUS,MUS,PLCNT,GI,THYM,GI,OVRY,PANC,MUS,GI,LNG,BLD,LIV,BRST,MUS,MUS,VAS,BLD,BRN,SKIN,SKIN,LNG	POL2,CFOS,ELF1,NFKB,OCT2,POU2F2,RFX5,SP1,TBP,POL24H8,TAF1,YY1		HLA-F	5'-UTR
6	29723526	0.97	1	rs2076183	G	A	0.27	0.12	ESC, ESDR, LNG, IPSC, FAT, STRM, BRST, BLD, MUS, BRN, SKIN, VAS, LIV, GI, ADRL, HRT, KID, PANC, PLCNT, THYM, OVRY, SPLN, CRVX, BONE	ESC, ESDR, LNG, IPSC, FAT, BLD, STRM, MUS, BRN, SKIN, LIV, GI, ADRL, HRT, PLCNT, THYM, PANC, SPLN, BRST, VAS, BONE	ESC,ESDR,ESC,IPSC,BRST,BLD,BLD,BLD,BLD,BLD,SKIN,SKIN,MUS,MUS,GI,MUS,BLD,SKIN	CTCF,POL2,OCT2,POU2F2,TAF1,TBP,POL24H8,YY1,RFX5	AP-1,ATF3,E2A,ERalpha-a,Pou2f2,RREB-1,ZEB1	HLA-F	synonymous
6	29723936	0.99	1	rs2076182	A	C	0.28	0.12	ESC, ESDR, LNG, IPSC, FAT, STRM, BRST, BLD, MUS, BRN, SKIN, VAS, LIV, GI, ADRL, HRT, KID, PANC, PLCNT, THYM, OVRY, SPLN, CRVX, BONE	ESC, ESDR, LNG, IPSC, FAT, BRST, BLD, STRM, MUS, BRN, SKIN, LIV, GI, ADRL, HRT, PLCNT, THYM, PANC, SPLN, VAS, BONE	IPSC,BLD,BLD,BLD	POL2,POL24H8,TAF1	AP-2,ATF3,FXR,HNF4,RXRA,VDR	HLA-F	intronic

6	29723967	0.97	1	rs2076181	C	G	0.27	0.12	ESC, ESDR, LNG, IPSC, FAT, STRM, BRST, BLD, MUS, BRN, SKIN, VAS, LIV, GI, ADRL, HRT, KID, PANC, PLCNT, THYM, OVRY, SPLN, CRVX, BONE	ESC, ESDR, LNG, IPSC, FAT, BRST, BLD, STRM, MUS, BRN, SKIN, LIV, GI, ADRL, HRT, PLCNT, THYM, PANC, SPLN, VAS, BONE	BLD,BLD,BLD,BLD	POL2,POL24H8,TAF1	CACD,ERalpha-a,GATA,GLI,RREB-1,SP1,Zfp281,Zfp740,Zic	HLA-F	intronic
6	29724557	0.99	1	rs9258186	A	G	0.27	0.12	ESC, ESDR, LNG, IPSC, FAT, STRM, BRST, BLD, MUS, BRN, SKIN, LIV, GI, ADRL, PANC, PLCNT, THYM, HRT, OVRY, SPLN, CRVX, BONE	ESC, ESDR, IPSC, FAT, BRST, BLD, STRM, BRN, SKIN, VAS, LIV, GI, ADRL, HRT, MUS, PLCNT, THYM, LNG, OVRY, PANC, SPLN, BONE	ESC,BLD,BLD,BLD,BLD	WHIP	CTCFL,CTCF,NRSF,RXRA,Sin3Ak-20	HLA-F	intronic
		0.96	0.99	rs140848774	AAATTTCTGAGGG	A	0.26	0.11	ESC, ESDR, IPSC, FAT, STRM, BRST, BLD, MUS, BRN, SKIN, LIV, GI, ADRL, PANC, LNG, PLCNT, THYM, HRT, OVRY, SPLN, CRVX, BONE	ESC, ESDR, IPSC, FAT, BRST, BLD, STRM, SKIN, VAS, LIV, BRN, GI, ADRL, HRT, MUS, PLCNT, THYM, LNG, OVRY, PANC, SPLN	BLD,BLD	WHIP	AIRE,CEBPD,EWSR1-FLI1,Pax-5,TCF12	HLA-F	intronic
		0.87	0.99	rs63428760	C	A	0.21	0.11	ESC, ESDR, IPSC, FAT, STRM, BRST, BLD, MUS, BRN, SKIN, LIV, GI, ADRL, PANC, PLCNT, THYM, HRT, LNG, OVRY, SPLN, CRVX, BONE	ESC, ESDR, IPSC, FAT, BRST, BLD, STRM, SKIN, VAS, LIV, BRN, GI, ADRL, HRT, MUS, PLCNT, THYM, LNG, OVRY, PANC, SPLN	BLD			HLA-F	intronic
6	29724952	0.99	1	rs2072898	T	G	0.27	0.12	ESDR, ESC, IPSC, STRM, BRST, BLD, BRN, SKIN, FAT, LIV, GI, ADRL, MUS, PLCNT, THYM, HRT, LNG, OVRY, PANC, SPLN, CRVX, BONE	ESDR, IPSC, FAT, ESC, BRST, BLD, SKIN, VAS, LIV, BRN, GI, ADRL, MUS, PLCNT, THYM, HRT, LNG, OVRY, PANC, SPLN	BLD,BLD,BLD			HLA-F	intronic

6	29725336	0.99	1	rs2076177	C	T	0.27	0.12	ESDR, ESC, IPSC, STRM, BRST, BLD, BRN, SKIN, FAT, LIV, GI, MUS, PLCNT, THYM, HRT, LNG, OVRY, PANC, SPLN, CRVX	ESDR, IPSC, BRST, BLD, SKIN, FAT, VAS, LIV, BRN, GI, MUS, PLCNT, THYM, HRT, LNG, OVRY, PANC, SPLN	BLD,BLD,BLD		HNF4,PRDM1,RXRA, Znf143,p53	HLA-F	intronic
6	29725722	0.97	1	rs2235383	A	G	0.27	0.12	ESDR, IPSC, BRST, BLD, SKIN, FAT, LIV, BRN, GI, MUS, PLCNT, THYM, HRT, LNG, OVRY, SPLN, CRVX	ESDR, BRST, BLD, SKIN, FAT, VAS, LIV, BRN, GI, MUS, PLCNT, THYM, HRT, LNG, OVRY, PANC, SPLN	BLD,BLD,BLD,BLD,BLD			HLA-F	intronic
6	29726793	0.99	1	rs3734813	A	G	0.27	0.12	ESDR, BRST, BLD, BRN, GI, SPLN, CRVX	BRST, BLD, SKIN, FAT, LIV, BRN, GI, HRT, LNG, MUS, SPLN	BLD	POL2,POL24H8	Nr2e3	HLA-F	intronic
6	29726903	0.96	0.99	rs3734814	A	C	0.27	0.11	ESDR, BLD, BRN, GI, SPLN, CRVX	BRST, BLD, SKIN, FAT, BRN, GI, HRT, LNG, MUS, SPLN	BLD,BLD	POL2,POL24H8	Nkx2	HLA-F	missense
6	29726904	0.96	0.99	rs3734815	A	T	0.27	0.11	ESDR, BLD, BRN, GI, SPLN, CRVX	BRST, BLD, SKIN, FAT, BRN, GI, HRT, LNG, MUS, SPLN	BLD,BLD	POL2,POL24H8		HLA-F	missense
6	29727823	0.99	1	rs9258187	T	G	0.27	0.12	BLD, GI	BRST, BLD, SKIN, BRN, GI, HRT, LNG, SPLN	BLD,BLD,BLD		MZF1::1-4,PU.1	HLA-F	intronic
6	29728340	0.97	1	rs3817827	T	A,C, G	0.26	0.11	BLD	BLD, SKIN, BRN, GI, HRT, LNG, SPLN	BLD,BLD,BLD	POL2,POL24H8		HLA-F	intronic
6	29728341	0.97	1	rs3817826	G	T	0.26	0.11	BLD	BLD, SKIN, BRN, GI, HRT, LNG, SPLN	BLD,BLD,BLD	POL2,POL24H8	HNF4,Zfx	HLA-F	intronic
6	29728468	1	1	rs2272874	T	C	0.27	0.12	BLD	BLD, SKIN, BRN, GI, HRT, LNG, SPLN	BLD,BLD	POL2,POL24H8	Smad	HLA-F	intronic
6	29728877	1	1	rs3736697	T	C	0.27	0.12	BLD	BLD, SKIN, GI, LNG, HRT, SPLN	BLD,THYM	POL2,POL24H8	Sox	HLA-F	intronic
6	29729072	1	1	rs3736694	A	C	0.27	0.12	BLD	BLD, SKIN, GI, SPLN	BLD,BLD,BLD		SEF-1,SP1	HLA-F	intronic
6	29730121	0.84	1	rs7742523	G	A	0.27	0.13	BLD	BLD, SKIN, VAS, HRT, GI, SPLN	BLD,BLD	POL2	TCF11::MafG	HLA-F	intronic
6	29730627	0.9	0.96	rs9258192	C	G	0.27	0.11	BLD	BLD, SKIN, VAS, HRT, GI, SPLN	BLD		BRCA1,GR,PLAG1	HLA-F	intronic
6	29731608	0.9	0.95	rs6910733	A	G	0.27	0.12		BLD, SKIN, HRT, GI, SPLN	BLD,BLD			HLA-F	intronic
6	29732747	0.9	0.95	rs7753946	A	G	0.27	0.12		BLD, SKIN			PLZF	HLA-F	intronic

6	29732986	0.9	0.95	rs7754692	G	C	0.27	0.12		BLD, SKIN			BDP1,Hand1,Pax-5,Pax-6,Zbtb12,p300	HLA-F	intronic
6	29733884	0.9	0.95	rs7741597	T	C	0.27	0.12		FAT, BRST, BLD, SKIN		JUND	Foxf1,Foxi1,Foxj2,Foxl1,Foxp1,Mef2,Pou1f1,Pou2f2,Pou3f2,TATA,TEF	HLA-F	intronic
6	29734023	0.9	0.95	rs7773358	C	G,T	0.27	0.12		FAT, BRST, BLD, SKIN, HRT, GI				HLA-F	intronic
6	29734166	0.9	0.95	rs7755571	T	C	0.27	0.12		FAT, BRST, BLD, SKIN, HRT, GI, MUS	BLD,SKIN		DMRT5,Myc	HLA-F	intronic
6	29735241	0.9	0.95	rs9258203	C	T	0.27	0.12		BRST, BLD			TATA	HLA-F	intronic
6	29736046	0.9	0.95	rs9258205	T	C	0.27	0.12		BRST, BLD, SKIN, HRT			Foxp1	HLA-F	intronic
6	29736666	0.9	0.95	rs9258207	C	T	0.27	0.12	BLD	BRST, BLD, SKIN, HRT				HLA-F	intronic
6	29737307	0.9	0.95	rs7751815	C	T	0.27	0.12	BLD	BRST, SKIN, HRT			Cdc5,ERalpha-a	HLA-F	intronic
6	29738403	0.9	0.95	rs9258211	G	A	0.27	0.12	GI, SPLN	LNG, BRST, BLD, SKIN, GI, HRT, MUS	BRST,BLD,BLD,BLD,SKIN	CTCF	EBF,NF-kappaB,NRSF	HLA-F	intronic
6	29738449	0.9	0.95	rs9258212	T	C	0.27	0.12	GI, SPLN	LNG, BRST, BLD, SKIN, GI, HRT	BLD,BLD,BLD		ERalpha-a,Esr2,GR,HNF4,RXRA	HLA-F	intronic
6	29739266	0.9	0.95	rs9258214	G	A	0.27	0.12	LNG, GI	LNG, BRST, SKIN, LIV	LNG	FOXA1,FOXA2		HLA-F-AS1	intronic
6	29739530	0.9	0.95	rs9258215	A	G	0.27	0.12	LNG, GI	LNG, BRST, SKIN, LIV				HLA-F-AS1	intronic
6	29739753	0.84	0.93	rs9258216	C	T	0.27	0.11	LNG, GI	LNG, BRST, SKIN, LIV			EBF,HDAC2,NRSF	HLA-F-AS1	intronic
6	29739883	0.84	0.95	rs9258217	C	T	0.27	0.11	LNG, GI	LNG, BRST, SKIN, LIV			BATF,SIX5	HLA-F-AS1	intronic
6	29740251	0.87	0.95	rs9258218	G	A	0.27	0.11	GI	LNG, BRST, SKIN, LIV			BDP1,VDR	HLA-F-AS1	intronic
7	99868964	0.94	0.98	rs10235630	G	A	0.26	0.44	ESDR	ESDR, IPSC			DMRT1,DMRT7,STAT,TATA	2.9kb 3' of CYP3A43	
7	99873449	0.94	0.98	rs2099446	T	C	0.29	0.44					TATA	2.5kb 3' of OR2AE1	

7	99878095	0.94	0.98	rs2527881	G	A	0.26	0.44		ESDR, PLCNT			CHOP::CEBPalpha,NF-kappaB	TRIM4	
7	99879803	1	1	rs2527927	G	A	0.27	0.44	ESDR					TRIM4	
7	99884014	1	1	rs2099445	G	A	0.30	0.44	ESDR				Myc,RFX5	TRIM4	
7	99889448	0.98	0.99	rs2572011	C	T	0.27	0.44	ESDR	ADRL				TRIM4	
7	99896257	0.95	0.98	rs2527920	G	C	0.27	0.44	ESDR	IPSC, BLD, BRN, SPLN			AIRE,TEF-1	TRIM4	intronic
7	99897849	0.95	0.98	rs2527918	T	G	0.27	0.44	ESDR, BLD, SKIN, SPLN	ESDR, ESC, BRST, BLD, SKIN, BRN, SPLN	IPSC	BATF,EBF1,PU1	EBF,Pax-4,Rad21,Zic	TRIM4	intronic
7	99902419	0.95	0.97	rs2527912	T	G	0.27	0.44	SPLN	ESC, ESDR			Foxp1,Mef2,Pax-4	TRIM4	intronic
7	99903276	0.95	0.98	rs2247762	T	C	0.27	0.44	SPLN				ZEB1	TRIM4	synonymous
7	99907431	0.93	0.97	rs2572002	G	A	0.28	0.44					AP-1,Evi-1,HNF1,Hmx,Homez,Ncx,Pou1f1,STAT	TRIM4	intronic
7	99907600	0.93	0.97	rs7781442	G	A	0.27	0.44	ESDR	BLD			CEBPA,CEBPB,SIX5	TRIM4	intronic
7	99911701	0.92	0.96	rs2572000	C	T	0.27	0.44	ESDR, BLD	BRST, BLD, LIV, BRN, GI, PANC, OVRY	PLCNT	CEBPB	Mrg1::Hoxa9,Pou5f1,ZBTB33	TRIM4	intronic
7	99912611	0.92	0.96	rs4727439	A	C	0.27	0.43	ESDR	BRST, BLD, LIV, BRN, GI, PANC			GCNF,HNF6,Nanog,Pou5f1,Sox	TRIM4	intronic
7	99913940	0.92	0.96	rs1865472	A	T	0.27	0.44	BLD, SKIN, GI	FAT, BRST, BLD, SKIN, LIV, BRN, GI, ADRL, PANC, THYM, HRT, SPLN	BLD,BLD,SKIN,SKIN,ADRL,LNG,BLD		CCNT2,GATA,Hltf,TAL1,TATA	TRIM4	intronic
7	99920468	0.92	0.96	rs2571995	C	G	0.27	0.44	ESC, ESDR, LNG, IPSC, FAT, STRM, BRST, BLD, MUS, BRN, SKIN, VAS, LIV, GI, ADRL, HRT, KID, PANC, OVRY, PLCNT, SPLN, CRVX, BONE	ESDR, ESC, IPSC, FAT, STRM, BRST, BLD, MUS, BRN, SKIN, VAS, LIV, GI, ADRL, HRT, KID, PANC, LNG, PLCNT, THYM, OVRY, SPLN, CRVX, BONE	BLD,BLD	SRF	AP-1,Gfi1,Gfi1b,NF-Y,NRSF,SP1	867bp 5' of TRIM4	

7	99922940	0.92	0.96	rs2527908	G	A	0.27	0.44					Irf,TATA	328bp 3' of GJC3	
7	99926008	0.92	0.96	rs1121592	T	C	0.27	0.44		BRST, SKIN, LIV			Foxc1,Foxf1,TEF	GJC3	intronic
7	99926866	0.92	0.96	rs2527904	C	T	0.27	0.44	SKIN	ESC, FAT, BRST, BLD, SKIN, LNG, LIV			Foxa,Ik-3,STAT	GJC3	intronic
7	99927448	0.92	0.96	rs2527902	G	A	0.27	0.44	BLD, THYM, GI	ESC, FAT, BRST, BLD, SKIN, ADRL, PLCNT, MUS, GI, LNG, LIV				GJC3	intronic
7	99927618	0.92	0.96	rs2527900	A	G	0.27	0.44	IPSC, BLD, THYM, GI	ESC, ESDR, FAT, BRST, BLD, SKIN, ADRL, PLCNT, MUS, GI, LNG, LIV			Rad21	GJC3	intronic
1	22365104	0.96	0.98	rs11810751	A	C	0.22	0.16	SPLN	ESC, ESDR, IPSC, FAT, BRN, LNG, PANC, SPLN, BLD		CTCF,ELF1,PU1		87kb 5' of ZBTB40	
1	22365585	0.96	0.98	rs7537281	A	T	0.22	0.16	ESDR, SPLN	ESC, ESDR, IPSC, FAT, BRN, MUS, LNG, SPLN, BLD			Myc,NRSF	86kb 5' of ZBTB40	
1	22365822	0.96	0.98	rs12728589	G	C	0.24	0.16	ESDR, SPLN	ESC, ESDR, BRN, BLD	ESC,ESDR,IPSC,BLD	TBP,CHD2,EGR1,ZBTB33	BCL,NF-kappaB,TATA	86kb 5' of ZBTB40	
1	22370719	1	1	rs34414754	A	C	0.23	0.16	ESDR, PLCNT, LNG	ESC, IPSC, SKIN, ADRL, THYM, SPLN, LIV			AP-3,Nkx2,Nobox,Pax7,RXRA	81kb 5' of ZBTB40	
1	22371367	0.97	1	rs6679981	G	A	0.24	0.17	LNG, SPLN	IPSC, SKIN, ADRL, SPLN, LIV			AP-1,BDP1,Maf,Myc,NF-E2,NRSF,ZNF263	80kb 5' of ZBTB40	
1	22371954	1	1	rs7524102	A	G	0.24	0.16	LNG, SPLN	IPSC, ADRL, BRN, SPLN			LRH1,Pou5f1	80kb 5' of ZBTB40	
1	22373858	0.99	1	rs34920465	A	G	0.23	0.16	SPLN	ESDR, BRN				78kb 5' of ZBTB40	
1	22374653	0.98	0.99	rs11802513	C	A	0.23	0.16	SPLN	ESDR, FAT, ADRL, BRN			CEBPD,GR,Gmeb1,Hic1,Rad21	77kb 5' of ZBTB40	
1	22375232	0.99	1	rs34568051	C	T	0.23	0.16		IPSC, FAT, ADRL, BRN, GI			AP-4,Ascl2,E2A,HEN1,LBP-1,LUN-1	77kb 5' of ZBTB40	

1	22375268	0.99	1	rs12751610	T	C,G	0.23	0.16		IPSC, FAT, ADRL, BRN, GI				77kb 5' of ZBTB40	
1	22375584	0.99	1	rs12563939	C	A	0.23	0.16	ESDR	ESC, ESDR, IPSC, FAT, STRM, GI, ADRL, SPLN, MUS, SKIN			VDR	76kb 5' of ZBTB40	
1	22375694	0.99	1	rs34553872	A	T	0.23	0.16	ESDR, FAT	ESC, ESDR, IPSC, FAT, STRM, GI, ADRL, SPLN, MUS, SKIN	MUS,MUS,SKIN			76kb 5' of ZBTB40	
1	22375738	0.99	1	rs12568930	T	C	0.23	0.16	ESDR, FAT	ESC, ESDR, IPSC, FAT, STRM, GI, ADRL, SPLN, MUS, SKIN	MUS,MUS,SKIN		Nrf1,STAT	76kb 5' of ZBTB40	
1	22376542	0.99	1	rs10493013	T	C	0.23	0.16	SPLN	ESC, ESDR, IPSC, FAT, GI, ADRL, SPLN, MUS, SKIN	SKIN		AP-1,Mef2	75kb 5' of ZBTB40	
1	22377698	0.99	1	rs11799474	T	G	0.23	0.16		ESDR, IPSC, FAT, ADRL				74kb 5' of ZBTB40	
1	22379941	0.99	1	rs6684375	C	T	0.23	0.16	SKIN, PLCNT	ESC, ESDR, LNG, IPSC, FAT, BRST, SKIN, GI, ADRL, MUS, BRN	SKIN	NRSF	EBF,ZBTB7A	72kb 5' of ZBTB40	
1	22384384	0.99	1	rs34963268	G	C	0.23	0.16		ESDR, IPSC, ESC			ATF3,E2F,HEY1,Jundm2	67kb 5' of ZBTB40	
1	22384980	0.98	0.99	rs6426749	G	C	0.23	0.16		ESDR			AP-2,NF-I,Sin3Ak-20	67kb 5' of ZBTB40	

Note: variants in red are best GWAS SNPs in TWAS-identified loci.
